# Resistome, mobilome, and virulome explored in clinical isolates derived from acne patients in Egypt: unveiling unique traits of an emerging coagulase-negative *Staphylococcus* pathogen

**DOI:** 10.3389/fcimb.2024.1328390

**Published:** 2024-02-02

**Authors:** Mai A. Amer, Manal M. Darwish, Noha S. Soliman, Heba M. Amin

**Affiliations:** ^1^ Department of Microbiology and Immunology, Faculty of Pharmacy, October University for Modern Sciences and Arts, Giza, Egypt; ^2^ Medical Microbiology and Immunology Department, Faculty of Medicine, Ain Shams University, Cairo, Egypt; ^3^ Clinical and Chemical Pathology Department, Faculty of Medicine, Cairo University, Cairo, Egypt

**Keywords:** CoNS, acne, antibiotic resistance, virulence, genome analysis, mobilizable genetic elements, *Staphylococcus epidermidis*, *Staphylococcus warneri*

## Abstract

Coagulase-negative staphylococci (CoNS) are a group of gram-positive staphylococcal species that naturally inhabit the healthy human skin and mucosa. The clinical impact of CoNS-associated infections has recently been regarded as a challenge for diagnosis and therapeutic options. CoNS-associated infections are primarily caused by bacterial resistance to antibiotics and biofilm formation. As antibiotics are still the most used treatment, this problem will likely persist in the future. The present study aimed to investigate the resistance and virulence of CoNS recovered from various acne lesions and explore their genetic basis. Skin swab samples were collected from participants with acne and healthy skin. All samples underwent conventional culture for the isolation of CoNS, MALDI-TOF confirmation, antibiotic susceptibility, and biofilm formation testing. A total of 85 CoNS isolates were recovered from the samples and preliminarily identified as *Staphylococcus epidermidis*. Isolates from the acne group (n = 60) showed the highest rates of resistance to penicillin (73%), cefoxitin (63%), clindamycin (53.3%), and erythromycin (48%), followed by levofloxacin (36.7%) and gentamycin (31.7%). The lowest rates of resistance were observed against tetracycline (28.3%), doxycycline (11.7%), and minocycline (8.3%). CoNS isolated from mild, moderate acne and healthy isolates did not show strong biofilm formation, whereas the isolates from the severe cases of the acne group showed strong biofilm formation (76.6%). Four extensively drug-resistant and strong biofilm-forming staphylococcal isolates recovered from patients with severe acne were selected for whole-genome sequencing (WGS), and their genomes were investigated using bioinformatics tools. Three of the sequenced genomes were identified as *S. epidermidis*; however, isolate 29AM was identified as *Staphylococcus warneri*, which is a newly emerging pathogen that is not commonly associated with acne and was not detected by MALDI-TOF. All the sequenced strains were multidrug-resistant and carried multiple resistance genes, including *blaZ*, *mecA*, *tet(K)*, *erm(C)*, *lnuA*, *vgaA*, *dfrC*, *fusB*, *fosBx1*, *norA*, and *vanT*, which were found to be located on plasmids and chromosomes. Virulence features were detected in all genomes in the presence of genes involved in adherence and biofilm formation (*icaA*, *icaB*, *icaC*, *sdrG*, *sdrH*, *atl*, *ebh*, and *ebp*). Only the *S. warneri* isolate 29AM contained immune evasion genes (*capB*, *capC*, *acpXL*, and *manA*), an anti-phagocytosis gene (*cdsA*), and other unique features. As a result of their potential pathogenicity and antibiotic resistance, CoNS must be monitored as an emerging pathogen associated with acne infections. To the best of our knowledge, this is the first report to isolate, identify, and correlate *S. warneri* with severe acne infections among Egyptian patients using WGS and bioinformatic analysis.

## 1 Introduction

*Staphylococcus* species frequently colonize the skin of birds and mammals*. Staphylococcus* species are distinguished by their ability to coagulate blood into two main groups: coagulase-positive staphylococci, *Staphylococcus aureus*, and coagulase-negative staphylococci (CoNS), which include most species, such as *Staphylococcus epidermidis* (Otto, 2010). CoNS is a common skin microbiome organism and can inhibit the adhesion of virulent *S. aureus* and other pathogens (Christensen and Brüggemann, 2014). *S. epidermidis* is the most commonly isolated staphylococcal species from the human skin (Becker et al., 2014). It primarily colonizes the head, nose, and axilla. *Staphylococcus hominis* and *Staphylococcus capitis* are two additional common human skin colonizers. The latter is more commonly detected in the head and is more prevalent in adolescence. *Staphylococcus haemolyticus* and *Staphylococcus warneri* are less frequently observed in the human skin. Additionally, species that typically reside on farms or domestic animals, such as *Staphylococcus sciuri* or *Staphylococcus intermedius*, may transiently colonize humans (Otto, 2010). CoNS frequently exhibit multiple drug resistance, have few effective therapeutic choices, result in incurable diseases, and accumulate resistant strains in communities and hospitals. Antimicrobial resistance has various major causes, ranging from a lack of infection control to the inappropriate use of antibiotics. Recent investigations have shown that CoNS are highly resistant to erythromycin, vancomycin, oxacillin, methicillin, and penicillin (França, 2023). Scientists are paying attention to the emergence of multidrug-resistant (MDR) strains among CoNS and *S. warneri*, which is considered an emerging pathogen that can cause serious infections (Alawad et al., 2022).

*S. epidermidis* is commonly regarded as a commensal microorganism because it is beneficial to the skin in healthy environments (Cogen et al., 2008; Yang et al., 2022). Commensal *S. epidermidis* undergoes mutualistic and symbiotic interaction with the cutaneous system (Brüggemann, 2010). The skin hosts and supplements *S. epidermidis* with nutrients; in exchange, bacteria participate in host defense, innate immunity, and skin homeostasis. Through their microbial surface components, these bacteria interact with extracellular matrix proteins in the human skin (Arrecubieta et al., 2007; Brüggemann, 2010), which subsequently permits the interaction pathways between skin cells and bacteria (Yang et al., 2022). *S. epidermidis* can prevent biofilm formation of pathogenic strains via the secretion of bacteriocins (Paluch et al., 2020). Additionally, during the healing process of wounds or skin diseases, *S. epidermidis* lipoteichoic acid (LTA) can reduce skin inflammation (Fournière et al., 2020).

Although *S. epidermidis* plays a physiological role in maintaining skin homeostasis, it can be linked to some skin pathologies such as acne vulgaris, a prevalent chronic inflammatory skin condition affecting the pilosebaceous unit, where *S. epidermidis* was found to be overrepresented (Fitz-Gibbon et al., 2013; O’Neill and Gallo, 2018). Acne is primarily caused by three factors: (1) bacterial strains (Downing et al., 1986; Aydemir, 2014; Kutlu et al., 2023), *C. acnes* and *S. epidermidis* are both present, with *C. acnes* making up less than 2% of the skin surface bacteria and *S. epidermidis* being overrepresented (Fitz-Gibbon et al., 2013; O’Neill and Gallo, 2018); (2) qualitative and quantitative excessive seborrhea in sebaceous glands in acne lesions; and (3) keratinocytes with hyperkeratinization of the pilosebaceous unit, which causes the production of comedones, papules, and pustules (Jahns et al., 2012). The role of biofilms in acne development is an active area of research. Biofilms are complex communities of microorganisms that adhere to surfaces and produce a protective extracellular matrix. The formation of microbial communities, notably *Propionibacterium acnes* (*P. acnes*), as biofilms, contributes to the obstruction of follicles and the accumulation of sebum, dead skin cells, and bacteria. In this structured matrix, *P. acnes* exhibits increased resistance to antimicrobial agents and host immune responses, fostering inflammation and contributing to the characteristic inflammatory lesions seen in acne. The resilience of biofilms poses challenges in treatment because their protective nature makes bacteria less susceptible to conventional therapies, potentially leading to treatment resistance and recurrent lesions. The chronic nature of acne, characterized by periods of exacerbation and remission, may be linked to the persistence of biofilms, emphasizing the need for research on disrupting biofilm formation as a potential target for innovative acne management strategies (Chen et al., 2017; Coenye et al., 2022).

Notably, some phylogenetic sequence types (STs) of *S. epidermidis* (e.g., ST2, ST5, ST23, and ST215) are associated with nosocomial infections, raising the possibility of their pathogenicity owing to their virulence and multidrug resistance properties (Månsson et al., 2015; Lee et al., 2018). Several virulence genes play an essential role in adhesion, biofilm development, and phenol-soluble modulation, in addition to the presence of mobile genetic elements (MGEs) that are involved in the acquisition and transmission of virulence and resistance features that enhance the pathogenicity of *S. epidermidis* (Bouchami et al., 2016; Rolo et al., 2017). One of the most important virulence factors of *S. epidermidis* is biofilm formation, which is mediated by intercellular adhesin (*ica*) and accumulation-associated protein (*aap*) genetic determinants (Arciola et al., 2015; Schaeffer et al., 2016). Biofilms are recognized as a common form of microbial growth that confer protection against the host immune system and antibiotic challenges (França, 2023). In CoNS biofilms, especially in *S. epidermidis*, the polysaccharide poly-N-acetylglucosamine (PNAG) is one of the most dominant metabolites, and up to 60% of the recovered clinical isolates produce the proteins encoded by the *icaADBC* genes (Ahmed et al., 2019). The first staphylococcal component with a key role in biofilm accumulation was identified as polysaccharide intercellular adhesin (PIA). However, not all strains of *S. epidermidis* strains possess the icaADBC operon and are formed by the protein products of this gene (Formosa-Dague et al., 2016; Schaeffer et al., 2016). Horizontal gene transfer (HGT) is one of the most significant methods for CoNS to acquire exogenous DNA and, as a result, antibiotic resistance genes in biofilms (Águila-Arcos et al., 2017). In recent years, attention has been focused on the arginine catabolic mobile element (ACME) system, a pathogenicity island hypothesized to promote host colonization and immune evasion (O’Connor et al., 2018). ACME likely descends from *S. epidermidis* and spreads horizontally to *S. aureus* (Onishi et al., 2013; Planet et al., 2013).

Considering emerging evidence for the pathogenicity of some specific strains of CoNS, assuming that not all staphylococcal isolates behave similarly, it has been challenging to identify clinically significant strains. To this end, we aimed to characterize CoNS isolated from healthy skin and acne lesions phenotypically by testing antimicrobial susceptibility and biofilm production, and genetically characterized CoNS isolated from severe acne infections using whole genome sequence (WGS) technology in order to understand the genetic basis of bacterial virulence, antibiotic resistance, and phylogenetic background.

## 2 Materials and methods

### 2.1 Sample collection

The present study was conducted with a total of 140 participants, divided into two groups: i) the acne group (n = 100) with various levels of severity, including mild (n = 25), moderate (n = 45), and severe acne (n = 30), and ii) the healthy group with healthy skin (n = 40). All participants were university students in Egypt aged 18–24 years. The patients recruited in this study were all non-diabetic, not previously treated with topical antibiotics, did not previously receive acne treatment, and had no history of underlying medical illnesses. For the female participants, skin samples were not collected during the menstrual period.

The participant clinician classified the degree of acne based on the clinical features into mild, moderate, and severe acne. The mild level was *Acne comedonica* (comedones and congestion), moderate level was papulopustular acne (mild papules and pustules), and severe form was pustule-nodular acne (severe pustules and nodules).

All participants provided written informed consent, and the study was approved by the research ethics committee of the pharmacy faculty of the October University for Modern Sciences and Arts (MSA) (approval number (M1/Ec1/2022PD).

### 2.2 Isolation of bacteria and preliminary identification of CoNS

Skin swabs of acne lesions were collected from the participants. For the growth of bacterial colonies, skin swabs were cultured in tryptic soy broth (TSB) (Oxoid, UK) at 37°C for 3 days (Huang et al., 2022). Purification was performed by serial subcultivation on 5% sheep blood agar (SBA) (Oxoid, UK) and mannitol salt agar (MSA) (Oxoid, UK) at 37°C for 24 h to obtain single colonies in pure culture. Isolates obtained from the blood and MSA were preliminarily identified as CoNS based on colony morphology and conventional biochemical reactions. Microscopic characteristics were studied using Gram staining. Catalase, DNase, slide coagulase activity, motility, and blood agar hemolysis tests were conducted according to the method by Freney et al. (1999). Isolate identity was subsequently confirmed using Matrix-Assisted Laser Desorption/Ionization Time-of-Flight Mass Spectrometry (MALDI-TOF MS) (Dubois et al., 2010).

### 2.3 Antibiotic susceptibility tests

The Kirby–Bauer disc diffusion method was used to screen CoNS isolates for antibiotic susceptibility. The adjusted bacterial culture, equivalent to 0.5 McFarland standard, was inoculated onto Muller Hinton agar (Lab M, UK) and incubated at 37°C for 24 h. Commercial antibiotic discs (Oxoid, England) were tested: penicillin (10 U), cefoxitin (30 μg), erythromycin (15 μg), vancomycin (30 μg), clindamycin (2 μg), tetracycline (30 μg), trimethoprim/sulfamethoxazole (1.25/23.75 μg), gentamicin (10 μg), levofloxacin (5 μg), tetracycline (30 μg), rifampin (5 μg), minocycline (30 μg), and doxycycline (30 μg). The interpretative results matched the Clinical and Laboratory Standards Institute standards (CLSI) (CLSI, 2022).

### 2.4 Phenotypic characterization of biofilm production of CoNS isolates

The crystal violet assay was used to evaluate the ability of the staphylococcal isolates to form biofilms (Amer et al., 2021). Briefly, overnight cultures were adjusted to 10^5^ CFU/ml and inoculated in 200 μl of TSB supplemented with 1% (v/v) glucose (LOBA Chemie, India), in 96-flat bottom polystyrene microtiter plate (Greiner Bio-one^®^, Germany). The microtiter plates were incubated at 37°C without shaking for 24 h. After incubation, microbial growth was determined by measuring the turbidity at 600 nm, after which the cells were removed, washed twice with sterile phosphate buffered saline (PBS), and left to air dry. The dried biofilms were then stained with 0.1% crystal violet (CV) (LOBA Chemie, India) for 15 min, excess stain was removed, the wells were washed with 200 μl sterile distilled water, excess water was removed, and the plates were dried. Finally, the stain was solubilized in 33% glacial acetic acid (LOBA Chemie, India). Colorimetric analysis of the biofilm biomass was performed at 545 nm. Biofilm formation was evaluated using the biofilm formation index, [BFI]: (AB − CW)/(GB − GW), where AB is the OD_545_ of the CV-stained microorganisms, CW is the OD_545_ nm of the stained blank wells containing only media, GB is the OD_600_ of the cell culture, and GW is the OD_600_ of the blank well. Isolates were classified into four groups: nonadherent (0.35), mild (0.35 to 0.69), moderate (0.70 to 1.09), and strong (>1.10) biofilm forming according to the specified semi-quantitative biofilm production classification described by Lucero-Mejía et al. (2020).

### 2.5 DNA extraction and detection of *icaA*, *icaD*, and *mecA* genes

Bacterial pellets of CoNS strains were pretreated with 180 µl lysis buffer formulated with 20 mM Tris–HCl, pH 8.0, 2 mM EDTA (ADWIC, Egypt), 1.2% Triton X-100, and lysozyme that was added immediately before use, at a final concentration of 20 mg/ml. After pretreatment, genomic DNA was extracted using the QIAamp^®^ DNA Mini Kit (QIAGEN, Germany) according to the manufacturer’s recommendations for gram-positive bacteria.

The presence of *icaA*, *icaD*, and *mecA* in the extracted DNA was detected by polymerase chain reaction using forward and reverse primers, as shown in **Table 1**. The amplification reaction was performed according to the method described by Petrelli et al. (2006), using a Biometra DNA thermal cycler (Hamburg, Germany). Amplicons were analyzed by electrophoresis on 1% agarose gels (Lonza, USA), stained with ethidium bromide dye (Sigma-Aldrich, USA), and visualized under a UV light transilluminator (LTF Labortechnik, Germany). Ruler Gene 100 bp A DNA ladder served as a DNA size indicator (Zhou et al., 2013).

**Table 1:** List of primers used in PCR.

Primers	Sequence (5’-3’)	Target Gene	Tm (°C)	Length	References
**icaA_F**	TCT CTT GCA GGA GCA ATC AA	*icaA*	56.4	188	Zhou et al. (2013)
**icaA_R**	TCA GGC ACT AAC ATC CAG CA		58.4		
**icaD_F**	ATG GTC AAG CCC AGA CAG AG	*icaD*	60.5	198	Zhou et al. (2013)
**icaD_R**	CGT GTT TTC AAC ATT TAA TGC AA		55.5		
**mecA_F**	AAA ATC GAT GGT AAA GGT TG GC	*mecA*	58.4	244	Zhou et al. (2013)
**mecA_R**	AGT TCT GCA GTA CCG GAT TT GC		62.1		

### 2.6 Genomic characterization of *S. epidermidis* using whole genome sequencing

#### 2.6.1 Library preparation and sequencing

Following DNA extraction using the QIAamp^®^ DNA Mini Kit (QIAGEN, Germany), sequencing libraries were prepared using the Nextera XT DNA Library Preparation Kit (Illumina, USA) according to the manufacturer’s instructions. Quality control was performed using an Agilent DNA 1000 chip, prior to shotgun sequencing using MiSeq (Illumina, USA). Preassembly processing of the generated reads was performed using FastQC. Trimmomatic v0.32 was used to remove low-quality (mean quality less than 25) or low-complexity reads, reads mapping to the human genome or large and small ribosomal units of bacteria, fungi, and humans, and known contaminants (e.g., phiX174, Illumina spike-in) (Bolger et al., 2014). All genomes are available in the NCBI genome (BioProject accession number PRJNA993660).

#### 2.6.2 Genome assembly and annotation

Pre-processed reads were assembled *de novo* using SPAdes version 3.6.1, with contigs shorter than 1,000 nucleotides discarded (Bankevich et al., 2012). The analysis was only selected for reconstructed genomes with an N50  > 50,000. The assembled contiguous sequences were submitted via the National Center for Biotechnology Information (NCBI) Prokaryotic Genome Annotation Pipeline to GenBank for gene annotation. The generated contigs were further analyzed to investigate the genetic elements of interest. After that, the most similar sequences were identified and a phylogenetic tree was constructed using the Bacterial and Viral Bioinformatics Resource Center (BV-BRC) (https://www.bv-brc.org/) (Olson et al., 2023).

#### 2.6.3 Pathogenicity, resistome, and virulome analysis

PathogenFinder was used to predict the pathogenicity of the isolates for human hosts (https://cge.cbs.dtu.dk/services/PathogenFinder/). The assembled genomes obtained from the WGS data were annotated to identify the predicted resistome using ResFinder 4.1 (with a minimum length and threshold of 60% and 90%, respectively) (https://cge.cbs.dtu.dk/services/ResFinder/), and the Comprehensive Antibiotic Resistance Database (CARD) available at (https://card.mcmaster.ca/analyze/rgi) (Alcock et al., 2020), using the default selection criteria “perfect and strict hits only.” These platforms were employed in combination to avoid the drawbacks of each platform.

The genetic basis (chromosomal single-nucleotide polymorphism [SNP]) for fluoroquinolone and rifampicin resistance genes from the assembled genomes was studied (Altschul et al., 1990). Alignment of *gyrA*, *gyrB*, *parC*, *parE*, and *rpoB* genes to the reference strains of *S. epidermidis* (ATCC^®^12228 and ATCC^®^35984) with their corresponding genes from the assembled isolates in this study. Mutations were analyzed using multiple sequence alignment (MSA) and SNP analysis tool provided by the BV-BRC (https://www.bv-brc.org/). Clustal Omega (1.2.4) provided by EMBL’s European Bioinformatics Institute (EMBL-EBI) (https://www.ebi.ac.uk/Tools/msa/clustalo/) was used to perform MSA alignments of the predicted amino acid sequences. Colored alignments were visualized using the multiple alignment viewer tool MView 1.63, available at (https://www.ebi.ac.uk/Tools/msa/mview/) and hosted by EMBL-EBI.

VirulenceFinder 2.0 (using a minimum length of 60% and a threshold of 90%) (https://cge.cbs.dtu.dk/services/VirulenceFinder/) (Joensen et al., 2014), virulence factor database (VFDB) (http://www.mgc.ac.cn/cgi-bin/VFs/v5/main.cgi?func=VFanalyzer) and BacWGSTdb (http://bacdb.cn/BacWGSTdb) were used for screening the presence of virulence genes. The virulence determinants related to *S. epidermidis* were examined including adhesion, enzymes, biofilm formation, secretion, immune evasion, toxins, anti-phagocytosis, and intracellular survival.

#### 2.6.4 *In silico* multilocus sequence typing

Multilocus sequence typing (MLST) was performed *in silico* using MLST 2.0, which is available on the website of the Center for Genomic Epidemiology (https://cge.cbs.dtu.dk/services/MLST/) (Larsen et al., 2012) and the public molecular typing database PubMLST (https://pubmlst.org/).

Sequence types were assigned comparing the internal fragments of the seven housekeeping genes (*arcC*, *aroE*, *gtr*, *mutS*, *pyrR*, *tpiA*, and *yqiL*) from *S. epidermidis* to determine the MLST sequence types (STs) (Thomas et al., 2007).

#### 2.6.5 Identification of mobile genetic elements

Mobile genetic elements (MGEs) related to ARGs and their genomic context were investigated using NCBI annotations. The web-based typing tool SCC*mec*Finder was used to determine the SCC*mec* types and their structural position in the *S. epidermidis* isolates *in silico* (https://cge.cbs.dtu.dk/services/SCC*mec*Finder/). Plasmid replicon types were detected *in silico* using PlasmidFinder 2.1, available at (https://cge.cbs.dtu.dk/services/PlasmidFinder/) (Carattoli et al., 2014).

Prophage sequences within the assembled genomes were detected and annotated using PHASTER tool (https://phaster.ca/) (Arndt et al., 2016). Only “intact” prophage regions discovered by PHASTER were considered. The sites of the prophage regions were BLASTED against CARD to determine whether they included resistance genes. MobileElementFinder v1.0.3 was used to identify ISs and transposons flanking the resistance genes (Johansson et al., 2021), available at https://cge.cbs.dtu.dk/services/MGE/. NCBI annotations were used to investigate the support environment for resistance genes. Insightful Science’s SnapGene viewer software v5.1.3.1, was used to examine the context of resistance genes by visualizing annotated contigs software (http://www.snapgene.com). Insertion sequences (ISs) were identified using BLAST analysis against the NCBI nucleotide database. Resistance islands were predicted using the IslandViewer4 web tool (http://www.pathogenomics.sfu.ca/islandviewer/) (Li and Durbin, 2010).

#### 2.6.6 Clustered regularly interspaced short palindromic repeats/CRISPR-associated system, arginine catabolic mobile element, and restriction–modification system

CRISPRCasFinder tool available at (https://crisprcas.i2bc.paris-saclay.fr/CrisprCasFinder/Index) utilizes the default advanced parameters for CRISPR and the clustering model “SubTyping” for Cas to search the genomes for clustered regularly interspaced short palindromic repeats (CRISPR) and cas genes.

The restriction–modification system (R–M system) was detected using a minimum length of 60% and %ID threshold of 95% using Restriction–ModificationFinder 1.1, available at (https://cge.cbs.dtu.dk/services/Restriction-ModificationFinder/) (Camacho et al., 2009). ACME genes were identified and mapped within the genomes. The arc, opp3, and kdp operons were used to align the ACME components, which were then classified as arc and opp3 operons (type I), arc operons alone (type II), opp3 operons alone (type III), arc and kdp operons (type IV), and arc, opp, and kdp operons (type V).

#### 2.6.7 Phylogenetic analyses using WGS-SNP and WGS-MLST trees

CSIPhylogeny (https://cge.cbs.dtu.dk/services/CSIPhylogeny/) was used to construct phylogenetic trees based on the maximum likelihood method of concatenated alignment of high-quality SNPs, which uses assembled contigs to perform SNP calling, SNP filtering, and phylogeny inference (Kaas et al., 2014). The analysis was performed on a platform using default parameters. The assembled genomes have been uploaded for comparison. To compare our isolates to *S. epidermidis* and *S. warneri* genomes available on the BV-BRC website, we searched and downloaded the *S. epidermidis* and *S. warneri* genomes and included them in the analysis. Figtree program was used to edit and visualize the phylogenetic tree (http://tree.bio.ed.ac.uk/software/figtree/).

The core genome single nucleotide polymorphism (cgSNP)-based phylogenetic tree was visualized using the interactive tree of life (iTOL) web tool v6.7 (https://itol.embl.de/itol.cgi) associated with the isolates, and other genomic information, and antibiotic resistance determinants. A phylogenetic tree was also constructed using the isolates and closely related genomes derived from BV-BRC (https://www.bv-brc.org/).

#### 2.6.8 Accession numbers

The assembled draft genomes from the Whole Genome Shotgun project were uploaded to the GenBank database under BioProject number PRJNA993660.

### 2.7 Statistical analysis

GraphPad Prism 8.0.0 for Windows (GraphPad Software Inc., CA, USA) was used for the statistical analysis. The independent samples t-test and two-way analysis of variance (ANOVA) were used to compare the antibiotic resistance determinants, biofilm-forming ability, and prevalence of biofilm-associated genes among CoNS isolates, with a *P*-value of 0.05 regarded as statistically significant.

## 3 Results

### 3.1 Clinical data and bacterial isolates

A total of 140 participants were enrolled in this study and divided into two groups: i) the acne group (n = 100) with various levels of severity, including mild (n = 25), moderate (n = 45), and severe acne (n = 30), and ii) the healthy group with healthy skin (n = 40). Sex distribution was represented in the form of 60 (60%) males and 40 (40%) females in the acne group, and 25 (62.5%) males and 15 (37.5%) females in the healthy group.

The culture of skin swabs yielded a total of 85 staphyloccal isolates that were preliminarily identified based on conventional biochemical reactions, all of which were gram-positive arranged in grape-like clusters, catalase-positive, DNase-negative, coagulase-negative, non-motile, and non-hemolytic on blood agar. All isolates were identified as *S. epidermidis* by MALDI-TOF MS. Sixty isolates were obtained from the acne group (60%), and 25 isolates (62.5%) were obtained from the healthy group.

### 3.2 Antibiotic susceptibility testing

**Figure 1:**
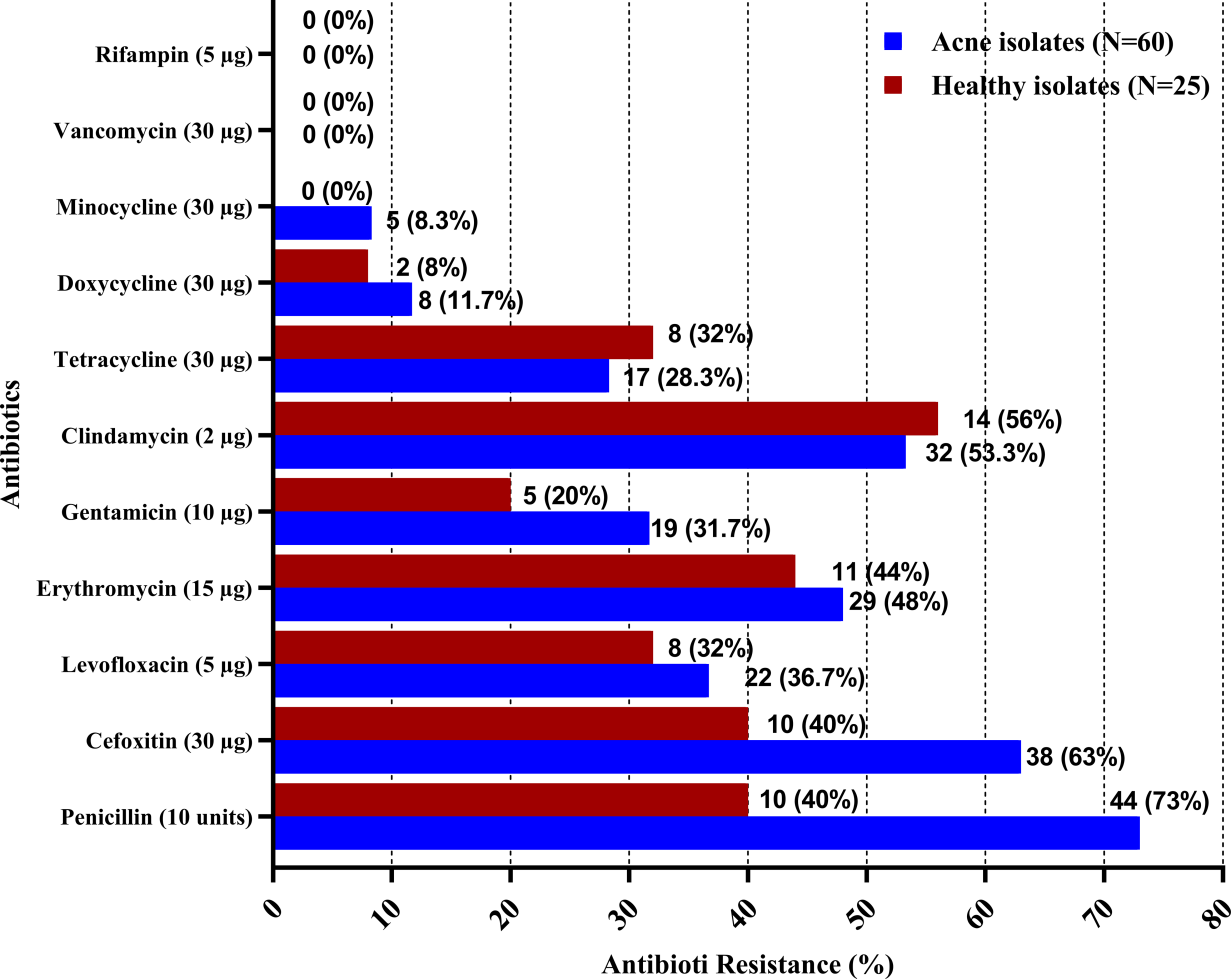
Antibiotic resistance among CoNS isolates from acne and healthy groups. Antibiotic susceptibility testing was performed for CoNS isolates using the Kirby–Bauer disk diffusion method, in accordance with the Clinical and Laboratory Standards Institute (CLSI).

The results of antimicrobial susceptibility testing of CoNS isolates from the acne and healthy groups are displayed in **Supplementary Table 1**, where isolates from the acne group (N = 60) showed the highest rates of resistance to penicillin (73%), cefoxitin (63%), clindamycin (53.3%), and erythromycin (48%), followed by levofloxacin (36.7%) and gentamycin (31.7%), while least rates of resistance were observed for tetracycline (28.3%), doxycycline (11.7%), and minocycline (8.3%). The antimicrobials with the highest rates of resistance among the healthy group isolates were clindamycin (56%), erythromycin (44%), penicillin (40%), and cefoxitin (40%), followed by levofloxacin (32%) and tetracycline (32%), whereas those with the lowest rates of resistance were gentamycin (20%), doxycycline (8%), and minocycline (0%). Comparing the resistance prevalence between the two groups of isolates showed a statistically significant difference (*P*-value <0.05) for penicillin and cefoxitin, however, no significant difference was observed for the other antimicrobials (**Supplementary Table 1**) (**Figure 1**). Out of the acne group, 15 isolates (25%) demonstrated multidrug resistance (MDR) to antibiotics, four of which were extensively drug resistant (XDR), whereas in the control group, two isolates exhibited resistance to multiple drugs (8%), and none were XDR. Statistical analysis revealed a significant difference between the two groups (*P*-value <0.05).

### 3.3 Phenotypic characteristics of biofilm production of CoNS isolates

**Figure 2:**
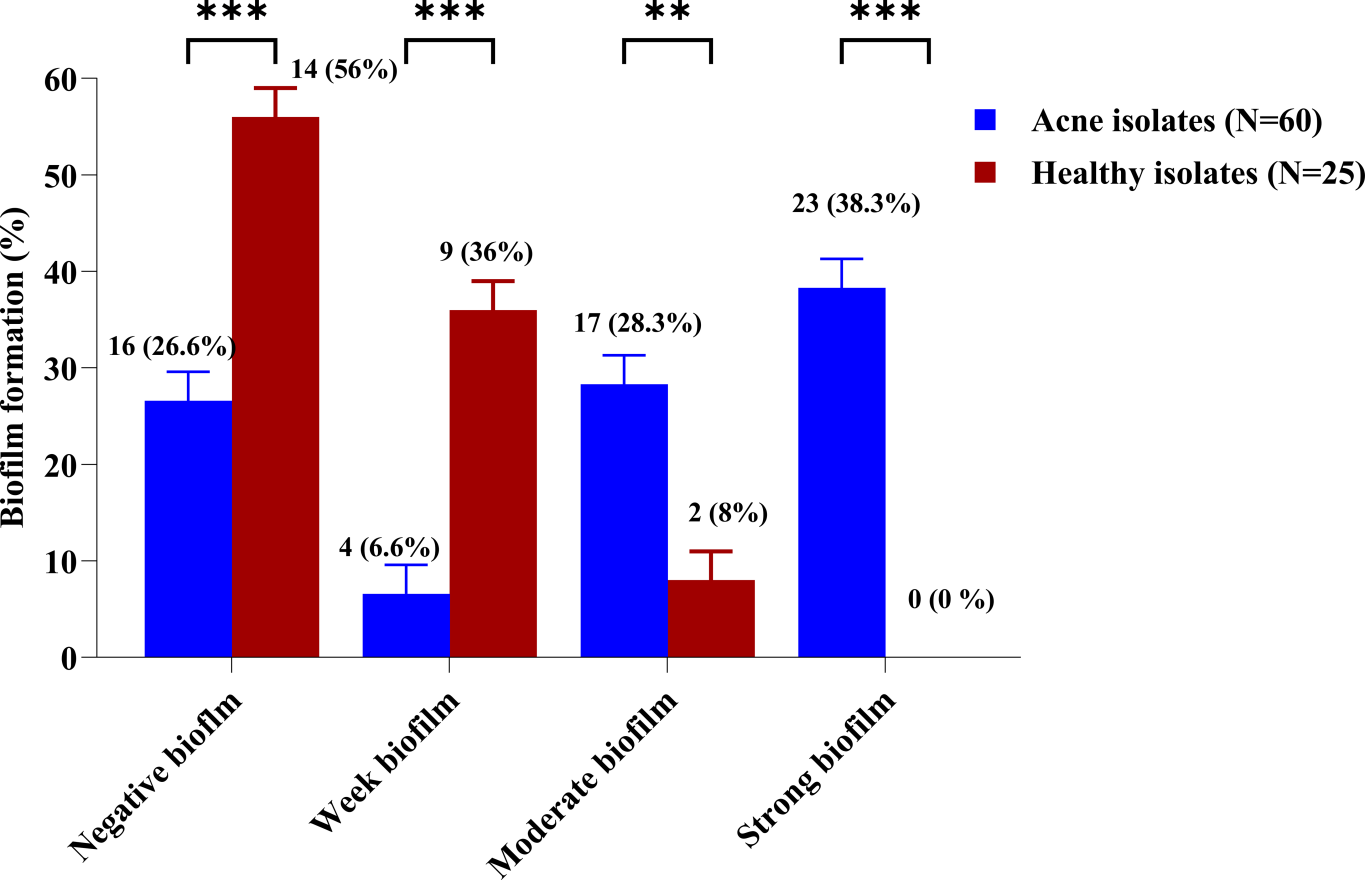
Biofilm formation percentage among CoNS isolates from the acne and healthy groups. The biofilm-forming ability of the CoNS isolates was assessed using a crystal violet assay. Adjusted overnight cultures were inoculated in 200 μl of TSB supplemented with 1% (v/v) glucose, in a 96-flat bottom polystyrene microtiter plate. The biofilms were then washed and stained with 0.1% crystal violet. The biofilm biomass was measured colorimetrically at 545 nm and evaluated using the biofilm formation index. Statistical analysis using two-way ANOVA, which was followed by multiple comparisons test with a significance level at ** p < 0.01; *** p < 0.001.

Biofilm formation ability of CoNS isolates was tested using crystal violet biofilm assay method. Forty-four isolates recovered from the acne group (73.3%) and 11 isolates recovered from the healthy group (44%) were able to form biofilm. Biofilm-producers demonstrated strong, moderate, and weak biofilm production at rates of 38.3%, 28.3%, and 6.6% among CoNS isolates of the acne group (n=60), while rates of 0%, 8%, and 36% among CoNS isolates of healthy group (n=25), respectively (**Supplementary Table 2**). The utilization of an unpaired t-test in statistical analysis indicated a significant contrast between the acne and healthy groups. Notably, only isolates retrieved from the severe acne group exhibited the ability to form strong biofilms (76.6%). All MDR isolates recovered from the acne group formed strong biofilms. (**Supplementary Table 2**) (**Figure 2**).

### 3.4 Molecularly detected *icaA*, *icaD*, and *mecA* genes

**Figure 3:**
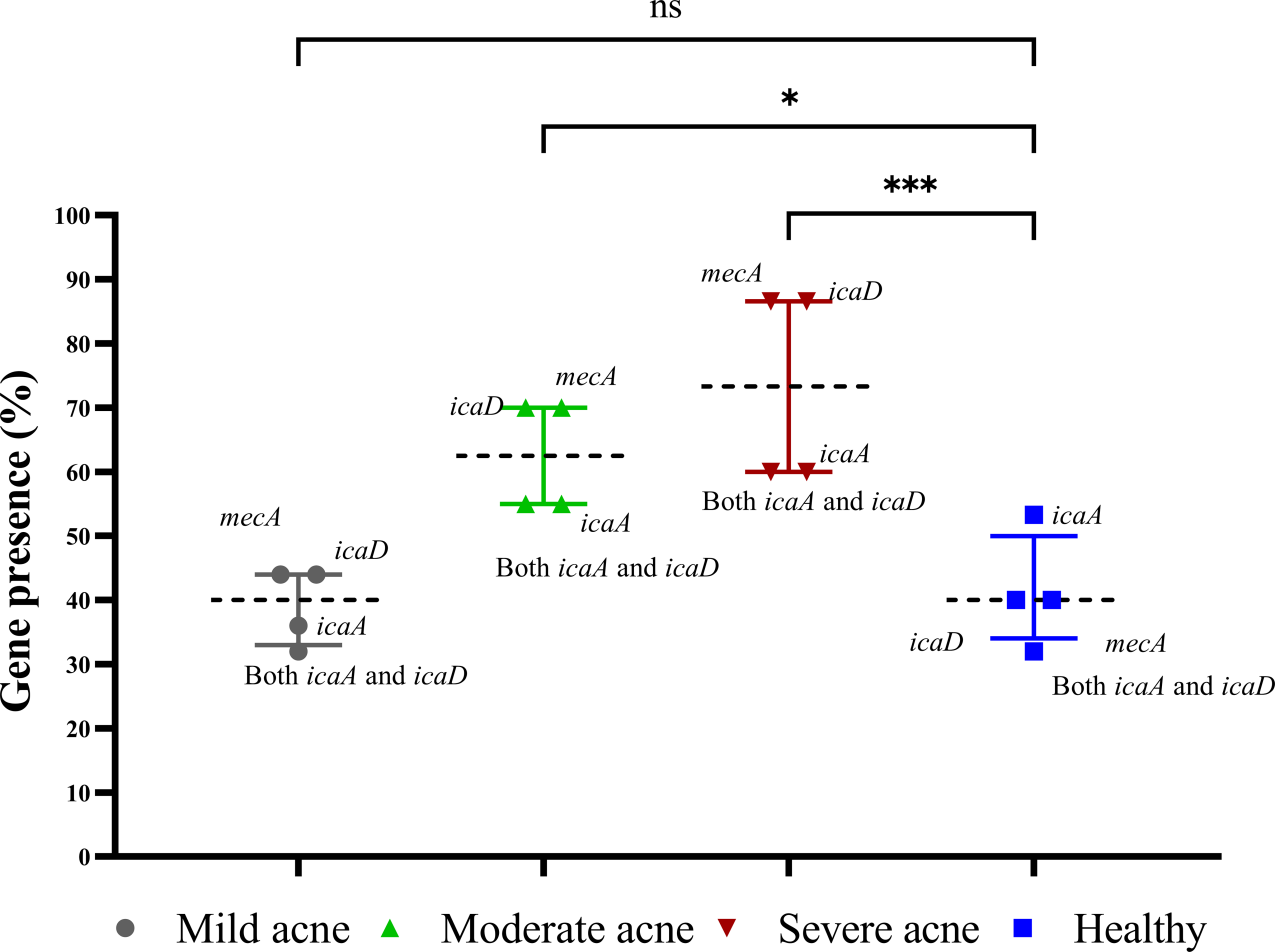
Prevalence of biofilm genes among CoNS isolates. The presence of *icaA*, *icaD*, and *mecA* in the extracted DNA was detected by polymerase chain reaction. The presence of the gene was represented as a percentage of the different acne and healthy groups, and the results are shown as medians with interquartile ranges. Statistical analysis using two-way ANOVA, which was followed by multiple comparisons test with a significance level at * p < 0.05; *** p < 0.001. ns, non-significant.

Biofilm-associated gene carriage was assessed in the isolates recovered from acne and healthy samples. The distribution of the biofilm-associated genes is shown (**Figure 3**). Two-way ANOVA in statistical analysis revealed a significant difference in the prevalence of biofilm-associated genes among CoNS isolates obtained from individuals with moderate and severe acne compared to those from mild acne and healthy individuals (*P*-value <0.05). Four XDR and strong biofilm-forming CoNS isolates were coded as 29AM, 36AM, 48AF, and 54AF were selected for further study using WGS. The antibiotic resistance characteristics of the four isolates are presented in **Table 2**.

**Table 2:** The antibiotic susceptibility profile and accompanying metadata for the four staphylococcal isolates.

Isolate Sex Source Sample			Biofilm formation	FOX	PEN	LEV	ERY	CHL	TET	DOX	MIN	CLI	RIF	SXT	VAN
36AM	M	Acne	Strong	R	R	R	R	R	R	R	R	R	S	R	S
29AM	M	Acne	Strong	R	R	R	R	R	R	R	R	I	S	R	S
48AF	F	Acne	Strong	R	R	R	R	R	R	R	R	R	S	R	S
54AF	F	Acne	Strong	R	R	R	R	R	R	R	R	R	S	R	S

The CLSI breakpoints for CoNS were used to interpret antibiotic susceptibility tests. R, resistant; I, intermediate; S, susceptible; M, male; F, female. FOX, cefoxitin; PEN, penicillin; LEV, levofloxacin; ERY, erythromycin; CHL, chloramphenicol; TET, tetracycline; DOX, doxycycline; MIN minocycline; CLI, clindamycin; RIF, rifampin; SXT, sulfamethoxazole/trimethoprim; VAN, vancomycin.

### 3.5 Genomic characterization of the selected CoNS isolates using WGS

#### 3.5.1 Genome and assembly features, as well as resistome characterization

Based on the short-read sequences, draft genomes comprising 65–294 contigs were assembled with a mean N50 of 80,333 bp, covering approximately 81%–86% of the reference genome. **Supplementary Table 3** displays the genome sequences and assembly parameters, such as size, number of contigs, number of RNAs, guanine–cytosine (GC) content (%), number of coding sequences, N50, and L50.

The isolates’ draft genome size ranged from 2.3 Mb to 2.6 Mb, with a GC content of 31.88% to 32.44%. Three isolates coded as 36AM, 48AF, and 54AF were confirmed to be *S. epidermidis* using WGS, while the isolate coded as 29AM was identified as *S. warneri*, another multidrug-resistant CoNS.

Antibiotic resistance genes (ARGs) conferring resistance to β-lactams (*blaZ*), methicillin/oxacillin (*mecA*), glycopeptides (*vanT*, *vanY*), fluoroquinolone [*norA*, *norC*, *sdrM*], tetracyclines [*tet(K)*], macrolide–lincosamide–streptogramin B antibiotic (MLSB) [*erm(C)*, *vgaA*, and *InuA*], trimethoprim-sulfamethoxazole (*dfrC*), aminocoumarin (*gyrB*), fosfomycin (*fosBx1*), and fusidic acid (*fusB*) were detected in the isolated genomes (**Table 3**). All staphylococcal isolates except isolate 54AF possessed the *blaZ* gene, while all isolates except *S. warneri* 29AM possessed the *mecA* gene. ARGs related to trimethoprim-sulfamethoxazole-, tetracycline-, doxycycline-, and erythromycin-resistance followed resistance phenotypes. Isolates phenotypically resistant to these antibiotics and their corresponding ARGs were detected in the genomic context.

**Table 3:** Genotypic characteristics of *Staphylococcus* isolates.

Isolates	Resistance genes (plasmid/chromosomal- mediated)	Plasmid replicon type	R–M system	*SCC*mec* type	ACME type	MLST	Insertion sequences	CRISPR-Cas elements	Pathogenicity score (no. of pathogenic families)
29AM	*blaZ*, *erm(C)*, *fusB*, *vanT vanY*, *vanW*, *dfrC*, *tet(k)*, *vgaA*, *sdrM*, *FosBx1*, *mdeA*, *sepA*, *gyrB*	rep13, rep7a, repUS35, rep10, rep5b, rep5d, rep10b, rep20, rep21, repUS9	–	–	–	Unknown^#^	IS256, ISSEP2	4 (2)	0.909 (31)
36AM	*mecA*, *blaZ*, *norA*, *norC*, *vanT vanY*, *dfrC*, *rpoC*,	rep13, rep5b, rep20, rep21, rep7a	–	SCC*mec* type V (5C2)	–	ST39	ISSep3, ISSep2	5 (3)	0.955 (86)
	*sdrM*, *FosBx1*, *mdeA*, *qacJ*, *lnuA*, *gyrB*, *sav1866*, *vgaA*								
48AF	*mecA*, *blaZ*, *norA*, *fusB*, *norC*, *sepA*, *sdrM*, *vanT*, *dfrC*, *mdeA*, *qacJ*, *gyrB* *blaZ*, *fosB*, *fusB*, *vga(A)*, *qacC*	rep13, rep7a, rep5b	–	SCC*mec* type II (A2)	–	ST87	–	5 (1)	0.945 (488)
54AF	*mecA*, *sepA*, *sdrM norA*, *norC*, *dfrC*, *vanT FosBx1*, *vanY*, *InuA*, *fosB*	rep7a, rep20	–	SCC*mec* type V (2B)	Present IVa	ST719	ISSep3, ISSep2	5 (3)	0.946 (150)

ACME, arginine catabolic mobile element; R–M system, restriction-modification system. ACME types I and II (arc and opp3 operons), III (opp3 operons only), IV (arc and kdp operons), and V (arc, opp, and kdp operons). Pathogenicity score: PathogenFinder predicts a bacteria’s pathogenicity to human hosts. Closet pathogenic family strain linkage: MLST, multilocus sequence typing; CRISPR-Cas, clustered regularly interspaced short palindromic repeats/CRISPR-associated; S. epidermidis ATCC^®^12228. The SCCmecFinder was used to predict SCCmec type.

The table represents the resistome and mobilome determinants detected by the bioinformatics analysis to the sequenced genomes.

*SCCmec typing was determined with the SCCmecFinder.

^#^Unknown indicates that a sequence type could not be determined for the isolate due to the absence of alleles in the draft genomes.

The four isolates showed agreement between the cefoxitin-resistant phenotype and either *mecA* or *blaZ* gene. The tetracycline-resistance gene *tet(K)* was only found in isolate 29AM, although all the tested isolates were phenotypically resistant to tetracycline.

Aminoglycoside resistance mechanisms were absent in all isolates, and none of the isolates were phenotypically resistant to gentamicin or amikacin. Furthermore, the MLSB resistance mechanism *erm (C)* was only detected in isolate 29AM, *vga (A)* was detected in all isolates except 54AF, *lnu (A)* was also detected in 36AM and 54AF isolates, and *dfrC* was detected in all isolates except 36AM. Antibiotic efflux pump genes (*norA*, *norC*, *sdrM*, *mdeA*, and *qacC*) were detected, which can also provide fluoroquinolone and macrolide resistance.

Mutations in *gyrA*, *gyrB*, *parC*, *pare*, and *rpoB* in *S. epidermidis* isolates compared to *S. epidermidis* ATCC^®^12228 were manually curated (**Table 4**). MSA of the predicted amino acid sequences of the genes carried by *S. epidermidis* isolates and close genomes retrieved from the BV-BRC database (**Supplementary Table 5**) compared to that of *S. epidermidis* strain ATCC^®^12228 was performed and visualized using M.View (**Supplementary Figures 1–5**). Moreover, MSA of *S. warneri* 29AM with the closely related *S. warneri* strains retrieved from the BV-BRC database (**Supplementary Table 6**) was also performed (**Supplementary Figures 6–10**), and there was no standard strain of *S. warneri*, so the gene mutation could not be detected. The genome of isolate 36AM possessed eight mutations in *gyrA*, two mutations in *gyrB*, seven mutations in *parC*, and two mutations in *pare*, but no mutations were detected in *rpoB*. The gemome of isolate 48AF did not harbor any mutations. Isolate 54AF contained eight mutations in *gyrA*, one in *gyrB*, seven in *parC*, four in *parE*, and one in *rpoB*.

**Table 4:** *S. epidermidis* isolates mutations in *gyrA*, *gyrB*, *parC*, *parE*, and *rpoB* genes.

Isolate	*gyrA*	*gyrB*	*parC*	*parE*	*rpoB*
36AM	T700N, N811K, A820V, S826T,T828N, T829A, T831K, M854T	V7OI, V221I,	K236R, K272R, K639N, E641K, S650T, I761V, N772K	N404S, K568N	–
48HF	–	–	–	–	–
54HM	N811K, A820V, S826T,T828N, T829A, T831K, M854T, E862K	V7OI	K236R, K272R, K639N, E641K, S650T, I761V, N772K	L373I*, N404S, K568N, I575T	Y737S

*Putatively novel mutations.

#### 3.5.2 Detected pathogenicity and virulome in the *Staphylococcus* genomes

**Figure 4:**
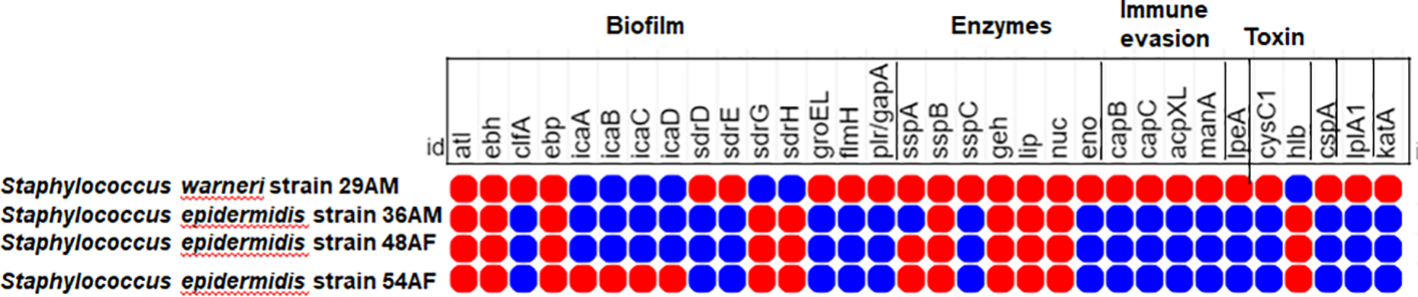
Heatmap showing the distribution of different virulence determinants among the sequenced isolates in the current study. The red circles denote the presence of virulence genes, whereas the lack of virulence genes is denoted by blue circles. The figure was created using the MORPHEUS online tool (https://software.broadinstitute.org/morpheus).

PathogenFinder estimated that the mean chance of isolates being pathogenic to humans ranged from 0.909 to 0.955 and matched numerous pathogenic families. Virulome analysis showed probable virulence genes encoding proteins from numerous *S. epidermidis* virulence categories, including adherence/biofilm production, enzymes, immune evasion, invasion, toxin, anti-phagocytosis, intracellular survival, and stress adaptability (**Figure 4**). Interestingly, the *S. warneri* isolate 29AM exclusively exhibited a higher number of virulence genes associated with acid resistance (*ureB*, *ureG*), anaerobic respiration (*narH*), antiphagocytosis (*cspA*), immune evasion (*capB*, *capC*, *acpXL*, *manA*), intracellular survival (*lplA1*), invasion (*lpeA*), iron uptake (*fagC*, *hemL*, *vctC*), lipid and fatty acid metabolism (*panD*), phagosome arresting (*ndK*), regulation (*lisR*, *sigA/rpoV*), stress adaptation (*katA*), and surface protein anchoring (*lgt*).

#### 3.5.3 WGS-based multilocus-sequence typing

*In silico* MLST analyses identified three different MLST types: sequence type (ST) isolate 36AM: ST39, isolate 48AF: ST87, and isolate 54AF: ST719. Isolate 29AM was not identified by MLST. The most resistant isolate belonged to ST87 and contained 16 ARGs encoding for resistance to multiple antibiotic drug classes (**Table 3**).

#### 3.5.4 Mobilome and the genetic support environment

*In silico* SCC*mec* typing/subtyping revealed three SCC*mec* types/subtypes, isolate 36AM belongs to SCC*mec* type V(5C2), isolate 48AF belongs to SCC*mec* type II (A2), and isolate 54AF belongs to SCC*mec* type V(2B),

PlasmidFinder and BacWGSTdb plasmid analysis showed ten distinct plasmid replicon types. Rep13 (3), rep7a (4), rep5a (3), and rep20 (3) were the most common types of plasmid replicons. Replicon types repUS35, rep10, rep5d, rep10b, rep21, repUS9 were only found in *S. warneri* 29AM.

ISs were detected in all tested isolates, except for isolate 48HF. Three distinct IS types from three distinct IS families were identified. IS families were IS256, 1S110, and IS200/IS605. IS256, family is well known to be associated with biofilm formation and virulence. IS256 family was found in *S. warneri* 29AM only. Furthermore, the resistance gene *erm(C)* was discovered exclusively in conjunction with transposon Tn554 in one isolate (29AM). The NCBI annotation of isolates 29AM, 36AM, and 48AF identified the presence of the *blaZ* gene flanked by the regulatory genes *blaR1* and *blaI*; however, this configuration was absent in isolate 54AF. The genetic context of the specific resistance genes investigated in this study was analyzed with a focus on illustrating the interplay between Mobile Genetic Elements (MGEs), ARGs, and virulence genes (**Table 5**).

**Table 5:** MGEs in *Staphylococcus* strains linked to antibiotic resistance genes.

Isolate	Contig	MGEs	Closest nucleotide homology between a plasmid/chromosome sequence (accession number)
**29AM**	14	*fusB*	*S. aureus* plasmid pUB101 (AY373761)
	20	IS256	*S. aureus* transposon 4001 *aacA-aphD* aminoglycoside resistance gene, complete cds, and right and left IS256 transposase gene (M18086)
	24	*bla(Z)*	*Streptococcus pneumoniae* genome assembly (CVLS01000543)
	29	rep5b	*S. epidermidis* plasmid SAP106B (GQ900455)
	31	repUS35	*S. warneri* SG1 plasmid clone pvSw2 genomic sequence (CP003671)
	34	rep5d	*S. aureus plasmid pJE1 remnant of replication protein Rep (rep), trimethoprim resistance protein* DfrA *(dfrA), thymidylate synthetase* ThyE *(thyE), and putative transposase* Tnp *(tnp) genes* (AF051916)
	36	rep5d	*S. aureus* plasmid pJE1 remnant of replication protein Rep (*rep*), trimethoprim resistance protein DfrA (*dfrA*), thymidylate synthetase ThyE (*thyE*), and putative transposase Tnp (*tnp*) genes, complete cds; and unknown gene (AF051916)
	37	*vga(A)* *rep7a*	*S. aureus* plasmid ATP-binding protein (*vga*) gene (M90056) *S. aureus* DNA, type-III staphylococcal cassette chromosome *mec* and SCCmercury: strain 85/2082 (AB037671)
	46	*tet(K)*	*S. aureus* tetracycline resistance plasmid pKH1, *tet* gene (U38656)
	48	repUS22	*S. aureus* plasmid SAP015B fragment (GQ900502)
	59	ISSep2	*S. epidermidis* ATCC *12228* (NC_004461)
	60	rep21	*S. aureus* plasmid pWBG754 (GQ900396)
	62	*erm(C)* *rep10*	*Bacillus subtilis* plasmid pIM13 (M13761) *S. aureus* strain E14 plasmid pDLK1 (GU562624)
	73	rep13	*S. aureus* plasmid pSK41 (AF051917)
	74	rep10b	*S. aureus* plasmid pSK6 (U96610)
	82	rep13	*S. aureus* strain WBG4364 plasmid pWBG1773 (EF537646)
**36AM**	48	ISSep3	*S. epidermidis* ATCC 12228 (NC_004461)
	78	*rep20* *blaZ*	*S. saprophyticus* subsp. *saprophyticus* MS1146 plasmid pSSAP2 (HE616681) *S. haemolyticus* plasmid NVH96 plasmid (AJ302698)
	87	*fosB*	*S. epidermidis* RP62A (CP000029)
	90	ISSep2	*S.epidermidis* ATCC 12228 (NC_004461)
	205	*rep5b*	*S. epidermidis* ATCC 12228 plasmid pSE-12228-06 (AE015935)
	206	*rep13* *qacC*	*S. epidermidis* CH plasmid pSepCH (AY092027) *S. aureus* replication (*rep*), control of replication (*cop*), and resistance protein (*QacC*) genes (M37889)
	223	*rep21* *lnu(A)*	*S. aureus* plasmid pWBG754 (GQ900396) *S. haemolyticus linA* gene encoding lincosamide resistance (M14039)
	276	*rep7a*	S*taphylococcus hyicus* plasmid pSTE1 (HE662694)
	376	*Tet(K):TPA*	*S. warneri* strain 16A plasmid (CP031268.1)
	60	rep21	*S. aureus* plasmid pWBG754 (GQ900396)
	73	rep13	*S. aureus* plasmid pSK41 (AF051917)
	74	rep10b	*S. aureus* plasmid pSK6 (U96610)
	82	rep13	*S. aureus* strain WBG4364 plasmid pWBG1773 (EF537646)
**48AF**	7	*fusB*	*S. aureus* plasmid pUB101 (AY373761)
	15	blaZ	*S.epidermidis* strain Mt1p16 Mt1p16_contig_78 (MAJJ01000071)
	16	*fosB*	*S. epidermidis* RP62A (CP000029)
	27	*ACME*	*S. aureus* strain USA300_R114 SCC*mec* IVa and ACME genetic islands (KF175393)
	37	rep7a	Glutathione S-transferase%2C unnamed subgroup 2 *[Klebsiella pneumoniae]* (SAU83488)
	38	rep5b	*S. epidermidis* plasmid SAP108B (GQ900459)
	39	rep7a	Glutathione S-transferase%2C unnamed subgroup 2 *[K. pneumoniae]* (SAU83488)
	46	rep13 *qacC*	*S. epidermidis* CH plasmid pSepCH (AY092027) *S. aureus* replication (rep), control of replication (cop), and resistance protein (QacC) genes (M37889)
	49	*vga(A)LC*	*S. haemolyticus* lincosamide-streptogramin A resistance protein (*vga(A)*LC) gene (DQ823382)
	*73*	rep7a	Glutathione S-transferase%2C unnamed subgroup 2 *[K. pneumoniae]* (SAU83488)
**54AF**	8	ISSEP2	*S. epidermidis* ATCC 12228 (NC_004461)
	10	*fosB*	*S. epidermidis* RP62A (CP000029)
	15	*ACME* ISSEP3	*S. aureus s*train USA300_R114 SCC*mec* IVa and ACME genetic islands (KF175393) *S. epidermidis* ATCC 12228 (NC_004461)
	16	rep20	*S. saprophyticus* subsp. *saprophyticus* MS1146 plasmid pSSAP2 (HE616681)
	35	*lnu (A)*	*S. haemolyticus linA* gene encoding lincosamide resistance (M14039)
	45	rep7a	*S. hyicus* plasmid pSTE1 (HE662694)

MGEs, mobile genetic elements.

The PHASTER tool discovered intact prophages incorporated into the genomes of isolates 29AM and 54AF; no phages were detected in isolates 36AM and 48AF. The most common prophage was PHAGE_Staphy_StB12 (n = 4). No resistance genes were found in any of the prophages. The characteristics of the prophages, such as GC content and the number of coding sequences, are shown in **Supplementary Table 4**.

### 3.6 Identification and classification of clustered regularly interspaced short palindromic

Repeats/CRISPR-Associated Elements, Arginine Catabolic Mobile Element (ACME), and Restriction–Modification (RM) systems. CRISPRCasFinder detects CRISPR sequences. Each isolate contained at least one CRISPR sequence. CRISPR-associated (Cas) genes were found in all isolates. None of the isolates contained an R–M system. ACME was identified in *S. epidermidis* isolates 48AF and 54AF and was classified as type IV (**Table 3**).

### 3.7 Phylogenetic relatedness of the study isolates with their closely related *Staphylococcus* strains in genomic database

**Figure 5:**
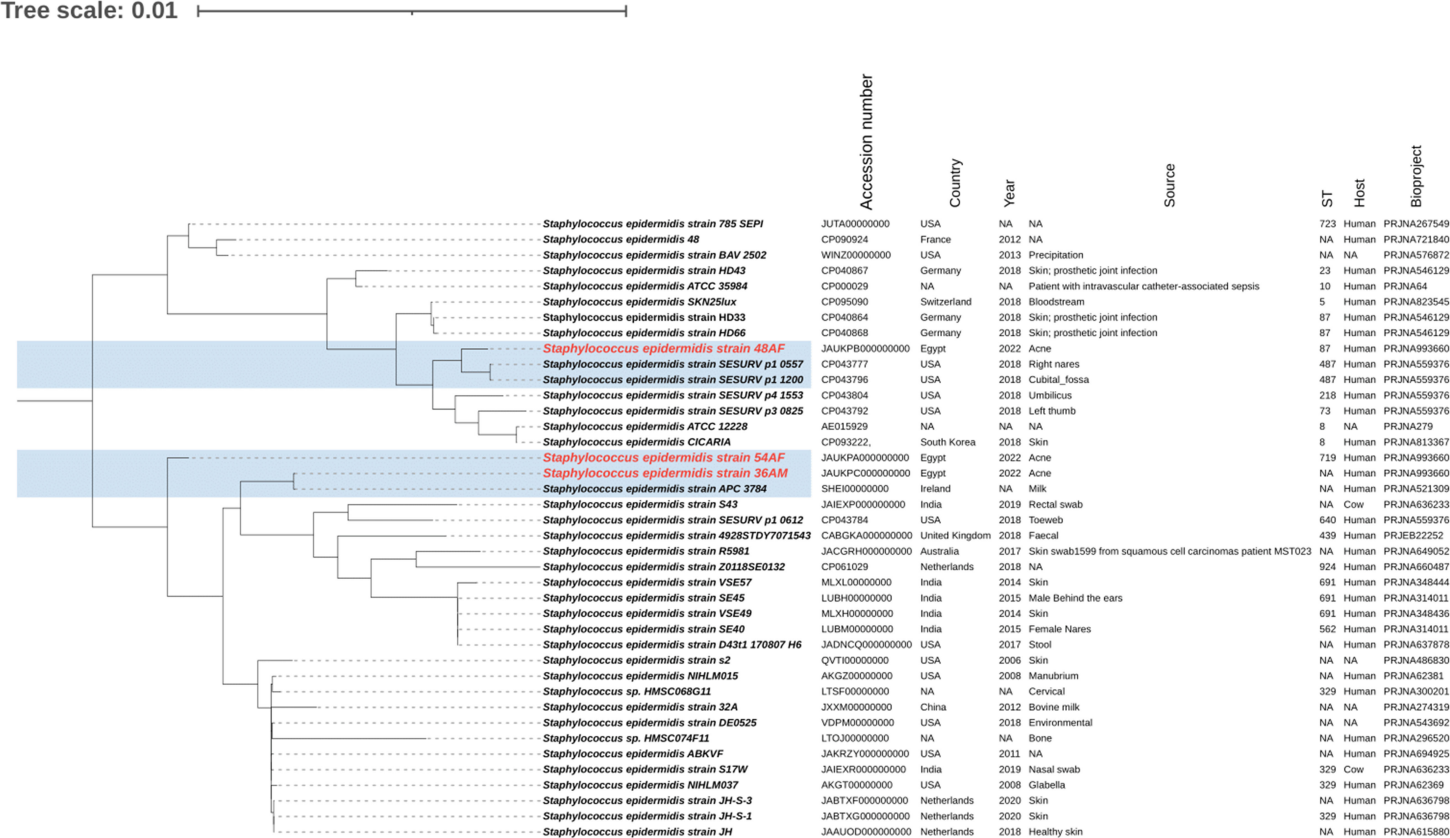
Whole genome-based phylogenetic tree of *S. epidermidis* isolates. The *S. epidermidis* genome sequenced in the current study was compared to the closely related genomes, highlighted in blue. The labels of the *S. epidermidis* strains sequenced in the current study are shown in red. The figure was created using the iTOL online tool v6.7 (https://itol.embl.de/).

**Figure 6:**
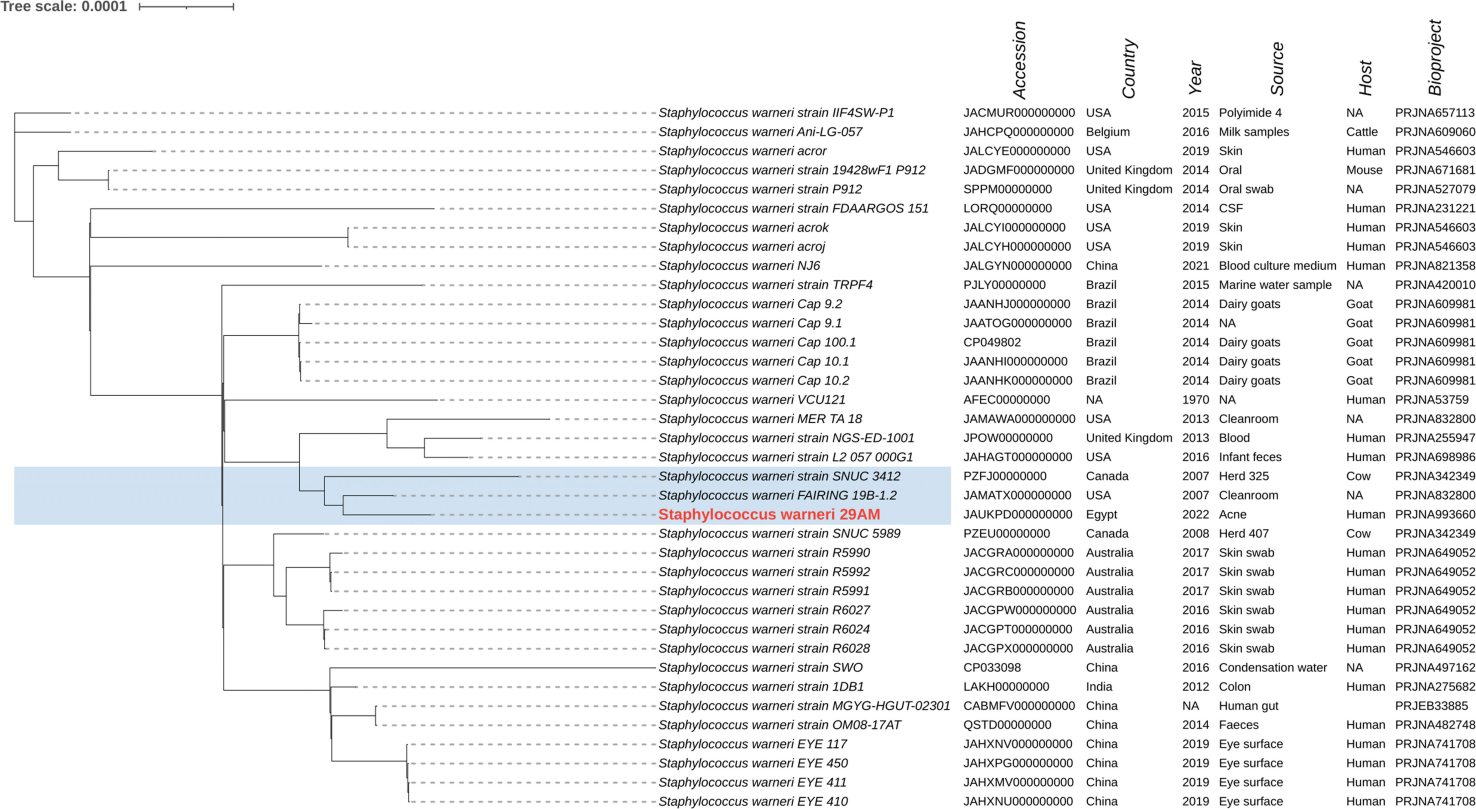
Whole genome-based phylogenetic tree of the *S. warneri* isolate 29AM. The *S. warneri* 29AM genome sequenced in the current study was compared to the closely related genomes, highlighted in blue. The label of the *S. warneri* strain sequenced in this study is shown in red. The figure was created using the iTOL online tool v6.7 (https://itol.embl.de/).

**Figure 7:**
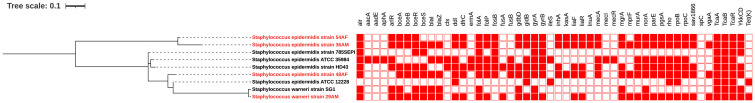
SNP-based phylogenetic tree of *Staphylococcus* isolates. SNP base phylogeny and heat map displaying the antimicrobial resistance genetic determinants among the sequenced isolates generated and selected genomes: *S. epidermidis* ATCC^®^12228, *S. epidermidis* ATCC^®^35984, *S. epidermidis* strain 785SEPI, and *S. warneri* strain SG1. Red and white indicate the presence and absence of ARGs, respectively. The labels of the strains sequenced in this study is shown in red. The figure was created using the iTOL online tool v6.7 (https://itol.embl.de/).

The phylogenetic relationships between the study isolates and their closely related genomes were determined for *S. epidermidis* isolates 36AM, 48AF, and 54AF core genomes and for *S. warneri* 29AM and were compared to the similar genomes detected by the BV-BRC database (**Supplementary Tables 5, 6**). A phylogenetic tree illustrating their genomic relatedness is shown in (**Figures 5**, **6**). An SNP-based phylogenetic tree was generated using the tested genomes and *S. epidermidis* ATCC^®^12228, *S. epidermidis* ATCC^®^35984, *S. epidermidis* strain 785SEPI, and *S. warneri* strain SG1 (**Figure 7**).

## 4 Discussion

Acne is a common inflammatory skin disorder that affects the sebaceous glands. Although acne develops through the interaction of several factors, the exact cause of acne remains unknown. The interaction between skin bacteria and host immunity is increasingly thought to play an essential role in this condition, with variable microbial composition and activity detected in patients with acne (Lee et al., 2018).

Our research explored the microbial dynamics within acne, studied the CoNS recovered from acne, and compared them to healthy skins. While the recovery rates of CoNS isolates from acne-diseased individuals closely paralleled those from their healthy skin counterparts, a significant contrast emerged in the phenotypic characterization of antibiotic resistance and biofilm production. Antibiotic susceptibility testing revealed a landscape of antibiotic resistance patterns. Although the antibiotic resistance profiles showed no substantial differences between the two groups, except for penicillin and cefoxitin, it is noteworthy that isolates from individuals with acne displayed significantly increased biofilm production compared to those from individuals with healthy skin. In the acne group, isolates exhibited the ability to form moderate (28.3%) and strong biofilms (38.3%), in marked contrast to the healthy isolates, where only 8% demonstrated moderate biofilm formation, and none exhibited strong biofilm production. This highlights the significance of going beyond the bacterial existence to comprehend the functional distinctions that contribute to the development and pathogenesis of acne. The present study explored the biofilm-associated genes (*icaA*, *icaD*, and *mecA*), which were particularly prevalent in clinical isolates from participants with moderate to severe acne. Co-expression of *icaA* and *icaD* significantly augmented biofilm formation, with *icaD* exhibiting a higher positive detection rate. The correlation between drug resistance and biofilm formation was significant. Bacteria in biofilms exhibit increased resistance to antibiotics because of the surrounding protective matrix. This enhanced resistance may contribute to the development of MDR and XDR strains. In our study, all the strong biofilm-forming CoNS were MDR. Strong biofilm formation may serve as a mechanism by which bacteria evade the effects of antimicrobial agents, allowing them to persist and proliferate. A protective biofilm matrix can shield bacteria from the action of antibiotics, making it more difficult to eradicate the infection. Additionally, the coexistence of drug resistance and strong biofilm formation may contribute to the persistence of bacterial infections leading to chronic and recurrent conditions (Coenye et al., 2022).

Four XDR and strong biofilm-forming CoNS isolates, recovered from severe acne infections, were selected for genomic analysis. We investigated the genomic features using WGS, including MGEs, genetic contexts of the identified resistance genes, and virulence factors. Upon analyzing the genomes of the four strains, three isolates (36AM, 48AF, and 54AF) were confirmed to be *S. epidermidis*, whereas isolate 29AM was identified as *S. warneri*, a common CoNS that is regularly found in human mucous membranes and various sites, including the eye, peritoneum, wounds, and urethra. While constituting approximately 1% of skin staphylococci and prevalent in 50% of healthy adults, *S. warneri* is typically non-pathogenic. However, recent studies have highlighted its emergence as a potential pathogen, particularly associated with implanted materials, even in the absence of foreign bodies, and in immunocompromised individuals. This shift challenged previous perceptions of its limited pathogenicity (Otto, 2010; França, 2023). The pathophysiology and epidemiology of this species are not well understood. Furthermore, methods used to distinguish *S. warneri* from *S. epidermidis*, such as conventional culturing techniques and MALDI-TOF MS, are not sufficiently sensitive (Dubois et al., 2010; Rosa et al., 2022). *S. epidermidis*, a common commensal on human epithelia, displays significant genetic diversity with over 400 recognized sequence types (STs), primarily clustering in CC2. The prevalent ST2, associated with invasiveness due to IS256 insertion sequences and *ica* genes, dominates in clinical isolates (Méric et al., 2015). The tested genomes were investigated for their ST types, and isolates 36AM, 48AF, and 54AF were identified as ST39, ST87, and ST719, respectively. Understanding the distribution of these STs in acne isolates could be relevant for investigating the genetic diversity and potential virulence factors associated with *S. epidermidis* in individuals with acne. Further analysis and comparison with STs from other contexts may provide insights into the role of specific genetic variants in the pathogenesis of acne or in the skin microbiome of patients with acne.

Genomic analysis of the isolates revealed a diverse and extensive drug resistance pattern involving various mechanisms, such as enzyme inactivation, target modification, and efflux pumps. Specific resistance genes (*dfrC*, *vanT*, *erm^©^*, *FOSBx1*, *blaZ*, and *norA*) and mutations in key genes (*gyrA*, *gyrB*, *parC*, *parE*, and *rpoB*) were identified. These findings align with those of previous studies and emphasize the adaptability of CoNS in acquiring resistance. The observed genetic diversity underscores the complexity of antibiotic resistance profiles (Xu et al., 2018; Cabrera-Contreras et al., 2019; Raue et al., 2020). The detection of *blaZ* in the isolates, along with their regulatory genes *blaR* and *blaI* in addition to the presence of the *mecA* gene, may explain the resistance to penicillin and cephalosporins observed in phenotypic antibiotic susceptibility testing, as reported by a previous study by Cabrera-Contreras et al. (2019). Despite the correlation between genotypic and phenotypic resistance to penicillin and cephalosporins, potential inconsistencies highlight the importance of considering factors such as sequencing quality.

Both pathogenic and commensal *S. epidermidis* strains share many virulence genes (Otto, 2009). Adherence/biofilm-forming genes and multidrug resistance were found in the sequenced isolates and confirmed by the identification of adherence-related genes. The *ica* operon and IS256, both of which have been linked to the pathogenicity of *S. epidermidis*, were not detected in any of the isolates (Murugesan et al., 2018). This is comparable to the results of a study in Mexico, where 50% of *S. epidermidis* isolates were negative for the *ica* operon (Cabrera-Contreras et al., 2019).

All sequenced genomes contained several virulence genes encoding proteins associated with the pathogenesis of acne. Several genes such as *atl*, *ebh*, *epb*, *sdrG*, and *sdrH* are involved in adhesion and biofilm formation. Enzymes implicated in acne production, including proteases (*sspA*, *sspB*) and lipases (*geh*, *lip*, *nuc*), were produced by all isolates. The presence of the *hlb* gene encoding β-hemolysin toxin was supported by Kumar et al. (2016), suggesting a role in tissue damage and pathogen spread into deeper tissues (O’Neill et al., 2020). Similar virulence determinants were detected in many studies on *S. epidermidis*, including the elastin binding protein gene *ebp*, the serine protease gene *sspA*, the autolysin gene *atlE*, the lipase gene *geh*, the cell wall-associated fibronectin-binding protein gene *ebh*, the nuclease gene *nuc*, and the *ica* genes (Salgueiro et al., 2017; Xu et al., 2018; Raue et al., 2020).

Compared to *S. epidermidis*, *S. warneri* strain 29AM exhibited additional virulence factors related to the production of toxins, resistance to acidic conditions, evasion of the immune system, intracellular survival capabilities, invasion mechanisms, iron uptake processes, lipid metabolism modulation, phagosome arresting ability, stress adaptation strategies, and surface protein anchoring. All of these virulence factors play integral roles in shaping the course of acne development. These findings provide insights into the diverse virulence profiles contributing to acne pathophysiology, with *S. warneri* strain 29AM displaying enhanced virulence factors that are not commonly associated with acne pathogens.

These antibiotic resistance and virulence genes are part of the accessory genome, which is organized both within and between species. The prediction of pathogenicity of isolates towards human hosts produced a high average likelihood score (*P*_score_ 0.937), close to 1.00. This pathogenicity score is attributed to the presence of numerous virulence genes in the isolates, which supports their pathogenic potential in humans.

MGEs play a significant role in acne pathophysiology, contributing to the genetic diversity and adaptability of bacteria involved in acne, such as *S. epidermidis* and *S. warneri*. MGEs, including SCC*mec* and ACME, serve as repositories for both resistance and virulence genes. Their mobility allows the transfer of genetic material within and across bacterial species through horizontal gene transfer, contributing to the exchange of traits within the bacterial population (Sheppard et al., 2016; Foster, 2017).

In the context of acne, the presence of MGEs is associated with the acquisition of virulence and resistance genes. For instance, the study identified IS256, an insertion sequence linked to virulence and biofilm development, in a strong biofilm-forming *S. warneri* 29AM isolate. This suggests that MGEs may enhance the genomic organization of bacteria involved in acne, potentially influencing their pathogenicity and ability to form biofilms, which is a key factor in acne development (Murugesan et al., 2018). IS256 has been shown in previous investigations to enhance the genomic organization of pathogenic *S. epidermidis* isolates (Cabrera-Contreras et al., 2019). The discovery of the IS families IS110 and IS200/IS605 coincides with the findings of Raue et al. (2020). Moreover, this study identified resistance genes frequently carried on plasmids, demonstrating how MGEs can facilitate the spread of antibiotic resistance. Plasmid-borne genes, such as *erm^©^*, *lnu(A)*, *tet(K)*, *qacC*, and *blaZ*, can be easily transferred between bacterial cells through conjugation, contributing to the dissemination of resistance traits within the bacterial population associated with acne (Cabrera-Contreras et al., 2019).

In this study, the prophages were found to be incapable of carrying resistance genes through transduction. However, transposons identified in two isolates, 36AM and 48AF, including Tn554 flanking *erm (A)*, may facilitate the movement of beneficial genes, particularly those linked to antibiotic resistance, in the context of acne-associated bacteria (Mbelle et al., 2019).

The CRISPR-Cas system, a bacterial defense mechanism against phage infection, was identified in all isolates, providing a memory of the viral genetic code to prevent future infections. Each isolate also contained at least one intact prophage (Makarova et al., 2020). In a recent study by Raue et al. (2020), four CRISPR elements were discovered in the genome of biofilm-positive and methicillin-susceptible *S. epidermidis* O47 strain. The restriction–modification (R–M) system was not detected in any of the isolates in this study. The R–M system, similar to the CRISPR-Cas system, is a bacterial defense system against bacteriophage invasion (Vasu and Nagaraja, 2013). These defense systems are crucial for protecting bacteria from viral infections, contributing to our understanding of their role in the complex pathogenesis of acne.

The ACME system, recognized as a pathogenicity island, was found in two isolates (48AF and 54AF), raising interest because of its potential role in host colonization, immune evasion, and the transfer of virulence or survival genes (O’Connor et al., 2018). Studies have evaluated the resistance and virulence profiles of *S. epidermidis* isolates from bloodstream infections and neonatal nares, and an ACME carriage rate of 16% was found (Salgueiro et al., 2017). ACME have a greater prevalence and variety in *S. epidermidis* than in *S. aureus*. ACME is present in bacterial chromosomes adjacent to the *SCCmec* type IV element in *S. aureus* (Diep et al., 2006; Ellington et al., 2008). The present study demonstrated an association between ACME and SCC*mec* type IV, which is consistent with the findings of Du et al. (2013). Most resistance genes are flanked by transposases, ISs, or both, and these can transmit resistance genes within and between plasmids and chromosomes, potentially within and between bacterial strains (Mbelle et al., 2019).

These findings provide insights into the genetic factors influencing acne pathogenesis, particularly in the context of bacterial defense mechanisms and the interplay of virulence and resistance genes. The diversity of MGEs observed in the *Staphylococcus* genomes indicates an active process of gene exchange, suggesting that MGEs contribute to the evolution and adaptation of bacteria involved in acne pathophysiology.

This study diverges from the previous focus on pathogenic *S. aureus* by emphasizing the potential pathogenicity of CoNS, which are typically considered commensals. To the best of our knowledge, this is the first report in Egypt that offers insights into the diversity, distribution, virulence, and resistance profiles of *S. epidermidis* and *S. warneri* isolates recovered from acne, establishing their relatedness to global strains through WGS. Despite the limited number of isolates, our research establishes a foundational understanding for future investigations, contributing to the comprehension of the potential role of CoNS in acne vulgaris. In conclusion, this study highlights the complex nature of acne, which explains its microbial interactions, biofilm formation, and virulence factors. This comprehensive insight is vital for developing targeted and personalized therapeutic strategies and advancing the understanding of acne pathophysiology for more effective treatment approaches.

## 5 Conclusion

CoNS is as a significant contributor to acne development and pathogenesis by releasing virulence factors, which are more abundant in acne patients than in normal skin. The diverse combinations of ARGs, virulence genes, and MGEs found in *S. epidermidis* and *S. warneri* isolates recovered from acne highlight the enriched content of mobilized antibiotic resistance and pathogenic features. This study emphasizes the importance of tracking CoNS genomes as emerging infectious agents and shedding light on their pathogenicity. Notably, our findings suggest that *S. warneri* is an emerging pathogen implicated in acne pathogenesis, urging reconsideration of its role in infectious processes. This study sheds light on the complex interplay between microbial elements and acne and offers valuable insights for future research and targeted interventions in acne management.

## Data availability statement

The datasets presented in this study can be found in online repositories. The names of the repository/repositories and accession number(s) can be found in the article/**Supplementary Material**.

## Ethics statement

The studies involving humans were approved by MSA University-Ethics Committee. The studies were conducted in accordance with the local legislation and institutional requirements. The participants provided their written informed consent to participate in this study.

## Author contributions

MA: Data curation, Formal analysis, Methodology, Resources, Software, Validation, Visualization, Writing – original draft, Writing – review & editing. MD: Data curation, Formal analysis, Methodology, Writing – original draft. NS: Investigation, Methodology, Project administration, Resources, Writing – original draft. HA: Conceptualization, Formal analysis, Investigation, Methodology, Project administration, Supervision, Validation, Writing – original draft, Writing – review & editing.
