# Supplementary material for: Resistome, mobilome, and virulome explored in clinical isolates derived from acne patients in Egypt: unveiling unique traits of an emerging coagulase-negative Staphylococcus pathogen
Source: Front Cell Infect Microbiol. 2024 Feb 2;14:1328390. doi: 10.3389/fcimb.2024.1328390 (PMC10869526; doi:10.3389/fcimb.2024.1328390)
Supplement: Supplementary file 1 [file DataSheet_1.docx]

Supplementary material

**Supplementary Table 1. Antimicrobial resistance prevalence of CoNS isolates from patients with acne vulgaris and healthy skin by disc diffusion method**

| Antibiotics | CoNS isolates from acne vulgaris (n=60) | | CoNS isolates from healthy skin (n=25) | | *P*-value |
| --- | --- | --- | --- | --- | --- |
|  | **Resistant**  **N (%)** | **Susceptible**  **N (%)** | **Resistant**  **N (%)** | **Susceptible**  **N (%)** |
| Penicillin  (10 units) | 44 (73%) | 16 (26.6%) | 10 (40%) | 15 (60%) | <0.05* |
| Cefoxitin (30 μg) | 38 (63%) | 22 (36.6%) | 10 (40%) | 15 (60%) | <0.05* |
| Levofloxacin  (5 μg) | 22 (36.7%) | 38 (63.3%) | 8(32%) | 17 (68%) | >0.05 |
| Erythromycin  (15 μg) | 29 (48%) | 31 (51.6%) | 11(44%) | 14 (56%) | >0.05 |
| Gentamicin  (10 μg) | 19 (31.7%) | 41 (68.3%) | 5 (20%) | 20 (80%) | >0.05 |
| Clindamycin  (2 μg) | 32 (53.3%) | 28 (46.6%) | 14 (56%) | 11 (44%) | >0.05 |
| Tetracycline  (30 μg) | 17 (28.3%) | 43 (71.6%) | 8 (32%) | 17 (68%) | >0.05 |
| Doxycycline  (30 μg) | 7 (11.7%) | 53 (88.3%) | 2(8%) | 23 (92%) | >0.05 |
| Minocycline  (30 μg) | 5 (8.3%) | 55 (91.6%) | 0 (0%) | 25 (100%) | >0.05 |
| Vancomycin  (30 μg) | 0 (0%) | 60 (100%) | 0 (0%) | 60 (100%) | >0.05 |
| Rifampin  (5 μg) | 0 (0%) | 60 (100%) | 0 (0%) | 60 (100%) | >0.05 |

(*): *P-*value < 0.05 is considered statistically significant.

**Supplementary Table 2*.* CoNS biofilm formation among different groups**

| Biofilm formation | Acne skin participants  N (%) | Healthy skin Participants  N (%) | *P*-value |
| --- | --- | --- | --- |
| Negative biofilm | 16 (26.6 %) | 14 (56%) | <0.05* |
| Weak biofilm producer | 4 (6.6 %) | 9 (36%) | <0.05* |
| Moderate biofilm producer | 17 (28.3 %) | 2 (8%) | <0.05* |
| Strong biofilm producer | 23 (38.3 %) | 0 (0%) | <0.05* |
| Total | 60 (100%) | 25 (100%) | <0.05* |

(*): *P-*value < 0.05 is considered statistically significant.

**Supplementary Table 3. Post-assembly metrics of the CoNS draft genomes generated in the current study**

| **ID** | **# Contigs** | **GC (%)** | **Genome fraction (%)** | **L50 N50 Total Length**  **(>= 0 bp)** | | | **Coding sequences (CDS)** | **tRNA** | **rRNA** |
| --- | --- | --- | --- | --- | --- | --- | --- | --- | --- |
| **29AM** | 114 | 32.44 | 82.464 | 5 | 156,505 | 2,68,121 | 2623 | 60 | 4 |
| **36AM** | 294 | 32.24 | 84.246 | 50 | 14,900 | 2,360,153 | 2317 | 52 | 2 |
| **46AF** | 90 | 31.88 | 84.320 | 8 | 110,039 | 2,525,218 | 2451 | 49 | 4 |
| **54AF** | 65 | 31.96 | 82.365 | 4 | 327,807 | 2,594,339 | 2538 | 55 | 2 |

N50, smallest contig of the size-sorted contigs that make up at least 50% of the respective assembly. L50, number of contigs that make up at least 50% of the respective total assembly length. GC, guanine–cytosine.

**Supplementary Table 4. Distribution of intact prophage regions among the *Staphylococcus* strains**

| **ID** | **Intact prophages** | **Region** | **Length (kb)** | | **No. CDS** | **GC%** | **Phage** | **Resistance genes** | |
| --- | --- | --- | --- | --- | --- | --- | --- | --- | --- |
| 29AM | 2 | 5 | 14.2 | 14416 | | 35.18 | PHAGE_Staphy_StB12_NC_020490 | - |  |
|  |  | 6 | 14.1 | | 14367 | 35.91 | PHAGE_Staphy_StB12_NC_020490 | - |  |
| 36AM | - | - | - | | - | - | - | - |  |
| 48AF | - | - | - | | - | - | - | - |  |
| 54AF | 2 | 1 | 14.2 | | 14416 | 35.18 | PHAGE_Staphy_StB12_NC_020490 | - |  |
|  |  | 2 | 14.1 | | 14367 | 35.91 | PHAGE_Staphy_StB12_NC_020490 | - |  |

# **Supplementary Table** **5. Genome accessions and metadata of the genomes retrieved from BV-BRC database included in the *S. epidermidis* genomic phylogeny analysis and mutation analysis.**

| **BV-BRC**  **ID** | **Strain** | **Accession** | **Bioproject** | **Country** | **Year** | **Specimen** | **ST** | **Host** |
| --- | --- | --- | --- | --- | --- | --- | --- | --- |
|  |  | **number** |  |  |  |  |  |  |
| 1282.1313 | 785_SEPI | JUTA00000000 | PRJNA267549 | USA | NA | NA | 723 | Human |
| 1282.2907 | VSE49 | MLXH00000000 | PRJNA348436 | India | 2014 | Skin | 691 | Human |
| 1282.291 | VSE57 | MLXL00000000 | PRJNA348444 | India | 2014 | Skin | 691 | Human |
| 1282.2929 | SE45 | LUBH00000000 | PRJNA314011 | India | 2015 | Male Behind the ears | 691 | Human |
| 1282.2938 | SE40 | LUBM00000000 | PRJNA314011 | India | 2015 | Female Nares | 562 | Human |
| 1282.326 | 32A | JXXM00000000 | PRJNA274319 | China | 2012 | Bovine milk | NA | NA |
| 1282.3356 | HD66 | CP040868 | PRJNA546129 | Germany | 2018 | Skin; prosthetic joint infection | 87 | Human |
| 1282.3357 | HD43 | CP040867 | PRJNA546129 | Germany | 2018 | Skin; prosthetic joint infection | 23 | Human |
| 1282.3358 | HD33 | CP040864 | PRJNA546129 | Germany | 2018 | Skin; prosthetic joint infection | 87 | Human |
| 1282.337 | DE0525 | VDPM00000000 | PRJNA543692 | USA | 2018 | Environmental | NA | NA |
| 1282.3432 | 4928STDY7071543 | CABGKA000000000 | PRJEB22252 | United Kingdom | 2018 | Faecal | 439 | Human |
| 1282.3647 | BAV 2502 | WINZ00000000 | PRJNA576872 | USA | 2013 | Precipitation | NA | NA |
| 1282.3652 | APC 3784 | SHEI00000000 | PRJNA521309 | Ireland | NA | Milk | NA | Human |
| 1282.378 | SESURV_p1_0557 | CP043777 | PRJNA559376 | USA | 2018 | Right nares | 487 | Human |
| 1282.3782 | SESURV_p1_0612 | CP043784 | PRJNA559376 | USA | 2018 | Toeweb | 640 | Human |
| 1282.3783 | SESURV_p4_1553 | CP043804 | PRJNA559376 | USA | 2018 | Umbilicus | 218 | Human |
| 1282.3784 | SESURV_p1_1200 | CP043796 | PRJNA559376 | USA | 2018 | Cubital_fossa | 487 | Human |
| 1282.3787 | SESURV_p3_0825 | CP043792 | PRJNA559376 | USA | 2018 | Left thumb | 73 | Human |
| 1282.3805 | JH | JAAUOD000000000 | PRJNA615880 | Netherlands | 2018 | Healthy skin | NA | Human |
| 1282.4083 | Z0118SE0132 | CP061029 | PRJNA660487 | NA | 2018 | NA | 924 | Human |
| 1282.4117 | R5981 | JACGRH000000000 | PRJNA649052 | Australia | 2017 | Skin swab1599 from squamous cell carcinomas patient MST023 | NA | Human |
| 1282.4164 | D43t1_170807_H6 | JADNCQ000000000 | PRJNA637878 | USA | 2017 | Stool | NA | Human |
| 1282.4262 | JH-S-3 | JABTXF000000000 | PRJNA636798 | Netherlands | 2020 | Skin | NA | Human |
| 1282.4263 | JH-S-1 | JABTXG000000000 | PRJNA636798 | Netherlands | 2020 | Skin | 329 | Human |
| 1282.4317 | s2 | QVTI00000000 | PRJNA486830 | USA | 2006 | Skin | NA | NA |
| 1282.4646 | S43 | JAIEXP000000000 | PRJNA636233 | India | 2019 | Rectal swab | NA | Cow |
| 1282.4647 | S17W | JAIEXR000000000 | PRJNA636233 | India | 2019 | Nasal swab | 329 | Cow |
| 1282.4742 | 48 | CP090924 | PRJNA721840 | France | 2012 | NA | NA | Human |
| 1282.4807 | ABKVF | JAKRZY000000000 | PRJNA694925 | USA | 2011 | NA | NA | Human |
| 1282.4853 | CICARIA | CP093222, | PRJNA813367 | South Korea | 2018 | Skin | 8 | Human |
| 1282.4875 | SKN25lux | CP095090 | PRJNA823545 | Switzerland | 2018 | Bloodstream | 5 | Human |
| 1715005.3 | HMSC074F11 | LTOJ00000000 | PRJNA296520 | NA | NA | Bone | NA | Human |
| 1739550.3 | HMSC068G11 | LTSF00000000 | PRJNA300201 | NA | NA | Cervical | 329 | Human |
| 176279.9 | RP62A | CP000029 | PRJNA64 | NA | NA | Patient with intravascular catheter-associated sepsis | 10 | Human |
| 176280.1 | ATCC 12228 | AE015929 | PRJNA279 | NA | NA | NA | 8 | NA |
| 979210.3 | NIHLM037 | AKGT00000000 | PRJNA62369 | USA | 2008 | Glabella | 329 | Human |
| 979216.3 | NIHLM015 | AKGZ00000000 | PRJNA62381 | USA | 2008 | Manubrium | NA | Human |

# **Supplementary Table** **6. Genome accessions and metadata of the genomes retrieved from BV-BRC database included in the *S. warneri* genomic phylogeny analysis and mutation analysis.**

| **BV-BRC ID** | **Strain** | **Accession** | **Bioproject** | **Country** | **Year** | **Specimen** | **Host** |
| --- | --- | --- | --- | --- | --- | --- | --- |
|  |  | **number** |  |  |  |  |  |
| 1292.116 | TRPF4 | PJLY00000000 | PRJNA420010 | Brazil | 2015 | Marine water sample collected about 100 m from Turtle Beach | NA |
| 1292.118 | P912 | SPPM00000000 | PRJNA527079 | United Kingdom | 2014 | Oral swab | NA |
| 1292.12 | NGS-ED-1001 | JPOW00000000 | PRJNA255947 | United Kingdom | 2013 | Blood | Human |
| 1292.13 | 1DB1 | LAKH00000000 | PRJNA275682 | India | 2012 | Colon | Human |
| 1292.13 | MGYG-HGUT-02301 | CABMFV000000000 | PRJEB33885 | China | NA | Human gut |  |
| 1292.161 | IIF4SW-P1 | JACMUR000000000 | PRJNA657113 | USA | 2015 | Polyimide 4 | NA |
| 1292.168 | 19428wF1_P912 | JADGMF000000000 | PRJNA671681 | United Kingdom | 2014 | Oral | Mouse |
| 1292.169 | R5992 | JACGRC000000000 | PRJNA649052 | Australia | 2017 | Skin swab1574 from squamous cell carcinomas patient MST020 | Human |
| 1292.17 | R5990 | JACGRA000000000 | PRJNA649052 | Australia | 2017 | Skin swab1574 from squamous cell carcinomas patient MST020 | Human |
| 1292.171 | R5991 | JACGRB000000000 | PRJNA649052 | Australia | 2017 | Skin swab1574 from squamous cell carcinomas patient MST020 | Human |
| 1292.172 | R6028 | JACGPX000000000 | PRJNA649052 | Australia | 2016 | Skin swab1470 from squamous cell carcinomas patient MST012 | Human |
| 1292.173 | R6027 | JACGPW000000000 | PRJNA649052 | Australia | 2016 | Skin swab1470 from squamous cell carcinomas patient MST012 | Human |
| 1292.174 | R6024 | JACGPT000000000 | PRJNA649052 | Australia | 2016 | Skin swab1470 from squamous cell carcinomas patient MST012 | Human |
| 1292.175 | J1101437_171009_F1 | JADMXG000000000 | PRJNA637878 | USA | 2017 | Stool | Human |
| 1292.189 | L2_057_000G1_dasL2_057_000G1_concoct_26_sub | JAHAGT000000000 | PRJNA698986 | USA | 2016 | Infant feces | Human |
| 1292.209 | Cap 100.1 | CP049802 | PRJNA609981 | Brazil | 2014 | Dairy goats with persistent mastitis before therapy with enrofloxacin | Goat |
| 1292.212 | Ani-LG-057 | JAHCPQ000000000 | PRJNA609060 | Belgium | 2016 | Milk samples | Cattle |
| 1292.215 | Cap 9.2 | JAANHJ000000000 | PRJNA609981 | Brazil | 2014 | Dairy goats with persistent mastitis after therapy with enrofloxacin | Goat |
| 1292.216 | Cap 10.1 | JAANHI000000000 | PRJNA609981 | Brazil | 2014 | Dairy goats with persistent mastitis before therapy with enrofloxacin | Goat |
| 1292.217 | Cap 9.1 | JAATOG000000000 | PRJNA609981 | Brazil | 2014 | NA | Goat |
| 1292.218 | Cap 10.2 | JAANHK000000000 | PRJNA609981 | Brazil | 2014 | Dairy goats with persistent mastitis after therapy with enrofloxacin | Goat |
| 1292.225 | acrok | JALCYI000000000 | PRJNA546603 | USA | 2019 | Skin | Human |
| 1292.228 | acroj | JALCYH000000000 | PRJNA546603 | USA | 2019 | Skin | Human |
| 1292.229 | acror | JALCYE000000000 | PRJNA546603 | USA | 2019 | Skin | Human |
| 1292.236 | NJ6 | JALGYN000000000 | PRJNA821358 | China | 2021 | Blood culture medium | Human |
| 1292.253 | EYE_450 | JAHXPG000000000 | PRJNA741708 | China | 2019 | Ocular surface of healthy person | Human |
| 1292.254 | EYE_117 | JAHXNV000000000 | PRJNA741708 | China | 2019 | Ocular surface of healthy person | Human |
| 1292.255 | EYE_410 | JAHXNU000000000 | PRJNA741708 | China | 2019 | Ocular surface of healthy person | Human |
| 1292.256 | EYE_411 | JAHXMV000000000 | PRJNA741708 | China | 2019 | Ocular surface of healthy person | Human |
| 1292.259 | MER TA 18 | JAMAWA000000000 | PRJNA832800 | USA | 2013 | Cleanroom | NA |
| 1292.26 | FAIRING 19B-1.2 | JAMATX000000000 | PRJNA832800 | USA | 2007 | Cleanroom | NA |
| 1292.3 | FDAARGOS_151 | LORQ00000000 | PRJNA231221 | USA | 2014 | CSF | Human |
| 1292.63 | SNUC 5989 | PZEU00000000 | PRJNA342349 | Canada | 2008 | Herd 407 | Cow |
| 1292.65 | SNUC 3412 | PZFJ00000000 | PRJNA342349 | Canada | 2007 | Herd 325 | Cow |
| 1292.77 | OM08-17AT | QSTD00000000 | PRJNA482748 | China | 2014 | Faeces | Human |
| 1292.91 | SWO | CP033098 | PRJNA497162 | China | 2016 | Condensation water of the Shenzhou-10 spacecraft | NA |
| 904338.3 | VCU121 | AFEC00000000 | PRJNA53759 | NA | 1970 | NA | Human |

cov pid  **1** **[ . . . . : . . .** **80**

1 ATCC35984 100.0% 100.0%  **MAELPQSRINERNITSEMRESFLDYAMSVIVSRALPDVRDGLKPVHRRILYGLNEQGMTPDKPYKKSARIVGDVMGKYHP**

2 ATCC12228 93.3% 99.9%  **------------------------------------------------------------DKPYKKSARIVGDVMGKYHP**

3 36AM 100.0% 99.0%  **MAELPQSRINERNITSEMRESFLDYAMSVIVSRALPDVRDGLKPVHRRILYGLNEQGMTPDKPYKKSARIVGDVMGKYHP**

4 48AF 100.0% 99.9%  **MAELPQSRINERNITSEMRESFLDYAMSVIVSRALPDVRDGLKPVHRRILYGLNEQGMTPDKPYKKSARIVGDVMGKYHP**

5 54AF 100.0% 99.0%  **MAELPQSRINERNITSEMRESFLDYAMSVIVSRALPDVRDGLKPVHRRILYGLNEQGMTPDKPYKKSARIVGDVMGKYHP**

6 NIHLM037 100.0% 99.1%  **MAELPQSRINERNITSEMRESFLDYAMSVIVSRALPDVRDGLKPVHRRILYGLNEQGMTPDKPYKKSARIVGDVMGKYHP**

7 785_SEPI 100.0% 99.0%  **MAELPQSRINERNITSEMRESFLDYAMSVIVSRALPDVRDGLKPVHRRILYGLNEQGMTPDKPYKKSARIVGDVMGKYHP**

8 VSE49 100.0% 99.1%  **MAELPQSRINERNITSEMRESFLDYAMSVIVSRALPDVRDGLKPVHRRILYGLNEQGMTPDKPYKKSARIVGDVMGKYHP**

9 VSE57 100.0% 99.1%  **MAELPQSRINERNITSEMRESFLDYAMSVIVSRALPDVRDGLKPVHRRILYGLNEQGMTPDKPYKKSARIVGDVMGKYHP**

10 SE45 100.0% 99.1%  **MAELPQSRINERNITSEMRESFLDYAMSVIVSRALPDVRDGLKPVHRRILYGLNEQGMTPDKPYKKSARIVGDVMGKYHP**

11 SE40 100.0% 99.1%  **MAELPQSRINERNITSEMRESFLDYAMSVIVSRALPDVRDGLKPVHRRILYGLNEQGMTPDKPYKKSARIVGDVMGKYHP**

12 32A 100.0% 99.1%  **MAELPQSRINERNITSEMRESFLDYAMSVIVSRALPDVRDGLKPVHRRILYGLNEQGMTPDKPYKKSARIVGDVMGKYHP**

13 HD66 100.0% 99.8%  **MAELPQSRINERNITSEMRESFLDYAMSVIVSRALPDVRDGLKPVHRRILYGLNEQGMTPDKPYKKSARIVGDVMGKYHP**

14 HD43 100.0% 99.9%  **MAELPQSRINERNITSEMRESFLDYAMSVIVSRALPDVRDGLKPVHRRILYGLNEQGMTPDKPYKKSARIVGDVMGKYHP**

15 HD33 100.0% 99.8%  **MAELPQSRINERNITSEMRESFLDYAMSVIVSRALPDVRDGLKPVHRRILYGLNEQGMTPDKPYKKSARIVGDVMGKYHP**

16 DE0525 100.0% 99.1%  **MAELPQSRINERNITSEMRESFLDYAMSVIVSRALPDVRDGLKPVHRRILYGLNEQGMTPDKPYKKSARIVGDVMGKYHP**

17 4928STDY7071543 100.0% 99.0%  **MAELPQSRINERNITSEMRESFLDYAMSVIVSRALPDVRDGLKPVHRRILYGLNEQGMTPDKPYKKSARIVGDVMGKYHP**

18 BAV2502 100.0% 99.0%  **MAELPQSRINERNITSEMRESFLDYAMSVIVSRALPDVRDGLKPVHRRILYGLNEQGMTPDKPYKKSARIVGDVMGKYHP**

19 APC3784 100.0% 99.0%  **MAELPQSRINERNITSEMRESFLDYAMSVIVSRALPDVRDGLKPVHRRILYGLNEQGMTPDKPYKKSARIVGDVMGKYHP**

20 SESURV_p1_0557 100.0% 99.9%  **MAELPQSRINERNITSEMRESFLDYAMSVIVSRALPDVRDGLKPVHRRILYGLNEQGMTPDKPYKKSARIVGDVMGKYHP**

21 SESURV_p1_0612 93.3% 99.0%  **------------------------------------------------------------DKPYKKSARIVGDVMGKYHP**

22 SESURV_p4_1553 100.0% 99.9%  **MAELPQSRINERNITSEMRESFLDYAMSVIVSRALPDVRDGLKPVHRRILYGLNEQGMTPDKPYKKSARIVGDVMGKYHP**

23 SESURV_p1_1200 100.0% 99.9%  **MAELPQSRINERNITSEMRESFLDYAMSVIVSRALPDVRDGLKPVHRRILYGLNEQGMTPDKPYKKSARIVGDVMGKYHP**

24 SESURV_p3_0825 93.3% 99.8%  **------------------------------------------------------------DKPYKKSARIVGDVMGKYHP**

25 JH 100.0% 98.9%  **MAELPQSRINERNITSEMRESFLDYAMSVIVSRALPDVRDGLKPVHRRILYGLNEQGMTPDKPYKKSARIVGDVMGKYHP**

26 Z0118SE0132 93.3% 98.9%  **------------------------------------------------------------DKPYKKSARIVGDVMGKYHP**

27 R5981 93.3% 98.9%  **------------------------------------------------------------DKPYKKSARIVGDVMGKYHP**

28 JH-S-3 100.0% 98.9%  **MAELPQSRINERNITSEMRESFLDYAMSVIVSRALPDVRDGLKPVHRRILYGLNEQGMTPDKPYKKSARIVGDVMGKYHP**

29 JH-S-1 93.3% 98.8%  **------------------------------------------------------------DKPYKKSARIVGDVMGKYHP**

30 s2 100.0% 99.1%  **MAELPQSRINERNITSEMRESFLDYAMSVIVSRALPDVRDGLKPVHRRILYGLNEQGMTPDKPYKKSARIVGDVMGKYHP**

31 S43 100.0% 99.1%  **MAELPQSRINERNITSEMRESFLDYAMSVIVSRALPDVRDGLKPVHRRILYGLNEQGMTPDKPYKKSARIVGDVMGKYHP**

32 S17W 93.3% 99.0%  **------------------------------------------------------------DKPYKKSARIVGDVMGKYHP**

33 48 100.0% 99.0%  **MAELPQSRINERNITSEMRESFLDYAMSVIVSRALPDVRDGLKPVHRRILYGLNEQGMTPDKPYKKSARIVGDVMGKYHP**

34 ABKVF 100.0% 99.0%  **MAELPQSRINERNITSEMRESFLDYAMSVIVSRALPDVRDGLKPVHRRILYGLNEQGMTPDKPYKKSARIVGDVMGKYHP**

35 CICARIA 100.0% 99.9%  **MAELPQSRINERNITSEMRESFLDYAMSVIVSRALPDVRDGLKPVHRRILYGLNEQGMTPDKPYKKSARIVGDVMGKYHP**

36 SKN25lux 100.0% 99.8%  **MAELPQSRINERNITSEMRESFLDYAMSVIVSRALPDVRDGLKPVHRRILYGLNEQGMTPDKPYKKSARIVGDVMGKYHP**

37 HMSC074F11 100.0% 99.1%  **MAELPQSRINERNITSEMRESFLDYAMSVIVSRALPDVRDGLKPVHRRILYGLNEQGMTPDKPYKKSARIVGDVMGKYHP**

38 HMSC068G11 100.0% 99.1%  **MAELPQSRINERNITSEMRESFLDYAMSVIVSRALPDVRDGLKPVHRRILYGLNEQGMTPDKPYKKSARIVGDVMGKYHP**

39 NIHLM015 100.0% 99.1%  **MAELPQSRINERNITSEMRESFLDYAMSVIVSRALPDVRDGLKPVHRRILYGLNEQGMTPDKPYKKSARIVGDVMGKYHP**

consensus/100%  **............................................................DKPYKKSARIVGDVMGKYHP**

consensus/90%  **............................................................DKPYKKSARIVGDVMGKYHP**

consensus/80%  **MAELPQSRINERNITSEMRESFLDYAMSVIVSRALPDVRDGLKPVHRRILYGLNEQGMTPDKPYKKSARIVGDVMGKYHP**

consensus/70%  **MAELPQSRINERNITSEMRESFLDYAMSVIVSRALPDVRDGLKPVHRRILYGLNEQGMTPDKPYKKSARIVGDVMGKYHP**

cov pid  **81**  **. 1 . . . . : .** **160**

1 ATCC35984 100.0% 100.0%  **HGDSSIYEAMVRMAQDFSYRYPLVDGQGNFGSMDGDGAAAMRYTEARMTKITLELLRDINKDTIDFIDNYDGNEREPSVL**

2 ATCC12228 93.3% 99.9%  **HGDSSIYEAMVRMAQDFSYRYPLVDGQGNFGSMDGDGAAAMRYTEARMTKITLELLRDINKDTIDFIDNYDGNEREPSVL**

3 36AM 100.0% 99.0%  **HGDSSIYEAMVRMAQDFSYRYPLVDGQGNFGSMDGDGAAAMRYTEARMTKITLELLRDINKDTIDFIDNYDGNEREPSVL**

4 48AF 100.0% 99.9%  **HGDSSIYEAMVRMAQDFSYRYPLVDGQGNFGSMDGDGAAAMRYTEARMTKITLELLRDINKDTIDFIDNYDGNEREPSVL**

5 54AF 100.0% 99.0%  **HGDSSIYEAMVRMAQDFSYRYPLVDGQGNFGSMDGDGAAAMRYTEARMTKITLELLRDINKDTIDFIDNYDGNEREPSVL**

6 NIHLM037 100.0% 99.1%  **HGDSSIYEAMVRMAQDFSYRYPLVDGQGNFGSMDGDGAAAMRYTEARMTKITLELLRDINKDTIDFIDNYDGNEREPSVL**

7 785_SEPI 100.0% 99.0%  **HGDSSIYEAMVRMAQDFSYRYPLVDGQGNFGSMDGDGAAAMRYTEARMTKITLELLRDINKDTIDFIDNYDGNEREPSVL**

8 VSE49 100.0% 99.1%  **HGDSSIYEAMVRMAQDFSYRYPLVDGQGNFGSMDGDGAAAMRYTEARMTKITLELLRDINKDTIDFIDNYDGNEREPSVL**

9 VSE57 100.0% 99.1%  **HGDSSIYEAMVRMAQDFSYRYPLVDGQGNFGSMDGDGAAAMRYTEARMTKITLELLRDINKDTIDFIDNYDGNEREPSVL**

10 SE45 100.0% 99.1%  **HGDSSIYEAMVRMAQDFSYRYPLVDGQGNFGSMDGDGAAAMRYTEARMTKITLELLRDINKDTIDFIDNYDGNEREPSVL**

11 SE40 100.0% 99.1%  **HGDSSIYEAMVRMAQDFSYRYPLVDGQGNFGSMDGDGAAAMRYTEARMTKITLELLRDINKDTIDFIDNYDGNEREPSVL**

12 32A 100.0% 99.1%  **HGDSSIYEAMVRMAQDFSYRYPLVDGQGNFGSMDGDGAAAMRYTEARMTKITLELLRDINKDTIDFIDNYDGNEREPSVL**

13 HD66 100.0% 99.8%  **HGDFSIYEAMVRMAQDFSYRYPLVDGQGNFGSMDGDGAAAMRYTEARMTKITLELLRDINKDTIDFIDNYDGNEREPSVL**

14 HD43 100.0% 99.9%  **HGDFSIYEAMVRMAQDFSYRYPLVDGQGNFGSMDGDGAAAMRYTEARMTKITLELLRDINKDTIDFIDNYDGNEREPSVL**

15 HD33 100.0% 99.8%  **HGDFSIYEAMVRMAQDFSYRYPLVDGQGNFGSMDGDGAAAMRYTEARMTKITLELLRDINKDTIDFIDNYDGNEREPSVL**

16 DE0525 100.0% 99.1%  **HGDSSIYEAMVRMAQDFSYRYPLVDGQGNFGSMDGDGAAAMRYTEARMTKITLELLRDINKDTIDFIDNYDGNEREPSVL**

17 4928STDY7071543 100.0% 99.0%  **HGDSSIYEAMVRMAQDFSYRYPLVDGQGNFGSMDGDGAAAMRYTEARMTKITLELLRDINKDTIDFIDNYDGNEREPSVL**

18 BAV2502 100.0% 99.0%  **HGDSSIYEAMVRMAQDFSYRYPLVDGQGNFGSMDGDGAAAMRYTEARMTKITLELLRDINKDTIDFIDNYDGNEREPSVL**

19 APC3784 100.0% 99.0%  **HGDSSIYEAMVRMAQDFSYRYPLVDGQGNFGSMDGDGAAAMRYTEARMTKITLELLRDINKDTIDFIDNYDGNEREPSVL**

20 SESURV_p1_0557 100.0% 99.9%  **HGDSSIYEAMVRMAQDFSYRYPLVDGQGNFGSMDGDGAAAMRYTEARMTKITLELLRDINKDTIDFIDNYDGNEREPSVL**

21 SESURV_p1_0612 93.3% 99.0%  **HGDSSIYEAMVRMAQDFSYRYPLVDGQGNFGSMDGDGAAAMRYTEARMTKITLELLRDINKDTIDFIDNYDGNEREPSVL**

22 SESURV_p4_1553 100.0% 99.9%  **HGDSSIYEAMVRMAQDFSYRYPLVDGQGNFGSMDGDGAAAMRYTEARMTKITLELLRDINKDTIDFIDNYDGNEREPSVL**

23 SESURV_p1_1200 100.0% 99.9%  **HGDSSIYEAMVRMAQDFSYRYPLVDGQGNFGSMDGDGAAAMRYTEARMTKITLELLRDINKDTIDFIDNYDGNEREPSVL**

24 SESURV_p3_0825 93.3% 99.8%  **HGDSSIYEAMVRMAQDFSYRYPLVDGQGNFGSMDGDGAAAMRYTEARMTKITLELLRDINKDTIDFIDNYDGNEREPSVL**

25 JH 100.0% 98.9%  **HGDSSIYEAMVRMAQDFSYRYPLVDGQGNFGSMDGDGAAAMRYTEARMTKITLELLRDINKDTIDFIDNYDGNEREPSVL**

26 Z0118SE0132 93.3% 98.9%  **HGDSSIYEAMVRMAQDFSYRYPLVDGQGNFGSMDGDGAAAMRYTEARMTKITLELLRDINKDTIDFIDNYDGNEREPSVL**

27 R5981 93.3% 98.9%  **HGDSSIYEAMVRMAQDFSYRYPLVDGQGNFGSMDGDGAAAMRYTEARMTKITLELLRDINKDTIDFIDNYDGNEREPSVL**

28 JH-S-3 100.0% 98.9%  **HGDSSIYEAMVRMAQDFSYRYPLVDGQGNFGSMDGDGAAAMRYTEARMTKITLELLRDINKDTIDFIDNYDGNEREPSVL**

29 JH-S-1 93.3% 98.8%  **HGDSSIYEAMVRMAQDFSYRYPLVDGQGNFGSMDGDGAAAMRYTEARMTKITLELLRDINKDTIDFIDNYDGNEREPSVL**

30 s2 100.0% 99.1%  **HGDSSIYEAMVRMAQDFSYRYPLVDGQGNFGSMDGDGAAAMRYTEARMTKITLELLRDINKDTIDFIDNYDGNEREPSVL**

31 S43 100.0% 99.1%  **HGDSSIYEAMVRMAQDFSYRYPLVDGQGNFGSMDGDGAAAMRYTEARMTKITLELLRDINKDTIDFIDNYDGNEREPSVL**

32 S17W 93.3% 99.0%  **HGDSSIYEAMVRMAQDFSYRYPLVDGQGNFGSMDGDGAAAMRYTEARMTKITLELLRDINKDTIDFIDNYDGNEREPSVL**

33 48 100.0% 99.0%  **HGDSSIYEAMVRMAQDFSYRYPLVDGQGNFGSMDGDGAAAMRYTEARMTKITLELLRDINKDTIDFIDNYDGNEREPSVL**

34 ABKVF 100.0% 99.0%  **HGDSSIYEAMVRMAQDFSYRYPLVDGQGNFGSMDGDGAAAMRYTEARMTKITLELLRDINKDTIDFIDNYDGNEREPSVL**

35 CICARIA 100.0% 99.9%  **HGDSSIYEAMVRMAQDFSYRYPLVDGQGNFGSMDGDGAAAMRYTEARMTKITLELLRDINKDTIDFIDNYDGNEREPSVL**

36 SKN25lux 100.0% 99.8%  **HGDFSIYEAMVRMAQDFSYRYPLVDGQGNFGSMDGDGAAAMRYTEARMTKITLELLRDINKDTIDFIDNYDGNEREPSVL**

37 HMSC074F11 100.0% 99.1%  **HGDSSIYEAMVRMAQDFSYRYPLVDGQGNFGSMDGDGAAAMRYTEARMTKITLELLRDINKDTIDFIDNYDGNEREPSVL**

38 HMSC068G11 100.0% 99.1%  **HGDSSIYEAMVRMAQDFSYRYPLVDGQGNFGSMDGDGAAAMRYTEARMTKITLELLRDINKDTIDFIDNYDGNEREPSVL**

39 NIHLM015 100.0% 99.1%  **HGDSSIYEAMVRMAQDFSYRYPLVDGQGNFGSMDGDGAAAMRYTEARMTKITLELLRDINKDTIDFIDNYDGNEREPSVL**

consensus/100%  **HGD.SIYEAMVRMAQDFSYRYPLVDGQGNFGSMDGDGAAAMRYTEARMTKITLELLRDINKDTIDFIDNYDGNEREPSVL**

consensus/90%  **HGD.SIYEAMVRMAQDFSYRYPLVDGQGNFGSMDGDGAAAMRYTEARMTKITLELLRDINKDTIDFIDNYDGNEREPSVL**

consensus/80%  **HGDSSIYEAMVRMAQDFSYRYPLVDGQGNFGSMDGDGAAAMRYTEARMTKITLELLRDINKDTIDFIDNYDGNEREPSVL**

consensus/70%  **HGDSSIYEAMVRMAQDFSYRYPLVDGQGNFGSMDGDGAAAMRYTEARMTKITLELLRDINKDTIDFIDNYDGNEREPSVL**

cov pid **161**  **. . . 2 . . . .** **240**

1 ATCC35984 100.0% 100.0%  **PARFPNLLVNGAAGIAVGMATNIPPHNLTEVIDGVLSLSKNPDITINELMEDIQGPDFPTAGLVLGKSGIRRAYETGRGS**

2 ATCC12228 93.3% 99.9%  **PARFPNLLVNGAAGIAVGMATNIPPHNLTEVIDGVLSLSKNPDITINELMEDIQGPDFPTAGLVLGKSGIRRAYETGRGS**

3 36AM 100.0% 99.0%  **PARFPNLLVNGAAGIAVGMATNIPPHNLTEVIDGVLSLSKNPDITINELMEDIQGPDFPTAGLVLGKSGIRRAYETGRGS**

4 48AF 100.0% 99.9%  **PARFPNLLVNGAAGIAVGMATNIPPHNLTEVIDGVLSLSKNPDITINELMEDIQGPDFPTAGLVLGKSGIRRAYETGRGS**

5 54AF 100.0% 99.0%  **PARFPNLLVNGAAGIAVGMATNIPPHNLTEVIDGVLSLSKNPDITINELMEDIQGPDFPTAGLVLGKSGIRRAYETGRGS**

6 NIHLM037 100.0% 99.1%  **PARFPNLLVNGAAGIAVGMATNIPPHNLTEVIDGVLSLSKNPDITINELMEDIQGPDFPTAGLVLGKSGIRRAYETGRGS**

7 785_SEPI 100.0% 99.0%  **PARFPNLLVNGAAGIAVGMATNIPPHNLTEVIDGVLSLSKNPDITINELMEDIQGPDFPTAGLVLGKSGIRRAYETGRGS**

8 VSE49 100.0% 99.1%  **PARFPNLLVNGAAGIAVGMATNIPPHNLTEVIDGVLSLSKNPDITINELMEDIQGPDFPTAGLVLGKSGIRRAYETGRGS**

9 VSE57 100.0% 99.1%  **PARFPNLLVNGAAGIAVGMATNIPPHNLTEVIDGVLSLSKNPDITINELMEDIQGPDFPTAGLVLGKSGIRRAYETGRGS**

10 SE45 100.0% 99.1%  **PARFPNLLVNGAAGIAVGMATNIPPHNLTEVIDGVLSLSKNPDITINELMEDIQGPDFPTAGLVLGKSGIRRAYETGRGS**

11 SE40 100.0% 99.1%  **PARFPNLLVNGAAGIAVGMATNIPPHNLTEVIDGVLSLSKNPDITINELMEDIQGPDFPTAGLVLGKSGIRRAYETGRGS**

12 32A 100.0% 99.1%  **PARFPNLLVNGAAGIAVGMATNIPPHNLTEVIDGVLSLSKNPDITINELMEDIQGPDFPTAGLVLGKSGIRRAYETGRGS**

13 HD66 100.0% 99.8%  **PARFPNLLVNGAAGIAVGMATNIPPHNLTEVIDGVLSLSKNPDITINELMEDIQGPDFPTAGLVLGKSGIRRAYETGRGS**

14 HD43 100.0% 99.9%  **PARFPNLLVNGAAGIAVGMATNIPPHNLTEVIDGVLSLSKNPDITINELMEDIQGPDFPTAGLVLGKSGIRRAYETGRGS**

15 HD33 100.0% 99.8%  **PARFPNLLVNGAAGIAVGMATNIPPHNLTEVIDGVLSLSKNPDITINELMEDIQGPDFPTAGLVLGKSGIRRAYETGRGS**

16 DE0525 100.0% 99.1%  **PARFPNLLVNGAAGIAVGMATNIPPHNLTEVIDGVLSLSKNPDITINELMEDIQGPDFPTAGLVLGKSGIRRAYETGRGS**

17 4928STDY7071543 100.0% 99.0%  **PARFPNLLVNGAAGIAVGMATNIPPHNLTEVIDGVLSLSKNPDITINELMEDVQGPDFPTAGLVLGKSGIRRAYETGRGS**

18 BAV2502 100.0% 99.0%  **PARFPNLLVNGAAGIAVGMATNIPPHNLTEVIDGVLSLSKNPDITINELMEDIQGPDFPTAGLVLGKSGIRRAYETGRGS**

19 APC3784 100.0% 99.0%  **PARFPNLLVNGAAGIAVGMATNIPPHNLTEVIDGVLSLSKNPDITINELMEDIQGPDFPTAGLVLGKSGIRRAYETGRGS**

20 SESURV_p1_0557 100.0% 99.9%  **PARFPNLLVNGAAGIAVGMATNIPPHNLTEVIDGVLSLSKNPDITINELMEDIQGPDFPTAGLVLGKSGIRRAYETGRGS**

21 SESURV_p1_0612 93.3% 99.0%  **PARFPNLLVNGAAGIAVGMATNIPPHNLTEVIDGVLSLSKNPDITINELMEDIQGPDFPTAGLVLGKSGIRRAYETGRGS**

22 SESURV_p4_1553 100.0% 99.9%  **PARFPNLLVNGAAGIAVGMATNIPPHNLTEVIDGVLSLSKNPDITINELMEDIQGPDFPTAGLVLGKSGIRRAYETGRGS**

23 SESURV_p1_1200 100.0% 99.9%  **PARFPNLLVNGAAGIAVGMATNIPPHNLTEVIDGVLSLSKNPDITINELMEDIQGPDFPTAGLVLGKSGIRRAYETGRGS**

24 SESURV_p3_0825 93.3% 99.8%  **PARFPNLLVNGAAGIAVGMATNIPPHNLTEVIDGVLSLSKNPDITINKLMEDIQGPDFPTAGLVLGKSGIRRAYETGRGS**

25 JH 100.0% 98.9%  **PARFPNLLVNGAAGIAVGMATNIPPHNLTEVIDGVLSLSKNPDITINELMEDIQGPDFPTAGLVLGKSGIRRAYETGRGS**

26 Z0118SE0132 93.3% 98.9%  **PARFPNLLVNGAAGIAVGMATNIPPHNLTEVIDGVLSLSKNPDITINELMEDVQGPDFPTAGLVLGKSGIRRAYETGRGS**

27 R5981 93.3% 98.9%  **PARFPNLLVNGAAGIAVGMATNIPPHNLTEVIDGVLSLSKNPDITINELMEDVQGPDFPTAGLVLGKSGIRRAYETGRGS**

28 JH-S-3 100.0% 98.9%  **PARFPNLLVNGAAGIAVGMATNIPPHNLTEVIDGVLSLSKNPDITINELMEDIQGPDFPTAGLVLGKSGIRRAYETGRGS**

29 JH-S-1 93.3% 98.8%  **PARFPNLLVNGAAGIAVGMATNIPPHNLTEVIDGVLSLSKNPDITINELMEDIQGPDFPTAGLVLGKSGIRRAYETGRGS**

30 s2 100.0% 99.1%  **PARFPNLLVNGAAGIAVGMATNIPPHNLTEVIDGVLSLSKNPDITINELMEDIQGPDFPTAGLVLGKSGIRRAYETGRGS**

31 S43 100.0% 99.1%  **PARFPNLLVNGAAGIAVGMATNIPPHNLTEVIDGVLSLSKNPDITINELMEDIQGPDFPTAGLVLGKSGIRRAYETGRGS**

32 S17W 93.3% 99.0%  **PARFPNLLVNGAAGIAVGMATNIPPHNLTEVIDGVLSLSKNPDITINELMEDIQGPDFPTAGLVLGKSGIRRAYETGRGS**

33 48 100.0% 99.0%  **PARFPNLLVNGAAGIAVGMATNIPPHNLTEVIDGVLSLSKNPDITINELMEDIQGPDFPTAGLVLGKSGIRRAYETGRGS**

34 ABKVF 100.0% 99.0%  **PARFPNLLVNGAAGIAVGMATNIPPHNLTEVIDGVLSLSKNPDITINELMEDIQGPDFPTAGLVLGKSGIRRAYETGRGS**

35 CICARIA 100.0% 99.9%  **PARFPNLLVNGAAGIAVGMATNIPPHNLTEVIDGVLSLSKNPDITINELMEDIQGPDFPTAGLVLGKSGIRRAYETGRGS**

36 SKN25lux 100.0% 99.8%  **PARFPNLLVNGAAGIAVGMATNIPPHNLTEVIDGVLSLSKNPDITINELMEDIQGPDFPTAGLVLGKSGIRRAYETGRGS**

37 HMSC074F11 100.0% 99.1%  **PARFPNLLVNGAAGIAVGMATNIPPHNLTEVIDGVLSLSKNPDITINELMEDIQGPDFPTAGLVLGKSGIRRAYETGRGS**

38 HMSC068G11 100.0% 99.1%  **PARFPNLLVNGAAGIAVGMATNIPPHNLTEVIDGVLSLSKNPDITINELMEDIQGPDFPTAGLVLGKSGIRRAYETGRGS**

39 NIHLM015 100.0% 99.1%  **PARFPNLLVNGAAGIAVGMATNIPPHNLTEVIDGVLSLSKNPDITINELMEDIQGPDFPTAGLVLGKSGIRRAYETGRGS**

consensus/100%  **PARFPNLLVNGAAGIAVGMATNIPPHNLTEVIDGVLSLSKNPDITINcLMEDlQGPDFPTAGLVLGKSGIRRAYETGRGS**

consensus/90%  **PARFPNLLVNGAAGIAVGMATNIPPHNLTEVIDGVLSLSKNPDITINELMEDIQGPDFPTAGLVLGKSGIRRAYETGRGS**

consensus/80%  **PARFPNLLVNGAAGIAVGMATNIPPHNLTEVIDGVLSLSKNPDITINELMEDIQGPDFPTAGLVLGKSGIRRAYETGRGS**

consensus/70%  **PARFPNLLVNGAAGIAVGMATNIPPHNLTEVIDGVLSLSKNPDITINELMEDIQGPDFPTAGLVLGKSGIRRAYETGRGS**

cov pid **241**  **: . . . . 3 . .** **320**

1 ATCC35984 100.0% 100.0%  **IQMRSRAEIEERGGGRQRIVVTEIPFQVNKARMIEKIAELVRDKKIDGITDLRDETSLRTGVRVVIDVRKDANASVILNN**

2 ATCC12228 93.3% 99.9%  **IQMRSRAEIEERGGGRQRIVVTEIPFQVNKARMIEKIAELVRDKKIDGITDLRDETSLRTGVRVVIDVRKDANASVILNN**

3 36AM 100.0% 99.0%  **IQMRSRAEIEERGGGRQRIVVTEIPFQVNKARMIEKIAELVRDKKIDGITDLRDETSLRTGVRVVIDVRKDANASVILNN**

4 48AF 100.0% 99.9%  **IQMRSRAEIEERGGGRQRIVVTEIPFQVNKARMIEKIAELVRDKKIDGITDLRDETSLRTGVRVVIDVRKDANASVILNN**

5 54AF 100.0% 99.0%  **IQMRSRAEIEERGGGRQRIVVTEIPFQVNKARMIEKIAELVRDKKIDGITDLRDETSLRTGVRVVIDVRKDANASVILNN**

6 NIHLM037 100.0% 99.1%  **IQMRSRAEIEERGGGRQRIVVTEIPFQVNKARMIEKIAELVRDKKIDGITDLRDETSLRTGVRVVIDVRKDANASVILNN**

7 785_SEPI 100.0% 99.0%  **IQMRSRAEIEERGGGRQRIVVTEIPFQVNKARMIEKIAELVRDKKIDGITDLRDETSLRTGVRVVIDVRKDANASVILNN**

8 VSE49 100.0% 99.1%  **IQMRSRAEIEERGGGRQRIVVTEIPFQVNKARMIEKIAELVRDKKIDGITDLRDETSLRTGVRVVIDVRKDANASVILNN**

9 VSE57 100.0% 99.1%  **IQMRSRAEIEERGGGRQRIVVTEIPFQVNKARMIEKIAELVRDKKIDGITDLRDETSLRTGVRVVIDVRKDANASVILNN**

10 SE45 100.0% 99.1%  **IQMRSRAEIEERGGGRQRIVVTEIPFQVNKARMIEKIAELVRDKKIDGITDLRDETSLRTGVRVVIDVRKDANASVILNN**

11 SE40 100.0% 99.1%  **IQMRSRAEIEERGGGRQRIVVTEIPFQVNKARMIEKIAELVRDKKIDGITDLRDETSLRTGVRVVIDVRKDANASVILNN**

12 32A 100.0% 99.1%  **IQMRSRAEIEERGGGRQRIVVTEIPFQVNKARMIEKIAELVRDKKIDGITDLRDETSLRTGVRVVIDVRKDANASVILNN**

13 HD66 100.0% 99.8%  **IQMRSRAEIEERGGGRQRIVVTEIPFQVNKARMIEKIAELVRDKKIDGITDLRDETSLRTGVRVVIDVRKDANASVILNN**

14 HD43 100.0% 99.9%  **IQMRSRAEIEERGGGRQRIVVTEIPFQVNKARMIEKIAELVRDKKIDGITDLRDETSLRTGVRVVIDVRKDANASVILNN**

15 HD33 100.0% 99.8%  **IQMRSRAEIEERGGGRQRIVVTEIPFQVNKARMIEKIAELVRDKKIDGITDLRDETSLRTGVRVVIDVRKDANASVILNN**

16 DE0525 100.0% 99.1%  **IQMRSRAEIEERGGGRQRIVVTEIPFQVNKARMIEKIAELVRDKKIDGITDLRDETSLRTGVRVVIDVRKDANASVILNN**

17 4928STDY7071543 100.0% 99.0%  **IQMRSRAEIEERGGGRQRIVVTEIPFQVNKARMIEKIAELVRDKKIDGITDLRDETSLRTGVRVVIDVRKDANASVILNN**

18 BAV2502 100.0% 99.0%  **IQMRSRAEIEERGGGRQRIVVTEIPFQVNKARMIEKIAELVRDKKIDGITDLRDETSLRTGVRVVIDVRKDANASVILNN**

19 APC3784 100.0% 99.0%  **IQMRSRAEIEERGGGRQRIVVTEIPFQVNKARMIEKIAELVRDKKIDGITDLRDETSLRTGVRVVIDVRKDANASVILNN**

20 SESURV_p1_0557 100.0% 99.9%  **IQMRSRAEIEERGGGRQRIVVTEIPFQVNKARMIEKIAELVRDKKIDGITDLRDETSLRTGVRVVIDVRKDANASVILNN**

21 SESURV_p1_0612 93.3% 99.0%  **IQMRSRAEIEERGGGRQRIVVTEIPFQVNKARMIEKIAELVRDKKIDGITDLRDETSLRTGVRVVIDVRKDANASVILNN**

22 SESURV_p4_1553 100.0% 99.9%  **IQMRSRAEIEERGGGRQRIVVTEIPFQVNKARMIEKIAELVRDKKIDGITDLRDETSLRTGVRVVIDVRKDANASVILNN**

23 SESURV_p1_1200 100.0% 99.9%  **IQMRSRAEIEERGGGRQRIVVTEIPFQVNKARMIEKIAELVRDKKIDGITDLRDETSLRTGVRVVIDVRKDANASVILNN**

24 SESURV_p3_0825 93.3% 99.8%  **IQMRSRAEIEERGGGRQRIVVTEIPFQVNKARMIEKIAELVRDKKIDGITDLRDETSLRTGVRVVIDVRKDANASVILNN**

25 JH 100.0% 98.9%  **IQMRSRAEIEERGGGRQRIVVTEIPFQVNKARMIEKIAELVRDKKIDGITDLRDETSLRTGVRVVIDVRKDANASVILNN**

26 Z0118SE0132 93.3% 98.9%  **IQMRSRAEIEERGGGRQRIVVTEIPFQVNKARMIEKIAELVRDKKIDGITDLRDETSLRTGVRVVIDVRKDANASVILNN**

27 R5981 93.3% 98.9%  **IQMRSRAEIEERGGGRQRIVVTEIPFQVNKARMIEKIAELVRDKKIDGITDLRDETSLRTGVRVVIDVRKDANASVILNN**

28 JH-S-3 100.0% 98.9%  **IQMRSRAEIEERGGGRQRIVVTEIPFQVNKARMIEKIAELVRDKKIDGITDLRDETSLRTGVRVVIDVRKDANASVILNN**

29 JH-S-1 93.3% 98.8%  **IQMRSRAEIEERGGGRQRIVVTEIPFQVNKARMIEKIAELVRDKKIDGITDLRDETSLRTGVRVVIDVRKDANASVILNN**

30 s2 100.0% 99.1%  **IQMRSRAEIEERGGGRQRIVVTEIPFQVNKARMIEKIAELVRDKKIDGITDLRDETSLRTGVRVVIDVRKDANASVILNN**

31 S43 100.0% 99.1%  **IQMRSRAEIEERGGGRQRIVVTEIPFQVNKARMIEKIAELVRDKKIDGITDLRDETSLRTGVRVVIDVRKDANASVILNN**

32 S17W 93.3% 99.0%  **IQMRSRAEIEERGGGRQRIVVTEIPFQVNKARMIEKIAELVRDKKIDGITDLRDETSLRTGVRVVIDVRKDANASVILNN**

33 48 100.0% 99.0%  **IQMRSRAEIEERGGGRQRIVVTEIPFQVNKARMIEKIAELVRDKKIDGITDLRDETSLRTGVRVVIDVRKDANASVILNN**

34 ABKVF 100.0% 99.0%  **IQMRSRAEIEERGGGRQRIVVTEIPFQVNKARMIEKIAELVRDKKIDGITDLRDETSLRTGVRVVIDVRKDANASVILNN**

35 CICARIA 100.0% 99.9%  **IQMRSRAEIEERGGGRQRIVVTEIPFQVNKARMIEKIAELVRDKKIDGITDLRDETSLRTGVRVVIDVRKDANASVILNN**

36 SKN25lux 100.0% 99.8%  **IQMRSRAEIEERGGGRQRIVVTEIPFQVNKARMIEKIAELVRDKKIDGITDLRDETSLRTGVRVVIDVRKDANASVILNN**

37 HMSC074F11 100.0% 99.1%  **IQMRSRAEIEERGGGRQRIVVTEIPFQVNKARMIEKIAELVRDKKIDGITDLRDETSLRTGVRVVIDVRKDANASVILNN**

38 HMSC068G11 100.0% 99.1%  **IQMRSRAEIEERGGGRQRIVVTEIPFQVNKARMIEKIAELVRDKKIDGITDLRDETSLRTGVRVVIDVRKDANASVILNN**

39 NIHLM015 100.0% 99.1%  **IQMRSRAEIEERGGGRQRIVVTEIPFQVNKARMIEKIAELVRDKKIDGITDLRDETSLRTGVRVVIDVRKDANASVILNN**

consensus/100%  **IQMRSRAEIEERGGGRQRIVVTEIPFQVNKARMIEKIAELVRDKKIDGITDLRDETSLRTGVRVVIDVRKDANASVILNN**

consensus/90%  **IQMRSRAEIEERGGGRQRIVVTEIPFQVNKARMIEKIAELVRDKKIDGITDLRDETSLRTGVRVVIDVRKDANASVILNN**

consensus/80%  **IQMRSRAEIEERGGGRQRIVVTEIPFQVNKARMIEKIAELVRDKKIDGITDLRDETSLRTGVRVVIDVRKDANASVILNN**

consensus/70%  **IQMRSRAEIEERGGGRQRIVVTEIPFQVNKARMIEKIAELVRDKKIDGITDLRDETSLRTGVRVVIDVRKDANASVILNN**

cov pid **321**  **. . : . . . . 4** **400**

1 ATCC35984 100.0% 100.0%  **LYKQTPLQTSFGVNMIALVNSRPKLINLKEALIHYLEHQKTVVRRRTEYNLKKARDRAHILEGLRIALDHIDEIITTIRE**

2 ATCC12228 93.3% 99.9%  **LYKQTPLQTSFGVNMIALVNGRPKLINLKEALIHYLEHQKTVVRRRTEYNLKKARDRAHILEGLRIALDHIDEIITTIRE**

3 36AM 100.0% 99.0%  **LYKQTPLQTSFGVNMIALVNGRPKLINLKEALIHYLEHQKTVVRRRTEYNLKKARDRAHILEGLRIALDHIDEIITTIRE**

4 48AF 100.0% 99.9%  **LYKQTPLQTSFGVNMIALVNGRPKLINLKEALIHYLEHQKTVVRRRTEYNLKKARDRAHILEGLRIALDHIDEIITTIRE**

5 54AF 100.0% 99.0%  **LYKQTPLQTSFGVNMIALVNGRPKLINLKEALIHYLEHQKTVVRRRTEYNLKKARDRAHILEGLRIALDHIDEIITTIRE**

6 NIHLM037 100.0% 99.1%  **LYKQTPLQTSFGVNMIALVNGRPKLINLKEALIHYLEHQKTVVRRRTEYNLKKARDRAHILEGLRIALDHIDEIITTIRE**

7 785_SEPI 100.0% 99.0%  **LYKQTPLQTSFGVNMIALVNGRPKLINLKEALIHYLEHQKTVVRRRTEYNLKKARDRAHILEGLRIALDHIDEIITTIRE**

8 VSE49 100.0% 99.1%  **LYKQTPLQTSFGVNMIALVNGRPKLINLKEALIHYLEHQKTVVRRRTEYNLKKARDRAHILEGLRIALDHIDEIITTIRE**

9 VSE57 100.0% 99.1%  **LYKQTPLQTSFGVNMIALVNGRPKLINLKEALIHYLEHQKTVVRRRTEYNLKKARDRAHILEGLRIALDHIDEIITTIRE**

10 SE45 100.0% 99.1%  **LYKQTPLQTSFGVNMIALVNGRPKLINLKEALIHYLEHQKTVVRRRTEYNLKKARDRAHILEGLRIALDHIDEIITTIRE**

11 SE40 100.0% 99.1%  **LYKQTPLQTSFGVNMIALVNGRPKLINLKEALIHYLEHQKTVVRRRTEYNLKKARDRAHILEGLRIALDHIDEIITTIRE**

12 32A 100.0% 99.1%  **LYKQTPLQTSFGVNMIALVNGRPKLINLKEALIHYLEHQKTVVRRRTEYNLKKARDRAHILEGLRIALDHIDEIITTIRE**

13 HD66 100.0% 99.8%  **LYKQTPLQTSFGVNMIALVNGRPKLINLKEALIHYLEHQKTVVRRRTEYNLKKARDRAHILEGLRIALDHIDEIITTIRE**

14 HD43 100.0% 99.9%  **LYKQTPLQTSFGVNMIALVNSRPKLINLKEALIHYLEHQKTVVRRRTEYNLKKARDRAHILEGLRIALDHIDEIITTIRE**

15 HD33 100.0% 99.8%  **LYKQTPLQTSFGVNMIALVNGRPKLINLKEALIHYLEHQKTVVRRRTEYNLKKARDRAHILEGLRIALDHIDEIITTIRE**

16 DE0525 100.0% 99.1%  **LYKQTPLQTSFGVNMIALVNGRPKLINLKEALIHYLEHQKTVVRRRTEYNLKKARDRAHILEGLRIALDHIDEIITTIRE**

17 4928STDY7071543 100.0% 99.0%  **LYKQTPLQTSFGVNMIALVNGRPKLINLKEALIHYLEHQKTVVRRRTEYNLKKARDRAHILEGLRIALDHIDEIITTIRE**

18 BAV2502 100.0% 99.0%  **LYKQTPLQTSFGVNMIALVNGRPKLINLKEALIHYLEHQKTVVRRRTEYNLKKARDRAHILEGLRIALDHIDEIITTIRE**

19 APC3784 100.0% 99.0%  **LYKQTPLQTSFGVNMIALVNGRPKLINLKEALIHYLEHQKTVVRRRTEYNLKKARDRAHILEGLRIALDHIDEIITTIRE**

20 SESURV_p1_0557 100.0% 99.9%  **LYKQTPLQTSFGVNMIALVNGRPKLINLKEALIHYLEHQKTVVRRRTEYNLKKARDRAHILEGLRIALDHIDEIITTIRE**

21 SESURV_p1_0612 93.3% 99.0%  **LYKQTPLQTSFGVNMIALVNGRPKLINLKEALIHYLEHQKTVVRRRTEYNLKKARDRAHILEGLRIALDHIDEIITTIRE**

22 SESURV_p4_1553 100.0% 99.9%  **LYKQTPLQTSFGVNMIALVNGRPKLINLKEALIHYLEHQKTVVRRRTEYNLKKARDRAHILEGLRIALDHIDEIITTIRE**

23 SESURV_p1_1200 100.0% 99.9%  **LYKQTPLQTSFGVNMIALVNGRPKLINLKEALIHYLEHQKTVVRRRTEYNLKKARDRAHILEGLRIALDHIDEIITTIRE**

24 SESURV_p3_0825 93.3% 99.8%  **LYKQTPLQTSFGVNMIALVNGRPKLINLKEALIHYLEHQKTVVRRRTEYNLKKARDRAHILEGLRIALDHIDEIITTIRE**

25 JH 100.0% 98.9%  **LYKQTPLQTSFGMNMIALVNGRPKLINLKEALIHYLEHQKTVVRRRTEYNLKKARDRAHILEGLRIALDHIDEIITTIRE**

26 Z0118SE0132 93.3% 98.9%  **LYKQTPLQTSFGVNMIALVNGRPKLINLKEALIHYLEHQKTVVRRRTEYNLKKARDRAHILEGLRIALDHIDEIITTIRE**

27 R5981 93.3% 98.9%  **LYKQTPLQTSFGVNMIALVNGRPKLINLKEALIHYLEHQKTVVRRRTEYNLKKARDRAHILEGLRIALDHIDEIITTIRE**

28 JH-S-3 100.0% 98.9%  **LYKQTPLQTSFGMNMIALVNGRPKLINLKEALIHYLEHQKTVVRRRTEYNLKKARDRAHILEGLRIALDHIDEIITTIRE**

29 JH-S-1 93.3% 98.8%  **LYKQTPLQTSFGMNMIALVNGRPKLINLKEALIHYLEHQKTVVRRRTEYNLKKARDRAHILEGLRIALDHIDEIITTIRE**

30 s2 100.0% 99.1%  **LYKQTPLQTSFGVNMIALVNGRPKLINLKEALIHYLEHQKTVVRRRTEYNLKKARDRAHILEGLRIALDHIDEIITTIRE**

31 S43 100.0% 99.1%  **LYKQTPLQTSFGVNMIALVNGRPKLINLKEALIHYLEHQKTVVRRRTEYNLKKARDRAHILEGLRIALDHIDEIITTIRE**

32 S17W 93.3% 99.0%  **LYKQTPLQTSFGVNMIALVNGRPKLINLKEALIHYLEHQKTVVRRRTEYNLKKARDRAHILEGLRIALDHIDEIITTIRE**

33 48 100.0% 99.0%  **LYKQTPLQTSFGVNMIALVNGRPKLINLKEALIHYLEHQKTVVRRRTEYNLKKARDRAHILEGLRIALDHIDEIITTIRE**

34 ABKVF 100.0% 99.0%  **LYKQTPLQTSFGVNMIALVNGRPKLINLKEALIHYLEHQKTVVRRRTEYNLKKARDRAHILEGLRIALDHIDEIITTIRE**

35 CICARIA 100.0% 99.9%  **LYKQTPLQTSFGVNMIALVNGRPKLINLKEALIHYLEHQKTVVRRRTEYNLKKARDRAHILEGLRIALDHIDEIITTIRE**

36 SKN25lux 100.0% 99.8%  **LYKQTPLQTSFGVNMIALVNGRPKLINLKEALIHYLEHQKTVVRRRTEYNLKKARDRAHILEGLRIALDHIDEIITTIRE**

37 HMSC074F11 100.0% 99.1%  **LYKQTPLQTSFGVNMIALVNGRPKLINLKEALIHYLEHQKTVVRRRTEYNLKKARDRAHILEGLRIALDHIDEIITTIRE**

38 HMSC068G11 100.0% 99.1%  **LYKQTPLQTSFGVNMIALVNGRPKLINLKEALIHYLEHQKTVVRRRTEYNLKKARDRAHILEGLRIALDHIDEIITTIRE**

39 NIHLM015 100.0% 99.1%  **LYKQTPLQTSFGVNMIALVNGRPKLINLKEALIHYLEHQKTVVRRRTEYNLKKARDRAHILEGLRIALDHIDEIITTIRE**

consensus/100%  **LYKQTPLQTSFGhNMIALVNuRPKLINLKEALIHYLEHQKTVVRRRTEYNLKKARDRAHILEGLRIALDHIDEIITTIRE**

consensus/90%  **LYKQTPLQTSFGVNMIALVNGRPKLINLKEALIHYLEHQKTVVRRRTEYNLKKARDRAHILEGLRIALDHIDEIITTIRE**

consensus/80%  **LYKQTPLQTSFGVNMIALVNGRPKLINLKEALIHYLEHQKTVVRRRTEYNLKKARDRAHILEGLRIALDHIDEIITTIRE**

consensus/70%  **LYKQTPLQTSFGVNMIALVNGRPKLINLKEALIHYLEHQKTVVRRRTEYNLKKARDRAHILEGLRIALDHIDEIITTIRE**

cov pid **401**  **. . . . : . . .** **480**

1 ATCC35984 100.0% 100.0%  **SDTDKIAMASLQERFKLTERQAQAILDMRLRRLTGLERDKIESEYNELLEYIKELEEILADEEVLLQLVRDELTEIKERF**

2 ATCC12228 93.3% 99.9%  **SDTDKIAMASLQERFKLTERQAQAILDMRLRRLTGLERDKIESEYNELLEYIKELEEILADEEVLLQLVRDELTEIKERF**

3 36AM 100.0% 99.0%  **SDTDKIAMASLQERFKLTERQAQAILDMRLRRLTGLERDKIESEYNELLEYIKELEEILADEEVLLQLVRDELTEIKERF**

4 48AF 100.0% 99.9%  **SDTDKIAMASLQERFKLTERQAQAILDMRLRRLTGLERDKIESEYNELLEYIKELEEILADEEVLLQLVRDELTEIKERF**

5 54AF 100.0% 99.0%  **SDTDKIAMASLQERFKLTERQAQAILDMRLRRLTGLERDKIESEYNELLEYIKELEEILADEEVLLQLVRDELTEIKERF**

6 NIHLM037 100.0% 99.1%  **SDTDKIAMASLQERFKLTERQAQAILDMRLRRLTGLERDKIESEYNELLEYIKELEEILADEEVLLQLVRDELTEIKERF**

7 785_SEPI 100.0% 99.0%  **SDTDKIAMASLQERFKLTERQAQAILDMRLRRLTGLERDKIESEYNELLEYIKELEEILADEEVLLQLVRDELTEIKERF**

8 VSE49 100.0% 99.1%  **SDTDKIAMASLQERFKLTERQAQAILDMRLRRLTGLERDKIESEYNELLEYIKELEEILADEEVLLQLVRDELTEIKERF**

9 VSE57 100.0% 99.1%  **SDTDKIAMASLQERFKLTERQAQAILDMRLRRLTGLERDKIESEYNELLEYIKELEEILADEEVLLQLVRDELTEIKERF**

10 SE45 100.0% 99.1%  **SDTDKIAMASLQERFKLTERQAQAILDMRLRRLTGLERDKIESEYNELLEYIKELEEILADEEVLLQLVRDELTEIKERF**

11 SE40 100.0% 99.1%  **SDTDKIAMASLQERFKLTERQAQAILDMRLRRLTGLERDKIESEYNELLEYIKELEEILADEEVLLQLVRDELTEIKERF**

12 32A 100.0% 99.1%  **SDTDKIAMASLQERFKLTERQAQAILDMRLRRLTGLERDKIESEYNELLEYIKELEEILADEEVLLQLVRDELTEIKERF**

13 HD66 100.0% 99.8%  **SDTDKIAMASLQERFKLTERQAQAILDMRLRRLTGLERDKIESEYNELLEYIKELEEILADEEVLLQLVRDELTEIKERF**

14 HD43 100.0% 99.9%  **SDTDKIAMASLQERFKLTERQAQAILDMRLRRLTGLERDKIESEYNELLEYIKELEEILADEEVLLQLVRDELTEIKERF**

15 HD33 100.0% 99.8%  **SDTDKIAMASLQERFKLTERQAQAILDMRLRRLTGLERDKIESEYNELLEYIKELEEILADEEVLLQLVRDELTEIKERF**

16 DE0525 100.0% 99.1%  **SDTDKIAMASLQERFKLTERQAQAILDMRLRRLTGLERDKIESEYNELLEYIKELEEILADEEVLLQLVRDELTEIKERF**

17 4928STDY7071543 100.0% 99.0%  **SDTDKIAMASLQERFKLTERQAQAILDMRLRRLTGLERDKIESEYNELLEYIKELEEILADEEVLLQLVRDELTEIKERF**

18 BAV2502 100.0% 99.0%  **SDTDKIAMASLQERFKLTERQAQAILDMRLRRLTGLERDKIESEYNELLEYIKELEEILADEEVLLQLVRDELTEIKERF**

19 APC3784 100.0% 99.0%  **SDTDKIAMASLQERFKLTERQAQAILDMRLRRLTGLERDKIESEYNELLEYIKELEEILADEEVLLQLVRDELTEIKERF**

20 SESURV_p1_0557 100.0% 99.9%  **SDTDKIAMASLQERFKLTERQAQAILDMRLRRLTGLERDKIESEYNELLEYIKELEEILADEEVLLQLVRDELTEIKERF**

21 SESURV_p1_0612 93.3% 99.0%  **SDTDKIAMASLQERFKLTERQAQAILDMRLRRLTGLERDKIESEYNELLEYIKELEEILADEEVLLQLVRDELTEIKERF**

22 SESURV_p4_1553 100.0% 99.9%  **SDTDKIAMASLQERFKLTERQAQAILDMRLRRLTGLERDKIESEYNELLEYIKELEEILADEEVLLQLVRDELTEIKERF**

23 SESURV_p1_1200 100.0% 99.9%  **SDTDKIAMASLQERFKLTERQAQAILDMRLRRLTGLERDKIESEYNELLEYIKELEEILADEEVLLQLVRDELTEIKERF**

24 SESURV_p3_0825 93.3% 99.8%  **SDTDKIAMASLQERFKLTERQAQAILDMRLRRLTGLERDKIESEYNELLEYIKELEEILADEEVLLQLVRDELTEIKERF**

25 JH 100.0% 98.9%  **SDTDKIAMASLQERFKLTERQAQAILDMRLRRLTGLERDKIESEYNELLEYIKELEEILADEEVLLQLVRDELTEIKERF**

26 Z0118SE0132 93.3% 98.9%  **SDTDKIAMASLQERFKLTERQAQAILDMRLRRLTGLERDKIESEYNELLEYIKELEEILADEEVLLQLVRDELTEIKERF**

27 R5981 93.3% 98.9%  **SDTDKIAMASLQERFKLTERQAQAILDMRLRRLTGLERDKIESEYNELLEYIKELEEILADEEVLLQLVRDELTEIKERF**

28 JH-S-3 100.0% 98.9%  **SDTDKIAMASLQERFKLTERQAQAILDMRLRRLTGLERDKIESEYNELLEYIKELEEILADEEVLLQLVRDELTEIKERF**

29 JH-S-1 93.3% 98.8%  **SDTDKIAMASLQERFKLTERQAQAILDMRLRRLTGLERDKIESEYNELLEYIKELEEILADEEVLLQLVRDELTEIKERF**

30 s2 100.0% 99.1%  **SDTDKIAMASLQERFKLTERQAQAILDMRLRRLTGLERDKIESEYNELLEYIKELEEILADEEVLLQLVRDELTEIKERF**

31 S43 100.0% 99.1%  **SDTDKIAMASLQERFKLTERQAQAILDMRLRRLTGLERDKIESEYNELLEYIKELEEILADEEVLLQLVRDELTEIKERF**

32 S17W 93.3% 99.0%  **SDTDKIAMASLQERFKLTERQAQAILDMRLRRLTGLERDKIESEYNELLEYIKELEEILADEEVLLQLVRDELTEIKERF**

33 48 100.0% 99.0%  **SDTDKIAMASLQERFKLTERQAQAILDMRLRRLTGLERDKIESEYNELLEYIKELEEILADEEVLLQLVRDELTEIKERF**

34 ABKVF 100.0% 99.0%  **SDTDKIAMASLQERFKLTERQAQAILDMRLRRLTGLERDKIESEYNELLEYIKELEEILADEEVLLQLVRDELTKIKERF**

35 CICARIA 100.0% 99.9%  **SDTDKIAMASLQERFKLTERQAQAILDMRLRRLTGLERDKIESEYNELLEYIKELEEILADEEVLLQLVRDELTEIKERF**

36 SKN25lux 100.0% 99.8%  **SDTDKIAMASLQERFKLTERQAQAILDMRLRRLTGLERDKIESEYNELLEYIKELEEILADEEVLLQLVRDELTEIKERF**

37 HMSC074F11 100.0% 99.1%  **SDTDKIAMASLQERFKLTERQAQAILDMRLRRLTGLERDKIESEYNELLEYIKELEEILADEEVLLQLVRDELTEIKERF**

38 HMSC068G11 100.0% 99.1%  **SDTDKIAMASLQERFKLTERQAQAILDMRLRRLTGLERDKIESEYNELLEYIKELEEILADEEVLLQLVRDELTEIKERF**

39 NIHLM015 100.0% 99.1%  **SDTDKIAMASLQERFKLTERQAQAILDMRLRRLTGLERDKIESEYNELLEYIKELEEILADEEVLLQLVRDELTEIKERF**

consensus/100%  **SDTDKIAMASLQERFKLTERQAQAILDMRLRRLTGLERDKIESEYNELLEYIKELEEILADEEVLLQLVRDELTcIKERF**

consensus/90%  **SDTDKIAMASLQERFKLTERQAQAILDMRLRRLTGLERDKIESEYNELLEYIKELEEILADEEVLLQLVRDELTEIKERF**

consensus/80%  **SDTDKIAMASLQERFKLTERQAQAILDMRLRRLTGLERDKIESEYNELLEYIKELEEILADEEVLLQLVRDELTEIKERF**

consensus/70%  **SDTDKIAMASLQERFKLTERQAQAILDMRLRRLTGLERDKIESEYNELLEYIKELEEILADEEVLLQLVRDELTEIKERF**

cov pid **481**  **. 5 . . . . : .** **560**

1 ATCC35984 100.0% 100.0%  **GDERRTEIQLGGLEDLEDEDLIPEEQIVITLSHNNYIKRLPVSTYRSQNRGGRGIQGMNTLDEDFVSQLVTMSTHDHVLF**

2 ATCC12228 93.3% 99.9%  **GDERRTEIQLGGLEDLEDEDLIPEEQIVITLSHNNYIKRLPVSTYRSQNRGGRGIQGMNTLDEDFVSQLVTMSTHDHVLF**

3 36AM 100.0% 99.0%  **GDERRTEIQLGGLEDLEDEDLIPEEQIVITLSHNNYIKRLPVSTYRSQNRGGRGIQGMNTLDEDFVSQLVTMSTHDHVLF**

4 48AF 100.0% 99.9%  **GDERRTEIQLGGLEDLEDEDLIPEEQIVITLSHNNYIKRLPVSTYRSQNRGGRGIQGMNTLDEDFVSQLVTMSTHDHVLF**

5 54AF 100.0% 99.0%  **GDERRTEIQLGGLEDLEDEDLIPEEQIVITLSHNNYIKRLPVSTYRSQNRGGRGIQGMNTLDEDFVSQLVTMSTHDHVLF**

6 NIHLM037 100.0% 99.1%  **GDERRTEIQLGGLEDLEDEDLIPEEQIVITLSHNNYIKRLPVSTYRSQNRGGRGIQGMNTLDEDFVSQLVTMSTHDHVLF**

7 785_SEPI 100.0% 99.0%  **GDERRTEIQLGGLEDLEDEDLIPEEQIVITLSHNNYIKRLPVSTYRSQNRGGRGIQGMNTLDEDFVSQLVTMSTHDHVLF**

8 VSE49 100.0% 99.1%  **GDERRTEIQLGGLEDLEDEDLIPEEQIVITLSHNNYIKRLPVSTYRSQNRGGRGIQGMNTLDEDFVSQLVTMSTHDHVLF**

9 VSE57 100.0% 99.1%  **GDERRTEIQLGGLEDLEDEDLIPEEQIVITLSHNNYIKRLPVSTYRSQNRGGRGIQGMNTLDEDFVSQLVTMSTHDHVLF**

10 SE45 100.0% 99.1%  **GDERRTEIQLGGLEDLEDEDLIPEEQIVITLSHNNYIKRLPVSTYRSQNRGGRGIQGMNTLDEDFVSQLVTMSTHDHVLF**

11 SE40 100.0% 99.1%  **GDERRTEIQLGGLEDLEDEDLIPEEQIVITLSHNNYIKRLPVSTYRSQNRGGRGIQGMNTLDEDFVSQLVTMSTHDHVLF**

12 32A 100.0% 99.1%  **GDERRTEIQLGGLEDLEDEDLIPEEQIVITLSHNNYIKRLPVSTYRSQNRGGRGIQGMNTLDEDFVSQLVTMSTHDHVLF**

13 HD66 100.0% 99.8%  **GDERRTEIQLGGLEDLEDEDLIPEEQIVITLSHNNYIKRLPVSTYRSQNRGGRGIQGMNTLDEDFVSQLVTMSTHDHVLF**

14 HD43 100.0% 99.9%  **GDERRTEIQLGGLEDLEDEDLIPEEQIVITLSHNNYIKRLPVSTYRSQNRGGRGIQGMNTLDEDFVSQLVTMSTHDHVLF**

15 HD33 100.0% 99.8%  **GDERRTEIQLGGLEDLEDEDLIPEEQIVITLSHNNYIKRLPVSTYRSQNRGGRGIQGMNTLDEDFVSQLVTMSTHDHVLF**

16 DE0525 100.0% 99.1%  **GDERRTEIQLGGLEDLEDEDLIPEEQIVITLSHNNYIKRLPVSTYRSQNRGGRGIQGMNTLDEDFVSQLVTMSTHDHVLF**

17 4928STDY7071543 100.0% 99.0%  **GDERRTEIQLGGLEDLEDEDLIPEEQIVITLSHNNYIKRLPVSTYRSQNRGGRGIQGMNTLDEDFVSQLVTMSTHDHVLF**

18 BAV2502 100.0% 99.0%  **GDERRTEIQLGGLEDLEDEDLIPEEQIVITLSHNNYIKRLPVSTYRSQNRGGRGIQGMNTLDEDFVSQLVTMSTHDHVLF**

19 APC3784 100.0% 99.0%  **GDERRTEIQLGGLEDLEDEDLIPEEQIVITLSHNNYIKRLPVSTYRSQNRGGRGIQGMNTLDEDFVSQLVTMSTHDHVLF**

20 SESURV_p1_0557 100.0% 99.9%  **GDERRTEIQLGGLEDLEDEDLIPEEQIVITLSHNNYIKRLPVSTYRSQNRGGRGIQGMNTLDEDFVSQLVTMSTHDHVLF**

21 SESURV_p1_0612 93.3% 99.0%  **GDERRTEIQLGGLEDLEDEDLIPEEQIVITLSHNNYIKRLPVSTYRSQNRGGRGIQGMNTLDEDFVSQLVTMSTHDHVLF**

22 SESURV_p4_1553 100.0% 99.9%  **GDERRTEIQLGGLEDLEDEDLIPEEQIVITLSHNNYIKRLPVSTYRSQNRGGRGIQGMNTLDEDFVSQLVTMSTHDHVLF**

23 SESURV_p1_1200 100.0% 99.9%  **GDERRTEIQLGGLEDLEDEDLIPEEQIVITLSHNNYIKRLPVSTYRSQNRGGRGIQGMNTLDEDFVSQLVTMSTHDHVLF**

24 SESURV_p3_0825 93.3% 99.8%  **GDERRTEIQLGGLEDLEDEDLIPEEQIVITLSHNNYIKRLPVSTYRSQNRGGRGIQGMNTLDEDFVSQLVTMSTHDHVLF**

25 JH 100.0% 98.9%  **GDERRTEIQLGGLEDLEDEDLIPEEQIVITLSHNNYIKRLPVSTYRSQNRGGRGIQGMNTLDEDFVSQLVTMSTHDHVLF**

26 Z0118SE0132 93.3% 98.9%  **GDERRTEIQLGGLEDLEDEDLIPEEQIVITLSHNNYIKRLPVSTYRSQNRGGRGIQGMNTLDEDFVSQLVTMSTHDHVLF**

27 R5981 93.3% 98.9%  **GDERRTEIQLGGLEDLEDEDLIPEEQIVITLSHNNYIKRLPVSTYRSQNRGGRGIQGMNTLDEDFVSQLVTMSTHDHVLF**

28 JH-S-3 100.0% 98.9%  **GDERRTEIQLGGLEDLEDEDLIPEEQIVITLSHNNYIKRLPVSTYRSQNRGGRGIQGMNTLDEDFVSQLVTMSTHDHVLF**

29 JH-S-1 93.3% 98.8%  **GDERRTEIQLGGLEDLEDEDLIPEEQIVITLSHNNYIKRLPVSTYRSQNRGGRGIQGMNTLDEDFVSQLVTMSTHDHVLF**

30 s2 100.0% 99.1%  **GDERRTEIQLGGLEDLEDEDLIPEEQIVITLSHNNYIKRLPVSTYRSQNRGGRGIQGMNTLDEDFVSQLVTMSTHDHVLF**

31 S43 100.0% 99.1%  **GDERRTEIQLGGLEDLEDEDLIPEEQIVITLSHNNYIKRLPVSTYRSQNRGGRGIQGMNTLDEDFVSQLVTMSTHDHVLF**

32 S17W 93.3% 99.0%  **GDERRTEIQLGGLEDLEDEDLIPEEQIVITLSHNNYIKRLPVSTYRSQNRGGRGIQGMNTLDEDFVSQLVTMSTHDHVLF**

33 48 100.0% 99.0%  **GDERRTEIQLGGLEDLEDEDLIPEEQIVITLSHNNYIKRLPVSTYRSQNRGGRGIQGMNTLDEDFVSQLVTMSTHDHVLF**

34 ABKVF 100.0% 99.0%  **GDERRTEIQLGGLEDLEDEDLIPEEQIVITLSHNNYIKRLPVSTYRSQNRGGRGIQGMNTLDEDFVSQLVTMSTHDHVLF**

35 CICARIA 100.0% 99.9%  **GDERRTEIQLGGLEDLEDEDLIPEEQIVITLSHNNYIKRLPVSTYRSQNRGGRGIQGMNTLDEDFVSQLVTMSTHDHVLF**

36 SKN25lux 100.0% 99.8%  **GDERRTEIQLGGLEDLEDEDLIPEEQIVITLSHNNYIKRLPVSTYRSQNRGGRGIQGMNTLDEDFVSQLVTMSTHDHVLF**

37 HMSC074F11 100.0% 99.1%  **GDERRTEIQLGGLEDLEDEDLIPEEQIVITLSHNNYIKRLPVSTYRSQNRGGRGIQGMNTLDEDFVSQLVTMSTHDHVLF**

38 HMSC068G11 100.0% 99.1%  **GDERRTEIQLGGLEDLEDEDLIPEEQIVITLSHNNYIKRLPVSTYRSQNRGGRGIQGMNTLDEDFVSQLVTMSTHDHVLF**

39 NIHLM015 100.0% 99.1%  **GDERRTEIQLGGLEDLEDEDLIPEEQIVITLSHNNYIKRLPVSTYRSQNRGGRGIQGMNTLDEDFVSQLVTMSTHDHVLF**

consensus/100%  **GDERRTEIQLGGLEDLEDEDLIPEEQIVITLSHNNYIKRLPVSTYRSQNRGGRGIQGMNTLDEDFVSQLVTMSTHDHVLF**

consensus/90%  **GDERRTEIQLGGLEDLEDEDLIPEEQIVITLSHNNYIKRLPVSTYRSQNRGGRGIQGMNTLDEDFVSQLVTMSTHDHVLF**

consensus/80%  **GDERRTEIQLGGLEDLEDEDLIPEEQIVITLSHNNYIKRLPVSTYRSQNRGGRGIQGMNTLDEDFVSQLVTMSTHDHVLF**

consensus/70%  **GDERRTEIQLGGLEDLEDEDLIPEEQIVITLSHNNYIKRLPVSTYRSQNRGGRGIQGMNTLDEDFVSQLVTMSTHDHVLF**

cov pid **561**  **. . . 6 . . . .** **640**

1 ATCC35984 100.0% 100.0%  **FTNKGRVYKLKGYEVPELSRQSKGIPIINAIELENDETISTMIAVKDLESEEDYLVFATKQGIVKRSSLSNFSRINKNGK**

2 ATCC12228 93.3% 99.9%  **FTNKGRVYKLKGYEVPELSRQSKGIPIINAIELENDETISTMIAVKDLESEEDYLVFATKQGIVKRSSLSNFSRINKNGK**

3 36AM 100.0% 99.0%  **FTNKGRVYKLKGYEVPELSRQSKGIPIINAIELENDETISTMIAVKDLESEEDYLVFATKQGIVKRSSLSNFSRINKNGK**

4 48AF 100.0% 99.9%  **FTNKGRVYKLKGYEVPELSRQSKGIPIINAIELENDETISTMIAVKDLESEEDYLVFATKQGIVKRSSLSNFSRINKNGK**

5 54AF 100.0% 99.0%  **FTNKGRVYKLKGYEVPELSRQSKGIPIINAIELENDETISTMIAVKDLESEEDYLVFATKQGIVKRSSLSNFSRINKNGK**

6 NIHLM037 100.0% 99.1%  **FTNKGRVYKLKGYEVPELSRQSKGIPIINAIELENDETISTMIAVKDLESEEDYLVFATKQGIVKRSSLSNFSRINKNGK**

7 785_SEPI 100.0% 99.0%  **FTNKGRVYKLKGYEVPELSRQSKGIPIINAIELENDETISTMIAVKDLESEEDYLVFATKQGIVKRSSLSNFSRINKNGK**

8 VSE49 100.0% 99.1%  **FTNKGRVYKLKGYEVPELSRQSKGIPIINAIELENDETISTMIAVKDLESEEDYLVFATKQGIVKRSSLSNFSRINKNGK**

9 VSE57 100.0% 99.1%  **FTNKGRVYKLKGYEVPELSRQSKGIPIINAIELENDETISTMIAVKDLESEEDYLVFATKQGIVKRSSLSNFSRINKNGK**

10 SE45 100.0% 99.1%  **FTNKGRVYKLKGYEVPELSRQSKGIPIINAIELENDETISTMIAVKDLESEEDYLVFATKQGIVKRSSLSNFSRINKNGK**

11 SE40 100.0% 99.1%  **FTNKGRVYKLKGYEVPELSRQSKGIPIINAIELENDETISTMIAVKDLESEEDYLVFATKQGIVKRSSLSNFSRINKNGK**

12 32A 100.0% 99.1%  **FTNKGRVYKLKGYEVPELSRQSKGIPIINAIELENDETISTMIAVKDLESEEDYLVFATKQGIVKRSSLSNFSRINKNGK**

13 HD66 100.0% 99.8%  **FTNKGRVYKLKGYEVPELSRQSKGIPIINAIELENDETISTMIAVKDLESEEDYLVFATKQGIVKRSSLSNFSRINKNGK**

14 HD43 100.0% 99.9%  **FTNKGRVYKLKGYEVPELSRQSKGIPIINAIELENDETISTMIAVKDLESEEDYLVFATKQGIVKRSSLSNFSRINKNGK**

15 HD33 100.0% 99.8%  **FTNKGRVYKLKGYEVPELSRQSKGIPIINAIELENDETISTMIAVKDLESEEDYLVFATKQGIVKRSSLSNFSRINKNGK**

16 DE0525 100.0% 99.1%  **FTNKGRVYKLKGYEVPELSRQSKGIPIINAIELENDETISTMIAVKDLESEEDYLVFATKQGIVKRSSLSNFSRINKNGK**

17 4928STDY7071543 100.0% 99.0%  **FTNKGRVYKLKGYEVPELSRQSKGIPIINAIELENDETISTMIAVKDLESEEDYLVFATKQGIVKRSSLSNFSRINKNGK**

18 BAV2502 100.0% 99.0%  **FTNKGRVYKLKGYEVPELSRQSKGIPIINAIELENDETISTMIAVKDLESEEDYLVFATKQGIVKRSSLSNFSRINKNGK**

19 APC3784 100.0% 99.0%  **FTNKGRVYKLKGYEVPELSRQSKGIPIINAIELENDETISTMIAVKDLESEEDYLVFATKQGIVKRSSLSNFSRINKNGK**

20 SESURV_p1_0557 100.0% 99.9%  **FTNKGRVYKLKGYEVPELSRQSKGIPIINAIELENDETISTMIAVKDLESEEDYLVFATKQGIVKRSSLSNFSRINKNGK**

21 SESURV_p1_0612 93.3% 99.0%  **FTNKGRVYKLKGYEVPELSRQSKGIPIINAIELENDETISTMIAVKDLESEEDYLVFATKQGIVKRSSLSNFSRINKNGK**

22 SESURV_p4_1553 100.0% 99.9%  **FTNKGRVYKLKGYEVPELSRQSKGIPIINAIELENDETISTMIAVKDLESEEDYLVFATKQGIVKRSSLSNFSRINKNGK**

23 SESURV_p1_1200 100.0% 99.9%  **FTNKGRVYKLKGYEVPELSRQSKGIPIINAIELENDETISTMIAVKDLESEEDYLVFATKQGIVKRSSLSNFSRINKNGK**

24 SESURV_p3_0825 93.3% 99.8%  **FTNKGRVYKLKGYEVPELSRQSKGIPIINAIELENDETISTMIAVKDLESEEDYLVFATKQGIVKRSSLSNFSRINKNGK**

25 JH 100.0% 98.9%  **FTNKGRVYKLKGYEVPELSRQSKGIPIINAIELENDETISTMIAVKDLESEEDYLVFATKQGIVKRSSLSNFSRINKNGK**

26 Z0118SE0132 93.3% 98.9%  **FTNKGRVYKLKGYEVPELSRQSKGIPIINAIELENDETISTMIAVKDLESEEDYLVFATKQGIVKRSSLSNFSRINKNGK**

27 R5981 93.3% 98.9%  **FTNKGRVYKLKGYEVPELSRQSKGIPIINAIELENDETISTMIAVKDLESEEDYLVFATKQGIVKRSSLSNFSRINKNGK**

28 JH-S-3 100.0% 98.9%  **FTNKGRVYKLKGYEVPELSRQSKGIPIINAIELENDETISTMIAVKDLESEEDYLVFATKQGIVKRSSLSNFSRINKNGK**

29 JH-S-1 93.3% 98.8%  **FTNKGRVYKLKGYEVPELSRQSKGIPIINAIELENDETISTMIAVKDLESEEDYLVFATKQGIVKRSSLSNFSRINKNGK**

30 s2 100.0% 99.1%  **FTNKGRVYKLKGYEVPELSRQSKGIPIINAIELENDETISTMIAVKDLESEEDYLVFATKQGIVKRSSLSNFSRINKNGK**

31 S43 100.0% 99.1%  **FTNKGRVYKLKGYEVPELSRQSKGIPIINAIELENDETISTMIAVKDLESEEDYLVFATKQGIVKRSSLSNFSRINKNGK**

32 S17W 93.3% 99.0%  **FTNKGRVYKLKGYEVPELSRQSKGIPIINAIELENDETISTMIAVKDLESEEDYLVFATKQGIVKRSSLSNFSRINKNGK**

33 48 100.0% 99.0%  **FTNKGRVYKLKGYEVPELSRQSKGIPIINAIELENDETISTMIAVKDLESEEDYLVFATKQGIVKRSSLSNFSRINKNGK**

34 ABKVF 100.0% 99.0%  **FTNKGRVYKLKGYEVPELSRQSKGIPIINAIELENDETISTMIAVKDLESEEDYLVFATKQGIVKRSSLSNFSRINKNGK**

35 CICARIA 100.0% 99.9%  **FTNKGRVYKLKGYEVPELSRQSKGIPIINAIELENDETISTMIAVKDLESEEDYLVFATKQGIVKRSSLSNFSRINKNGK**

36 SKN25lux 100.0% 99.8%  **FTNKGRVYKLKGYEVPELSRQSKGIPIINAIELENDETISTMIAVKDLESEEDYLVFATKQGIVKRSSLSNFSRINKNGK**

37 HMSC074F11 100.0% 99.1%  **FTNKGRVYKLKGYEVPELSRQSKGIPIINAIELENDETISTMIAVKDLESEEDYLVFATKQGIVKRSSLSNFSRINKNGK**

38 HMSC068G11 100.0% 99.1%  **FTNKGRVYKLKGYEVPELSRQSKGIPIINAIELENDETISTMIAVKDLESEEDYLVFATKQGIVKRSSLSNFSRINKNGK**

39 NIHLM015 100.0% 99.1%  **FTNKGRVYKLKGYEVPELSRQSKGIPIINAIELENDETISTMIAVKDLESEEDYLVFATKQGIVKRSSLSNFSRINKNGK**

consensus/100%  **FTNKGRVYKLKGYEVPELSRQSKGIPIINAIELENDETISTMIAVKDLESEEDYLVFATKQGIVKRSSLSNFSRINKNGK**

consensus/90%  **FTNKGRVYKLKGYEVPELSRQSKGIPIINAIELENDETISTMIAVKDLESEEDYLVFATKQGIVKRSSLSNFSRINKNGK**

consensus/80%  **FTNKGRVYKLKGYEVPELSRQSKGIPIINAIELENDETISTMIAVKDLESEEDYLVFATKQGIVKRSSLSNFSRINKNGK**

consensus/70%  **FTNKGRVYKLKGYEVPELSRQSKGIPIINAIELENDETISTMIAVKDLESEEDYLVFATKQGIVKRSSLSNFSRINKNGK**

cov pid **641**  **: . . . . 7 . .** **720**

1 ATCC35984 100.0% 100.0%  **IAINFKEDDELIAVRLTTGNEDILIGTAHASLIRFSESTLRPLGRTAAGVKGISLREGDTVVGLDVADSESEDEVLVVTE**

2 ATCC12228 93.3% 99.9%  **IAINFKEDDELIAVRLTTGNEDILIGTAHASLIRFSESTLRPLGRTAAGVKGISLREGDTVVGLDVADSESEDEVLVVTE**

3 36AM 100.0% 99.0%  **IAINFKEDDELIAVRLTTGNEDILIGTAHASLIRFSESTLRPLGRTAAGVKGISLREGDNVVGLDVADSESEDEVLVVTE**

4 48AF 100.0% 99.9%  **IAINFKEDDELIAVRLTTGNEDILIGTAHASLIRFSESTLRPLGRTAAGVKGISLREGDTVVGLDVADSESEDEVLVVTE**

5 54AF 100.0% 99.0%  **IAINFKEDDELIAVRLTTGNEDILIGTAHASLIRFSESTLRPLGRTAAGVKGISLREGDTVVGLDVADSESEDEVLVVTE**

6 NIHLM037 100.0% 99.1%  **IAINFKEDDELIAVRLTTGNEDILIGTAHASLIRFSESTLRPLGRTAAGVKGISLREGDTVVGLDVADSESEDEVLVVTE**

7 785_SEPI 100.0% 99.0%  **IAINFKEDDELIAVRLTTGNEDILIGTAHASLIRFSESTLRPLGRTAAGVKGISLREGDTVVGLDVADSESEDEVLVVTE**

8 VSE49 100.0% 99.1%  **IAINFKEDDELIAVRLTTGNEDILIGTAHASLIRFSESTLRPLGRTAAGVKGISLREGDTVVGLDVADSESEDEVLVVTE**

9 VSE57 100.0% 99.1%  **IAINFKEDDELIAVRLTTGNEDILIGTAHASLIRFSESTLRPLGRTAAGVKGISLREGDTVVGLDVADSESEDEVLVVTE**

10 SE45 100.0% 99.1%  **IAINFKEDDELIAVRLTTGNEDILIGTAHASLIRFSESTLRPLGRTAAGVKGISLREGDTVVGLDVADSESEDEVLVVTE**

11 SE40 100.0% 99.1%  **IAINFKEDDELIAVRLTTGNEDILIGTAHASLIRFSESTLRPLGRTAAGVKGISLREGDTVVGLDVADSESEDEVLVVTE**

12 32A 100.0% 99.1%  **IAINFKEDDELIAVRLTTGNEDILIGTAHASLIRFSESTLRPLGRTAAGVKGISLREGDTVVGLDVADSESEDEVLVVTE**

13 HD66 100.0% 99.8%  **IAINFKEDDELIAVRLTTGNEDILIGTAHASLIRFSESTLRPLGRTAAGVKGISLREGDTVVGLDVADSESEDEVLVVTE**

14 HD43 100.0% 99.9%  **IAINFKEDDELIAVRLTTGNEDILIGTAHASLIRFSESTLRPLGRTAAGVKGISLREGDTVVGLDVADSESEDEVLVVTE**

15 HD33 100.0% 99.8%  **IAINFKEDDELIAVRLTTGNEDILIGTAHASLIRFSESTLRPLGRTAAGVKGISLREGDTVVGLDVADSESEDEVLVVTE**

16 DE0525 100.0% 99.1%  **IAINFKEDDELIAVRLTTGNEDILIGTAHASLIRFSESTLRPLGRTAAGVKGISLREGDTVVGLDVADSESEDEVLVVTE**

17 4928STDY7071543 100.0% 99.0%  **IAINFKEDDELIAVRLTTGNEDILIGTAHASLIRFSESTLRPLGRTAAGVKGISLREGDTVVGLDVADSESEDEVLVVTE**

18 BAV2502 100.0% 99.0%  **IAINFKEDDELIAVRLTTGNEDILIGTAHASLIRFSESTLRPLGRTAAGVKGISLREGDTVVGLDVADSESEDEVLVVTE**

19 APC3784 100.0% 99.0%  **IAINFKEDDELIAVRLTTGNEDILIGTAHASLIRFSESTLRPLGRTAAGVKGISLREGDNVVGLDVADSESEDEVLVVTE**

20 SESURV_p1_0557 100.0% 99.9%  **IAINFKEDDELIAVRLTTGNEDILIGTAHASLIRFSESTLRPLGRTAAGVKGISLREGDTVVGLDVADSESEDEVLVVTE**

21 SESURV_p1_0612 93.3% 99.0%  **IAINFKEDDELIAVRLTTGNEDILIGTAHASLIRFSESTLRPLGRTAAGVKGISLREGDTVVGLDVADSESEDEVLVVTE**

22 SESURV_p4_1553 100.0% 99.9%  **IAINFKEDDELIAVRLTTGNEDILIGTAHASLIRFSESTLRPLGRTAAGVKGISLREGDTVVGLDVADSESEDEVLVVTE**

23 SESURV_p1_1200 100.0% 99.9%  **IAINFKEDDELIAVRLTTGNEDILIGTAHASLIRFSESTLRPLGRTAAGVKGISLREGDTVVGLDVADSESEDEVLVVTE**

24 SESURV_p3_0825 93.3% 99.8%  **IAINFKEDDELIAVRLTTGNEDILIGTAHASLIRFSESTLRPLGRTAAGVKGISLREGDTVVGLDVADSESEDEVLVVTE**

25 JH 100.0% 98.9%  **IAINFKEDDELIAVRLTTGNEDILIGTAHASLIRFSESTLRPLGRTAAGVKGISLREGDTVVGLDVADSESEDEVLVVTE**

26 Z0118SE0132 93.3% 98.9%  **IAINFKEDDELIAVRLTTGNEDILIGTAHASLIRFSESTLRPLGRTAAGVKGISLREGDTVVGLDVADSESEDEVLVVTE**

27 R5981 93.3% 98.9%  **IAINFKEDDELIAVRLTTGNEDILIGTAHASLIRFSESTLRPLGRTAAGVKGISLREGDTVVGLDVADSESEDEVLVVTE**

28 JH-S-3 100.0% 98.9%  **IAINFKEDDELIAVRLTTGNEDILIGTAHASLIRFSESTLRPLGRTAAGVKGISLREGDTVVGLDVADSESEDEVLVVTE**

29 JH-S-1 93.3% 98.8%  **IAINFKEDDELIAVRLTTGNEDILIGTAHASLIRFSESTLRPLGRTAAGVKGISLREGDTVVGLDVADSESEDEVLVVTE**

30 s2 100.0% 99.1%  **IAINFKEDDELIAVRLTTGNEDILIGTAHASLIRFSESTLRPLGRTAAGVKGISLREGDTVVGLDVADSESEDEVLVVTE**

31 S43 100.0% 99.1%  **IAINFKEDDELIAVRLTTGNEDILIGTAHASLIRFSESTLRPLGRTAAGVKGISLREGDTVVGLDVADSESEDEVLVVTE**

32 S17W 93.3% 99.0%  **IAINFKEDDELIAVRLTTGNEDILIGTAHASLIRFSESTLRPLGRTAAGVKGISLREGDTVVGLDVADSESEDEVLVVTE**

33 48 100.0% 99.0%  **IAINFKEDDELIAVRLTTGNEDILIGTAHASLIRFSESTLRPLGRTAAGVKGISLREGDTVVGLDVADSESEDEVLVVTE**

34 ABKVF 100.0% 99.0%  **IAINFKEDDELIAVRLTTGNEDILIGTAHASLIRFSESTLRPLGRTAAGVKGISLREGDTVVGLDVADSESEDEVLVVTE**

35 CICARIA 100.0% 99.9%  **IAINFKEDDELIAVRLTTGNEDILIGTAHASLIRFSESTLRPLGRTAAGVKGISLREGDTVVGLDVADSESEDEVLVVTE**

36 SKN25lux 100.0% 99.8%  **IAINFKEDDELIAVRLTTGNEDILIGTAHASLIRFSESTLRPLGRTAAGVKGISLREGDTVVGLDVADSESEDEVLVVTE**

37 HMSC074F11 100.0% 99.1%  **IAINFKEDDELIAVRLTTGNEDILIGTAHASLIRFSESTLRPLGRTAAGVKGISLREGDTVVGLDVADSESEDEVLVVTE**

38 HMSC068G11 100.0% 99.1%  **IAINFKEDDELIAVRLTTGNEDILIGTAHASLIRFSESTLRPLGRTAAGVKGISLREGDTVVGLDVADSESEDEVLVVTE**

39 NIHLM015 100.0% 99.1%  **IAINFKEDDELIAVRLTTGNEDILIGTAHASLIRFSESTLRPLGRTAAGVKGISLREGDTVVGLDVADSESEDEVLVVTE**

consensus/100%  **IAINFKEDDELIAVRLTTGNEDILIGTAHASLIRFSESTLRPLGRTAAGVKGISLREGDsVVGLDVADSESEDEVLVVTE**

consensus/90%  **IAINFKEDDELIAVRLTTGNEDILIGTAHASLIRFSESTLRPLGRTAAGVKGISLREGDTVVGLDVADSESEDEVLVVTE**

consensus/80%  **IAINFKEDDELIAVRLTTGNEDILIGTAHASLIRFSESTLRPLGRTAAGVKGISLREGDTVVGLDVADSESEDEVLVVTE**

consensus/70%  **IAINFKEDDELIAVRLTTGNEDILIGTAHASLIRFSESTLRPLGRTAAGVKGISLREGDTVVGLDVADSESEDEVLVVTE**

cov pid **721**  **. . : . . . . 8** **800**

1 ATCC35984 100.0% 100.0%  **NGYGKRTPVSEYRLSNRGGKGIKTATITERNGNIVCITTVTGEEDLMVVTNAGVIIRLDVHDISQNGRAAQGVRLMKLGD**

2 ATCC12228 93.3% 99.9%  **NGYGKRTPVSEYRLSNRGGKGIKTATITERNGNIVCITTVTGEEDLMVVTNAGVIIRLDVHDISQNGRAAQGVRLMKLGD**

3 36AM 100.0% 99.0%  **NGYGKRTPVSEYRLSNRGGKGIKTATITERNGNIVCITTVTGEEDLMVVTNAGVIIRLDVHDISQNGRAAQGVRLMKLGD**

4 48AF 100.0% 99.9%  **NGYGKRTPVSEYRLSNRGGKGIKTATITERNGNIVCITTVTGEEDLMVVTNAGVIIRLDVHDISQNGRAAQGVRLMKLGD**

5 54AF 100.0% 99.0%  **NGYGKRTPVSEYRLSNRGGKGIKTATITERNGNIVCITTVTGEEDLMVVTNAGVIIRLDVHDISQNGRAAQGVRLMKLGD**

6 NIHLM037 100.0% 99.1%  **NGYGKRTPVSEYRLSNRGGKGIKTATITERNGNIVCITTVTGEEDLMVVTNAGVIIRLDVHDISQNGRAAQGVRLMKLGD**

7 785_SEPI 100.0% 99.0%  **NGYGKRTPVSEYRLSNRGGKGIKTATITERNGNIVCITTVTGEEDLMVVTNAGVIIRLDVHDISQNGRAAQGVRLMKLGD**

8 VSE49 100.0% 99.1%  **NGYGKRTPVSEYRLSNRGGKGIKTATITERNGNIVCITTVTGEEDLMVVTNAGVIIRLDVHDISQNGRAAQGVRLMKLGD**

9 VSE57 100.0% 99.1%  **NGYGKRTPVSEYRLSNRGGKGIKTATITERNGNIVCITTVTGEEDLMVVTNAGVIIRLDVHDISQNGRAAQGVRLMKLGD**

10 SE45 100.0% 99.1%  **NGYGKRTPVSEYRLSNRGGKGIKTATITERNGNIVCITTVTGEEDLMVVTNAGVIIRLDVHDISQNGRAAQGVRLMKLGD**

11 SE40 100.0% 99.1%  **NGYGKRTPVSEYRLSNRGGKGIKTATITERNGNIVCITTVTGEEDLMVVTNAGVIIRLDVHDISQNGRAAQGVRLMKLGD**

12 32A 100.0% 99.1%  **NGYGKRTPVSEYRLSNRGGKGIKTATITERNGNIVCITTVTGEEDLMVVTNAGVIIRLDVHDISQNGRAAQGVRLMKLGD**

13 HD66 100.0% 99.8%  **NGYGKRTPVSEYRLSNRGGKGIKTATITERNGNIVCITTVTGEEDLMVVTNAGVIIRLDVHDISQNGRAAQGVRLMKLGD**

14 HD43 100.0% 99.9%  **NGYGKRTPVSEYRLSNRGGKGIKTATITERNGNIVCITTVTGEEDLMVVTNAGVIIRLDVHDISQNGRAAQGVRLMKLGD**

15 HD33 100.0% 99.8%  **NGYGKRTPVSEYRLSNRGGKGIKTATITERNGNIVCITTVTGEEDLMVVTNAGVIIRLDVHDISQNGRAAQGVRLMKLGD**

16 DE0525 100.0% 99.1%  **NGYGKRTPVSEYRLSNRGGKGIKTATITERNGNIVCITTVTGEEDLMVVTNAGVIIRLDVHDISQNGRAAQGVRLMKLGD**

17 4928STDY7071543 100.0% 99.0%  **NGYGKRTPVSEYRLSNRGGKGIKTATITERNGNIVCITTVTGEEDLMVVTNAGVIIRLDVHDISQNGRAAQGVRLMKLGD**

18 BAV2502 100.0% 99.0%  **NGYGKRTPVSEYRLSNRGGKGIKTATITERNGNIVCITTVTGEEDLMVVTNAGVIIRLDVHDISQNGRAAQGVRLMKLGD**

19 APC3784 100.0% 99.0%  **NGYGKRTPVSEYRLSNRGGKGIKTATITERNGNIVCITTVTGEEDLMVVTNAGVIIRLDVHDISQNGRAAQGVRLMKLGD**

20 SESURV_p1_0557 100.0% 99.9%  **NGYGKRTPVSEYRLSNRGGKGIKTATITERNGNIVCITTVTGEEDLMVVTNAGVIIRLDVHDISQNGRAAQGVRLMKLGD**

21 SESURV_p1_0612 93.3% 99.0%  **NGYGKRTPVSEYRLSNRGGKGIKTATITERNGNIVCITTVTGEEDLMVVTNAGVIIRLDVHDISQNGRAAQGVRLMKLGD**

22 SESURV_p4_1553 100.0% 99.9%  **NGYGKRTPVSEYRLSNRGGKGIKTATITERNGNIVCITTVTGEEDLMVVTNAGVIIRLDVHDISQNGRAAQGVRLMKLGD**

23 SESURV_p1_1200 100.0% 99.9%  **NGYGKRTPVSEYRLSNRGGKGIKTATITERNGNIVCITTVTGEEDLMVVTNAGVIIRLDVHDISQNGRAAQGVRLMKLGD**

24 SESURV_p3_0825 93.3% 99.8%  **NGYGKRTPVSEYRLSNRGGKGIKTATITERNGNIVCITTVTGEEDLMVVTNAGVIIRLDVHDISQNGRAAQGVRLMKLGD**

25 JH 100.0% 98.9%  **NGYGKRTPVSEYRLSNRGGKGIKTATITERNGNIVCITTVTGEEDLMVVTNAGVIIRLDVHDISQNGRAAQGVRLMKLGD**

26 Z0118SE0132 93.3% 98.9%  **NGYGKRTPVSEYRLSNRGGKGIKTATITERNGNIVCITTVTGEEDLMVVTNAGVIIRLDVHDISQNGRAAQGVRLMKLGD**

27 R5981 93.3% 98.9%  **NGYGKRTPVSEYRLSNRGGKGIKTATITERNGNIVCITTVTGEEDLMVVTNAGVIIRLDVHDISQNGRAAQGVRLMKLGD**

28 JH-S-3 100.0% 98.9%  **NGYGKRTPVSEYRLSNRGGKGIKTATITERNGNIVCITTVTGEEDLMVVTNAGVIIRLDVHDISQNGRAAQGVRLMKLGD**

29 JH-S-1 93.3% 98.8%  **NGYGKRTPVSEYRLSNRGGKGIKTATITERNGNIVCITTVTGEEDLMVVTNAGVIIRLDVHDISQNGRAAQGVRLMKLGD**

30 s2 100.0% 99.1%  **NGYGKRTPVSEYRLSNRGGKGIKTATITERNGNIVCITTVTGEEDLMVVTNAGVIIRLDVHDISQNGRAAQGVRLMKLGD**

31 S43 100.0% 99.1%  **NGYGKRTPVSEYRLSNRGGKGIKTATITERNGNIVCITTVTGEEDLMVVTNAGVIIRLDVHDISQNGRAAQGVRLMKLGD**

32 S17W 93.3% 99.0%  **NGYGKRTPVSEYRLSNRGGKGIKTATITERNGNIVCITTVTGEEDLMVVTNAGVIIRLDVHDISQNGRAAQGVRLMKLGD**

33 48 100.0% 99.0%  **NGYGKRTPVSEYRLSNRGGKGIKTATITERNGNIVCITTVTGEEDLMVVTNAGVIIRLDVHDISQNGRAAQGVRLMKLGD**

34 ABKVF 100.0% 99.0%  **NGYGKRTPVSEYRLSNRGGKGIKTATITERNGNIVCITTVTGEEDLMVVTNAGVIIRLDVHDISQNGRAAQGVRLMKLGD**

35 CICARIA 100.0% 99.9%  **NGYGKRTPVSEYRLSNRGGKGIKTATITERNGNIVCITTVTGEEDLMVVTNAGVIIRLDVHDISQNGRAAQGVRLMKLGD**

36 SKN25lux 100.0% 99.8%  **NGYGKRTPVSEYRLSNRGGKGIKTATITERNGNIVCITTVTGEEDLMVVTNAGVIIRLDVHDISQNGRAAQGVRLMKLGD**

37 HMSC074F11 100.0% 99.1%  **NGYGKRTPVSEYRLSNRGGKGIKTATITERNGNIVCITTVTGEEDLMVVTNAGVIIRLDVHDISQNGRAAQGVRLMKLGD**

38 HMSC068G11 100.0% 99.1%  **NGYGKRTPVSEYRLSNRGGKGIKTATITERNGNIVCITTVTGEEDLMVVTNAGVIIRLDVHDISQNGRAAQGVRLMKLGD**

39 NIHLM015 100.0% 99.1%  **NGYGKRTPVSEYRLSNRGGKGIKTATITERNGNIVCITTVTGEEDLMVVTNAGVIIRLDVHDISQNGRAAQGVRLMKLGD**

consensus/100%  **NGYGKRTPVSEYRLSNRGGKGIKTATITERNGNIVCITTVTGEEDLMVVTNAGVIIRLDVHDISQNGRAAQGVRLMKLGD**

consensus/90%  **NGYGKRTPVSEYRLSNRGGKGIKTATITERNGNIVCITTVTGEEDLMVVTNAGVIIRLDVHDISQNGRAAQGVRLMKLGD**

consensus/80%  **NGYGKRTPVSEYRLSNRGGKGIKTATITERNGNIVCITTVTGEEDLMVVTNAGVIIRLDVHDISQNGRAAQGVRLMKLGD**

consensus/70%  **NGYGKRTPVSEYRLSNRGGKGIKTATITERNGNIVCITTVTGEEDLMVVTNAGVIIRLDVHDISQNGRAAQGVRLMKLGD**

cov pid **801**  **. . . . : . . .** **880**

1 ATCC35984 100.0% 100.0%  **GQFVSTVAKVNEEDDNEENADEAQQSTTTETADVEEVVDDQTPGNAIHTEGDAEMESVESPENDDRIDIRQDFMDRVNED**

2 ATCC12228 93.3% 99.9%  **GQFVSTVAKVNEEDDNEENADEAQQSTTTETADVEEVVDDQTPGNAIHTEGDAEMESVESPENDDRIDIRQDFMDRVNED**

3 36AM 100.0% 99.0%  **GQFVSTVAKVKEEDDNEENVDEAQQTTNAEKADVEEVVDDQTPGNAIHTEGDAETESVESPENDDRIDIRQDFMDRVNED**

4 48AF 100.0% 99.9%  **GQFVSTVAKVNEEDDNEENADEAQQSTTTETADVEEVVDDQTPGNAIHTEGDAEMESVESPENDDRIDIRQDFMDRVNED**

5 54AF 100.0% 99.0%  **GQFVSTVAKVKEEDDNEENVDEAQQTTSAEKADVEEVVDDQTPGNAIHTEGDAETESVESPKNDDRIDIRQDFMDRVNED**

6 NIHLM037 100.0% 99.1%  **GQFVSTVAKVKEEDDNEENVDEAQQTTSAEKADVEEVVDDQTPGNAIHTEGDAETESVESPENDDRIDIRQDFMDRVNED**

7 785_SEPI 100.0% 99.0%  **GQFVSTVAKVKEEDDNEENVDEAQQTTSAEKADVEEVVDDQTPGNAIHTEGDAETESVESPKNDDRIDIRQDFMDRVNED**

8 VSE49 100.0% 99.1%  **GQFVSTVAKVKEEDDNEENVDEAQQTTSAEKADVEEVVDDQTPGNAIHTEGDAEMESVESPKNDDRIDIRQDFMDRVNED**

9 VSE57 100.0% 99.1%  **GQFVSTVAKVKEEDDNEENVDEAQQTTSAEKADVEEVVDDQTPGNAIHTEGDAEMESVESPKNDDRIDIRQDFMDRVNED**

10 SE45 100.0% 99.1%  **GQFVSTVAKVKEEDDNEENVDEAQQTTSAEKADVEEVVDDQTPGNAIHTEGDAEMESVESPKNDDRIDIRQDFMDRVNED**

11 SE40 100.0% 99.1%  **GQFVSTVAKVKEEDDNEENVDEAQQTTSAEKADVEEVVDDQTPGNAIHTEGDAEMESVESPKNDDRIDIRQDFMDRVNED**

12 32A 100.0% 99.1%  **GQFVSTVAKVKEEDDNEENVDEAQQTTSAEKADVEEVVDDQTPGNAIHTEGDAETESVESPENDDRIDIRQDFMDRVNED**

13 HD66 100.0% 99.8%  **GQFVSTVAKVNEEDDNEENADEAQQSTTTETADVEEVVDDQTPGNAIHTEGDAEMESVESPENDDRIDIRQDFMDRVNED**

14 HD43 100.0% 99.9%  **GQFVSTVAKVNEEDDNEENADEAQQSTTTETADVEEVVDDQTPGNAIHTEGDAEMESVESPENDDRIDIRQDFMDRVNED**

15 HD33 100.0% 99.8%  **GQFVSTVAKVNEEDDNEENADEAQQSTTTETADVEEVVDDQTPGNAIHTEGDAEMESVESPENDDRIDIRQDFMDRVNED**

16 DE0525 100.0% 99.1%  **GQFVSTVAKVKEEDDNEENVDEAQQTTSAEKADVEEVVDDQTPGNAIHTEGDAETESVESPENDDRIDIRQDFMDRVNED**

17 4928STDY7071543 100.0% 99.0%  **GQFVSTVAKVKEEDDNEENVDEAQQTTSAEKADVEEVVDDQTPGNAIHTEGDAETESVESPENDDRIDIRQDFMDRVNED**

18 BAV2502 100.0% 99.0%  **GQFVSTVAKVKEEDDNEENVDEAQQTTSAEKADVEEVVDDQTPGNAIHTEGDAETESVESPKNDDRIDIRQDFMDRVNED**

19 APC3784 100.0% 99.0%  **GQFVSTVAKVKEEDDNEENVDEAQQTTNAEKADVEEVVDDQTPGNAIHTEGDAETESVESPENDDRIDIRQDFMDRVNED**

20 SESURV_p1_0557 100.0% 99.9%  **GQFVSTVAKVNEEDDNEENADEAQQSTTTETADVEEVVDDQTPGNAIHTEGDAEMESVESPENDDRIDIRQDFMDRVNED**

21 SESURV_p1_0612 93.3% 99.0%  **GQFVSTVAKVKEEDDNEENVDEAQQTTTAEKADVEELVDDQTPGNAIHTEGDAETESVESPENDDRIDIRQDFMDRVNED**

22 SESURV_p4_1553 100.0% 99.9%  **GQFVSTVAKVNEEDDNEENADEAQQSTTTETADVEEVVDDQTPGNAIHTEGDAEMESVESPENDDRIDIRQDFMDRVNED**

23 SESURV_p1_1200 100.0% 99.9%  **GQFVSTVAKVNEEDDNEENADEAQQSTTTETADVEEVVDDQTPGNAIHTEGDAEMESVESPENDDRIDIRQDFMDRVNED**

24 SESURV_p3_0825 93.3% 99.8%  **GQFVSTVAKVNEEDDNEENADEAQQSTTTETADVEEVVDDQTPGNAIHTEGDAEMESVESPENDDRIDIRQDFMDRVNED**

25 JH 100.0% 98.9%  **GQFVSTVAKVKEEDDNEENVDEAQQTTSAEKADVEEVVDDQTPGNAIHTEGDAETESVESLENDDRIDIRQDFMDRVNED**

26 Z0118SE0132 93.3% 98.9%  **GQFVSTVAKVKEEDDDEKNVDEAQQTTTAEKADVEEVVDDQTPGNAIHTEGDAEMESVESPENDDRIDIRQDFMDRVNED**

27 R5981 93.3% 98.9%  **GQFVSTVAKVKEEDDNEENVDEAQQTTSAEKADVEEVVDDQTPGNAIHTEGDAETESVESPENDDRIDIRQDFMDRVNED**

28 JH-S-3 100.0% 98.9%  **GQFVSTVAKVKEEDDNEENVDEAQQTTSAEKADVEEVVDDQTPGNAIHTEGDAETESVESLENDDRIDIRQDFMDRVNED**

29 JH-S-1 93.3% 98.8%  **GQFVSTVAKVKEEDDNEENVDEAQQTTSAEKADVEEVVDDQTPGNAIHTEGDAETESVESLENDDRIDIRQDFMDRVNED**

30 s2 100.0% 99.1%  **GQFVSTVAKVKEEDDNEENVDEAQQTTSAEKADVEEVVDDQTPGNAIHTEGDAETESVESPENDDRIDIRQDFMDRVNED**

31 S43 100.0% 99.1%  **GQFVSTVAKVKEEDDDEKNVDEAQQTTTAEKADVEEVVDDQTPGNAIHTEGDAEMESVESPENDDRIDIRQDFMDRVNED**

32 S17W 93.3% 99.0%  **GQFVSTVAKVKEEDDNEENVDEAQQTTSAEKADVEEVVDDQTPGNAIHTEGDAETESVESPENDDRIDIRQDFMDRVNED**

33 48 100.0% 99.0%  **GQFVSTVAKVKEEDDNEENVDEAQQTTSAEKADVEEVVDDQTPGNAIHTEGDAETESVESPKNDDRIDIRQDFMDRVNED**

34 ABKVF 100.0% 99.0%  **GQFVSTVAKVKEEDDNEENVDEAQQTTSAEKADVEEVVDDQTPGNAIHTEGDAETESVESPENDDRIDIRQDFMDRVNED**

35 CICARIA 100.0% 99.9%  **GQFVSTVAKVNEEDDNEENADEAQQSTTTETADVEEVVDDQTPGNAIHTEGDAEMESVESPENDDRIDIRQDFMDRVNED**

36 SKN25lux 100.0% 99.8%  **GQFVSTVAKVNEEDDNEENADEAQQSTTTETADVEEVVDDQTPGNAIHTEGDAEMESVESPENDDRIDIRQDFMDRVNED**

37 HMSC074F11 100.0% 99.1%  **GQFVSTVAKVKEEDDNEENVDEAQQTTSAEKADVEEVVDDQTPGNAIHTEGDAETESVESPENDDRIDIRQDFMDRVNED**

38 HMSC068G11 100.0% 99.1%  **GQFVSTVAKVKEEDDNEENVDEAQQTTSAEKADVEEVVDDQTPGNAIHTEGDAETESVESPENDDRIDIRQDFMDRVNED**

39 NIHLM015 100.0% 99.1%  **GQFVSTVAKVKEEDDNEENVDEAQQTTSAEKADVEEVVDDQTPGNAIHTEGDAETESVESPENDDRIDIRQDFMDRVNED**

consensus/100%  **GQFVSTVAKVpEEDDsEcNsDEAQQoTssEpADVEElVDDQTPGNAIHTEGDAEhESVES.cNDDRIDIRQDFMDRVNED**

consensus/90%  **GQFVSTVAKVpEEDDNEENsDEAQQoTosEpADVEEVVDDQTPGNAIHTEGDAEhESVESPcNDDRIDIRQDFMDRVNED**

consensus/80%  **GQFVSTVAKVpEEDDNEENsDEAQQoTosEpADVEEVVDDQTPGNAIHTEGDAEhESVESPcNDDRIDIRQDFMDRVNED**

consensus/70%  **GQFVSTVAKVpEEDDNEENsDEAQQoTosEpADVEEVVDDQTPGNAIHTEGDAEhESVESPENDDRIDIRQDFMDRVNED**

cov pid **881**  **. ]** **893**

1 ATCC35984 100.0% 100.0%  **IESASDNEEDSDE**

2 ATCC12228 93.3% 99.9%  **IESASDNEEDSDE**

3 36AM 100.0% 99.0%  **IESASDNEEDSDE**

4 48AF 100.0% 99.9%  **IESASDNEEDSDE**

5 54AF 100.0% 99.0%  **IESASDNEEDSDE**

6 NIHLM037 100.0% 99.1%  **IESASDNEEDSDE**

7 785_SEPI 100.0% 99.0%  **IESASDNEEDSDE**

8 VSE49 100.0% 99.1%  **IESASDNEEDSDE**

9 VSE57 100.0% 99.1%  **IESASDNEEDSDE**

10 SE45 100.0% 99.1%  **IESASDNEEDSDE**

11 SE40 100.0% 99.1%  **IESASDNEEDSDE**

12 32A 100.0% 99.1%  **IESASDNEEDSDE**

13 HD66 100.0% 99.8%  **IESASDNEEDSDE**

14 HD43 100.0% 99.9%  **IESASDNEEDSDE**

15 HD33 100.0% 99.8%  **IESASDNEEDSDE**

16 DE0525 100.0% 99.1%  **IESASDNEEDSDE**

17 4928STDY7071543 100.0% 99.0%  **IESASDNEEDSDE**

18 BAV2502 100.0% 99.0%  **IESASDNEEDSDE**

19 APC3784 100.0% 99.0%  **IESASDNEEDSDE**

20 SESURV_p1_0557 100.0% 99.9%  **IESASDNEEDSDE**

21 SESURV_p1_0612 93.3% 99.0%  **IESASDNEEDSDE**

22 SESURV_p4_1553 100.0% 99.9%  **IESASDNEEDSDE**

23 SESURV_p1_1200 100.0% 99.9%  **IESASDNEEDSDE**

24 SESURV_p3_0825 93.3% 99.8%  **IESASDNEEDSDE**

25 JH 100.0% 98.9%  **IESASDNEEDSDE**

26 Z0118SE0132 93.3% 98.9%  **IESASDNEEDSDE**

27 R5981 93.3% 98.9%  **IESASDNEEDSDE**

28 JH-S-3 100.0% 98.9%  **IESASDNEEDSDE**

29 JH-S-1 93.3% 98.8%  **IESASDNEEDSDE**

30 s2 100.0% 99.1%  **IESASDNEEDSDE**

31 S43 100.0% 99.1%  **IESASDNEEDSDE**

32 S17W 93.3% 99.0%  **IESASDNEEDSDE**

33 48 100.0% 99.0%  **IESASDNEEDSDE**

34 ABKVF 100.0% 99.0%  **IESASDNEEDSDE**

35 CICARIA 100.0% 99.9%  **IESASDNEEDSDE**

36 SKN25lux 100.0% 99.8%  **IESASDNEEDSDE**

37 HMSC074F11 100.0% 99.1%  **IESASDNEEDSDE**

38 HMSC068G11 100.0% 99.1%  **IESASDNEEDSDE**

39 NIHLM015 100.0% 99.1%  **IESASDNEEDSDE**

consensus/100%  **IESASDNEEDSDE**

consensus/90%  **IESASDNEEDSDE**

consensus/80%  **IESASDNEEDSDE**

consensus/70%  **IESASDNEEDSDE**

**Supplementary Figure 1.** Multiple sequence alignment of the predicted amino acid sequences of GyrA carried by *S. epidermidis* isolates and close genomes retrieved from the BV-BRC database compared to that of *S. epidermidis* strain ATCC®12228 and ATCC®35984. The alignment was visualized using MView version 1.63 hosted by the EMBL-EBI; cov, coverage; pid, percent identity.

cov pid  **1** **[ . . . . : . . .** **80**

1 ATCC35984 100.0% 100.0%  **MVNTLSDVNNTDNYGAGQIQVLEGLEAVRKRPGMYIGSTSERGLHHLVWEIVDNSIDEALAGYASHIEVVIEKDNWIKVT**

2 ATCC12228 90.7% 100.0%  **------------------------------------------------------------AGYASHIEVVIEKDNWIKVT**

3 36AM 90.7% 99.7%  **------------------------------------------------------------AGYASHIEVIIEKDNWIKVT**

4 48AF 100.0% 100.0%  **MVNTLSDVNNTDNYGAGQIQVLEGLEAVRKRPGMYIGSTSERGLHHLVWEIVDNSIDEALAGYASHIEVVIEKDNWIKVT**

5 54AF 100.0% 99.7%  **MVNTLSDVNNTDNYGAGQIQVLEGLEAVRKRPGMYIGSTSERGLHHLVWEIVDNSIDEALAGYASHIEVIIEKDNWIKVT**

6 785_SEPI 100.0% 99.7%  **MVNTLSDVNNTDNYGAGQIQVLEGLEAVRKRPGMYIGSTSERGLHHLVWEIVDNSIDEALAGYASHIEVIIEKDNWIKVT**

7 VSE49 100.0% 99.7%  **MVNTLSDVNNTDNYGAGQIQVLEGLEAVRKRPGMYIGSTSERGLHHLVWEIVDNSIDEALAGYASHIEVIIEKDNWIKVT**

8 VSE57 100.0% 99.7%  **MVNTLSDVNNTDNYGAGQIQVLEGLEAVRKRPGMYIGSTSERGLHHLVWEIVDNSIDEALAGYASHIEVIIEKDNWIKVT**

9 SE45 100.0% 99.7%  **MVNTLSDVNNTDNYGAGQIQVLEGLEAVRKRPGMYIGSTSERGLHHLVWEIVDNSIDEALAGYASHIEVIIEKDNWIKVT**

10 SE40 100.0% 99.7%  **MVNTLSDVNNTDNYGAGQIQVLEGLEAVRKRPGMYIGSTSERGLHHLVWEIVDNSIDEALAGYASHIEVIIEKDNWIKVT**

11 32A 100.0% 99.7%  **MVNTLSDVNNTDNYGAGQIQVLEGLEAVRKRPGMYIGSTSERGLHHLVWEIVDNSIDEALAGYASHIEVIIEKDNWIKVT**

12 HD66 100.0% 100.0%  **MVNTLSDVNNTDNYGAGQIQVLEGLEAVRKRPGMYIGSTSERGLHHLVWEIVDNSIDEALAGYASHIEVVIEKDNWIKVT**

13 HD43 100.0% 100.0%  **MVNTLSDVNNTDNYGAGQIQVLEGLEAVRKRPGMYIGSTSERGLHHLVWEIVDNSIDEALAGYASHIEVVIEKDNWIKVT**

14 HD33 100.0% 100.0%  **MVNTLSDVNNTDNYGAGQIQVLEGLEAVRKRPGMYIGSTSERGLHHLVWEIVDNSIDEALAGYASHIEVVIEKDNWIKVT**

15 DE0525 100.0% 99.7%  **MVNTLSDVNNTDNYGAGQIQVLEGLEAVRKRPGMYIGSTSERGLHHLVWEIVDNSIDEALAGYASHIEVIIEKDNWIKVT**

16 4928STDY7071543 100.0% 99.7%  **MVNTLSDVNNTDNYGAGQIQVLEGLEAVRKRPGMYIGSISERGLHHLVWEIVDNSIDEALAGYASHIEVIIEKDNWIKVT**

17 BAV 100.0% 99.7%  **MVNTLSDVNNTDNYGAGQIQVLEGLEAVRKRPGMYIGSTSERGLHHLVWEIVDNSIDEALAGYASHIEVIIEKDNWIKVT**

18 APC 100.0% 99.7%  **MVNTLSDVNNTDNYGAGQIQVLEGLEAVRKRPGMYIGSTSERGLHHLVWEIVDNSIDEALAGYASHIEVIIEKDNWIKVT**

19 SESURV_p1_0557 100.0% 100.0%  **MVNTLSDVNNTDNYGAGQIQVLEGLEAVRKRPGMYIGSTSERGLHHLVWEIVDNSIDEALAGYASHIEVVIEKDNWIKVT**

20 SESURV_p1_0612 100.0% 99.8%  **MVNTLSDVNNTDNYGAGQIQVLEGLEAVRKRPGMYIGSTSERGLHHLVWEIVDNSIDEALAGYASHIEVIIEKDNWIKVT**

21 SESURV_p4_1553 100.0% 99.7%  **MVNTLSDVNNTDNYGAGQIQVLEGLEAVRKRPGMYIGSTSERGLHHLVWEIVDNSIDEALAGYASHIEVIIEKDNWIKVT**

22 SESURV_p1_1200 100.0% 100.0%  **MVNTLSDVNNTDNYGAGQIQVLEGLEAVRKRPGMYIGSTSERGLHHLVWEIVDNSIDEALAGYASHIEVVIEKDNWIKVT**

23 SESURV_p3_0825 90.7% 100.0%  **------------------------------------------------------------AGYASHIEVVIEKDNWIKVT**

24 JH 100.0% 99.8%  **MVNTLSDVNNTDNYGAGQIQVLEGLEAVRKRPGMYIGSTSERGLHHLVWEIVDNSIDEALAGYASHIEVIIEKDNWIKVT**

25 Z0118SE0132 100.0% 99.8%  **MVNTLSDVNNTDNYGAGQIQVLEGLEAVRKRPGMYIGSTSERGLHHLVWEIVDNSIDEALAGYASHIEVIIEKDNWIKVT**

26 R5981 100.0% 99.8%  **MVNTLSDVNNTDNYGAGQIQVLEGLEAVRKRPGMYIGSTSERGLHHLVWEIVDNSIDEALAGYASHIEVIIEKDNWIKVT**

27 JH-S-3 100.0% 99.8%  **MVNTLSDVNNTDNYGAGQIQVLEGLEAVRKRPGMYIGSTSERGLHHLVWEIVDNSIDEALAGYASHIEVIIEKDNWIKVT**

28 JH-S-1 100.0% 99.8%  **MVNTLSDVNNTDNYGAGQIQVLEGLEAVRKRPGMYIGSTSERGLHHLVWEIVDNSIDEALAGYASHIEVIIEKDNWIKVT**

29 s2 100.0% 99.7%  **MVNTLSDVNNTDNYGAGQIQVLEGLEAVRKRPGMYIGSTSERGLHHLVWEIVDNSIDEALAGYASHIEVIIEKDNWIKVT**

30 S43 100.0% 99.8%  **MVNTLSDVNNTDNYGAGQIQVLEGLEAVRKRPGMYIGSTSERGLHHLVWEIVDNSIDEALAGYASHIEVIIEKDNWIKVT**

31 S17W 90.7% 99.7%  **------------------------------------------------------------AGYASHIEVIIEKDNWIKVT**

32 48 100.0% 99.7%  **MVNTLSDVNNTDNYGAGQIQVLEGLEAVRKRPGMYIGSTSERGLHHLVWEIVDNSIDEALAGYASHIEVIIEKDNWIKVT**

33 ABKVF 100.0% 99.7%  **MVNTLSDVNNTDNYGAGQIQVLEGLEAVRKRPGMYIGSTSERGLHHLVWEIVDNSIDEALAGYASHIEVIIEKDNWIKVT**

34 CICARIA 100.0% 100.0%  **MVNTLSDVNNTDNYGAGQIQVLEGLEAVRKRPGMYIGSTSERGLHHLVWEIVDNSIDEALAGYASHIEVVIEKDNWIKVT**

35 SKN25lux 90.7% 100.0%  **------------------------------------------------------------AGYASHIEVVIEKDNWIKVT**

36 HMSC074F11 90.7% 99.7%  **------------------------------------------------------------AGYASHIEVIIEKDNWIKVT**

37 HMSC068G11 100.0% 99.7%  **MVNTLSDVNNTDNYGAGQIQVLEGLEAVRKRPGMYIGSTSERGLHHLVWEIVDNSIDEALAGYASHIEVIIEKDNWIKVT**

38 NIHLM037 90.7% 99.7%  **------------------------------------------------------------AGYASHIEVIIEKDNWIKVT**

39 NIHLM015 90.7% 99.7%  **------------------------------------------------------------AGYASHIEVIIEKDNWIKVT**

consensus/100%  **............................................................AGYASHIEVlIEKDNWIKVT**

consensus/90%  **............................................................AGYASHIEVlIEKDNWIKVT**

consensus/80%  **............................................................AGYASHIEVlIEKDNWIKVT**

consensus/70%  **MVNTLSDVNNTDNYGAGQIQVLEGLEAVRKRPGMYIGSTSERGLHHLVWEIVDNSIDEALAGYASHIEVIIEKDNWIKVT**

cov pid  **81**  **. 1 . . . . : .** **160**

1 ATCC35984 100.0% 100.0%  **DNGRGIPVDIQEKMGRPAVEVILTVLHAGGKFGGGGYKVSGGLHGVGSSVVNALSQDLEVYVHRNGTIYHQAYKQGVPQF**

2 ATCC12228 90.7% 100.0%  **DNGRGIPVDIQEKMGRPAVEVILTVLHAGGKFGGGGYKVSGGLHGVGSSVVNALSQDLEVYVHRNGTIYHQAYKQGVPQF**

3 36AM 90.7% 99.7%  **DNGRGIPVDIQEKMGRPAVEVILTVLHAGGKFGGGGYKVSGGLHGVGSSVVNALSQDLEVYVHRNGTIYHQAYKQGVPQF**

4 48AF 100.0% 100.0%  **DNGRGIPVDIQEKMGRPAVEVILTVLHAGGKFGGGGYKVSGGLHGVGSSVVNALSQDLEVYVHRNGTIYHQAYKQGVPQF**

5 54AF 100.0% 99.7%  **DNGRGIPVDIQEKMGRPAVEVILTVLHAGGKFGGGGYKVSGGLHGVGSSVVNALSQDLEVYVHRNGTIYHQAYKQGVPQF**

6 785_SEPI 100.0% 99.7%  **DNGRGIPVDIQEKMGRPAVEVILTVLHAGGKFGGGGYKVSGGLHGVGSSVVNALSQDLEVYVHRNGTIYHQAYKQGVPQF**

7 VSE49 100.0% 99.7%  **DNGRGIPVDIQEKMGRPAVEVILTVLHAGGKFGGGGYKVSGGLHGVGSSVVNALSQDLEVYVHRNGTIYHQAYKQGVPQF**

8 VSE57 100.0% 99.7%  **DNGRGIPVDIQEKMGRPAVEVILTVLHAGGKFGGGGYKVSGGLHGVGSSVVNALSQDLEVYVHRNGTIYHQAYKQGVPQF**

9 SE45 100.0% 99.7%  **DNGRGIPVDIQEKMGRPAVEVILTVLHAGGKFGGGGYKVSGGLHGVGSSVVNALSQDLEVYVHRNGTIYHQAYKQGVPQF**

10 SE40 100.0% 99.7%  **DNGRGIPVDIQEKMGRPAVEVILTVLHAGGKFGGGGYKVSGGLHGVGSSVVNALSQDLEVYVHRNGTIYHQAYKQGVPQF**

11 32A 100.0% 99.7%  **DNGRGIPVDIQEKMGRPAVEVILTVLHAGGKFGGGGYKVSGGLHGVGSSVVNALSQDLEVYVHRNGTIYHQAYKQGVPQF**

12 HD66 100.0% 100.0%  **DNGRGIPVDIQEKMGRPAVEVILTVLHAGGKFGGGGYKVSGGLHGVGSSVVNALSQDLEVYVHRNGTIYHQAYKQGVPQF**

13 HD43 100.0% 100.0%  **DNGRGIPVDIQEKMGRPAVEVILTVLHAGGKFGGGGYKVSGGLHGVGSSVVNALSQDLEVYVHRNGTIYHQAYKQGVPQF**

14 HD33 100.0% 100.0%  **DNGRGIPVDIQEKMGRPAVEVILTVLHAGGKFGGGGYKVSGGLHGVGSSVVNALSQDLEVYVHRNGTIYHQAYKQGVPQF**

15 DE0525 100.0% 99.7%  **DNGRGIPVDIQEKMGRPAVEVILTVLHAGGKFGGGGYKVSGGLHGVGSSVVNALSQDLEVYVHRNGTIYHQAYKQGVPQF**

16 4928STDY7071543 100.0% 99.7%  **DNGRGIPVDIQEKMGRPAVEVILTVLHAGGKFGGGGYKVSGGLHGVGSSVVNALSQDLEVYVHRNGTIYHQAYKQGVPQF**

17 BAV 100.0% 99.7%  **DNGRGIPVDIQEKMGRPAVEVILTVLHAGGKFGGGGYKVSGGLHGVGSSVVNALSQDLEVYVHRNGTIYHQAYKQGVPQF**

18 APC 100.0% 99.7%  **DNGRGIPVDIQEKMGRPAVEVILTVLHAGGKFGGGGYKVSGGLHGVGSSVVNALSQDLEVYVHRNGTIYHQAYKQGVPQF**

19 SESURV_p1_0557 100.0% 100.0%  **DNGRGIPVDIQEKMGRPAVEVILTVLHAGGKFGGGGYKVSGGLHGVGSSVVNALSQDLEVYVHRNGTIYHQAYKQGVPQF**

20 SESURV_p1_0612 100.0% 99.8%  **DNGRGIPVDIQEKMGRPAVEVILTVLHAGGKFGGGGYKVSGGLHGVGSSVVNALSQDLEVYVHRNGTIYHQAYKQGVPQF**

21 SESURV_p4_1553 100.0% 99.7%  **DNGRGIPVDIQEKMGRPAVEVILTVLHAGGKFGGGGYKVSGGLHGVGSSVVNALSQDLEVYVHRNGTIYHQAYKQGVPQF**

22 SESURV_p1_1200 100.0% 100.0%  **DNGRGIPVDIQEKMGRPAVEVILTVLHAGGKFGGGGYKVSGGLHGVGSSVVNALSQDLEVYVHRNGTIYHQAYKQGVPQF**

23 SESURV_p3_0825 90.7% 100.0%  **DNGRGIPVDIQEKMGRPAVEVILTVLHAGGKFGGGGYKVSGGLHGVGSSVVNALSQDLEVYVHRNGTIYHQAYKQGVPQF**

24 JH 100.0% 99.8%  **DNGRGIPVDIQEKMGRPAVEVILTVLHAGGKFGGGGYKVSGGLHGVGSSVVNALSQDLEVYVHRNGTIYHQAYKQGVPQF**

25 Z0118SE0132 100.0% 99.8%  **DNGRGIPVDIQEKMGRPAVEVILTVLHAGGKFGGGGYKVSGGLHGVGSSVVNALSQDLEVYVHRNGTIYHQAYKQGVPQF**

26 R5981 100.0% 99.8%  **DNGRGIPVDIQEKMGRPAVEVILTVLHAGGKFGGGGYKVSGGLHGVGSSVVNALSQDLEVYVHRNGTIYHQAYKQGVPQF**

27 JH-S-3 100.0% 99.8%  **DNGRGIPVDIQEKMGRPAVEVILTVLHAGGKFGGGGYKVSGGLHGVGSSVVNALSQDLEVYVHRNGTIYHQAYKQGVPQF**

28 JH-S-1 100.0% 99.8%  **DNGRGIPVDIQEKMGRPAVEVILTVLHAGGKFGGGGYKVSGGLHGVGSSVVNALSQDLEVYVHRNGTIYHQAYKQGVPQF**

29 s2 100.0% 99.7%  **DNGRGIPVDIQEKMGRPAVEVILTVLHAGGKFGGGGYKVSGGLHGVGSSVVNALSQDLEVYVHRNGTIYHQAYKQGVPQF**

30 S43 100.0% 99.8%  **DNGRGIPVDIQEKMGRPAVEVILTVLHAGGKFGGGGYKVSGGLHGVGSSVVNALSQDLEVYVHRNGTIYHQAYKQGVPQF**

31 S17W 90.7% 99.7%  **DNGRGIPVDIQEKMGRPAVEVILTVLHAGGKFGGGGYKVSGGLHGVGSSVVNALSQDLEVYVHRNGTIYHQAYKQGVPQF**

32 48 100.0% 99.7%  **DNGRGIPVDIQEKMGRPAVEVILTVLHAGGKFGGGGYKVSGGLHGVGSSVVNALSQDLEVYVHRNGTIYHQAYKQGVPQF**

33 ABKVF 100.0% 99.7%  **DNGRGIPVDIQEKMGRPAVEVILTVLHAGGKFGGGGYKVSGGLHGVGSSVVNALSQDLEVYVHRNGTIYHQAYKQGVPQF**

34 CICARIA 100.0% 100.0%  **DNGRGIPVDIQEKMGRPAVEVILTVLHAGGKFGGGGYKVSGGLHGVGSSVVNALSQDLEVYVHRNGTIYHQAYKQGVPQF**

35 SKN25lux 90.7% 100.0%  **DNGRGIPVDIQEKMGRPAVEVILTVLHAGGKFGGGGYKVSGGLHGVGSSVVNALSQDLEVYVHRNGTIYHQAYKQGVPQF**

36 HMSC074F11 90.7% 99.7%  **DNGRGIPVDIQEKMGRPAVEVILTVLHAGGKFGGGGYKVSGGLHGVGSSVVNALSQDLEVYVHRNGTIYHQAYKQGVPQF**

37 HMSC068G11 100.0% 99.7%  **DNGRGIPVDIQEKMGRPAVEVILTVLHAGGKFGGGGYKVSGGLHGVGSSVVNALSQDLEVYVHRNGTIYHQAYKQGVPQF**

38 NIHLM037 90.7% 99.7%  **DNGRGIPVDIQEKMGRPAVEVILTVLHAGGKFGGGGYKVSGGLHGVGSSVVNALSQDLEVYVHRNGTIYHQAYKQGVPQF**

39 NIHLM015 90.7% 99.7%  **DNGRGIPVDIQEKMGRPAVEVILTVLHAGGKFGGGGYKVSGGLHGVGSSVVNALSQDLEVYVHRNGTIYHQAYKQGVPQF**

consensus/100%  **DNGRGIPVDIQEKMGRPAVEVILTVLHAGGKFGGGGYKVSGGLHGVGSSVVNALSQDLEVYVHRNGTIYHQAYKQGVPQF**

consensus/90%  **DNGRGIPVDIQEKMGRPAVEVILTVLHAGGKFGGGGYKVSGGLHGVGSSVVNALSQDLEVYVHRNGTIYHQAYKQGVPQF**

consensus/80%  **DNGRGIPVDIQEKMGRPAVEVILTVLHAGGKFGGGGYKVSGGLHGVGSSVVNALSQDLEVYVHRNGTIYHQAYKQGVPQF**

consensus/70%  **DNGRGIPVDIQEKMGRPAVEVILTVLHAGGKFGGGGYKVSGGLHGVGSSVVNALSQDLEVYVHRNGTIYHQAYKQGVPQF**

cov pid **161**  **. . . 2 . . . .** **240**

1 ATCC35984 100.0% 100.0%  **DLKEIGDTDKTGTAIRFKADKEIFTETTVYNYETLQKRIRELAFLNKGIQITLKDEREEEVREDSYHYEGGIKSYVDLLN**

2 ATCC12228 90.7% 100.0%  **DLKEIGDTDKTGTAIRFKADKEIFTETTVYNYETLQKRIRELAFLNKGIQITLKDEREEEVREDSYHYEGGIKSYVDLLN**

3 36AM 90.7% 99.7%  **DLKEIGDTDKTGTAIRFKADKEIFTETTVYNYETLQKRIRELAFLNKGIQITLKDEREEEIREDSYHYEGGIKSYVDLLN**

4 48AF 100.0% 100.0%  **DLKEIGDTDKTGTAIRFKADKEIFTETTVYNYETLQKRIRELAFLNKGIQITLKDEREEEVREDSYHYEGGIKSYVDLLN**

5 54AF 100.0% 99.7%  **DLKEIGDTDKTGTVIRFKADKEIFTETTVYNYETLQKRIRELAFLNKGIQITLKDEREEEVREDSYHYEGGIKSYVDLLN**

6 785_SEPI 100.0% 99.7%  **DLKEIGDTDKTGTVIRFKADKEIFTETTVYNYETLQKRIRELAFLNKGIQITLKDEREEEVREDSYHYEGGIKSYVDLLN**

7 VSE49 100.0% 99.7%  **DLKEIGDTDKTGTAIRFKADKEIFTETTVYNYETLQKRIRELAFLNKGIQITLKDEREEEVREDSYHYEGGIKSYVDLLN**

8 VSE57 100.0% 99.7%  **DLKEIGDTDKTGTAIRFKADKEIFTETTVYNYETLQKRIRELAFLNKGIQITLKDEREEEVREDSYHYEGGIKSYVDLLN**

9 SE45 100.0% 99.7%  **DLKEIGDTDKTGTAIRFKADKEIFTETTVYNYETLQKRIRELAFLNKGIQITLKDEREEEVREDSYHYEGGIKSYVDLLN**

10 SE40 100.0% 99.7%  **DLKEIGDTDKTGTAIRFKADKEIFTETTVYNYETLQKRIRELAFLNKGIQITLKDEREEEVREDSYHYEGGIKSYVDLLN**

11 32A 100.0% 99.7%  **DLKEIGDTDKTGTVIRFKADKEIFTETTVYNYETLQKRIRELAFLNKGIQITLKDEREEEVREDSYHYEGGIKSYVDLLN**

12 HD66 100.0% 100.0%  **DLKEIGDTDKTGTAIRFKADKEIFTETTVYNYETLQKRIRELAFLNKGIQITLKDEREEEVREDSYHYEGGIKSYVDLLN**

13 HD43 100.0% 100.0%  **DLKEIGDTDKTGTAIRFKADKEIFTETTVYNYETLQKRIRELAFLNKGIQITLKDEREEEVREDSYHYEGGIKSYVDLLN**

14 HD33 100.0% 100.0%  **DLKEIGDTDKTGTAIRFKADKEIFTETTVYNYETLQKRIRELAFLNKGIQITLKDEREEEVREDSYHYEGGIKSYVDLLN**

15 DE0525 100.0% 99.7%  **DLKEIGDTDKTGTVIRFKADKEIFTETTVYNYETLQKRIRELAFLNKGIQITLKDEREEEVREDSYHYEGGIKSYVDLLN**

16 4928STDY7071543 100.0% 99.7%  **DLKEIGDTDKTGTAIRFKADKEIFTETTVYNYETLQKRIRELAFLNKGIQITLKDEREEEVREDSYHYEGGIKSYVDLLN**

17 BAV 100.0% 99.7%  **DLKEIGDTDKTGTVIRFKADKEIFTETTVYNYETLQKRIRELAFLNKGIQITLKDEREEEVREDSYHYEGGIKSYVDLLN**

18 APC 100.0% 99.7%  **DLKEIGDTDKTGTAIRFKADKEIFTETTVYNYETLQKRIRELAFLNKGIQITLKDEREEEIREDSYHYEGGIKSYVDLLN**

19 SESURV_p1_0557 100.0% 100.0%  **DLKEIGDTDKTGTAIRFKADKEIFTETTVYNYETLQKRIRELAFLNKGIQITLKDEREEEVREDSYHYEGGIKSYVDLLN**

20 SESURV_p1_0612 100.0% 99.8%  **DLKEIGDTDKTGTAIRFKADKEIFTETTVYNYETLQKRIRELAFLNKGIQITLKDEREEEVREDSYHYEGGIKSYVDLLN**

21 SESURV_p4_1553 100.0% 99.7%  **DLKEIGDTDKTGTVIRFKADKEIFTETTVYNYETLQKRIRELAFLNKGIQITLKDEREEEVREDSYHYEGGIKSYVDLLN**

22 SESURV_p1_1200 100.0% 100.0%  **DLKEIGDTDKTGTAIRFKADKEIFTETTVYNYETLQKRIRELAFLNKGIQITLKDEREEEVREDSYHYEGGIKSYVDLLN**

23 SESURV_p3_0825 90.7% 100.0%  **DLKEIGDTDKTGTAIRFKADKEIFTETTVYNYETLQKRIRELAFLNKGIQITLKDEREEEVREDSYHYEGGIKSYVDLLN**

24 JH 100.0% 99.8%  **DLKEIGDTDKTGTAIRFKADKEIFTETTVYNYETLQKRIRELAFLNKGIQITLKDEREEEVREDSYHYEGGIKSYVDLLN**

25 Z0118SE0132 100.0% 99.8%  **DLKEIGDTDKTGTAIRFKADKEIFTETTVYNYETLQKRIRELAFLNKGIQITLKDEREEEVREDSYHYEGGIKSYVDLLN**

26 R5981 100.0% 99.8%  **DLKEIGDTDKTGTAIRFKADKEIFTETTVYNYETLQKRIRELAFLNKGIQITLKDEREEEVREDSYHYEGGIKSYVDLLN**

27 JH-S-3 100.0% 99.8%  **DLKEIGDTDKTGTAIRFKADKEIFTETTVYNYETLQKRIRELAFLNKGIQITLKDEREEEVREDSYHYEGGIKSYVDLLN**

28 JH-S-1 100.0% 99.8%  **DLKEIGDTDKTGTAIRFKADKEIFTETTVYNYETLQKRIRELAFLNKGIQITLKDEREEEVREDSYHYEGGIKSYVDLLN**

29 s2 100.0% 99.7%  **DLKEIGDTDKTGTVIRFKADKEIFTETTVYNYETLQKRIRELAFLNKGIQITLKDEREEEVREDSYHYEGGIKSYVDLLN**

30 S43 100.0% 99.8%  **DLKEIGDTDKTGTAIRFKADKEIFTETTVYNYETLQKRIRELAFLNKGIQITLKDEREEEVREDSYHYEGGIKSYVDLLN**

31 S17W 90.7% 99.7%  **DLKEIGDTDKTGTVIRFKADKEIFTETTVYNYETLQKRIRELAFLNKGIQITLKDEREEEVREDSYHYEGGIKSYVDLLN**

32 48 100.0% 99.7%  **DLKEIGDTDKTGTVIRFKADKEIFTETTVYNYETLQKRIRELAFLNKGIQITLKDEREEEVREDSYHYEGGIKSYVDLLN**

33 ABKVF 100.0% 99.7%  **DLKEIGDTDKTGTVIRFKADKEIFTETTVYNYETLQKRIRELAFLNKGIQITLKDEREEEVREDSYHYEGGIKSYVDLLN**

34 CICARIA 100.0% 100.0%  **DLKEIGDTDKTGTAIRFKADKEIFTETTVYNYETLQKRIRELAFLNKGIQITLKDEREEEVREDSYHYEGGIKSYVDLLN**

35 SKN25lux 90.7% 100.0%  **DLKEIGDTDKTGTAIRFKADKEIFTETTVYNYETLQKRIRELAFLNKGIQITLKDEREEEVREDSYHYEGGIKSYVDLLN**

36 HMSC074F11 90.7% 99.7%  **DLKEIGDTDKTGTVIRFKADKEIFTETTVYNYETLQKRIRELAFLNKGIQITLKDEREEEVREDSYHYEGGIKSYVDLLN**

37 HMSC068G11 100.0% 99.7%  **DLKEIGDTDKTGTVIRFKADKEIFTETTVYNYETLQKRIRELAFLNKGIQITLKDEREEEVREDSYHYEGGIKSYVDLLN**

38 NIHLM037 90.7% 99.7%  **DLKEIGDTDKTGTVIRFKADKEIFTETTVYNYETLQKRIRELAFLNKGIQITLKDEREEEVREDSYHYEGGIKSYVDLLN**

39 NIHLM015 90.7% 99.7%  **DLKEIGDTDKTGTVIRFKADKEIFTETTVYNYETLQKRIRELAFLNKGIQITLKDEREEEVREDSYHYEGGIKSYVDLLN**

consensus/100%  **DLKEIGDTDKTGTsIRFKADKEIFTETTVYNYETLQKRIRELAFLNKGIQITLKDEREEElREDSYHYEGGIKSYVDLLN**

consensus/90%  **DLKEIGDTDKTGTsIRFKADKEIFTETTVYNYETLQKRIRELAFLNKGIQITLKDEREEEVREDSYHYEGGIKSYVDLLN**

consensus/80%  **DLKEIGDTDKTGTsIRFKADKEIFTETTVYNYETLQKRIRELAFLNKGIQITLKDEREEEVREDSYHYEGGIKSYVDLLN**

consensus/70%  **DLKEIGDTDKTGTsIRFKADKEIFTETTVYNYETLQKRIRELAFLNKGIQITLKDEREEEVREDSYHYEGGIKSYVDLLN**

cov pid **241**  **: . . . . 3 . .** **320**

1 ATCC35984 100.0% 100.0%  **ENKEPLHDEPIYIHQSKDDIEVEIALQYNSGYATNLLTYANNIHTYEGGTHEDGFKRALTRVLNSYGTQSKIIKEDKDRL**

2 ATCC12228 90.7% 100.0%  **ENKEPLHDEPIYIHQSKDDIEVEIALQYNSGYATNLLTYANNIHTYEGGTHEDGFKRALTRVLNSYGTQSKIIKEDKDRL**

3 36AM 90.7% 99.7%  **ENKEPLHDEPIYIHQSKDDIEVEIALQYNSGYATNLLTYANNIHTYEGGTHEDGFKRALTRVLNSYGTQSKIIKEDKDRL**

4 48AF 100.0% 100.0%  **ENKEPLHDEPIYIHQSKDDIEVEIALQYNSGYATNLLTYANNIHTYEGGTHEDGFKRALTRVLNSYGTQSKIIKEDKDRL**

5 54AF 100.0% 99.7%  **ENKEPLHDEPIYIHQSKDDIEVEIALQYNSGYATNLLTYANNIHTYEGGTHEDGFKRALTRVLNSYGTQSKIIKEDKDRL**

6 785_SEPI 100.0% 99.7%  **ENKEPLHDEPIYIHQSKDDIEVEIALQYNSGYATNLLTYANNIHTYEGGTHEDGFKRALTRVLNSYGTQSKIIKEDKDRL**

7 VSE49 100.0% 99.7%  **ENKEPLHDEPIYIHQSKDDIEVEIALQYNSGYATNLLTYANNIHTYEGGTHEDGFKRALTRVLNSYGTQNKIIKEDKDRL**

8 VSE57 100.0% 99.7%  **ENKEPLHDEPIYIHQSKDDIEVEIALQYNSGYATNLLTYANNIHTYEGGTHEDGFKRALTRVLNSYGTQNKIIKEDKDRL**

9 SE45 100.0% 99.7%  **ENKEPLHDEPIYIHQSKDDIEVEIALQYNSGYATNLLTYANNIHTYEGGTHEDGFKRALTRVLNSYGTQNKIIKEDKDRL**

10 SE40 100.0% 99.7%  **ENKEPLHDEPIYIHQSKDDIEVEIALQYNSGYATNLLTYANNIHTYEGGTHEDGFKRALTRVLNSYGTQNKIIKEDKDRL**

11 32A 100.0% 99.7%  **ENKEPLHDEPIYIHQSKDDIEVEIALQYNSGYATNLLTYANNIHTYEGGTHEDGFKRALTRVLNSYGTQSKIIKEDKDRL**

12 HD66 100.0% 100.0%  **ENKEPLHDEPIYIHQSKDDIEVEIALQYNSGYATNLLTYANNIHTYEGGTHEDGFKRALTRVLNSYGTQSKIIKEDKDRL**

13 HD43 100.0% 100.0%  **ENKEPLHDEPIYIHQSKDDIEVEIALQYNSGYATNLLTYANNIHTYEGGTHEDGFKRALTRVLNSYGTQSKIIKEDKDRL**

14 HD33 100.0% 100.0%  **ENKEPLHDEPIYIHQSKDDIEVEIALQYNSGYATNLLTYANNIHTYEGGTHEDGFKRALTRVLNSYGTQSKIIKEDKDRL**

15 DE0525 100.0% 99.7%  **ENKEPLHDEPIYIHQSKDDIEVEIALQYNSGYATNLLTYANNIHTYEGGTHEDGFKRALTRVLNSYGTQSKIIKEDKDRL**

16 4928STDY7071543 100.0% 99.7%  **ENKEPLHDEPIYIHQSKDDIEVEIALQYNSGYATNLLTYANNIHTYEGGTHEDGFKRALTRVLNSYGTQSKIIKEDKDRL**

17 BAV 100.0% 99.7%  **ENKEPLHDEPIYIHQSKDDIEVEIALQYNSGYATNLLTYANNIHTYEGGTHEDGFKRALTRVLNSYGTQSKIIKEDKDRL**

18 APC 100.0% 99.7%  **ENKEPLHDEPIYIHQSKDDIEVEIALQYNSGYATNLLTYANNIHTYEGGTHEDGFKRALTRVLNSYGTQSKIIKEDKDRL**

19 SESURV_p1_0557 100.0% 100.0%  **ENKEPLHDEPIYIHQSKDDIEVEIALQYNSGYATNLLTYANNIHTYEGGTHEDGFKRALTRVLNSYGTQSKIIKEDKDRL**

20 SESURV_p1_0612 100.0% 99.8%  **ENKEPLHDEPIYIHQSKDDIEVEIALQYNSGYATNLLTYANNIHTYEGGTHEDGFKRALTRVLNSYGTQSKIIKEDKDRL**

21 SESURV_p4_1553 100.0% 99.7%  **ENKEPLHDEPIYIHQSKDDIEVEIALQYNSGYATNLLTYANNIHTYEGGTHEDGFKRALTRVLNSYGTQSKIIKEDKDRL**

22 SESURV_p1_1200 100.0% 100.0%  **ENKEPLHDEPIYIHQSKDDIEVEIALQYNSGYATNLLTYANNIHTYEGGTHEDGFKRALTRVLNSYGTQSKIIKEDKDRL**

23 SESURV_p3_0825 90.7% 100.0%  **ENKEPLHDEPIYIHQSKDDIEVEIALQYNSGYATNLLTYANNIHTYEGGTHEDGFKRALTRVLNSYGTQSKIIKEDKDRL**

24 JH 100.0% 99.8%  **ENKEPLHDEPIYIHQSKDDIEVEIALQYNSGYATNLLTYANNIHTYEGGTHEDGFKRALTRVLNSYGTQSKIIKEDKDRL**

25 Z0118SE0132 100.0% 99.8%  **ENKEPLHDEPIYIHQSKDDIEVEIALQYNSGYATNLLTYANNIHTYEGGTHEDGFKRALTRVLNSYGTQSKIIKEDKDRL**

26 R5981 100.0% 99.8%  **ENKEPLHDEPIYIHQSKDDIEVEIALQYNSGYATNLLTYANNIHTYEGGTHEDGFKRALTRVLNSYGTQSKIIKEDKDRL**

27 JH-S-3 100.0% 99.8%  **ENKEPLHDEPIYIHQSKDDIEVEIALQYNSGYATNLLTYANNIHTYEGGTHEDGFKRALTRVLNSYGTQSKIIKEDKDRL**

28 JH-S-1 100.0% 99.8%  **ENKEPLHDEPIYIHQSKDDIEVEIALQYNSGYATNLLTYANNIHTYEGGTHEDGFKRALTRVLNSYGTQSKIIKEDKDRL**

29 s2 100.0% 99.7%  **ENKEPLHDEPIYIHQSKDDIEVEIALQYNSGYATNLLTYANNIHTYEGGTHEDGFKRALTRVLNSYGTQSKIIKEDKDRL**

30 S43 100.0% 99.8%  **ENKEPLHDEPIYIHQSKDDIEVEIALQYNSGYATNLLTYANNIHTYEGGTHEDGFKRALTRVLNSYGTQSKIIKEDKDRL**

31 S17W 90.7% 99.7%  **ENKEPLHDEPIYIHQSKDDIEVEIALQYNSGYATNLLTYANNIHTYEGGTHEDGFKRALTRVLNSYGTQSKIIKEDKDRL**

32 48 100.0% 99.7%  **ENKEPLHDEPIYIHQSKDDIEVEIALQYNSGYATNLLTYANNIHTYEGGTHEDGFKRALTRVLNSYGTQSKIIKEDKDRL**

33 ABKVF 100.0% 99.7%  **ENKEPLHDEPIYIHQSKDDIEVEIALQYNSGYATNLLTYANNIHTYEGGTHEDGFKRALTRVLNSYGTQSKIIKEDKDRL**

34 CICARIA 100.0% 100.0%  **ENKEPLHDEPIYIHQSKDDIEVEIALQYNSGYATNLLTYANNIHTYEGGTHEDGFKRALTRVLNSYGTQSKIIKEDKDRL**

35 SKN25lux 90.7% 100.0%  **ENKEPLHDEPIYIHQSKDDIEVEIALQYNSGYATNLLTYANNIHTYEGGTHEDGFKRALTRVLNSYGTQSKIIKEDKDRL**

36 HMSC074F11 90.7% 99.7%  **ENKEPLHDEPIYIHQSKDDIEVEIALQYNSGYATNLLTYANNIHTYEGGTHEDGFKRALTRVLNSYGTQSKIIKEDKDRL**

37 HMSC068G11 100.0% 99.7%  **ENKEPLHDEPIYIHQSKDDIEVEIALQYNSGYATNLLTYANNIHTYEGGTHEDGFKRALTRVLNSYGTQSKIIKEDKDRL**

38 NIHLM037 90.7% 99.7%  **ENKEPLHDEPIYIHQSKDDIEVEIALQYNSGYATNLLTYANNIHTYEGGTHEDGFKRALTRVLNSYGTQSKIIKEDKDRL**

39 NIHLM015 90.7% 99.7%  **ENKEPLHDEPIYIHQSKDDIEVEIALQYNSGYATNLLTYANNIHTYEGGTHEDGFKRALTRVLNSYGTQSKIIKEDKDRL**

consensus/100%  **ENKEPLHDEPIYIHQSKDDIEVEIALQYNSGYATNLLTYANNIHTYEGGTHEDGFKRALTRVLNSYGTQsKIIKEDKDRL**

consensus/90%  **ENKEPLHDEPIYIHQSKDDIEVEIALQYNSGYATNLLTYANNIHTYEGGTHEDGFKRALTRVLNSYGTQsKIIKEDKDRL**

consensus/80%  **ENKEPLHDEPIYIHQSKDDIEVEIALQYNSGYATNLLTYANNIHTYEGGTHEDGFKRALTRVLNSYGTQSKIIKEDKDRL**

consensus/70%  **ENKEPLHDEPIYIHQSKDDIEVEIALQYNSGYATNLLTYANNIHTYEGGTHEDGFKRALTRVLNSYGTQSKIIKEDKDRL**

cov pid **321**  **. . : . . . . 4** **400**

1 ATCC35984 100.0% 100.0%  **SGEDTREGLTAVVSIKHGDPQFEGQTKTKLGNSEVRQVVDRLFSEHFERFLYENPSVGRIIVEKGIMASRARVAAKKARE**

2 ATCC12228 90.7% 100.0%  **SGEDTREGLTAVVSIKHGDPQFEGQTKTKLGNSEVRQVVDRLFSEHFERFLYENPSVGRIIVEKGIMASRARVAAKKARE**

3 36AM 90.7% 99.7%  **SGEDTREGLTAVVSIKHGDPQFEGQTKTKLGNSEVRQVVDRLFSEHFERFLYENPSVGRIIVEKGIMASRARVAAKKARE**

4 48AF 100.0% 100.0%  **SGEDTREGLTAVVSIKHGDPQFEGQTKTKLGNSEVRQVVDRLFSEHFERFLYENPSVGRIIVEKGIMASRARVAAKKARE**

5 54AF 100.0% 99.7%  **SGEDTREGLTAVVSIKHGDPQFEGQTKTKLGNSEVRQVVDRLFSEHFERFLYENPSVGRIIVEKGIMASRARVAAKKARE**

6 785_SEPI 100.0% 99.7%  **SGEDTREGLTAVVSIKHGDPQFEGQTKTKLGNSEVRQVVDRLFSEHFERFLYENPSVGRIIVEKGIMASRARVAAKKARE**

7 VSE49 100.0% 99.7%  **SGEDTREGLTAVVSIKHGDPQFEGQTKTKLGNSEVRQVVDRLFSEHFERFLYENPSVGRIIVEKGIMASRARVAAKKARE**

8 VSE57 100.0% 99.7%  **SGEDTREGLTAVVSIKHGDPQFEGQTKTKLGNSEVRQVVDRLFSEHFERFLYENPSVGRIIVEKGIMASRARVAAKKARE**

9 SE45 100.0% 99.7%  **SGEDTREGLTAVVSIKHGDPQFEGQTKTKLGNSEVRQVVDRLFSEHFERFLYENPSVGRIIVEKGIMASRARVAAKKARE**

10 SE40 100.0% 99.7%  **SGEDTREGLTAVVSIKHGDPQFEGQTKTKLGNSEVRQVVDRLFSEHFERFLYENPSVGRIIVEKGIMASRARVAAKKARE**

11 32A 100.0% 99.7%  **SGEDTREGLTAVVSIKHGDPQFEGQTKTKLGNSEVRQVVDRLFSEHFERFLYENPSVGRIIVEKGIMASRARVAAKKARE**

12 HD66 100.0% 100.0%  **SGEDTREGLTAVVSIKHGDPQFEGQTKTKLGNSEVRQVVDRLFSEHFERFLYENPSVGRIIVEKGIMASRARVAAKKARE**

13 HD43 100.0% 100.0%  **SGEDTREGLTAVVSIKHGDPQFEGQTKTKLGNSEVRQVVDRLFSEHFERFLYENPSVGRIIVEKGIMASRARVAAKKARE**

14 HD33 100.0% 100.0%  **SGEDTREGLTAVVSIKHGDPQFEGQTKTKLGNSEVRQVVDRLFSEHFERFLYENPSVGRIIVEKGIMASRARVAAKKARE**

15 DE0525 100.0% 99.7%  **SGEDTREGLTAVVSIKHGDPQFEGQTKTKLGNSEVRQVVDRLFSEHFERFLYENPSVGRIIVEKGIMASRARVAAKKARE**

16 4928STDY7071543 100.0% 99.7%  **SGEDTREGLTAVVSIKHGDPQFEGQTKTKLGNSEVRQVVDRLFSEHFERFLYENPSVGRIIVEKGIMASRARVAAKKARE**

17 BAV 100.0% 99.7%  **SGEDTREGLTAVVSIKHGDPQFEGQTKTKLGNSEVRQVVDRLFSEHFERFLYENPSVGRIIVEKGIMASRARVAAKKARE**

18 APC 100.0% 99.7%  **SGEDTREGLTAVVSIKHGDPQFEGQTKTKLGNSEVRQVVDRLFSEHFERFLYENPSVGRIIVEKGIMASRARVAAKKARE**

19 SESURV_p1_0557 100.0% 100.0%  **SGEDTREGLTAVVSIKHGDPQFEGQTKTKLGNSEVRQVVDRLFSEHFERFLYENPSVGRIIVEKGIMASRARVAAKKARE**

20 SESURV_p1_0612 100.0% 99.8%  **SGEDTREGLTAVVSIKHGDPQFEGQTKTKLGNSEVRQVVDRLFSEHFERFLYENPSVGRIIVEKGIMASRARVAAKKARE**

21 SESURV_p4_1553 100.0% 99.7%  **SGEDTREGLTAVVSIKHGDPQFEGQTKTKLGNSEVRQVVDRLFSEHFERFLYENPSVGRIIVEKGIMASRARVAAKKARE**

22 SESURV_p1_1200 100.0% 100.0%  **SGEDTREGLTAVVSIKHGDPQFEGQTKTKLGNSEVRQVVDRLFSEHFERFLYENPSVGRIIVEKGIMASRARVAAKKARE**

23 SESURV_p3_0825 90.7% 100.0%  **SGEDTREGLTAVVSIKHGDPQFEGQTKTKLGNSEVRQVVDRLFSEHFERFLYENPSVGRIIVEKGIMASRARVAAKKARE**

24 JH 100.0% 99.8%  **SGEDTREGLTAVVSIKHGDPQFEGQTKTKLGNSEVRQVVDRLFSEHFERFLYENPSVGRIIVEKGIMASRARVAAKKARE**

25 Z0118SE0132 100.0% 99.8%  **SGEDTREGLTAVVSIKHGDPQFEGQTKTKLGNSEVRQVVDRLFSEHFERFLYENPSVGRIIVEKGIMASRARVAAKKARE**

26 R5981 100.0% 99.8%  **SGEDTREGLTAVVSIKHGDPQFEGQTKTKLGNSEVRQVVDRLFSEHFERFLYENPSVGRIIVEKGIMASRARVAAKKARE**

27 JH-S-3 100.0% 99.8%  **SGEDTREGLTAVVSIKHGDPQFEGQTKTKLGNSEVRQVVDRLFSEHFERFLYENPSVGRIIVEKGIMASRARVAAKKARE**

28 JH-S-1 100.0% 99.8%  **SGEDTREGLTAVVSIKHGDPQFEGQTKTKLGNSEVRQVVDRLFSEHFERFLYENPSVGRIIVEKGIMASRARVAAKKARE**

29 s2 100.0% 99.7%  **SGEDTREGLTAVVSIKHGDPQFEGQTKTKLGNSEVRQVVDRLFSEHFERFLYENPSVGRIIVEKGIMASRARVAAKKARE**

30 S43 100.0% 99.8%  **SGEDTREGLTAVVSIKHGDPQFEGQTKTKLGNSEVRQVVDRLFSEHFERFLYENPSVGRIIVEKGIMASRARVAAKKARE**

31 S17W 90.7% 99.7%  **SGEDTREGLTAVVSIKHGDPQFEGQTKTKLGNSEVRQVVDRLFSEHFERFLYENPSVGRIIVEKGIMASRARVAAKKARE**

32 48 100.0% 99.7%  **SGEDTREGLTAVVSIKHGDPQFEGQTKTKLGNSEVRQVVDRLFSEHFERFLYENPSVGRIIVEKGIMASRARVAAKKARE**

33 ABKVF 100.0% 99.7%  **SGEDTREGLTAVVSIKHGDPQFEGQTKTKLGNSEVRQVVDRLFSEHFERFLYENPSVGRIIVEKGIMASRARVAAKKARE**

34 CICARIA 100.0% 100.0%  **SGEDTREGLTAVVSIKHGDPQFEGQTKTKLGNSEVRQVVDRLFSEHFERFLYENPSVGRIIVEKGIMASRARVAAKKARE**

35 SKN25lux 90.7% 100.0%  **SGEDTREGLTAVVSIKHGDPQFEGQTKTKLGNSEVRQVVDRLFSEHFERFLYENPSVGRIIVEKGIMASRARVAAKKARE**

36 HMSC074F11 90.7% 99.7%  **SGEDTREGLTAVVSIKHGDPQFEGQTKTKLGNSEVRQVVDRLFSEHFERFLYENPSVGRIIVEKGIMASRARVAAKKARE**

37 HMSC068G11 100.0% 99.7%  **SGEDTREGLTAVVSIKHGDPQFEGQTKTKLGNSEVRQVVDRLFSEHFERFLYENPSVGRIIVEKGIMASRARVAAKKARE**

38 NIHLM037 90.7% 99.7%  **SGEDTREGLTAVVSIKHGDPQFEGQTKTKLGNSEVRQVVDRLFSEHFERFLYENPSVGRIIVEKGIMASRARVAAKKARE**

39 NIHLM015 90.7% 99.7%  **SGEDTREGLTAVVSIKHGDPQFEGQTKTKLGNSEVRQVVDRLFSEHFERFLYENPSVGRIIVEKGIMASRARVAAKKARE**

consensus/100%  **SGEDTREGLTAVVSIKHGDPQFEGQTKTKLGNSEVRQVVDRLFSEHFERFLYENPSVGRIIVEKGIMASRARVAAKKARE**

consensus/90%  **SGEDTREGLTAVVSIKHGDPQFEGQTKTKLGNSEVRQVVDRLFSEHFERFLYENPSVGRIIVEKGIMASRARVAAKKARE**

consensus/80%  **SGEDTREGLTAVVSIKHGDPQFEGQTKTKLGNSEVRQVVDRLFSEHFERFLYENPSVGRIIVEKGIMASRARVAAKKARE**

consensus/70%  **SGEDTREGLTAVVSIKHGDPQFEGQTKTKLGNSEVRQVVDRLFSEHFERFLYENPSVGRIIVEKGIMASRARVAAKKARE**

cov pid **401**  **. . . . : . . .** **480**

1 ATCC35984 100.0% 100.0%  **VTRRKSALDVSSLPGKLADCSSKNPEESEIFLVEGDSAGGSTKSGRDSRTQAILPLRGKILNVEKARLDRILNNNEIRQM**

2 ATCC12228 90.7% 100.0%  **VTRRKSALDVSSLPGKLADCSSKNPEESEIFLVEGDSAGGSTKSGRDSRTQAILPLRGKILNVEKARLDRILNNNEIRQM**

3 36AM 90.7% 99.7%  **VTRRKSALDVSSLPGKLADCSSKNPEESEIFLVEGDSAGGSTKSGRDSRTQAILPLRGKILNVEKARLDRILNNNEIRQM**

4 48AF 100.0% 100.0%  **VTRRKSALDVSSLPGKLADCSSKNPEESEIFLVEGDSAGGSTKSGRDSRTQAILPLRGKILNVEKARLDRILNNNEIRQM**

5 54AF 100.0% 99.7%  **VTRRKSALDVSSLPGKLADCSSKNPEESEIFLVEGDSAGGSTKSGRDSRTQAILPLRGKILNVEKARLDRILNNNEIRQM**

6 785_SEPI 100.0% 99.7%  **VTRRKSALDVSSLPGKLADCSSKNPEESEIFLVEGDSAGGSTKSGRDSRTQAILPLRGKILNVEKARLDRILNNNEIRQM**

7 VSE49 100.0% 99.7%  **VTRRKSALDVSSLPGKLADCSSKNPEESEIFLVEGDSAGGSTKSGRDSRTQAILPLRGKILNVEKARLDRILNNNEIRQM**

8 VSE57 100.0% 99.7%  **VTRRKSALDVSSLPGKLADCSSKNPEESEIFLVEGDSAGGSTKSGRDSRTQAILPLRGKILNVEKARLDRILNNNEIRQM**

9 SE45 100.0% 99.7%  **VTRRKSALDVSSLPGKLADCSSKNPEESEIFLVEGDSAGGSTKSGRDSRTQAILPLRGKILNVEKARLDRILNNNEIRQM**

10 SE40 100.0% 99.7%  **VTRRKSALDVSSLPGKLADCSSKNPEESEIFLVEGDSAGGSTKSGRDSRTQAILPLRGKILNVEKARLDRILNNNEIRQM**

11 32A 100.0% 99.7%  **VTRRKSALDVSSLPGKLADCSSKNPEESEIFLVEGDSAGGSTKSGRDSRTQAILPLRGKILNVEKARLDRILNNNEIRQM**

12 HD66 100.0% 100.0%  **VTRRKSALDVSSLPGKLADCSSKNPEESEIFLVEGDSAGGSTKSGRDSRTQAILPLRGKILNVEKARLDRILNNNEIRQM**

13 HD43 100.0% 100.0%  **VTRRKSALDVSSLPGKLADCSSKNPEESEIFLVEGDSAGGSTKSGRDSRTQAILPLRGKILNVEKARLDRILNNNEIRQM**

14 HD33 100.0% 100.0%  **VTRRKSALDVSSLPGKLADCSSKNPEESEIFLVEGDSAGGSTKSGRDSRTQAILPLRGKILNVEKARLDRILNNNEIRQM**

15 DE0525 100.0% 99.7%  **VTRRKSALDVSSLPGKLADCSSKNPEESEIFLVEGDSAGGSTKSGRDSRTQAILPLRGKILNVEKARLDRILNNNEIRQM**

16 4928STDY7071543 100.0% 99.7%  **VTRRKSALDVSSLPGKLADCSSKNPEESEIFLVEGDSAGGSTKSGRDSRTQAILPLRGKILNVEKARLDRILNNNEIRQM**

17 BAV 100.0% 99.7%  **VTRRKSALDVSSLPGKLADCSSKNPEESEIFLVEGDSAGGSTKSGRDSRTQAILPLRGKILNVEKARLDRILNNNEIRQM**

18 APC 100.0% 99.7%  **VTRRKSALDVSSLPGKLADCSSKNPEESEIFLVEGDSAGGSTKSGRDSRTQAILPLRGKILNVEKARLDRILNNNEIRQM**

19 SESURV_p1_0557 100.0% 100.0%  **VTRRKSALDVSSLPGKLADCSSKNPEESEIFLVEGDSAGGSTKSGRDSRTQAILPLRGKILNVEKARLDRILNNNEIRQM**

20 SESURV_p1_0612 100.0% 99.8%  **VTRRKSALDVSSLPGKLADCSSKNPEESEIFLVEGDSAGGSTKSGRDSRTQAILPLRGKILNVEKARLDRILNNNEIRQM**

21 SESURV_p4_1553 100.0% 99.7%  **VTRRKSALDVSSLPGKLADCSSKNPEESEIFLVEGDSAGGSTKSGRDSRTQAILPLRGKILNVEKARLDRILNNNEIRQM**

22 SESURV_p1_1200 100.0% 100.0%  **VTRRKSALDVSSLPGKLADCSSKNPEESEIFLVEGDSAGGSTKSGRDSRTQAILPLRGKILNVEKARLDRILNNNEIRQM**

23 SESURV_p3_0825 90.7% 100.0%  **VTRRKSALDVSSLPGKLADCSSKNPEESEIFLVEGDSAGGSTKSGRDSRTQAILPLRGKILNVEKARLDRILNNNEIRQM**

24 JH 100.0% 99.8%  **VTRRKSALDVSSLPGKLADCSSKNPEESEIFLVEGDSAGGSTKSGRDSRTQAILPLRGKILNVEKARLDRILNNNEIRQM**

25 Z0118SE0132 100.0% 99.8%  **VTRRKSALDVSSLPGKLADCSSKNPEESEIFLVEGDSAGGSTKSGRDSRTQAILPLRGKILNVEKARLDRILNNNEIRQM**

26 R5981 100.0% 99.8%  **VTRRKSALDVSSLPGKLADCSSKNPEESEIFLVEGDSAGGSTKSGRDSRTQAILPLRGKILNVEKARLDRILNNNEIRQM**

27 JH-S-3 100.0% 99.8%  **VTRRKSALDVSSLPGKLADCSSKNPEESEIFLVEGDSAGGSTKSGRDSRTQAILPLRGKILNVEKARLDRILNNNEIRQM**

28 JH-S-1 100.0% 99.8%  **VTRRKSALDVSSLPGKLADCSSKNPEESEIFLVEGDSAGGSTKSGRDSRTQAILPLRGKILNVEKARLDRILNNNEIRQM**

29 s2 100.0% 99.7%  **VTRRKSALDVSSLPGKLADCSSKNPEESEIFLVEGDSAGGSTKSGRDSRTQAILPLRGKILNVEKARLDRILNNNEIRQM**

30 S43 100.0% 99.8%  **VTRRKSALDVSSLPGKLADCSSKNPEESEIFLVEGDSAGGSTKSGRDSRTQAILPLRGKILNVEKARLDRILNNNEIRQM**

31 S17W 90.7% 99.7%  **VTRRKSALDVSSLPGKLADCSSKNPEESEIFLVEGDSAGGSTKSGRDSRTQAILPLRGKILNVEKARLDRILNNNEIRQM**

32 48 100.0% 99.7%  **VTRRKSALDVSSLPGKLADCSSKNPEESEIFLVEGDSAGGSTKSGRDSRTQAILPLRGKILNVEKARLDRILNNNEIRQM**

33 ABKVF 100.0% 99.7%  **VTRRKSALDVSSLPGKLADCSSKNPEESEIFLVEGDSAGGSTKSGRDSRTQAILPLRGKILNVEKARLDRILNNNEIRQM**

34 CICARIA 100.0% 100.0%  **VTRRKSALDVSSLPGKLADCSSKNPEESEIFLVEGDSAGGSTKSGRDSRTQAILPLRGKILNVEKARLDRILNNNEIRQM**

35 SKN25lux 90.7% 100.0%  **VTRRKSALDVSSLPGKLADCSSKNPEESEIFLVEGDSAGGSTKSGRDSRTQAILPLRGKILNVEKARLDRILNNNEIRQM**

36 HMSC074F11 90.7% 99.7%  **VTRRKSALDVSSLPGKLADCSSKNPEESEIFLVEGDSAGGSTKSGRDSRTQAILPLRGKILNVEKARLDRILNNNEIRQM**

37 HMSC068G11 100.0% 99.7%  **VTRRKSALDVSSLPGKLADCSSKNPEESEIFLVEGDSAGGSTKSGRDSRTQAILPLRGKILNVEKARLDRILNNNEIRQM**

38 NIHLM037 90.7% 99.7%  **VTRRKSALDVSSLPGKLADCSSKNPEESEIFLVEGDSAGGSTKSGRDSRTQAILPLRGKILNVEKARLDRILNNNEIRQM**

39 NIHLM015 90.7% 99.7%  **VTRRKSALDVSSLPGKLADCSSKNPEESEIFLVEGDSAGGSTKSGRDSRTQAILPLRGKILNVEKARLDRILNNNEIRQM**

consensus/100%  **VTRRKSALDVSSLPGKLADCSSKNPEESEIFLVEGDSAGGSTKSGRDSRTQAILPLRGKILNVEKARLDRILNNNEIRQM**

consensus/90%  **VTRRKSALDVSSLPGKLADCSSKNPEESEIFLVEGDSAGGSTKSGRDSRTQAILPLRGKILNVEKARLDRILNNNEIRQM**

consensus/80%  **VTRRKSALDVSSLPGKLADCSSKNPEESEIFLVEGDSAGGSTKSGRDSRTQAILPLRGKILNVEKARLDRILNNNEIRQM**

consensus/70%  **VTRRKSALDVSSLPGKLADCSSKNPEESEIFLVEGDSAGGSTKSGRDSRTQAILPLRGKILNVEKARLDRILNNNEIRQM**

cov pid **481**  **. 5 . . . . : .** **560**

1 ATCC35984 100.0% 100.0%  **ITAFGTGIGGEFDISKARYHKIVIMTDADVDGAHIRTLLLTFFYRFMRPLIEAGYVYIAQPPLYKLTQGKQKYYVFNDRE**

2 ATCC12228 90.7% 100.0%  **ITAFGTGIGGEFDISKARYHKIVIMTDADVDGAHIRTLLLTFFYRFMRPLIEAGYVYIAQPPLYKLTQGKQKYYVFNDRE**

3 36AM 90.7% 99.7%  **ITAFGTGIGGEFDISKARYHKIVIMTDADVDGAHIRTLLLTFFYRFMRPLIEAGYVYIAQPPLYKLTQGKQKYYVFNDRE**

4 48AF 100.0% 100.0%  **ITAFGTGIGGEFDISKARYHKIVIMTDADVDGAHIRTLLLTFFYRFMRPLIEAGYVYIAQPPLYKLTQGKQKYYVFNDRE**

5 54AF 100.0% 99.7%  **ITAFGTGIGGEFDISKARYHKIVIMTDADVDGAHIRTLLLTFFYRFMRPLIEAGYVYIAQPPLYKLTQGKQKYYVFNDRE**

6 785_SEPI 100.0% 99.7%  **ITAFGTGIGGEFDISKARYHKIVIMTDADVDGAHIRTLLLTFFYRFMRPLIEAGYVYIAQPPLYKLTQGKQKYYVFNDRE**

7 VSE49 100.0% 99.7%  **ITAFGTGIGGEFDISKARYHKIVIMTDADVDGAHIRTLLLTFFYRFMRPLIEAGYVYIAQPPLYKLTQGKQKYYVFNDRE**

8 VSE57 100.0% 99.7%  **ITAFGTGIGGEFDISKARYHKIVIMTDADVDGAHIRTLLLTFFYRFMRPLIEAGYVYIAQPPLYKLTQGKQKYYVFNDRE**

9 SE45 100.0% 99.7%  **ITAFGTGIGGEFDISKARYHKIVIMTDADVDGAHIRTLLLTFFYRFMRPLIEAGYVYIAQPPLYKLTQGKQKYYVFNDRE**

10 SE40 100.0% 99.7%  **ITAFGTGIGGEFDISKARYHKIVIMTDADVDGAHIRTLLLTFFYRFMRPLIEAGYVYIAQPPLYKLTQGKQKYYVFNDRE**

11 32A 100.0% 99.7%  **ITAFGTGIGGEFDISKARYHKIVIMTDADVDGAHIRTLLLTFFYRFMRPLIEAGYVYIAQPPLYKLTQGKQKYYVFNDRE**

12 HD66 100.0% 100.0%  **ITAFGTGIGGEFDISKARYHKIVIMTDADVDGAHIRTLLLTFFYRFMRPLIEAGYVYIAQPPLYKLTQGKQKYYVFNDRE**

13 HD43 100.0% 100.0%  **ITAFGTGIGGEFDISKARYHKIVIMTDADVDGAHIRTLLLTFFYRFMRPLIEAGYVYIAQPPLYKLTQGKQKYYVFNDRE**

14 HD33 100.0% 100.0%  **ITAFGTGIGGEFDISKARYHKIVIMTDADVDGAHIRTLLLTFFYRFMRPLIEAGYVYIAQPPLYKLTQGKQKYYVFNDRE**

15 DE0525 100.0% 99.7%  **ITAFGTGIGGEFDISKARYHKIVIMTDADVDGAHIRTLLLTFFYRFMRPLIEAGYVYIAQPPLYKLTQGKQKYYVFNDRE**

16 4928STDY7071543 100.0% 99.7%  **ITAFGTGIGGEFDISKARYHKIVIMTDADVDGAHIRTLLLTFFYRFMRPLIEAGYVYIAQPPLYKLTQGKQKYYVFNDRE**

17 BAV 100.0% 99.7%  **ITAFGTGIGGEFDISKARYHKIVIMTDADVDGAHIRTLLLTFFYRFMRPLIEAGYVYIAQPPLYKLTQGKQKYYVFNDRE**

18 APC 100.0% 99.7%  **ITAFGTGIGGEFDISKARYHKIVIMTDADVDGAHIRTLLLTFFYRFMRPLIEAGYVYIAQPPLYKLTQGKQKYYVFNDRE**

19 SESURV_p1_0557 100.0% 100.0%  **ITAFGTGIGGEFDISKARYHKIVIMTDADVDGAHIRTLLLTFFYRFMRPLIEAGYVYIAQPPLYKLTQGKQKYYVFNDRE**

20 SESURV_p1_0612 100.0% 99.8%  **ITAFGTGIGGEFDISKARYHKIVIMTDADVDGAHIRTLLLTFFYRFMRPLIEAGYVYIAQPPLYKLTQGKQKYYVFNDRE**

21 SESURV_p4_1553 100.0% 99.7%  **ITAFGTGIGGEFDISKARYHKIVIMTDADVDGAHIRTLLLTFFYRFMRPLIEAGYVYIAQPPLYKLTQGKQKYYVFNDRE**

22 SESURV_p1_1200 100.0% 100.0%  **ITAFGTGIGGEFDISKARYHKIVIMTDADVDGAHIRTLLLTFFYRFMRPLIEAGYVYIAQPPLYKLTQGKQKYYVFNDRE**

23 SESURV_p3_0825 90.7% 100.0%  **ITAFGTGIGGEFDISKARYHKIVIMTDADVDGAHIRTLLLTFFYRFMRPLIEAGYVYIAQPPLYKLTQGKQKYYVFNDRE**

24 JH 100.0% 99.8%  **ITAFGTGIGGEFDISKARYHKIVIMTDADVDGAHIRTLLLTFFYRFMRPLIEAGYVYIAQPPLYKLTQGKQKYYVFNDRE**

25 Z0118SE0132 100.0% 99.8%  **ITAFGTGIGGEFDISKARYHKIVIMTDADVDGAHIRTLLLTFFYRFMRPLIEAGYVYIAQPPLYKLTQGKQKYYVFNDRE**

26 R5981 100.0% 99.8%  **ITAFGTGIGGEFDISKARYHKIVIMTDADVDGAHIRTLLLTFFYRFMRPLIEAGYVYIAQPPLYKLTQGKQKYYVFNDRE**

27 JH-S-3 100.0% 99.8%  **ITAFGTGIGGEFDISKARYHKIVIMTDADVDGAHIRTLLLTFFYRFMRPLIEAGYVYIAQPPLYKLTQGKQKYYVFNDRE**

28 JH-S-1 100.0% 99.8%  **ITAFGTGIGGEFDISKARYHKIVIMTDADVDGAHIRTLLLTFFYRFMRPLIEAGYVYIAQPPLYKLTQGKQKYYVFNDRE**

29 s2 100.0% 99.7%  **ITAFGTGIGGEFDISKARYHKIVIMTDADVDGAHIRTLLLTFFYRFMRPLIEAGYVYIAQPPLYKLTQGKQKYYVFNDRE**

30 S43 100.0% 99.8%  **ITAFGTGIGGEFDISKARYHKIVIMTDADVDGAHIRTLLLTFFYRFMRPLIEAGYVYIAQPPLYKLTQGKQKYYVFNDRE**

31 S17W 90.7% 99.7%  **ITAFGTGIGGEFDISKARYHKIVIMTDADVDGAHIRTLLLTFFYRFMRPLIEAGYVYIAQPPLYKLTQGKQKYYVFNDRE**

32 48 100.0% 99.7%  **ITAFGTGIGGEFDISKARYHKIVIMTDADVDGAHIRTLLLTFFYRFMRPLIEAGYVYIAQPPLYKLTQGKQKYYVFNDRE**

33 ABKVF 100.0% 99.7%  **ITAFGTGIGGEFDISKARYHKIVIMTDADVDGAHIRTLLLTFFYRFMRPLIEAGYVYIAQPPLYKLTQGKQKYYVFNDRE**

34 CICARIA 100.0% 100.0%  **ITAFGTGIGGEFDISKARYHKIVIMTDADVDGAHIRTLLLTFFYRFMRPLIEAGYVYIAQPPLYKLTQGKQKYYVFNDRE**

35 SKN25lux 90.7% 100.0%  **ITAFGTGIGGEFDISKARYHKIVIMTDADVDGAHIRTLLLTFFYRFMRPLIEAGYVYIAQPPLYKLTQGKQKYYVFNDRE**

36 HMSC074F11 90.7% 99.7%  **ITAFGTGIGGEFDISKARYHKIVIMTDADVDGAHIRTLLLTFFYRFMRPLIEAGYVYIAQPPLYKLTQGKQKYYVFNDRE**

37 HMSC068G11 100.0% 99.7%  **ITAFGTGIGGEFDISKARYHKIVIMTDADVDGAHIRTLLLTFFYRFMRPLIEAGYVYIAQPPLYKLTQGKQKYYVFNDRE**

38 NIHLM037 90.7% 99.7%  **ITAFGTGIGGEFDISKARYHKIVIMTDADVDGAHIRTLLLTFFYRFMRPLIEAGYVYIAQPPLYKLTQGKQKYYVFNDRE**

39 NIHLM015 90.7% 99.7%  **ITAFGTGIGGEFDISKARYHKIVIMTDADVDGAHIRTLLLTFFYRFMRPLIEAGYVYIAQPPLYKLTQGKQKYYVFNDRE**

consensus/100%  **ITAFGTGIGGEFDISKARYHKIVIMTDADVDGAHIRTLLLTFFYRFMRPLIEAGYVYIAQPPLYKLTQGKQKYYVFNDRE**

consensus/90%  **ITAFGTGIGGEFDISKARYHKIVIMTDADVDGAHIRTLLLTFFYRFMRPLIEAGYVYIAQPPLYKLTQGKQKYYVFNDRE**

consensus/80%  **ITAFGTGIGGEFDISKARYHKIVIMTDADVDGAHIRTLLLTFFYRFMRPLIEAGYVYIAQPPLYKLTQGKQKYYVFNDRE**

consensus/70%  **ITAFGTGIGGEFDISKARYHKIVIMTDADVDGAHIRTLLLTFFYRFMRPLIEAGYVYIAQPPLYKLTQGKQKYYVFNDRE**

cov pid **561**  **. . . 6 . . . .** **640**

1 ATCC35984 100.0% 100.0%  **LDKLKQELNPSPKWSIARYKGLGEMNADQLWETTMNPEHRSMLQVRLEDAIDADQTFEMLMGDVVENRRQFIEDNAVYAN**

2 ATCC12228 90.7% 100.0%  **LDKLKQELNPSPKWSIARYKGLGEMNADQLWETTMNPEHRSMLQVRLEDAIDADQTFEMLMGDVVENRRQFIEDNAVYAN**

3 36AM 90.7% 99.7%  **LDKLKQELNPSPKWSIARYKGLGEMNADQLWETTMNPEHRSMLQVRLEDAIDADQTFEMLMGDVVENRRQFIEDNAVYAN**

4 48AF 100.0% 100.0%  **LDKLKQELNPSPKWSIARYKGLGEMNADQLWETTMNPEHRSMLQVRLEDAIDADQTFEMLMGDVVENRRQFIEDNAVYAN**

5 54AF 100.0% 99.7%  **LDKLKQELNPSPKWSIARYKGLGEMNADQLWETTMNPEHRSMLQVRLEDAIDADQTFEMLMGDVVENRRQFIEDNAVYAN**

6 785_SEPI 100.0% 99.7%  **LDKLKQELNPSPKWSIARYKGLGEMNADQLWETTMNPEHRSMLQVRLEDAIDADQTFEMLMGDVVENRRQFIEDNAVYAN**

7 VSE49 100.0% 99.7%  **LDKLKQELNPSPKWSIARYKGLGEMNADQLWETTMNPEHRSMLQVRLEDAIDADQTFEMLMGDVVENRRQFIEDNAVYAN**

8 VSE57 100.0% 99.7%  **LDKLKQELNPSPKWSIARYKGLGEMNADQLWETTMNPEHRSMLQVRLEDAIDADQTFEMLMGDVVENRRQFIEDNAVYAN**

9 SE45 100.0% 99.7%  **LDKLKQELNPSPKWSIARYKGLGEMNADQLWETTMNPEHRSMLQVRLEDAIDADQTFEMLMGDVVENRRQFIEDNAVYAN**

10 SE40 100.0% 99.7%  **LDKLKQELNPSPKWSIARYKGLGEMNADQLWETTMNPEHRSMLQVRLEDAIDADQTFEMLMGDVVENRRQFIEDNAVYAN**

11 32A 100.0% 99.7%  **LDKLKQELNPSPKWSIARYKGLGEMNADQLWETTMNPEHRSMLQVRLEDAIDADQTFEMLMGDVVENRRQFIEDNAVYAN**

12 HD66 100.0% 100.0%  **LDKLKQELNPSPKWSIARYKGLGEMNADQLWETTMNPEHRSMLQVRLEDAIDADQTFEMLMGDVVENRRQFIEDNAVYAN**

13 HD43 100.0% 100.0%  **LDKLKQELNPSPKWSIARYKGLGEMNADQLWETTMNPEHRSMLQVRLEDAIDADQTFEMLMGDVVENRRQFIEDNAVYAN**

14 HD33 100.0% 100.0%  **LDKLKQELNPSPKWSIARYKGLGEMNADQLWETTMNPEHRSMLQVRLEDAIDADQTFEMLMGDVVENRRQFIEDNAVYAN**

15 DE0525 100.0% 99.7%  **LDKLKQELNPSPKWSIARYKGLGEMNADQLWETTMNPEHRSMLQVRLEDAIDADQTFEMLMGDVVENRRQFIEDNAVYAN**

16 4928STDY7071543 100.0% 99.7%  **LDKLKQELNPSPKWSIARYKGLGEMNADQLWETTMNPEHRSMLQVRLEDAIDADQTFEMLMGDVVENRRQFIEDNAVYAN**

17 BAV 100.0% 99.7%  **LDKLKQELNPSPKWSIARYKGLGEMNADQLWETTMNPEHRSMLQVRLEDAIDADQTFEMLMGDVVENRRQFIEDNAVYAN**

18 APC 100.0% 99.7%  **LDKLKQELNPSPKWSIARYKGLGEMNADQLWETTMNPEHRSMLQVRLEDAIDADQTFEMLMGDVVENRRQFIEDNAVYAN**

19 SESURV_p1_0557 100.0% 100.0%  **LDKLKQELNPSPKWSIARYKGLGEMNADQLWETTMNPEHRSMLQVRLEDAIDADQTFEMLMGDVVENRRQFIEDNAVYAN**

20 SESURV_p1_0612 100.0% 99.8%  **LDKLKQELNPSPKWSIARYKGLGEMNADQLWETTMNPEHRSMLQVRLEDAIDADQTFEMLMGDVVENRRQFIEDNAVYAN**

21 SESURV_p4_1553 100.0% 99.7%  **LDKLKQELNPSPKWSIARYKGLGEMNADQLWETTMNPEHRSMLQVRLEDAIDADQTFEMLMGDVVENRRQFIEDNAVYAN**

22 SESURV_p1_1200 100.0% 100.0%  **LDKLKQELNPSPKWSIARYKGLGEMNADQLWETTMNPEHRSMLQVRLEDAIDADQTFEMLMGDVVENRRQFIEDNAVYAN**

23 SESURV_p3_0825 90.7% 100.0%  **LDKLKQELNPSPKWSIARYKGLGEMNADQLWETTMNPEHRSMLQVRLEDAIDADQTFEMLMGDVVENRRQFIEDNAVYAN**

24 JH 100.0% 99.8%  **LDKLKQELNPSPKWSIARYKGLGEMNADQLWETTMNPEHRSMLQVRLEDAIDADQTFEMLMGDVVENRRQFIEDNAVYAN**

25 Z0118SE0132 100.0% 99.8%  **LDKLKQELNPSPKWSIARYKGLGEMNADQLWETTMNPEHRSMLQVRLEDAIDADQTFEMLMGDVVENRRQFIEDNAVYAN**

26 R5981 100.0% 99.8%  **LDKLKQELNPSPKWSIARYKGLGEMNADQLWETTMNPEHRSMLQVRLEDAIDADQTFEMLMGDVVENRRQFIEDNAVYAN**

27 JH-S-3 100.0% 99.8%  **LDKLKQELNPSPKWSIARYKGLGEMNADQLWETTMNPEHRSMLQVRLEDAIDADQTFEMLMGDVVENRRQFIEDNAVYAN**

28 JH-S-1 100.0% 99.8%  **LDKLKQELNPSPKWSIARYKGLGEMNADQLWETTMNPEHRSMLQVRLEDAIDADQTFEMLMGDVVENRRQFIEDNAVYAN**

29 s2 100.0% 99.7%  **LDKLKQELNPSPKWSIARYKGLGEMNADQLWETTMNPEHRSMLQVRLEDAIDADQTFEMLMGDVVENRRQFIEDNAVYAN**

30 S43 100.0% 99.8%  **LDKLKQELNPSPKWSIARYKGLGEMNADQLWETTMNPEHRSMLQVRLEDAIDADQTFEMLMGDVVENRRQFIEDNAVYAN**

31 S17W 90.7% 99.7%  **LDKLKQELNPSPKWSIARYKGLGEMNADQLWETTMNPEHRSMLQVRLEDAIDADQTFEMLMGDVVENRRQFIEDNAVYAN**

32 48 100.0% 99.7%  **LDKLKQELNPSPKWSIARYKGLGEMNADQLWETTMNPEHRSMLQVRLEDAIDADQTFEMLMGDVVENRRQFIEDNAVYAN**

33 ABKVF 100.0% 99.7%  **LDKLKQELNPSPKWSIARYKGLGEMNADQLWETTMNPEHRSMLQVRLEDAIDADQTFEMLMGDVVENRRQFIEDNAVYAN**

34 CICARIA 100.0% 100.0%  **LDKLKQELNPSPKWSIARYKGLGEMNADQLWETTMNPEHRSMLQVRLEDAIDADQTFEMLMGDVVENRRQFIEDNAVYAN**

35 SKN25lux 90.7% 100.0%  **LDKLKQELNPSPKWSIARYKGLGEMNADQLWETTMNPEHRSMLQVRLEDAIDADQTFEMLMGDVVENRRQFIEDNAVYAN**

36 HMSC074F11 90.7% 99.7%  **LDKLKQELNPSPKWSIARYKGLGEMNADQLWETTMNPEHRSMLQVRLEDAIDADQTFEMLMGDVVENRRQFIEDNAVYAN**

37 HMSC068G11 100.0% 99.7%  **LDKLKQELNPSPKWSIARYKGLGEMNADQLWETTMNPEHRSMLQVRLEDAIDADQTFEMLMGDVVENRRQFIEDNAVYAN**

38 NIHLM037 90.7% 99.7%  **LDKLKQELNPSPKWSIARYKGLGEMNADQLWETTMNPEHRSMLQVRLEDAIDADQTFEMLMGDVVENRRQFIEDNAVYAN**

39 NIHLM015 90.7% 99.7%  **LDKLKQELNPSPKWSIARYKGLGEMNADQLWETTMNPEHRSMLQVRLEDAIDADQTFEMLMGDVVENRRQFIEDNAVYAN**

consensus/100%  **LDKLKQELNPSPKWSIARYKGLGEMNADQLWETTMNPEHRSMLQVRLEDAIDADQTFEMLMGDVVENRRQFIEDNAVYAN**

consensus/90%  **LDKLKQELNPSPKWSIARYKGLGEMNADQLWETTMNPEHRSMLQVRLEDAIDADQTFEMLMGDVVENRRQFIEDNAVYAN**

consensus/80%  **LDKLKQELNPSPKWSIARYKGLGEMNADQLWETTMNPEHRSMLQVRLEDAIDADQTFEMLMGDVVENRRQFIEDNAVYAN**

consensus/70%  **LDKLKQELNPSPKWSIARYKGLGEMNADQLWETTMNPEHRSMLQVRLEDAIDADQTFEMLMGDVVENRRQFIEDNAVYAN**

cov pid **641**  **]** **643**

1 ATCC35984 100.0% 100.0%  **LDF**

2 ATCC12228 90.7% 100.0%  **LDF**

3 36AM 90.7% 99.7%  **LDF**

4 48AF 100.0% 100.0%  **LDF**

5 54AF 100.0% 99.7%  **LDF**

6 785_SEPI 100.0% 99.7%  **LDF**

7 VSE49 100.0% 99.7%  **LDF**

8 VSE57 100.0% 99.7%  **LDF**

9 SE45 100.0% 99.7%  **LDF**

10 SE40 100.0% 99.7%  **LDF**

11 32A 100.0% 99.7%  **LDF**

12 HD66 100.0% 100.0%  **LDF**

13 HD43 100.0% 100.0%  **LDF**

14 HD33 100.0% 100.0%  **LDF**

15 DE0525 100.0% 99.7%  **LDF**

16 4928STDY7071543 100.0% 99.7%  **LDF**

17 BAV 100.0% 99.7%  **LDF**

18 APC 100.0% 99.7%  **LDF**

19 SESURV_p1_0557 100.0% 100.0%  **LDF**

20 SESURV_p1_0612 100.0% 99.8%  **LDF**

21 SESURV_p4_1553 100.0% 99.7%  **LDF**

22 SESURV_p1_1200 100.0% 100.0%  **LDF**

23 SESURV_p3_0825 90.7% 100.0%  **LDF**

24 JH 100.0% 99.8%  **LDF**

25 Z0118SE0132 100.0% 99.8%  **LDF**

26 R5981 100.0% 99.8%  **LDF**

27 JH-S-3 100.0% 99.8%  **LDF**

28 JH-S-1 100.0% 99.8%  **LDF**

29 s2 100.0% 99.7%  **LDF**

30 S43 100.0% 99.8%  **LDF**

31 S17W 90.7% 99.7%  **LDF**

32 48 100.0% 99.7%  **LDF**

33 ABKVF 100.0% 99.7%  **LDF**

34 CICARIA 100.0% 100.0%  **LDF**

35 SKN25lux 90.7% 100.0%  **LDF**

36 HMSC074F11 90.7% 99.7%  **LDF**

37 HMSC068G11 100.0% 99.7%  **LDF**

38 NIHLM037 90.7% 99.7%  **LDF**

39 NIHLM015 90.7% 99.7%  **LDF**

consensus/100%  **LDF**

consensus/90%  **LDF**

consensus/80%  **LDF**

consensus/70%  **LDF**

**Supplementary Figure 2.** Multiple sequence alignment of the predicted amino acid sequences of GyrB carried by *S. epidermidis* isolates and close genomes retrieved from the BV-BRC database compared to that of *S. epidermidis* strain ATCC®12228 and ATCC®35984. The alignment was visualized using MView version 1.63 hosted by the EMBL-EBI; cov, coverage; pid, percent identity.

cov pid  **1** **[ . . . . : . . .** **80**

1 ATCC35984 100.0% 100.0%  **MSEIIQDLSLEDVIGDRFGRYSKYIIQERALPDVRDGLKPVQRRILFAMYSSGNTYDKNFRKSAKTVGDVIGQYHPHGDS**

2 ATCC12228 100.0% 99.9%  **MSEIIQDLSLEDVIGDRFGRYSKYIIQERALPDVRDGLKPVQRRILFAMYSSGNTYDKNFRKSAKTVGDVIGQYHPHGDS**

3 36AM 92.5% 98.9%  **------------------------------------------------------------RKSAKTVGDVIGQYHPHGDS**

4 48AF 100.0% 99.9%  **MSEIIQDLSLEDVIGDRFGRYSKYIIQERALPDVRDGLKPVQRRILFAMYSSGNTYDKNFRKSAKTVGDVIGQYHPHGDS**

5 54AF 100.0% 99.0%  **MSEIIQDLSLEDVIGDRFGRYSKYIIQERALPDVRDGLKPVQRRILFAMYSSGNTYDKNFRKSAKTVGDVIGQYHPHGDS**

6 785_SEPI 100.0% 99.1%  **MSEIIQDLSLEDVIGDRFGRYSKYIIQERALPDVRDGLKPVQRRILFAMYSSGNTYDKNFRKSAKTVGDVIGQYHPHGDS**

7 VSE49 100.0% 99.2%  **MSEIIQDLSLEDVIGDRFGRYSKYIIQERALPDVRDGLKPVQRRILFAMYSSGNTYDKNFRKSAKTVGDVIGQYHPHGDS**

8 VSE57 100.0% 99.2%  **MSEIIQDLSLEDVIGDRFGRYSKYIIQERALPDVRDGLKPVQRRILFAMYSSGNTYDKNFRKSAKTVGDVIGQYHPHGDS**

9 SE45 100.0% 99.2%  **MSEIIQDLSLEDVIGDRFGRYSKYIIQERALPDVRDGLKPVQRRILFAMYSSGNTYDKNFRKSAKTVGDVIGQYHPHGDS**

10 SE40 100.0% 99.2%  **MSEIIQDLSLEDVIGDRFGRYSKYIIQERALPDVRDGLKPVQRRILFAMYSSGNTYDKNFRKSAKTVGDVIGQYHPHGDS**

11 32A 100.0% 97.8%  **MSEIIQDLSLEDVIGDRFGRYSKYIIQERALPDVRDGLKPVQRRILFAMYSSGNTYDKNFRKSAKTVGDVIGQYHPHGDS**

12 HD66 100.0% 99.9%  **MSEIIQDLSLEDVIGDRFGRYSKYIIQERALPDVRDGLKPVQRRILFAMYSSGNTYDKNFRKSAKTVGDVIGQYHPHGDY**

13 HD43 100.0% 99.9%  **MSEIIQDLSLEDVIGDRFGRYSKYIIQERALPDVRDGLKPVQRRILFAMYSSGNTYDKNFRKSAKTVGDVIGQYHPHGDF**

14 HD33 100.0% 99.9%  **MSEIIQDLSLEDVIGDRFGRYSKYIIQERALPDVRDGLKPVQRRILFAMYSSGNTYDKNFRKSAKTVGDVIGQYHPHGDY**

15 DE0525 100.0% 99.0%  **MSEIIQDLSLEDVIGDRFGRYSKYIIQERALPDVRDGLKPVQRRILFAMYSSGNTYDKNFRKSAKTVGDVIGQYHPHGDS**

16 4928STDY7071543 100.0% 99.0%  **MSEIIQDLSLEDVIGDRFGRYSKYIIQERALPDVRDGLKPVQRRILFAMYSSGNTYDKNFRKSAKTVGDVIGQYHPHGDS**

17 BAV2502 100.0% 99.1%  **MSEIIQDLSLEDVIGDRFGRYSKYIIQERALPDVRDGLKPVQRRILFAMYSSGNTYDKNFRKSAKTVGDVIGQYHPHGDS**

18 APC3784 100.0% 99.0%  **MSEIIQDLSLEDVIGDRFGRYSKYIIQERALPDVRDGLKPVQRRILFAMYSSGNTYDKNFRKSAKTVGDVIGQYHPHGDS**

19 SESURV_p1_0557 100.0% 99.9%  **MSEIIQDLSLEDVIGDRFGRYSKYIIQERALPDVRDGLKPVQRRILFAMYSSGNTYDKNFRKSAKTVGDVIGQYHPHGDA**

20 SESURV_p1_0612 100.0% 99.0%  **MSEIIQDLSLEDVIGDRFGRYSKYIIQERALPDVRDGLKPVQRRILFAMYSSGNTYDKNFRKSAKTVGDVIGQYHPHGDS**

21 SESURV_p4_1553 100.0% 100.0%  **MSEIIQDLSLEDVIGDRFGRYSKYIIQERALPDVRDGLKPVQRRILFAMYSSGNTYDKNFRKSAKTVGDVIGQYHPHGDS**

22 SESURV_p1_1200 100.0% 99.9%  **MSEIIQDLSLEDVIGDRFGRYSKYIIQERALPDVRDGLKPVQRRILFAMYSSGNTYDKNFRKSAKTVGDVIGQYHPHGDA**

23 SESURV_p3_0825 100.0% 99.9%  **MSEIIQDLSLEDVIGDRFGRYSKYIIQERALPDVRDGLKPVQRRILFAMYSSGNTYDKNFRKSAKTVGDVIGQYHPHGDS**

24 JH 100.0% 99.0%  **MSEIIQDLSLEDVIGDRFGRYSKYIIQERALPDVRDGLKPVQRRILFAMYSSGNTYDKNFRKSAKTVGDVIGQYHPHGDS**

25 Z0118SE0132 100.0% 98.9%  **MSEIIQDLSLEDVIGDRFGRYSKYIIQERALPDVRDGLKPVQRRILFAMYSSGNTYDKNFRKSAKTVGDVIGQYHPHGDS**

26 R5981 100.0% 98.9%  **MSEIIQDLSLEDVIGDRFGRYSKYIIQERALPDVRDGLKPVQRRILFAMYSSGNTYDKNFRKSAKTVGDVIGQYHPHGDS**

27 JH-S-3 100.0% 99.0%  **MSEIIQDLSLEDVIGDRFGRYSKYIIQERALPDVRDGLKPVQRRILFAMYSSGNTYDKNFRKSAKTVGDVIGQYHPHGDS**

28 JH-S-1 100.0% 99.0%  **MSEIIQDLSLEDVIGDRFGRYSKYIIQERALPDVRDGLKPVQRRILFAMYSSGNTYDKNFRKSAKTVGDVIGQYHPHGDS**

29 s2 100.0% 98.9%  **MSEIIQDLSLEDVIGDRFGRYSKYIIQERALPDVRDGLKPVQRRILFAMYSSGNTYDKNFRKSAKTVGDVIGQYHPHGDS**

30 S43 100.0% 99.0%  **MSEIIQDLSLEDVIGDRFGRYSKYIIQERALPDVRDGLKPVQRRILFAMYSSGNTYDKNFRKSAKTVGDVIGQYHPHGDS**

31 S17W 100.0% 99.0%  **MSEIIQDLSLEDVIGDRFGRYSKYIIQERALPDVRDGLKPVQRRILFAMYSSGNTYDKNFRKSAKTVGDVIGQYHPHGDS**

32 48 100.0% 99.1%  **MSEIIQDLSLEDVIGDRFGRYSKYIIQERALPDVRDGLKPVQRRILFAMYSSGNTYDKNFRKSAKTVGDVIGQYHPHGDS**

33 ABKVF 100.0% 99.0%  **MSEIIQDLSLEDVIGDRFGRYSKYIIQERALPDVRDGLKPVQRRILFAMYSSGNTYDKNFRKSAKTVGDVIGQYHPHGDS**

34 CICARIA 100.0% 99.9%  **MSEIIQDLSLEDVIGDRFGRYSKYIIQERALPDVRDGLKPVQRRILFAMYSSGNTYDKNFRKSAKTVGDVIGQYHPHGDS**

35 SKN25lux 100.0% 99.9%  **MSEIIQDLSLEDVIGDRFGRYSKYIIQERALPDVRDGLKPVQRRILFAMYSSGNTYDKNFRKSAKTVGDVIGQYHPHGDY**

36 HMSC074F11 100.0% 99.0%  **MSEIIQDLSLEDVIGDRFGRYSKYIIQERALPDVRDGLKPVQRRILFAMYSSGNTYDKNFRKSAKTVGDVIGQYHPHGDS**

37 HMSC068G11 100.0% 99.0%  **MSEIIQDLSLEDVIGDRFGRYSKYIIQERALPDVRDGLKPVQRRILFAMYSSGNTYDKNFRKSAKTVGDVIGQYHPHGDS**

38 NIHLM037 100.0% 99.0%  **MSEIIQDLSLEDVIGDRFGRYSKYIIQERALPDVRDGLKPVQRRILFAMYSSGNTYDKNFRKSAKTVGDVIGQYHPHGDS**

39 NIHLM015 100.0% 99.0%  **MSEIIQDLSLEDVIGDRFGRYSKYIIQERALPDVRDGLKPVQRRILFAMYSSGNTYDKNFRKSAKTVGDVIGQYHPHGDS**

consensus/100%  **............................................................RKSAKTVGDVIGQYHPHGD.**

consensus/90%  **MSEIIQDLSLEDVIGDRFGRYSKYIIQERALPDVRDGLKPVQRRILFAMYSSGNTYDKNFRKSAKTVGDVIGQYHPHGD.**

consensus/80%  **MSEIIQDLSLEDVIGDRFGRYSKYIIQERALPDVRDGLKPVQRRILFAMYSSGNTYDKNFRKSAKTVGDVIGQYHPHGDS**

consensus/70%  **MSEIIQDLSLEDVIGDRFGRYSKYIIQERALPDVRDGLKPVQRRILFAMYSSGNTYDKNFRKSAKTVGDVIGQYHPHGDS**

cov pid  **81**  **. 1 . . . . : .** **160**

1 ATCC35984 100.0% 100.0%  **SVYDAMVRLSQDWKLRHVLIEMHGNNGSIDNDPPAAMRYTEAKLSQLSEELLRDINKETVSFIPNYDDTTLEPMVLPARF**

2 ATCC12228 100.0% 99.9%  **SVYDAMVRLSQDWKLRHVLIEMHGNNGSIDNDPPAAMRYTEAKLSQLSEELLRDINKETVSFIPNYDDTTLEPMVLPARF**

3 36AM 92.5% 98.9%  **SVYDAMVRLSQDWKLRHVLIEMHGNNGSIDNDPPAAMRYTEAKLSQLSEELLRDINKETVSFIPNYDDTTLEPMVLPARF**

4 48AF 100.0% 99.9%  **SVYDAMVRLSQDWKLRHVLIEMHGNNGSIDNDPPAAMRYTEAKLSQLSEELLRDINKETVSFIPNYDDTTLEPMVLPARF**

5 54AF 100.0% 99.0%  **SVYDAMVRLSQDWKLRHVLIEMHGNNGSIDNDPPAAMRYTEAKLSQLSEELLRDINKETVSFIPNYDDTTLEPMVLPARF**

6 785_SEPI 100.0% 99.1%  **SVYDAMVRLSQDWKLRHVLIEMHGNNGSIDNDPPAAMRYTEAKLSQLSEELLRDINKETVSFIPNYDDTTLEPMVLPARF**

7 VSE49 100.0% 99.2%  **SVYDAMVRLSQDWKLRHVLIEMHGNNGSIDNDPPAAMRYTEAKLSQLSEELLRDINKETVSFIPNYDDTTLEPMVLPARF**

8 VSE57 100.0% 99.2%  **SVYDAMVRLSQDWKLRHVLIEMHGNNGSIDNDPPAAMRYTEAKLSQLSEELLRDINKETVSFIPNYDDTTLEPMVLPARF**

9 SE45 100.0% 99.2%  **SVYDAMVRLSQDWKLRHVLIEMHGNNGSIDNDPPAAMRYTEAKLSQLSEELLRDINKETVSFIPNYDDTTLEPMVLPARF**

10 SE40 100.0% 99.2%  **SVYDAMVRLSQDWKLRHVLIEMHGNNGSIDNDPPAAMRYTEAKLSQLSEELLRDINKETVSFIPNYDDTTLEPMVLPARF**

11 32A 100.0% 97.8%  **SVYDAMVRLSQDWKLRHVLIEMHGNNGSIDNDPPAAMRYTEAKLSQLSEELLRDINKETVSFIPNYDDTTLEPMVLPARF**

12 HD66 100.0% 99.9%  **SVYDAMVRLSQDWKLRHVLIEMHGNNGSIDNDPPAAMRYTEAKLSQLSEELLRDINKETVSFIPNYDDTTLEPMVLPARF**

13 HD43 100.0% 99.9%  **SVYDAMVRLSQDWKLRHVLIEMHGNNGSIDNDPPAAMRYTEAKLSQLSEELLRDINKETVSFIPNYDDTTLEPMVLPARF**

14 HD33 100.0% 99.9%  **SVYDAMVRLSQDWKLRHVLIEMHGNNGSIDNDPPAAMRYTEAKLSQLSEELLRDINKETVSFIPNYDDTTLEPMVLPARF**

15 DE0525 100.0% 99.0%  **SVYDAMVRLSQDWKLRHVLIEMHGNNGSIDNDPPAAMRYTEAKLSQLSEELLRDINKETVSFIPNYDDTTLEPMVLPARF**

16 4928STDY7071543 100.0% 99.0%  **SVYDAMVRLSQDWKLRHVLIEMHGNNGSIDNDPPAAMRYTEAKLSQLSEELLRDINKETVSFIPNYDDTTLEPMVLPARF**

17 BAV2502 100.0% 99.1%  **SVYDAMVRLSQDWKLRHVLIEMHGNNGSIDNDPPAAMRYTEAKLSQLSEELLRDINKETVSFIPNYDDTTLEPMVLPARF**

18 APC3784 100.0% 99.0%  **SVYDAMVRLSQDWKLRHVLIEMHGNNGSIDNDPPAAMRYTEAKLSQLSEELLRDINKETVSFIPNYDDTTLEPMVLPARF**

19 SESURV_p1_0557 100.0% 99.9%  **SVYDAMVRLSQDWKLRHVLIEMHGNNGSIDNDPPAAMRYTEAKLSQLSEELLRDINKETVSFIPNYDDTTLEPMVLPARF**

20 SESURV_p1_0612 100.0% 99.0%  **SVYDAMVRLSQDWKLRHVLIEMHGNNGSIDNDPPAAMRYTEAKLSQLSEELLRDINKETVSFIPNYDDTTLEPMVLPARF**

21 SESURV_p4_1553 100.0% 100.0%  **SVYDAMVRLSQDWKLRHVLIEMHGNNGSIDNDPPAAMRYTEAKLSQLSEELLRDINKETVSFIPNYDDTTLEPMVLPARF**

22 SESURV_p1_1200 100.0% 99.9%  **SVYDAMVRLSQDWKLRHVLIEMHGNNGSIDNDPPAAMRYTEAKLSQLSEELLRDINKETVSFIPNYDDTTLEPMVLPARF**

23 SESURV_p3_0825 100.0% 99.9%  **SVYDAMVRLSQDWKLRHVLIEMHGNNGSIDNDPPAAMRYTEAKLSQLSEELLRDINKETVSFIPNYDDTTLEPMVLPARF**

24 JH 100.0% 99.0%  **SVYDAMVRLSQDWKLRHVLIEMHGNNGSIDNDPPAAMRYTEAKLSQLSEELLRDINKETVSFIPNYDDTTLEPMVLPARF**

25 Z0118SE0132 100.0% 98.9%  **SVYDAMVRLSQDWKLRHVLIEMHGNNGSIDNDPPAAMRYTEAKLSQLSEELLRDINKETVSFIPNYDDTTLEPMVLPARF**

26 R5981 100.0% 98.9%  **SVYDAMVRLSQDWKLRHVLIEMHGNNGSIDNDPPAAMRYTEAKLSQLSEELLRDINKETVSFIPNYDDTTLEPMVLPARF**

27 JH-S-3 100.0% 99.0%  **SVYDAMVRLSQDWKLRHVLIEMHGNNGSIDNDPPAAMRYTEAKLSQLSEELLRDINKETVSFIPNYDDTTLEPMVLPARF**

28 JH-S-1 100.0% 99.0%  **SVYDAMVRLSQDWKLRHVLIEMHGNNGSIDNDPPAAMRYTEAKLSQLSEELLRDINKETVSFIPNYDDTTLEPMVLPARF**

29 s2 100.0% 98.9%  **SVYDAMVRLSQDWKLRHVLIEMHGNNGSIDNDPPAAMRYTEAKLSQLSEELLRDINKETVSFIPNYDDTTLEPMVLPARF**

30 S43 100.0% 99.0%  **SVYDAMVRLSQDWKLRHVLIEMHGNNGSIDNDPPAAMRYTEAKLSQLSEELLRDINKETVSFIPNYDDTTLEPMVLPARF**

31 S17W 100.0% 99.0%  **SVYDAMVRLSQDWKLRHVLIEMHGNNGSIDNDPPAAMRYTEAKLSQLSEELLRDINKETVSFIPNYDDTTLEPMVLPARF**

32 48 100.0% 99.1%  **SVYDAMVRLSQDWKLRHVLIEMHGNNGSIDNDPPAAMRYTEAKLSQLSEELLRDINKETVSFIPNYDDTTLEPMVLPARF**

33 ABKVF 100.0% 99.0%  **SVYDAMVRLSQDWKLRHVLIEMHGNNGSIDNDPPAAMRYTEAKLSQLSEELLRDINKETVSFIPNYDDTTLEPMVLPARF**

34 CICARIA 100.0% 99.9%  **SVYDAMVRLSQDWKLRHVLIEMHGNNGSIDNDPPAAMRYTEAKLSQLSEELLRDINKETVSFIPNYDDTTLEPMVLPARF**

35 SKN25lux 100.0% 99.9%  **SVYDAMVRLSQDWKLRHVLIEMHGNNGSIDNDPPAAMRYTEAKLSQLSEELLRDINKETVSFIPNYDDTTLEPMVLPARF**

36 HMSC074F11 100.0% 99.0%  **SVYDAMVRLSQDWKLRHVLIEMHGNNGSIDNDPPAAMRYTEAKLSQLSEELLRDINKETVSFIPNYDDTTLEPMVLPARF**

37 HMSC068G11 100.0% 99.0%  **SVYDAMVRLSQDWKLRHVLIEMHGNNGSIDNDPPAAMRYTEAKLSQLSEELLRDINKETVSFIPNYDDTTLEPMVLPARF**

38 NIHLM037 100.0% 99.0%  **SVYDAMVRLSQDWKLRHVLIEMHGNNGSIDNDPPAAMRYTEAKLSQLSEELLRDINKETVSFIPNYDDTTLEPMVLPARF**

39 NIHLM015 100.0% 99.0%  **SVYDAMVRLSQDWKLRHVLIEMHGNNGSIDNDPPAAMRYTEAKLSQLSEELLRDINKETVSFIPNYDDTTLEPMVLPARF**

consensus/100%  **SVYDAMVRLSQDWKLRHVLIEMHGNNGSIDNDPPAAMRYTEAKLSQLSEELLRDINKETVSFIPNYDDTTLEPMVLPARF**

consensus/90%  **SVYDAMVRLSQDWKLRHVLIEMHGNNGSIDNDPPAAMRYTEAKLSQLSEELLRDINKETVSFIPNYDDTTLEPMVLPARF**

consensus/80%  **SVYDAMVRLSQDWKLRHVLIEMHGNNGSIDNDPPAAMRYTEAKLSQLSEELLRDINKETVSFIPNYDDTTLEPMVLPARF**

consensus/70%  **SVYDAMVRLSQDWKLRHVLIEMHGNNGSIDNDPPAAMRYTEAKLSQLSEELLRDINKETVSFIPNYDDTTLEPMVLPARF**

cov pid **161**  **. . . 2 . . . .** **240**

1 ATCC35984 100.0% 100.0%  **PNLLINGSTGISSGYATDIPPHNLAEVIQGTLKYIDQPDITINQLMKYIKGPDFPTGGIIQGIEGIKKAYETGKGKVVVR**

2 ATCC12228 100.0% 99.9%  **PNLLINGSTGISSGYATDIPPHNLAEVIQGTLKYIDQPDITINQLMKYIKGPDFPTGGIIQGIEGIKKAYETGKGKVVVR**

3 36AM 92.5% 98.9%  **PNLLINGSTGISSGYATDIPPHNLAEVIQGTLKYIDQPDITINQLMKYIKGPDFPTGGIIQGIEGIKKAYETGKGRVVVR**

4 48AF 100.0% 99.9%  **PNLLINGSTGISSGYATDIPPHNLAEVIQGTLKYIDQPDITINQLMKYIKGPDFPTGGIIQGIEGIKKAYETGKGKVVVR**

5 54AF 100.0% 99.0%  **PNLLINGSTGISSGYATDIPPHNLAEVIQGTLKYIDQPDITINQLMKYIKGPDFPTGGIIQGIEGIKKAYETGKGRVVVR**

6 785_SEPI 100.0% 99.1%  **PNLLINGSTGISSGYATDIPPHNLAEVIQGTLKYIDQPDITINQLMKYIKGPDFPTGGIIQGIEGIKKAYETGKGRVVVR**

7 VSE49 100.0% 99.2%  **PNLLINGSTGISSGYATDIPPHNLAEVIQGTLKYIDQPDITINQLMKYIKGPDFPTGGIIQGIEGIKKAYETGKGRVVVR**

8 VSE57 100.0% 99.2%  **PNLLINGSTGISSGYATDIPPHNLAEVIQGTLKYIDQPDITINQLMKYIKGPDFPTGGIIQGIEGIKKAYETGKGRVVVR**

9 SE45 100.0% 99.2%  **PNLLINGSTGISSGYATDIPPHNLAEVIQGTLKYIDQPDITINQLMKYIKGPDFPTGGIIQGIEGIKKAYETGKGRVVVR**

10 SE40 100.0% 99.2%  **PNLLINGSTGISSGYATDIPPHNLAEVIQGTLKYIDQPDITINQLMKYIKGPDFPTGGIIQGIEGIKKAYETGKGRVVVR**

11 32A 100.0% 97.8%  **PNLLINGSTGISSGYATDIPPHNLAEVIQGTLKYIDQPDITINQLMKYIKGPDFPTGGIIQGIEGIKKAYETGKGRVVVR**

12 HD66 100.0% 99.9%  **PNLLINGSTGISSGYATDIPPHNLAEVIQGTLKYIDQPDITINQLMKYIKGPDFPTGGIIQGIEGIKKAYETGKGKVVVR**

13 HD43 100.0% 99.9%  **PNLLINGSTGISSGYATDIPPHNLAEVIQGTLKYIDQPDITINQLMKYIKGPDFPTGGIIQGIEGIKKAYETGKGKVVVR**

14 HD33 100.0% 99.9%  **PNLLINGSTGISSGYATDIPPHNLAEVIQGTLKYIDQPDITINQLMKYIKGPDFPTGGIIQGIEGIKKAYETGKGKVVVR**

15 DE0525 100.0% 99.0%  **PNLLINGSTGISSGYATDIPPHNLAEVIQGTLKYIDQPDITINQLMKYIKGPDFPTGGIIQGIEGIKKAYETGKGRVVVR**

16 4928STDY7071543 100.0% 99.0%  **PNLLINGSTGISSGYATDIPPHNLAEVIQGTLKYIDQPDITINQLMKYIKGPDFPTGGIIQGIEGIKKAYETGKGRVVVR**

17 BAV2502 100.0% 99.1%  **PNLLINGSTGISSGYATDIPPHNLAEVIQGTLKYIDQPDITINQLMKYIKGPDFPTGGIIQGIEGIKKAYETGKGRVVVR**

18 APC3784 100.0% 99.0%  **PNLLINGSTGISSGYATDIPPHNLAEVIQGTLKYIDQPDITINQLMKYIKGPDFPTGGIIQGIEGIKKAYETGKGRVVVR**

19 SESURV_p1_0557 100.0% 99.9%  **PNLLINGSTGISSGYATDIPPHNLAEVIQGTLKYIDQPDITINQLMKYIKGPDFPTGGIIQGIEGIKKAYETGKGKVVVR**

20 SESURV_p1_0612 100.0% 99.0%  **PNLLINGSTGISSGYATDIPPHNLAEVIQGTLKYIDQPDITINQLMKYIKGPDFPTGGIIQGIEGIKKAYETGKGRVVVR**

21 SESURV_p4_1553 100.0% 100.0%  **PNLLINGSTGISSGYATDIPPHNLAEVIQGTLKYIDQPDITINQLMKYIKGPDFPTGGIIQGIEGIKKAYETGKGKVVVR**

22 SESURV_p1_1200 100.0% 99.9%  **PNLLINGSTGISSGYATDIPPHNLAEVIQGTLKYIDQPDITINQLMKYIKGPDFPTGGIIQGIEGIKKAYETGKGKVVVR**

23 SESURV_p3_0825 100.0% 99.9%  **PNLLINGSTGISSGYATDIPPHNLAEVIQGTLKYIDQPDITINQLMKYIKGPDFPTGGIIQGIEGIKKAYETGKGKVVVR**

24 JH 100.0% 99.0%  **PNLLINGSTGISSGYATDIPPHNLAEVIQGTLKYIDQPDITINQLMKYIKGPDFPTGGIIQGIEGIKKAYETGKGRVVVR**

25 Z0118SE0132 100.0% 98.9%  **PNLLINGSTGISSGYATDIPPHNLAEVIQGTLKYIDQPDITINQLMKYIKGPDFPTGGIIQGIEGIKKAYETGKGRVVVR**

26 R5981 100.0% 98.9%  **PNLLINGSTGISSGYATDIPPHNLAEVIQGTLKYIDQPDITINQLMKYIKGPDFPTGGIIQGIEGIKKAYETGKGRVVVR**

27 JH-S-3 100.0% 99.0%  **PNLLINGSTGISSGYATDIPPHNLAEVIQGTLKYIDQPDITINQLMKYIKGPDFPTGGIIQGIEGIKKAYETGKGRVVVR**

28 JH-S-1 100.0% 99.0%  **PNLLINGSTGISSGYATDIPPHNLAEVIQGTLKYIDQPDITINQLMKYIKGPDFPTGGIIQGIEGIKKAYETGKGRVVVR**

29 s2 100.0% 98.9%  **PNLLINGSTGISSGYATDIPPHNLAEVIQGTLKYIDQPDITINQLMKYIKGPDFPTGGIIQGIEGIKKAYETGKGRVVVR**

30 S43 100.0% 99.0%  **PNLLINGSTGISSGYATDIPPHNLAEVIQGTLKYIDQPDITINQLMKYIKGPDFPTGGIIQGIEGIKKAYETGKGRVVVR**

31 S17W 100.0% 99.0%  **PNLLINGSTGISSGYATDIPPHNLAEVIQGTLKYIDQPDITINQLMKYIKGPDFPTGGIIQGIEGIKKAYETGKGRVVVR**

32 48 100.0% 99.1%  **PNLLINGSTGISSGYATDIPPHNLAEVIQGTLKYIDQPDITINQLMKYIKGPDFPTGGIIQGIEGIKKAYETGKGRVVVR**

33 ABKVF 100.0% 99.0%  **PNLLINGSTGISSGYATDIPPHNLAEVIQGTLKYIDQPDITINQLMKYIKGPDFPTGGIIQGIEGIKKAYETGKGRVVVR**

34 CICARIA 100.0% 99.9%  **PNLLINGSTGISSGYATDIPPHNLAEVIQGTLKYIDQPDITINQLMKYIKGPDFPTGGIIQGIEGIKKAYETGKGKVVVR**

35 SKN25lux 100.0% 99.9%  **PNLLINGSTGISSGYATDIPPHNLAEVIQGTLKYIDQPDITINQLMKYIKGPDFPTGGIIQGIEGIKKAYETGKGKVVVR**

36 HMSC074F11 100.0% 99.0%  **PNLLINGSTGISSGYATDIPPHNLAEVIQGTLKYIDQPDITINQLMKYIKGPDFPTGGIIQGIEGIKKAYETGKGRVVVR**

37 HMSC068G11 100.0% 99.0%  **PNLLINGSTGISSGYATDIPPHNLAEVIQGTLKYIDQPDITINQLMKYIKGPDFPTGGIIQGIEGIKKAYETGKGRVVVR**

38 NIHLM037 100.0% 99.0%  **PNLLINGSTGISSGYATDIPPHNLAEVIQGTLKYIDQPDITINQLMKYIKGPDFPTGGIIQGIEGIKKAYETGKGRVVVR**

39 NIHLM015 100.0% 99.0%  **PNLLINGSTGISSGYATDIPPHNLAEVIQGTLKYIDQPDITINQLMKYIKGPDFPTGGIIQGIEGIKKAYETGKGRVVVR**

consensus/100%  **PNLLINGSTGISSGYATDIPPHNLAEVIQGTLKYIDQPDITINQLMKYIKGPDFPTGGIIQGIEGIKKAYETGKG+VVVR**

consensus/90%  **PNLLINGSTGISSGYATDIPPHNLAEVIQGTLKYIDQPDITINQLMKYIKGPDFPTGGIIQGIEGIKKAYETGKG+VVVR**

consensus/80%  **PNLLINGSTGISSGYATDIPPHNLAEVIQGTLKYIDQPDITINQLMKYIKGPDFPTGGIIQGIEGIKKAYETGKG+VVVR**

consensus/70%  **PNLLINGSTGISSGYATDIPPHNLAEVIQGTLKYIDQPDITINQLMKYIKGPDFPTGGIIQGIEGIKKAYETGKG+VVVR**

cov pid **241**  **: . . . . 3 . .** **320**

1 ATCC35984 100.0% 100.0%  **SRVDEEPLRSGRKQLIVTEIPYEVNKSSLVKRIDELRADKKVDGIVEVRDETDRTGLRIAIELKKDANSESIKNYLYKNS**

2 ATCC12228 100.0% 99.9%  **SRVDEEPLRSGRKQLIVTEIPYEVNKSSLVKKIDELRADKKVDGIVEVRDETDRTGLRIAIELKKDANSESIKNYLYKNS**

3 36AM 92.5% 98.9%  **SRVDEEPLRSGRKQLIVTEIPYEVNKSSLVKRIDELRADKKVDGIVEVRDETDRTGLRIAIELKKDANSESIKNYLYKNS**

4 48AF 100.0% 99.9%  **SRVDEEPLRSGRKQLIVTEIPYEVNKSSLVKKIDELRADKKVDGIVEVRDETDRTGLRIAIELKKDANSESIKNYLYKNS**

5 54AF 100.0% 99.0%  **SRVDEEPLRSGLKQLIVTEIPYEVNKSSLVKRIDELRADKKVDGIVEVRDETDRTGLRIAIELKKDANSESIKNYLYKNS**

6 785_SEPI 100.0% 99.1%  **SRVDEEPLRSGRKQLIVTEIPYEVNKSSLVKRIDELRADKKVDGIVEVRDETDRTGLRIAIELKKDANSESIKNYLYKNS**

7 VSE49 100.0% 99.2%  **SRVDEEPLRSGRKQLIVTEIPYEVNKSSLVKRIDELRADKKVDGIVEVRDETDRTGLRIAIELKKDANSESIKNYLYKNS**

8 VSE57 100.0% 99.2%  **SRVDEEPLRSGRKQLIVTEIPYEVNKSSLVKRIDELRADKKVDGIVEVRDETDRTGLRIAIELKKDANSESIKNYLYKNS**

9 SE45 100.0% 99.2%  **SRVDEEPLRSGRKQLIVTEIPYEVNKSSLVKRIDELRADKKVDGIVEVRDETDRTGLRIAIELKKDANSESIKNYLYKNS**

10 SE40 100.0% 99.2%  **SRVDEEPLRSGRKQLIVTEIPYEVNKSSLVKRIDELRADKKVDGIVEVRDETDRTGLRIAIELKKDANSESIKNYLYKNS**

11 32A 100.0% 97.8%  **SRVDEEPLRSGRKQLIVTEIPYEVNKSSLVKRIDELRADKKVDGIVEVRDETDRTGLRIAIELKKDANSESIKNYLYKNS**

12 HD66 100.0% 99.9%  **SRVDEEPLRSGRKQLIVTEIPYEVNKSSLVKRIDELRADKKVDGIVEVRDETDRTGLRIAIELKKDANSESIKNYLYKNS**

13 HD43 100.0% 99.9%  **SRVDEEPLRSGRKQLIVTEIPYEVNKSSLVKRIDELRADKKVDGIVEVRDETDRTGLRIAIELKKDANSESIKNYLYKNS**

14 HD33 100.0% 99.9%  **SRVDEEPLRSGRKQLIVTEIPYEVNKSSLVKRIDELRADKKVDGIVEVRDETDRTGLRIAIELKKDANSESIKNYLYKNS**

15 DE0525 100.0% 99.0%  **SRVDEEPLRSGRKQLIVTEIPYEVNKSSLVKRIDELRADKKVDGIVEVRDETDRTGLRIAIELKKDANSESIKNYLYKNS**

16 4928STDY7071543 100.0% 99.0%  **SRVDEEPLRSGRKQLIVTEIPYEVNKSSLVKRIDELRADKKVDGIVEVRDETDRTGLRIAIELKKDANSESIKNYLYKNS**

17 BAV2502 100.0% 99.1%  **SRVDEEPLRSGRKQLIVTEIPYEVNKSSLVKRIDELRADKKVDGIVEVRDETDRTGLRIAIELKKDANSESIKNYLYKNS**

18 APC3784 100.0% 99.0%  **SRVDEEPLRSGRKQLIVTEIPYEVNKSSLVKRIDELRADKKVDGIVEVRDETDRTGLRIAIELKKDANSESIKNYLYKNS**

19 SESURV_p1_0557 100.0% 99.9%  **SRVDEEPLRSGRKQLIVTEIPYEVNKSSLVKRIDELRADKKVDGIVEVRDETDRTGLRIAIELKKDANSESIKNYLYKNS**

20 SESURV_p1_0612 100.0% 99.0%  **SRVDEEPLRSGRKQLIVTEIPYEVNKSSLVKRIDELRADKKVDGIVEVRDETDRTGLRIAIELKKDANSESIKNYLYKNS**

21 SESURV_p4_1553 100.0% 100.0%  **SRVDEEPLRSGRKQLIVTEIPYEVNKSSLVKRIDELRADKKVDGIVEVRDETDRTGLRIAIELKKDANSESIKNYLYKNS**

22 SESURV_p1_1200 100.0% 99.9%  **SRVDEEPLRSGRKQLIVTEIPYEVNKSSLVKRIDELRADKKVDGIVEVRDETDRTGLRIAIELKKDANSESIKNYLYKNS**

23 SESURV_p3_0825 100.0% 99.9%  **SRVDEESLRSGRKQLIVTEIPYEVNKSSLVKRIDELRADKKVDGIVEVRDETDRTGLRIAIELKKDANSESIKNYLYKNS**

24 JH 100.0% 99.0%  **SRVDEEPLRSGRKQLIVTEIPYEVNKSSLVKRIDELRADKKVDGIVEVRDETDRTGLRIAIELKKDANSESIKNYLYKNS**

25 Z0118SE0132 100.0% 98.9%  **SRVDEEPLRSGRKQLIVTEIPYEVNKSSLVKRIDELRADKKVDGIVEVRDETDRTGLRIAIELKKDANSESIKNYLYKNS**

26 R5981 100.0% 98.9%  **SRVDEEPLRSGRKQLIVTEIPYEVNKSSLVKRIDELRADKKVDGIVEVRDETDRTGLRIAIELKKDANSESIKNYLYKNS**

27 JH-S-3 100.0% 99.0%  **SRVDEEPLRSGRKQLIVTEIPYEVNKSSLVKRIDELRADKKVDGIVEVRDETDRTGLRIAIELKKDANSESIKNYLYKNS**

28 JH-S-1 100.0% 99.0%  **SRVDEEPLRSGRKQLIVTEIPYEVNKSSLVKRIDELRADKKVDGIVEVRDETDRTGLRIAIELKKDANSESIKNYLYKNS**

29 s2 100.0% 98.9%  **SRVDEEPLRSGRKQLIVTEIPYEVNKSSLVKRIDELRADKKVDGIVEVRDETDRTGLRIAIELKKDANSESIKNYLYKNS**

30 S43 100.0% 99.0%  **SRVDEEPLRSGRKQLIVTEIPYEVNKSSLVKRIDELRADKKVDGIVEVRDETDRTGLRIAIELKKDANSESIKNYLYKNS**

31 S17W 100.0% 99.0%  **SRVDEEPLRSGRKQLIVTEIPYEVNKSSLVKRIDELRADKKVDGIVEVRDETDRTGLRIAIELKKDANSESIKNYLYKNS**

32 48 100.0% 99.1%  **SRVDEEPLRSGRKQLIVTEIPYEVNKSSLVKRIDELRADKKVDGIVEVRDETDRTGLRIAIELKKDANSESIKNYLYKNS**

33 ABKVF 100.0% 99.0%  **SRVDEEPLRSGRKQLIVTEIPYEVNKSSLVKRIDELRADKKVDGIVEVRDETDRTGLRIAIELKKDANSESIKNYLYKNS**

34 CICARIA 100.0% 99.9%  **SRVDEEPLRSGRKQLIVTEIPYEVNKSSLVKKIDELRADKKVDGIVEVRDETDRTGLRIAIELKKDANSESIKNYLYKNS**

35 SKN25lux 100.0% 99.9%  **SRVDEEPLRSGRKQLIVTEIPYEVNKSSLVKRIDELRADKKVDGIVEVRDETDRTGLRIAIELKKDANSESIKNYLYKNS**

36 HMSC074F11 100.0% 99.0%  **SRVDEEPLRSGRKQLIVTEIPYEVNKSSLVKRIDELRADKKVDGIVEVRDETDRTGLRIAIELKKDANSESIKNYLYKNS**

37 HMSC068G11 100.0% 99.0%  **SRVDEEPLRSGRKQLIVTEIPYEVNKSSLVKRIDELRADKKVDGIVEVRDETDRTGLRIAIELKKDANSESIKNYLYKNS**

38 NIHLM037 100.0% 99.0%  **SRVDEEPLRSGRKQLIVTEIPYEVNKSSLVKRIDELRADKKVDGIVEVRDETDRTGLRIAIELKKDANSESIKNYLYKNS**

39 NIHLM015 100.0% 99.0%  **SRVDEEPLRSGRKQLIVTEIPYEVNKSSLVKRIDELRADKKVDGIVEVRDETDRTGLRIAIELKKDANSESIKNYLYKNS**

consensus/100%  **SRVDEEsLRSGhKQLIVTEIPYEVNKSSLVK+IDELRADKKVDGIVEVRDETDRTGLRIAIELKKDANSESIKNYLYKNS**

consensus/90%  **SRVDEEPLRSGRKQLIVTEIPYEVNKSSLVKRIDELRADKKVDGIVEVRDETDRTGLRIAIELKKDANSESIKNYLYKNS**

consensus/80%  **SRVDEEPLRSGRKQLIVTEIPYEVNKSSLVKRIDELRADKKVDGIVEVRDETDRTGLRIAIELKKDANSESIKNYLYKNS**

consensus/70%  **SRVDEEPLRSGRKQLIVTEIPYEVNKSSLVKRIDELRADKKVDGIVEVRDETDRTGLRIAIELKKDANSESIKNYLYKNS**

cov pid **321**  **. . : . . . . 4** **400**

1 ATCC35984 100.0% 100.0%  **DLQISYNFNMVAISEGRPKLMGLREIIESYLNHQIEVVTNRTRYDLEQAEKRMHIVEGLMKALSILDEVIALIRNSKNKK**

2 ATCC12228 100.0% 99.9%  **DLQISYNFNMVAISEGRPKLMGLREIIESYLNHQIEVVTNRTRYDLEQAEKRMHIVEGLMKALSILDEVIALIRNSKNKK**

3 36AM 92.5% 98.9%  **DLQISYNFNMVAISEGRPKLMGLREIIESYLNHQIEVVTNRTRYDLEQAEKRMHIVEGLMKALSILDEVIALIRNSKNKK**

4 48AF 100.0% 99.9%  **DLQISYNFNMVAISEGRPKLMGLREIIESYLNHQIEVVTNRTRYDLEQAEKRMHIVEGLMKALSILDEVIALIRNSKNKK**

5 54AF 100.0% 99.0%  **DLQISYNFNMVAISEGRPKLMGLREIIESYLNHQIEVVTNRTRYDLEQAEKRMHIVEGLMKALSILDEVIALIRNSKNKK**

6 785_SEPI 100.0% 99.1%  **DLQISYNFNMVAISEGRPKLMGLREIIESYLNHQIEVVTNRTRYDLEQAEKRMHIVEGLMKALSILDEVIALIRNSKNKK**

7 VSE49 100.0% 99.2%  **DLQISYNFNMVAISEGRPKLMGLREIIESYLNHQIEVVTNRTRYDLEQAEKRMHIVEGLMKALSILDEVIALIRNSKNKK**

8 VSE57 100.0% 99.2%  **DLQISYNFNMVAISEGRPKLMGLREIIESYLNHQIEVVTNRTRYDLEQAEKRMHIVEGLMKALSILDEVIALIRNSKNKK**

9 SE45 100.0% 99.2%  **DLQISYNFNMVAISEGRPKLMGLREIIESYLNHQIEVVTNRTRYDLEQAEKRMHIVEGLMKALSILDEVIALIRNSKNKK**

10 SE40 100.0% 99.2%  **DLQISYNFNMVAISEGRPKLMGLREIIESYLNHQIEVVTNRTRYDLEQAEKRMHIVEGLMKALSILDEVIALIRNSKNKK**

11 32A 100.0% 97.8%  **DLQISYNFNMVAISEGRPKLMGLREIIESYLNHQIEVVTNRTRYDLEQAEKRMHIVEGLMKALSILDEVIALIRNSKNKK**

12 HD66 100.0% 99.9%  **DLQISYNFNMVAISEGRPKLMGLREIIESYLNHQIEVVTNRTRYDLEQAEKRMHIVEGLMKALSILDEVIALIRNSKNKK**

13 HD43 100.0% 99.9%  **DLQISYNFNMVAISEGRPKLMGLREIIESYLNHQIEVVTNRTRYDLEQAEKRMHIVEGLMKALSILDEVIALIRNSKNKK**

14 HD33 100.0% 99.9%  **DLQISYNFNMVAISEGRPKLMGLREIIESYLNHQIEVVTNRTRYDLEQAEKRMHIVEGLMKALSILDEVIALIRNSKNKK**

15 DE0525 100.0% 99.0%  **DLQISYNFNMVAISEGRPKLMGLREIIESYLNHQIEVVTNRTRYDLEQAEKRMHIVEGLMKALSILDEVIALIRNSKNKK**

16 4928STDY7071543 100.0% 99.0%  **DLQISYNFNMVAISEGRPKLMGLREIIESYLNHQIEVVTNRTRYDLEQAEKRMHIVEGLMKALSILDEVIALIRNSKNKK**

17 BAV2502 100.0% 99.1%  **DLQISYNFNMVAISEGRPKLMGLREIIESYLNHQIEVVTNRTRYDLEQAEKRMHIVEGLMKALSILDEVIALIRNSKNKK**

18 APC3784 100.0% 99.0%  **DLQISYNFNMVAISEGRPKLMGLREIIESYLNHQIEVVTNRTRYDLEQAEKRMHIVEGLMKALSILDEVIALIRNSKNKK**

19 SESURV_p1_0557 100.0% 99.9%  **DLQISYNFNMVAISEGRPKLMGLREIIESYLNHQIEVVTNRTRYDLEQAEKRMHIVEGLMKALSILDEVIALIRNSKNKK**

20 SESURV_p1_0612 100.0% 99.0%  **DLQISYNFNMVAISEGRPKLMGLREIIESYLNHQIEVVTNRTRYDLEQAEKRMHIVEGLMKALSILDEVIALIRNSKNKK**

21 SESURV_p4_1553 100.0% 100.0%  **DLQISYNFNMVAISEGRPKLMGLREIIESYLNHQIEVVTNRTRYDLEQAEKRMHIVEGLMKALSILDEVIALIRNSKNKK**

22 SESURV_p1_1200 100.0% 99.9%  **DLQISYNFNMVAISEGRPKLMGLREIIESYLNHQIEVVTNRTRYDLEQAEKRMHIVEGLMKALSILDEVIALIRNSKNKK**

23 SESURV_p3_0825 100.0% 99.9%  **DLQISYNFNMVAISEGRPKLMGLREIIESYLNHQIEVVTNRTRYDLEQAEKRMHIVEGLMKALSILDEVIALIRNSKNKK**

24 JH 100.0% 99.0%  **DLQISYNFNMVAISEGRPKLMGLREIIESYLNHQIEVVTNRTRYDLEQAEKRMHIVEGLMKALSILDEVIALIRNSKNKK**

25 Z0118SE0132 100.0% 98.9%  **DLQISYNFNMVAISEGRPKLMGLREIIESYLNHQIEVVTNRTRYDLEQAEKRMHIVEGLMKALSILDEVIALIRNSKNKK**

26 R5981 100.0% 98.9%  **DLQISYNFNMVAISEGRPKLMGLREIIESYLNHQIEVVTNRTRYDLEQAEKRMHIVEGLMKALSILDEVIALIRNSKNKK**

27 JH-S-3 100.0% 99.0%  **DLQISYNFNMVAISEGRPKLMGLREIIESYLNHQIEVVTNRTRYDLEQAEKRMHIVEGLMKALSILDEVIALIRNSKNKK**

28 JH-S-1 100.0% 99.0%  **DLQISYNFNMVAISEGRPKLMGLREIIESYLNHQIEVVTNRTRYDLEQAEKRMHIVEGLMKALSILDEVIALIRNSKNKK**

29 s2 100.0% 98.9%  **DLQISYNFNMVAISEGRPKLMGLRGIIESYLNHQIEVVTNRTRYDLEQAEKRMHIVEGLMKALSILDEVIALIRNSKNKK**

30 S43 100.0% 99.0%  **DLQISYNFNMVAISEGRPKLMGLREIIESYLNHQIEVVTNRTRYDLEQAEKRMHIVEGLMKALSILDEVIALIRNSKNKK**

31 S17W 100.0% 99.0%  **DLQISYNFNMVAISEGRPKLMGLREIIESYLNHQIEVVTNRTRYDLEQAEKRMHIVEGLMKALSILDEVIALIRNSKNKK**

32 48 100.0% 99.1%  **DLQISYNFNMVAISEGRPKLMGLREIIESYLNHQIEVVTNRTRYDLEQAEKRMHIVEGLMKALSILDEVIALIRNSKNKK**

33 ABKVF 100.0% 99.0%  **DLQISYNFNMVAISEGRPKLMGLREIIESYLNHQIEVVTNRTRYDLEQAEKRMHIVEGLMKALSILDEVIALIRNSKNKK**

34 CICARIA 100.0% 99.9%  **DLQISYNFNMVAISEGRPKLMGLREIIESYLNHQIEVVTNRTRYDLEQAEKRMHIVEGLMKALSILDEVIALIRNSKNKK**

35 SKN25lux 100.0% 99.9%  **DLQISYNFNMVAISEGRPKLMGLREIIESYLNHQIEVVTNRTRYDLEQAEKRMHIVEGLMKALSILDEVIALIRNSKNKK**

36 HMSC074F11 100.0% 99.0%  **DLQISYNFNMVAISEGRPKLMGLREIIESYLNHQIEVVTNRTRYDLEQAEKRMHIVEGLMKALSILDEVIALIRNSKNKK**

37 HMSC068G11 100.0% 99.0%  **DLQISYNFNMVAISEGRPKLMGLREIIESYLNHQIEVVTNRTRYDLEQAEKRMHIVEGLMKALSILDEVIALIRNSKNKK**

38 NIHLM037 100.0% 99.0%  **DLQISYNFNMVAISEGRPKLMGLREIIESYLNHQIEVVTNRTRYDLEQAEKRMHIVEGLMKALSILDEVIALIRNSKNKK**

39 NIHLM015 100.0% 99.0%  **DLQISYNFNMVAISEGRPKLMGLREIIESYLNHQIEVVTNRTRYDLEQAEKRMHIVEGLMKALSILDEVIALIRNSKNKK**

consensus/100%  **DLQISYNFNMVAISEGRPKLMGLRtIIESYLNHQIEVVTNRTRYDLEQAEKRMHIVEGLMKALSILDEVIALIRNSKNKK**

consensus/90%  **DLQISYNFNMVAISEGRPKLMGLREIIESYLNHQIEVVTNRTRYDLEQAEKRMHIVEGLMKALSILDEVIALIRNSKNKK**

consensus/80%  **DLQISYNFNMVAISEGRPKLMGLREIIESYLNHQIEVVTNRTRYDLEQAEKRMHIVEGLMKALSILDEVIALIRNSKNKK**

consensus/70%  **DLQISYNFNMVAISEGRPKLMGLREIIESYLNHQIEVVTNRTRYDLEQAEKRMHIVEGLMKALSILDEVIALIRNSKNKK**

cov pid **401**  **. . . . : . . .** **480**

1 ATCC35984 100.0% 100.0%  **DAKDNLVAEYDFTEAQAEAIVMLQLYRLTNTDIEALKKEHEELEALIKELRNILDNHEALLAVIKDELNEIKKKFKVDRL**

2 ATCC12228 100.0% 99.9%  **DAKDNLVAEYDFTEAQAEAIVMLQLYRLTNTDIEALKKEHEELEALIKELRNILDNHEALLAVIKDELNEIKKKFKVDRL**

3 36AM 92.5% 98.9%  **DAKDNLVAEYDFTEAQAEAIVMLQLYRLTNTDIEALKKEHEELEALIKELRNILDNHEALLAVIKDELNEIKKKFKVDRL**

4 48AF 100.0% 99.9%  **DAKDNLVAEYDFTEAQAEAIVMLQLYRLTNTDIEALKKEHEELEALIKELRNILDNHEALLAVIKDELNEIKKKFKVDRL**

5 54AF 100.0% 99.0%  **DAKDNLVAEYDFTEAQAEAIVMLQLYRLTNTDIEALKKEHEELEALIKELRNILDNHEALLAVIKDELNEIKKKFKVDRL**

6 785_SEPI 100.0% 99.1%  **DAKDNLVAEYDFTEAQAEAIVMLQLYRLTNTDIEALKKEHEELEALIKELRNILDNHEALLAVIKDELNEIKKKFKVDRL**

7 VSE49 100.0% 99.2%  **DAKDNLVAEYDFTEAQAEAIVMLQLYRLTNTDIEALKKEHEELEALIKELRNILDNHEALLAVIKDELNEIKKKFKVDRL**

8 VSE57 100.0% 99.2%  **DAKDNLVAEYDFTEAQAEAIVMLQLYRLTNTDIEALKKEHEELEALIKELRNILDNHEALLAVIKDELNEIKKKFKVDRL**

9 SE45 100.0% 99.2%  **DAKDNLVAEYDFTEAQAEAIVMLQLYRLTNTDIEALKKEHEELEALIKELRNILDNHEALLAVIKDELNEIKKKFKVDRL**

10 SE40 100.0% 99.2%  **DAKDNLVAEYDFTEAQAEAIVMLQLYRLTNTDIEALKKEHEELEALIKELRNILDNHEALLAVIKDELNEIKKKFKVDRL**

11 32A 100.0% 97.8%  **DAKDNLVAEYDFTEAQAEAIVMLQLYRLTNTDIEALKKEHEELEALIKELRNILDNHEALLAVIKDELNEIKKKFKVDRL**

12 HD66 100.0% 99.9%  **DAKDNLVAEYDFTEAQAEAIVMLQLYRLTNTDIEALKKEHEELEALIKELRNILDNHEALLAVIKDELNEIKKKFKVDRL**

13 HD43 100.0% 99.9%  **DAKDNLVAEYDFTEAQAEAIVMLQLYRLTNTDIEALKKEHEELEALIKELRNILDNHEALLAVIKDELNEIKKKFKVDRL**

14 HD33 100.0% 99.9%  **DAKDNLVAEYDFTEAQAEAIVMLQLYRLTNTDIEALKKEHEELEALIKELRNILDNHEALLAVIKDELNEIKKKFKVDRL**

15 DE0525 100.0% 99.0%  **DAKDNLVAEYDFTEAQAEAIVMLQLYRLTNTDIEALKKEHEELEALIKELRNILDNHEALLAVIKDELNEIKKKFKVDRL**

16 4928STDY7071543 100.0% 99.0%  **DAKDNLVAEYDFTEAQAEAIVMLQLYRLTNTDIEALKKEHEELEALIKELRNILDNHEALLAVIKDELNEIKKKFKVDRL**

17 BAV2502 100.0% 99.1%  **DAKDNLVAEYDFTEAQAEAIVMLQLYRLTNTDIEALKKEHEELEALIKELRNILDNHEALLAVIKDELNEIKKKFKVDRL**

18 APC3784 100.0% 99.0%  **DAKDNLVAEYDFTEAQAEAIVMLQLYRLTNTDIEALKKEHEELEALIKELRNILDNHEALLAVIKDELNEIKKKFKVDRL**

19 SESURV_p1_0557 100.0% 99.9%  **DAKDNLVAEYDFTEAQAEAIVMLQLYRLTNTDIEALKKEHEELEALIKELRNILDNHEALLAVIKDELNEIKKKFKVDRL**

20 SESURV_p1_0612 100.0% 99.0%  **DAKDNLVAEYDFTEAQAEAIVMLQLYRLTNTDIEALKKEHEELEALIKELRNILDNHEALLAVIKDELNEIKKKFKVDRL**

21 SESURV_p4_1553 100.0% 100.0%  **DAKDNLVAEYDFTEAQAEAIVMLQLYRLTNTDIEALKKEHEELEALIKELRNILDNHEALLAVIKDELNEIKKKFKVDRL**

22 SESURV_p1_1200 100.0% 99.9%  **DAKDNLVAEYDFTEAQAEAIVMLQLYRLTNTDIEALKKEHEELEALIKELRNILDNHEALLAVIKDELNEIKKKFKVDRL**

23 SESURV_p3_0825 100.0% 99.9%  **DAKDNLVAEYDFTEAQAEAIVMLQLYRLTNTDIEALKKEHEELEALIKELRNILDNHEALLAVIKDELNEIKKKFKVDRL**

24 JH 100.0% 99.0%  **DAKDNLVAEYDFTEAQAEAIVMLQLYRLTNTDIEALKKEHEELEALIKELRNILDNHEALLAVIKDELNEIKKKFKVDRL**

25 Z0118SE0132 100.0% 98.9%  **DAKDNLVAEYGFTEAQAEAIVMLQLYRLTNTDIEALKKEHEELEALIKELRNILDNHEALLAVIKDELNEIKKKFKVDRL**

26 R5981 100.0% 98.9%  **DAKDNLVAEYGFTEAQAEAIVMLQLYRLTNTDIEALKKEHEELEALIKELRNILDNHEALLAVIKDELNEIKKKFKVDRL**

27 JH-S-3 100.0% 99.0%  **DAKDNLVAEYDFTEAQAEAIVMLQLYRLTNTDIEALKKEHEELEALIKELRNILDNHEALLAVIKDELNEIKKKFKVDRL**

28 JH-S-1 100.0% 99.0%  **DAKDNLVAEYDFTEAQAEAIVMLQLYRLTNTDIEALKKEHEELEALIKELRNILDNHEALLAVIKDELNEIKKKFKVDRL**

29 s2 100.0% 98.9%  **DAKDNLVAEYDFTEAQAEAIVMLQLYRLTNTDIEALKKEHEELEALIKELRNILDNHEALLAVIKDELNEIKKKFKVDRL**

30 S43 100.0% 99.0%  **DAKDNLVAEYDFTEAQAEAIVMLQLYRLTNTDIEALKKEHEELEALIKELRNILDNPEALLAVIKDELNEIKKKFKVDRL**

31 S17W 100.0% 99.0%  **DAKDNLVAEYDFTEAQAEAIVMLQLYRLTNTDIEALKKEHEELEALIKELRNILDNHEALLAVIKDELNEIKKKFKVDRL**

32 48 100.0% 99.1%  **DAKDNLVAEYDFTEAQAEAIVMLQLYRLTNTDIEALKKEHEELEALIKELRNILDNHEALLAVIKDELNEIKKKFKVDRL**

33 ABKVF 100.0% 99.0%  **DAKDNLVAEYDFTEAQAEAIVMLQLYRLTNTDIEALKKEHEELEALIKELRNILDNHEALLAVIKDELNEIKKKFKVDRL**

34 CICARIA 100.0% 99.9%  **DAKDNLVAEYDFTEAQAEAIVMLQLYRLTNTDIEALKKEHEELEALIKELRNILDNHEALLAVIKDELNEIKKKFKVDRL**

35 SKN25lux 100.0% 99.9%  **DAKDNLVAEYDFTEAQAEAIVMLQLYRLTNTDIEALKKEHEELEALIKELRNILDNHEALLAVIKDELNEIKKKFKVDRL**

36 HMSC074F11 100.0% 99.0%  **DAKDNLVAEYDFTEAQAEAIVMLQLYRLTNTDIEALKKEHEELEALIKELRNILDNHEALLAVIKDELNEIKKKFKVDRL**

37 HMSC068G11 100.0% 99.0%  **DAKDNLVAEYDFTEAQAEAIVMLQLYRLTNTDIEALKKEHEELEALIKELRNILDNHEALLAVIKDELNEIKKKFKVDRL**

38 NIHLM037 100.0% 99.0%  **DAKDNLVAEYDFTEAQAEAIVMLQLYRLTNTDIEALKKEHEELEALIKELRNILDNHEALLAVIKDELNEIKKKFKVDRL**

39 NIHLM015 100.0% 99.0%  **DAKDNLVAEYDFTEAQAEAIVMLQLYRLTNTDIEALKKEHEELEALIKELRNILDNHEALLAVIKDELNEIKKKFKVDRL**

consensus/100%  **DAKDNLVAEYsFTEAQAEAIVMLQLYRLTNTDIEALKKEHEELEALIKELRNILDN.EALLAVIKDELNEIKKKFKVDRL**

consensus/90%  **DAKDNLVAEYDFTEAQAEAIVMLQLYRLTNTDIEALKKEHEELEALIKELRNILDNHEALLAVIKDELNEIKKKFKVDRL**

consensus/80%  **DAKDNLVAEYDFTEAQAEAIVMLQLYRLTNTDIEALKKEHEELEALIKELRNILDNHEALLAVIKDELNEIKKKFKVDRL**

consensus/70%  **DAKDNLVAEYDFTEAQAEAIVMLQLYRLTNTDIEALKKEHEELEALIKELRNILDNHEALLAVIKDELNEIKKKFKVDRL**

cov pid **481**  **. 5 . . . . : .** **560**

1 ATCC35984 100.0% 100.0%  **STIEAEISEIKIDKEVMVPSEEVILSLTQHGYIKRTSTRSFNASGVTEIGLKDGDRLLKHESVNTQDTVLVFTNKGRYLF**

2 ATCC12228 100.0% 99.9%  **STIEAEISEIKIDKEVMVPSEEVILSLTQHGYIKRTSTRSFNASGVTEIGLKDGDRLLKHESVNTQDTVLVFTNKGRYLF**

3 36AM 92.5% 98.9%  **STIEAEISEIKIDKEVMVPSEEVILSLTQHGYIKRTSTRSFNASGVTEIGLKDGDRLLKHEAVNTQDTVLVFTNKGRYLF**

4 48AF 100.0% 99.9%  **STIEAEISEIKIDKEVMVPSEEVILSLTQHGYIKRTSTRSFNASGVTEIGLKDGDRLLKHESVNTQDTVLVFTNKGRYLF**

5 54AF 100.0% 99.0%  **STIEAEISEIKIDKEVMVPSEEVILSLTQHGYIKRTSTRSFNASGVTEIGLKDGDRLLKHEAVNTQDTVLVFTNKGRYLF**

6 785_SEPI 100.0% 99.1%  **STIEAEISEIKIDKEVMVPSEEVILSLTQHGYIKRTSTRSFNASGVTEIGLKDGDRLLKHEAVNTQDTVLVFTNKGRYLF**

7 VSE49 100.0% 99.2%  **STIEAEISEIKIDKEVMVPSEEVILSLTQHGYIKRTSTRSFNASGVTEIGLKDGDRLLKHESVNTQDTVLVFTNKGRYLF**

8 VSE57 100.0% 99.2%  **STIEAEISEIKIDKEVMVPSEEVILSLTQHGYIKRTSTRSFNASGVTEIGLKDGDRLLKHESVNTQDTVLVFTNKGRYLF**

9 SE45 100.0% 99.2%  **STIEAEISEIKIDKEVMVPSEEVILSLTQHGYIKRTSTRSFNASGVTEIGLKDGDRLLKHESVNTQDTVLVFTNKGRYLF**

10 SE40 100.0% 99.2%  **STIEAEISEIKIDKEVMVPSEEVILSLTQHGYIKRTSTRSFNASGVTEIGLKDGDRLLKHESVNTQDTVLVFTNKGRYLF**

11 32A 100.0% 97.8%  **STIEAEISEIKIDKEVMVPSEEVILSLTQHGYIKRTSTRSFNASGVTEIGLKDGDRLLKHEAVNTQDTVLVFTNKGRYLF**

12 HD66 100.0% 99.9%  **STIEAEISEIKIDKEVMVPSEEVILSLTQHGYIKRTSTRSFNASGVTEIGLKDGDRLLKHESVNTQDTVLVFTNKGRYLF**

13 HD43 100.0% 99.9%  **STIEAEISEIKIDKEVMVPSEEVILSLTQHGYIKRTSTRSFNASGVTEIGLKDGDRLLKHESVNTQDTVLVFTNKGRYLF**

14 HD33 100.0% 99.9%  **STIEAEISEIKIDKEVMVPSEEVILSLTQHGYIKRTSTRSFNASGVTEIGLKDGDRLLKHESVNTQDTVLVFTNKGRYLF**

15 DE0525 100.0% 99.0%  **STIEAEISEIKIDKEVMVPSEEVILSLTQHGYIKRTSTRSFNASGVTEIGLKDGDRLLKHEAVNTQDTVLVFTNKGRYLF**

16 4928STDY7071543 100.0% 99.0%  **STIEAEISEIKIDKEVMVPSEEVILSLTQHGYIKRTSTRSFNASGVTEIGLKDGDRLLKHEAVNTQDTVLVFTNKGRYLF**

17 BAV2502 100.0% 99.1%  **STIEAEISEIKIDKEVMVPSEEVILSLTQHGYIKRTSTRSFNASGVTEIGLKDGDRLLKHEAVNTQDTVLVFTNKGRYLF**

18 APC3784 100.0% 99.0%  **STIEAEISEIKIDKEVMVPSEEVILSLTQHGYIKRTSTRSFNASGVTEIGLKDGDRLLKHEAVNTQDTVLVFTNKGRYLF**

19 SESURV_p1_0557 100.0% 99.9%  **STIEAEISEIKIDKEVMVPSEEVILSLTQHGYIKRTSTRSFNASGVTEIGLKDGDRLLKHESVNTQDTVLVFTNKGRYLF**

20 SESURV_p1_0612 100.0% 99.0%  **STIEAEISEIKIDKEVMVPSEEVILSLTQHGYIKRTSTRSFNASGVTEIGLKDGDRLLKHEAVNTQDTVLVFTNKGRYLF**

21 SESURV_p4_1553 100.0% 100.0%  **STIEAEISEIKIDKEVMVPSEEVILSLTQHGYIKRTSTRSFNASGVTEIGLKDGDRLLKHESVNTQDTVLVFTNKGRYLF**

22 SESURV_p1_1200 100.0% 99.9%  **STIEAEISEIKIDKEVMVPSEEVILSLTQHGYIKRTSTRSFNASGVTEIGLKDGDRLLKHESVNTQDTVLVFTNKGRYLF**

23 SESURV_p3_0825 100.0% 99.9%  **STIEAEISEIKIDKEVMVPSEEVILSLTQHGYIKRTSTRSFNASGVTEIGLKDGDRLLKHESVNTQDTVLVFTNKGRYLF**

24 JH 100.0% 99.0%  **STIEAEISEIKIDKEVMVPSEEVILSLTQHGYIKRTSTRSFNASGVTEIGLKDGDRLLKHEAVNTQDTVLVFTNKGRYLF**

25 Z0118SE0132 100.0% 98.9%  **STIEAEISEIKIDKEVMVPSEEVILSLTQHGYIKRTSTRSFNASGVTEIGLKDGDRLLKHEAVNTQDTVLVFTNKGRYLF**

26 R5981 100.0% 98.9%  **STIEAEISEIKIDKEVMVPSEEVILSLTQHGYIKRTSTRSFNASGVTEIGLKDGDRLLKHEAVNTQDTVLVFTNKGRYLF**

27 JH-S-3 100.0% 99.0%  **STIEAEISEIKIDKEVMVPSEEVILSLTQHGYIKRTSTRSFNASGVTEIGLKDGDRLLKHEAVNTQDTVLVFTNKGRYLF**

28 JH-S-1 100.0% 99.0%  **STIEAEISEIKIDKEVMVPSEEVILSLTQHGYIKRTSTRSFNASGVTEIGLKDGDRLLKHEAVNTQDTVLVFTNKGRYLF**

29 s2 100.0% 98.9%  **STIEAEISEIKIDKEVMVPSEEVILSLTQHGYIKRTSTRSFNASGVTEIGLKDGDRLLKHEAVNTQDTVLVFTNKGRYLF**

30 S43 100.0% 99.0%  **STIEAEISEIKIDKEVMVPSEEVILSLTQHGYIKRTSTRSFNASGVTEIGLKDGDRLLKHEAVNTQDTVLVFTNKGRYLF**

31 S17W 100.0% 99.0%  **STIEAEISEIKIDKEVMVPSEEVILSLTQHGYIKRTSTRSFNASGVTEIGLKDGDRLLKHEAVNTQDTVLVFTNKGRYLF**

32 48 100.0% 99.1%  **STIEAEISEIKIDKEVMVPSEEVILSLTQHGYIKRTSTRSFNASGVTEIGLKDGDRLLKHEAVNTQDTVLVFTNKGRYLF**

33 ABKVF 100.0% 99.0%  **STIEAEISEIKIDKEVMVPSEEVILSLTQHGYIKRTSTRSFNASGVTEIGLKDGDRLLKHEAVNTQDTVLVFTNKGRYLF**

34 CICARIA 100.0% 99.9%  **STIEAEISEIKIDKEVMVPSEEVILSLTQHGYIKRTSTRSFNASGVTEIGLKDGDRLLKHESVNTQDTVLVFTNKGRYLF**

35 SKN25lux 100.0% 99.9%  **STIEAEISEIKIDKEVMVPSEEVILSLTQHGYIKRTSTRSFNASGVTEIGLKDGDRLLKHESVNTQDTVLVFTNKGRYLF**

36 HMSC074F11 100.0% 99.0%  **STIEAEISEIKIDKEVMVPSEEVILSLTQHGYIKRTSTRSFNASGVTEIGLKDGDRLLKHEAVNTQDTVLVFTNKGRYLF**

37 HMSC068G11 100.0% 99.0%  **STIEAEISEIKIDKEVMVPSEEVILSLTQHGYIKRTSTRSFNASGVTEIGLKDGDRLLKHEAVNTQDTVLVFTNKGRYLF**

38 NIHLM037 100.0% 99.0%  **STIEAEISEIKIDKEVMVPSEEVILSLTQHGYIKRTSTRSFNASGVTEIGLKDGDRLLKHEAVNTQDTVLVFTNKGRYLF**

39 NIHLM015 100.0% 99.0%  **STIEAEISEIKIDKEVMVPSEEVILSLTQHGYIKRTSTRSFNASGVTEIGLKDGDRLLKHEAVNTQDTVLVFTNKGRYLF**

consensus/100%  **STIEAEISEIKIDKEVMVPSEEVILSLTQHGYIKRTSTRSFNASGVTEIGLKDGDRLLKHEuVNTQDTVLVFTNKGRYLF**

consensus/90%  **STIEAEISEIKIDKEVMVPSEEVILSLTQHGYIKRTSTRSFNASGVTEIGLKDGDRLLKHEuVNTQDTVLVFTNKGRYLF**

consensus/80%  **STIEAEISEIKIDKEVMVPSEEVILSLTQHGYIKRTSTRSFNASGVTEIGLKDGDRLLKHEuVNTQDTVLVFTNKGRYLF**

consensus/70%  **STIEAEISEIKIDKEVMVPSEEVILSLTQHGYIKRTSTRSFNASGVTEIGLKDGDRLLKHEuVNTQDTVLVFTNKGRYLF**

cov pid **561**  **. . . 6 . . . .** **640**

1 ATCC35984 100.0% 100.0%  **IPVHKLADIRWKELGQHISQIVPIDEDEEVVNVYNEKDFKNEAFYIMATKNGMIKKSSASQFKTTRFNKPLINMKVKDKD**

2 ATCC12228 100.0% 99.9%  **IPVHKLADIRWKELGQHISQIVPIDEDEEVVNVYNEKDFKNEAFYIMATKNGMIKKSSASQFKTTRFNKPLINMKVKDKD**

3 36AM 92.5% 98.9%  **IPVHKLADIRWKELGQHISQIVPIDEDEEVVNVYNEKDFKHEAFYIMATKNGMIKKSSASQFKTTRFNKPLINMKVKDND**

4 48AF 100.0% 99.9%  **IPVHKLADIRWKELGQHISQIVPIDEDEEVVNVYNEKDFKNEAFYIMATKNGMIKKSSASQFKTTRFNKPLINMKVKDKD**

5 54AF 100.0% 99.0%  **IPVHKLADIRWKELGQHISQIVPIDEDEEVVNVYNEKDFKNEAFYIMATKNGMIKKSSASQFKTTRFNKPLINMKVKDND**

6 785_SEPI 100.0% 99.1%  **IPVHKLADIRWKELGQHISQIVPIDEDEEVVNVYNEKDFKNEAFYIMATKNGMIKKSSASQFKTTRFNKPLINMKVKDND**

7 VSE49 100.0% 99.2%  **IPVHKLADIRWKELGQHISQIVPIDEDEEVVNVYNEKDFKNEAFYIMATKNGMIKKSSASQFKTTRFNKPLINMKVKDND**

8 VSE57 100.0% 99.2%  **IPVHKLADIRWKELGQHISQIVPIDEDEEVVNVYNEKDFKNEAFYIMATKNGMIKKSSASQFKTTRFNKPLINMKVKDND**

9 SE45 100.0% 99.2%  **IPVHKLADIRWKELGQHISQIVPIDEDEEVVNVYNEKDFKNEAFYIMATKNGMIKKSSASQFKTTRFNKPLINMKVKDND**

10 SE40 100.0% 99.2%  **IPVHKLADIRWKELGQHISQIVPIDEDEEVVNVYNEKDFKNEAFYIMATKNGMIKKSSASQFKTTRFNKPLINMKVKDND**

11 32A 100.0% 97.8%  **IPVHKLADIRWKELGQHISQIVPIDEDEEVVNVYNEKDFKHEAFYIMATKNGMIKKSSASQFKTTRFNKPLINMKVKDND**

12 HD66 100.0% 99.9%  **IPVHKLADIRWKELGQHISQIVPIDEDEEVVNVYNEKDFKNEAFYIMATKNGMIKKSSASQFKTTRFNKPLINMKVKDKD**

13 HD43 100.0% 99.9%  **IPVHKLADIRWKELGQHISQIVPIDEDEEVVNVYNEKDFKNEAFYIMATKNGMIKKSSASQFKTTRFNKPLINMKVKDKD**

14 HD33 100.0% 99.9%  **IPVHKLADIRWKELGQHISQIVPIDEDEEVVNVYNEKDFKNEAFYIMATKNGMIKKSSASQFKTTRFNKPLINMKVKDKD**

15 DE0525 100.0% 99.0%  **IPVHKLADIRWKELGQHISQIVPIDEDEEVVNVYNEKDFKHEAFYIMATKNGMIKKSSASQFKTTRFNKPLINMKVKDND**

16 4928STDY7071543 100.0% 99.0%  **IPVHKLADIRWKELGQHISQIVPIDEDEEVVNVYNEKDFKHEAFYIMATKNGMIKKSSASQFKTTRFNKPLINMKVKDND**

17 BAV2502 100.0% 99.1%  **IPVHKLADIRWKELGQHISQIVPIDEDEEVVNVYNEKDFKNEAFYIMATKNGMIKKSSASQFKTTRFNKPLINMKVKDND**

18 APC3784 100.0% 99.0%  **IPVHKLADIRWKELGQHISQIVPIDEDEEVVNVYNEKDFKHEAFYIMATKNGMIKKSSASQFKTTRFNKPLINMKVKDND**

19 SESURV_p1_0557 100.0% 99.9%  **IPVHKLADIRWKELGQHISQIVPIDEDEEVVNVYNEKDFKNEAFYIMATKNGMIKKSSASQFKTTRFNKPLINMKVKDKD**

20 SESURV_p1_0612 100.0% 99.0%  **IPVHKLADIRWKELGQHISQIVPIDEDEEVVNVYNEKDFKHEAFYIMATKNGMIKKSSASQFKTTRFNKPLINMKVKDND**

21 SESURV_p4_1553 100.0% 100.0%  **IPVHKLADIRWKELGQHISQIVPIDEDEEVVNVYNEKDFKNEAFYIMATKNGMIKKSSASQFKTTRFNKPLINMKVKDKD**

22 SESURV_p1_1200 100.0% 99.9%  **IPVHKLADIRWKELGQHISQIVPIDEDEEVVNVYNEKDFKNEAFYIMATKNGMIKKSSASQFKTTRFNKPLINMKVKDKD**

23 SESURV_p3_0825 100.0% 99.9%  **IPVHKLADIRWKELGQHISQIVPIDEDEEVVNVYNEKDFKNEAFYIMATKNGMIKKSSASQFKTTRFNKPLINMKVKDKD**

24 JH 100.0% 99.0%  **IPVHKLADIRWKELGQHISQIVPIDEDEEVVNVYNEKDFKHEAFYIMATKNGMIKKSSASQFKTTRFNKPLINMKVKDND**

25 Z0118SE0132 100.0% 98.9%  **IPVHKLADIRWKELGQHISQIVPIDEDEEVVNVYNEKDFKHEAFYIMATKNGMIKKSSASQFKTTRFNKPLINMKVKDND**

26 R5981 100.0% 98.9%  **IPVHKLADIRWKELGQHISQIVPIDEDEEVVNVYNEKDFKNGAFYIMATKNGMIKKSSASQFKTTRFNKPLINMKVKDND**

27 JH-S-3 100.0% 99.0%  **IPVHKLADIRWKELGQHISQIVPIDEDEEVVNVYNEKDFKHEAFYIMATKNGMIKKSSASQFKTTRFNKPLINMKVKDND**

28 JH-S-1 100.0% 99.0%  **IPVHKLADIRWKELGQHISQIVPIDEDEEVVNVYNEKDFKHEAFYIMATKNGMIKKSSASQFKTTRFNKPLINMKVKDND**

29 s2 100.0% 98.9%  **IPVHKLADIRWKELGQHISQIVPIDEDEEVVNVYNEKDFKHEAFYIMATKNGMIKKSSASQFKTTRFNKPLINMKVKDND**

30 S43 100.0% 99.0%  **IPVHKLADIRWKELGQHISQIVPIDEDEEVVNVYNEKDFKNEAFYIMATKNGMIKKSSASQFKTTRFNKPLINMKVKDND**

31 S17W 100.0% 99.0%  **IPVHKLADIRWKELGQHISQIVPIDEDEEVVNVYNEKDFKHEAFYIMATKNGMIKKSSASQFKTTRFNKPLINMKVKDND**

32 48 100.0% 99.1%  **IPVHKLADIRWKELGQHISQIVPIDEDEEVVNVYNEKDFKNEAFYIMATKNGMIKKSSASQFKTTRFNKPLINMKVKDND**

33 ABKVF 100.0% 99.0%  **IPVHKLADIRWKELGQHISQIVPIDEDEEVVNVYNEKDFKHEAFYIMATKNGMIKKSSASQFKTTRFNKPLINMKVKDND**

34 CICARIA 100.0% 99.9%  **IPVHKLADIRWKELGQHISQIVPIDEDEEVVNVYNEKDFKNEAFYIMATKNGMIKKSSASQFKTTRFNKPLINMKVKDKD**

35 SKN25lux 100.0% 99.9%  **IPVHKLADIRWKELGQHISQIVPIDEDEEVVNVYNEKDFKNEAFYIMATKNGMIKKSSASQFKTTRFNKPLINMKVKDKD**

36 HMSC074F11 100.0% 99.0%  **IPVHKLADIRWKELGQHISQIVPIDEDEEVVNVYNEKDFKHEAFYIMATKNGMIKKSSASQFKTTRFNKPLINMKVKDND**

37 HMSC068G11 100.0% 99.0%  **IPVHKLADIRWKELGQHISQIVPIDEDEEVVNVYNEKDFKHEAFYIMATKNGMIKKSSASQFKTTRFNKPLINMKVKDND**

38 NIHLM037 100.0% 99.0%  **IPVHKLADIRWKELGQHISQIVPIDEDEEVVNVYNEKDFKHEAFYIMATKNGMIKKSSASQFKTTRFNKPLINMKVKDND**

39 NIHLM015 100.0% 99.0%  **IPVHKLADIRWKELGQHISQIVPIDEDEEVVNVYNEKDFKHEAFYIMATKNGMIKKSSASQFKTTRFNKPLINMKVKDND**

consensus/100%  **IPVHKLADIRWKELGQHISQIVPIDEDEEVVNVYNEKDFKptAFYIMATKNGMIKKSSASQFKTTRFNKPLINMKVKDpD**

consensus/90%  **IPVHKLADIRWKELGQHISQIVPIDEDEEVVNVYNEKDFKpEAFYIMATKNGMIKKSSASQFKTTRFNKPLINMKVKDpD**

consensus/80%  **IPVHKLADIRWKELGQHISQIVPIDEDEEVVNVYNEKDFKpEAFYIMATKNGMIKKSSASQFKTTRFNKPLINMKVKDpD**

consensus/70%  **IPVHKLADIRWKELGQHISQIVPIDEDEEVVNVYNEKDFKpEAFYIMATKNGMIKKSSASQFKTTRFNKPLINMKVKDpD**

cov pid **641**  **: . . . . 7 . .** **720**

1 ATCC35984 100.0% 100.0%  **ELINVVRLESDQLITVLTHKGMSLTYSTNELSDTGLRAAGVKSINLKDEDYVVMTEDVNDSDSIIMVTQRGAMKRIDFNV**

2 ATCC12228 100.0% 99.9%  **ELINVVRLESDQLITVLTHKGMSLTYSTNELSDTGLRAAGVKSINLKDEDYVVMTEDVNDSDSIIMVTQRGAMKRIDFNV**

3 36AM 92.5% 98.9%  **KLINVVRLETDQLITVLTHKGMSLTYSTNELSDTGLRAAGVKSINLKDEDYVVMTEDVNDSDSIIMVTQRGAMKRIDFNV**

4 48AF 100.0% 99.9%  **ELINVVRLESDQLITVLTHKGMSLTYSTNELSDTGLRAAGVKSINLKDEDYVVMTEDVNDSDSIIMVTQRGAMKRIDFNV**

5 54AF 100.0% 99.0%  **KLINVVRLETDQLITVLTHKGMSLTYSTNELSDTGLRAAGVKSINLKDEDYVVMTEDVNDSDSIIMVTQRGAMKRIDFNV**

6 785_SEPI 100.0% 99.1%  **KLINVVRLETDQLITVLTHKGMSLTYSTNELSDTGLRAAGVKSINLKDEDYVVMTEDVNDSDSIIMVTQRGAMKRIDFNV**

7 VSE49 100.0% 99.2%  **KLINVVRLETDQLITVLTHKGMSLTYSTNELSDTGLRAAGVKSINLKDEDYVVMTEDVNDSDSIIMVTQRGAMKRIDFNV**

8 VSE57 100.0% 99.2%  **KLINVVRLETDQLITVLTHKGMSLTYSTNELSDTGLRAAGVKSINLKDEDYVVMTEDVNDSDSIIMVTQRGAMKRIDFNV**

9 SE45 100.0% 99.2%  **KLINVVRLETDQLITVLTHKGMSLTYSTNELSDTGLRAAGVKSINLKDEDYVVMTEDVNDSDSIIMVTQRGAMKRIDFNV**

10 SE40 100.0% 99.2%  **KLINVVRLETDQLITVLTHKGMSLTYSTNELSDTGLRAAGVKSINLKDEDYVVMTEDVNDSDSIIMVTQRGAMKRIDFNV**

11 32A 100.0% 97.8%  **KLINVVRLETDQLITVLTHKGMSLTYSTNELSDTGLRAAGVKSINLKDEDYVVMTEDVNDSDSIIMVTQRGAMKRIDFNV**

12 HD66 100.0% 99.9%  **ELINVVRLESDQLITVLTHKGMSLTYSTNELSDTGLRAAGVKSINLKDEDYVVMTEDVNDSDSIIMVTQRGAMKRIDFNV**

13 HD43 100.0% 99.9%  **ELINVVRLESDQLITVLTHKGMSLTYSTNELSDTGLRAAGVKSINLKDEDYVVMTEDVNDSDSIIMVTQRGAMKRIDFNV**

14 HD33 100.0% 99.9%  **ELINVVRLESDQLITVLTHKGMSLTYSTNELSDTGLRAAGVKSINLKDEDYVVMTEDVNDSDSIIMVTQRGAMKRIDFNV**

15 DE0525 100.0% 99.0%  **KLINVVRLETDQLITVLTHKGMSLTYSTNELSDTGLRAAGVKSINLKDEDYVVMTEDVNDSDSIIMVTQRGAMKRIDFNV**

16 4928STDY7071543 100.0% 99.0%  **KLINVVRLETDQLITVLTHKGMSLTYSTNELSDTGLRAAGVKSINLKDEDYVVMTEDVNDSDSIIMVTQRGAMKRIDFNV**

17 BAV2502 100.0% 99.1%  **KLINVVRLETDQLITVLTHKGMSLTYSTNELSDTGLRAAGVKSINLKDEDYVVMTEDVNDSDSIIMVTQRGAMKRIDFNV**

18 APC3784 100.0% 99.0%  **KLINVVRLETDQLITVLTHKGMSLTYSTNELSDTGLRAAGVKSINLKDEDYVVMTEDVNDSDSIIMVTQRGAMKRIDFNV**

19 SESURV_p1_0557 100.0% 99.9%  **ELINVVRLESDQLITVLTHKGMSLTYSTNELSDTGLRAAGVKSINLKDEDYVVMTEDVNDSDSIIMVTQRGAMKRIDFNV**

20 SESURV_p1_0612 100.0% 99.0%  **KLINVVRLETDQLITVLTHKGMSLTYSTNELSDTGLRAAGVKSINLKDEDYVVMTEDVNDSDSIIMVTQRGAMKRIDFNV**

21 SESURV_p4_1553 100.0% 100.0%  **ELINVVRLESDQLITVLTHKGMSLTYSTNELSDTGLRAAGVKSINLKDEDYVVMTEDVNDSDSIIMVTQRGAMKRIDFNV**

22 SESURV_p1_1200 100.0% 99.9%  **ELINVVRLESDQLITVLTHKGMSLTYSTNELSDTGLRAAGVKSINLKDEDYVVMTEDVNDSDSIIMVTQRGAMKRIDFNV**

23 SESURV_p3_0825 100.0% 99.9%  **ELINVVRLESDQLITVLTHKGMSLTYSTNELSDTGLRAAGVKSINLKDEDYVVMTEDVNDSDSIIMVTQRGAMKRIDFNV**

24 JH 100.0% 99.0%  **KLINVVRLETDQLITVLTHKGMSLTYSTNELSDTGLRAAGVKSINLKDEDYVVMTEDVNDSDSIIMVTQRGAMKRIDFNV**

25 Z0118SE0132 100.0% 98.9%  **KLINVVRLETDQLITVLTHKGMSLTYSTNELSDTGLRAAGVKSINLKDEDYVVMTEDVNDSDSIIMVTQRGAMKRIDFNV**

26 R5981 100.0% 98.9%  **KLINVVRLETDQLITVLTHKGMSLTYSTNELSDTGLRAAGVKSINLKDEDYVVMTEDVNDSDSIIMVTQRGAMKRIDFNV**

27 JH-S-3 100.0% 99.0%  **KLINVVRLETDQLITVLTHKGMSLTYSTNELSDTGLRAAGVKSINLKDEDYVVMTEDVNDSDSIIMVTQRGAMKRIDFNV**

28 JH-S-1 100.0% 99.0%  **KLINVVRLETDQLITVLTHKGMSLTYSTNELSDTGLRAAGVKSINLKDEDYVVMTEDVNDSDSIIMVTQRGAMKRIDFNV**

29 s2 100.0% 98.9%  **KLINVVRLETDQLITVLTHKGMSLTYSTNELSDTGLRAAGVKSINLKDEDYVVMTEDVNDSDSIIMVTQRGAMKRIDFNV**

30 S43 100.0% 99.0%  **KLINVVRLETDQLITVLTHKGMSLTYSTNELSDTGLRAAGVKSINLKDEDYVVMTEDVNDSDSIIMVTQRGAMKRIDFNV**

31 S17W 100.0% 99.0%  **KLINVVRLETDQLITVLTHKGMSLTYSTNELSDTGLRAAGVKSINLKDEDYVVMTEDVNDSDSIIMVTQRGAMKRIDFNV**

32 48 100.0% 99.1%  **KLINVVRLETDQLITVLTHKGMSLTYSTNELSDTGLRAAGVKSINLKDEDYVVMTEDVNDSDSIIMVTQRGAMKRIDFNV**

33 ABKVF 100.0% 99.0%  **KLINVVRLETDQLITVLTHKGMSLTYSTNELSDTGLRAAGVKSINLKDEDYVVMTEDVNDSDSIIMVTQRGAMKRIDFNV**

34 CICARIA 100.0% 99.9%  **ELINVVRLESDQLITVLTHKGMSLTYSTNELSDTGLRAAGVKSINLKDEDYVVMTEDVNDSDSIIMVTQRGAMKRIDFNV**

35 SKN25lux 100.0% 99.9%  **ELINVVRLESDQLITVLTHKGMSLTYSTNELSDTGLRAAGVKSINLKDEDYVVMTEDVNDSDSIIMVTQRGAMKRIDFNV**

36 HMSC074F11 100.0% 99.0%  **KLINVVRLETDQLITVLTHKGMSLTYSTNELSDTGLRAAGVKSINLKDEDYVVMTEDVNDSDSIIMVTQRGAMKRIDFNV**

37 HMSC068G11 100.0% 99.0%  **KLINVVRLETDQLITVLTHKGMSLTYSTNELSDTGLRAAGVKSINLKDEDYVVMTEDVNDSDSIIMVTQRGAMKRIDFNV**

38 NIHLM037 100.0% 99.0%  **KLINVVRLETDQLITVLTHKGMSLTYSTNELSDTGLRAAGVKSINLKDEDYVVMTEDVNDSDSIIMVTQRGAMKRIDFNV**

39 NIHLM015 100.0% 99.0%  **KLINVVRLETDQLITVLTHKGMSLTYSTNELSDTGLRAAGVKSINLKDEDYVVMTEDVNDSDSIIMVTQRGAMKRIDFNV**

consensus/100%  **cLINVVRLEoDQLITVLTHKGMSLTYSTNELSDTGLRAAGVKSINLKDEDYVVMTEDVNDSDSIIMVTQRGAMKRIDFNV**

consensus/90%  **cLINVVRLEoDQLITVLTHKGMSLTYSTNELSDTGLRAAGVKSINLKDEDYVVMTEDVNDSDSIIMVTQRGAMKRIDFNV**

consensus/80%  **cLINVVRLEoDQLITVLTHKGMSLTYSTNELSDTGLRAAGVKSINLKDEDYVVMTEDVNDSDSIIMVTQRGAMKRIDFNV**

consensus/70%  **cLINVVRLEoDQLITVLTHKGMSLTYSTNELSDTGLRAAGVKSINLKDEDYVVMTEDVNDSDSIIMVTQRGAMKRIDFNV**

cov pid **721**  **. . : . . . . 8** **800**

1 ATCC35984 100.0% 100.0%  **LQEAKRAQRGITLLKELKKKPHRIVAGAVV---KENHTKYIVFSQHHEEYGNIDDVHLSEQYTNGSFIIDTDDFGEVESM**

2 ATCC12228 100.0% 99.9%  **LQEAKRAQRGITLLKELKKKPHRIVAGAVV---KENHTKYIVFSQHHEEYGNIDDVHLSEQYTNGSFIIDTDDFGEVESM**

3 36AM 92.5% 98.9%  **LQEAKRAQRGITLLKELKKKPHRIVAGAVV---KENHTKYVVFSQHHEEYGKIDDVHLSEQYTNGSFIIDTDDFGEVESM**

4 48AF 100.0% 99.9%  **LQEAKRAQRGITLLKELKKKPHRIVAGAVV---KENHTKYIVFSQHHEEYGNIDDVHLSEQYTNGSFIIDTDDFGEVESM**

5 54AF 100.0% 99.0%  **LQEAKRAQRGITLLKELKKKPHRIVAGAVV---KENHTKYVVFSQHHEEYGKIDDVHLSEQYTNGSFIIDTDDFGEVESM**

6 785_SEPI 100.0% 99.1%  **LQEAKRAQRGITLLKELKKKPHRIVAGAVV---KENHTKYVVFSQHHEEYGKIDDVHLSEQYTNGSFIIDTDDFGEVESM**

7 VSE49 100.0% 99.2%  **LQEAKRAQRGITLLKELKKKPHRIVAGAVV---KENHTKYVVFSQHHEEYGKIDDVHLSEQYTNGSFIIDTDDFGEVESM**

8 VSE57 100.0% 99.2%  **LQEAKRAQRGITLLKELKKKPHRIVAGAVV---KENHTKYVVFSQHHEEYGKIDDVHLSEQYTNGSFIIDTDDFGEVESM**

9 SE45 100.0% 99.2%  **LQEAKRAQRGITLLKELKKKPHRIVAGAVV---KENHTKYVVFSQHHEEYGKIDDVHLSEQYTNGSFIIDTDDFGEVESM**

10 SE40 100.0% 99.2%  **LQEAKRAQRGITLLKELKKKPHRIVAGAVV---KENHTKYVVFSQHHEEYGKIDDVHLSEQYTNGSFIIDTDDFGEVESM**

11 32A 100.0% 97.8%  **LQEAKRAQRGITLLKELKKKPHRIVAKKKKKKXXXXHTKYVVFSQHHEEYGKIDDVHLSEQYTNGSFIIDTDDFGEVESM**

12 HD66 100.0% 99.9%  **LQEAKRAQRGITLLKELKKKPHRIVAGAVV---KENHTKYIVFSQHHEEYGNIDDVHLSEQYTNGSFIIDTDDFGEVESM**

13 HD43 100.0% 99.9%  **LQEAKRAQRGITLLKELKKKPHRIVAGAVV---KENHTKYIVFSQHHEEYGNIDDVHLSEQYTNGSFIIDTDDFGEVESM**

14 HD33 100.0% 99.9%  **LQEAKRAQRGITLLKELKKKPHRIVAGAVV---KENHTKYIVFSQHHEEYGNIDDVHLSEQYTNGSFIIDTDDFGEVESM**

15 DE0525 100.0% 99.0%  **LQEAKRAQRGITLLKELKKKPHRIVAGAVV---KENHTKYVVFSQHHEEYGKIDDVHLSEQYTNGSFIIDTDDFGEVESM**

16 4928STDY7071543 100.0% 99.0%  **LQEAKRAQRGITLLKELKKKPHRIVAGAVV---KENHTKYVVFSQHHEEYGKIDDVHLSEQYTNGSFIIDTDDFGEVESM**

17 BAV2502 100.0% 99.1%  **LQEAKRAQRGITLLKELKKKPHRIVAGAVV---KENHTKYVVFSQHHEEYGKIDDVHLSEQYTNGSFIIDTDDFGEVESM**

18 APC3784 100.0% 99.0%  **LQEAKRAQRGITLLKELKKKPHRIVAGAVV---KENHTKYVVFSQHHEEYGKIDDVHLSEQYTNGSFIIDTDDFGEVESM**

19 SESURV_p1_0557 100.0% 99.9%  **LQEAKRAQRGITLLKELKKKPHRIVAGAVV---KENHTKYIVFSQHHEEYGNIDDVHLSEQYTNGSFIIDTDDFGEVESM**

20 SESURV_p1_0612 100.0% 99.0%  **LQEAKRAQRGITLLKELKKKPHRIVAGAVV---KENHTKYVVFSQHHEEYGKIDDVHLSEQYTNGSFIIDTDDFGEVESM**

21 SESURV_p4_1553 100.0% 100.0%  **LQEAKRAQRGITLLKELKKKPHRIVAGAVV---KENHTKYIVFSQHHEEYGNIDDVHLSEQYTNGSFIIDTDDFGEVESM**

22 SESURV_p1_1200 100.0% 99.9%  **LQEAKRAQRGITLLKELKKKPHRIVAGAVV---KENHTKYIVFSQHHEEYGNIDDVHLSEQYTNGSFIIDTDDFGEVESM**

23 SESURV_p3_0825 100.0% 99.9%  **LQEAKRAQRGITLLKELKKKPHRIVAGAVV---KENHTKYIVFSQHHEEYGNIDDVHLSEQYTNGSFIIDTDDFGEVESM**

24 JH 100.0% 99.0%  **LQEAKRAQRGITLLKELKKKPHRIVAGAVV---KENHTKYVVFSQHHEEYGKIDDVHLSEQYTNGSFIIDTDDFGEVESM**

25 Z0118SE0132 100.0% 98.9%  **LQEAKRAQRGITLLKELKKKPHRIVAGAVV---KENHTKYVVFSQHHEEYGKIDDVHLSEQYTNGSFIIDTDDFGEVESM**

26 R5981 100.0% 98.9%  **LQEAKRAQRGITLLKELKKKPHRIVAGAVV---KENHTKYVVFSQHHEEYGKIDDVHLSEQYTNGSFIIDTDDFGEVESM**

27 JH-S-3 100.0% 99.0%  **LQEAKRAQRGITLLKELKKKPHRIVAGAVV---KENHTKYVVFSQHHEEYGKIDDVHLSEQYTNGSFIIDTDDFGEVESM**

28 JH-S-1 100.0% 99.0%  **LQEAKRAQRGITLLKELKKKPHRIVAGAVV---KENHTKYVVFSQHHEEYGKIDDVHLSEQYTNGSFIIDTDDFGEVESM**

29 s2 100.0% 98.9%  **LQEAKRAQRGITLLKELKKKPHRIVAGAVV---KENHTKYVVFSQHHEEYGKIDDVHLSEQYTNGSFIIDTDDFGEVESM**

30 S43 100.0% 99.0%  **LQEAKRAQRGITLLKELKKKPHRIVAGAVV---KENHTKYVVFSQHHEEYGEIDDVHLSEQYTNGSFIIDTDDFGEVESM**

31 S17W 100.0% 99.0%  **LQEAKRAQRGITLLKELKKKPHRIVAGAVV---KENHTKYVVFSQHHEEYGKIDDVHLSEQYTNGSFIIDTDDFGEVESM**

32 48 100.0% 99.1%  **LQEAKRAQRGITLLKELKKKPHRIVAGAVV---KENHTKYVVFSQHHEEYGKIDDVHLSEQYTNGSFIIDTDDFGEVESM**

33 ABKVF 100.0% 99.0%  **LQEAKRAQRGITLLKELKKKPHRIVAGAVV---KENHTKYVVFSQHHEEYGKIDDVHLSEQYTNGSFIIDTDDFGEVESM**

34 CICARIA 100.0% 99.9%  **LQEAKRAQRGITLLKELKKKPHRIVAGAVV---KENHTKYIVFSQHHEEYGNIDDVHLSEQYTNGSFIIDTDDFGEVESM**

35 SKN25lux 100.0% 99.9%  **LQEAKRAQRGITLLKELKKKPHRIVAGAVV---KENHTKYIVFSQHHEEYGNIDDVHLSEQYTNGSFIIDTDDFGEVESM**

36 HMSC074F11 100.0% 99.0%  **LQEAKRAQRGITLLKELKKKPHRIVAGAVV---KENHTKYVVFSQHHEEYGKIDDVHLSEQYTNGSFIIDTDDFGEVESM**

37 HMSC068G11 100.0% 99.0%  **LQEAKRAQRGITLLKELKKKPHRIVAGAVV---KENHTKYVVFSQHHEEYGKIDDVHLSEQYTNGSFIIDTDDFGEVESM**

38 NIHLM037 100.0% 99.0%  **LQEAKRAQRGITLLKELKKKPHRIVAGAVV---KENHTKYVVFSQHHEEYGKIDDVHLSEQYTNGSFIIDTDDFGEVESM**

39 NIHLM015 100.0% 99.0%  **LQEAKRAQRGITLLKELKKKPHRIVAGAVV---KENHTKYVVFSQHHEEYGKIDDVHLSEQYTNGSFIIDTDDFGEVESM**

consensus/100%  **LQEAKRAQRGITLLKELKKKPHRIVAtthh......HTKYlVFSQHHEEYGpIDDVHLSEQYTNGSFIIDTDDFGEVESM**

consensus/90%  **LQEAKRAQRGITLLKELKKKPHRIVAGAVV...KENHTKYlVFSQHHEEYGpIDDVHLSEQYTNGSFIIDTDDFGEVESM**

consensus/80%  **LQEAKRAQRGITLLKELKKKPHRIVAGAVV...KENHTKYlVFSQHHEEYGpIDDVHLSEQYTNGSFIIDTDDFGEVESM**

consensus/70%  **LQEAKRAQRGITLLKELKKKPHRIVAGAVV...KENHTKYlVFSQHHEEYGpIDDVHLSEQYTNGSFIIDTDDFGEVESM**

cov pid **801**  **]** **803**

1 ATCC35984 100.0% 100.0%  **ILE**

2 ATCC12228 100.0% 99.9%  **ILE**

3 36AM 92.5% 98.9%  **ILE**

4 48AF 100.0% 99.9%  **ILE**

5 54AF 100.0% 99.0%  **ILE**

6 785_SEPI 100.0% 99.1%  **ILE**

7 VSE49 100.0% 99.2%  **ILE**

8 VSE57 100.0% 99.2%  **ILE**

9 SE45 100.0% 99.2%  **ILE**

10 SE40 100.0% 99.2%  **ILE**

11 32A 100.0% 97.8%  **ILE**

12 HD66 100.0% 99.9%  **ILE**

13 HD43 100.0% 99.9%  **ILE**

14 HD33 100.0% 99.9%  **ILE**

15 DE0525 100.0% 99.0%  **ILE**

16 4928STDY7071543 100.0% 99.0%  **ILE**

17 BAV2502 100.0% 99.1%  **ILE**

18 APC3784 100.0% 99.0%  **ILE**

19 SESURV_p1_0557 100.0% 99.9%  **ILE**

20 SESURV_p1_0612 100.0% 99.0%  **ILE**

21 SESURV_p4_1553 100.0% 100.0%  **ILE**

22 SESURV_p1_1200 100.0% 99.9%  **ILE**

23 SESURV_p3_0825 100.0% 99.9%  **ILE**

24 JH 100.0% 99.0%  **ILE**

25 Z0118SE0132 100.0% 98.9%  **ILE**

26 R5981 100.0% 98.9%  **ILE**

27 JH-S-3 100.0% 99.0%  **ILE**

28 JH-S-1 100.0% 99.0%  **ILE**

29 s2 100.0% 98.9%  **ILE**

30 S43 100.0% 99.0%  **ILE**

31 S17W 100.0% 99.0%  **ILE**

32 48 100.0% 99.1%  **ILE**

33 ABKVF 100.0% 99.0%  **ILE**

34 CICARIA 100.0% 99.9%  **ILE**

35 SKN25lux 100.0% 99.9%  **ILE**

36 HMSC074F11 100.0% 99.0%  **ILE**

37 HMSC068G11 100.0% 99.0%  **ILE**

38 NIHLM037 100.0% 99.0%  **ILE**

39 NIHLM015 100.0% 99.0%  **ILE**

consensus/100%  **ILE**

consensus/90%  **ILE**

consensus/80%  **ILE**

consensus/70%  **ILE**

**Supplementary Figure 3.** Multiple sequence alignment of the predicted amino acid sequences of ParC carried by *S. epidermidis* isolates and close genomes retrieved from the BV-BRC database compared to that of *S. epidermidis* strain ATCC®12228 and ATCC®35984. The alignment was visualized using MView version 1.63 hosted by the EMBL-EBI; cov, coverage; pid, percent identity.

cov pid  **1** **[ . . . . : . . .** **80**

1 ATCC35984 100.0% 100.0%  **MAMNKQNNYSDDSIQVLEGLEAVRKRPGMYIGSTDKRGLHHLVYEVVDNSVDEVLNGYGDAITVTINQDGSISIEDNGRG**

2 ATCC12228 100.0% 100.0%  **MAMNKQNNYSDDSIQVLEGLEAVRKRPGMYIGSTDKRGLHHLVYEVVDNSVDEVLNGYGDAITVTINQDGSISIEDNGRG**

3 36AM 100.0% 99.5%  **MAMNKQNNYSDDSIQVLEGLEAVRKRPGMYIGSTDKRGLHHLVYEVVDNSVDEVLNGYGDAITVTINQDGSISIEDNGRG**

4 48AF 100.0% 100.0%  **MAMNKQNNYSDDSIQVLEGLEAVRKRPGMYIGSTDKRGLHHLVYEVVDNSVDEVLNGYGDAITVTINQDGSISIEDNGRG**

5 54AF 100.0% 99.4%  **MAMNKQNNYSDDSIQVLEGLEAVRKRPGMYIGSTDKRGLHHLVYEVVDNSVDEVLNGYGDAITVTINQDGSISIEDNGRG**

6 785_SEPI 100.0% 99.5%  **MAMNKQNNYSDDSIQVLEGLEAVRKRPGMYIGSTDKRGLHHLVYEVVDNSVDEVLNGYGDAITVTINQDGSISIEDNGRG**

7 VSE49 100.0% 99.4%  **MAMNKQNNYSDDSIQVLEGLEAVRKRPGMYIGSTDKRGLHHLVYEVVDNSVDEVLNGYGDAITVTINQDGSISIEDNGRG**

8 VSE57 100.0% 99.4%  **MAMNKQNNYSDDSIQVLEGLEAVRKRPGMYIGSTDKRGLHHLVYEVVDNSVDEVLNGYGDAITVTINQDGSISIEDNGRG**

9 SE45 100.0% 99.4%  **MAMNKQNNYSDDSIQVLEGLEAVRKRPGMYIGSTDKRGLHHLVYEVVDNSVDEVLNGYGDAITVTINQDGSISIEDNGRG**

10 SE40 100.0% 99.4%  **MAMNKQNNYSDDSIQVLEGLEAVRKRPGMYIGSTDKRGLHHLVYEVVDNSVDEVLNGYGDAITVTINQDGSISIEDNGRG**

11 32A 100.0% 99.4%  **MAMNKQNNYSDDSIQVLEGLEAVRKRPGMYIGSTDKRGLHHLVYEVVDNSVDEVLNGYGDAITLTINQDGSISIEDNGRG**

12 HD66 100.0% 100.0%  **MAMNKQNNYSDDSIQVLEGLEAVRKRPGMYIGSTDKRGLHHLVYEVVDNSVDEVLNGYGDAITVTINQDGSISIEDNGRG**

13 HD43 91.0% 100.0%  **------------------------------------------------------------AITVTINQDGSISIEDNGRG**

14 HD33 100.0% 100.0%  **MAMNKQNNYSDDSIQVLEGLEAVRKRPGMYIGSTDKRGLHHLVYEVVDNSVDEVLNGYGDAITVTINQDGSISIEDNGRG**

15 DE0525 100.0% 99.4%  **MAMNKQNNYSDDSIQVLEGLEAVRKRPGMYIGSTDKRGLHHLVYEVVDNSVDEVLNGYGDAITLTINQDGSISIEDNGRG**

16 4928STDY7071543 100.0% 99.4%  **MAMNKQNNYSDDSIQVLEGLEAVRKRPGMYIGSTDKRGLHHLVYEVVDNSVDEVLNGYGDAITVTINQDGSISIEDNGRG**

17 BAV2502 100.0% 99.5%  **MAMNKQNNYSDDSIQVLEGLEAVRKRPGMYIGSTDKRGLHHLVYEVVDNSVDEVLNGYGDAITVTINQDGSISIEDNGRG**

18 APC3784 100.0% 99.5%  **MAMNKQNNYSDDSIQVLEGLEAVRKRPGMYIGSTDKRGLHHLVYEVVDNSVDEVLNGYGDAITVTINQDGSISIEDNGRG**

19 SESURV_p1_0557 100.0% 99.8%  **MAMNKQNNYSDDSIQVLEGLEAVRKRPGMYIGSTDKRGLHHLVYEVVDNSVDEVLNGYGDAITVTINQDGSISIEDNGRG**

20 SESURV_p1_0612 100.0% 99.7%  **MAMNKQNNYSDDSIQVLEGLEAVRKRPGMYIGSTDKRGLHHLVYEVVDNSVDEVLNGYGDAITVTINQDGSISIEDNGRG**

21 SESURV_p4_1553 100.0% 99.8%  **MAMNKQNNYSDDSIQVLEGLEAVRKRPGMYIGSTDKRGLHHLVYEVVDNSVDEVLNGYGDAITVTINQDGSISIEDNGRG**

22 SESURV_p1_1200 100.0% 99.8%  **MAMNKQNNYSDDSIQVLEGLEAVRKRPGMYIGSTDKRGLHHLVYEVVDNSVDEVLNGYGDAITVTINQDGSISIEDNGRG**

23 SESURV_p3_0825 100.0% 99.7%  **MAMNKQNNYSDDSIQVLEGLEAVRKRPGMYIGSTDKRGLHHLVYEVVDNSVDEVLNGYGDAITVTINQDGSISIEDNGRG**

24 JH 100.0% 99.4%  **MAMNKQNNYSDDSIQVLEGLEAVRKRPGMYIGSTDKRGLHHLVYEVVDNSVDEVLNGYGDAITLTINQDGSISIEDNGRG**

25 Z0118SE0132 100.0% 99.7%  **MAMNKQNNYSDDSIQVLEGLEAVRKRPGMYIGSTDKRGLHHLVYEVVDNSVDEVLNGYGDAITVTINQDGSISIEDNGRG**

26 R5981 100.0% 99.8%  **MAMNKQNNYSDDSIQVLEGLEAVRKRPGMYIGSTDKRGLHHLVYEVVDNSVDEVLNGYGDAITVTINQDGSISIEDNGRG**

27 JH-S-3 100.0% 99.4%  **MAMNKQNNYSDDSIQVLEGLEAVRKRPGMYIGSTDKRGLHHLVYEVVDNSVDEVLNGYGDAITLTINQDGSISIEDNGRG**

28 JH-S-1 100.0% 99.4%  **MAMNKQNNYSDDSIQVLEGLEAVRKRPGMYIGSTDKRGLHHLVYEVVDNSVDEVLNGYGDAITLTINQDGSISIEDNGRG**

29 s2 100.0% 99.5%  **MAMNKQNNYSDDSIQVLEGLEAVRKRPGMYIGSTDKRGLHHLVYEVVDNSVDEVLNGYGDAITVTINQDGSISIEDNGRG**

30 S43 100.0% 99.7%  **MAMNKQNNYSDDSIQVLEGLEAVRKRPGMYIGSTDKRGLHHLVYEVVDNSVDEVLNGYGDAITVTINQDGSISIEDNGRG**

31 S17W 100.0% 99.4%  **MAMNKQNNYSDDSIQVLEGLEAVRKRPGMYIGSTDKRGLHHLVYEVVDNSVDEVLNGYGDAITLTINQDGSISIEDNGRG**

32 48 100.0% 99.5%  **MAMNKQNNYSDDSIQVLEGLEAVRKRPGMYIGSTDKRGLHHLVYEVVDNSVDEVLNGYGDAITVTINQDGSISIEDNGRG**

33 ABKVF 100.0% 99.4%  **MAMNKQNNYSDDSIQVLEGLEAVRKRPGMYIGSTDKRGLHHLVYEVVDNSVDEVLNGYGDAITLTINQDGSISIEDNGRG**

34 CICARIA 100.0% 100.0%  **MAMNKQNNYSDDSIQVLEGLEAVRKRPGMYIGSTDKRGLHHLVYEVVDNSVDEVLNGYGDAITVTINQDGSISIEDNGRG**

35 SKN25lux 100.0% 99.8%  **MAMNKQNNYSDDSIQVLEGLEAVRKRPGMYIGSTDKRGLHHLVYEVVDNSVDEVLNGYGDAITVTINQDGSISIEDNGRG**

36 HMSC074F11 100.0% 99.4%  **MAMNKQNNYSDDSIQVLEGLEAVRKRPGMYIGSTDKRGLHHLVYEVVDNSVDEVLNGYGDAITLTINQDGSISIEDNGRG**

37 HMSC068G11 100.0% 99.4%  **MAMNKQNNYSDDSIQVLEGLEAVRKRPGMYIGSTDKRGLHHLVYEVVDNSVDEVLNGYGDAITLTINQDGSISIEDNGRG**

38 NIHLM037 56.2% 99.2%  **--------------------------------------------------------------------------------**

39 NIHLM015 100.0% 99.4%  **MAMNKQNNYSDDSIQVLEGLEAVRKRPGMYIGSTDKRGLHHLVYEVVDNSVDEVLNGYGDAITLTINQDGSISIEDNGRG**

consensus/100%  **................................................................................**

consensus/90%  **MAMNKQNNYSDDSIQVLEGLEAVRKRPGMYIGSTDKRGLHHLVYEVVDNSVDEVLNGYGDAITlTINQDGSISIEDNGRG**

consensus/80%  **MAMNKQNNYSDDSIQVLEGLEAVRKRPGMYIGSTDKRGLHHLVYEVVDNSVDEVLNGYGDAITlTINQDGSISIEDNGRG**

consensus/70%  **MAMNKQNNYSDDSIQVLEGLEAVRKRPGMYIGSTDKRGLHHLVYEVVDNSVDEVLNGYGDAITVTINQDGSISIEDNGRG**

cov pid  **81**  **. 1 . . . . : .** **160**

1 ATCC35984 100.0% 100.0%  **MPTGIHASGKPTAEVIFTVLHAGGKFGQGGYKTSGGLHGVGASVVNALSEWLEVEIHRDGNIYTQNFKNGGIPATGLVKT**

2 ATCC12228 100.0% 100.0%  **MPTGIHASGKPTAEVIFTVLHAGGKFGQGGYKTSGGLHGVGASVVNALSEWLEVEIHRDGNIYTQNFKNGGIPATGLVKT**

3 36AM 100.0% 99.5%  **MPTGIHASGKPTAEVIFTVLHAGGKFGQGGYKTSGGLHGVGASVVNALSEWLEVEIHRDGNIYTQNFKNGGIPATGLVKT**

4 48AF 100.0% 100.0%  **MPTGIHASGKPTAEVIFTVLHAGGKFGQGGYKTSGGLHGVGASVVNALSEWLEVEIHRDGNIYTQNFKNGGIPATGLVKT**

5 54AF 100.0% 99.4%  **MPTGIHASGKPTAEVIFTVLHAGGKFGQGGYKTSGGLHGVGASVVNALSEWLEVEIHRDGNIYTQNFKNGGIPATGLVKT**

6 785_SEPI 100.0% 99.5%  **MPTGIHASGKPTAEVIFTVLHAGGKFGQGGYKTSGGLHGVGASVVNALSEWLEVEIHRDGNIYTQNFKNGGIPATGLVKT**

7 VSE49 100.0% 99.4%  **MPTGIHASGKPTAEVIFTVLHAGGKFGQGGYKTSGGLHGVGASVVNALSEWLEVEIHRDGNIYTQNFKNGGIPATGLVKT**

8 VSE57 100.0% 99.4%  **MPTGIHASGKPTAEVIFTVLHAGGKFGQGGYKTSGGLHGVGASVVNALSEWLEVEIHRDGNIYTQNFKNGGIPATGLVKT**

9 SE45 100.0% 99.4%  **MPTGIHASGKPTAEVIFTVLHAGGKFGQGGYKTSGGLHGVGASVVNALSEWLEVEIHRDGNIYTQNFKNGGIPATGLVKT**

10 SE40 100.0% 99.4%  **MPTGIHASGKPTAEVIFTVLHAGGKFGQGGYKTSGGLHGVGASVVNALSEWLEVEIHRDGNIYTQNFKNGGIPATGLVKT**

11 32A 100.0% 99.4%  **MPTGIHASGKPTAEVIFTVLHAGGKFGQGGYKTSGGLHGVGASVVNALSEWLEVEIHRDGNIYTQNFKNGGIPATGLVKT**

12 HD66 100.0% 100.0%  **MPTGIHASGKPTAEVIFTVLHAGGKFGQGGYKTSGGLHGVGASVVNALSEWLEVEIHRDGNIYTQNFKNGGIPATGLVKT**

13 HD43 91.0% 100.0%  **MPTGIHASGKPTAEVIFTVLHAGGKFGQGGYKTSGGLHGVGASVVNALSEWLEVEIHRDGNIYTQNFKNGGIPATGLVKT**

14 HD33 100.0% 100.0%  **MPTGIHASGKPTAEVIFTVLHAGGKFGQGGYKTSGGLHGVGASVVNALSEWLEVEIHRDGNIYTQNFKNGGIPATGLVKT**

15 DE0525 100.0% 99.4%  **MPTGIHASGKPTAEVIFTVLHAGGKFGQGGYKTSGGLHGVGASVVNALSEWLEVEIHRDGNIYTQNFKNGGIPATGLVKT**

16 4928STDY7071543 100.0% 99.4%  **MPTGIHASGKPTAEVIFTVLHAGGKFGQGGYKTSGGLHGVGASVVNALSEWLEVEIHRDGNIYTQNFKNGGIPATGLVKT**

17 BAV2502 100.0% 99.5%  **MPTGIHASGKPTAEVIFTVLHAGGKFGQGGYKTSGGLHGVGASVVNALSEWLEVEIHRDGNIYTQNFKNGGIPATGLVKT**

18 APC3784 100.0% 99.5%  **MPTGIHASGKPTAEVIFTVLHAGGKFGQGGYKTSGGLHGVGASVVNALSEWLEVEIHRDGNIYTQNFKNGGIPATGLVKT**

19 SESURV_p1_0557 100.0% 99.8%  **MPTGIHVSGKPTAEVIFTVLHAGGKFGQGGYKTSGGLHGVGASVVNALSEWLEVEIHRDGNIYTQNFKNGGIPATGLVKT**

20 SESURV_p1_0612 100.0% 99.7%  **MPTGIHASGKPTAEVIFTVLHAGGKFGQGGYKTSGGLHGVGASVVNALSEWLEVEIHRDGNIYTQNFKNGGIPATGLVKT**

21 SESURV_p4_1553 100.0% 99.8%  **MPTGIHVSGKPTAEVIFTVLHAGGKFGQGGYKTSGGLHGVGASVVNALSEWLEVEIHRDGNIYTQNFKNGGIPATGLVKT**

22 SESURV_p1_1200 100.0% 99.8%  **MPTGIHVSGKPTAEVIFTVLHAGGKFGQGGYKTSGGLHGVGASVVNALSEWLEVEIHRDGNIYTQNFKNGGIPATGLVKT**

23 SESURV_p3_0825 100.0% 99.7%  **MPTGIHASGKPTAEVIFTVLHAGGKFGQGGYKTSGGLHGVGASVVNALSEWLEVEIHRDGNIYTQNFKNGGIPATGLVKT**

24 JH 100.0% 99.4%  **MPTGIHASGKPTAEVIFTVLHAGGKFGQGGYKTSGGLHGVGASVVNALSEWLEVEIHRDGNIYTQNFKNGGIPATGLVKT**

25 Z0118SE0132 100.0% 99.7%  **MPTGIHASGKPTAEVIFTVLHAGGKFGQGGYKTSGGLHGVGASVVNALSEWLEVEIHRDGNIYTQNFKNGGIPATGLVKT**

26 R5981 100.0% 99.8%  **MPTGIHASGKPTAEVIFTVLHAGGKFGQGGYKTSGGLHGVGASVVNALSEWLEVEIHRDGNIYTQNFKNGGIPATGLVKT**

27 JH-S-3 100.0% 99.4%  **MPTGIHASGKPTAEVIFTVLHAGGKFGQGGYKTSGGLHGVGASVVNALSEWLEVEIHRDGNIYTQNFKNGGIPATGLVKT**

28 JH-S-1 100.0% 99.4%  **MPTGIHASGKPTAEVIFTVLHAGGKFGQGGYKTSGGLHGVGASVVNALSEWLEVEIHRDGNIYTQNFKNGGIPATGLVKT**

29 s2 100.0% 99.5%  **MPTGIHASGKPTAEVIFTVLHAGGKFGQGGYKTSGGLHGVGASVVNALSEWLEVEIHRDGNIYTQNFKNGGIPATGLVKT**

30 S43 100.0% 99.7%  **MPTGIHASGKPTAEVIFTVLHAGGKFGQGGYKTSGGLHGVGASVVNALSEWLEVEIHRDGNIYTQNFKNGGIPATGLVKT**

31 S17W 100.0% 99.4%  **MPTGIHASGKPTAEVIFTVLHAGGKFGQGGYKTSGGLHGVGASVVNALSEWLEVEIHRDGNIYTQNFKNGGIPATGLVKT**

32 48 100.0% 99.5%  **MPTGIHASGKPTAEVIFTVLHAGGKFGQGGYKTSGGLHGVGASVVNALSEWLEVEIHRDGNIYTQNFKNGGIPATGLVKT**

33 ABKVF 100.0% 99.4%  **MPTGIHASGKPTAEVIFTVLHAGGKFGQGGYKTSGGLHGVGASVVNALSEWLEVEIHRDGNIYTQNFKNGGIPATGLVKT**

34 CICARIA 100.0% 100.0%  **MPTGIHASGKPTAEVIFTVLHAGGKFGQGGYKTSGGLHGVGASVVNALSEWLEVEIHRDGNIYTQNFKNGGIPATGLVKT**

35 SKN25lux 100.0% 99.8%  **MPTGIHASGKPTAEVIFTVLHAGGKFGQGGYKTSGGLHGVGASVVNALSEWLEVEIHRDGNIYTQNFKNGGIPATGLVKT**

36 HMSC074F11 100.0% 99.4%  **MPTGIHASGKPTAEVIFTVLHAGGKFGQGGYKTSGGLHGVGASVVNALSEWLEVEIHRDGNIYTQNFKNGGIPATGLVKT**

37 HMSC068G11 100.0% 99.4%  **MPTGIHASGKPTAEVIFTVLHAGGKFGQGGYKTSGGLHGVGASVVNALSEWLEVEIHRDGNIYTQNFKNGGIPATGLVKT**

38 NIHLM037 56.2% 99.2%  **--------------------------------------------------------------------------------**

39 NIHLM015 100.0% 99.4%  **MPTGIHASGKPTAEVIFTVLHAGGKFGQGGYKTSGGLHGVGASVVNALSEWLEVEIHRDGNIYTQNFKNGGIPATGLVKT**

consensus/100%  **................................................................................**

consensus/90%  **MPTGIHsSGKPTAEVIFTVLHAGGKFGQGGYKTSGGLHGVGASVVNALSEWLEVEIHRDGNIYTQNFKNGGIPATGLVKT**

consensus/80%  **MPTGIHASGKPTAEVIFTVLHAGGKFGQGGYKTSGGLHGVGASVVNALSEWLEVEIHRDGNIYTQNFKNGGIPATGLVKT**

consensus/70%  **MPTGIHASGKPTAEVIFTVLHAGGKFGQGGYKTSGGLHGVGASVVNALSEWLEVEIHRDGNIYTQNFKNGGIPATGLVKT**

cov pid **161**  **. . . 2 . . . .** **240**

1 ATCC35984 100.0% 100.0%  **GKTKKTGTKVTFKPDSEIFKSTTTFNFDILSERLQESAFLLKDLKITLTDLRSGKEREEIYHYEEGIKEFVSYVNEGKEV**

2 ATCC12228 100.0% 100.0%  **GKTKKTGTKVTFKPDSEIFKSTTTFNFDILSERLQESAFLLKDLKITLTDLRSGKEREEIYHYEEGIKEFVSYVNEGKEV**

3 36AM 100.0% 99.5%  **GKTKKTGTKVTFKPDSEIFKSTTTFNFDILSERLQESAFLLKDLKITLTDLRSGKEREEIYHYEEGIKEFVSYVNEGKEV**

4 48AF 100.0% 100.0%  **GKTKKTGTKVTFKPDSEIFKSTTTFNFDILSERLQESAFLLKDLKITLTDLRSGKEREEIYHYEEGIKEFVSYVNEGKEV**

5 54AF 100.0% 99.4%  **GKTKKTGTKVTFKPDSEIFKSTTTFNFDILSERLQESAFLLKDLKITLTDLRSGKEREEIYHYEEGIKEFVSYVNEGKEV**

6 785_SEPI 100.0% 99.5%  **GKTKKTGTKVTFKPDSEIFKSTTTFNFDILSERLQESAFLLKDLKITLTDLRSGKEREEIYHYEEGIKEFVSYVNEGKEV**

7 VSE49 100.0% 99.4%  **GKTKKTGTKVTFKPDPEIFKSTTTFNFDILSERLQESAFLLKDLKITLTDLRSGKEREEIYHYEEGIKEFVSYVNEGKEV**

8 VSE57 100.0% 99.4%  **GKTKKTGTKVTFKPDPEIFKSTTTFNFDILSERLQESAFLLKDLKITLTDLRSGKEREEIYHYEEGIKEFVSYVNEGKEV**

9 SE45 100.0% 99.4%  **GKTKKTGTKVTFKPDPEIFKSTTTFNFDILSERLQESAFLLKDLKITLTDLRSGKEREEIYHYEEGIKEFVSYVNEGKEV**

10 SE40 100.0% 99.4%  **GKTKKTGTKVTFKPDPEIFKSTTTFNFDILSERLQESAFLLKDLKITLTDLRSGKEREEIYHYEEGIKEFVSYVNEGKEV**

11 32A 100.0% 99.4%  **GKTKKTGTKVTFKPDSEIFKSTTTFNFDILSERLQESAFLLKDLKITLTDLRSGKEREEIYHYEEGIKEFVSYVNEGKEV**

12 HD66 100.0% 100.0%  **GKTKKTGTKVTFKPDSEIFKSTTTFNFDILSERLQESAFLLKDLKITLTDLRSGKEREEIYHYEEGIKEFVSYVNEGKEV**

13 HD43 91.0% 100.0%  **GKTKKTGTKVTFKPDSEIFKSTTTFNFDILSERLQESAFLLKDLKITLTDLRSGKEREEIYHYEEGIKEFVSYVNEGKEV**

14 HD33 100.0% 100.0%  **GKTKKTGTKVTFKPDSEIFKSTTTFNFDILSERLQESAFLLKDLKITLTDLRSGKEREEIYHYEEGIKEFVSYVNEGKEV**

15 DE0525 100.0% 99.4%  **GKTKKTGTKVTFKPDSEIFKSTTTFNFDILSERLQESAFLLKDLKITLTDLRSGKEREEIYHYEEGIKEFVSYVNEGKEV**

16 4928STDY7071543 100.0% 99.4%  **GKTKKTGTKVTFKPDPEIFKSTTTFNFDILSERLQESAFLLKDLKITLTDLRSGKEREEIYHYEEGIKEFVSYVNEGKEV**

17 BAV2502 100.0% 99.5%  **GKTKKTGTKVTFKPDSEIFKSTTTFNFDILSERLQESAFLLKDLKITLTDLRSGKEREEIYHYEEGIKEFVSYVNEGKEV**

18 APC3784 100.0% 99.5%  **GKTKKTGTKVTFKPDSEIFKSTTTFNFDILSERLQESAFLLKDLKITLTDLRSGKEREEIYHYEEGIKEFVSYVNEGKEV**

19 SESURV_p1_0557 100.0% 99.8%  **GKTKKTGTKVTFKPDSEIFKSTTTFNFDILSERLQESAFLLKDLKITLTDLRSGKEREEIYHYEEGIKEFVSYVNEGKEV**

20 SESURV_p1_0612 100.0% 99.7%  **GKTKKTGTKVTFKPDSEIFKSTTTFNFDILSERLQESAFLLKDLKITLTDLRSGKEREEIYHYEEGIKEFVSYVNEGKEV**

21 SESURV_p4_1553 100.0% 99.8%  **GKTKKTGTKVTFKPDSEIFKSTTTFNFDILSERLQESAFLLKDLKITLTDLRSGKEREEIYHYEEGIKEFVSYVNEGKEV**

22 SESURV_p1_1200 100.0% 99.8%  **GKTKKTGTKVTFKPDSEIFKSTTTFNFDILSERLQESAFLLKDLKITLTDLRSGKEREEIYHYEEGIKEFVSYVNEGKEV**

23 SESURV_p3_0825 100.0% 99.7%  **EKTKKTGTKVTFKPDSEIFKSTTTFNFDILSERLQESAFLLKDLKITLTDLRSGKEREEIYHYEEGIKEFVSYVNEGKEV**

24 JH 100.0% 99.4%  **GKTKKTGTKVTFKPDSEIFKSTTTFNFDILSERLQESAFLLKDLKITLTDLRSGKEREEIYHYEEGIKEFVSYVNEGKEV**

25 Z0118SE0132 100.0% 99.7%  **GKTKKTGTKVTFKPDPEIFKSTTTFNFDILSERLQESAFLLKDLKITLTDLRSGKEREEIYHYEEGIKEFVSYVNEGKEV**

26 R5981 100.0% 99.8%  **GKTKKTGTKVTFKPDSEIFKSTTTFNFDILSERLQESAFLLKDLKITLTDLRSGKEREEIYHYEEGIKEFVSYVNEGKEV**

27 JH-S-3 100.0% 99.4%  **GKTKKTGTKVTFKPDSEIFKSTTTFNFDILSERLQESAFLLKDLKITLTDLRSGKEREEIYHYEEGIKEFVSYVNEGKEV**

28 JH-S-1 100.0% 99.4%  **GKTKKTGTKVTFKPDSEIFKSTTTFNFDILSERLQESAFLLKDLKITLTDLRSGKEREEIYHYEEGIKEFVSYVNEGKEV**

29 s2 100.0% 99.5%  **GKTKKTGTKVTFKPDPEIFKSTTTFNFDILSERLQESAFLLKDLKITLTDLRSGKEREEIYHYEEGIKEFVSYVNEGKEV**

30 S43 100.0% 99.7%  **GKTKKTGTKVTFKPDSEIFKSTTTFNFDILSERLQESAFLLKDLKITLTDLRSGKEREEIYHYEEGIKEFVSYVNEGKEV**

31 S17W 100.0% 99.4%  **GKTKKTGTKVTFKPDSEIFKSTTTFNFDILSERLQESAFLLKDLKITLTDLRSGKEREEIYHYEEGIKEFVSYVNEGKEV**

32 48 100.0% 99.5%  **GKTKKTGTKVTFKPDSEIFKSTTTFNFDILSERLQESAFLLKDLKITLTDLRSGKEREEIYHYEEGIKEFVSYVNEGKEV**

33 ABKVF 100.0% 99.4%  **GKTKKTGTKVTFKPDSEIFKSTTTFNFDILSERLQESAFLLKDLKITLTDLRSGKEREEIYHYEEGIKEFVSYVNEGKEV**

34 CICARIA 100.0% 100.0%  **GKTKKTGTKVTFKPDSEIFKSTTTFNFDILSERLQESAFLLKDLKITLTDLRSGKEREEIYHYEEGIKEFVSYVNEGKEV**

35 SKN25lux 100.0% 99.8%  **GKTKKTGTKVTFKPDSEIFKSTTTFNFDILSERLQESAFLLKDLKITLTDLRSGKEREEIYHYEEGIKEFVSYVNEGKEV**

36 HMSC074F11 100.0% 99.4%  **GKTKKTGTKVTFKPDSEIFKSTTTFNFDILSERLQESAFLLKDLKITLTDLRSGKEREEIYHYEEGIKEFVSYVNEGKEV**

37 HMSC068G11 100.0% 99.4%  **GKTKKTGTKVTFKPDSEIFKSTTTFNFDILSERLQESAFLLKDLKITLTDLRSGKEREEIYHYEEGIKEFVSYVNEGKEV**

38 NIHLM037 56.2% 99.2%  **--------------------------------------------------------------------------------**

39 NIHLM015 100.0% 99.4%  **GKTKKTGTKVTFKPDSEIFKSTTTFNFDILSERLQESAFLLKDLKITLTDLRSGKEREEIYHYEEGIKEFVSYVNEGKEV**

consensus/100%  **................................................................................**

consensus/90%  **GKTKKTGTKVTFKPDsEIFKSTTTFNFDILSERLQESAFLLKDLKITLTDLRSGKEREEIYHYEEGIKEFVSYVNEGKEV**

consensus/80%  **GKTKKTGTKVTFKPDsEIFKSTTTFNFDILSERLQESAFLLKDLKITLTDLRSGKEREEIYHYEEGIKEFVSYVNEGKEV**

consensus/70%  **GKTKKTGTKVTFKPDSEIFKSTTTFNFDILSERLQESAFLLKDLKITLTDLRSGKEREEIYHYEEGIKEFVSYVNEGKEV**

cov pid **241**  **: . . . . 3 . .** **320**

1 ATCC35984 100.0% 100.0%  **LHDVTTFAGHSNGIEVDVAFQYNDQYSESILSFVNNVRTKDGGTHEVGFKTAMTRVFNEYARRINELKDKDKNLDGNDIR**

2 ATCC12228 100.0% 100.0%  **LHDVTTFAGHSNGIEVDVAFQYNDQYSESILSFVNNVRTKDGGTHEVGFKTAMTRVFNEYARRINELKDKDKNLDGNDIR**

3 36AM 100.0% 99.5%  **LHDVTTFAGHSNGIEVDVAFQYNDQYSESILSFVNNVRTKDGGTHEVGFKTAMTRVFNEYARRINELKDKDKNLDGNDIR**

4 48AF 100.0% 100.0%  **LHDVTTFAGHSNGIEVDVAFQYNDQYSESILSFVNNVRTKDGGTHEVGFKTAMTRVFNEYARRINELKDKDKNLDGNDIR**

5 54AF 100.0% 99.4%  **LHDVTTFAGHSNGIEVDVAFQYNDQYSESILSFVNNVRTKDGGTHEVGFKTAMTRVFNEYARRINELKDKDKNLDGNDIR**

6 785_SEPI 100.0% 99.5%  **LHDVTTFAGHSNGIEVDVAFQYNDQYSESILSFVNNVRTKDGGTHEVGFKTAMTRVFNEYARRINELKDKDKNLDGNDIR**

7 VSE49 100.0% 99.4%  **LHDVTTFAGHSNGIEVDVAFQYNDQYSESILSFVNNVRTKDGGTHEVGFKTAMTRVFNEYARRINELKDKDKNLDGNDIR**

8 VSE57 100.0% 99.4%  **LHDVTTFAGHSNGIEVDVAFQYNDQYSESILSFVNNVRTKDGGTHEVGFKTAMTRVFNEYARRINELKDKDKNLDGNDIR**

9 SE45 100.0% 99.4%  **LHDVTTFAGHSNGIEVDVAFQYNDQYSESILSFVNNVRTKDGGTHEVGFKTAMTRVFNEYARRINELKDKDKNLDGNDIR**

10 SE40 100.0% 99.4%  **LHDVTTFAGHSNGIEVDVAFQYNDQYSESILSFVNNVRTKDGGTHEVGFKTAMTRVFNEYARRINELKDKDKNLDGNDIR**

11 32A 100.0% 99.4%  **LHDVTTFAGHSNGIEVDVAFQYNDQYSESILSFVNNVRTKDGGTHEVGFKTAMTRVFNEYARRINELKDKDKNLDGNDIR**

12 HD66 100.0% 100.0%  **LHDVTTFAGHSNGIEVDVAFQYNDQYSESILSFVNNVRTKDGGTHEVGFKTAMTRVFNEYARRINELKDKDKNLDGNDIR**

13 HD43 91.0% 100.0%  **LHDVTTFAGHSNGIEVDVAFQYNDQYSESILSFVNNVRTKDGGTHEVGFKTAMTRVFNEYARRINELKDKDKNLDGNDIR**

14 HD33 100.0% 100.0%  **LHDVTTFAGHSNGIEVDVAFQYNDQYSESILSFVNNVRTKDGGTHEVGFKTAMTRVFNEYARRINELKDKDKNLDGNDIR**

15 DE0525 100.0% 99.4%  **LHDVTTFAGHSNGIEVDVAFQYNDQYSESILSFVNNVRTKDGGTHEVGFKTAMTRVFNEYARRINELKDKDKNLDGNDIR**

16 4928STDY7071543 100.0% 99.4%  **LHDVTTFAGHSNGIEVDVAFQYNDQYSESILSFVNNVRTKDGGTHEVGFKTAMTRVFNEYARRINELKDKDKNLDGNDIR**

17 BAV2502 100.0% 99.5%  **LHDVTTFAGHSNGIEVDVAFQYNDQYSESILSFVNNVRTKDGGTHEVGFKTAMTRVFNEYARRINELKDKDKNLDGNDIR**

18 APC3784 100.0% 99.5%  **LHDVTTFAGHSNGIEVDVAFQYNDQYSESILSFVNNVRTKDGGTHEVGFKTAMTRVFNEYARRINELKDKDKNLDGNDIR**

19 SESURV_p1_0557 100.0% 99.8%  **LHDVTTFAGHSNGIEVDVAFQYNDQYSESILSFVNNVRTKDGGTHEVGFKTAMTRVFNEYARRINELKDKDKNLDGNDIR**

20 SESURV_p1_0612 100.0% 99.7%  **LHDVTTFAGHSNGIEVDVAFQYNDQYSESILSFVNNVRTKDGGTHEVGFKTAMTRVFNEYARRINELKDKDKNLDGNDIR**

21 SESURV_p4_1553 100.0% 99.8%  **LHDVTTFAGHSNGIEVDVAFQYNDQYSESILSFVNNVRTKDGGTHEVGFKTAMTRVFNEYARRINELKDKDKNLDGNDIR**

22 SESURV_p1_1200 100.0% 99.8%  **LHDVTTFAGHSNGIEVDVAFQYNDQYSESILSFVNNVRTKDGGTHEVGFKTAMTRVFNEYARRINELKDKDKNLDGNDIR**

23 SESURV_p3_0825 100.0% 99.7%  **LHDVTTFAGHSNGIEVDVAFQYNDQYSESILSFVNNVRTKDGGTHEVGFKTAMTRVFNEYARRINELKDKDKNLDGNDIR**

24 JH 100.0% 99.4%  **LHDVTTFAGHSNGIEVDVAFQYNDQYSESILSFVNNVRTKDGGTHEVGFKTAMTRVFNEYARRINELKDKDKNLDGNDIR**

25 Z0118SE0132 100.0% 99.7%  **LHDVTTFAGHSNGIEVDVAFQYNDQYSESILSFVNNVRTKDGGTHEVGFKTAMTRVFNEYARRINELKDKDKNLDGNDIR**

26 R5981 100.0% 99.8%  **LHDVTTFAGHSNGIEVDVAFQYNDQYSESILSFVNNVRTKDGGTHEVGFKTAMTRVFNEYARRINELKDKDKNLDGNDIR**

27 JH-S-3 100.0% 99.4%  **LHDVTTFAGHSNGIEVDVAFQYNDQYSESILSFVNNVRTKDGGTHEVGFKTAMTRVFNEYARRINELKDKDKNLDGNDIR**

28 JH-S-1 100.0% 99.4%  **LHDVTTFAGHSNGIEVDVAFQYNDQYSESILSFVNNVRTKDGGTHEVGFKTAMTRVFNEYARRINELKDKDKNLDGNDIR**

29 s2 100.0% 99.5%  **LHDVTTFAGHSNGIEVDVAFQYNDQYSESILSFVNNVRTKDGGTHEVGFKTAMTRVFNEYARRINELKDKDKNLDGNDIR**

30 S43 100.0% 99.7%  **LHDVTTFAGHSNGIEVDVAFQYNDQYSESILSFVNNVRTKDGGTHEVGFKTAMTRVFNEYARRINELKDKDKNLDGNDIR**

31 S17W 100.0% 99.4%  **LHDVTTFAGHSNGIEVDVAFQYNDQYSESILSFVNNVRTKDGGTHEVGFKTAMTRVFNEYARRINELKDKDKNLDGNDIR**

32 48 100.0% 99.5%  **LHDVTTFAGHSNGIEVDVAFQYNDQYSESILSFVNNVRTKDGGTHEVGFKTAMTRVFNEYARRINELKDKDKNLDGNDIR**

33 ABKVF 100.0% 99.4%  **LHDVTTFAGHSNGIEVDVAFQYNDQYSESILSFVNNVRTKDGGTHEVGFKTAMTRVFNEYARRINELKDKDKNLDGNDIR**

34 CICARIA 100.0% 100.0%  **LHDVTTFAGHSNGIEVDVAFQYNDQYSESILSFVNNVRTKDGGTHEVGFKTAMTRVFNEYARRINELKDKDKNLDGNDIR**

35 SKN25lux 100.0% 99.8%  **LHDVTTFAGHSNGIEVDVAFQYNDQYSESILSFVNNVRTKDGGTHEVGFKTAMTRVFNEYARRINELKDKDKNLDGNDIR**

36 HMSC074F11 100.0% 99.4%  **LHDVTTFAGHSNGIEVDVAFQYNDQYSESILSFVNNVRTKDGGTHEVGFKTAMTRVFNEYARRINELKDKDKNLDGNDIR**

37 HMSC068G11 100.0% 99.4%  **LHDVTTFAGHSNGIEVDVAFQYNDQYSESILSFVNNVRTKDGGTHEVGFKTAMTRVFNEYARRINELKDKDKNLDGNDIR**

38 NIHLM037 56.2% 99.2%  **----------------------------------------------------MTRVFNEYARRINELKDKDKNLDGNDIR**

39 NIHLM015 100.0% 99.4%  **LHDVTTFAGHSNGIEVDVAFQYNDQYSESILSFVNNVRTKDGGTHEVGFKTAMTRVFNEYARRINELKDKDKNLDGNDIR**

consensus/100%  **....................................................MTRVFNEYARRINELKDKDKNLDGNDIR**

consensus/90%  **LHDVTTFAGHSNGIEVDVAFQYNDQYSESILSFVNNVRTKDGGTHEVGFKTAMTRVFNEYARRINELKDKDKNLDGNDIR**

consensus/80%  **LHDVTTFAGHSNGIEVDVAFQYNDQYSESILSFVNNVRTKDGGTHEVGFKTAMTRVFNEYARRINELKDKDKNLDGNDIR**

consensus/70%  **LHDVTTFAGHSNGIEVDVAFQYNDQYSESILSFVNNVRTKDGGTHEVGFKTAMTRVFNEYARRINELKDKDKNLDGNDIR**

cov pid **321**  **. . : . . . . 4** **400**

1 ATCC35984 100.0% 100.0%  **EGLTAIISVRIPEELLQFEGQTKSKLGTSEARSAVDSVVSEKLPYYLEEKGQLSKSLVKKAIKAQQAREAARKAREDARS**

2 ATCC12228 100.0% 100.0%  **EGLTAIISVRIPEELLQFEGQTKSKLGTSEARSAVDSVVSEKLPYYLEEKGQLSKSLVKKAIKAQQAREAARKAREDARS**

3 36AM 100.0% 99.5%  **EGLTAIISVRIPEELLQFEGQTKSKLGTSEARSAVDSVVSEKLPYYLEEKGQLSKSLVKKAIKAQQAREAARKAREDARS**

4 48AF 100.0% 100.0%  **EGLTAIISVRIPEELLQFEGQTKSKLGTSEARSAVDSVVSEKLPYYLEEKGQLSKSLVKKAIKAQQAREAARKAREDARS**

5 54AF 100.0% 99.4%  **EGLTAIISVRIPEELLQFEGQTKSKLGTSEARSAVDSVVSEKLPYYLEEKGQISKSLVKKAIKAQQAREAARKAREDARS**

6 785_SEPI 100.0% 99.5%  **EGLTAIISVRIPEELLQFEGQTKSKLGTSEARSAVDSVVSEKLPYYLEEKGQLSKSLVKKAIKAQQAREAARKAREDARS**

7 VSE49 100.0% 99.4%  **EGLTAIISVRIPEELLQFEGQTKSKLGTSEARSAVDSVVSEKLPYYLEEKGQLSKSLVKKAIKAQQAREAARKAREDARS**

8 VSE57 100.0% 99.4%  **EGLTAIISVRIPEELLQFEGQTKSKLGTSEARSAVDSVVSEKLPYYLEEKGQLSKSLVKKAIKAQQAREAARKAREDARS**

9 SE45 100.0% 99.4%  **EGLTAIISVRIPEELLQFEGQTKSKLGTSEARSAVDSVVSEKLPYYLEEKGQLSKSLVKKAIKAQQAREAARKAREDARS**

10 SE40 100.0% 99.4%  **EGLTAIISVRIPEELLQFEGQTKSKLGTSEARSAVDSVVSEKLPYYLEEKGQLSKSLVKKAIKAQQAREAARKAREDARS**

11 32A 100.0% 99.4%  **EGLTAIISVRIPEELLQFEGQTKSKLGTSEARSAVDSVVSEKLPYYLEEKGQLSKSLVKKAIKAQQAREAARKAREDARS**

12 HD66 100.0% 100.0%  **EGLTAIISVRIPEELLQFEGQTKSKLGTSEARSAVDSVVSEKLPYYLEEKGQLSKSLVKKAIKAQQAREAARKAREDARS**

13 HD43 91.0% 100.0%  **EGLTAIISVRIPEELLQFEGQTKSKLGTSEARSAVDSVVSEKLPYYLEEKGQLSKSLVKKAIKAQQAREAARKAREDARS**

14 HD33 100.0% 100.0%  **EGLTAIISVRIPEELLQFEGQTKSKLGTSEARSAVDSVVSEKLPYYLEEKGQLSKSLVKKAIKAQQAREAARKAREDARS**

15 DE0525 100.0% 99.4%  **EGLTAIISVRIPEELLQFEGQTKSKLGTSEARSAVDSVVSEKLPYYLEEKGQLSKSLVKKAIKAQQAREAARKAREDARS**

16 4928STDY7071543 100.0% 99.4%  **EGLTAIISVRIPEELLQFEGQTKSKLGTSEARSAVDSVVSEKLPYYLEEKGQLSKSLVKKAIKAQQAREAARKAREDARS**

17 BAV2502 100.0% 99.5%  **EGLTAIISVRIPEELLQFEGQTKSKLGTSEARSAVDSVVSEKLPYYLEEKGQLSKSLVKKAIKAQQAREAARKAREDARS**

18 APC3784 100.0% 99.5%  **EGLTAIISVRIPEELLQFEGQTKSKLGTSEARSAVDSVVSEKLPYYLEEKGQLSKSLVKKAIKAQQAREAARKAREDARS**

19 SESURV_p1_0557 100.0% 99.8%  **EGLTAIISVRIPEELLQFEGQTKSKLGTSEARSAVDSVVSEKLPYYLEEKGQLSKSLVKKAIKAQQAREAARKAREDARS**

20 SESURV_p1_0612 100.0% 99.7%  **EGLTAIISVRIPEELLQFEGQTKSKLGTSEARSAVDSVVSEKLPYYLEEKGQLSKSLVKKAIKAQQAREAARKAREDARS**

21 SESURV_p4_1553 100.0% 99.8%  **EGLTAIISVRIPEELLQFEGQTKSKLGTSEARSAVDSVVSEKLPYYLEEKGQLSKSLVKKAIKAQQAREAARKAREDARS**

22 SESURV_p1_1200 100.0% 99.8%  **EGLTAIISVRIPEELLQFEGQTKSKLGTSEARSAVDSVVSEKLPYYLEEKGQLSKSLVKKAIKAQQAREAARKAREDARS**

23 SESURV_p3_0825 100.0% 99.7%  **EGLTAIISVRIPEELLQFEGQTKSKLGTSEARSAVDSVVSEKLPYYLEEKGQLSKSLVKKAIKAQQAREAARKAREDARS**

24 JH 100.0% 99.4%  **EGLTAIISVRIPEELLQFEGQTKSKLGTSEARSAVDSVVSEKLPYYLEEKGQLSKSLVKKAIKAQQAREAARKAREDARS**

25 Z0118SE0132 100.0% 99.7%  **EGLTAIISVRIPEELLQFEGQTKSKLGTSEARSAVDSVVSEKLPYYLEEKGQLSKSLVKKAIKAQQAREAARKAREDARS**

26 R5981 100.0% 99.8%  **EGLTAIISVRIPEELLQFEGQTKSKLGTSEARSAVDSVVSEKLPYYLEEKGQLSKSLVKKAIKAQQAREAARKAREDARS**

27 JH-S-3 100.0% 99.4%  **EGLTAIISVRIPEELLQFEGQTKSKLGTSEARSAVDSVVSEKLPYYLEEKGQLSKSLVKKAIKAQQAREAARKAREDARS**

28 JH-S-1 100.0% 99.4%  **EGLTAIISVRIPEELLQFEGQTKSKLGTSEARSAVDSVVSEKLPYYLEEKGQLSKSLVKKAIKAQQAREAARKAREDARS**

29 s2 100.0% 99.5%  **EGLTAIISVRIPEELLQFEGQTKSKLGTSEARSAVDSVVSEKLPYYLEEKGQLSKSLVKKAIKAQQAREAARKAREDARS**

30 S43 100.0% 99.7%  **EGLTAIISVRIPEELLQFEGQTKSKLGTSEARSAVDSVVSEKLPYYLEEKGQLSKSLVKKAIKAQQAREAARKAREDARS**

31 S17W 100.0% 99.4%  **EGLTAIISVRIPEELLQFEGQTKSKLGTSEARSAVDSVVSEKLPYYLEEKGQLSKSLVKKAIKAQQAREAARKAREDARS**

32 48 100.0% 99.5%  **EGLTAIISVRIPEELLQFEGQTKSKLGTSEARSAVDSVVSEKLPYYLEEKGQLSKSLVKKAIKAQQAREAARKAREDARS**

33 ABKVF 100.0% 99.4%  **EGLTAIISVRIPEELLQFEGQTKSKLGTSEARSAVDSVVSEKLPYYLEEKGQLSKSLVKKAIKAQQAREAARKAREDARS**

34 CICARIA 100.0% 100.0%  **EGLTAIISVRIPEELLQFEGQTKSKLGTSEARSAVDSVVSEKLPYYLEEKGQLSKSLVKKAIKAQQAREAARKAREDARS**

35 SKN25lux 100.0% 99.8%  **EGLTAIISVRIPEEFLQFEGQTKSKLGTSEARSAVDSVVSEKLPYYLEEKGQLSKSLVKKAIKAQQAREAARKAREDARS**

36 HMSC074F11 100.0% 99.4%  **EGLTAIISVRIPEELLQFEGQTKSKLGTSEARSAVDSVVSEKLPYYLEEKGQLSKSLVKKAIKAQQAREAARKAREDARS**

37 HMSC068G11 100.0% 99.4%  **EGLTAIISVRIPEELLQFEGQTKSKLGTSEARSAVDSVVSEKLPYYLEEKGQLSKSLVKKAIKAQQAREAARKAREDARS**

38 NIHLM037 56.2% 99.2%  **EGLTAIISVRIPEELLQFEGQTKSKLGTSEARSAVDSVVSEKLPYYLEEKGQLSKSLVKKAIKAQQAREAARKAREDARS**

39 NIHLM015 100.0% 99.4%  **EGLTAIISVRIPEELLQFEGQTKSKLGTSEARSAVDSVVSEKLPYYLEEKGQLSKSLVKKAIKAQQAREAARKAREDARS**

consensus/100%  **EGLTAIISVRIPEEhLQFEGQTKSKLGTSEARSAVDSVVSEKLPYYLEEKGQlSKSLVKKAIKAQQAREAARKAREDARS**

consensus/90%  **EGLTAIISVRIPEELLQFEGQTKSKLGTSEARSAVDSVVSEKLPYYLEEKGQLSKSLVKKAIKAQQAREAARKAREDARS**

consensus/80%  **EGLTAIISVRIPEELLQFEGQTKSKLGTSEARSAVDSVVSEKLPYYLEEKGQLSKSLVKKAIKAQQAREAARKAREDARS**

consensus/70%  **EGLTAIISVRIPEELLQFEGQTKSKLGTSEARSAVDSVVSEKLPYYLEEKGQLSKSLVKKAIKAQQAREAARKAREDARS**

cov pid **401**  **. . . . : . . .** **480**

1 ATCC35984 100.0% 100.0%  **GKKNKRKDTLLSGKLTPAQSKNTDKNELYLVEGDSAGGSAKLGRDRKFQAILPLRGKVINTEKARLEDIFKNEEINTIIH**

2 ATCC12228 100.0% 100.0%  **GKKNKRKDTLLSGKLTPAQSKNTDKNELYLVEGDSAGGSAKLGRDRKFQAILPLRGKVINTEKARLEDIFKNEEINTIIH**

3 36AM 100.0% 99.5%  **GKKSKRKDTLLSGKLTPAQSKNTDKNELYLVEGDSAGGSAKLGRDRKFQAILPLRGKVINTEKARLEDIFKNEEINTIIH**

4 48AF 100.0% 100.0%  **GKKNKRKDTLLSGKLTPAQSKNTDKNELYLVEGDSAGGSAKLGRDRKFQAILPLRGKVINTEKARLEDIFKNEEINTIIH**

5 54AF 100.0% 99.4%  **GKKSKRKDTLLSGKLTPAQSKNTDKNELYLVEGDSAGGSAKLGRDRKFQAILPLRGKVINTEKARLEDIFKNEEINTIIH**

6 785_SEPI 100.0% 99.5%  **GKKSKRKDTLLSGKLTPAQSKNTDKNELYLVEGDSAGGSAKLGRDRKFQAILPLRGKVINTEKARLEDIFKNEEINTIIH**

7 VSE49 100.0% 99.4%  **GKKSKRKDTLLSGKLTPAQSKNTDKNELYLVEGDSAGGSAKLGRDRKFQAILPLRGKVINTEKARLEDIFKNEEINTIIH**

8 VSE57 100.0% 99.4%  **GKKSKRKDTLLSGKLTPAQSKNTDKNELYLVEGDSAGGSAKLGRDRKFQAILPLRGKVINTEKARLEDIFKNEEINTIIH**

9 SE45 100.0% 99.4%  **GKKSKRKDTLLSGKLTPAQSKNTDKNELYLVEGDSAGGSAKLGRDRKFQAILPLRGKVINTEKARLEDIFKNEEINTIIH**

10 SE40 100.0% 99.4%  **GKKSKRKDTLLSGKLTPAQSKNTDKNELYLVEGDSAGGSAKLGRDRKFQAILPLRGKVINTEKARLEDIFKNEEINTIIH**

11 32A 100.0% 99.4%  **GKKSKRKDTLLSGKLTPAQSKNTDKNELYLVEGDSAGGSAKLGRDRKFQAILPLRGKVINTEKARLEDIFKNEEINTIIH**

12 HD66 100.0% 100.0%  **GKKNKRKDTLLSGKLTPAQSKNTDKNELYLVEGDSAGGSAKLGRDRKFQAILPLRGKVINTEKARLEDIFKNEEINTIIH**

13 HD43 91.0% 100.0%  **GKKNKRKDTLLSGKLTPAQSKNTDKNELYLVEGDSAGGSAKLGRDRKFQAILPLRGKVINTEKARLEDIFKNEEINTIIH**

14 HD33 100.0% 100.0%  **GKKNKRKDTLLSGKLTPAQSKNTDKNELYLVEGDSAGGSAKLGRDRKFQAILPLRGKVINTEKARLEDIFKNEEINTIIH**

15 DE0525 100.0% 99.4%  **GKKSKRKDTLLSGKLTPAQSKNTDKNELYLVEGDSAGGSAKLGRDRKFQAILPLRGKVINTEKARLEDIFKNEEINTIIH**

16 4928STDY7071543 100.0% 99.4%  **GKKSKRKDTLLSGKLTPAQSKNTDKNELYLVEGDSAGGSAKLGRDRKFQAILPLRGKVINTEKARLEDIFKNEEINTIIH**

17 BAV2502 100.0% 99.5%  **GKKSKRKDTLLSGKLTPAQSKNTDKNELYLVEGDSAGGSAKLGRDRKFQAILPLRGKVINTEKARLEDIFKNEEINTIIH**

18 APC3784 100.0% 99.5%  **GKKSKRKDTLLSGKLTPAQSKNTDKNELYLVEGDSAGGSAKLGRDRKFQAILPLRGKVINTEKARLEDIFKNEEINTIIH**

19 SESURV_p1_0557 100.0% 99.8%  **GKKNKRKDTLLSGKLTPAQSKNTDKNELYLVEGDSAGGSAKLGRDRKFQAILPLRGKVINTEKARLEDIFKNEEINTIIH**

20 SESURV_p1_0612 100.0% 99.7%  **GKKSKRKDTLLSGKLTPAQSKNTDKNELYLVEGDSAGGSAKLGRDRKFQAILPLRGKVINTEKARLEDIFKNEEINTIIH**

21 SESURV_p4_1553 100.0% 99.8%  **GKKNKRKDTLLSGKLTPAQSKNTDKNELYLVEGDSAGGSAKLGRDRKFQAILPLRGKVINTEKARLEDIFKNEEINTIIH**

22 SESURV_p1_1200 100.0% 99.8%  **GKKNKRKDTLLSGKLTPAQSKNTDKNELYLVEGDSAGGSAKLGRDRKFQAILPLRGKVINTEKARLEDIFKNEEINTIIH**

23 SESURV_p3_0825 100.0% 99.7%  **GKKNKRKDTLLSGKLTPAQSKNTDKNELYLVEGDSAGGSAKLGRDRKFQAILPLRGKVINTEKARLEDIFKNEEINTIIH**

24 JH 100.0% 99.4%  **GKKSKRKDTLLSGKLTPAQSKNTDKNELYLVEGDSAGGSAKLGRDRKFQAILPLRGKVINTEKARLEDIFKNEEINTIIH**

25 Z0118SE0132 100.0% 99.7%  **GKKNKRKDTLLSGKLTPAQSKNTDKNELYLVEGDSAGGSAKLGRDRKFQAILPLRGKVINTEKARLEDIFKNEEINTIIH**

26 R5981 100.0% 99.8%  **GKKNKRKDTLLSGKLTPAQSKNTDKNELYLVEGDSAGGSAKLGRDRKFQAILPLRGKVINTEKARLEDIFKNEEINTIIH**

27 JH-S-3 100.0% 99.4%  **GKKSKRKDTLLSGKLTPAQSKNTDKNELYLVEGDSAGGSAKLGRDRKFQAILPLRGKVINTEKARLEDIFKNEEINTIIH**

28 JH-S-1 100.0% 99.4%  **GKKSKRKDTLLSGKLTPAQSKNTDKNELYLVEGDSAGGSAKLGRDRKFQAILPLRGKVINTEKARLEDIFKNEEINTIIH**

29 s2 100.0% 99.5%  **GKKSKRKDTLLSGKLTPAQSKNTDKNELYLVEGDSAGGSAKLGRDRKFQAILPLRGKVINTEKARLEDIFKNEEINTIIH**

30 S43 100.0% 99.7%  **GKKSKRKDTLLSGKLTPAQSKNTDKNELYLVEGDSAGGSAKLGRDRKFQAILPLRGKVINTEKARLEDIFKNEEINTIIH**

31 S17W 100.0% 99.4%  **GKKSKRKDTLLSGKLTPAQSKNTDKNELYLVEGDSAGGSAKLGRDRKFQAILPLRGKVINTEKARLEDIFKNEEINTIIH**

32 48 100.0% 99.5%  **GKKSKRKDTLLSGKLTPAQSKNTDKNELYLVEGDSAGGSAKLGRDRKFQAILPLRGKVINTEKARLEDIFKNEEINTIIH**

33 ABKVF 100.0% 99.4%  **GKKSKRKDTLLSGKLTPAQSKNTDKNELYLVEGDSAGGSAKLGRDRKFQAILPLRGKVINTEKARLEDIFKNEEINTIIH**

34 CICARIA 100.0% 100.0%  **GKKNKRKDTLLSGKLTPAQSKNTDKNELYLVEGDSAGGSAKLGRDRKFQAILPLRGKVINTEKARLEDIFKNEEINTIIH**

35 SKN25lux 100.0% 99.8%  **GKKNKRKDTLLSGKLTPAQSKNTDKNELYLVEGDSAGGSAKLGRDRKFQAILPLRGKVINTEKARLEDIFKNEEINTIIH**

36 HMSC074F11 100.0% 99.4%  **GKKSKRKDTLLSGKLTPAQSKNTDKNELYLVEGDSAGGSAKLGRDRKFQAILPLRGKVINTEKARLEDIFKNEEINTIIH**

37 HMSC068G11 100.0% 99.4%  **GKKSKRKDTLLSGKLTPAQSKNTDKNELYLVEGDSAGGSAKLGRDRKFQAILPLRGKVINTEKARLEDIFKNEEINTIIH**

38 NIHLM037 56.2% 99.2%  **GKKSKRKDTLLSGKLTPAQSKNTDKNELYLVEGDSAGGSAKLGRDRKFQAILPLRGKVINTEKARLEDIFKNEEINTIIH**

39 NIHLM015 100.0% 99.4%  **GKKSKRKDTLLSGKLTPAQSKNTDKNELYLVEGDSAGGSAKLGRDRKFQAILPLRGKVINTEKARLEDIFKNEEINTIIH**

consensus/100%  **GKKsKRKDTLLSGKLTPAQSKNTDKNELYLVEGDSAGGSAKLGRDRKFQAILPLRGKVINTEKARLEDIFKNEEINTIIH**

consensus/90%  **GKKsKRKDTLLSGKLTPAQSKNTDKNELYLVEGDSAGGSAKLGRDRKFQAILPLRGKVINTEKARLEDIFKNEEINTIIH**

consensus/80%  **GKKsKRKDTLLSGKLTPAQSKNTDKNELYLVEGDSAGGSAKLGRDRKFQAILPLRGKVINTEKARLEDIFKNEEINTIIH**

consensus/70%  **GKKsKRKDTLLSGKLTPAQSKNTDKNELYLVEGDSAGGSAKLGRDRKFQAILPLRGKVINTEKARLEDIFKNEEINTIIH**

cov pid **481**  **. 5 . . . . : .** **560**

1 ATCC35984 100.0% 100.0%  **TIGAGVGTDFKIEDSNYNRIIIMTDADTDGAHIQVLLLTFFFKYMKPLVQAGRVFIALPPLYKLEKGKGKNKKVEYAWTD**

2 ATCC12228 100.0% 100.0%  **TIGAGVGTDFKIEDSNYNRIIIMTDADTDGAHIQVLLLTFFFKYMKPLVQAGRVFIALPPLYKLEKGKGKNKKVEYAWTD**

3 36AM 100.0% 99.5%  **TIGAGVGTDFKIEDSNYNRIIIMTDADTDGAHIQVLLLTFFFKYMKPLVQAGRVFIALPPLYKLEKGKGKNKKVEYAWTD**

4 48AF 100.0% 100.0%  **TIGAGVGTDFKIEDSNYNRIIIMTDADTDGAHIQVLLLTFFFKYMKPLVQAGRVFIALPPLYKLEKGKGKNKKVEYAWTD**

5 54AF 100.0% 99.4%  **TIGAGVGTDFKIEDSNYNRIIIMTDADTDGAHIQVLLLTFFFKYMKPLVQAGRVFIALPPLYKLEKGKGKNKKVEYAWTD**

6 785_SEPI 100.0% 99.5%  **TIGAGVGTDFKIEDSNYNRIIIMTDADTDGAHIQVLLLTFFFKYMKPLVQAGRVFIALPPLYKLEKGKGKNKKVEYAWTD**

7 VSE49 100.0% 99.4%  **TIGAGVGTDFKIEDSNYNRIIIMTDADTDGAHIQVLLLTFFFKYMKPLVQAGRVFIALPPLYKLEKGKGKNKKVEYAWTD**

8 VSE57 100.0% 99.4%  **TIGAGVGTDFKIEDSNYNRIIIMTDADTDGAHIQVLLLTFFFKYMKPLVQAGRVFIALPPLYKLEKGKGKNKKVEYAWTD**

9 SE45 100.0% 99.4%  **TIGAGVGTDFKIEDSNYNRIIIMTDADTDGAHIQVLLLTFFFKYMKPLVQAGRVFIALPPLYKLEKGKGKNKKVEYAWTD**

10 SE40 100.0% 99.4%  **TIGAGVGTDFKIEDSNYNRIIIMTDADTDGAHIQVLLLTFFFKYMKPLVQAGRVFIALPPLYKLEKGKGKNKKVEYAWTD**

11 32A 100.0% 99.4%  **TIGAGVGTDFKIEDSNYNRIIIMTDADTDGAHIQVLLLTFFFKYMKPLVQAGRVFIALPPLYKLEKGKGKNKKVEYAWTD**

12 HD66 100.0% 100.0%  **TIGAGVGTDFKIEDSNYNRIIIMTDADTDGAHIQVLLLTFFFKYMKPLVQAGRVFIALPPLYKLEKGKGKNKKVEYAWTD**

13 HD43 91.0% 100.0%  **TIGAGVGTDFKIEDSNYNRIIIMTDADTDGAHIQVLLLTFFFKYMKPLVQAGRVFIALPPLYKLEKGKGKNKKVEYAWTD**

14 HD33 100.0% 100.0%  **TIGAGVGTDFKIEDSNYNRIIIMTDADTDGAHIQVLLLTFFFKYMKPLVQAGRVFIALPPLYKLEKGKGKNKKVEYAWTD**

15 DE0525 100.0% 99.4%  **TIGAGVGTDFKIEDSNYNRIIIMTDADTDGAHIQVLLLTFFFKYMKPLVQAGRVFIALPPLYKLEKGKGKNKKVEYAWTD**

16 4928STDY7071543 100.0% 99.4%  **TIGAGVGTDFKIEDSNYNRIIIMTDADTDGAHIQVLLLTFFFKYMKPLVQAGRVFIALPPLYKLEKGKGKNKKVEYAWTD**

17 BAV2502 100.0% 99.5%  **TIGAGVGTDFKIEDSNYNRIIIMTDADTDGAHIQVLLLTFFFKYMKPLVQAGRVFIALPPLYKLEKGKGKNKKVEYAWTD**

18 APC3784 100.0% 99.5%  **TIGAGVGTDFKIEDSNYNRIIIMTDADTDGAHIQVLLLTFFFKYMKPLVQAGRVFIALPPLYKLEKGKGKNKKVEYAWTD**

19 SESURV_p1_0557 100.0% 99.8%  **TIGAGVGTDFKIEDSNYNRIIIMTDADTDGAHIQVLLLTFFFKYMKPLVQAGRVFIALPPLYKLEKGKGKNKKVEYAWTD**

20 SESURV_p1_0612 100.0% 99.7%  **TIGAGVGTDFKIEDSNYNRIIIMTDADTDGAHIQVLLLTFFFKYMKPLVQAGRVFIALPPLYKLEKGKGKNKKVEYAWTD**

21 SESURV_p4_1553 100.0% 99.8%  **TIGAGVGTDFKIEDSNYNRIIIMTDADTDGAHIQVLLLTFFFKYMKPLVQAGRVFIALPPLYKLEKGKGKNKKVEYAWTD**

22 SESURV_p1_1200 100.0% 99.8%  **TIGAGVGTDFKIEDSNYNRIIIMTDADTDGAHIQVLLLTFFFKYMKPLVQAGRVFIALPPLYKLEKGKGKNKKVEYAWTD**

23 SESURV_p3_0825 100.0% 99.7%  **TIGAGVGTDFKIEDSNYNRIIIMTDADTDGAHIQVLLLTFFFKYMKPLVQAGRVFIALPPLYKLEKGKGKNKKVEYAWTD**

24 JH 100.0% 99.4%  **TIGAGVGTDFKIEDSNYNRIIIMTDADTDGAHIQVLLLTFFFKYMKPLVQAGRVFIALPPLYKLEKGKGKNKKVEYAWTD**

25 Z0118SE0132 100.0% 99.7%  **TIGAGVGTDFKIEDSNYNRIIIMTDADTDGAHIQVLLLTFFFKYMKPLVQAGRVFIALPPLYKLEKGKGKNKKVEYAWTD**

26 R5981 100.0% 99.8%  **TIGAGVGTDFKIEDSNYNRIIIMTDADTDGAHIQVLLLTFFFKYMKPLVQAGRVFIALPPLYKLEKGKGKNKKVEYAWTD**

27 JH-S-3 100.0% 99.4%  **TIGAGVGTDFKIEDSNYNRIIIMTDADTDGAHIQVLLLTFFFKYMKPLVQAGRVFIALPPLYKLEKGKGKNKKVEYAWTD**

28 JH-S-1 100.0% 99.4%  **TIGAGVGTDFKIEDSNYNRIIIMTDADTDGAHIQVLLLTFFFKYMKPLVQAGRVFIALPPLYKLEKGKGKNKKVEYAWTD**

29 s2 100.0% 99.5%  **TIGAGVGTDFKIEDSNYNRIIIMTDADTDGAHIQVLLLTFFFKYMKPLVQAGRVFIALPPLYKLEKGKGKNKKVEYAWTD**

30 S43 100.0% 99.7%  **TIGAGVGTDFKIEDSNYNRIIIMTDADTDGAHIQVLLLTFFFKYMKPLVQAGRVFIALPPLYKLEKGKGKNKKVEYAWTD**

31 S17W 100.0% 99.4%  **TIGAGVGTDFKIEDSNYNRIIIMTDADTDGAHIQVLLLTFFFKYMKPLVQAGRVFIALPPLYKLEKGKGKNKKVEYAWTD**

32 48 100.0% 99.5%  **TIGAGVGTDFKIEDSNYNRIIIMTDADTDGAHIQVLLLTFFFKYMKPLVQAGRVFIALPPLYKLEKGKGKNKKVEYAWTD**

33 ABKVF 100.0% 99.4%  **TIGAGVGTDFKIEDSNYNRIIIMTDADTDGAHIQVLLLTFFFKYMKPLVQAGRVFIALPPLYKLEKGKGKNKKVEYAWTD**

34 CICARIA 100.0% 100.0%  **TIGAGVGTDFKIEDSNYNRIIIMTDADTDGAHIQVLLLTFFFKYMKPLVQAGRVFIALPPLYKLEKGKGKNKKVEYAWTD**

35 SKN25lux 100.0% 99.8%  **TIGAGVGTDFKIEDSNYNRIIIMTDADTDGAHIQVLLLTFFFKYMKPLVQAGRVFIALPPLYKLEKGKGKNKKVEYAWTD**

36 HMSC074F11 100.0% 99.4%  **TIGAGVGTDFKIEDSNYNRIIIMTDADTDGAHIQVLLLTFFFKYMKPLVQAGRVFIALPPLYKLEKGKGKNKKVEYAWTD**

37 HMSC068G11 100.0% 99.4%  **TIGAGVGTDFKIEDSNYNRIIIMTDADTDGAHIQVLLLTFFFKYMKPLVQAGRVFIALPPLYKLEKGKGKNKKVEYAWTD**

38 NIHLM037 56.2% 99.2%  **TIGAGVGTDFKIEDSNYNRIIIMTDADTDGAHIQVLLLTFFFKYMKPLVQAGRVFIALPPLYKLEKGKGKNKKVEYAWTD**

39 NIHLM015 100.0% 99.4%  **TIGAGVGTDFKIEDSNYNRIIIMTDADTDGAHIQVLLLTFFFKYMKPLVQAGRVFIALPPLYKLEKGKGKNKKVEYAWTD**

consensus/100%  **TIGAGVGTDFKIEDSNYNRIIIMTDADTDGAHIQVLLLTFFFKYMKPLVQAGRVFIALPPLYKLEKGKGKNKKVEYAWTD**

consensus/90%  **TIGAGVGTDFKIEDSNYNRIIIMTDADTDGAHIQVLLLTFFFKYMKPLVQAGRVFIALPPLYKLEKGKGKNKKVEYAWTD**

consensus/80%  **TIGAGVGTDFKIEDSNYNRIIIMTDADTDGAHIQVLLLTFFFKYMKPLVQAGRVFIALPPLYKLEKGKGKNKKVEYAWTD**

consensus/70%  **TIGAGVGTDFKIEDSNYNRIIIMTDADTDGAHIQVLLLTFFFKYMKPLVQAGRVFIALPPLYKLEKGKGKNKKVEYAWTD**

cov pid **561**  **. . . 6 . . . .** **640**

1 ATCC35984 100.0% 100.0%  **EELENLQKQLGKGFILQRYKGLGEMNPEQLWETTMNPETRTLIRVQVEDEVRSSKRVTTLMGDKVAPRREWIEKHVEFGM**

2 ATCC12228 100.0% 100.0%  **EELENLQKQLGKGFILQRYKGLGEMNPEQLWETTMNPETRTLIRVQVEDEVRSSKRVTTLMGDKVAPRREWIEKHVEFGM**

3 36AM 100.0% 99.5%  **EELENLQNQLGKGFTLQRYKGLGEMNPEQLWETTMNPETRTLIRVQVEDEVRSSKRVTTLMGDKVAPRREWIEKHVEFGM**

4 48AF 100.0% 100.0%  **EELENLQKQLGKGFILQRYKGLGEMNPEQLWETTMNPETRTLIRVQVEDEVRSSKRVTTLMGDKVAPRREWIEKHVEFGM**

5 54AF 100.0% 99.4%  **EELENLQNQLGKGFTLQRYKGLGEMNPEQLWETTMNPETRTLIRVQVEDEVRSSKRVTTLMGDKVAPRREWIEKHVEFGM**

6 785_SEPI 100.0% 99.5%  **EELENLQNQLGKGFTLQRYKGLGEMNPEQLWETTMNPETRTLIRVQVEDEVRSSKRVTTLMGDKVAPRREWIEKHVEFGM**

7 VSE49 100.0% 99.4%  **EELENLQNQLGKGFTLQRYKGLGEMNPEQLWETTMNPETRTLIRVQVEDEVRSSKRVTTLMGDKVAPRREWIEKHVEFGM**

8 VSE57 100.0% 99.4%  **EELENLQNQLGKGFTLQRYKGLGEMNPEQLWETTMNPETRTLIRVQVEDEVRSSKRVTTLMGDKVAPRREWIEKHVEFGM**

9 SE45 100.0% 99.4%  **EELENLQNQLGKGFTLQRYKGLGEMNPEQLWETTMNPETRTLIRVQVEDEVRSSKRVTTLMGDKVAPRREWIEKHVEFGM**

10 SE40 100.0% 99.4%  **EELENLQNQLGKGFTLQRYKGLGEMNPEQLWETTMNPETRTLIRVQVEDEVRSSKRVTTLMGDKVAPRREWIEKHVEFGM**

11 32A 100.0% 99.4%  **EELENLQNQLGKGFTLQRYKGLGEMNPEQLWETTMNPETRTLIRVQVEDEVRSSKRVTTLMGDKVAPRREWIEKHVEFGM**

12 HD66 100.0% 100.0%  **EELENLQKQLGKGFILQRYKGLGEMNPEQLWETTMNPETRTLIRVQVEDEVRSSKRVTTLMGDKVAPRREWIEKHVEFGM**

13 HD43 91.0% 100.0%  **EELENLQKQLGKGFILQRYKGLGEMNPEQLWETTMNPETRTLIRVQVEDEVRSSKRVTTLMGDKVAPRREWIEKHVEFGM**

14 HD33 100.0% 100.0%  **EELENLQKQLGKGFILQRYKGLGEMNPEQLWETTMNPETRTLIRVQVEDEVRSSKRVTTLMGDKVAPRREWIEKHVEFGM**

15 DE0525 100.0% 99.4%  **EELENLQNQLGKGFTLQRYKGLGEMNPEQLWETTMNPETRTLIRVQVEDEVRSSKRVTTLMGDKVAPRREWIEKHVEFGM**

16 4928STDY7071543 100.0% 99.4%  **EELENLQNQLGKGFTLQRYKGLGEMNPEQLWETTMNPETRTLIRVQVEDEVRSSKRVTTLMGDKVAPRREWIEKHVEFGM**

17 BAV2502 100.0% 99.5%  **EELENLQNQLGKGFTLQRYKGLGEMNPEQLWETTMNPETRTLIRVQVEDEVRSSKRVTTLMGDKVAPRREWIEKHVEFGM**

18 APC3784 100.0% 99.5%  **EELENLQNQLGKGFTLQRYKGLGEMNPEQLWETTMNPETRTLIRVQVEDEVRSSKRVTTLMGDKVAPRREWIEKHVEFGM**

19 SESURV_p1_0557 100.0% 99.8%  **EELENLQKQLGKGFILQRYKGLGEMNPEQLWETTMNPETRTLIRVQVEDEVRSSKRVTTLMGDKVAPRREWIEKHVEFGM**

20 SESURV_p1_0612 100.0% 99.7%  **EELENLQKQLGKGFTLQRYKGLGEMNPEQLWETTMNPETRTLIRVQVEDEVRSSKRVTTLMGDKVAPRREWIEKHVEFGM**

21 SESURV_p4_1553 100.0% 99.8%  **EELENLQKQLGKGFILQRYKGLGEMNPEQLWETTMNPETRTLIRVQVEDEVRSSKRVTTLMGDKVAPRREWIEKHVEFGM**

22 SESURV_p1_1200 100.0% 99.8%  **EELENLQKQLGKGFILQRYKGLGEMNPEQLWETTMNPETRTLIRVQVEDEVRSSKRVTTLMGDKVAPRREWIEKHVEFGM**

23 SESURV_p3_0825 100.0% 99.7%  **EELENLQKQLGKGFILQRYKGLGEMNPEQLWETTMNPETRTLIRVQVEDEVRSSKRVTTLMGDKVAPRREWIEKHVEFGM**

24 JH 100.0% 99.4%  **EELENLQNQLGKGFTLQRYKGLGEMNPEQLWETTMNPETRTLIRVQVEDEVRSSKRVTTLMGDKVAPRREWIEKHVEFGM**

25 Z0118SE0132 100.0% 99.7%  **EELENLQKQLGKGFTLQRYKGLGEMNPEQLWETTMNPETRTLIRVQVEDEVRSSKRVTTLMGDKVAPRREWIEKHVEFGM**

26 R5981 100.0% 99.8%  **EELENLQKQLGKGFTLQRYKGLGEMNPEQLWETTMNPETRTLIRVQVEDEVRSSKRVTTLMGDKVAPRREWIEKHVEFGM**

27 JH-S-3 100.0% 99.4%  **EELENLQNQLGKGFTLQRYKGLGEMNPEQLWETTMNPETRTLIRVQVEDEVRSSKRVTTLMGDKVAPRREWIEKHVEFGM**

28 JH-S-1 100.0% 99.4%  **EELENLQNQLGKGFTLQRYKGLGEMNPEQLWETTMNPETRTLIRVQVEDEVRSSKRVTTLMGDKVAPRREWIEKHVEFGM**

29 s2 100.0% 99.5%  **EELENLQKQLGKGFTLQRYKGLGEMNPEQLWETTMNPETRTLIRVQVEDEVRSSKRVTTLMGDKVAPRREWIEKHVEFGM**

30 S43 100.0% 99.7%  **EELENLQKQLGKGFTLQRYKGLGEMNPEQLWETTMNPETRTLIRVQVEDEVRSSKRVTTLMGDKVAPRREWIEKHVEFGM**

31 S17W 100.0% 99.4%  **EELENLQNQLGKGFTLQRYKGLGEMNPEQLWETTMNPETRTLIRVQVEDEVRSSKRVTTLMGDKVAPRREWIEKHVEFGM**

32 48 100.0% 99.5%  **EELENLQNQLGKGFTLQRYKGLGEMNPEQLWETTMNPETRTLIRVQVEDEVRSSKRVTTLMGDKVAPRREWIEKHVEFGM**

33 ABKVF 100.0% 99.4%  **EELENLQNQLGKGFTLQRYKGLGEMNPEQLWETTMNPETRTLIRVQVEDEVRSSKRVTTLMGDKVAPRREWIEKHVEFGM**

34 CICARIA 100.0% 100.0%  **EELENLQKQLGKGFILQRYKGLGEMNPEQLWETTMNPETRTLIRVQVEDEVRSSKRVTTLMGDKVAPRREWIEKHVEFGM**

35 SKN25lux 100.0% 99.8%  **EELENLQKQLGKGFILQRYKGLGEMNPEQLWETTMNPETRTLIRVQVEDEVRSSKRVTTLMGDKVAPRREWIEKHVEFGM**

36 HMSC074F11 100.0% 99.4%  **EELENLQNQLGKGFTLQRYKGLGEMNPEQLWETTMNPETRTLIRVQVEDEVRSSKRVTTLMGDKVAPRREWIEKHVEFGM**

37 HMSC068G11 100.0% 99.4%  **EELENLQNQLGKGFTLQRYKGLGEMNPEQLWETTMNPETRTLIRVQVEDEVRSSKRVTTLMGDKVAPRREWIEKHVEFGM**

38 NIHLM037 56.2% 99.2%  **EELENLQNQLGKGFTLQRYKGLGEMNPEQLWETTMNPETRTLIRVQVEDEVRSSKRVTTLMGDKVAPRREWIEKHVEFGM**

39 NIHLM015 100.0% 99.4%  **EELENLQNQLGKGFTLQRYKGLGEMNPEQLWETTMNPETRTLIRVQVEDEVRSSKRVTTLMGDKVAPRREWIEKHVEFGM**

consensus/100%  **EELENLQpQLGKGFhLQRYKGLGEMNPEQLWETTMNPETRTLIRVQVEDEVRSSKRVTTLMGDKVAPRREWIEKHVEFGM**

consensus/90%  **EELENLQpQLGKGFhLQRYKGLGEMNPEQLWETTMNPETRTLIRVQVEDEVRSSKRVTTLMGDKVAPRREWIEKHVEFGM**

consensus/80%  **EELENLQpQLGKGFhLQRYKGLGEMNPEQLWETTMNPETRTLIRVQVEDEVRSSKRVTTLMGDKVAPRREWIEKHVEFGM**

consensus/70%  **EELENLQpQLGKGFhLQRYKGLGEMNPEQLWETTMNPETRTLIRVQVEDEVRSSKRVTTLMGDKVAPRREWIEKHVEFGM**

cov pid **641**  **: . ]** **666**

1 ATCC35984 100.0% 100.0%  **QEDQSILDNKEVQILENEKYIEEETN**

2 ATCC12228 100.0% 100.0%  **QEDQSILDNKEVQILENEKYIEEETN**

3 36AM 100.0% 99.5%  **QEDQSILDNKEVQILENEKYIEEETN**

4 48AF 100.0% 100.0%  **QEDQSILDNKEVQILENEKYIEEETN**

5 54AF 100.0% 99.4%  **QEDQSILDNKEVQILENEKYIEEETN**

6 785_SEPI 100.0% 99.5%  **QEDQSILDNKEVQILENEKYIEEETN**

7 VSE49 100.0% 99.4%  **QEDQSILDNKEVQILENEKYIEEETN**

8 VSE57 100.0% 99.4%  **QEDQSILDNKEVQILENEKYIEEETN**

9 SE45 100.0% 99.4%  **QEDQSILDNKEVQILENEKYIEEETN**

10 SE40 100.0% 99.4%  **QEDQSILDNKEVQILENEKYIEEETN**

11 32A 100.0% 99.4%  **QEDQSILDNKEVQILENEKYIEEETN**

12 HD66 100.0% 100.0%  **QEDQSILDNKEVQILENEKYIEEETN**

13 HD43 91.0% 100.0%  **QEDQSILDNKEVQILENEKYIEEETN**

14 HD33 100.0% 100.0%  **QEDQSILDNKEVQILENEKYIEEETN**

15 DE0525 100.0% 99.4%  **QEDQSILDNKEVQILENEKYIEEETN**

16 4928STDY7071543 100.0% 99.4%  **QEDQSILDNKEVQILENEKYIEEETN**

17 BAV2502 100.0% 99.5%  **QEDQSILDNKEVQILENEKYIEEETN**

18 APC3784 100.0% 99.5%  **QEDQSILDNKEVQILENEKYIEEETN**

19 SESURV_p1_0557 100.0% 99.8%  **QEDQSILDNKEVQILENEKYIEEETN**

20 SESURV_p1_0612 100.0% 99.7%  **QEDQSILDNKEVQILENEKYIEEETN**

21 SESURV_p4_1553 100.0% 99.8%  **QEDQSILDNKEVQILENEKYIEEETN**

22 SESURV_p1_1200 100.0% 99.8%  **QEDQSILDNKEVQILENEKYIEEETN**

23 SESURV_p3_0825 100.0% 99.7%  **QEDQSILDNKEVQILENEKYIEEEMN**

24 JH 100.0% 99.4%  **QEDQSILDNKEVQILENEKYIEEETN**

25 Z0118SE0132 100.0% 99.7%  **QEDQSILDNKEVQILENEKYIEEETN**

26 R5981 100.0% 99.8%  **QEDQSILDNKEVQILENEKYIEEETN**

27 JH-S-3 100.0% 99.4%  **QEDQSILDNKEVQILENEKYIEEETN**

28 JH-S-1 100.0% 99.4%  **QEDQSILDNKEVQILENEKYIEEETN**

29 s2 100.0% 99.5%  **QEDQSILDNKEVQILENEKYIEEETN**

30 S43 100.0% 99.7%  **QEDQSILDNKEVQILENEKYIEEETN**

31 S17W 100.0% 99.4%  **QEDQSILDNKEVQILENEKYIEEETN**

32 48 100.0% 99.5%  **QEDQSILDNKEVQILENEKYIEEETN**

33 ABKVF 100.0% 99.4%  **QEDQSILDNKEVQILENEKYIEEETN**

34 CICARIA 100.0% 100.0%  **QEDQSILDNKEVQILENEKYIEEETN**

35 SKN25lux 100.0% 99.8%  **QEDQSILDNKEVQILENEKYIEEETN**

36 HMSC074F11 100.0% 99.4%  **QEDQSILDNKEVQILENEKYIEEETN**

37 HMSC068G11 100.0% 99.4%  **QEDQSILDNKEVQILENEKYIEEETN**

38 NIHLM037 56.2% 99.2%  **QEDQSILDNKEVQILENEKYIEEETN**

39 NIHLM015 100.0% 99.4%  **QEDQSILDNKEVQILENEKYIEEETN**

consensus/100%  **QEDQSILDNKEVQILENEKYIEEEhN**

consensus/90%  **QEDQSILDNKEVQILENEKYIEEETN**

consensus/80%  **QEDQSILDNKEVQILENEKYIEEETN**

consensus/70%  **QEDQSILDNKEVQILENEKYIEEETN**

**Supplementary Figure 4.** Multiple sequence alignment of the predicted amino acid sequences of ParE carried by *S. epidermidis* isolates and close genomes retrieved from the BV-BRC database compared to that of *S. epidermidis* strain ATCC®12228 and ATCC®35984. The alignment was visualized using MView version 1.63 hosted by the EMBL-EBI; cov, coverage; pid, percent identity.

1 ATCC35984 100.0% 100.0%  **MAGQVVQYGRHRKRRNYARISEVLELPNLIEIQTKSYDWFLKEGLLEMFRDISPIEDFTGNLSLEFVDYRLGEPKYDLEE**

2 ATCC12228 100.0% 100.0%  **MAGQVVQYGRHRKRRNYARISEVLELPNLIEIQTKSYDWFLKEGLLEMFRDISPIEDFTGNLSLEFVDYRLGEPKYDLEE**

3 36AM 94.9% 100.0%  **------------------------------------------------------------NLSLEFVDYRLGEPKYDLEE**

4 48AF 100.0% 100.0%  **MAGQVVQYGRHRKRRNYARISEVLELPNLIEIQTKSYDWFLKEGLLEMFRDISPIEDFTGNLSLEFVDYRLGEPKYDLEE**

5 54AF 100.0% 99.9%  **MAGQVVQYGRHRKRRNYARISEVLELPNLIEIQTKSYDWFLKEGLLEMFRDISPIEDFTGNLSLEFVDYRLGEPKYDLEE**

6 785_SEPI 100.0% 99.9%  **MAGQVVQYGRHRKRRNYARISEVLELPNLIEIQTKSYDWFLKEGLLEMFRDISPIEDFTGNLSLEFVDYRLGEPKYDLEE**

7 VSE49 100.0% 100.0%  **MAGQVVQYGRHRKRRNYARISEVLELPNLIEIQTKSYDWFLKEGLLEMFRDISPIEDFTGNLSLEFVDYRLGEPKYDLEE**

8 VSE57 100.0% 100.0%  **MAGQVVQYGRHRKRRNYARISEVLELPNLIEIQTKSYDWFLKEGLLEMFRDISPIEDFTGNLSLEFVDYRLGEPKYDLEE**

9 SE45 100.0% 100.0%  **MAGQVVQYGRHRKRRNYARISEVLELPNLIEIQTKSYDWFLKEGLLEMFRDISPIEDFTGNLSLEFVDYRLGEPKYDLEE**

10 SE40 100.0% 100.0%  **MAGQVVQYGRHRKRRNYARISEVLELPNLIEIQTKSYDWFLKEGLLEMFRDISPIEDFTGNLSLEFVDYRLGEPKYDLEE**

11 32A 100.0% 99.2%  **MAGQVVQYGRHRKRRNYARISEVLELPNLIEIQTKSYDWFLKEGLLEMFRDISPIEDFTGNLSLEFVDYRLGEPKYDLEE**

12 HD66 100.0% 100.0%  **MAGQVVQYGRHRKRRNYARISEVLELPNLIEIQTKSYDWFLKEGLLEMFRDISPIEDFTGNLSLEFVDYRLGEPKYDLEE**

13 HD43 100.0% 99.8%  **MAGQVVQYGRHRKRRNYARISEVLELPNLIEIQTKSYDWFLKEGLLEMFRDISPIEDFTGNLSLEFVDYRLGEPKYDLEE**

14 HD33 100.0% 99.9%  **MAGQVVQYGRHRKRRNYARISEVLELPNLIEIQTKSYDWFLKEGLLEMFRDISPIEDFTGNLSLEFVDYRLGEPKYDLEE**

15 DE0525 100.0% 100.0%  **MAGQVVQYGRHRKRRNYARISEVLELPNLIEIQTKSYDWFLKEGLLEMFRDISPIEDFTGNLSLEFVDYRLGEPKYDLEE**

16 4928STDY7071543 94.9% 100.0%  **------------------------------------------------------------NLSLEFVDYRLGEPKYDLEE**

17 BAV2502 94.9% 99.9%  **------------------------------------------------------------NLSLEFVDYRLGEPKYDLEE**

18 APC3784 100.0% 100.0%  **MAGQVVQYGRHRKRRNYARISEVLELPNLIEIQTKSYDWFLKEGLLEMFRDISPIEDFTGNLSLEFVDYRLGEPKYDLEE**

19 SESURV_p1_0557 100.0% 100.0%  **MAGQVVQYGRHRKRRNYARISEVLELPNLIEIQTKSYDWFLKEGLLEMFRDISPIEDFTGNLSLEFVDYRLGEPKYDLEE**

20 SESURV_p1_0612 100.0% 100.0%  **MAGQVVQYGRHRKRRNYARISEVLELPNLIEIQTKSYDWFLKEGLLEMFRDISPIEDFTGNLSLEFVDYRLGEPKYDLEE**

21 SESURV_p4_1553 100.0% 100.0%  **MAGQVVQYGRHRKRRNYARISEVLELPNLIEIQTKSYDWFLKEGLLEMFRDISPIEDFTGNLSLEFVDYRLGEPKYDLEE**

22 SESURV_p1_1200 100.0% 100.0%  **MAGQVVQYGRHRKRRNYARISEVLELPNLIEIQTKSYDWFLKEGLLEMFRDISPIEDFTGNLSLEFVDYRLGEPKYDLEE**

23 SESURV_p3_0825 100.0% 100.0%  **MAGQVVQYGRHRKRRNYARISEVLELPNLIEIQTKSYDWFLKEGLLEMFRDISPIEDFTGNLSLEFVDYRLGEPKYDLEE**

24 JH 100.0% 100.0%  **MAGQVVQYGRHRKRRNYARISEVLELPNLIEIQTKSYDWFLKEGLLEMFRDISPIEDFTGNLSLEFVDYRLGEPKYDLEE**

25 Z0118SE0132 100.0% 99.9%  **MAGQVVQYGRHRKRRNYARISEVLELPNLIEIQTKSYDWFLKEGLLEMFRDISPIEDFTGNLSLEFVDYRLGEPKYDLEE**

26 R5981 100.0% 100.0%  **MAGQVVQYGRHRKRRNYARISEVLELPNLIEIQTKSYDWFLKEGLLEMFRDISPIEDFTGNLSLEFVDYRLGEPKYDLEE**

27 JH-S-3 100.0% 100.0%  **MAGQVVQYGRHRKRRNYARISEVLELPNLIEIQTKSYDWFLKEGLLEMFRDISPIEDFTGNLSLEFVDYRLGEPKYDLEE**

28 JH-S-1 100.0% 100.0%  **MAGQVVQYGRHRKRRNYARISEVLELPNLIEIQTKSYDWFLKEGLLEMFRDISPIEDFTGNLSLEFVDYRLGEPKYDLEE**

29 s2 100.0% 99.9%  **MAGQVVQYGRHRKRRNYARISEVLELPNLIEIQTKSYDWFLKEGLLEMFRDISPIEDFTGNLSLEFVDYRLGEPKYDLEE**

30 S43 100.0% 100.0%  **MAGQVVQYGRHRKRRNYARISEVLELPNLIEIQTKSYDWFLKEGLLEMFRDISPIEDFTGNLSLEFVDYRLGEPKYDLEE**

31 S17W 100.0% 100.0%  **MAGQVVQYGRHRKRRNYARISEVLELPNLIEIQTKSYDWFLKEGLLEMFRDISPIEDFTGNLSLEFVDYRLGEPKYDLEE**

32 48 100.0% 99.9%  **MAGQVVQYGRHRKRRNYARISEVLELPNLIEIQTKSYDWFLKEGLLEMFRDISPIEDFTGNLSLEFVDYRLGEPKYDLEE**

33 ABKVF 100.0% 99.9%  **MAGQVVQYGRHRKRRNYARISEVLELPNLIEIQTKSYDWFLKEGLLEMFRDISPIEDFTGNLSLEFVDYRLGEPKYDLEE**

34 CICARIA 100.0% 100.0%  **MAGQVVQYGRHRKRRNYARISEVLELPNLIEIQTKSYDWFLKEGLLEMFRDISPIEDFTGNLSLEFVDYRLGEPKYDLEE**

35 SKN25lux 100.0% 100.0%  **MAGQVVQYGRHRKRRNYARISEVLELPNLIEIQTKSYDWFLKEGLLEMFRDISPIEDFTGNLSLEFVDYRLGEPKYDLEE**

36 HMSC074F11 100.0% 100.0%  **MAGQVVQYGRHRKRRNYARISEVLELPNLIEIQTKSYDWFLKEGLLEMFRDISPIEDFTGNLSLEFVDYRLGEPKYDLEE**

37 HMSC068G11 100.0% 100.0%  **MAGQVVQYGRHRKRRNYARISEVLELPNLIEIQTKSYDWFLKEGLLEMFRDISPIEDFTGNLSLEFVDYRLGEPKYDLEE**

38 NIHLM037 100.0% 99.9%  **MAGQVVQYGRHRKRRNYARISEVLELPNLIEIQTKSYDWFLKEGLLEMFRDISPIEDFTGNLSLEFVDYRLGEPKYDLEE**

39 NIHLM015 100.0% 100.0%  **MAGQVVQYGRHRKRRNYARISEVLELPNLIEIQTKSYDWFLKEGLLEMFRDISPIEDFTGNLSLEFVDYRLGEPKYDLEE**

consensus/100%  **............................................................NLSLEFVDYRLGEPKYDLEE**

consensus/90%  **MAGQVVQYGRHRKRRNYARISEVLELPNLIEIQTKSYDWFLKEGLLEMFRDISPIEDFTGNLSLEFVDYRLGEPKYDLEE**

consensus/80%  **MAGQVVQYGRHRKRRNYARISEVLELPNLIEIQTKSYDWFLKEGLLEMFRDISPIEDFTGNLSLEFVDYRLGEPKYDLEE**

consensus/70%  **MAGQVVQYGRHRKRRNYARISEVLELPNLIEIQTKSYDWFLKEGLLEMFRDISPIEDFTGNLSLEFVDYRLGEPKYDLEE**

cov pid  **81**  **. 1 . . . . : .** **160**

1 ATCC35984 100.0% 100.0%  **SKNRDATYAAPLRVKVRLIIKETGEVKEQEVFMGDFPLMTDTGTFVINGAERVIVSQLVRSPSVYFNEKIDKNGRENYDA**

2 ATCC12228 100.0% 100.0%  **SKNRDATYAAPLRVKVRLIIKETGEVKEQEVFMGDFPLMTDTGTFVINGAERVIVSQLVRSPSVYFNEKIDKNGRENYDA**

3 36AM 94.9% 100.0%  **SKNRDATYAAPLRVKVRLIIKETGEVKEQEVFMGDFPLMTDTGTFVINGAERVIVSQLVRSPSVYFNEKIDKNGRENYDA**

4 48AF 100.0% 100.0%  **SKNRDATYAAPLRVKVRLIIKETGEVKEQEVFMGDFPLMTDTGTFVINGAERVIVSQLVRSPSVYFNEKIDKNGRENYDA**

5 54AF 100.0% 99.9%  **SKNRDATYAAPLRVKVRLIIKETGEVKEQEVFMGDFPLMTDTGTFVINGAERVIVSQLVRSPSVYFNEKIDKNGRENYDA**

6 785_SEPI 100.0% 99.9%  **SKNRDATYAAPLRVKVRLIIKETGEVKEQEVFMGDFPLMTDTGTFVINGAERVIVSQLVRSPSVYFNEKIDKNGRENYDA**

7 VSE49 100.0% 100.0%  **SKNRDATYAAPLRVKVRLIIKETGEVKEQEVFMGDFPLMTDTGTFVINGAERVIVSQLVRSPSVYFNEKIDKNGRENYDA**

8 VSE57 100.0% 100.0%  **SKNRDATYAAPLRVKVRLIIKETGEVKEQEVFMGDFPLMTDTGTFVINGAERVIVSQLVRSPSVYFNEKIDKNGRENYDA**

9 SE45 100.0% 100.0%  **SKNRDATYAAPLRVKVRLIIKETGEVKEQEVFMGDFPLMTDTGTFVINGAERVIVSQLVRSPSVYFNEKIDKNGRENYDA**

10 SE40 100.0% 100.0%  **SKNRDATYAAPLRVKVRLIIKETGEVKEQEVFMGDFPLMTDTGTFVINGAERVIVSQLVRSPSVYFNEKIDKNGRENYDA**

11 32A 100.0% 99.2%  **SKNRDATYAAPLRVKVRLIIKETGEVKEQEVFMGDFPLMTDTGTFVINGAERVIVSQLVRSPSVYFNEKIDKNGRENYDA**

12 HD66 100.0% 100.0%  **SKNRDATYAAPLRVKVRLIIKETGEVKEQEVFMGDFPLMTDTGTFVINGAERVIVSQLVRSPSVYFNEKIDKNGRENYDA**

13 HD43 100.0% 99.8%  **SKNRDATYAAPLRVKVRLIIKETGEVKEQEVFMGDFPLMTDTGTFVINGAERVIVSQLVRSPSVYFNEKIDKNGRENYDA**

14 HD33 100.0% 99.9%  **SKNRDATYAAPLRVKVRLIIKETGEVKEQEVFMGDFPLMTDTGTFVINGAERVIVSQLVRSPSVYFNEKIDKNGRENYDA**

15 DE0525 100.0% 100.0%  **SKNRDATYAAPLRVKVRLIIKETGEVKEQEVFMGDFPLMTDTGTFVINGAERVIVSQLVRSPSVYFNEKIDKNGRENYDA**

16 4928STDY7071543 94.9% 100.0%  **SKNRDATYAAPLRVKVRLIIKETGEVKEQEVFMGDFPLMTDTGTFVINGAERVIVSQLVRSPSVYFNEKIDKNGRENYDA**

17 BAV2502 94.9% 99.9%  **SKNRDATYAAPLRVKVRLIIKETGEVKEQEVFMGDFPLMTDTGTFVINGAERVIVSQLVRSPSVYFNEKIDKNGRENYDA**

18 APC3784 100.0% 100.0%  **SKNRDATYAAPLRVKVRLIIKETGEVKEQEVFMGDFPLMTDTGTFVINGAERVIVSQLVRSPSVYFNEKIDKNGRENYDA**

19 SESURV_p1_0557 100.0% 100.0%  **SKNRDATYAAPLRVKVRLIIKETGEVKEQEVFMGDFPLMTDTGTFVINGAERVIVSQLVRSPSVYFNEKIDKNGRENYDA**

20 SESURV_p1_0612 100.0% 100.0%  **SKNRDATYAAPLRVKVRLIIKETGEVKEQEVFMGDFPLMTDTGTFVINGAERVIVSQLVRSPSVYFNEKIDKNGRENYDA**

21 SESURV_p4_1553 100.0% 100.0%  **SKNRDATYAAPLRVKVRLIIKETGEVKEQEVFMGDFPLMTDTGTFVINGAERVIVSQLVRSPSVYFNEKIDKNGRENYDA**

22 SESURV_p1_1200 100.0% 100.0%  **SKNRDATYAAPLRVKVRLIIKETGEVKEQEVFMGDFPLMTDTGTFVINGAERVIVSQLVRSPSVYFNEKIDKNGRENYDA**

23 SESURV_p3_0825 100.0% 100.0%  **SKNRDATYAAPLRVKVRLIIKETGEVKEQEVFMGDFPLMTDTGTFVINGAERVIVSQLVRSPSVYFNEKIDKNGRENYDA**

24 JH 100.0% 100.0%  **SKNRDATYAAPLRVKVRLIIKETGEVKEQEVFMGDFPLMTDTGTFVINGAERVIVSQLVRSPSVYFNEKIDKNGRENYDA**

25 Z0118SE0132 100.0% 99.9%  **SKNRDATYAAPLRVKVRLIIKETGEVKEQEVFMGDFPLMTDTGTFVINGAERVIVSQLVRSPSVYFNEKIDKNGRENYDA**

26 R5981 100.0% 100.0%  **SKNRDATYAAPLRVKVRLIIKETGEVKEQEVFMGDFPLMTDTGTFVINGAERVIVSQLVRSPSVYFNEKIDKNGRENYDA**

27 JH-S-3 100.0% 100.0%  **SKNRDATYAAPLRVKVRLIIKETGEVKEQEVFMGDFPLMTDTGTFVINGAERVIVSQLVRSPSVYFNEKIDKNGRENYDA**

28 JH-S-1 100.0% 100.0%  **SKNRDATYAAPLRVKVRLIIKETGEVKEQEVFMGDFPLMTDTGTFVINGAERVIVSQLVRSPSVYFNEKIDKNGRENYDA**

29 s2 100.0% 99.9%  **SKNRDATYAAPLRVKVRLIIKETGEVKEQEVFMGDFPLMTDTGTFVINGAERVIVSQLVRSPSVYFNEKIDKNGRENYDA**

30 S43 100.0% 100.0%  **SKNRDATYAAPLRVKVRLIIKETGEVKEQEVFMGDFPLMTDTGTFVINGAERVIVSQLVRSPSVYFNEKIDKNGRENYDA**

31 S17W 100.0% 100.0%  **SKNRDATYAAPLRVKVRLIIKETGEVKEQEVFMGDFPLMTDTGTFVINGAERVIVSQLVRSPSVYFNEKIDKNGRENYDA**

32 48 100.0% 99.9%  **SKNRDATYAAPLRVKVRLIIKETGEVKEQEVFMGDFPLMTDTGTFVINGAERVIVSQLVRSPSVYFNEKIDKNGRENYDA**

33 ABKVF 100.0% 99.9%  **SKNRDATYAAPLRVKVRLIIKETGEVKEQEVFMGDFPLMTDTGTFVINGAERVIVSQLVRSPSVYFNEKIDKNGRENYDA**

34 CICARIA 100.0% 100.0%  **SKNRDATYAAPLRVKVRLIIKETGEVKEQEVFMGDFPLMTDTGTFVINGAERVIVSQLVRSPSVYFNEKIDKNGRENYDA**

35 SKN25lux 100.0% 100.0%  **SKNRDATYAAPLRVKVRLIIKETGEVKEQEVFMGDFPLMTDTGTFVINGAERVIVSQLVRSPSVYFNEKIDKNGRENYDA**

36 HMSC074F11 100.0% 100.0%  **SKNRDATYAAPLRVKVRLIIKETGEVKEQEVFMGDFPLMTDTGTFVINGAERVIVSQLVRSPSVYFNEKIDKNGRENYDA**

37 HMSC068G11 100.0% 100.0%  **SKNRDATYAAPLRVKVRLIIKETGEVKEQEVFMGDFPLMTDTGTFVINGAERVIVSQLVRSPSVYFNEKIDKNGRENYDA**

38 NIHLM037 100.0% 99.9%  **SKNRDATYAAPLRVKVRLIIKETGEVKEQEVFMGDFPLMTDTGTFVINGAERVIVSQLVRSPSVYFNEKIDKNGRENYDA**

39 NIHLM015 100.0% 100.0%  **SKNRDATYAAPLRVKVRLIIKETGEVKEQEVFMGDFPLMTDTGTFVINGAERVIVSQLVRSPSVYFNEKIDKNGRENYDA**

consensus/100%  **SKNRDATYAAPLRVKVRLIIKETGEVKEQEVFMGDFPLMTDTGTFVINGAERVIVSQLVRSPSVYFNEKIDKNGRENYDA**

consensus/90%  **SKNRDATYAAPLRVKVRLIIKETGEVKEQEVFMGDFPLMTDTGTFVINGAERVIVSQLVRSPSVYFNEKIDKNGRENYDA**

consensus/80%  **SKNRDATYAAPLRVKVRLIIKETGEVKEQEVFMGDFPLMTDTGTFVINGAERVIVSQLVRSPSVYFNEKIDKNGRENYDA**

consensus/70%  **SKNRDATYAAPLRVKVRLIIKETGEVKEQEVFMGDFPLMTDTGTFVINGAERVIVSQLVRSPSVYFNEKIDKNGRENYDA**

cov pid  **161**  **. . . 2 . . . .** **240**

1 ATCC35984 100.0% 100.0%  **TIIPNRGAWLEYETDAKDVVYVRIDRTRKLPLTVLLRALGFSTDQEIVDLLGDSEYLRNTLEKDGTENTEQALLEIYERL**

2 ATCC12228 100.0% 100.0%  **TIIPNRGAWLEYETDAKDVVYVRIDRTRKLPLTVLLRALGFSTDQEIVDLLGDSEYLRNTLEKDGTENTEQALLEIYERL**

3 36AM 94.9% 100.0%  **TIIPNRGAWLEYETDAKDVVYVRIDRTRKLPLTVLLRALGFSTDQEIVDLLGDSEYLRNTLEKDGTENTEQALLEIYERL**

4 48AF 100.0% 100.0%  **TIIPNRGAWLEYETDAKDVVYVRIDRTRKLPLTVLLRALGFSTDQEIVDLLGDSEYLRNTLEKDGTENTEQALLEIYERL**

5 54AF 100.0% 99.9%  **TIIPNRGAWLEYETDAKDVVYVRIDRTRKLPLTVLLRALGFSTDQEIVDLLGDSEYLRNTLEKDGTENTEQALLEIYERL**

6 785_SEPI 100.0% 99.9%  **TIIPNRGAWLEYETDAKDVVYVRIDRTRKLPLTVLLRALGFSTDQEIVDLLGDSEYLRNTLEKDGTENTEQALLEIYERL**

7 VSE49 100.0% 100.0%  **TIIPNRGAWLEYETDAKDVVYVRIDRTRKLPLTVLLRALGFSTDQEIVDLLGDSEYLRNTLEKDGTENTEQALLEIYERL**

8 VSE57 100.0% 100.0%  **TIIPNRGAWLEYETDAKDVVYVRIDRTRKLPLTVLLRALGFSTDQEIVDLLGDSEYLRNTLEKDGTENTEQALLEIYERL**

9 SE45 100.0% 100.0%  **TIIPNRGAWLEYETDAKDVVYVRIDRTRKLPLTVLLRALGFSTDQEIVDLLGDSEYLRNTLEKDGTENTEQALLEIYERL**

10 SE40 100.0% 100.0%  **TIIPNRGAWLEYETDAKDVVYVRIDRTRKLPLTVLLRALGFSTDQEIVDLLGDSEYLRNTLEKDGTENTEQALLEIYERL**

11 32A 100.0% 99.2%  **TIIPNRGAWLEYETDAKDVVYVRIDRTRKLPLTVLLRALGFSTDQEIVDLLGDSEYLRNTLEKDGTENTEQALLEIYERL**

12 HD66 100.0% 100.0%  **TIIPNRGAWLEYETDAKDVVYVRIDRTRKLPLTVLLRALGFSTDQEIVDLLGDSEYLRNTLEKDGTENTEQALLEIYERL**

13 HD43 100.0% 99.8%  **TIIPNRGAWLEYETDAKDVVYVRIDRTRKLPLTVLLRALGFSTDQEIVDLLGDSEYLRNTLEKDGTENTEQALLEIYERL**

14 HD33 100.0% 99.9%  **TIIPNRGAWLEYETDAKDVVYVRIDRTRKLPLTVLLRALGFSTDQEIVDLLGDSEYLRNTLEKDGTENTEQALLEIYERL**

15 DE0525 100.0% 100.0%  **TIIPNRGAWLEYETDAKDVVYVRIDRTRKLPLTVLLRALGFSTDQEIVDLLGDSEYLRNTLEKDGTENTEQALLEIYERL**

16 4928STDY7071543 94.9% 100.0%  **TIIPNRGAWLEYETDAKDVVYVRIDRTRKLPLTVLLRALGFSTDQEIVDLLGDSEYLRNTLEKDGTENTEQALLEIYERL**

17 BAV2502 94.9% 99.9%  **TIIPNRGAWLEYETDAKDVVYVRIDRTRKLPLTVLLRALGFSTDQEIVDLLGDSEYLRNTLEKDGTENTEQALLEIYERL**

18 APC3784 100.0% 100.0%  **TIIPNRGAWLEYETDAKDVVYVRIDRTRKLPLTVLLRALGFSTDQEIVDLLGDSEYLRNTLEKDGTENTEQALLEIYERL**

19 SESURV_p1_0557 100.0% 100.0%  **TIIPNRGAWLEYETDAKDVVYVRIDRTRKLPLTVLLRALGFSTDQEIVDLLGDSEYLRNTLEKDGTENTEQALLEIYERL**

20 SESURV_p1_0612 100.0% 100.0%  **TIIPNRGAWLEYETDAKDVVYVRIDRTRKLPLTVLLRALGFSTDQEIVDLLGDSEYLRNTLEKDGTENTEQALLEIYERL**

21 SESURV_p4_1553 100.0% 100.0%  **TIIPNRGAWLEYETDAKDVVYVRIDRTRKLPLTVLLRALGFSTDQEIVDLLGDSEYLRNTLEKDGTENTEQALLEIYERL**

22 SESURV_p1_1200 100.0% 100.0%  **TIIPNRGAWLEYETDAKDVVYVRIDRTRKLPLTVLLRALGFSTDQEIVDLLGDSEYLRNTLEKDGTENTEQALLEIYERL**

23 SESURV_p3_0825 100.0% 100.0%  **TIIPNRGAWLEYETDAKDVVYVRIDRTRKLPLTVLLRALGFSTDQEIVDLLGDSEYLRNTLEKDGTENTEQALLEIYERL**

24 JH 100.0% 100.0%  **TIIPNRGAWLEYETDAKDVVYVRIDRTRKLPLTVLLRALGFSTDQEIVDLLGDSEYLRNTLEKDGTENTEQALLEIYERL**

25 Z0118SE0132 100.0% 99.9%  **TIIPNRGAWLEYETDAKDVVYVRIDRTRKLPLTVLLRALGFSTDQEIVDLLGDSEYLRNTLEKDGTENTEQALLEIYERL**

26 R5981 100.0% 100.0%  **TIIPNRGAWLEYETDAKDVVYVRIDRTRKLPLTVLLRALGFSTDQEIVDLLGDSEYLRNTLEKDGTENTEQALLEIYERL**

27 JH-S-3 100.0% 100.0%  **TIIPNRGAWLEYETDAKDVVYVRIDRTRKLPLTVLLRALGFSTDQEIVDLLGDSEYLRNTLEKDGTENTEQALLEIYERL**

28 JH-S-1 100.0% 100.0%  **TIIPNRGAWLEYETDAKDVVYVRIDRTRKLPLTVLLRALGFSTDQEIVDLLGDSEYLRNTLEKDGTENTEQALLEIYERL**

29 s2 100.0% 99.9%  **TIIPNRGAWLEYETDAKDVVYVRIDRTRKLPLTVLLRALGFSTDQEIVDLLGDSEYLRNTLEKDGTENTEQALLEIYERL**

30 S43 100.0% 100.0%  **TIIPNRGAWLEYETDAKDVVYVRIDRTRKLPLTVLLRALGFSTDQEIVDLLGDSEYLRNTLEKDGTENTEQALLEIYERL**

31 S17W 100.0% 100.0%  **TIIPNRGAWLEYETDAKDVVYVRIDRTRKLPLTVLLRALGFSTDQEIVDLLGDSEYLRNTLEKDGTENTEQALLEIYERL**

32 48 100.0% 99.9%  **TIIPNRGAWLEYETDAKDVVYVRIDRTRKLPLTVLLRALGFSTDQEIVDLLGDSEYLRNTLEKDGTENTEQALLEIYERL**

33 ABKVF 100.0% 99.9%  **TIIPNRGAWLEYETDAKDVVYVRIDRTRKLPLTVLLRALGFSTDQEIVDLLGDSEYLRNTLEKDGTENTEQALLEIYERL**

34 CICARIA 100.0% 100.0%  **TIIPNRGAWLEYETDAKDVVYVRIDRTRKLPLTVLLRALGFSTDQEIVDLLGDSEYLRNTLEKDGTENTEQALLEIYERL**

35 SKN25lux 100.0% 100.0%  **TIIPNRGAWLEYETDAKDVVYVRIDRTRKLPLTVLLRALGFSTDQEIVDLLGDSEYLRNTLEKDGTENTEQALLEIYERL**

36 HMSC074F11 100.0% 100.0%  **TIIPNRGAWLEYETDAKDVVYVRIDRTRKLPLTVLLRALGFSTDQEIVDLLGDSEYLRNTLEKDGTENTEQALLEIYERL**

37 HMSC068G11 100.0% 100.0%  **TIIPNRGAWLEYETDAKDVVYVRIDRTRKLPLTVLLRALGFSTDQEIVDLLGDSEYLRNTLEKDGTENTEQALLEIYERL**

38 NIHLM037 100.0% 99.9%  **TIIPNRGAWLEYETDAKDVVYVRIDRTRKLPLTVLLRALGFSTDQEIVDLLGDSEYLRNTLEKDGTENTEQALLEIYERL**

39 NIHLM015 100.0% 100.0%  **TIIPNRGAWLEYETDAKDVVYVRIDRTRKLPLTVLLRALGFSTDQEIVDLLGDSEYLRNTLEKDGTENTEQALLEIYERL**

consensus/100%  **TIIPNRGAWLEYETDAKDVVYVRIDRTRKLPLTVLLRALGFSTDQEIVDLLGDSEYLRNTLEKDGTENTEQALLEIYERL**

consensus/90%  **TIIPNRGAWLEYETDAKDVVYVRIDRTRKLPLTVLLRALGFSTDQEIVDLLGDSEYLRNTLEKDGTENTEQALLEIYERL**

consensus/80%  **TIIPNRGAWLEYETDAKDVVYVRIDRTRKLPLTVLLRALGFSTDQEIVDLLGDSEYLRNTLEKDGTENTEQALLEIYERL**

consensus/70%  **TIIPNRGAWLEYETDAKDVVYVRIDRTRKLPLTVLLRALGFSTDQEIVDLLGDSEYLRNTLEKDGTENTEQALLEIYERL**

cov pid  **241**  **: . . . . 3 . .** **320**

1 ATCC35984 100.0% 100.0%  **RPGEPPTVENAKSLLYSRFFDPKRYDLASVGRYKANKKLHLKHRLFNQKLAEPIVNSETGEIVVDEGTVLDRRKLDEIMD**

2 ATCC12228 100.0% 100.0%  **RPGEPPTVENAKSLLYSRFFDPKRYDLASVGRYKANKKLHLKHRLFNQKLAEPIVNSETGEIVVDEGTVLDRRKLDEIMD**

3 36AM 94.9% 100.0%  **RPGEPPTVENAKSLLYSRFFDPKRYDLASVGRYKANKKLHLKHRLFNQKLAEPIVNSETGEIVVDEGTVLDRRKLDEIMD**

4 48AF 100.0% 100.0%  **RPGEPPTVENAKSLLYSRFFDPKRYDLASVGRYKANKKLHLKHRLFNQKLAEPIVNSETGEIVVDEGTVLDRRKLDEIMD**

5 54AF 100.0% 99.9%  **RPGEPPTVENAKSLLYSRFFDPKRYDLASVGRYKANKKLHLKHRLFNQKLAEPIVNSETGEIVVDEGTVLDRRKLDEIMD**

6 785_SEPI 100.0% 99.9%  **RPGEPPTVENAKSLLYSRFFDPKRYDLASVGRYKANKKLHLKHRLFNQKLAEPIVNSETGEIVVDEGTVLDRRKLDEIMD**

7 VSE49 100.0% 100.0%  **RPGEPPTVENAKSLLYSRFFDPKRYDLASVGRYKANKKLHLKHRLFNQKLAEPIVNSETGEIVVDEGTVLDRRKLDEIMD**

8 VSE57 100.0% 100.0%  **RPGEPPTVENAKSLLYSRFFDPKRYDLASVGRYKANKKLHLKHRLFNQKLAEPIVNSETGEIVVDEGTVLDRRKLDEIMD**

9 SE45 100.0% 100.0%  **RPGEPPTVENAKSLLYSRFFDPKRYDLASVGRYKANKKLHLKHRLFNQKLAEPIVNSETGEIVVDEGTVLDRRKLDEIMD**

10 SE40 100.0% 100.0%  **RPGEPPTVENAKSLLYSRFFDPKRYDLASVGRYKANKKLHLKHRLFNQKLAEPIVNSETGEIVVDEGTVLDRRKLDEIMD**

11 32A 100.0% 99.2%  **RPGEPPTVENAKSLLYSRFFDPKRYDLASVGRYKANKKLHLKHRLFNQKLAEPIVNSETGEIVVDEGTVLDRRKLDEIMD**

12 HD66 100.0% 100.0%  **RPGEPPTVENAKSLLYSRFFDPKRYDLASVGRYKANKKLHLKHRLFNQKLAEPIVNSETGEIVVDEGTVLDRRKLDEIMD**

13 HD43 100.0% 99.8%  **RPGEPPTVENAKSLLYSRFFDPKRYDLASVGRYKANKKLHLKHRLFNQKLAEPIVNSETGEIVVDEGTVLDRRKLDEIMD**

14 HD33 100.0% 99.9%  **RPGEPPTVENAKSLLYSRFFDPKRYDLASVGRYKANKKLHLKHRLFNQKLAEPIVNSETGEIVVDEGTVLDRRKLDEIMD**

15 DE0525 100.0% 100.0%  **RPGEPPTVENAKSLLYSRFFDPKRYDLASVGRYKANKKLHLKHRLFNQKLAEPIVNSETGEIVVDEGTVLDRRKLDEIMD**

16 4928STDY7071543 94.9% 100.0%  **RPGEPPTVENAKSLLYSRFFDPKRYDLASVGRYKANKKLHLKHRLFNQKLAEPIVNSETGEIVVDEGTVLDRRKLDEIMD**

17 BAV2502 94.9% 99.9%  **RPGEPPTVENAKSLLYSRFFDPKRYDLASVGRYKANKKLHLKHRLFNQKLAEPIVNSETGEIVVDEGTVLDRRKLDEIMD**

18 APC3784 100.0% 100.0%  **RPGEPPTVENAKSLLYSRFFDPKRYDLASVGRYKANKKLHLKHRLFNQKLAEPIVNSETGEIVVDEGTVLDRRKLDEIMD**

19 SESURV_p1_0557 100.0% 100.0%  **RPGEPPTVENAKSLLYSRFFDPKRYDLASVGRYKANKKLHLKHRLFNQKLAEPIVNSETGEIVVDEGTVLDRRKLDEIMD**

20 SESURV_p1_0612 100.0% 100.0%  **RPGEPPTVENAKSLLYSRFFDPKRYDLASVGRYKANKKLHLKHRLFNQKLAEPIVNSETGEIVVDEGTVLDRRKLDEIMD**

21 SESURV_p4_1553 100.0% 100.0%  **RPGEPPTVENAKSLLYSRFFDPKRYDLASVGRYKANKKLHLKHRLFNQKLAEPIVNSETGEIVVDEGTVLDRRKLDEIMD**

22 SESURV_p1_1200 100.0% 100.0%  **RPGEPPTVENAKSLLYSRFFDPKRYDLASVGRYKANKKLHLKHRLFNQKLAEPIVNSETGEIVVDEGTVLDRRKLDEIMD**

23 SESURV_p3_0825 100.0% 100.0%  **RPGEPPTVENAKSLLYSRFFDPKRYDLASVGRYKANKKLHLKHRLFNQKLAEPIVNSETGEIVVDEGTVLDRRKLDEIMD**

24 JH 100.0% 100.0%  **RPGEPPTVENAKSLLYSRFFDPKRYDLASVGRYKANKKLHLKHRLFNQKLAEPIVNSETGEIVVDEGTVLDRRKLDEIMD**

25 Z0118SE0132 100.0% 99.9%  **RPGEPPTVENAKSLLYSRFFDPKRYDLASVGRYKANKKLHLKHRLFNQKLAEPIVNSETGEIVVDEGTVLDRRKLDEIMD**

26 R5981 100.0% 100.0%  **RPGEPPTVENAKSLLYSRFFDPKRYDLASVGRYKANKKLHLKHRLFNQKLAEPIVNSETGEIVVDEGTVLDRRKLDEIMD**

27 JH-S-3 100.0% 100.0%  **RPGEPPTVENAKSLLYSRFFDPKRYDLASVGRYKANKKLHLKHRLFNQKLAEPIVNSETGEIVVDEGTVLDRRKLDEIMD**

28 JH-S-1 100.0% 100.0%  **RPGEPPTVENAKSLLYSRFFDPKRYDLASVGRYKANKKLHLKHRLFNQKLAEPIVNSETGEIVVDEGTVLDRRKLDEIMD**

29 s2 100.0% 99.9%  **RPGEPPTVENAKSLLYSRFFDPKRYDLASVGRYKANKKLHLKHRLFNQKLAEPIVNSETGEIVVDEGTVLDRRKLDEIMD**

30 S43 100.0% 100.0%  **RPGEPPTVENAKSLLYSRFFDPKRYDLASVGRYKANKKLHLKHRLFNQKLAEPIVNSETGEIVVDEGTVLDRRKLDEIMD**

31 S17W 100.0% 100.0%  **RPGEPPTVENAKSLLYSRFFDPKRYDLASVGRYKANKKLHLKHRLFNQKLAEPIVNSETGEIVVDEGTVLDRRKLDEIMD**

32 48 100.0% 99.9%  **RPGEPPTVENAKSLLYSRFFDPKRYDLASVGRYKANKKLHLKHRLFNQKLAEPIVNSETGEIVVDEGTVLDRRKLDEIMD**

33 ABKVF 100.0% 99.9%  **RPGEPPTVENAKSLLYSRFFDPKRYDLASVGRYKANKKLHLKHRLFNQKLAEPIVNSETGEIVVDEGTVLDRRKLDEIMD**

34 CICARIA 100.0% 100.0%  **RPGEPPTVENAKSLLYSRFFDPKRYDLASVGRYKANKKLHLKHRLFNQKLAEPIVNSETGEIVVDEGTVLDRRKLDEIMD**

35 SKN25lux 100.0% 100.0%  **RPGEPPTVENAKSLLYSRFFDPKRYDLASVGRYKANKKLHLKHRLFNQKLAEPIVNSETGEIVVDEGTVLDRRKLDEIMD**

36 HMSC074F11 100.0% 100.0%  **RPGEPPTVENAKSLLYSRFFDPKRYDLASVGRYKANKKLHLKHRLFNQKLAEPIVNSETGEIVVDEGTVLDRRKLDEIMD**

37 HMSC068G11 100.0% 100.0%  **RPGEPPTVENAKSLLYSRFFDPKRYDLASVGRYKANKKLHLKHRLFNQKLAEPIVNSETGEIVVDEGTVLDRRKLDEIMD**

38 NIHLM037 100.0% 99.9%  **RPGEPPTVENAKSLLYSRFFDPKRYDLASVGRYKANKKLHLKHRLFNQKLAEPIVNSETGEIVVDEGTVLDRRKLDEIMD**

39 NIHLM015 100.0% 100.0%  **RPGEPPTVENAKSLLYSRFFDPKRYDLASVGRYKANKKLHLKHRLFNQKLAEPIVNSETGEIVVDEGTVLDRRKLDEIMD**

consensus/100%  **RPGEPPTVENAKSLLYSRFFDPKRYDLASVGRYKANKKLHLKHRLFNQKLAEPIVNSETGEIVVDEGTVLDRRKLDEIMD**

consensus/90%  **RPGEPPTVENAKSLLYSRFFDPKRYDLASVGRYKANKKLHLKHRLFNQKLAEPIVNSETGEIVVDEGTVLDRRKLDEIMD**

consensus/80%  **RPGEPPTVENAKSLLYSRFFDPKRYDLASVGRYKANKKLHLKHRLFNQKLAEPIVNSETGEIVVDEGTVLDRRKLDEIMD**

consensus/70%  **RPGEPPTVENAKSLLYSRFFDPKRYDLASVGRYKANKKLHLKHRLFNQKLAEPIVNSETGEIVVDEGTVLDRRKLDEIMD**

cov pid  **321**  **. . : . . . . 4** **400**

1 ATCC35984 100.0% 100.0%  **VLETNANSEVFELEGSVIDEPVEIQSIKVYVPNDEEGRTTTVIGNALPDSEVKCITPADIVASMSYFFNLLNGIGYTDDI**

2 ATCC12228 100.0% 100.0%  **VLETNANSEVFELEGSVIDEPVEIQSIKVYVPNDEEGRTTTVIGNALPDSEVKCITPADIVASMSYFFNLLNGIGYTDDI**

3 36AM 94.9% 100.0%  **VLETNANSEVFELEGSVIDEPVEIQSIKVYVPNDEEGRTTTVIGNALPDSEVKCITPADIVASMSYFFNLLNGIGYTDDI**

4 48AF 100.0% 100.0%  **VLETNANSEVFELEGSVIDEPVEIQSIKVYVPNDEEGRTTTVIGNALPDSEVKCITPADIVASMSYFFNLLNGIGYTDDI**

5 54AF 100.0% 99.9%  **VLETNANSEVFELEGSVIDEPVEIQSIKVYVPNDEEGRTTTVIGNALPDSEVKCITPADIVASMSYFFNLLNGIGYTDDI**

6 785_SEPI 100.0% 99.9%  **VLETNANSEVFELEGSVIDEPVEIQSIKVYVPNDEEGRTTTVIGNALPDSEVKCITPADIVASMSYFFNLLNGIGYTDDI**

7 VSE49 100.0% 100.0%  **VLETNANSEVFELEGSVIDEPVEIQSIKVYVPNDEEGRTTTVIGNALPDSEVKCITPADIVASMSYFFNLLNGIGYTDDI**

8 VSE57 100.0% 100.0%  **VLETNANSEVFELEGSVIDEPVEIQSIKVYVPNDEEGRTTTVIGNALPDSEVKCITPADIVASMSYFFNLLNGIGYTDDI**

9 SE45 100.0% 100.0%  **VLETNANSEVFELEGSVIDEPVEIQSIKVYVPNDEEGRTTTVIGNALPDSEVKCITPADIVASMSYFFNLLNGIGYTDDI**

10 SE40 100.0% 100.0%  **VLETNANSEVFELEGSVIDEPVEIQSIKVYVPNDEEGRTTTVIGNALPDSEVKCITPADIVASMSYFFNLLNGIGYTDDI**

11 32A 100.0% 99.2%  **VLETNANSEVFELEGSVIDEPVEIQSIKVYVPNDEEGRTTTVIGNALPDSEVKCITPADIVASMSYFFNLLNGIGYTDDI**

12 HD66 100.0% 100.0%  **VLETNANSEVFELEGSVIDEPVEIQSIKVYVPNDEEGRTTTVIGNALPDSEVKCITPADIVASMSYFFNLLNGIGYTDDI**

13 HD43 100.0% 99.8%  **VLETNANSEVFELEGSVIDEPVEIQSIKVYVPNDEEGRTTTVIGNALPDSEVKCITPADIVASMSYFFNLLNGIGYTDDI**

14 HD33 100.0% 99.9%  **VLETNANSEVFELEGSVIDEPVEIQSIKVYVPNDEEGRTTTVIGNALPDSEVKCITPADIVASMSYFFNLLNGIGYTDDI**

15 DE0525 100.0% 100.0%  **VLETNANSEVFELEGSVIDEPVEIQSIKVYVPNDEEGRTTTVIGNALPDSEVKCITPADIVASMSYFFNLLNGIGYTDDI**

16 4928STDY7071543 94.9% 100.0%  **VLETNANSEVFELEGSVIDEPVEIQSIKVYVPNDEEGRTTTVIGNALPDSEVKCITPADIVASMSYFFNLLNGIGYTDDI**

17 BAV2502 94.9% 99.9%  **VLETNANSEVFELEGSVIDEPVEIQSIKVYVPNDEEGRTTTVIGNALPDSEVKCITPADIVASMSYFFNLLNGIGYTDDI**

18 APC3784 100.0% 100.0%  **VLETNANSEVFELEGSVIDEPVEIQSIKVYVPNDEEGRTTTVIGNALPDSEVKCITPADIVASMSYFFNLLNGIGYTDDI**

19 SESURV_p1_0557 100.0% 100.0%  **VLETNANSEVFELEGSVIDEPVEIQSIKVYVPNDEEGRTTTVIGNALPDSEVKCITPADIVASMSYFFNLLNGIGYTDDI**

20 SESURV_p1_0612 100.0% 100.0%  **VLETNANSEVFELEGSVIDEPVEIQSIKVYVPNDEEGRTTTVIGNALPDSEVKCITPADIVASMSYFFNLLNGIGYTDDI**

21 SESURV_p4_1553 100.0% 100.0%  **VLETNANSEVFELEGSVIDEPVEIQSIKVYVPNDEEGRTTTVIGNALPDSEVKCITPADIVASMSYFFNLLNGIGYTDDI**

22 SESURV_p1_1200 100.0% 100.0%  **VLETNANSEVFELEGSVIDEPVEIQSIKVYVPNDEEGRTTTVIGNALPDSEVKCITPADIVASMSYFFNLLNGIGYTDDI**

23 SESURV_p3_0825 100.0% 100.0%  **VLETNANSEVFELEGSVIDEPVEIQSIKVYVPNDEEGRTTTVIGNALPDSEVKCITPADIVASMSYFFNLLNGIGYTDDI**

24 JH 100.0% 100.0%  **VLETNANSEVFELEGSVIDEPVEIQSIKVYVPNDEEGRTTTVIGNALPDSEVKCITPADIVASMSYFFNLLNGIGYTDDI**

25 Z0118SE0132 100.0% 99.9%  **VLETNANSEVFELEGSVIDEPVEIQSIKVYVPNDEEGCTTTVIGNALPDSEVKCITPADIVASMSYFFNLLNGIGYTDDI**

26 R5981 100.0% 100.0%  **VLETNANSEVFELEGSVIDEPVEIQSIKVYVPNDEEGRTTTVIGNALPDSEVKCITPADIVASMSYFFNLLNGIGYTDDI**

27 JH-S-3 100.0% 100.0%  **VLETNANSEVFELEGSVIDEPVEIQSIKVYVPNDEEGRTTTVIGNALPDSEVKCITPADIVASMSYFFNLLNGIGYTDDI**

28 JH-S-1 100.0% 100.0%  **VLETNANSEVFELEGSVIDEPVEIQSIKVYVPNDEEGRTTTVIGNALPDSEVKCITPADIVASMSYFFNLLNGIGYTDDI**

29 s2 100.0% 99.9%  **VLETNANSEVFELEGSVIDEPVEIQSIKVYVPNDEEGRTTTVIGNALPDSEVKCITPADIVASMSYFFNLLNGIGYTDDI**

30 S43 100.0% 100.0%  **VLETNANSEVFELEGSVIDEPVEIQSIKVYVPNDEEGRTTTVIGNALPDSEVKCITPADIVASMSYFFNLLNGIGYTDDI**

31 S17W 100.0% 100.0%  **VLETNANSEVFELEGSVIDEPVEIQSIKVYVPNDEEGRTTTVIGNALPDSEVKCITPADIVASMSYFFNLLNGIGYTDDI**

32 48 100.0% 99.9%  **VLETNANSEVFELEGSVIDEPVEIQSIKVYVPNDEEGRTTTVIGNALPDSEVKCITPADIVASMSYFFNLLNGIGYTDDI**

33 ABKVF 100.0% 99.9%  **VLETNANSEVFELEGRVIDEPVEIQSIKVYVPNDEEGRTTTVIGNALPDSEVKCITPADIVASMSYFFNLLNGIGYTDDI**

34 CICARIA 100.0% 100.0%  **VLETNANSEVFELEGSVIDEPVEIQSIKVYVPNDEEGRTTTVIGNALPDSEVKCITPADIVASMSYFFNLLNGIGYTDDI**

35 SKN25lux 100.0% 100.0%  **VLETNANSEVFELEGSVIDEPVEIQSIKVYVPNDEEGRTTTVIGNALPDSEVKCITPADIVASMSYFFNLLNGIGYTDDI**

36 HMSC074F11 100.0% 100.0%  **VLETNANSEVFELEGSVIDEPVEIQSIKVYVPNDEEGRTTTVIGNALPDSEVKCITPADIVASMSYFFNLLNGIGYTDDI**

37 HMSC068G11 100.0% 100.0%  **VLETNANSEVFELEGSVIDEPVEIQSIKVYVPNDEEGRTTTVIGNALPDSEVKCITPADIVASMSYFFNLLNGIGYTDDI**

38 NIHLM037 100.0% 99.9%  **VLETNANSEVFELEGSVIDEPVEIQSIKVYVPNDEEGRTTTVIGNALPDSEVKCITPADIVASMSYFFNLLNGIGYTDDI**

39 NIHLM015 100.0% 100.0%  **VLETNANSEVFELEGSVIDEPVEIQSIKVYVPNDEEGRTTTVIGNALPDSEVKCITPADIVASMSYFFNLLNGIGYTDDI**

consensus/100%  **VLETNANSEVFELEGpVIDEPVEIQSIKVYVPNDEEGpTTTVIGNALPDSEVKCITPADIVASMSYFFNLLNGIGYTDDI**

consensus/90%  **VLETNANSEVFELEGSVIDEPVEIQSIKVYVPNDEEGRTTTVIGNALPDSEVKCITPADIVASMSYFFNLLNGIGYTDDI**

consensus/80%  **VLETNANSEVFELEGSVIDEPVEIQSIKVYVPNDEEGRTTTVIGNALPDSEVKCITPADIVASMSYFFNLLNGIGYTDDI**

consensus/70%  **VLETNANSEVFELEGSVIDEPVEIQSIKVYVPNDEEGRTTTVIGNALPDSEVKCITPADIVASMSYFFNLLNGIGYTDDI**

cov pid  **401**  **. . . . : . . .** **480**

1 ATCC35984 100.0% 100.0%  **DHLGNRRLRSVGELLQNQFRIGLSRMERVVRERMSIQDTDSITPQQLINIRPVIASIKEFFGSSQLSQFMDQANPLAELT**

2 ATCC12228 100.0% 100.0%  **DHLGNRRLRSVGELLQNQFRIGLSRMERVVRERMSIQDTDSITPQQLINIRPVIASIKEFFGSSQLSQFMDQANPLAELT**

3 36AM 94.9% 100.0%  **DHLGNRRLRSVGELLQNQFRIGLSRMERVVRERMSIQDTDSITPQQLINIRPVIASIKEFFGSSQLSQFMDQANPLAELT**

4 48AF 100.0% 100.0%  **DHLGNRRLRSVGELLQNQFRIGLSRMERVVRERMSIQDTDSITPQQLINIRPVIASIKEFFGSSQLSQFMDQANPLAELT**

5 54AF 100.0% 99.9%  **DHLGNRRLRSVGELLQNQFRIGLSRMERVVRERMSIQDTDSITPQQLINIRPVIASIKEFFGSSQLSQFMDQANPLAELT**

6 785_SEPI 100.0% 99.9%  **DHLGNRRLRSVGELLQNQFRIGLSRMERVVRERMSIQDTDSITPQQLINIRPVIASIKEFFGSSQLSQFMDQANPLAELT**

7 VSE49 100.0% 100.0%  **DHLGNRRLRSVGELLQNQFRIGLSRMERVVRERMSIQDTDSITPQQLINIRPVIASIKEFFGSSQLSQFMDQANPLAELT**

8 VSE57 100.0% 100.0%  **DHLGNRRLRSVGELLQNQFRIGLSRMERVVRERMSIQDTDSITPQQLINIRPVIASIKEFFGSSQLSQFMDQANPLAELT**

9 SE45 100.0% 100.0%  **DHLGNRRLRSVGELLQNQFRIGLSRMERVVRERMSIQDTDSITPQQLINIRPVIASIKEFFGSSQLSQFMDQANPLAELT**

10 SE40 100.0% 100.0%  **DHLGNRRLRSVGELLQNQFRIGLSRMERVVRERMSIQDTDSITPQQLINIRPVIASIKEFFGSSQLSQFMDQANPLAELT**

11 32A 100.0% 99.2%  **DHLGNRRLRSVGELLQNQFRIGLSRMERVVRERMSIQDTDSITPQQLINIRPVIASIKEFFGSSQLSQFMDQANPLAELT**

12 HD66 100.0% 100.0%  **DHLGNRRLRSVGELLQNQFRIGLSRMERVVRERMSIQDTDSITPQQLINIRPVIASIKEFFGSSQLSQFMDQANPLAELT**

13 HD43 100.0% 99.8%  **DHLGNRRLRSVGELLQNQFRIGLSRMERVVRERMSIQDTDSITPQQLINIRPVIASIKEFFGSSQLSQFMEQANPLAELT**

14 HD33 100.0% 99.9%  **DHLGNRRLRSVGELLQNQFRIGLSRMERVVRERMSIQDTDSITPQQLINIRPVIASIKEFFGSSQLSQFMDQANPLAELT**

15 DE0525 100.0% 100.0%  **DHLGNRRLRSVGELLQNQFRIGLSRMERVVRERMSIQDTDSITPQQLINIRPVIASIKEFFGSSQLSQFMDQANPLAELT**

16 4928STDY7071543 94.9% 100.0%  **DHLGNRRLRSVGELLQNQFRIGLSRMERVVRERMSIQDTDSITPQQLINIRPVIASIKEFFGSSQLSQFMDQANPLAELT**

17 BAV2502 94.9% 99.9%  **DHLGNRRLRSVGELLQNQFRIGLSRMERVVRERMSIQDTDSITPQQLINIRPVIASIKEFFGSSQLSQFMDQANPLAELT**

18 APC3784 100.0% 100.0%  **DHLGNRRLRSVGELLQNQFRIGLSRMERVVRERMSIQDTDSITPQQLINIRPVIASIKEFFGSSQLSQFMDQANPLAELT**

19 SESURV_p1_0557 100.0% 100.0%  **DHLGNRRLRSVGELLQNQFRIGLSRMERVVRERMSIQDTDSITPQQLINIRPVIASIKEFFGSSQLSQFMDQANPLAELT**

20 SESURV_p1_0612 100.0% 100.0%  **DHLGNRRLRSVGELLQNQFRIGLSRMERVVRERMSIQDTDSITPQQLINIRPVIASIKEFFGSSQLSQFMDQANPLAELT**

21 SESURV_p4_1553 100.0% 100.0%  **DHLGNRRLRSVGELLQNQFRIGLSRMERVVRERMSIQDTDSITPQQLINIRPVIASIKEFFGSSQLSQFMDQANPLAELT**

22 SESURV_p1_1200 100.0% 100.0%  **DHLGNRRLRSVGELLQNQFRIGLSRMERVVRERMSIQDTDSITPQQLINIRPVIASIKEFFGSSQLSQFMDQANPLAELT**

23 SESURV_p3_0825 100.0% 100.0%  **DHLGNRRLRSVGELLQNQFRIGLSRMERVVRERMSIQDTDSITPQQLINIRPVIASIKEFFGSSQLSQFMDQANPLAELT**

24 JH 100.0% 100.0%  **DHLGNRRLRSVGELLQNQFRIGLSRMERVVRERMSIQDTDSITPQQLINIRPVIASIKEFFGSSQLSQFMDQANPLAELT**

25 Z0118SE0132 100.0% 99.9%  **DHLGNRRLRSVGELLQNQFRIGLSRMERVVRERMSIQDTDSITPQQLINIRPVIASIKEFFGSSQLSQFMDQANPLAELT**

26 R5981 100.0% 100.0%  **DHLGNRRLRSVGELLQNQFRIGLSRMERVVRERMSIQDTDSITPQQLINIRPVIASIKEFFGSSQLSQFMDQANPLAELT**

27 JH-S-3 100.0% 100.0%  **DHLGNRRLRSVGELLQNQFRIGLSRMERVVRERMSIQDTDSITPQQLINIRPVIASIKEFFGSSQLSQFMDQANPLAELT**

28 JH-S-1 100.0% 100.0%  **DHLGNRRLRSVGELLQNQFRIGLSRMERVVRERMSIQDTDSITPQQLINIRPVIASIKEFFGSSQLSQFMDQANPLAELT**

29 s2 100.0% 99.9%  **DHLGNRRLRSVGELLQNQFRIGLSRMERVVRERMSIQDTDSITPQQLINIRPVIASIKEFFGSSQLSQFMDQANPLAELT**

30 S43 100.0% 100.0%  **DHLGNRRLRSVGELLQNQFRIGLSRMERVVRERMSIQDTDSITPQQLINIRPVIASIKEFFGSSQLSQFMDQANPLAELT**

31 S17W 100.0% 100.0%  **DHLGNRRLRSVGELLQNQFRIGLSRMERVVRERMSIQDTDSITPQQLINIRPVIASIKEFFGSSQLSQFMDQANPLAELT**

32 48 100.0% 99.9%  **DHLGNRRLRSVGELLQNQFRIGLSRMERVVRERMSIQDTDSITPQQLINIRPVIASIKEFFGSSQLSQFMDQANPLAELT**

33 ABKVF 100.0% 99.9%  **DHLGNRRLRSVGELLQNQFRIGLSRMERVVRERMSIQDTDSITPQQLINIRPVIASIKEFFGSSQLSQFMDQANPLAELT**

34 CICARIA 100.0% 100.0%  **DHLGNRRLRSVGELLQNQFRIGLSRMERVVRERMSIQDTDSITPQQLINIRPVIASIKEFFGSSQLSQFMDQANPLAELT**

35 SKN25lux 100.0% 100.0%  **DHLGNRRLRSVGELLQNQFRIGLSRMERVVRERMSIQDTDSITPQQLINIRPVIASIKEFFGSSQLSQFMDQANPLAELT**

36 HMSC074F11 100.0% 100.0%  **DHLGNRRLRSVGELLQNQFRIGLSRMERVVRERMSIQDTDSITPQQLINIRPVIASIKEFFGSSQLSQFMDQANPLAELT**

37 HMSC068G11 100.0% 100.0%  **DHLGNRRLRSVGELLQNQFRIGLSRMERVVRERMSIQDTDSITPQQLINIRPVIASIKEFFGSSQLSQFMDQANPLAELT**

38 NIHLM037 100.0% 99.9%  **DHLGNRRLRSVGELLQNQFRIGLSRMERVVRERMSIQDTDSITPQQLINIRPVIASIKEFFGSSQLSQFMDQANPLAELT**

39 NIHLM015 100.0% 100.0%  **DHLGNRRLRSVGELLQNQFRIGLSRMERVVRERMSIQDTDSITPQQLINIRPVIASIKEFFGSSQLSQFMDQANPLAELT**

consensus/100%  **DHLGNRRLRSVGELLQNQFRIGLSRMERVVRERMSIQDTDSITPQQLINIRPVIASIKEFFGSSQLSQFM-QANPLAELT**

consensus/90%  **DHLGNRRLRSVGELLQNQFRIGLSRMERVVRERMSIQDTDSITPQQLINIRPVIASIKEFFGSSQLSQFMDQANPLAELT**

consensus/80%  **DHLGNRRLRSVGELLQNQFRIGLSRMERVVRERMSIQDTDSITPQQLINIRPVIASIKEFFGSSQLSQFMDQANPLAELT**

consensus/70%  **DHLGNRRLRSVGELLQNQFRIGLSRMERVVRERMSIQDTDSITPQQLINIRPVIASIKEFFGSSQLSQFMDQANPLAELT**

cov pid  **481**  **. 5 . . . . : .** **560**

1 ATCC35984 100.0% 100.0%  **HKRRLSALGPGGLTRERAQMEVRDVHYSHYGRMCPIETPEGPNIGLINSLSSYARVNEFGFIETPYRKVDLDTNSITDQI**

2 ATCC12228 100.0% 100.0%  **HKRRLSALGPGGLTRERAQMEVRDVHYSHYGRMCPIETPEGPNIGLINSLSSYARVNEFGFIETPYRKVDLDTNSITDQI**

3 36AM 94.9% 100.0%  **HKRRLSALGPGGLTRERAQMEVRDVHYSHYGRMCPIETPEGPNIGLINSLSSYARVNEFGFIETPYRKVDLDTNSITDQI**

4 48AF 100.0% 100.0%  **HKRRLSALGPGGLTRERAQMEVRDVHYSHYGRMCPIETPEGPNIGLINSLSSYARVNEFGFIETPYRKVDLDTNSITDQI**

5 54AF 100.0% 99.9%  **HKRRLSALGPGGLTRERAQMEVRDVHYSHYGRMCPIETPEGPNIGLINSLSSYARVNEFGFIETPYRKVDLDTNSITDQI**

6 785_SEPI 100.0% 99.9%  **HKRRLSALGPGGLTRERAQMEVRDVHYSHYGRMCPIETPEGPNIGLINSLSSYARVNEFGFIETPYRKVDLDTNSITDQI**

7 VSE49 100.0% 100.0%  **HKRRLSALGPGGLTRERAQMEVRDVHYSHYGRMCPIETPEGPNIGLINSLSSYARVNEFGFIETPYRKVDLDTNSITDQI**

8 VSE57 100.0% 100.0%  **HKRRLSALGPGGLTRERAQMEVRDVHYSHYGRMCPIETPEGPNIGLINSLSSYARVNEFGFIETPYRKVDLDTNSITDQI**

9 SE45 100.0% 100.0%  **HKRRLSALGPGGLTRERAQMEVRDVHYSHYGRMCPIETPEGPNIGLINSLSSYARVNEFGFIETPYRKVDLDTNSITDQI**

10 SE40 100.0% 100.0%  **HKRRLSALGPGGLTRERAQMEVRDVHYSHYGRMCPIETPEGPNIGLINSLSSYARVNEFGFIETPYRKVDLDTNSITDQI**

11 32A 100.0% 99.2%  **HKRRLSALGPGGLTRERAQMEVRDVHYSHYGRMCPIETPEGPNIGLINSLSSYARVNEFGFIETPYRKVDLDTNSITDQI**

12 HD66 100.0% 100.0%  **HKRRLSALGPGGLTRERAQMEVRDVHYSHYGRMCPIETPEGPNIGLINSLSSYARVNEFGFIETPYRKVDLDTNSITDQI**

13 HD43 100.0% 99.8%  **HKRRLSALGPGGLTRERAQMEVRDVHYSHYGRMCPIETPEGPNIGLMNSLSSYARVNEFGFIETPYRKVDLDTNSITDQI**

14 HD33 100.0% 99.9%  **HKRRLSALGPGGLTRERAQMEVRDVHYSHYGRMCPIETPEGPNIGLINSLSSYARVNEFGFIETPYRKVDLDTNSITDQI**

15 DE0525 100.0% 100.0%  **HKRRLSALGPGGLTRERAQMEVRDVHYSHYGRMCPIETPEGPNIGLINSLSSYARVNEFGFIETPYRKVDLDTNSITDQI**

16 4928STDY7071543 94.9% 100.0%  **HKRRLSALGPGGLTRERAQMEVRDVHYSHYGRMCPIETPEGPNIGLINSLSSYARVNEFGFIETPYRKVDLDTNSITDQI**

17 BAV2502 94.9% 99.9%  **HKRRLSALGPGGLTRERAQMEVRDVHYSHYGRMCPIETPEGPNIGLINSLSSYARVNEFGFIETPYRKVDLDTNSITDQI**

18 APC3784 100.0% 100.0%  **HKRRLSALGPGGLTRERAQMEVRDVHYSHYGRMCPIETPEGPNIGLINSLSSYARVNEFGFIETPYRKVDLDTNSITDQI**

19 SESURV_p1_0557 100.0% 100.0%  **HKRRLSALGPGGLTRERAQMEVRDVHYSHYGRMCPIETPEGPNIGLINSLSSYARVNEFGFIETPYRKVDLDTNSITDQI**

20 SESURV_p1_0612 100.0% 100.0%  **HKRRLSALGPGGLTRERAQMEVRDVHYSHYGRMCPIETPEGPNIGLINSLSSYARVNEFGFIETPYRKVDLDTNSITDQI**

21 SESURV_p4_1553 100.0% 100.0%  **HKRRLSALGPGGLTRERAQMEVRDVHYSHYGRMCPIETPEGPNIGLINSLSSYARVNEFGFIETPYRKVDLDTNSITDQI**

22 SESURV_p1_1200 100.0% 100.0%  **HKRRLSALGPGGLTRERAQMEVRDVHYSHYGRMCPIETPEGPNIGLINSLSSYARVNEFGFIETPYRKVDLDTNSITDQI**

23 SESURV_p3_0825 100.0% 100.0%  **HKRRLSALGPGGLTRERAQMEVRDVHYSHYGRMCPIETPEGPNIGLINSLSSYARVNEFGFIETPYRKVDLDTNSITDQI**

24 JH 100.0% 100.0%  **HKRRLSALGPGGLTRERAQMEVRDVHYSHYGRMCPIETPEGPNIGLINSLSSYARVNEFGFIETPYRKVDLDTNSITDQI**

25 Z0118SE0132 100.0% 99.9%  **HKRRLSALGPGGLTRERAQMEVRDVHYSHYGRMCPIETPEGPNIGLINSLSSYARVNEFGFIETPYRKVDLDTNSITDQI**

26 R5981 100.0% 100.0%  **HKRRLSALGPGGLTRERAQMEVRDVHYSHYGRMCPIETPEGPNIGLINSLSSYARVNEFGFIETPYRKVDLDTNSITDQI**

27 JH-S-3 100.0% 100.0%  **HKRRLSALGPGGLTRERAQMEVRDVHYSHYGRMCPIETPEGPNIGLINSLSSYARVNEFGFIETPYRKVDLDTNSITDQI**

28 JH-S-1 100.0% 100.0%  **HKRRLSALGPGGLTRERAQMEVRDVHYSHYGRMCPIETPEGPNIGLINSLSSYARVNEFGFIETPYRKVDLDTNSITDQI**

29 s2 100.0% 99.9%  **HKRRLSALGPGGLTRERAQMEVRDVHYSHYGRMCPIETPEGPNIGLINSLSSYARVNEFGFIETPYRKVDLDTNSITDQI**

30 S43 100.0% 100.0%  **HKRRLSALGPGGLTRERAQMEVRDVHYSHYGRMCPIETPEGPNIGLINSLSSYARVNEFGFIETPYRKVDLDTNSITDQI**

31 S17W 100.0% 100.0%  **HKRRLSALGPGGLTRERAQMEVRDVHYSHYGRMCPIETPEGPNIGLINSLSSYARVNEFGFIETPYRKVDLDTNSITDQI**

32 48 100.0% 99.9%  **HKRRLSALGPGGLTRERAQMEVRDVHYSHYGRMCPIETPEGPNIGLINSLSSYARVNEFGFIETPYRKVDLDTNSITDQI**

33 ABKVF 100.0% 99.9%  **HKRRLSALGPGGLTRERAQMEVRDVHYSHYGRMCPIETPEGPNIGLINSLSSYARVNEFGFIETPYRKVDLDTNSITDQI**

34 CICARIA 100.0% 100.0%  **HKRRLSALGPGGLTRERAQMEVRDVHYSHYGRMCPIETPEGPNIGLINSLSSYARVNEFGFIETPYRKVDLDTNSITDQI**

35 SKN25lux 100.0% 100.0%  **HKRRLSALGPGGLTRERAQMEVRDVHYSHYGRMCPIETPEGPNIGLINSLSSYARVNEFGFIETPYRKVDLDTNSITDQI**

36 HMSC074F11 100.0% 100.0%  **HKRRLSALGPGGLTRERAQMEVRDVHYSHYGRMCPIETPEGPNIGLINSLSSYARVNEFGFIETPYRKVDLDTNSITDQI**

37 HMSC068G11 100.0% 100.0%  **HKRRLSALGPGGLTRERAQMEVRDVHYSHYGRMCPIETPEGPNIGLINSLSSYARVNEFGFIETPYRKVDLDTNSITDQI**

38 NIHLM037 100.0% 99.9%  **HKRRLSALGPGGLTRERAQMEVRDVHYSHYGRMCPIETPEGPNIGLINSLSSYARVNEFGFIETPYRKVDLDTNSITEQI**

39 NIHLM015 100.0% 100.0%  **HKRRLSALGPGGLTRERAQMEVRDVHYSHYGRMCPIETPEGPNIGLINSLSSYARVNEFGFIETPYRKVDLDTNSITDQI**

consensus/100%  **HKRRLSALGPGGLTRERAQMEVRDVHYSHYGRMCPIETPEGPNIGLhNSLSSYARVNEFGFIETPYRKVDLDTNSIT-QI**

consensus/90%  **HKRRLSALGPGGLTRERAQMEVRDVHYSHYGRMCPIETPEGPNIGLINSLSSYARVNEFGFIETPYRKVDLDTNSITDQI**

consensus/80%  **HKRRLSALGPGGLTRERAQMEVRDVHYSHYGRMCPIETPEGPNIGLINSLSSYARVNEFGFIETPYRKVDLDTNSITDQI**

consensus/70%  **HKRRLSALGPGGLTRERAQMEVRDVHYSHYGRMCPIETPEGPNIGLINSLSSYARVNEFGFIETPYRKVDLDTNSITDQI**

cov pid  **561**  **. . . 6 . . . .** **640**

1 ATCC35984 100.0% 100.0%  **DYLTADEEDSYVVAQANSRLDENGRFLDDEVVCRFRGNNTVMAKEKMDYMDVSPKQVVSAATACIPFLENDDSNRALMGA**

2 ATCC12228 100.0% 100.0%  **DYLTADEEDSYVVAQANSRLDENGRFLDDEVVCRFRGNNTVMAKEKMDYMDVSPKQVVSAATACIPFLENDDSNRALMGA**

3 36AM 94.9% 100.0%  **DYLTADEEDSYVVAQANSRLDENGRFLDDEVVCRFRGNNTVMAKEKMDYMDVSPKQVVSAATACIPFLENDDSNRALMGA**

4 48AF 100.0% 100.0%  **DYLTADEEDSYVVAQANSRLDENGRFLDDEVVCRFRGNNTVMAKEKMDYMDVSPKQVVSAATACIPFLENDDSNRALMGA**

5 54AF 100.0% 99.9%  **DYLTADEEDSYVVAQANSRLDENGRFLDDEVVCRFRGNNTVMAKEKMDYMDVSPKQVVSAATACIPFLENDDSNRALMGA**

6 785_SEPI 100.0% 99.9%  **DYLTADEEDSYVVAQANSRLDENGRFLDDEVVCRFRGNNTVMAKEKMDYMDVSPKQVVSAATACIPFLENDDSNRALMGA**

7 VSE49 100.0% 100.0%  **DYLTADEEDSYVVAQANSRLDENGRFLDDEVVCRFRGNNTVMAKEKMDYMDVSPKQVVSAATACIPFLENDDSNRALMGA**

8 VSE57 100.0% 100.0%  **DYLTADEEDSYVVAQANSRLDENGRFLDDEVVCRFRGNNTVMAKEKMDYMDVSPKQVVSAATACIPFLENDDSNRALMGA**

9 SE45 100.0% 100.0%  **DYLTADEEDSYVVAQANSRLDENGRFLDDEVVCRFRGNNTVMAKEKMDYMDVSPKQVVSAATACIPFLENDDSNRALMGA**

10 SE40 100.0% 100.0%  **DYLTADEEDSYVVAQANSRLDENGRFLDDEVVCRFRGNNTVMAKEKMDYMDVSPKQVVSAATACIPFLENDDSNRALMGA**

11 32A 100.0% 99.2%  **DYLTADEEDSYVVAQANSRLDENGRFLDDEVVCRFRGNNTVMAKEKMDYMDVSPKQVVSAATACIPFLENDDSNRALMGA**

12 HD66 100.0% 100.0%  **DYLTADEEDSYVVAQANSRLDENGRFLDDEVVCRFRGNNTVMAKEKMDYMDVSPKQVVSAATACIPFLENDDSNRALMGA**

13 HD43 100.0% 99.8%  **DYLTADEEDSYVVAQANSRLDENGRFLDDEVVCRFRGNNTVMAKEKMDYMDVSPKQVVSAATACIPFLENDDSNRALMGA**

14 HD33 100.0% 99.9%  **DYLTADEEDSYVVAQANSRLDENGRFLDDEVVCRFRGNNTVMAKEKMDYMDVSPKQVVSAATACIPFLENDDSNRALMGA**

15 DE0525 100.0% 100.0%  **DYLTADEEDSYVVAQANSRLDENGRFLDDEVVCRFRGNNTVMAKEKMDYMDVSPKQVVSAATACIPFLENDDSNRALMGA**

16 4928STDY7071543 94.9% 100.0%  **DYLTADEEDSYVVAQANSRLDENGRFLDDEVVCRFRGNNTVMAKEKMDYMDVSPKQVVSAATACIPFLENDDSNRALMGA**

17 BAV2502 94.9% 99.9%  **DYLTADEEDSYVVAQANSRLDENGRFLDDEVVCRFRGNNTVMAKEKMDYMDVSPKQVVSAATACIPFLENDDSNRALMGA**

18 APC3784 100.0% 100.0%  **DYLTADEEDSYVVAQANSRLDENGRFLDDEVVCRFRGNNTVMAKEKMDYMDVSPKQVVSAATACIPFLENDDSNRALMGA**

19 SESURV_p1_0557 100.0% 100.0%  **DYLTADEEDSYVVAQANSRLDENGRFLDDEVVCRFRGNNTVMAKEKMDYMDVSPKQVVSAATACIPFLENDDSNRALMGA**

20 SESURV_p1_0612 100.0% 100.0%  **DYLTADEEDSYVVAQANSRLDENGRFLDDEVVCRFRGNNTVMAKEKMDYMDVSPKQVVSAATACIPFLENDDSNRALMGA**

21 SESURV_p4_1553 100.0% 100.0%  **DYLTADEEDSYVVAQANSRLDENGRFLDDEVVCRFRGNNTVMAKEKMDYMDVSPKQVVSAATACIPFLENDDSNRALMGA**

22 SESURV_p1_1200 100.0% 100.0%  **DYLTADEEDSYVVAQANSRLDENGRFLDDEVVCRFRGNNTVMAKEKMDYMDVSPKQVVSAATACIPFLENDDSNRALMGA**

23 SESURV_p3_0825 100.0% 100.0%  **DYLTADEEDSYVVAQANSRLDENGRFLDDEVVCRFRGNNTVMAKEKMDYMDVSPKQVVSAATACIPFLENDDSNRALMGA**

24 JH 100.0% 100.0%  **DYLTADEEDSYVVAQANSRLDENGRFLDDEVVCRFRGNNTVMAKEKMDYMDVSPKQVVSAATACIPFLENDDSNRALMGA**

25 Z0118SE0132 100.0% 99.9%  **DYLTADEEDSYVVAQANSRLDENGRFLDDEVVCRFRGNNTVMAKEKMDYMDVSPKQVVSAATACIPFLENDDSNRALMGA**

26 R5981 100.0% 100.0%  **DYLTADEEDSYVVAQANSRLDENGRFLDDEVVCRFRGNNTVMAKEKMDYMDVSPKQVVSAATACIPFLENDDSNRALMGA**

27 JH-S-3 100.0% 100.0%  **DYLTADEEDSYVVAQANSRLDENGRFLDDEVVCRFRGNNTVMAKEKMDYMDVSPKQVVSAATACIPFLENDDSNRALMGA**

28 JH-S-1 100.0% 100.0%  **DYLTADEEDSYVVAQANSRLDENGRFLDDEVVCRFRGNNTVMAKEKMDYMDVSPKQVVSAATACIPFLENDDSNRALMGA**

29 s2 100.0% 99.9%  **DYLTADEEDSYVVAQANSRLEENGRFLDDEVVCRFRGNNTVMAKEKMDYMDVSPKQVVSAATACIPFLENDDSNRALMGA**

30 S43 100.0% 100.0%  **DYLTADEEDSYVVAQANSRLDENGRFLDDEVVCRFRGNNTVMAKEKMDYMDVSPKQVVSAATACIPFLENDDSNRALMGA**

31 S17W 100.0% 100.0%  **DYLTADEEDSYVVAQANSRLDENGRFLDDEVVCRFRGNNTVMAKEKMDYMDVSPKQVVSAATACIPFLENDDSNRALMGA**

32 48 100.0% 99.9%  **DYLTADEEDSYVVAQANSRLDENGRFLDDEVVCRFRGNNTVMAKEKMDYMDVSPKQVVSAATACIPFLENDDSNRALMGA**

33 ABKVF 100.0% 99.9%  **DYLTADEEDSYVVAQANSRLDENGRFLDDEVVCRFRGNNTVMAKEKMDYMDVSPKQVVSAATACIPFLENDDSNRALMGA**

34 CICARIA 100.0% 100.0%  **DYLTADEEDSYVVAQANSRLDENGRFLDDEVVCRFRGNNTVMAKEKMDYMDVSPKQVVSAATACIPFLENDDSNRALMGA**

35 SKN25lux 100.0% 100.0%  **DYLTADEEDSYVVAQANSRLDENGRFLDDEVVCRFRGNNTVMAKEKMDYMDVSPKQVVSAATACIPFLENDDSNRALMGA**

36 HMSC074F11 100.0% 100.0%  **DYLTADEEDSYVVAQANSRLDENGRFLDDEVVCRFRGNNTVMAKEKMDYMDVSPKQVVSAATACIPFLENDDSNRALMGA**

37 HMSC068G11 100.0% 100.0%  **DYLTADEEDSYVVAQANSRLDENGRFLDDEVVCRFRGNNTVMAKEKMDYMDVSPKQVVSAATACIPFLENDDSNRALMGA**

38 NIHLM037 100.0% 99.9%  **DYLTADEEDSYVVAQANSRLDENGRFLDDEVVCRFRGNNTVMAKEKMDYMDVSPKQVVSAATACIPFLENDDSNRALMGA**

39 NIHLM015 100.0% 100.0%  **DYLTADEEDSYVVAQANSRLDENGRFLDDEVVCRFRGNNTVMAKEKMDYMDVSPKQVVSAATACIPFLENDDSNRALMGA**

consensus/100%  **DYLTADEEDSYVVAQANSRL-ENGRFLDDEVVCRFRGNNTVMAKEKMDYMDVSPKQVVSAATACIPFLENDDSNRALMGA**

consensus/90%  **DYLTADEEDSYVVAQANSRLDENGRFLDDEVVCRFRGNNTVMAKEKMDYMDVSPKQVVSAATACIPFLENDDSNRALMGA**

consensus/80%  **DYLTADEEDSYVVAQANSRLDENGRFLDDEVVCRFRGNNTVMAKEKMDYMDVSPKQVVSAATACIPFLENDDSNRALMGA**

consensus/70%  **DYLTADEEDSYVVAQANSRLDENGRFLDDEVVCRFRGNNTVMAKEKMDYMDVSPKQVVSAATACIPFLENDDSNRALMGA**

cov pid  **641**  **: . . . . 7 . .** **720**

1 ATCC35984 100.0% 100.0%  **NMQRQAVPLMNPEAPFVGTGMEHVAARDSGAAITAKHRGRVEHVESNEILVRRLVEENGTEHEGELDRYPLAKFKRSNSG**

2 ATCC12228 100.0% 100.0%  **NMQRQAVPLMNPEAPFVGTGMEHVAARDSGAAITAKHRGRVEHVESNEILVRRLVEENGTEHEGELDRYPLAKFKRSNSG**

3 36AM 94.9% 100.0%  **NMQRQAVPLMNPEAPFVGTGMEHVAARDSGAAITAKHRGRVEHVESNEILVRRLVEENGTEHEGELDRYPLAKFKRSNSG**

4 48AF 100.0% 100.0%  **NMQRQAVPLMNPEAPFVGTGMEHVAARDSGAAITAKHRGRVEHVESNEILVRRLVEENGTEHEGELDRYPLAKFKRSNSG**

5 54AF 100.0% 99.9%  **NMQRQAVPLMNPEAPFVGTGMEHVAARDSGAAITAKHRGRVEHVESNEILVRRLVEENGTEHEGELDRYPLAKFKRSNSG**

6 785_SEPI 100.0% 99.9%  **NMQRQAVPLMNPEAPFVGTGMEHVAARDSGAAITAKHRGRVEHVESNEILVRRLVEENGTEHEGELDRYPLAKFKRSNSG**

7 VSE49 100.0% 100.0%  **NMQRQAVPLMNPEAPFVGTGMEHVAARDSGAAITAKHRGRVEHVESNEILVRRLVEENGTEHEGELDRYPLAKFKRSNSG**

8 VSE57 100.0% 100.0%  **NMQRQAVPLMNPEAPFVGTGMEHVAARDSGAAITAKHRGRVEHVESNEILVRRLVEENGTEHEGELDRYPLAKFKRSNSG**

9 SE45 100.0% 100.0%  **NMQRQAVPLMNPEAPFVGTGMEHVAARDSGAAITAKHRGRVEHVESNEILVRRLVEENGTEHEGELDRYPLAKFKRSNSG**

10 SE40 100.0% 100.0%  **NMQRQAVPLMNPEAPFVGTGMEHVAARDSGAAITAKHRGRVEHVESNEILVRRLVEENGTEHEGELDRYPLAKFKRSNSG**

11 32A 100.0% 99.2%  **NMQRQAVPLMNPEAPFVGTGMEHVAARDSGAAITAKHRGRVEHVESNEILVRRLVEENGTEHEGELDRYPLAKFKRSNSG**

12 HD66 100.0% 100.0%  **NMQRQAVPLMNPEAPFVGTGMEHVAARDSGAAITAKHRGRVEHVESNEILVRRLVEENGTEHEGELDRYPLAKFKRSNSG**

13 HD43 100.0% 99.8%  **NMQRQAVPLMNPEAPFVGTGMEHVAARDSGAAITAKHRGRVEHVESNEILVRRLVEENGTEHEGELDRYPLAKFKRSNSG**

14 HD33 100.0% 99.9%  **NMQRQAVPLMNPEAPFVGTGMEHVAARDSGAAITAKHRGRVEHVESNEILVRRLVEENGTEHEGELDRYPLAKFKRSNSG**

15 DE0525 100.0% 100.0%  **NMQRQAVPLMNPEAPFVGTGMEHVAARDSGAAITAKHRGRVEHVESNEILVRRLVEENGTEHEGELDRYPLAKFKRSNSG**

16 4928STDY7071543 94.9% 100.0%  **NMQRQAVPLMNPEAPFVGTGMEHVAARDSGAAITAKHRGRVEHVESNEILVRRLVEENGTEHEGELDRYPLAKFKRSNSG**

17 BAV2502 94.9% 99.9%  **NMQRQAVPLMNPEAPFVGTGMEHVAARDSGAAITAKHRGRVEHVESNEILVRRLVEENGTEHEGELDRYPLAKFKRSNSG**

18 APC3784 100.0% 100.0%  **NMQRQAVPLMNPEAPFVGTGMEHVAARDSGAAITAKHRGRVEHVESNEILVRRLVEENGTEHEGELDRYPLAKFKRSNSG**

19 SESURV_p1_0557 100.0% 100.0%  **NMQRQAVPLMNPEAPFVGTGMEHVAARDSGAAITAKHRGRVEHVESNEILVRRLVEENGTEHEGELDRYPLAKFKRSNSG**

20 SESURV_p1_0612 100.0% 100.0%  **NMQRQAVPLMNPEAPFVGTGMEHVAARDSGAAITAKHRGRVEHVESNEILVRRLVEENGTEHEGELDRYPLAKFKRSNSG**

21 SESURV_p4_1553 100.0% 100.0%  **NMQRQAVPLMNPEAPFVGTGMEHVAARDSGAAITAKHRGRVEHVESNEILVRRLVEENGTEHEGELDRYPLAKFKRSNSG**

22 SESURV_p1_1200 100.0% 100.0%  **NMQRQAVPLMNPEAPFVGTGMEHVAARDSGAAITAKHRGRVEHVESNEILVRRLVEENGTEHEGELDRYPLAKFKRSNSG**

23 SESURV_p3_0825 100.0% 100.0%  **NMQRQAVPLMNPEAPFVGTGMEHVAARDSGAAITAKHRGRVEHVESNEILVRRLVEENGTEHEGELDRYPLAKFKRSNSG**

24 JH 100.0% 100.0%  **NMQRQAVPLMNPEAPFVGTGMEHVAARDSGAAITAKHRGRVEHVESNEILVRRLVEENGTEHEGELDRYPLAKFKRSNSG**

25 Z0118SE0132 100.0% 99.9%  **NMQRQAVPLMNPEAPFVGTGMEHVAARDSGAAITAKHRGRVEHVESNEILVRRLVEENGTEHEGELDRYPLAKFKRSNSG**

26 R5981 100.0% 100.0%  **NMQRQAVPLMNPEAPFVGTGMEHVAARDSGAAITAKHRGRVEHVESNEILVRRLVEENGTEHEGELDRYPLAKFKRSNSG**

27 JH-S-3 100.0% 100.0%  **NMQRQAVPLMNPEAPFVGTGMEHVAARDSGAAITAKHRGRVEHVESNEILVRRLVEENGTEHEGELDRYPLAKFKRSNSG**

28 JH-S-1 100.0% 100.0%  **NMQRQAVPLMNPEAPFVGTGMEHVAARDSGAAITAKHRGRVEHVESNEILVRRLVEENGTEHEGELDRYPLAKFKRSNSG**

29 s2 100.0% 99.9%  **NMQRQAVPLMNPEAPFVGTGMEHVAARDSGAAITAKHRGRVEHVESNEILVRRLVEENGTEHEGELDRYPLAKFKRSNSG**

30 S43 100.0% 100.0%  **NMQRQAVPLMNPEAPFVGTGMEHVAARDSGAAITAKHRGRVEHVESNEILVRRLVEENGTEHEGELDRYPLAKFKRSNSG**

31 S17W 100.0% 100.0%  **NMQRQAVPLMNPEAPFVGTGMEHVAARDSGAAITAKHRGRVEHVESNEILVRRLVEENGTEHEGELDRYPLAKFKRSNSG**

32 48 100.0% 99.9%  **NMQRQAVPLMNPEAPFVGTGMEHVAARDSGAAITAKHRGRVEHVESNEILVRRLVEENGTEHEGELDRYPLAKFKRSNSG**

33 ABKVF 100.0% 99.9%  **NMQRQAVPLMNPEAPFVGTGMEHVAARDSGAAITAKHRGRVEHVESNEILVRRLVEENGTEHEGELDRYPLAKFKRSNSG**

34 CICARIA 100.0% 100.0%  **NMQRQAVPLMNPEAPFVGTGMEHVAARDSGAAITAKHRGRVEHVESNEILVRRLVEENGTEHEGELDRYPLAKFKRSNSG**

35 SKN25lux 100.0% 100.0%  **NMQRQAVPLMNPEAPFVGTGMEHVAARDSGAAITAKHRGRVEHVESNEILVRRLVEENGTEHEGELDRYPLAKFKRSNSG**

36 HMSC074F11 100.0% 100.0%  **NMQRQAVPLMNPEAPFVGTGMEHVAARDSGAAITAKHRGRVEHVESNEILVRRLVEENGTEHEGELDRYPLAKFKRSNSG**

37 HMSC068G11 100.0% 100.0%  **NMQRQAVPLMNPEAPFVGTGMEHVAARDSGAAITAKHRGRVEHVESNEILVRRLVEENGTEHEGELDRYPLAKFKRSNSG**

38 NIHLM037 100.0% 99.9%  **NMQRQAVPLMNPEAPFVGTGMEHVAARDSGAAITAKHRGRVEHVESNEILVRRLVEENGTEHEGELDRYPLAKFKRSNSG**

39 NIHLM015 100.0% 100.0%  **NMQRQAVPLMNPEAPFVGTGMEHVAARDSGAAITAKHRGRVEHVESNEILVRRLVEENGTEHEGELDRYPLAKFKRSNSG**

consensus/100%  **NMQRQAVPLMNPEAPFVGTGMEHVAARDSGAAITAKHRGRVEHVESNEILVRRLVEENGTEHEGELDRYPLAKFKRSNSG**

consensus/90%  **NMQRQAVPLMNPEAPFVGTGMEHVAARDSGAAITAKHRGRVEHVESNEILVRRLVEENGTEHEGELDRYPLAKFKRSNSG**

consensus/80%  **NMQRQAVPLMNPEAPFVGTGMEHVAARDSGAAITAKHRGRVEHVESNEILVRRLVEENGTEHEGELDRYPLAKFKRSNSG**

consensus/70%  **NMQRQAVPLMNPEAPFVGTGMEHVAARDSGAAITAKHRGRVEHVESNEILVRRLVEENGTEHEGELDRYPLAKFKRSNSG**

cov pid  **721**  **. . : . . . . 8** **800**

1 ATCC35984 100.0% 100.0%  **TCYNQRPIVSIGDVVEYNEILADGPSMELGEMALGRNVVVGFMTWDGYNYEDAVIMSERLVKDD---VYTSIHIEEYESE**

2 ATCC12228 100.0% 100.0%  **TCYNQRPIVSIGDVVEYNEILADGPSMELGEMALGRNVVVGFMTWDGYNYEDAVIMSERLVKDD---VYTSIHIEEYESE**

3 36AM 94.9% 100.0%  **TCYNQRPIVSIGDVVEYNEILADGPSMELGEMALGRNVVVGFMTWDGYNYEDAVIMSERLVKDD---VYTSIHIEEYESE**

4 48AF 100.0% 100.0%  **TCYNQRPIVSIGDVVEYNEILADGPSMELGEMALGRNVVVGFMTWDGYNYEDAVIMSERLVKDD---VYTSIHIEEYESE**

5 54AF 100.0% 99.9%  **TCYNQRPIVSIGDVVESNEILADGPSMELGEMALGRNVVVGFMTWDGYNYEDAVIMSERLVKDD---VYTSIHIEEYESE**

6 785_SEPI 100.0% 99.9%  **TCYNQRPIVSIGDVVESNEILADGPSMELGEMALGRNVVVGFMTWDGYNYEDAVIMSERLVKDD---VYTSIHIEEYESE**

7 VSE49 100.0% 100.0%  **TCYNQRPIVSIGDVVEYNEILADGPSMELGEMALGRNVVVGFMTWDGYNYEDAVIMSERLVKDD---VYTSIHIEEYESE**

8 VSE57 100.0% 100.0%  **TCYNQRPIVSIGDVVEYNEILADGPSMELGEMALGRNVVVGFMTWDGYNYEDAVIMSERLVKDD---VYTSIHIEEYESE**

9 SE45 100.0% 100.0%  **TCYNQRPIVSIGDVVEYNEILADGPSMELGEMALGRNVVVGFMTWDGYNYEDAVIMSERLVKDD---VYTSIHIEEYESE**

10 SE40 100.0% 100.0%  **TCYNQRPIVSIGDVVEYNEILADGPSMELGEMALGRNVVVGFMTWDGYNYEDAVIMSERLVKDD---VYTSIHIEEYESE**

11 32A 100.0% 99.2%  **TCYNQRPIVSIGDVVEYNEILADGPSMELGEMALGRNVVVGFMTWDGYNYEDAVIMSERKKKKKKIXXXXSIHIEEYESE**

12 HD66 100.0% 100.0%  **TCYNQRPIVSIGDVVEYNEILADGPSMELGEMALGRNVVVGFMTWDGYNYEDAVIMSERLVKDD---VYTSIHIEEYESE**

13 HD43 100.0% 99.8%  **TCYNQRPIVSIGDVVEYNEILADGPSMELGEMALGRNVVVGFMTWDGYNYEDAVIMSERLVKDD---VYTSIHIEEYESE**

14 HD33 100.0% 99.9%  **TCYNQRPIVSIGDVVEYNEILADGPSMELGEMALGRNVVVGFMTWDGYNYEDAVIMSERLVKDD---VYTSIHIEEYESE**

15 DE0525 100.0% 100.0%  **TCYNQRPIVSIGDVVEYNEILADGPSMELGEMALGRNVVVGFMTWDGYNYEDAVIMSERLVKDD---VYTSIHIEEYESE**

16 4928STDY7071543 94.9% 100.0%  **TCYNQRPIVSIGDVVEYNEILADGPSMELGEMALGRNVVVGFMTWDGYNYEDAVIMSERLVKDD---VYTSIHIEEYESE**

17 BAV2502 94.9% 99.9%  **TCYNQRPIVSIGDVVESNEILADGPSMELGEMALGRNVVVGFMTWDGYNYEDAVIMSERLVKDD---VYTSIHIEEYESE**

18 APC3784 100.0% 100.0%  **TCYNQRPIVSIGDVVEYNEILADGPSMELGEMALGRNVVVGFMTWDGYNYEDAVIMSERLVKDD---VYTSIHIEEYESE**

19 SESURV_p1_0557 100.0% 100.0%  **TCYNQRPIVSIGDVVEYNEILADGPSMELGEMALGRNVVVGFMTWDGYNYEDAVIMSERLVKDD---VYTSIHIEEYESE**

20 SESURV_p1_0612 100.0% 100.0%  **TCYNQRPIVSIGDVVEYNEILADGPSMELGEMALGRNVVVGFMTWDGYNYEDAVIMSERLVKDD---VYTSIHIEEYESE**

21 SESURV_p4_1553 100.0% 100.0%  **TCYNQRPIVSIGDVVEYNEILADGPSMELGEMALGRNVVVGFMTWDGYNYEDAVIMSERLVKDD---VYTSIHIEEYESE**

22 SESURV_p1_1200 100.0% 100.0%  **TCYNQRPIVSIGDVVEYNEILADGPSMELGEMALGRNVVVGFMTWDGYNYEDAVIMSERLVKDD---VYTSIHIEEYESE**

23 SESURV_p3_0825 100.0% 100.0%  **TCYNQRPIVSIGDVVEYNEILADGPSMELGEMALGRNVVVGFMTWDGYNYEDAVIMSERLVKDD---VYTSIHIEEYESE**

24 JH 100.0% 100.0%  **TCYNQRPIVSIGDVVEYNEILADGPSMELGEMALGRNVVVGFMTWDGYNYEDAVIMSERLVKDD---VYTSIHIEEYESE**

25 Z0118SE0132 100.0% 99.9%  **TCYNQRPIVSIGDVVEYNEILADGPSMELGEMALGRNVVVGFMTWDGYNYEDAVIMSERLVKDD---VYTSIHIEEYESE**

26 R5981 100.0% 100.0%  **TCYNQRPIVSIGDVVEYNEILADGPSMELGEMALGRNVVVGFMTWDGYNYEDAVIMSERLVKDD---VYTSIHIEEYESE**

27 JH-S-3 100.0% 100.0%  **TCYNQRPIVSIGDVVEYNEILADGPSMELGEMALGRNVVVGFMTWDGYNYEDAVIMSERLVKDD---VYTSIHIEEYESE**

28 JH-S-1 100.0% 100.0%  **TCYNQRPIVSIGDVVEYNEILADGPSMELGEMALGRNVVVGFMTWDGYNYEDAVIMSERLVKDD---VYTSIHIEEYESE**

29 s2 100.0% 99.9%  **TCYNQRPIVSIGDVVEYNEILADGPSMELGEMALGRNVVVGFMTWDGYNYEDAVIMSERLVKDD---VYTSIHIEEYESE**

30 S43 100.0% 100.0%  **TCYNQRPIVSIGDVVEYNEILADGPSMELGEMALGRNVVVGFMTWDGYNYEDAVIMSERLVKDD---VYTSIHIEEYESE**

31 S17W 100.0% 100.0%  **TCYNQRPIVSIGDVVEYNEILADGPSMELGEMALGRNVVVGFMTWDGYNYEDAVIMSERLVKDD---VYTSIHIEEYESE**

32 48 100.0% 99.9%  **TCYNQRPIVSIGDVVESNEILADGPSMELGEMALGRNVVVGFMTWDGYNYEDAVIMSERLVKDD---VYTSIHIEEYESE**

33 ABKVF 100.0% 99.9%  **TCYNQRPIVSIGDVVEYNEILADGPSMELGEMALGRNVVVGFMTWDGYNYEDAVIMSERLVKDD---VYTSIHIEEYESE**

34 CICARIA 100.0% 100.0%  **TCYNQRPIVSIGDVVEYNEILADGPSMELGEMALGRNVVVGFMTWDGYNYEDAVIMSERLVKDD---VYTSIHIEEYESE**

35 SKN25lux 100.0% 100.0%  **TCYNQRPIVSIGDVVEYNEILADGPSMELGEMALGRNVVVGFMTWDGYNYEDAVIMSERLVKDD---VYTSIHIEEYESE**

36 HMSC074F11 100.0% 100.0%  **TCYNQRPIVSIGDVVEYNEILADGPSMELGEMALGRNVVVGFMTWDGYNYEDAVIMSERLVKDD---VYTSIHIEEYESE**

37 HMSC068G11 100.0% 100.0%  **TCYNQRPIVSIGDVVEYNEILADGPSMELGEMALGRNVVVGFMTWDGYNYEDAVIMSERLVKDD---VYTSIHIEEYESE**

38 NIHLM037 100.0% 99.9%  **TCYNQRPIVSIGDVVEYNEILADGPSMELGEMALGRNVVVGFMTWDGYNYEDAVIMSERLVKDD---VYTSIHIEEYESE**

39 NIHLM015 100.0% 100.0%  **TCYNQRPIVSIGDVVEYNEILADGPSMELGEMALGRNVVVGFMTWDGYNYEDAVIMSERLVKDD---VYTSIHIEEYESE**

consensus/100%  **TCYNQRPIVSIGDVVE.NEILADGPSMELGEMALGRNVVVGFMTWDGYNYEDAVIMSERhhKcc......SIHIEEYESE**

consensus/90%  **TCYNQRPIVSIGDVVE.NEILADGPSMELGEMALGRNVVVGFMTWDGYNYEDAVIMSERLVKDD...VYTSIHIEEYESE**

consensus/80%  **TCYNQRPIVSIGDVVEYNEILADGPSMELGEMALGRNVVVGFMTWDGYNYEDAVIMSERLVKDD...VYTSIHIEEYESE**

consensus/70%  **TCYNQRPIVSIGDVVEYNEILADGPSMELGEMALGRNVVVGFMTWDGYNYEDAVIMSERLVKDD...VYTSIHIEEYESE**

cov pid  **801**  **. . . . : . . .** **880**

1 ATCC35984 100.0% 100.0%  **ARDTKLGPEEITRDIPNVSESALKNLDDRGIVYVGAEVKDGDILVGKVTPKGVTELTAEERLLHAIFGEKAREVRDTSLR**

2 ATCC12228 100.0% 100.0%  **ARDTKLGPEEITRDIPNVSESALKNLDDRGIVYVGAEVKDGDILVGKVTPKGVTELTAEERLLHAIFGEKAREVRDTSLR**

3 36AM 94.9% 100.0%  **ARDTKLGPEEITRDIPNVSESALKNLDDRGIVYVGAEVKDGDILVGKVTPKGVTELTAEERLLHAIFGEKAREVRDTSLR**

4 48AF 100.0% 100.0%  **ARDTKLGPEEITRDIPNVSESALKNLDDRGIVYVGAEVKDGDILVGKVTPKGVTELTAEERLLHAIFGEKAREVRDTSLR**

5 54AF 100.0% 99.9%  **ARDTKLGPEEITRDIPNVSESALKNLDDRGIVYVGAEVKDGDILVGKVTPKGVTELTAEERLLHAIFGEKAREVRDTSLR**

6 785_SEPI 100.0% 99.9%  **ARDTKLGPEEITRDIPNVSESALKNLDDRGIVYVGAEVKDGDILVGKVTPKGVTELTAEERLLHAIFGEKAREVRDTSLR**

7 VSE49 100.0% 100.0%  **ARDTKLGPEEITRDIPNVSESALKNLDDRGIVYVGAEVKDGDILVGKVTPKGVTELTAEERLLHAIFGEKAREVRDTSLR**

8 VSE57 100.0% 100.0%  **ARDTKLGPEEITRDIPNVSESALKNLDDRGIVYVGAEVKDGDILVGKVTPKGVTELTAEERLLHAIFGEKAREVRDTSLR**

9 SE45 100.0% 100.0%  **ARDTKLGPEEITRDIPNVSESALKNLDDRGIVYVGAEVKDGDILVGKVTPKGVTELTAEERLLHAIFGEKAREVRDTSLR**

10 SE40 100.0% 100.0%  **ARDTKLGPEEITRDIPNVSESALKNLDDRGIVYVGAEVKDGDILVGKVTPKGVTELTAEERLLHAIFGEKAREVRDTSLR**

11 32A 100.0% 99.2%  **ARDTKLGPEEITRDIPNVSESALKNLDDRGIVYVGAEVKDGDILVGKVTPKGVTELTAEERLLHAIFGEKAREVRDTSLR**

12 HD66 100.0% 100.0%  **ARDTKLGPEEITRDIPNVSESALKNLDDRGIVYVGAEVKDGDILVGKVTPKGVTELTAEERLLHAIFGEKAREVRDTSLR**

13 HD43 100.0% 99.8%  **ARDTKLGPEEITRDIPNVSESALKNLDDRGIVYVGAEVKDGDILVGKVTPKGVTELTAEERLLHAIFGEKAREVRDTSLR**

14 HD33 100.0% 99.9%  **ARDTKLGPEEITRDIPNVSESALKNLDDRGIVYVGAEVKDGDILVGKVTPKGVTELTAEERLLHAIFGEKAREVRDTSLR**

15 DE0525 100.0% 100.0%  **ARDTKLGPEEITRDIPNVSESALKNLDDRGIVYVGAEVKDGDILVGKVTPKGVTELTAEERLLHAIFGEKAREVRDTSLR**

16 4928STDY7071543 94.9% 100.0%  **ARDTKLGPEEITRDIPNVSESALKNLDDRGIVYVGAEVKDGDILVGKVTPKGVTELTAEERLLHAIFGEKAREVRDTSLR**

17 BAV2502 94.9% 99.9%  **ARDTKLGPEEITRDIPNVSESALKNLDDRGIVYVGAEVKDGDILVGKVTPKGVTELTAEERLLHAIFGEKAREVRDTSLR**

18 APC3784 100.0% 100.0%  **ARDTKLGPEEITRDIPNVSESALKNLDDRGIVYVGAEVKDGDILVGKVTPKGVTELTAEERLLHAIFGEKAREVRDTSLR**

19 SESURV_p1_0557 100.0% 100.0%  **ARDTKLGPEEITRDIPNVSESALKNLDDRGIVYVGAEVKDGDILVGKVTPKGVTELTAEERLLHAIFGEKAREVRDTSLR**

20 SESURV_p1_0612 100.0% 100.0%  **ARDTKLGPEEITRDIPNVSESALKNLDDRGIVYVGAEVKDGDILVGKVTPKGVTELTAEERLLHAIFGEKAREVRDTSLR**

21 SESURV_p4_1553 100.0% 100.0%  **ARDTKLGPEEITRDIPNVSESALKNLDDRGIVYVGAEVKDGDILVGKVTPKGVTELTAEERLLHAIFGEKAREVRDTSLR**

22 SESURV_p1_1200 100.0% 100.0%  **ARDTKLGPEEITRDIPNVSESALKNLDDRGIVYVGAEVKDGDILVGKVTPKGVTELTAEERLLHAIFGEKAREVRDTSLR**

23 SESURV_p3_0825 100.0% 100.0%  **ARDTKLGPEEITRDIPNVSESALKNLDDRGIVYVGAEVKDGDILVGKVTPKGVTELTAEERLLHAIFGEKAREVRDTSLR**

24 JH 100.0% 100.0%  **ARDTKLGPEEITRDIPNVSESALKNLDDRGIVYVGAEVKDGDILVGKVTPKGVTELTAEERLLHAIFGEKAREVRDTSLR**

25 Z0118SE0132 100.0% 99.9%  **ARDTKLGPEEITRDIPNVSESALKNLDDRGIVYVGAEVKDGDILVGKVTPKGVTELTAEERLLHAIFGEKAREVRDTSLR**

26 R5981 100.0% 100.0%  **ARDTKLGPEEITRDIPNVSESALKNLDDRGIVYVGAEVKDGDILVGKVTPKGVTELTAEERLLHAIFGEKAREVRDTSLR**

27 JH-S-3 100.0% 100.0%  **ARDTKLGPEEITRDIPNVSESALKNLDDRGIVYVGAEVKDGDILVGKVTPKGVTELTAEERLLHAIFGEKAREVRDTSLR**

28 JH-S-1 100.0% 100.0%  **ARDTKLGPEEITRDIPNVSESALKNLDDRGIVYVGAEVKDGDILVGKVTPKGVTELTAEERLLHAIFGEKAREVRDTSLR**

29 s2 100.0% 99.9%  **ARDTKLGPEEITRDIPNVSESALKNLDDRGIVYVGAEVKDGDILVGKVTPKGVTELTAEERLLHAIFGEKAREVRDTSLR**

30 S43 100.0% 100.0%  **ARDTKLGPEEITRDIPNVSESALKNLDDRGIVYVGAEVKDGDILVGKVTPKGVTELTAEERLLHAIFGEKAREVRDTSLR**

31 S17W 100.0% 100.0%  **ARDTKLGPEEITRDIPNVSESALKNLDDRGIVYVGAEVKDGDILVGKVTPKGVTELTAEERLLHAIFGEKAREVRDTSLR**

32 48 100.0% 99.9%  **ARDTKLGPEEITRDIPNVSESALKNLDDRGIVYVGAEVKDGDILVGKVTPKGVTELTAEERLLHAIFGEKAREVRDTSLR**

33 ABKVF 100.0% 99.9%  **ARDTKLGPEEITRDIPNVSESALKNLDDRGIVYVGAEVKDGDILVGKVTPKGVTELTAEERLLHAIFGEKAREVRDTSLR**

34 CICARIA 100.0% 100.0%  **ARDTKLGPEEITRDIPNVSESALKNLDDRGIVYVGAEVKDGDILVGKVTPKGVTELTAEERLLHAIFGEKAREVRDTSLR**

35 SKN25lux 100.0% 100.0%  **ARDTKLGPEEITRDIPNVSESALKNLDDRGIVYVGAEVKDGDILVGKVTPKGVTELTAEERLLHAIFGEKAREVRDTSLR**

36 HMSC074F11 100.0% 100.0%  **ARDTKLGPEEITRDIPNVSESALKNLDDRGIVYVGAEVKDGDILVGKVTPKGVTELTAEERLLHAIFGEKAREVRDTSLR**

37 HMSC068G11 100.0% 100.0%  **ARDTKLGPEEITRDIPNVSESALKNLDDRGIVYVGAEVKDGDILVGKVTPKGVTELTAEERLLHAIFGEKAREVRDTSLR**

38 NIHLM037 100.0% 99.9%  **ARDTKLGPEEITRDIPNVSESALKNLDDRGIVYVGAEVKDGDILVGKVTPKGVTELTAEERLLHAIFGEKAREVRDTSLR**

39 NIHLM015 100.0% 100.0%  **ARDTKLGPEEITRDIPNVSESALKNLDDRGIVYVGAEVKDGDILVGKVTPKGVTELTAEERLLHAIFGEKAREVRDTSLR**

consensus/100%  **ARDTKLGPEEITRDIPNVSESALKNLDDRGIVYVGAEVKDGDILVGKVTPKGVTELTAEERLLHAIFGEKAREVRDTSLR**

consensus/90%  **ARDTKLGPEEITRDIPNVSESALKNLDDRGIVYVGAEVKDGDILVGKVTPKGVTELTAEERLLHAIFGEKAREVRDTSLR**

consensus/80%  **ARDTKLGPEEITRDIPNVSESALKNLDDRGIVYVGAEVKDGDILVGKVTPKGVTELTAEERLLHAIFGEKAREVRDTSLR**

consensus/70%  **ARDTKLGPEEITRDIPNVSESALKNLDDRGIVYVGAEVKDGDILVGKVTPKGVTELTAEERLLHAIFGEKAREVRDTSLR**

cov pid  **881**  **. 9 . . . . : .** **960**

1 ATCC35984 100.0% 100.0%  **VPHGAGGIVLDVKVFNREEGDDTLSPGVNQLVRVYIVQKRKIHVGDKMCGRHGNKGVISKIVPEEDMPYLPDGRPIDIML**

2 ATCC12228 100.0% 100.0%  **VPHGAGGIVLDVKVFNREEGDDTLSPGVNQLVRVYIVQKRKIHVGDKMCGRHGNKGVISKIVPEEDMPYLPDGRPIDIML**

3 36AM 94.9% 100.0%  **VPHGAGGIVLDVKVFNREEGDDTLSPGVNQLVRVYIVQKRKIHVGDKMCGRHGNKGVISKIVPEEDMPYLPDGRPIDIML**

4 48AF 100.0% 100.0%  **VPHGAGGIVLDVKVFNREEGDDTLSPGVNQLVRVYIVQKRKIHVGDKMCGRHGNKGVISKIVPEEDMPYLPDGRPIDIML**

5 54AF 100.0% 99.9%  **VPHGAGGIVLDVKVFNREEGDDTLSPGVNQLVRVYIVQKRKIHVGDKMCGRHGNKGVISKIVPEEDMPYLPDGRPIDIML**

6 785_SEPI 100.0% 99.9%  **VPHGAGGIVLDVKVFNREEGDDTLSPGVNQLVRVYIVQKRKIHVGDKMCGRHGNKGVISKIVPEEDMPYLPDGRPIDIML**

7 VSE49 100.0% 100.0%  **VPHGAGGIVLDVKVFNREEGDDTLSPGVNQLVRVYIVQKRKIHVGDKMCGRHGNKGVISKIVPEEDMPYLPDGRPIDIML**

8 VSE57 100.0% 100.0%  **VPHGAGGIVLDVKVFNREEGDDTLSPGVNQLVRVYIVQKRKIHVGDKMCGRHGNKGVISKIVPEEDMPYLPDGRPIDIML**

9 SE45 100.0% 100.0%  **VPHGAGGIVLDVKVFNREEGDDTLSPGVNQLVRVYIVQKRKIHVGDKMCGRHGNKGVISKIVPEEDMPYLPDGRPIDIML**

10 SE40 100.0% 100.0%  **VPHGAGGIVLDVKVFNREEGDDTLSPGVNQLVRVYIVQKRKIHVGDKMCGRHGNKGVISKIVPEEDMPYLPDGRPIDIML**

11 32A 100.0% 99.2%  **VPHGAGGIVLDVKVFNREEGDDTLSPGVNQLVRVYIVQKRKIHVGDKMCGRHGNKGVISKIVPEEDMPYLPDGRPIDIML**

12 HD66 100.0% 100.0%  **VPHGAGGIVLDVKVFNREEGDDTLSPGVNQLVRVYIVQKRKIHVGDKMCGRHGNKGVISKIVPEEDMPYLPDGRPIDIML**

13 HD43 100.0% 99.8%  **VPHGAGGIVLDVKVFNREEGDDTLSPGVNQLVRVYIVQKRKIHVGDKMCGRHGNKGVISKIVPEEDMPYLPDGRPIDIML**

14 HD33 100.0% 99.9%  **VPHGAGGIVLDVKVFNREEGDDTLSPGVNQLVRVYIVQKRKIHVGDKMCGRHGNKGVISKIVPEEDMPYLPDGRPIDIIL**

15 DE0525 100.0% 100.0%  **VPHGAGGIVLDVKVFNREEGDDTLSPGVNQLVRVYIVQKRKIHVGDKMCGRHGNKGVISKIVPEEDMPYLPDGRPIDIML**

16 4928STDY7071543 94.9% 100.0%  **VPHGAGGIVLDVKVFNREEGDDTLSPGVNQLVRVYIVQKRKIHVGDKMCGRHGNKGVISKIVPEEDMPYLPDGRPIDIML**

17 BAV2502 94.9% 99.9%  **VPHGAGGIVLDVKVFNREEGDDTLSPGVNQLVRVYIVQKRKIHVGDKMCGRHGNKGVISKIVPEEDMPYLPDGRPIDIML**

18 APC3784 100.0% 100.0%  **VPHGAGGIVLDVKVFNREEGDDTLSPGVNQLVRVYIVQKRKIHVGDKMCGRHGNKGVISKIVPEEDMPYLPDGRPIDIML**

19 SESURV_p1_0557 100.0% 100.0%  **VPHGAGGIVLDVKVFNREEGDDTLSPGVNQLVRVYIVQKRKIHVGDKMCGRHGNKGVISKIVPEEDMPYLPDGRPIDIML**

20 SESURV_p1_0612 100.0% 100.0%  **VPHGAGGIVLDVKVFNREEGDDTLSPGVNQLVRVYIVQKRKIHVGDKMCGRHGNKGVISKIVPEEDMPYLPDGRPIDIML**

21 SESURV_p4_1553 100.0% 100.0%  **VPHGAGGIVLDVKVFNREEGDDTLSPGVNQLVRVYIVQKRKIHVGDKMCGRHGNKGVISKIVPEEDMPYLPDGRPIDIML**

22 SESURV_p1_1200 100.0% 100.0%  **VPHGAGGIVLDVKVFNREEGDDTLSPGVNQLVRVYIVQKRKIHVGDKMCGRHGNKGVISKIVPEEDMPYLPDGRPIDIML**

23 SESURV_p3_0825 100.0% 100.0%  **VPHGAGGIVLDVKVFNREEGDDTLSPGVNQLVRVYIVQKRKIHVGDKMCGRHGNKGVISKIVPEEDMPYLPDGRPIDIML**

24 JH 100.0% 100.0%  **VPHGAGGIVLDVKVFNREEGDDTLSPGVNQLVRVYIVQKRKIHVGDKMCGRHGNKGVISKIVPEEDMPYLPDGRPIDIML**

25 Z0118SE0132 100.0% 99.9%  **VPHGAGGIVLDVKVFNREEGDDTLSPGVNQLVRVYIVQKRKIHVGDKMCGRHGNKGVISKIVPEEDMPYLPDGRPIDIML**

26 R5981 100.0% 100.0%  **VPHGAGGIVLDVKVFNREEGDDTLSPGVNQLVRVYIVQKRKIHVGDKMCGRHGNKGVISKIVPEEDMPYLPDGRPIDIML**

27 JH-S-3 100.0% 100.0%  **VPHGAGGIVLDVKVFNREEGDDTLSPGVNQLVRVYIVQKRKIHVGDKMCGRHGNKGVISKIVPEEDMPYLPDGRPIDIML**

28 JH-S-1 100.0% 100.0%  **VPHGAGGIVLDVKVFNREEGDDTLSPGVNQLVRVYIVQKRKIHVGDKMCGRHGNKGVISKIVPEEDMPYLPDGRPIDIML**

29 s2 100.0% 99.9%  **VPHGAGGIVLDVKVFNREEGDDTLSPGVNQLVRVYIVQKRKIHVGDKMCGRHGNKGVISKIVPEEDMPYLPDGRPIDIML**

30 S43 100.0% 100.0%  **VPHGAGGIVLDVKVFNREEGDDTLSPGVNQLVRVYIVQKRKIHVGDKMCGRHGNKGVISKIVPEEDMPYLPDGRPIDIML**

31 S17W 100.0% 100.0%  **VPHGAGGIVLDVKVFNREEGDDTLSPGVNQLVRVYIVQKRKIHVGDKMCGRHGNKGVISKIVPEEDMPYLPDGRPIDIML**

32 48 100.0% 99.9%  **VPHGAGGIVLDVKVFNREEGDDTLSPGVNQLVRVYIVQKRKIHVGDKMCGRHGNKGVISKIVPEEDMPYLPDGRPIDIML**

33 ABKVF 100.0% 99.9%  **VPHGAGGIVLDVKVFNREEGDDTLSPGVNQLVRVYIVQKRKIHVGDKMCGRHGNKGVISKIVPEEDMPYLPDGRPIDIML**

34 CICARIA 100.0% 100.0%  **VPHGAGGIVLDVKVFNREEGDDTLSPGVNQLVRVYIVQKRKIHVGDKMCGRHGNKGVISKIVPEEDMPYLPDGRPIDIML**

35 SKN25lux 100.0% 100.0%  **VPHGAGGIVLDVKVFNREEGDDTLSPGVNQLVRVYIVQKRKIHVGDKMCGRHGNKGVISKIVPEEDMPYLPDGRPIDIML**

36 HMSC074F11 100.0% 100.0%  **VPHGAGGIVLDVKVFNREEGDDTLSPGVNQLVRVYIVQKRKIHVGDKMCGRHGNKGVISKIVPEEDMPYLPDGRPIDIML**

37 HMSC068G11 100.0% 100.0%  **VPHGAGGIVLDVKVFNREEGDDTLSPGVNQLVRVYIVQKRKIHVGDKMCGRHGNKGVISKIVPEEDMPYLPDGRPIDIML**

38 NIHLM037 100.0% 99.9%  **VPHGAGGIVLDVKVFNREEGDDTLSPGVNQLVRVYIVQKRKIHVGDKMCGRHGNKGVISKIVPEEDMPYLPDGRPIDIML**

39 NIHLM015 100.0% 100.0%  **VPHGAGGIVLDVKVFNREEGDDTLSPGVNQLVRVYIVQKRKIHVGDKMCGRHGNKGVISKIVPEEDMPYLPDGRPIDIML**

consensus/100%  **VPHGAGGIVLDVKVFNREEGDDTLSPGVNQLVRVYIVQKRKIHVGDKMCGRHGNKGVISKIVPEEDMPYLPDGRPIDIhL**

consensus/90%  **VPHGAGGIVLDVKVFNREEGDDTLSPGVNQLVRVYIVQKRKIHVGDKMCGRHGNKGVISKIVPEEDMPYLPDGRPIDIML**

consensus/80%  **VPHGAGGIVLDVKVFNREEGDDTLSPGVNQLVRVYIVQKRKIHVGDKMCGRHGNKGVISKIVPEEDMPYLPDGRPIDIML**

consensus/70%  **VPHGAGGIVLDVKVFNREEGDDTLSPGVNQLVRVYIVQKRKIHVGDKMCGRHGNKGVISKIVPEEDMPYLPDGRPIDIML**

cov pid  **961**  **. . . 0 . . . .** **1040**

1 ATCC35984 100.0% 100.0%  **NPLGVPSRMNIGQVLELHLGMAAKNLGIHVASPVFDGANDDDVWSTIEEAGMARDGKTVLYDGRTGEPFDNRISVGVMYM**

2 ATCC12228 100.0% 100.0%  **NPLGVPSRMNIGQVLELHLGMAAKNLGIHVASPVFDGANDDDVWSTIEEAGMARDGKTVLYDGRTGEPFDNRISVGVMYM**

3 36AM 94.9% 100.0%  **NPLGVPSRMNIGQVLELHLGMAAKNLGIHVASPVFDGANDDDVWSTIEEAGMARDGKTVLYDGRTGEPFDNRISVGVMYM**

4 48AF 100.0% 100.0%  **NPLGVPSRMNIGQVLELHLGMAAKNLGIHVASPVFDGANDDDVWSTIEEAGMARDGKTVLYDGRTGEPFDNRISVGVMYM**

5 54AF 100.0% 99.9%  **NPLGVPSRMNIGQVLELHLGMAAKNLGIHVASPVFDGANDDDVWSTIEEAGMARDGKTVLYDGRTGEPFDNRISVGVMYM**

6 785_SEPI 100.0% 99.9%  **NPLGVPSRMNIGQVLELHLGMAAKNLGIHVASPVFDGANDDDVWSTIEEAGMARDGKTVLYDGRTGEPFDNRISVGVMYM**

7 VSE49 100.0% 100.0%  **NPLGVPSRMNIGQVLELHLGMAAKNLGIHVASPVFDGANDDDVWSTIEEAGMARDGKTVLYDGRTGEPFDNRISVGVMYM**

8 VSE57 100.0% 100.0%  **NPLGVPSRMNIGQVLELHLGMAAKNLGIHVASPVFDGANDDDVWSTIEEAGMARDGKTVLYDGRTGEPFDNRISVGVMYM**

9 SE45 100.0% 100.0%  **NPLGVPSRMNIGQVLELHLGMAAKNLGIHVASPVFDGANDDDVWSTIEEAGMARDGKTVLYDGRTGEPFDNRISVGVMYM**

10 SE40 100.0% 100.0%  **NPLGVPSRMNIGQVLELHLGMAAKNLGIHVASPVFDGANDDDVWSTIEEAGMARDGKTVLYDGRTGEPFDNRISVGVMYM**

11 32A 100.0% 99.2%  **NPLGVPSRMNIGQVLELHLGMAAKNLGIHVASPVFDGANDDDVWSTIEEAGMARDGKTVLYDGRTGEPFDNRISVGVMYM**

12 HD66 100.0% 100.0%  **NPLGVPSRMNIGQVLELHLGMAAKNLGIHVASPVFDGANDDDVWSTIEEAGMARDGKTVLYDGRTGEPFDNRISVGVMYM**

13 HD43 100.0% 99.8%  **NPLGVPSRMNIGQVLELHLGMAAKNLGIHVASPVFDGANDDDVWSTIEEAGMARDGKTVLYDGRTGEPFDNRISVGVMYM**

14 HD33 100.0% 99.9%  **NPLGVPSRMNIGQVLELHLGMAAKNLGIHVASPVFDGANDDDVWSTIEEAGMARDGKTVLYDGRTGEPFDNRISVGVMYM**

15 DE0525 100.0% 100.0%  **NPLGVPSRMNIGQVLELHLGMAAKNLGIHVASPVFDGANDDDVWSTIEEAGMARDGKTVLYDGRTGEPFDNRISVGVMYM**

16 4928STDY7071543 94.9% 100.0%  **NPLGVPSRMNIGQVLELHLGMAAKNLGIHVASPVFDGANDDDVWSTIEEAGMARDGKTVLYDGRTGEPFDNRISVGVMYM**

17 BAV2502 94.9% 99.9%  **NPLGVPSRMNIGQVLELHLGMAAKNLGIHVASPVFDGANDDDVWSTIEEAGMARDGKTVLYDGRTGEPFDNRISVGVMYM**

18 APC3784 100.0% 100.0%  **NPLGVPSRMNIGQVLELHLGMAAKNLGIHVASPVFDGANDDDVWSTIEEAGMARDGKTVLYDGRTGEPFDNRISVGVMYM**

19 SESURV_p1_0557 100.0% 100.0%  **NPLGVPSRMNIGQVLELHLGMAAKNLGIHVASPVFDGANDDDVWSTIEEAGMARDGKTVLYDGRTGEPFDNRISVGVMYM**

20 SESURV_p1_0612 100.0% 100.0%  **NPLGVPSRMNIGQVLELHLGMAAKNLGIHVASPVFDGANDDDVWSTIEEAGMARDGKTVLYDGRTGEPFDNRISVGVMYM**

21 SESURV_p4_1553 100.0% 100.0%  **NPLGVPSRMNIGQVLELHLGMAAKNLGIHVASPVFDGANDDDVWSTIEEAGMARDGKTVLYDGRTGEPFDNRISVGVMYM**

22 SESURV_p1_1200 100.0% 100.0%  **NPLGVPSRMNIGQVLELHLGMAAKNLGIHVASPVFDGANDDDVWSTIEEAGMARDGKTVLYDGRTGEPFDNRISVGVMYM**

23 SESURV_p3_0825 100.0% 100.0%  **NPLGVPSRMNIGQVLELHLGMAAKNLGIHVASPVFDGANDDDVWSTIEEAGMARDGKTVLYDGRTGEPFDNRISVGVMYM**

24 JH 100.0% 100.0%  **NPLGVPSRMNIGQVLELHLGMAAKNLGIHVASPVFDGANDDDVWSTIEEAGMARDGKTVLYDGRTGEPFDNRISVGVMYM**

25 Z0118SE0132 100.0% 99.9%  **NPLGVPSRMNIGQVLELHLGMAAKNLGIHVASPVFDGANDDDVWSTIEEAGMARDGKTVLYDGRTGEPFDNRISVGVMYM**

26 R5981 100.0% 100.0%  **NPLGVPSRMNIGQVLELHLGMAAKNLGIHVASPVFDGANDDDVWSTIEEAGMARDGKTVLYDGRTGEPFDNRISVGVMYM**

27 JH-S-3 100.0% 100.0%  **NPLGVPSRMNIGQVLELHLGMAAKNLGIHVASPVFDGANDDDVWSTIEEAGMARDGKTVLYDGRTGEPFDNRISVGVMYM**

28 JH-S-1 100.0% 100.0%  **NPLGVPSRMNIGQVLELHLGMAAKNLGIHVASPVFDGANDDDVWSTIEEAGMARDGKTVLYDGRTGEPFDNRISVGVMYM**

29 s2 100.0% 99.9%  **NPLGVPSRMNIGQVLELHLGMAAKNLGIHVASPVFDGANDDDVWSTIEEAGMARDGKTVLYDGRTGEPFDNRISVGVMYM**

30 S43 100.0% 100.0%  **NPLGVPSRMNIGQVLELHLGMAAKNLGIHVASPVFDGANDDDVWSTIEEAGMARDGKTVLYDGRTGEPFDNRISVGVMYM**

31 S17W 100.0% 100.0%  **NPLGVPSRMNIGQVLELHLGMAAKNLGIHVASPVFDGANDDDVWSTIEEAGMARDGKTVLYDGRTGEPFDNRISVGVMYM**

32 48 100.0% 99.9%  **NPLGVPSRMNIGQVLELHLGMAAKNLGIHVASPVFDGANDDDVWSTIEEAGMARDGKTVLYDGRTGEPFDNRISVGVMYM**

33 ABKVF 100.0% 99.9%  **NPLGVPSRMNIGQVLELHLGMAAKNLGIHVASPVFDGANDDDVWSTIEEAGMARDGKTVLYDGRTGEPFDNRISVGVMYM**

34 CICARIA 100.0% 100.0%  **NPLGVPSRMNIGQVLELHLGMAAKNLGIHVASPVFDGANDDDVWSTIEEAGMARDGKTVLYDGRTGEPFDNRISVGVMYM**

35 SKN25lux 100.0% 100.0%  **NPLGVPSRMNIGQVLELHLGMAAKNLGIHVASPVFDGANDDDVWSTIEEAGMARDGKTVLYDGRTGEPFDNRISVGVMYM**

36 HMSC074F11 100.0% 100.0%  **NPLGVPSRMNIGQVLELHLGMAAKNLGIHVASPVFDGANDDDVWSTIEEAGMARDGKTVLYDGRTGEPFDNRISVGVMYM**

37 HMSC068G11 100.0% 100.0%  **NPLGVPSRMNIGQVLELHLGMAAKNLGIHVASPVFDGANDDDVWSTIEEAGMARDGKTVLYDGRTGEPFDNRISVGVMYM**

38 NIHLM037 100.0% 99.9%  **NPLGVPSRMNIGQVLELHLGMAAKNLGIHVASPVFDGANDDDVWSTIEEAGMARDGKTVLYDGRTGEPFDNRISVGVMYM**

39 NIHLM015 100.0% 100.0%  **NPLGVPSRMNIGQVLELHLGMAAKNLGIHVASPVFDGANDDDVWSTIEEAGMARDGKTVLYDGRTGEPFDNRISVGVMYM**

consensus/100%  **NPLGVPSRMNIGQVLELHLGMAAKNLGIHVASPVFDGANDDDVWSTIEEAGMARDGKTVLYDGRTGEPFDNRISVGVMYM**

consensus/90%  **NPLGVPSRMNIGQVLELHLGMAAKNLGIHVASPVFDGANDDDVWSTIEEAGMARDGKTVLYDGRTGEPFDNRISVGVMYM**

consensus/80%  **NPLGVPSRMNIGQVLELHLGMAAKNLGIHVASPVFDGANDDDVWSTIEEAGMARDGKTVLYDGRTGEPFDNRISVGVMYM**

consensus/70%  **NPLGVPSRMNIGQVLELHLGMAAKNLGIHVASPVFDGANDDDVWSTIEEAGMARDGKTVLYDGRTGEPFDNRISVGVMYM**

cov pid **1041**  **: . . . . 1 . .** **1120**

1 ATCC35984 100.0% 100.0%  **LKLAHMVDDKLHARSTGPYSLVTQQPLGGKAQFGGQRFGEMEVWALEAYGAAYTLQEILTYKSDDTVGRVKTYESIVKGE**

2 ATCC12228 100.0% 100.0%  **LKLAHMVDDKLHARSTGPYSLVTQQPLGGKAQFGGQRFGEMEVWALEAYGAAYTLQEILTYKSDDTVGRVKTYESIVKGE**

3 36AM 94.9% 100.0%  **LKLAHMVDDKLHARSTGPYSLVTQQPLGGKAQFGGQRFGEMEVWALEAYGAAYTLQEILTYKSDDTVGRVKTYESIVKGE**

4 48AF 100.0% 100.0%  **LKLAHMVDDKLHARSTGPYSLVTQQPLGGKAQFGGQRFGEMEVWALEAYGAAYTLQEILTYKSDDTVGRVKTYESIVKGE**

5 54AF 100.0% 99.9%  **LKLAHMVDDKLHARSTGPYSLVTQQPLGGKAQFGGQRFGEMEVWALEAYGAAYTLQEILTYKSDDTVGRVKTYESIVKGE**

6 785_SEPI 100.0% 99.9%  **LKLAHMVDDKLHARSTGPYSLVTQQPLGGKAQFGGQRFGEMEVWALEAYGAAYTLQEILTYKSDDTVGRVKTYESIVKGE**

7 VSE49 100.0% 100.0%  **LKLAHMVDDKLHARSTGPYSLVTQQPLGGKAQFGGQRFGEMEVWALEAYGAAYTLQEILTYKSDDTVGRVKTYESIVKGE**

8 VSE57 100.0% 100.0%  **LKLAHMVDDKLHARSTGPYSLVTQQPLGGKAQFGGQRFGEMEVWALEAYGAAYTLQEILTYKSDDTVGRVKTYESIVKGE**

9 SE45 100.0% 100.0%  **LKLAHMVDDKLHARSTGPYSLVTQQPLGGKAQFGGQRFGEMEVWALEAYGAAYTLQEILTYKSDDTVGRVKTYESIVKGE**

10 SE40 100.0% 100.0%  **LKLAHMVDDKLHARSTGPYSLVTQQPLGGKAQFGGQRFGEMEVWALEAYGAAYTLQEILTYKSDDTVGRVKTYESIVKGE**

11 32A 100.0% 99.2%  **LKLAHMVDDKLHARSTGPYSLVTQQPLGGKAQFGGQRFGEMEVWALEAYGAAYTLQEILTYKSDDTVGRVKTYESIVKGE**

12 HD66 100.0% 100.0%  **LKLAHMVDDKLHARSTGPYSLVTQQPLGGKAQFGGQRFGEMEVWALEAYGAAYTLQEILTYKSDDTVGRVKTYESIVKGE**

13 HD43 100.0% 99.8%  **LKLAHMVDDKLHARSTGPYSLVTQQPLGGKAQFGGQRFGEMEVWALEAYGAAYTLQEILTYKSDDTVGRVKTYESIVKGE**

14 HD33 100.0% 99.9%  **LKLAHMVDDKLHARSTGPYSLVTQQPLGGKAQFGGQRFGEMEVWALEAYGAAYTLQEILTYKSDDTVGRVKTYESIVKGE**

15 DE0525 100.0% 100.0%  **LKLAHMVDDKLHARSTGPYSLVTQQPLGGKAQFGGQRFGEMEVWALEAYGAAYTLQEILTYKSDDTVGRVKTYESIVKGE**

16 4928STDY7071543 94.9% 100.0%  **LKLAHMVDDKLHARSTGPYSLVTQQPLGGKAQFGGQRFGEMEVWALEAYGAAYTLQEILTYKSDDTVGRVKTYESIVKGE**

17 BAV2502 94.9% 99.9%  **LKLAHMVDDKLHARSTGPYSLVTQQPLGGKAQFGGQRFGEMEVWALEAYGAAYTLQEILTYKSDDTVGRVKTYESIVKGE**

18 APC3784 100.0% 100.0%  **LKLAHMVDDKLHARSTGPYSLVTQQPLGGKAQFGGQRFGEMEVWALEAYGAAYTLQEILTYKSDDTVGRVKTYESIVKGE**

19 SESURV_p1_0557 100.0% 100.0%  **LKLAHMVDDKLHARSTGPYSLVTQQPLGGKAQFGGQRFGEMEVWALEAYGAAYTLQEILTYKSDDTVGRVKTYESIVKGE**

20 SESURV_p1_0612 100.0% 100.0%  **LKLAHMVDDKLHARSTGPYSLVTQQPLGGKAQFGGQRFGEMEVWALEAYGAAYTLQEILTYKSDDTVGRVKTYESIVKGE**

21 SESURV_p4_1553 100.0% 100.0%  **LKLAHMVDDKLHARSTGPYSLVTQQPLGGKAQFGGQRFGEMEVWALEAYGAAYTLQEILTYKSDDTVGRVKTYESIVKGE**

22 SESURV_p1_1200 100.0% 100.0%  **LKLAHMVDDKLHARSTGPYSLVTQQPLGGKAQFGGQRFGEMEVWALEAYGAAYTLQEILTYKSDDTVGRVKTYESIVKGE**

23 SESURV_p3_0825 100.0% 100.0%  **LKLAHMVDDKLHARSTGPYSLVTQQPLGGKAQFGGQRFGEMEVWALEAYGAAYTLQEILTYKSDDTVGRVKTYESIVKGE**

24 JH 100.0% 100.0%  **LKLAHMVDDKLHARSTGPYSLVTQQPLGGKAQFGGQRFGEMEVWALEAYGAAYTLQEILTYKSDDTVGRVKTYESIVKGE**

25 Z0118SE0132 100.0% 99.9%  **LKLAHMVDDKLHARSTGPYSLVTQQPLGGKAQFGGQRFGEMEVWALEAYGAAYTLQEILTYKSDDTVGRVKTYESIVKGE**

26 R5981 100.0% 100.0%  **LKLAHMVDDKLHARSTGPYSLVTQQPLGGKAQFGGQRFGEMEVWALEAYGAAYTLQEILTYKSDDTVGRVKTYESIVKGE**

27 JH-S-3 100.0% 100.0%  **LKLAHMVDDKLHARSTGPYSLVTQQPLGGKAQFGGQRFGEMEVWALEAYGAAYTLQEILTYKSDDTVGRVKTYESIVKGE**

28 JH-S-1 100.0% 100.0%  **LKLAHMVDDKLHARSTGPYSLVTQQPLGGKAQFGGQRFGEMEVWALEAYGAAYTLQEILTYKSDDTVGRVKTYESIVKGE**

29 s2 100.0% 99.9%  **LKLAHMVDDKLHARSTGPYSLVTQQPLGGKAQFGGQRFGEMEVWALEAYGAAYTLQEILTYKSDDTVGRVKTYESIVKGE**

30 S43 100.0% 100.0%  **LKLAHMVDDKLHARSTGPYSLVTQQPLGGKAQFGGQRFGEMEVWALEAYGAAYTLQEILTYKSDDTVGRVKTYESIVKGE**

31 S17W 100.0% 100.0%  **LKLAHMVDDKLHARSTGPYSLVTQQPLGGKAQFGGQRFGEMEVWALEAYGAAYTLQEILTYKSDDTVGRVKTYESIVKGE**

32 48 100.0% 99.9%  **LKLAHMVDDKLHARSTGPYSLVTQQPLGGKAQFGGQRFGEMEVWALEAYGAAYTLQEILTYKSDDTVGRVKTYESIVKGE**

33 ABKVF 100.0% 99.9%  **LKLAHMVDDKLHARSTGPYSLVTQQPLGGKAQFGGQRFGEMEVWALEAYGAAYTLQEILTYKSDDTVGRVKTYESIVKGE**

34 CICARIA 100.0% 100.0%  **LKLAHMVDDKLHARSTGPYSLVTQQPLGGKAQFGGQRFGEMEVWALEAYGAAYTLQEILTYKSDDTVGRVKTYESIVKGE**

35 SKN25lux 100.0% 100.0%  **LKLAHMVDDKLHARSTGPYSLVTQQPLGGKAQFGGQRFGEMEVWALEAYGAAYTLQEILTYKSDDTVGRVKTYESIVKGE**

36 HMSC074F11 100.0% 100.0%  **LKLAHMVDDKLHARSTGPYSLVTQQPLGGKAQFGGQRFGEMEVWALEAYGAAYTLQEILTYKSDDTVGRVKTYESIVKGE**

37 HMSC068G11 100.0% 100.0%  **LKLAHMVDDKLHARSTGPYSLVTQQPLGGKAQFGGQRFGEMEVWALEAYGAAYTLQEILTYKSDDTVGRVKTYESIVKGE**

38 NIHLM037 100.0% 99.9%  **LKLAHMVDDKLHARSTGPYSLVTQQPLGGKAQFGGQRFGEMEVWALEAYGAAYTLQEILTYKSDDTVGRVKTYESIVKGE**

39 NIHLM015 100.0% 100.0%  **LKLAHMVDDKLHARSTGPYSLVTQQPLGGKAQFGGQRFGEMEVWALEAYGAAYTLQEILTYKSDDTVGRVKTYESIVKGE**

consensus/100%  **LKLAHMVDDKLHARSTGPYSLVTQQPLGGKAQFGGQRFGEMEVWALEAYGAAYTLQEILTYKSDDTVGRVKTYESIVKGE**

consensus/90%  **LKLAHMVDDKLHARSTGPYSLVTQQPLGGKAQFGGQRFGEMEVWALEAYGAAYTLQEILTYKSDDTVGRVKTYESIVKGE**

consensus/80%  **LKLAHMVDDKLHARSTGPYSLVTQQPLGGKAQFGGQRFGEMEVWALEAYGAAYTLQEILTYKSDDTVGRVKTYESIVKGE**

consensus/70%  **LKLAHMVDDKLHARSTGPYSLVTQQPLGGKAQFGGQRFGEMEVWALEAYGAAYTLQEILTYKSDDTVGRVKTYESIVKGE**

cov pid **1121**  **. . : . . . ]** **1186**

1 ATCC35984 100.0% 100.0%  **NISRPSVPESFRVLMKELQSLGLDVKVMDEHDNEIEMADVDDEDAAERKVDLQQKSAPESQKETTD**

2 ATCC12228 100.0% 100.0%  **NISRPSVPESFRVLMKELQSLGLDVKVMDEHDNEIEMADVDDEDAAERKVDLQQKSAPESQKETTD**

3 36AM 94.9% 100.0%  **NISRPSVPESFRVLMKELQSLGLDVKVMDEHDNEIEMADVDDEDAAERKVDLQQKSAPESQKETTD**

4 48AF 100.0% 100.0%  **NISRPSVPESFRVLMKELQSLGLDVKVMDEHDNEIEMADVDDEDAAERKVDLQQKSAPESQKETTD**

5 54AF 100.0% 99.9%  **NISRPSVPESFRVLMKELQSLGLDVKVMDEHDNEIEMADVDDEDAAERKVDLQQKSAPESQKETTD**

6 785_SEPI 100.0% 99.9%  **NISRPSVPESFRVLMKELQSLGLDVKVMDEHDNEIEMADVDDEDAAERKVDLQQKSAPESQKETTD**

7 VSE49 100.0% 100.0%  **NISRPSVPESFRVLMKELQSLGLDVKVMDEHDNEIEMADVDDEDAAERKVDLQQKSAPESQKETTD**

8 VSE57 100.0% 100.0%  **NISRPSVPESFRVLMKELQSLGLDVKVMDEHDNEIEMADVDDEDAAERKVDLQQKSAPESQKETTD**

9 SE45 100.0% 100.0%  **NISRPSVPESFRVLMKELQSLGLDVKVMDEHDNEIEMADVDDEDAAERKVDLQQKSAPESQKETTD**

10 SE40 100.0% 100.0%  **NISRPSVPESFRVLMKELQSLGLDVKVMDEHDNEIEMADVDDEDAAERKVDLQQKSAPESQKETTD**

11 32A 100.0% 99.2%  **NISRPSVPESFRVLMKELQSLGLDVKVMDEHDNEIEMADVDDEDAAERKVDLQQKSAPESQKETTD**

12 HD66 100.0% 100.0%  **NISRPSVPESFRVLMKELQSLGLDVKVMDEHDNEIEMADVDDEDAAERKVDLQQKSAPESQKETTD**

13 HD43 100.0% 99.8%  **NISRPSVPESFRVLMKELQSLGLDVKVMDEHDNEIEMADVDDEDAAERKVDLQQKSAPESQKETTD**

14 HD33 100.0% 99.9%  **NISRPSVPESFRVLMKELQSLGLDVKVMDEHDNEIEMADVDDEDAAERKVDLQQKSAPESQKETTD**

15 DE0525 100.0% 100.0%  **NISRPSVPESFRVLMKELQSLGLDVKVMDEHDNEIEMADVDDEDAAERKVDLQQKSAPESQKETTD**

16 4928STDY7071543 94.9% 100.0%  **NISRPSVPESFRVLMKELQSLGLDVKVMDEHDNEIEMADVDDEDAAERKVDLQQKSAPESQKETTD**

17 BAV2502 94.9% 99.9%  **NISRPSVPESFRVLMKELQSLGLDVKVMDEHDNEIEMADVDDEDAAERKVDLQQKSAPESQKETTD**

18 APC3784 100.0% 100.0%  **NISRPSVPESFRVLMKELQSLGLDVKVMDEHDNEIEMADVDDEDAAERKVDLQQKSAPESQKETTD**

19 SESURV_p1_0557 100.0% 100.0%  **NISRPSVPESFRVLMKELQSLGLDVKVMDEHDNEIEMADVDDEDAAERKVDLQQKSAPESQKETTD**

20 SESURV_p1_0612 100.0% 100.0%  **NISRPSVPESFRVLMKELQSLGLDVKVMDEHDNEIEMADVDDEDAAERKVDLQQKSAPESQKETTD**

21 SESURV_p4_1553 100.0% 100.0%  **NISRPSVPESFRVLMKELQSLGLDVKVMDEHDNEIEMADVDDEDAAERKVDLQQKSAPESQKETTD**

22 SESURV_p1_1200 100.0% 100.0%  **NISRPSVPESFRVLMKELQSLGLDVKVMDEHDNEIEMADVDDEDAAERKVDLQQKSAPESQKETTD**

23 SESURV_p3_0825 100.0% 100.0%  **NISRPSVPESFRVLMKELQSLGLDVKVMDEHDNEIEMADVDDEDAAERKVDLQQKSAPESQKETTD**

24 JH 100.0% 100.0%  **NISRPSVPESFRVLMKELQSLGLDVKVMDEHDNEIEMADVDDEDAAERKVDLQQKSAPESQKETTD**

25 Z0118SE0132 100.0% 99.9%  **NISRPSVPESFRVLMKELQSLGLDVKVMDEHDNEIEMADVDDEDAAERKVDLQQKSAPESQKETTD**

26 R5981 100.0% 100.0%  **NISRPSVPESFRVLMKELQSLGLDVKVMDEHDNEIEMADVDDEDAAERKVDLQQKSAPESQKETTD**

27 JH-S-3 100.0% 100.0%  **NISRPSVPESFRVLMKELQSLGLDVKVMDEHDNEIEMADVDDEDAAERKVDLQQKSAPESQKETTD**

28 JH-S-1 100.0% 100.0%  **NISRPSVPESFRVLMKELQSLGLDVKVMDEHDNEIEMADVDDEDAAERKVDLQQKSAPESQKETTD**

29 s2 100.0% 99.9%  **NISRPSVPESFRVLMKELQSLGLDVKVMDEHDNEIEMADVDDEDAAERKVDLQQKSAPESQKETTD**

30 S43 100.0% 100.0%  **NISRPSVPESFRVLMKELQSLGLDVKVMDEHDNEIEMADVDDEDAAERKVDLQQKSAPESQKETTD**

31 S17W 100.0% 100.0%  **NISRPSVPESFRVLMKELQSLGLDVKVMDEHDNEIEMADVDDEDAAERKVDLQQKSAPESQKETTD**

32 48 100.0% 99.9%  **NISRPSVPESFRVLMKELQSLGLDVKVMDEHDNEIEMADVDDEDAAERKVDLQQKSAPESQKETTD**

33 ABKVF 100.0% 99.9%  **NISRPSVPESFRVLMKELQSLGLDVKVMDEHDNEIEMADVDDEDAAERKVDLQQKSAPESQKETTD**

34 CICARIA 100.0% 100.0%  **NISRPSVPESFRVLMKELQSLGLDVKVMDEHDNEIEMADVDDEDAAERKVDLQQKSAPESQKETTD**

35 SKN25lux 100.0% 100.0%  **NISRPSVPESFRVLMKELQSLGLDVKVMDEHDNEIEMADVDDEDAAERKVDLQQKSAPESQKETTD**

36 HMSC074F11 100.0% 100.0%  **NISRPSVPESFRVLMKELQSLGLDVKVMDEHDNEIEMADVDDEDAAERKVDLQQKSAPESQKETTD**

37 HMSC068G11 100.0% 100.0%  **NISRPSVPESFRVLMKELQSLGLDVKVMDEHDNEIEMADVDDEDAAERKVDLQQKSAPESQKETTD**

38 NIHLM037 100.0% 99.9%  **NISRPSVPESFRVLMKELQSLGLDVKVMDEHDNEIEMADVDDEDAAERKVDLQQKSAPESQKETTD**

39 NIHLM015 100.0% 100.0%  **NISRPSVPESFRVLMKELQSLGLDVKVMDEHDNEIEMADVDDEDAAERKVDLQQKSAPESQKETTD**

consensus/100%  **NISRPSVPESFRVLMKELQSLGLDVKVMDEHDNEIEMADVDDEDAAERKVDLQQKSAPESQKETTD**

consensus/90%  **NISRPSVPESFRVLMKELQSLGLDVKVMDEHDNEIEMADVDDEDAAERKVDLQQKSAPESQKETTD**

consensus/80%  **NISRPSVPESFRVLMKELQSLGLDVKVMDEHDNEIEMADVDDEDAAERKVDLQQKSAPESQKETTD**

consensus/70%  **NISRPSVPESFRVLMKELQSLGLDVKVMDEHDNEIEMADVDDEDAAERKVDLQQKSAPESQKETTD**

**Supplementary Figure 5.** Multiple sequence alignment of the predicted amino acid sequences of RpoB carried by *S. epidermidis* isolates and close genomes retrieved from the BV-BRC database compared to that of *S. epidermidis* strain ATCC®12228 and ATCC®35984. The alignment was visualized using MView version 1.63 hosted by the EMBL-EBI; cov, coverage; pid, percent identity.

cov pid  **1** **[ . . . . : . . .** **80**

1 29AM 100.0% 100.0%  **MAELPQSRINERNITNEMRESFLDYAMSVIVSRALPDVRDGLKPVHRRILYGLNEQGMTPDKPYKKSARIVGDVMGKYHP**

2 TRPF4 100.0% 100.0%  **MAELPQSRINERNITNEMRESFLDYAMSVIVSRALPDVRDGLKPVHRRILYGLNEQGMTPDKPYKKSARIVGDVMGKYHP**

3 P912 100.0% 100.0%  **MAELPQSRINERNITNEMRESFLDYAMSVIVSRALPDVRDGLKPVHRRILYGLNEQGMTPDKPYKKSARIVGDVMGKYHP**

4 NGS-ED-1001 93.3% 99.9%  **------------------------------------------------------------DKPYKKSARIVGDVMGKYHP**

5 1DB1 100.0% 100.0%  **MAELPQSRINERNITNEMRESFLDYAMSVIVSRALPDVRDGLKPVHRRILYGLNEQGMTPDKPYKKSARIVGDVMGKYHP**

6 MGYG-HGUT-02301 100.0% 100.0%  **MAELPQSRINERNITNEMRESFLDYAMSVIVSRALPDVRDGLKPVHRRILYGLNEQGMTPDKPYKKSARIVGDVMGKYHP**

7 IIF4SW-P1 100.0% 100.0%  **MAELPQSRINERNITNEMRESFLDYAMSVIVSRALPDVRDGLKPVHRRILYGLNEQGMTPDKPYKKSARIVGDVMGKYHP**

8 19428wF1_P912 100.0% 100.0%  **MAELPQSRINERNITNEMRESFLDYAMSVIVSRALPDVRDGLKPVHRRILYGLNEQGMTPDKPYKKSARIVGDVMGKYHP**

9 R5992 100.0% 100.0%  **MAELPQSRINERNITNEMRESFLDYAMSVIVSRALPDVRDGLKPVHRRILYGLNEQGMTPDKPYKKSARIVGDVMGKYHP**

10 R5990 100.0% 100.0%  **MAELPQSRINERNITNEMRESFLDYAMSVIVSRALPDVRDGLKPVHRRILYGLNEQGMTPDKPYKKSARIVGDVMGKYHP**

11 R5991 100.0% 100.0%  **MAELPQSRINERNITNEMRESFLDYAMSVIVSRALPDVRDGLKPVHRRILYGLNEQGMTPDKPYKKSARIVGDVMGKYHP**

12 R6028 100.0% 100.0%  **MAELPQSRINERNITNEMRESFLDYAMSVIVSRALPDVRDGLKPVHRRILYGLNEQGMTPDKPYKKSARIVGDVMGKYHP**

13 R6027 100.0% 100.0%  **MAELPQSRINERNITNEMRESFLDYAMSVIVSRALPDVRDGLKPVHRRILYGLNEQGMTPDKPYKKSARIVGDVMGKYHP**

14 R6024 100.0% 100.0%  **MAELPQSRINERNITNEMRESFLDYAMSVIVSRALPDVRDGLKPVHRRILYGLNEQGMTPDKPYKKSARIVGDVMGKYHP**

15 L2_057 100.0% 100.0%  **MAELPQSRINERNITNEMRESFLDYAMSVIVSRALPDVRDGLKPVHRRILYGLNEQGMTPDKPYKKSARIVGDVMGKYHP**

16 Cap 100.0% 99.9%  **MAELPQSRINERNITNEMRESFLDYAMSVIVSRALPDVRDGLKPVHRRILYGLNEQGMTPDKPYKKSARIVGDVMGKYHP**

17 Ani-LG-057 100.0% 100.0%  **MAELPQSRINERNITNEMRESFLDYAMSVIVSRALPDVRDGLKPVHRRILYGLNEQGMTPDKPYKKSARIVGDVMGKYHP**

18 Cap9.2 100.0% 99.9%  **MAELPQSRINERNITNEMRESFLDYAMSVIVSRALPDVRDGLKPVHRRILYGLNEQGMTPDKPYKKSARIVGDVMGKYHP**

19 Cap10.1 100.0% 99.9%  **MAELPQSRINERNITNEMRESFLDYAMSVIVSRALPDVRDGLKPVHRRILYGLNEQGMTPDKPYKKSARIVGDVMGKYHP**

20 Cap9.1 100.0% 99.9%  **MAELPQSRINERNITNEMRESFLDYAMSVIVSRALPDVRDGLKPVHRRILYGLNEQGMTPDKPYKKSARIVGDVMGKYHP**

21 Cap10.2 100.0% 99.9%  **MAELPQSRINERNITNEMRESFLDYAMSVIVSRALPDVRDGLKPVHRRILYGLNEQGMTPDKPYKKSARIVGDVMGKYHP**

22 acrok 100.0% 99.9%  **MAELPQSRINERNITNEMRESFLDYAMSVIVSRALPDVRDGLKPVHRRILYGLNEQGMTPDKPYKKSARIVGDVMGKYHP**

23 acroj 100.0% 99.9%  **MAELPQSRINERNITNEMRESFLDYAMSVIVSRALPDVRDGLKPVHRRILYGLNEQGMTPDKPYKKSARIVGDVMGKYHP**

24 acror 100.0% 100.0%  **MAELPQSRINERNITNEMRESFLDYAMSVIVSRALPDVRDGLKPVHRRILYGLNEQGMTPDKPYKKSARIVGDVMGKYHP**

25 NJ6 100.0% 100.0%  **MAELPQSRINERNITNEMRESFLDYAMSVIVSRALPDVRDGLKPVHRRILYGLNEQGMTPDKPYKKSARIVGDVMGKYHP**

26 EYE_450 100.0% 100.0%  **MAELPQSRINERNITNEMRESFLDYAMSVIVSRALPDVRDGLKPVHRRILYGLNEQGMTPDKPYKKSARIVGDVMGKYHP**

27 EYE_117 100.0% 100.0%  **MAELPQSRINERNITNEMRESFLDYAMSVIVSRALPDVRDGLKPVHRRILYGLNEQGMTPDKPYKKSARIVGDVMGKYHP**

28 EYE_410 100.0% 100.0%  **MAELPQSRINERNITNEMRESFLDYAMSVIVSRALPDVRDGLKPVHRRILYGLNEQGMTPDKPYKKSARIVGDVMGKYHP**

29 EYE_411 100.0% 100.0%  **MAELPQSRINERNITNEMRESFLDYAMSVIVSRALPDVRDGLKPVHRRILYGLNEQGMTPDKPYKKSARIVGDVMGKYHP**

30 MERTA18 100.0% 100.0%  **MAELPQSRINERNITNEMRESFLDYAMSVIVSRALPDVRDGLKPVHRRILYGLNEQGMTPDKPYKKSARIVGDVMGKYHP**

31 FAIRING19B-1.2 100.0% 100.0%  **MAELPQSRINERNITNEMRESFLDYAMSVIVSRALPDVRDGLKPVHRRILYGLNEQGMTPDKPYKKSARIVGDVMGKYHP**

32 FDAARGOS_151 100.0% 100.0%  **MAELPQSRINERNITNEMRESFLDYAMSVIVSRALPDVRDGLKPVHRRILYGLNEQGMTPDKPYKKSARIVGDVMGKYHP**

33 SNUC5989 100.0% 100.0%  **MAELPQSRINERNITNEMRESFLDYAMSVIVSRALPDVRDGLKPVHRRILYGLNEQGMTPDKPYKKSARIVGDVMGKYHP**

34 SNUC3412 100.0% 100.0%  **MAELPQSRINERNITNEMRESFLDYAMSVIVSRALPDVRDGLKPVHRRILYGLNEQGMTPDKPYKKSARIVGDVMGKYHP**

35 OM08-17AT 100.0% 100.0%  **MAELPQSRINERNITNEMRESFLDYAMSVIVSRALPDVRDGLKPVHRRILYGLNEQGMTPDKPYKKSARIVGDVMGKYHP**

36 SWO 100.0% 100.0%  **MAELPQSRINERNITNEMRESFLDYAMSVIVSRALPDVRDGLKPVHRRILYGLNEQGMTPDKPYKKSARIVGDVMGKYHP**

37 VCU121 93.3% 100.0%  **------------------------------------------------------------DKPYKKSARIVGDVMGKYHP**

consensus/100%  **............................................................DKPYKKSARIVGDVMGKYHP**

consensus/90%  **MAELPQSRINERNITNEMRESFLDYAMSVIVSRALPDVRDGLKPVHRRILYGLNEQGMTPDKPYKKSARIVGDVMGKYHP**

consensus/80%  **MAELPQSRINERNITNEMRESFLDYAMSVIVSRALPDVRDGLKPVHRRILYGLNEQGMTPDKPYKKSARIVGDVMGKYHP**

consensus/70%  **MAELPQSRINERNITNEMRESFLDYAMSVIVSRALPDVRDGLKPVHRRILYGLNEQGMTPDKPYKKSARIVGDVMGKYHP**

cov pid  **81**  **. 1 . . . . : .** **160**

1 29AM 100.0% 100.0%  **HGDSSIYEAMVRMAQEFSYRYPLVDGQGNFGSMDGDGAAAMRYTEARMTKITLELLRDINKDTIDFIDNYDGNEREPSVL**

2 TRPF4 100.0% 100.0%  **HGDSSIYEAMVRMAQEFSYRYPLVDGQGNFGSMDGDGAAAMRYTEARMTKITLELLRDINKDTIDFIDNYDGNEREPSVL**

3 P912 100.0% 100.0%  **HGDSSIYEAMVRMAQEFSYRYPLVDGQGNFGSMDGDGAAAMRYTEARMTKITLELLRDINKDTIDFIDNYDGNEREPSVL**

4 NGS-ED-1001 93.3% 99.9%  **HGDSSIYEAMVRMAQEFSYRYPLVDGQGNFGSMDGDGAAAMRYTEARMTKITLELLRDINKDTIDFIDNYDGNEREPSVL**

5 1DB1 100.0% 100.0%  **HGDSSIYEAMVRMAQEFSYRYPLVDGQGNFGSMDGDGAAAMRYTEARMTKITLELLRDINKDTIDFIDNYDGNEREPSVL**

6 MGYG-HGUT-02301 100.0% 100.0%  **HGDSSIYEAMVRMAQEFSYRYPLVDGQGNFGSMDGDGAAAMRYTEARMTKITLELLRDINKDTIDFIDNYDGNEREPSVL**

7 IIF4SW-P1 100.0% 100.0%  **HGDSSIYEAMVRMAQEFSYRYPLVDGQGNFGSMDGDGAAAMRYTEARMTKITLELLRDINKDTIDFIDNYDGNEREPSVL**

8 19428wF1_P912 100.0% 100.0%  **HGDSSIYEAMVRMAQEFSYRYPLVDGQGNFGSMDGDGAAAMRYTEARMTKITLELLRDINKDTIDFIDNYDGNEREPSVL**

9 R5992 100.0% 100.0%  **HGDSSIYEAMVRMAQEFSYRYPLVDGQGNFGSMDGDGAAAMRYTEARMTKITLELLRDINKDTIDFIDNYDGNEREPSVL**

10 R5990 100.0% 100.0%  **HGDSSIYEAMVRMAQEFSYRYPLVDGQGNFGSMDGDGAAAMRYTEARMTKITLELLRDINKDTIDFIDNYDGNEREPSVL**

11 R5991 100.0% 100.0%  **HGDSSIYEAMVRMAQEFSYRYPLVDGQGNFGSMDGDGAAAMRYTEARMTKITLELLRDINKDTIDFIDNYDGNEREPSVL**

12 R6028 100.0% 100.0%  **HGDSSIYEAMVRMAQEFSYRYPLVDGQGNFGSMDGDGAAAMRYTEARMTKITLELLRDINKDTIDFIDNYDGNEREPSVL**

13 R6027 100.0% 100.0%  **HGDSSIYEAMVRMAQEFSYRYPLVDGQGNFGSMDGDGAAAMRYTEARMTKITLELLRDINKDTIDFIDNYDGNEREPSVL**

14 R6024 100.0% 100.0%  **HGDSSIYEAMVRMAQEFSYRYPLVDGQGNFGSMDGDGAAAMRYTEARMTKITLELLRDINKDTIDFIDNYDGNEREPSVL**

15 L2_057 100.0% 100.0%  **HGDSSIYEAMVRMAQEFSYRYPLVDGQGNFGSMDGDGAAAMRYTEARMTKITLELLRDINKDTIDFIDNYDGNEREPSVL**

16 Cap 100.0% 99.9%  **HGDSSIYEAMVRMAQEFSYRYPLVDGQGNFGSMDGDGAAAMRYTEARMTKITLELLRDINKDTIDFIDNYDGNEREPSVL**

17 Ani-LG-057 100.0% 100.0%  **HGDSSIYEAMVRMAQEFSYRYPLVDGQGNFGSMDGDGAAAMRYTEARMTKITLELLRDINKDTIDFIDNYDGNEREPSVL**

18 Cap9.2 100.0% 99.9%  **HGDSSIYEAMVRMAQEFSYRYPLVDGQGNFGSMDGDGAAAMRYTEARMTKITLELLRDINKDTIDFIDNYDGNEREPSVL**

19 Cap10.1 100.0% 99.9%  **HGDSSIYEAMVRMAQEFSYRYPLVDGQGNFGSMDGDGAAAMRYTEARMTKITLELLRDINKDTIDFIDNYDGNEREPSVL**

20 Cap9.1 100.0% 99.9%  **HGDSSIYEAMVRMAQEFSYRYPLVDGQGNFGSMDGDGAAAMRYTEARMTKITLELLRDINKDTIDFIDNYDGNEREPSVL**

21 Cap10.2 100.0% 99.9%  **HGDSSIYEAMVRMAQEFSYRYPLVDGQGNFGSMDGDGAAAMRYTEARMTKITLELLRDINKDTIDFIDNYDGNEREPSVL**

22 acrok 100.0% 99.9%  **HGDSSIYEAMVRMAQEFSYRYPLVDGQGNFGSMDGDGAAAMRYTEARMTKITLELLRDINKDTIDFIDNYDGNEREPSVL**

23 acroj 100.0% 99.9%  **HGDSSIYEAMVRMAQEFSYRYPLVDGQGNFGSMDGDGAAAMRYTEARMTKITLELLRDINKDTIDFIDNYDGNEREPSVL**

24 acror 100.0% 100.0%  **HGDSSIYEAMVRMAQEFSYRYPLVDGQGNFGSMDGDGAAAMRYTEARMTKITLELLRDINKDTIDFIDNYDGNEREPSVL**

25 NJ6 100.0% 100.0%  **HGDSSIYEAMVRMAQEFSYRYPLVDGQGNFGSMDGDGAAAMRYTEARMTKITLELLRDINKDTIDFIDNYDGNEREPSVL**

26 EYE_450 100.0% 100.0%  **HGDSSIYEAMVRMAQEFSYRYPLVDGQGNFGSMDGDGAAAMRYTEARMTKITLELLRDINKDTIDFIDNYDGNEREPSVL**

27 EYE_117 100.0% 100.0%  **HGDSSIYEAMVRMAQEFSYRYPLVDGQGNFGSMDGDGAAAMRYTEARMTKITLELLRDINKDTIDFIDNYDGNEREPSVL**

28 EYE_410 100.0% 100.0%  **HGDSSIYEAMVRMAQEFSYRYPLVDGQGNFGSMDGDGAAAMRYTEARMTKITLELLRDINKDTIDFIDNYDGNEREPSVL**

29 EYE_411 100.0% 100.0%  **HGDSSIYEAMVRMAQEFSYRYPLVDGQGNFGSMDGDGAAAMRYTEARMTKITLELLRDINKDTIDFIDNYDGNEREPSVL**

30 MERTA18 100.0% 100.0%  **HGDSSIYEAMVRMAQEFSYRYPLVDGQGNFGSMDGDGAAAMRYTEARMTKITLELLRDINKDTIDFIDNYDGNEREPSVL**

31 FAIRING19B-1.2 100.0% 100.0%  **HGDSSIYEAMVRMAQEFSYRYPLVDGQGNFGSMDGDGAAAMRYTEARMTKITLELLRDINKDTIDFIDNYDGNEREPSVL**

32 FDAARGOS_151 100.0% 100.0%  **HGDSSIYEAMVRMAQEFSYRYPLVDGQGNFGSMDGDGAAAMRYTEARMTKITLELLRDINKDTIDFIDNYDGNEREPSVL**

33 SNUC5989 100.0% 100.0%  **HGDSSIYEAMVRMAQEFSYRYPLVDGQGNFGSMDGDGAAAMRYTEARMTKITLELLRDINKDTIDFIDNYDGNEREPSVL**

34 SNUC3412 100.0% 100.0%  **HGDSSIYEAMVRMAQEFSYRYPLVDGQGNFGSMDGDGAAAMRYTEARMTKITLELLRDINKDTIDFIDNYDGNEREPSVL**

35 OM08-17AT 100.0% 100.0%  **HGDSSIYEAMVRMAQEFSYRYPLVDGQGNFGSMDGDGAAAMRYTEARMTKITLELLRDINKDTIDFIDNYDGNEREPSVL**

36 SWO 100.0% 100.0%  **HGDSSIYEAMVRMAQEFSYRYPLVDGQGNFGSMDGDGAAAMRYTEARMTKITLELLRDINKDTIDFIDNYDGNEREPSVL**

37 VCU121 93.3% 100.0%  **HGDSSIYEAMVRMAQEFSYRYPLVDGQGNFGSMDGDGAAAMRYTEARMTKITLELLRDINKDTIDFIDNYDGNEREPSVL**

consensus/100%  **HGDSSIYEAMVRMAQEFSYRYPLVDGQGNFGSMDGDGAAAMRYTEARMTKITLELLRDINKDTIDFIDNYDGNEREPSVL**

consensus/90%  **HGDSSIYEAMVRMAQEFSYRYPLVDGQGNFGSMDGDGAAAMRYTEARMTKITLELLRDINKDTIDFIDNYDGNEREPSVL**

consensus/80%  **HGDSSIYEAMVRMAQEFSYRYPLVDGQGNFGSMDGDGAAAMRYTEARMTKITLELLRDINKDTIDFIDNYDGNEREPSVL**

consensus/70%  **HGDSSIYEAMVRMAQEFSYRYPLVDGQGNFGSMDGDGAAAMRYTEARMTKITLELLRDINKDTIDFIDNYDGNEREPSVL**

cov pid **161**  **. . . 2 . . . .** **240**

1 29AM 100.0% 100.0%  **PARFPNLLVNGAAGIAVGMATNIPPHNLTEVIDGVLSLSKNPDISISELMEDIQGPDFPTAGLILGKSGIRRAYETGRGS**

2 TRPF4 100.0% 100.0%  **PARFPNLLVNGAAGIAVGMATNIPPHNLTEVIDGVLSLSKNPDISISELMEDIQGPDFPTAGLILGKSGIRRAYETGRGS**

3 P912 100.0% 100.0%  **PARFPNLLVNGAAGIAVGMATNIPPHNLTEVIDGVLSLSKNPDISISELMEDIQGPDFPTAGLILGKSGIRRAYETGRGS**

4 NGS-ED-1001 93.3% 99.9%  **PARFPNLLVNGAAGIAVGMATNIPPHNLTEVIDGVLSLSKNPDISISELMEDIQGPDFPTAGLILGKSGIRRAYETGRGS**

5 1DB1 100.0% 100.0%  **PARFPNLLVNGAAGIAVGMATNIPPHNLTEVIDGVLSLSKNPDISISELMEDIQGPDFPTAGLILGKSGIRRAYETGRGS**

6 MGYG-HGUT-02301 100.0% 100.0%  **PARFPNLLVNGAAGIAVGMATNIPPHNLTEVIDGVLSLSKNPDISISELMEDIQGPDFPTAGLILGKSGIRRAYETGRGS**

7 IIF4SW-P1 100.0% 100.0%  **PARFPNLLVNGAAGIAVGMATNIPPHNLTEVIDGVLSLSKNPDISISELMEDIQGPDFPTAGLILGKSGIRRAYETGRGS**

8 19428wF1_P912 100.0% 100.0%  **PARFPNLLVNGAAGIAVGMATNIPPHNLTEVIDGVLSLSKNPDISISELMEDIQGPDFPTAGLILGKSGIRRAYETGRGS**

9 R5992 100.0% 100.0%  **PARFPNLLVNGAAGIAVGMATNIPPHNLTEVIDGVLSLSKNPDISISELMEDIQGPDFPTAGLILGKSGIRRAYETGRGS**

10 R5990 100.0% 100.0%  **PARFPNLLVNGAAGIAVGMATNIPPHNLTEVIDGVLSLSKNPDISISELMEDIQGPDFPTAGLILGKSGIRRAYETGRGS**

11 R5991 100.0% 100.0%  **PARFPNLLVNGAAGIAVGMATNIPPHNLTEVIDGVLSLSKNPDISISELMEDIQGPDFPTAGLILGKSGIRRAYETGRGS**

12 R6028 100.0% 100.0%  **PARFPNLLVNGAAGIAVGMATNIPPHNLTEVIDGVLSLSKNPDISISELMEDIQGPDFPTAGLILGKSGIRRAYETGRGS**

13 R6027 100.0% 100.0%  **PARFPNLLVNGAAGIAVGMATNIPPHNLTEVIDGVLSLSKNPDISISELMEDIQGPDFPTAGLILGKSGIRRAYETGRGS**

14 R6024 100.0% 100.0%  **PARFPNLLVNGAAGIAVGMATNIPPHNLTEVIDGVLSLSKNPDISISELMEDIQGPDFPTAGLILGKSGIRRAYETGRGS**

15 L2_057 100.0% 100.0%  **PARFPNLLVNGAAGIAVGMATNIPPHNLTEVIDGVLSLSKNPDISISELMEDIQGPDFPTAGLILGKSGIRRAYETGRGS**

16 Cap 100.0% 99.9%  **PARFPNLLVNGAAGIAVGMATNIPPHNLTEVIDGVLSLSKNPDISISELMEDIQGPDFPTAGLILGKSGIRRAYETGRGS**

17 Ani-LG-057 100.0% 100.0%  **PARFPNLLVNGAAGIAVGMATNIPPHNLTEVIDGVLSLSKNPDISISELMEDIQGPDFPTAGLILGKSGIRRAYETGRGS**

18 Cap9.2 100.0% 99.9%  **PARFPNLLVNGAAGIAVGMATNIPPHNLTEVIDGVLSLSKNPDISISELMEDIQGPDFPTAGLILGKSGIRRAYETGRGS**

19 Cap10.1 100.0% 99.9%  **PARFPNLLVNGAAGIAVGMATNIPPHNLTEVIDGVLSLSKNPDISISELMEDIQGPDFPTAGLILGKSGIRRAYETGRGS**

20 Cap9.1 100.0% 99.9%  **PARFPNLLVNGAAGIAVGMATNIPPHNLTEVIDGVLSLSKNPDISISELMEDIQGPDFPTAGLILGKSGIRRAYETGRGS**

21 Cap10.2 100.0% 99.9%  **PARFPNLLVNGAAGIAVGMATNIPPHNLTEVIDGVLSLSKNPDISISELMEDIQGPDFPTAGLILGKSGIRRAYETGRGS**

22 acrok 100.0% 99.9%  **PARFPNLLVNGAAGIAVGMATNIPPHNLTEVIDGVLSLSKNPDISISELMEDIQGPDFPTAGLILGKSGIRRAYETGRGS**

23 acroj 100.0% 99.9%  **PARFPNLLVNGAAGIAVGMATNIPPHNLTEVIDGVLSLSKNPDISISELMEDIQGPDFPTAGLILGKSGIRRAYETGRGS**

24 acror 100.0% 100.0%  **PARFPNLLVNGAAGIAVGMATNIPPHNLTEVIDGVLSLSKNPDISISELMEDIQGPDFPTAGLILGKSGIRRAYETGRGS**

25 NJ6 100.0% 100.0%  **PARFPNLLVNGAAGIAVGMATNIPPHNLTEVIDGVLSLSKNPDISISELMEDIQGPDFPTAGLILGKSGIRRAYETGRGS**

26 EYE_450 100.0% 100.0%  **PARFPNLLVNGAAGIAVGMATNIPPHNLTEVIDGVLSLSKNPDISISELMEDIQGPDFPTAGLILGKSGIRRAYETGRGS**

27 EYE_117 100.0% 100.0%  **PARFPNLLVNGAAGIAVGMATNIPPHNLTEVIDGVLSLSKNPDISISELMEDIQGPDFPTAGLILGKSGIRRAYETGRGS**

28 EYE_410 100.0% 100.0%  **PARFPNLLVNGAAGIAVGMATNIPPHNLTEVIDGVLSLSKNPDISISELMEDIQGPDFPTAGLILGKSGIRRAYETGRGS**

29 EYE_411 100.0% 100.0%  **PARFPNLLVNGAAGIAVGMATNIPPHNLTEVIDGVLSLSKNPDISISELMEDIQGPDFPTAGLILGKSGIRRAYETGRGS**

30 MERTA18 100.0% 100.0%  **PARFPNLLVNGAAGIAVGMATNIPPHNLTEVIDGVLSLSKNPDISISELMEDIQGPDFPTAGLILGKSGIRRAYETGRGS**

31 FAIRING19B-1.2 100.0% 100.0%  **PARFPNLLVNGAAGIAVGMATNIPPHNLTEVIDGVLSLSKNPDISISELMEDIQGPDFPTAGLILGKSGIRRAYETGRGS**

32 FDAARGOS_151 100.0% 100.0%  **PARFPNLLVNGAAGIAVGMATNIPPHNLTEVIDGVLSLSKNPDISISELMEDIQGPDFPTAGLILGKSGIRRAYETGRGS**

33 SNUC5989 100.0% 100.0%  **PARFPNLLVNGAAGIAVGMATNIPPHNLTEVIDGVLSLSKNPDISISELMEDIQGPDFPTAGLILGKSGIRRAYETGRGS**

34 SNUC3412 100.0% 100.0%  **PARFPNLLVNGAAGIAVGMATNIPPHNLTEVIDGVLSLSKNPDISISELMEDIQGPDFPTAGLILGKSGIRRAYETGRGS**

35 OM08-17AT 100.0% 100.0%  **PARFPNLLVNGAAGIAVGMATNIPPHNLTEVIDGVLSLSKNPDISISELMEDIQGPDFPTAGLILGKSGIRRAYETGRGS**

36 SWO 100.0% 100.0%  **PARFPNLLVNGAAGIAVGMATNIPPHNLTEVIDGVLSLSKNPDISISELMEDIQGPDFPTAGLILGKSGIRRAYETGRGS**

37 VCU121 93.3% 100.0%  **PARFPNLLVNGAAGIAVGMATNIPPHNLTEVIDGVLSLSKNPDISISELMEDIQGPDFPTAGLILGKSGIRRAYETGRGS**

consensus/100%  **PARFPNLLVNGAAGIAVGMATNIPPHNLTEVIDGVLSLSKNPDISISELMEDIQGPDFPTAGLILGKSGIRRAYETGRGS**

consensus/90%  **PARFPNLLVNGAAGIAVGMATNIPPHNLTEVIDGVLSLSKNPDISISELMEDIQGPDFPTAGLILGKSGIRRAYETGRGS**

consensus/80%  **PARFPNLLVNGAAGIAVGMATNIPPHNLTEVIDGVLSLSKNPDISISELMEDIQGPDFPTAGLILGKSGIRRAYETGRGS**

consensus/70%  **PARFPNLLVNGAAGIAVGMATNIPPHNLTEVIDGVLSLSKNPDISISELMEDIQGPDFPTAGLILGKSGIRRAYETGRGS**

cov pid **241**  **: . . . . 3 . .** **320**

1 29AM 100.0% 100.0%  **IQMRSRAEIEERGGGRQRIVVTEIPFQVNKARMIEKIAELVRDKKIDGITDLRDETSLRTGVRVVIDIRKDANASVILNN**

2 TRPF4 100.0% 100.0%  **IQMRSRAEIEERGGGRQRIVVTEIPFQVNKARMIEKIAELVRDKKIDGITDLRDETSLRTGVRVVIDIRKDANASVILNN**

3 P912 100.0% 100.0%  **IQMRSRAEIEERGGGRQRIVVTEIPFQVNKARMIEKIAELVRDKKIDGITDLRDETSLRTGVRVVIDIRKDANASVILNN**

4 NGS-ED-1001 93.3% 99.9%  **IQMRSRAEIEERGGGRQRIVVTEIPFQVNKARMIEKIAELVRDKKIDGITDLRDETSLRTGVRVVIDIRKDANASVILNN**

5 1DB1 100.0% 100.0%  **IQMRSRAEIEERGGGRQRIVVTEIPFQVNKARMIEKIAELVRDKKIDGITDLRDETSLRTGVRVVIDIRKDANASVILNN**

6 MGYG-HGUT-02301 100.0% 100.0%  **IQMRSRAEIEERGGGRQRIVVTEIPFQVNKARMIEKIAELVRDKKIDGITDLRDETSLRTGVRVVIDIRKDANASVILNN**

7 IIF4SW-P1 100.0% 100.0%  **IQMRSRAEIEERGGGRQRIVVTEIPFQVNKARMIEKIAELVRDKKIDGITDLRDETSLRTGVRVVIDIRKDANASVILNN**

8 19428wF1_P912 100.0% 100.0%  **IQMRSRAEIEERGGGRQRIVVTEIPFQVNKARMIEKIAELVRDKKIDGITDLRDETSLRTGVRVVIDIRKDANASVILNN**

9 R5992 100.0% 100.0%  **IQMRSRAEIEERGGGRQRIVVTEIPFQVNKARMIEKIAELVRDKKIDGITDLRDETSLRTGVRVVIDIRKDANASVILNN**

10 R5990 100.0% 100.0%  **IQMRSRAEIEERGGGRQRIVVTEIPFQVNKARMIEKIAELVRDKKIDGITDLRDETSLRTGVRVVIDIRKDANASVILNN**

11 R5991 100.0% 100.0%  **IQMRSRAEIEERGGGRQRIVVTEIPFQVNKARMIEKIAELVRDKKIDGITDLRDETSLRTGVRVVIDIRKDANASVILNN**

12 R6028 100.0% 100.0%  **IQMRSRAEIEERGGGRQRIVVTEIPFQVNKARMIEKIAELVRDKKIDGITDLRDETSLRTGVRVVIDIRKDANASVILNN**

13 R6027 100.0% 100.0%  **IQMRSRAEIEERGGGRQRIVVTEIPFQVNKARMIEKIAELVRDKKIDGITDLRDETSLRTGVRVVIDIRKDANASVILNN**

14 R6024 100.0% 100.0%  **IQMRSRAEIEERGGGRQRIVVTEIPFQVNKARMIEKIAELVRDKKIDGITDLRDETSLRTGVRVVIDIRKDANASVILNN**

15 L2_057 100.0% 100.0%  **IQMRSRAEIEERGGGRQRIVVTEIPFQVNKARMIEKIAELVRDKKIDGITDLRDETSLRTGVRVVIDIRKDANASVILNN**

16 Cap 100.0% 99.9%  **IQMRSRAEIEERGGGRQRIVVTEIPFQVNKARMIEKIAELVRDKKIDGITDLRDETSLRTGVRVVIDIRKDANASVILNN**

17 Ani-LG-057 100.0% 100.0%  **IQMRSRAEIEERGGGRQRIVVTEIPFQVNKARMIEKIAELVRDKKIDGITDLRDETSLRTGVRVVIDIRKDANASVILNN**

18 Cap9.2 100.0% 99.9%  **IQMRSRAEIEERGGGRQRIVVTEIPFQVNKARMIEKIAELVRDKKIDGITDLRDETSLRTGVRVVIDIRKDANASVILNN**

19 Cap10.1 100.0% 99.9%  **IQMRSRAEIEERGGGRQRIVVTEIPFQVNKARMIEKIAELVRDKKIDGITDLRDETSLRTGVRVVIDIRKDANASVILNN**

20 Cap9.1 100.0% 99.9%  **IQMRSRAEIEERGGGRQRIVVTEIPFQVNKARMIEKIAELVRDKKIDGITDLRDETSLRTGVRVVIDIRKDANASVILNN**

21 Cap10.2 100.0% 99.9%  **IQMRSRAEIEERGGGRQRIVVTEIPFQVNKARMIEKIAELVRDKKIDGITDLRDETSLRTGVRVVIDIRKDANASVILNN**

22 acrok 100.0% 99.9%  **IQMRSRAEIEERGGGRQRIVVTEIPFQVNKARMIEKIAELVRDKKIDGITDLRDETSLRTGVRVVIDIRKDANASVILNN**

23 acroj 100.0% 99.9%  **IQMRSRAEIEERGGGRQRIVVTEIPFQVNKARMIEKIAELVRDKKIDGITDLRDETSLRTGVRVVIDIRKDANASVILNN**

24 acror 100.0% 100.0%  **IQMRSRAEIEERGGGRQRIVVTEIPFQVNKARMIEKIAELVRDKKIDGITDLRDETSLRTGVRVVIDIRKDANASVILNN**

25 NJ6 100.0% 100.0%  **IQMRSRAEIEERGGGRQRIVVTEIPFQVNKARMIEKIAELVRDKKIDGITDLRDETSLRTGVRVVIDIRKDANASVILNN**

26 EYE_450 100.0% 100.0%  **IQMRSRAEIEERGGGRQRIVVTEIPFQVNKARMIEKIAELVRDKKIDGITDLRDETSLRTGVRVVIDIRKDANASVILNN**

27 EYE_117 100.0% 100.0%  **IQMRSRAEIEERGGGRQRIVVTEIPFQVNKARMIEKIAELVRDKKIDGITDLRDETSLRTGVRVVIDIRKDANASVILNN**

28 EYE_410 100.0% 100.0%  **IQMRSRAEIEERGGGRQRIVVTEIPFQVNKARMIEKIAELVRDKKIDGITDLRDETSLRTGVRVVIDIRKDANASVILNN**

29 EYE_411 100.0% 100.0%  **IQMRSRAEIEERGGGRQRIVVTEIPFQVNKARMIEKIAELVRDKKIDGITDLRDETSLRTGVRVVIDIRKDANASVILNN**

30 MERTA18 100.0% 100.0%  **IQMRSRAEIEERGGGRQRIVVTEIPFQVNKARMIEKIAELVRDKKIDGITDLRDETSLRTGVRVVIDIRKDANASVILNN**

31 FAIRING19B-1.2 100.0% 100.0%  **IQMRSRAEIEERGGGRQRIVVTEIPFQVNKARMIEKIAELVRDKKIDGITDLRDETSLRTGVRVVIDIRKDANASVILNN**

32 FDAARGOS_151 100.0% 100.0%  **IQMRSRAEIEERGGGRQRIVVTEIPFQVNKARMIEKIAELVRDKKIDGITDLRDETSLRTGVRVVIDIRKDANASVILNN**

33 SNUC5989 100.0% 100.0%  **IQMRSRAEIEERGGGRQRIVVTEIPFQVNKARMIEKIAELVRDKKIDGITDLRDETSLRTGVRVVIDIRKDANASVILNN**

34 SNUC3412 100.0% 100.0%  **IQMRSRAEIEERGGGRQRIVVTEIPFQVNKARMIEKIAELVRDKKIDGITDLRDETSLRTGVRVVIDIRKDANASVILNN**

35 OM08-17AT 100.0% 100.0%  **IQMRSRAEIEERGGGRQRIVVTEIPFQVNKARMIEKIAELVRDKKIDGITDLRDETSLRTGVRVVIDIRKDANASVILNN**

36 SWO 100.0% 100.0%  **IQMRSRAEIEERGGGRQRIVVTEIPFQVNKARMIEKIAELVRDKKIDGITDLRDETSLRTGVRVVIDIRKDANASVILNN**

37 VCU121 93.3% 100.0%  **IQMRSRAEIEERGGGRQRIVVTEIPFQVNKARMIEKIAELVRDKKIDGITDLRDETSLRTGVRVVIDIRKDANASVILNN**

consensus/100%  **IQMRSRAEIEERGGGRQRIVVTEIPFQVNKARMIEKIAELVRDKKIDGITDLRDETSLRTGVRVVIDIRKDANASVILNN**

consensus/90%  **IQMRSRAEIEERGGGRQRIVVTEIPFQVNKARMIEKIAELVRDKKIDGITDLRDETSLRTGVRVVIDIRKDANASVILNN**

consensus/80%  **IQMRSRAEIEERGGGRQRIVVTEIPFQVNKARMIEKIAELVRDKKIDGITDLRDETSLRTGVRVVIDIRKDANASVILNN**

consensus/70%  **IQMRSRAEIEERGGGRQRIVVTEIPFQVNKARMIEKIAELVRDKKIDGITDLRDETSLRTGVRVVIDIRKDANASVILNN**

cov pid **321**  **. . : . . . . 4** **400**

1 29AM 100.0% 100.0%  **LYKQTPLQTSFGVNMIALVNGRPKLINLKEALVHYLEHQKTVVRRRTEYNLRKAKDRAHILEGLRIALDHIDEIISTIRE**

2 TRPF4 100.0% 100.0%  **LYKQTPLQTSFGVNMIALVNGRPKLINLKEALVHYLEHQKTVVRRRTEYNLRKAKDRAHILEGLRIALDHIDEIISTIRE**

3 P912 100.0% 100.0%  **LYKQTPLQTSFGVNMIALVNGRPKLINLKEALVHYLEHQKTVVRRRTEYNLRKAKDRAHILEGLRIALDHIDEIISTIRE**

4 NGS-ED-1001 93.3% 99.9%  **LYKQTPLQTSFGVNMIALVNGRPKLINLKEALVHYLEHQKTVVRRRTEYNLRKAKDRAHILEGLRIALDHIDEIISTIRE**

5 1DB1 100.0% 100.0%  **LYKQTPLQTSFGVNMIALVNGRPKLINLKEALVHYLEHQKTVVRRRTEYNLRKAKDRAHILEGLRIALDHIDEIISTIRE**

6 MGYG-HGUT-02301 100.0% 100.0%  **LYKQTPLQTSFGVNMIALVNGRPKLINLKEALVHYLEHQKTVVRRRTEYNLRKAKDRAHILEGLRIALDHIDEIISTIRE**

7 IIF4SW-P1 100.0% 100.0%  **LYKQTPLQTSFGVNMIALVNGRPKLINLKEALVHYLEHQKTVVRRRTEYNLRKAKDRAHILEGLRIALDHIDEIISTIRE**

8 19428wF1_P912 100.0% 100.0%  **LYKQTPLQTSFGVNMIALVNGRPKLINLKEALVHYLEHQKTVVRRRTEYNLRKAKDRAHILEGLRIALDHIDEIISTIRE**

9 R5992 100.0% 100.0%  **LYKQTPLQTSFGVNMIALVNGRPKLINLKEALVHYLEHQKTVVRRRTEYNLRKAKDRAHILEGLRIALDHIDEIISTIRE**

10 R5990 100.0% 100.0%  **LYKQTPLQTSFGVNMIALVNGRPKLINLKEALVHYLEHQKTVVRRRTEYNLRKAKDRAHILEGLRIALDHIDEIISTIRE**

11 R5991 100.0% 100.0%  **LYKQTPLQTSFGVNMIALVNGRPKLINLKEALVHYLEHQKTVVRRRTEYNLRKAKDRAHILEGLRIALDHIDEIISTIRE**

12 R6028 100.0% 100.0%  **LYKQTPLQTSFGVNMIALVNGRPKLINLKEALVHYLEHQKTVVRRRTEYNLRKAKDRAHILEGLRIALDHIDEIISTIRE**

13 R6027 100.0% 100.0%  **LYKQTPLQTSFGVNMIALVNGRPKLINLKEALVHYLEHQKTVVRRRTEYNLRKAKDRAHILEGLRIALDHIDEIISTIRE**

14 R6024 100.0% 100.0%  **LYKQTPLQTSFGVNMIALVNGRPKLINLKEALVHYLEHQKTVVRRRTEYNLRKAKDRAHILEGLRIALDHIDEIISTIRE**

15 L2_057 100.0% 100.0%  **LYKQTPLQTSFGVNMIALVNGRPKLINLKEALVHYLEHQKTVVRRRTEYNLRKAKDRAHILEGLRIALDHIDEIISTIRE**

16 Cap 100.0% 99.9%  **LYKQTPLQTSFGVNMIALVNGRPKLINLKEALVHYLEHQKTVVRRRTEYNLRKAKDRAHILEGLRIALDHIDEIISTIRE**

17 Ani-LG-057 100.0% 100.0%  **LYKQTPLQTSFGVNMIALVNGRPKLINLKEALVHYLEHQKTVVRRRTEYNLRKAKDRAHILEGLRIALDHIDEIISTIRE**

18 Cap9.2 100.0% 99.9%  **LYKQTPLQTSFGVNMIALVNGRPKLINLKEALVHYLEHQKTVVRRRTEYNLRKAKDRAHILEGLRIALDHIDEIISTIRE**

19 Cap10.1 100.0% 99.9%  **LYKQTPLQTSFGVNMIALVNGRPKLINLKEALVHYLEHQKTVVRRRTEYNLRKAKDRAHILEGLRIALDHIDEIISTIRE**

20 Cap9.1 100.0% 99.9%  **LYKQTPLQTSFGVNMIALVNGRPKLINLKEALVHYLEHQKTVVRRRTEYNLRKAKDRAHILEGLRIALDHIDEIISTIRE**

21 Cap10.2 100.0% 99.9%  **LYKQTPLQTSFGVNMIALVNGRPKLINLKEALVHYLEHQKTVVRRRTEYNLRKAKDRAHILEGLRIALDHIDEIISTIRE**

22 acrok 100.0% 99.9%  **LYKQTPLQTSFGVNMIALVNGRPKLINLKEALVHYLEHQKTVVRRRTEYNLRKAKDRAHILEGLRIALDHIDEIISTIRE**

23 acroj 100.0% 99.9%  **LYKQTPLQTSFGVNMIALVNGRPKLINLKEALVHYLEHQKTVVRRRTEYNLRKAKDRAHILEGLRIALDHIDEIISTIRE**

24 acror 100.0% 100.0%  **LYKQTPLQTSFGVNMIALVNGRPKLINLKEALVHYLEHQKTVVRRRTEYNLRKAKDRAHILEGLRIALDHIDEIISTIRE**

25 NJ6 100.0% 100.0%  **LYKQTPLQTSFGVNMIALVNGRPKLINLKEALVHYLEHQKTVVRRRTEYNLRKAKDRAHILEGLRIALDHIDEIISTIRE**

26 EYE_450 100.0% 100.0%  **LYKQTPLQTSFGVNMIALVNGRPKLINLKEALVHYLEHQKTVVRRRTEYNLRKAKDRAHILEGLRIALDHIDEIISTIRE**

27 EYE_117 100.0% 100.0%  **LYKQTPLQTSFGVNMIALVNGRPKLINLKEALVHYLEHQKTVVRRRTEYNLRKAKDRAHILEGLRIALDHIDEIISTIRE**

28 EYE_410 100.0% 100.0%  **LYKQTPLQTSFGVNMIALVNGRPKLINLKEALVHYLEHQKTVVRRRTEYNLRKAKDRAHILEGLRIALDHIDEIISTIRE**

29 EYE_411 100.0% 100.0%  **LYKQTPLQTSFGVNMIALVNGRPKLINLKEALVHYLEHQKTVVRRRTEYNLRKAKDRAHILEGLRIALDHIDEIISTIRE**

30 MERTA18 100.0% 100.0%  **LYKQTPLQTSFGVNMIALVNGRPKLINLKEALVHYLEHQKTVVRRRTEYNLRKAKDRAHILEGLRIALDHIDEIISTIRE**

31 FAIRING19B-1.2 100.0% 100.0%  **LYKQTPLQTSFGVNMIALVNGRPKLINLKEALVHYLEHQKTVVRRRTEYNLRKAKDRAHILEGLRIALDHIDEIISTIRE**

32 FDAARGOS_151 100.0% 100.0%  **LYKQTPLQTSFGVNMIALVNGRPKLINLKEALVHYLEHQKTVVRRRTEYNLRKAKDRAHILEGLRIALDHIDEIISTIRE**

33 SNUC5989 100.0% 100.0%  **LYKQTPLQTSFGVNMIALVNGRPKLINLKEALVHYLEHQKTVVRRRTEYNLRKAKDRAHILEGLRIALDHIDEIISTIRE**

34 SNUC3412 100.0% 100.0%  **LYKQTPLQTSFGVNMIALVNGRPKLINLKEALVHYLEHQKTVVRRRTEYNLRKAKDRAHILEGLRIALDHIDEIISTIRE**

35 OM08-17AT 100.0% 100.0%  **LYKQTPLQTSFGVNMIALVNGRPKLINLKEALVHYLEHQKTVVRRRTEYNLRKAKDRAHILEGLRIALDHIDEIISTIRE**

36 SWO 100.0% 100.0%  **LYKQTPLQTSFGVNMIALVNGRPKLINLKEALVHYLEHQKTVVRRRTEYNLRKAKDRAHILEGLRIALDHIDEIISTIRE**

37 VCU121 93.3% 100.0%  **LYKQTPLQTSFGVNMIALVNGRPKLINLKEALVHYLEHQKTVVRRRTEYNLRKAKDRAHILEGLRIALDHIDEIISTIRE**

consensus/100%  **LYKQTPLQTSFGVNMIALVNGRPKLINLKEALVHYLEHQKTVVRRRTEYNLRKAKDRAHILEGLRIALDHIDEIISTIRE**

consensus/90%  **LYKQTPLQTSFGVNMIALVNGRPKLINLKEALVHYLEHQKTVVRRRTEYNLRKAKDRAHILEGLRIALDHIDEIISTIRE**

consensus/80%  **LYKQTPLQTSFGVNMIALVNGRPKLINLKEALVHYLEHQKTVVRRRTEYNLRKAKDRAHILEGLRIALDHIDEIISTIRE**

consensus/70%  **LYKQTPLQTSFGVNMIALVNGRPKLINLKEALVHYLEHQKTVVRRRTEYNLRKAKDRAHILEGLRIALDHIDEIISTIRE**

cov pid **401**  **. . . . : . . .** **480**

1 29AM 100.0% 100.0%  **SETDKVAMESLQERFKLSERQAQAILDMRLRRLTGLERDKIESEYNELLAYIDELEAILADEEVLLQLVRDELTEIKERY**

2 TRPF4 100.0% 100.0%  **SETDKVAMESLQERFKLSERQAQAILDMRLRRLTGLERDKIESEYNELLAYIDELEAILADEEVLLQLVRDELTEIKERY**

3 P912 100.0% 100.0%  **SETDKVAMESLQERFKLSERQAQAILDMRLRRLTGLERDKIESEYNELLAYIDELEAILADEEVLLQLVRDELTEIKERY**

4 NGS-ED-1001 93.3% 99.9%  **SETDKVAMESLQERFKLSERQAQAILDMRLRRLTGLERDKIESEYNELLAYIDELESILADEEVLLQLVRDELTEIKERY**

5 1DB1 100.0% 100.0%  **SETDKVAMESLQERFKLSERQAQAILDMRLRRLTGLERDKIESEYNELLAYIDELEAILADEEVLLQLVRDELTEIKERY**

6 MGYG-HGUT-02301 100.0% 100.0%  **SETDKVAMESLQERFKLSERQAQAILDMRLRRLTGLERDKIESEYNELLAYIDELEAILADEEVLLQLVRDELTEIKERY**

7 IIF4SW-P1 100.0% 100.0%  **SETDKVAMESLQERFKLSERQAQAILDMRLRRLTGLERDKIESEYNELLAYIDELEAILADEEVLLQLVRDELTEIKERY**

8 19428wF1_P912 100.0% 100.0%  **SETDKVAMESLQERFKLSERQAQAILDMRLRRLTGLERDKIESEYNELLAYIDELEAILADEEVLLQLVRDELTEIKERY**

9 R5992 100.0% 100.0%  **SETDKVAMESLQERFKLSERQAQAILDMRLRRLTGLERDKIESEYNELLAYIDELEAILADEEVLLQLVRDELTEIKERY**

10 R5990 100.0% 100.0%  **SETDKVAMESLQERFKLSERQAQAILDMRLRRLTGLERDKIESEYNELLAYIDELEAILADEEVLLQLVRDELTEIKERY**

11 R5991 100.0% 100.0%  **SETDKVAMESLQERFKLSERQAQAILDMRLRRLTGLERDKIESEYNELLAYIDELEAILADEEVLLQLVRDELTEIKERY**

12 R6028 100.0% 100.0%  **SETDKVAMESLQERFKLSERQAQAILDMRLRRLTGLERDKIESEYNELLAYIDELEAILADEEVLLQLVRDELTEIKERY**

13 R6027 100.0% 100.0%  **SETDKVAMESLQERFKLSERQAQAILDMRLRRLTGLERDKIESEYNELLAYIDELEAILADEEVLLQLVRDELTEIKERY**

14 R6024 100.0% 100.0%  **SETDKVAMESLQERFKLSERQAQAILDMRLRRLTGLERDKIESEYNELLAYIDELEAILADEEVLLQLVRDELTEIKERY**

15 L2_057 100.0% 100.0%  **SETDKVAMESLQERFKLSERQAQAILDMRLRRLTGLERDKIESEYNELLAYIDELEAILADEEVLLQLVRDELTEIKERY**

16 Cap 100.0% 99.9%  **SETDKVAMESLQERFKLSERQAQAILDMRLRRLTGLERDKIESEYNELLAYIGELEAILADEEVLLQLVRDELTEIKERY**

17 Ani-LG-057 100.0% 100.0%  **SETDKVAMESLQERFKLSERQAQAILDMRLRRLTGLERDKIESEYNELLAYIDELEAILADEEVLLQLVRDELTEIKERY**

18 Cap9.2 100.0% 99.9%  **SETDKVAMESLQERFKLSERQAQAILDMRLRRLTGLERDKIESEYNELLAYIGELEAILADEEVLLQLVRDELTEIKERY**

19 Cap10.1 100.0% 99.9%  **SETDKVAMESLQERFKLSERQAQAILDMRLRRLTGLERDKIESEYNELLAYIGELEAILADEEVLLQLVRDELTEIKERY**

20 Cap9.1 100.0% 99.9%  **SETDKVAMESLQERFKLSERQAQAILDMRLRRLTGLERDKIESEYNELLAYIGELEAILADEEVLLQLVRDELTEIKERY**

21 Cap10.2 100.0% 99.9%  **SETDKVAMESLQERFKLSERQAQAILDMRLRRLTGLERDKIESEYNELLAYIGELEAILADEEVLLQLVRDELTEIKERY**

22 acrok 100.0% 99.9%  **SETDKVAMESLQERFKLSERQAQAILDMRLRRLTGLERDKIESEYNELLAYIDELEAILADEEVLLQLVRDELTEIKERY**

23 acroj 100.0% 99.9%  **SETDKVAMESLQERFKLSERQAQAILDMRLRRLTGLERDKIESEYNELLAYIDELEAILADEEVLLQLVRDELTEIKERY**

24 acror 100.0% 100.0%  **SETDKVAMESLQERFKLSERQAQAILDMRLRRLTGLERDKIESEYNELLAYIDELEAILADEEVLLQLVRDELTEIKERY**

25 NJ6 100.0% 100.0%  **SETDKVAMESLQERFKLSERQAQAILDMRLRRLTGLERDKIESEYNELLAYIDELEAILADEEVLLQLVRDELTEIKERY**

26 EYE_450 100.0% 100.0%  **SETDKVAMESLQERFKLSERQAQAILDMRLRRLTGLERDKIESEYNELLAYIDELEAILADEEVLLQLVRDELTEIKERY**

27 EYE_117 100.0% 100.0%  **SETDKVAMESLQERFKLSERQAQAILDMRLRRLTGLERDKIESEYNELLAYIDELEAILADEEVLLQLVRDELTEIKERY**

28 EYE_410 100.0% 100.0%  **SETDKVAMESLQERFKLSERQAQAILDMRLRRLTGLERDKIESEYNELLAYIDELEAILADEEVLLQLVRDELTEIKERY**

29 EYE_411 100.0% 100.0%  **SETDKVAMESLQERFKLSERQAQAILDMRLRRLTGLERDKIESEYNELLAYIDELEAILADEEVLLQLVRDELTEIKERY**

30 MERTA18 100.0% 100.0%  **SETDKVAMESLQERFKLSERQAQAILDMRLRRLTGLERDKIESEYNELLAYIDELEAILADEEVLLQLVRDELTEIKERY**

31 FAIRING19B-1.2 100.0% 100.0%  **SETDKVAMESLQERFKLSERQAQAILDMRLRRLTGLERDKIESEYNELLAYIDELEAILADEEVLLQLVRDELTEIKERY**

32 FDAARGOS_151 100.0% 100.0%  **SETDKVAMESLQERFKLSERQAQAILDMRLRRLTGLERDKIESEYNELLAYIDELEAILADEEVLLQLVRDELTEIKERY**

33 SNUC5989 100.0% 100.0%  **SETDKVAMESLQERFKLSERQAQAILDMRLRRLTGLERDKIESEYNELLAYIDELEAILADEEVLLQLVRDELTEIKERY**

34 SNUC3412 100.0% 100.0%  **SETDKVAMESLQERFKLSERQAQAILDMRLRRLTGLERDKIESEYNELLAYIDELEAILADEEVLLQLVRDELTEIKERY**

35 OM08-17AT 100.0% 100.0%  **SETDKVAMESLQERFKLSERQAQAILDMRLRRLTGLERDKIESEYNELLAYIDELEAILADEEVLLQLVRDELTEIKERY**

36 SWO 100.0% 100.0%  **SETDKVAMESLQERFKLSERQAQAILDMRLRRLTGLERDKIESEYNELLAYIDELEAILADEEVLLQLVRDELTEIKERY**

37 VCU121 93.3% 100.0%  **SETDKVAMESLQERFKLSERQAQAILDMRLRRLTGLERDKIESEYNELLAYIDELEAILADEEVLLQLVRDELTEIKERY**

consensus/100%  **SETDKVAMESLQERFKLSERQAQAILDMRLRRLTGLERDKIESEYNELLAYIsELEuILADEEVLLQLVRDELTEIKERY**

consensus/90%  **SETDKVAMESLQERFKLSERQAQAILDMRLRRLTGLERDKIESEYNELLAYIsELEAILADEEVLLQLVRDELTEIKERY**

consensus/80%  **SETDKVAMESLQERFKLSERQAQAILDMRLRRLTGLERDKIESEYNELLAYIDELEAILADEEVLLQLVRDELTEIKERY**

consensus/70%  **SETDKVAMESLQERFKLSERQAQAILDMRLRRLTGLERDKIESEYNELLAYIDELEAILADEEVLLQLVRDELTEIKERY**

cov pid **481**  **. 5 . . . . : .** **560**

1 29AM 100.0% 100.0%  **GDERRTEIQLGGLDDLEDEDLIPEEQIVITLSHNNYIKRLPVSTYRAQNRGGRGVQGMNTLEEDFVSQLVTLSTHDNVLF**

2 TRPF4 100.0% 100.0%  **GDERRTEIQLGGLDDLEDEDLIPEEQIVITLSHNNYIKRLPVSTYRAQNRGGRGVQGMNTLEEDFVSQLVTLSTHDNVLF**

3 P912 100.0% 100.0%  **GDERRTEIQLGGLDDLEDEDLIPEEQIVITLSHNNYIKRLPVSTYRAQNRGGRGVQGMNTLEEDFVSQLVTLSTHDNVLF**

4 NGS-ED-1001 93.3% 99.9%  **GDERRTEIQLGGLDDLEDEDLIPEEQIVITLSHNNYIKRLPVSTYRAQNRGGRGVQGMNTLEEDFVSQLVTLSTHDNVLF**

5 1DB1 100.0% 100.0%  **GDERRTEIQLGGLDDLEDEDLIPEEQIVITLSHNNYIKRLPVSTYRAQNRGGRGVQGMNTLEEDFVSQLVTLSTHDNVLF**

6 MGYG-HGUT-02301 100.0% 100.0%  **GDERRTEIQLGGLDDLEDEDLIPEEQIVITLSHNNYIKRLPVSTYRAQNRGGRGVQGMNTLEEDFVSQLVTLSTHDNVLF**

7 IIF4SW-P1 100.0% 100.0%  **GDERRTEIQLGGLDDLEDEDLIPEEQIVITLSHNNYIKRLPVSTYRAQNRGGRGVQGMNTLEEDFVSQLVTLSTHDNVLF**

8 19428wF1_P912 100.0% 100.0%  **GDERRTEIQLGGLDDLEDEDLIPEEQIVITLSHNNYIKRLPVSTYRAQNRGGRGVQGMNTLEEDFVSQLVTLSTHDNVLF**

9 R5992 100.0% 100.0%  **GDERRTEIQLGGLDDLEDEDLIPEEQIVITLSHNNYIKRLPVSTYRAQNRGGRGVQGMNTLEEDFVSQLVTLSTHDNVLF**

10 R5990 100.0% 100.0%  **GDERRTEIQLGGLDDLEDEDLIPEEQIVITLSHNNYIKRLPVSTYRAQNRGGRGVQGMNTLEEDFVSQLVTLSTHDNVLF**

11 R5991 100.0% 100.0%  **GDERRTEIQLGGLDDLEDEDLIPEEQIVITLSHNNYIKRLPVSTYRAQNRGGRGVQGMNTLEEDFVSQLVTLSTHDNVLF**

12 R6028 100.0% 100.0%  **GDERRTEIQLGGLDDLEDEDLIPEEQIVITLSHNNYIKRLPVSTYRAQNRGGRGVQGMNTLEEDFVSQLVTLSTHDNVLF**

13 R6027 100.0% 100.0%  **GDERRTEIQLGGLDDLEDEDLIPEEQIVITLSHNNYIKRLPVSTYRAQNRGGRGVQGMNTLEEDFVSQLVTLSTHDNVLF**

14 R6024 100.0% 100.0%  **GDERRTEIQLGGLDDLEDEDLIPEEQIVITLSHNNYIKRLPVSTYRAQNRGGRGVQGMNTLEEDFVSQLVTLSTHDNVLF**

15 L2_057 100.0% 100.0%  **GDERRTEIQLGGLDDLEDEDLIPEEQIVITLSHNNYIKRLPVSTYRAQNRGGRGVQGMNTLEEDFVSQLVTLSTHDNVLF**

16 Cap 100.0% 99.9%  **GDERRTEIQLGGLDDLEDEDLIPEEQIVITLSHNNYIKRLPVSTYRAQNRGGRGVQGMNTLEEDFVSQLVTLSTHDNVLF**

17 Ani-LG-057 100.0% 100.0%  **GDERRTEIQLGGLDDLEDEDLIPEEQIVITLSHNNYIKRLPVSTYRAQNRGGRGVQGMNTLEEDFVSQLVTLSTHDNVLF**

18 Cap9.2 100.0% 99.9%  **GDERRTEIQLGGLDDLEDEDLIPEEQIVITLSHNNYIKRLPVSTYRAQNRGGRGVQGMNTLEEDFVSQLVTLSTHDNVLF**

19 Cap10.1 100.0% 99.9%  **GDERRTEIQLGGLDDLEDEDLIPEEQIVITLSHNNYIKRLPVSTYRAQNRGGRGVQGMNTLEEDFVSQLVTLSTHDNVLF**

20 Cap9.1 100.0% 99.9%  **GDERRTEIQLGGLDDLEDEDLIPEEQIVITLSHNNYIKRLPVSTYRAQNRGGRGVQGMNTLEEDFVSQLVTLSTHDNVLF**

21 Cap10.2 100.0% 99.9%  **GDERRTEIQLGGLDDLEDEDLIPEEQIVITLSHNNYIKRLPVSTYRAQNRGGRGVQGMNTLEEDFVSQLVTLSTHDNVLF**

22 acrok 100.0% 99.9%  **GDERRTEIQLGRLDDLEDEDLIPEEQIVITLSHNNYIKRLPVSTYRAQNRGGRGVQGMNTLEEDFVSQLVTLSTHDNVLF**

23 acroj 100.0% 99.9%  **GDERRTEIQLGRLDDLEDEDLIPEEQIVITLSHNNYIKRLPVSTYRAQNRGGRGVQGMNTLEEDFVSQLVTLSTHDNVLF**

24 acror 100.0% 100.0%  **GDERRTEIQLGGLDDLEDEDLIPEEQIVITLSHNNYIKRLPVSTYRAQNRGGRGVQGMNTLEEDFVSQLVTLSTHDNVLF**

25 NJ6 100.0% 100.0%  **GDERRTEIQLGGLDDLEDEDLIPEEQIVITLSHNNYIKRLPVSTYRAQNRGGRGVQGMNTLEEDFVSQLVTLSTHDNVLF**

26 EYE_450 100.0% 100.0%  **GDERRTEIQLGGLDDLEDEDLIPEEQIVITLSHNNYIKRLPVSTYRAQNRGGRGVQGMNTLEEDFVSQLVTLSTHDNVLF**

27 EYE_117 100.0% 100.0%  **GDERRTEIQLGGLDDLEDEDLIPEEQIVITLSHNNYIKRLPVSTYRAQNRGGRGVQGMNTLEEDFVSQLVTLSTHDNVLF**

28 EYE_410 100.0% 100.0%  **GDERRTEIQLGGLDDLEDEDLIPEEQIVITLSHNNYIKRLPVSTYRAQNRGGRGVQGMNTLEEDFVSQLVTLSTHDNVLF**

29 EYE_411 100.0% 100.0%  **GDERRTEIQLGGLDDLEDEDLIPEEQIVITLSHNNYIKRLPVSTYRAQNRGGRGVQGMNTLEEDFVSQLVTLSTHDNVLF**

30 MERTA18 100.0% 100.0%  **GDERRTEIQLGGLDDLEDEDLIPEEQIVITLSHNNYIKRLPVSTYRAQNRGGRGVQGMNTLEEDFVSQLVTLSTHDNVLF**

31 FAIRING19B-1.2 100.0% 100.0%  **GDERRTEIQLGGLDDLEDEDLIPEEQIVITLSHNNYIKRLPVSTYRAQNRGGRGVQGMNTLEEDFVSQLVTLSTHDNVLF**

32 FDAARGOS_151 100.0% 100.0%  **GDERRTEIQLGGLDDLEDEDLIPEEQIVITLSHNNYIKRLPVSTYRAQNRGGRGVQGMNTLEEDFVSQLVTLSTHDNVLF**

33 SNUC5989 100.0% 100.0%  **GDERRTEIQLGGLDDLEDEDLIPEEQIVITLSHNNYIKRLPVSTYRAQNRGGRGVQGMNTLEEDFVSQLVTLSTHDNVLF**

34 SNUC3412 100.0% 100.0%  **GDERRTEIQLGGLDDLEDEDLIPEEQIVITLSHNNYIKRLPVSTYRAQNRGGRGVQGMNTLEEDFVSQLVTLSTHDNVLF**

35 OM08-17AT 100.0% 100.0%  **GDERRTEIQLGGLDDLEDEDLIPEEQIVITLSHNNYIKRLPVSTYRAQNRGGRGVQGMNTLEEDFVSQLVTLSTHDNVLF**

36 SWO 100.0% 100.0%  **GDERRTEIQLGGLDDLEDEDLIPEEQIVITLSHNNYIKRLPVSTYRAQNRGGRGVQGMNTLEEDFVSQLVTLSTHDNVLF**

37 VCU121 93.3% 100.0%  **GDERRTEIQLGGLDDLEDEDLIPEEQIVITLSHNNYIKRLPVSTYRAQNRGGRGVQGMNTLEEDFVSQLVTLSTHDNVLF**

consensus/100%  **GDERRTEIQLGtLDDLEDEDLIPEEQIVITLSHNNYIKRLPVSTYRAQNRGGRGVQGMNTLEEDFVSQLVTLSTHDNVLF**

consensus/90%  **GDERRTEIQLGGLDDLEDEDLIPEEQIVITLSHNNYIKRLPVSTYRAQNRGGRGVQGMNTLEEDFVSQLVTLSTHDNVLF**

consensus/80%  **GDERRTEIQLGGLDDLEDEDLIPEEQIVITLSHNNYIKRLPVSTYRAQNRGGRGVQGMNTLEEDFVSQLVTLSTHDNVLF**

consensus/70%  **GDERRTEIQLGGLDDLEDEDLIPEEQIVITLSHNNYIKRLPVSTYRAQNRGGRGVQGMNTLEEDFVSQLVTLSTHDNVLF**

cov pid **561**  **. . . 6 . . . .** **640**

1 29AM 100.0% 100.0%  **FTNKGRVYKLKGYEVPELSRQSKGIPIVNAIELENDESISTMIAVKDLESEDDYLVFATRKGIVKRSSLSNFSRINKNGK**

2 TRPF4 100.0% 100.0%  **FTNKGRVYKLKGYEVPELSRQSKGIPIVNAIELENDESISTMIAVKDLESEDDYLVFATRKGIVKRSSLSNFSRINKNGK**

3 P912 100.0% 100.0%  **FTNKGRVYKLKGYEVPELSRQSKGIPIVNAIELENDESISTMIAVKDLESEDDYLVFATRKGIVKRSSLSNFSRINKNGK**

4 NGS-ED-1001 93.3% 99.9%  **FTNKGRVYKLKGYEVPELSRQSKGIPIVNAIELENDESISTMIAVKDLESEDDYLVFATRKGIVKRSSLSNFSRINKNGK**

5 1DB1 100.0% 100.0%  **FTNKGRVYKLKGYEVPELSRQSKGIPIVNAIELENDESISTMIAVKDLESEDDYLVFATRKGIVKRSSLSNFSRINKNGK**

6 MGYG-HGUT-02301 100.0% 100.0%  **FTNKGRVYKLKGYEVPELSRQSKGIPIVNAIELENDESISTMIAVKDLESEDDYLVFATRKGIVKRSSLSNFSRINKNGK**

7 IIF4SW-P1 100.0% 100.0%  **FTNKGRVYKLKGYEVPELSRQSKGIPIVNAIELENDESISTMIAVKDLESEDDYLVFATRKGIVKRSSLSNFSRINKNGK**

8 19428wF1_P912 100.0% 100.0%  **FTNKGRVYKLKGYEVPELSRQSKGIPIVNAIELENDESISTMIAVKDLESEDDYLVFATRKGIVKRSSLSNFSRINKNGK**

9 R5992 100.0% 100.0%  **FTNKGRVYKLKGYEVPELSRQSKGIPIVNAIELENDESISTMIAVKDLESEDDYLVFATRKGIVKRSSLSNFSRINKNGK**

10 R5990 100.0% 100.0%  **FTNKGRVYKLKGYEVPELSRQSKGIPIVNAIELENDESISTMIAVKDLESEDDYLVFATRKGIVKRSSLSNFSRINKNGK**

11 R5991 100.0% 100.0%  **FTNKGRVYKLKGYEVPELSRQSKGIPIVNAIELENDESISTMIAVKDLESEDDYLVFATRKGIVKRSSLSNFSRINKNGK**

12 R6028 100.0% 100.0%  **FTNKGRVYKLKGYEVPELSRQSKGIPIVNAIELENDESISTMIAVKDLESEDDYLVFATRKGIVKRSSLSNFSRINKNGK**

13 R6027 100.0% 100.0%  **FTNKGRVYKLKGYEVPELSRQSKGIPIVNAIELENDESISTMIAVKDLESEDDYLVFATRKGIVKRSSLSNFSRINKNGK**

14 R6024 100.0% 100.0%  **FTNKGRVYKLKGYEVPELSRQSKGIPIVNAIELENDESISTMIAVKDLESEDDYLVFATRKGIVKRSSLSNFSRINKNGK**

15 L2_057 100.0% 100.0%  **FTNKGRVYKLKGYEVPELSRQSKGIPIVNAIELENDESISTMIAVKDLESEDDYLVFATRKGIVKRSSLSNFSRINKNGK**

16 Cap 100.0% 99.9%  **FTNKGRVYKLKGYEVPELSRQSKGIPIVNAIELENDESISTMIAVKDLESEDDYLVFATRKGIVKRSSLSNFSRINKNGK**

17 Ani-LG-057 100.0% 100.0%  **FTNKGRVYKLKGYEVPELSRQSKGIPIVNAIELENDESISTMIAVKDLESEDDYLVFATRKGIVKRSSLSNFSRINKNGK**

18 Cap9.2 100.0% 99.9%  **FTNKGRVYKLKGYEVPELSRQSKGIPIVNAIELENDESISTMIAVKDLESEDDYLVFATRKGIVKRSSLSNFSRINKNGK**

19 Cap10.1 100.0% 99.9%  **FTNKGRVYKLKGYEVPELSRQSKGIPIVNAIELENDESISTMIAVKDLESEDDYLVFATRKGIVKRSSLSNFSRINKNGK**

20 Cap9.1 100.0% 99.9%  **FTNKGRVYKLKGYEVPELSRQSKGIPIVNAIELENDESISTMIAVKDLESEDDYLVFATRKGIVKRSSLSNFSRINKNGK**

21 Cap10.2 100.0% 99.9%  **FTNKGRVYKLKGYEVPELSRQSKGIPIVNAIELENDESISTMIAVKDLESEDDYLVFATRKGIVKRSSLSNFSRINKNGK**

22 acrok 100.0% 99.9%  **FTNKGRVYKLKGYEVPELSRQSKGIPIVNAIELENDESISTMIAVKDLESEDDYLVFATRKGIVKRSSLSNFSRINKNGK**

23 acroj 100.0% 99.9%  **FTNKGRVYKLKGYEVPELSRQSKGIPIVNAIELENDESISTMIAVKDLESEDDYLVFATRKGIVKRSSLSNFSRINKNGK**

24 acror 100.0% 100.0%  **FTNKGRVYKLKGYEVPELSRQSKGIPIVNAIELENDESISTMIAVKDLESEDDYLVFATRKGIVKRSSLSNFSRINKNGK**

25 NJ6 100.0% 100.0%  **FTNKGRVYKLKGYEVPELSRQSKGIPIVNAIELENDESISTMIAVKDLESEDDYLVFATRKGIVKRSSLSNFSRINKNGK**

26 EYE_450 100.0% 100.0%  **FTNKGRVYKLKGYEVPELSRQSKGIPIVNAIELENDESISTMIAVKDLESEDDYLVFATRKGIVKRSSLSNFSRINKNGK**

27 EYE_117 100.0% 100.0%  **FTNKGRVYKLKGYEVPELSRQSKGIPIVNAIELENDESISTMIAVKDLESEDDYLVFATRKGIVKRSSLSNFSRINKNGK**

28 EYE_410 100.0% 100.0%  **FTNKGRVYKLKGYEVPELSRQSKGIPIVNAIELENDESISTMIAVKDLESEDDYLVFATRKGIVKRSSLSNFSRINKNGK**

29 EYE_411 100.0% 100.0%  **FTNKGRVYKLKGYEVPELSRQSKGIPIVNAIELENDESISTMIAVKDLESEDDYLVFATRKGIVKRSSLSNFSRINKNGK**

30 MERTA18 100.0% 100.0%  **FTNKGRVYKLKGYEVPELSRQSKGIPIVNAIELENDESISTMIAVKDLESEDDYLVFATRKGIVKRSSLSNFSRINKNGK**

31 FAIRING19B-1.2 100.0% 100.0%  **FTNKGRVYKLKGYEVPELSRQSKGIPIVNAIELENDESISTMIAVKDLESEDDYLVFATRKGIVKRSSLSNFSRINKNGK**

32 FDAARGOS_151 100.0% 100.0%  **FTNKGRVYKLKGYEVPELSRQSKGIPIVNAIELENDESISTMIAVKDLESEDDYLVFATRKGIVKRSSLSNFSRINKNGK**

33 SNUC5989 100.0% 100.0%  **FTNKGRVYKLKGYEVPELSRQSKGIPIVNAIELENDESISTMIAVKDLESEDDYLVFATRKGIVKRSSLSNFSRINKNGK**

34 SNUC3412 100.0% 100.0%  **FTNKGRVYKLKGYEVPELSRQSKGIPIVNAIELENDESISTMIAVKDLESEDDYLVFATRKGIVKRSSLSNFSRINKNGK**

35 OM08-17AT 100.0% 100.0%  **FTNKGRVYKLKGYEVPELSRQSKGIPIVNAIELENDESISTMIAVKDLESEDDYLVFATRKGIVKRSSLSNFSRINKNGK**

36 SWO 100.0% 100.0%  **FTNKGRVYKLKGYEVPELSRQSKGIPIVNAIELENDESISTMIAVKDLESEDDYLVFATRKGIVKRSSLSNFSRINKNGK**

37 VCU121 93.3% 100.0%  **FTNKGRVYKLKGYEVPELSRQSKGIPIVNAIELENDESISTMIAVKDLESEDDYLVFATRKGIVKRSSLSNFSRINKNGK**

consensus/100%  **FTNKGRVYKLKGYEVPELSRQSKGIPIVNAIELENDESISTMIAVKDLESEDDYLVFATRKGIVKRSSLSNFSRINKNGK**

consensus/90%  **FTNKGRVYKLKGYEVPELSRQSKGIPIVNAIELENDESISTMIAVKDLESEDDYLVFATRKGIVKRSSLSNFSRINKNGK**

consensus/80%  **FTNKGRVYKLKGYEVPELSRQSKGIPIVNAIELENDESISTMIAVKDLESEDDYLVFATRKGIVKRSSLSNFSRINKNGK**

consensus/70%  **FTNKGRVYKLKGYEVPELSRQSKGIPIVNAIELENDESISTMIAVKDLESEDDYLVFATRKGIVKRSSLSNFSRINKNGK**

cov pid **641**  **: . . . . 7 . .** **720**

1 29AM 100.0% 100.0%  **IAINFKEDDELIAVRLTDGEEDILIGTAHASLIRFSEKALRPLGRTAAGVKGITLREGDEVVGLDVAHANSEDEVLVVTE**

2 TRPF4 100.0% 100.0%  **IAINFKEDDELIAVRLTDGEEDILIGTAHASLIRFSEKALRPLGRTAAGVKGITLREGDEVVGLDVAHANSEDEVLVVTE**

3 P912 100.0% 100.0%  **IAINFKEDDELIAVRLTDGEEDILIGTAHASLIRFSEKALRPLGRTAAGVKGITLREGDEVVGLDVAHANSEDEVLVVTE**

4 NGS-ED-1001 93.3% 99.9%  **IAINFKEDDELIAVRLTDGEEDILIGTAHASLIRFSEKALRPLGRTAAGVKGITLREGDEVVGLDVAHANSEDEVLVVTE**

5 1DB1 100.0% 100.0%  **IAINFKEDDELIAVRLTDGEEDILIGTAHASLIRFSEKALRPLGRTAAGVKGITLREGDEVVGLDVAHANSEDEVLVVTE**

6 MGYG-HGUT-02301 100.0% 100.0%  **IAINFKEDDELIAVRLTDGEEDILIGTAHASLIRFSEKALRPLGRTAAGVKGITLREGDEVVGLDVAHANSEDEVLVVTE**

7 IIF4SW-P1 100.0% 100.0%  **IAINFKEDDELIAVRLTDGEEDILIGTAHASLIRFSEKALRPLGRTAAGVKGITLREGDEVVGLDVAHANSEDEVLVVTE**

8 19428wF1_P912 100.0% 100.0%  **IAINFKEDDELIAVRLTDGEEDILIGTAHASLIRFSEKALRPLGRTAAGVKGITLREGDEVVGLDVAHANSEDEVLVVTE**

9 R5992 100.0% 100.0%  **IAINFKEDDELIAVRLTDGEEDILIGTAHASLIRFSEKALRPLGRTAAGVKGITLREGDEVVGLDVAHANSEDEVLVVTE**

10 R5990 100.0% 100.0%  **IAINFKEDDELIAVRLTDGEEDILIGTAHASLIRFSEKALRPLGRTAAGVKGITLREGDEVVGLDVAHANSEDEVLVVTE**

11 R5991 100.0% 100.0%  **IAINFKEDDELIAVRLTDGEEDILIGTAHASLIRFSEKALRPLGRTAAGVKGITLREGDEVVGLDVAHANSEDEVLVVTE**

12 R6028 100.0% 100.0%  **IAINFKEDDELIAVRLTDGEEDILIGTAHASLIRFSEKALRPLGRTAAGVKGITLREGDEVVGLDVAHANSEDEVLVVTE**

13 R6027 100.0% 100.0%  **IAINFKEDDELIAVRLTDGEEDILIGTAHASLIRFSEKALRPLGRTAAGVKGITLREGDEVVGLDVAHANSEDEVLVVTE**

14 R6024 100.0% 100.0%  **IAINFKEDDELIAVRLTDGEEDILIGTAHASLIRFSEKALRPLGRTAAGVKGITLREGDEVVGLDVAHANSEDEVLVVTE**

15 L2_057 100.0% 100.0%  **IAINFKEDDELIAVRLTDGEEDILIGTAHASLIRFSEKALRPLGRTAAGVKGITLREGDEVVGLDVAHANSEDEVLVVTE**

16 Cap 100.0% 99.9%  **IAINFKEDDELIAVRLTDGEEDILIGTAHASLIRFSEKALRPLGRTAAGVKGITLREGDEVVGLDVAHANSEDEVLVVTE**

17 Ani-LG-057 100.0% 100.0%  **IAINFKEDDELIAVRLTDGEEDILIGTAHASLIRFSEKALRPLGRTAAGVKGITLREGDEVVGLDVAHANSEDEVLVVTE**

18 Cap9.2 100.0% 99.9%  **IAINFKEDDELIAVRLTDGEEDILIGTAHASLIRFSEKALRPLGRTAAGVKGITLREGDEVVGLDVAHANSEDEVLVVTE**

19 Cap10.1 100.0% 99.9%  **IAINFKEDDELIAVRLTDGEEDILIGTAHASLIRFSEKALRPLGRTAAGVKGITLREGDEVVGLDVAHANSEDEVLVVTE**

20 Cap9.1 100.0% 99.9%  **IAINFKEDDELIAVRLTDGEEDILIGTAHASLIRFSEKALRPLGRTAAGVKGITLREGDEVVGLDVAHANSEDEVLVVTE**

21 Cap10.2 100.0% 99.9%  **IAINFKEDDELIAVRLTDGEEDILIGTAHASLIRFSEKALRPLGRTAAGVKGITLREGDEVVGLDVAHANSEDEVLVVTE**

22 acrok 100.0% 99.9%  **IAINFKEDDELIAVRLTDGEEDILIGTAHASLIRFSEKALRPLGRTAAGVKGITLREGDEVVGLDVAHANSEDEVLVVTE**

23 acroj 100.0% 99.9%  **IAINFKEDDELIAVRLTDGEEDILIGTAHASLIRFSEKALRPLGRTAAGVKGITLREGDEVVGLDVAHANSEDEVLVVTE**

24 acror 100.0% 100.0%  **IAINFKEDDELIAVRLTDGEEDILIGTAHASLIRFSEKALRPLGRTAAGVKGITLREGDEVVGLDVAHANSEDEVLVVTE**

25 NJ6 100.0% 100.0%  **IAINFKEDDELIAVRLTDGEEDILIGTAHASLIRFSEKALRPLGRTAAGVKGITLREGDEVVGLDVAHANSEDEVLVVTE**

26 EYE_450 100.0% 100.0%  **IAINFKEDDELIAVRLTDGEEDILIGTAHASLIRFSEKALRPLGRTAAGVKGITLREGDEVVGLDVAHANSEDEVLVVTE**

27 EYE_117 100.0% 100.0%  **IAINFKEDDELIAVRLTDGEEDILIGTAHASLIRFSEKALRPLGRTAAGVKGITLREGDEVVGLDVAHANSEDEVLVVTE**

28 EYE_410 100.0% 100.0%  **IAINFKEDDELIAVRLTDGEEDILIGTAHASLIRFSEKALRPLGRTAAGVKGITLREGDEVVGLDVAHANSEDEVLVVTE**

29 EYE_411 100.0% 100.0%  **IAINFKEDDELIAVRLTDGEEDILIGTAHASLIRFSEKALRPLGRTAAGVKGITLREGDEVVGLDVAHANSEDEVLVVTE**

30 MERTA18 100.0% 100.0%  **IAINFKEDDELIAVRLTDGEEDILIGTAHASLIRFSEKALRPLGRTAAGVKGITLREGDEVVGLDVAHANSEDEVLVVTE**

31 FAIRING19B-1.2 100.0% 100.0%  **IAINFKEDDELIAVRLTDGEEDILIGTAHASLIRFSEKALRPLGRTAAGVKGITLREGDEVVGLDVAHANSEDEVLVVTE**

32 FDAARGOS_151 100.0% 100.0%  **IAINFKEDDELIAVRLTDGEEDILIGTAHASLIRFSEKALRPLGRTAAGVKGITLREGDEVVGLDVAHANSEDEVLVVTE**

33 SNUC5989 100.0% 100.0%  **IAINFKEDDELIAVRLTDGEEDILIGTAHASLIRFSEKALRPLGRTAAGVKGITLREGDEVVGLDVAHANSEDEVLVVTE**

34 SNUC3412 100.0% 100.0%  **IAINFKEDDELIAVRLTDGEEDILIGTAHASLIRFSEKALRPLGRTAAGVKGITLREGDEVVGLDVAHANSEDEVLVVTE**

35 OM08-17AT 100.0% 100.0%  **IAINFKEDDELIAVRLTDGEEDILIGTAHASLIRFSEKALRPLGRTAAGVKGITLREGDEVVGLDVAHANSEDEVLVVTE**

36 SWO 100.0% 100.0%  **IAINFKEDDELIAVRLTDGEEDILIGTAHASLIRFSEKALRPLGRTAAGVKGITLREGDEVVGLDVAHANSEDEVLVVTE**

37 VCU121 93.3% 100.0%  **IAINFKEDDELIAVRLTDGEEDILIGTAHASLIRFSEKALRPLGRTAAGVKGITLREGDEVVGLDVAHANSEDEVLVVTE**

consensus/100%  **IAINFKEDDELIAVRLTDGEEDILIGTAHASLIRFSEKALRPLGRTAAGVKGITLREGDEVVGLDVAHANSEDEVLVVTE**

consensus/90%  **IAINFKEDDELIAVRLTDGEEDILIGTAHASLIRFSEKALRPLGRTAAGVKGITLREGDEVVGLDVAHANSEDEVLVVTE**

consensus/80%  **IAINFKEDDELIAVRLTDGEEDILIGTAHASLIRFSEKALRPLGRTAAGVKGITLREGDEVVGLDVAHANSEDEVLVVTE**

consensus/70%  **IAINFKEDDELIAVRLTDGEEDILIGTAHASLIRFSEKALRPLGRTAAGVKGITLREGDEVVGLDVAHANSEDEVLVVTE**

cov pid **721**  **. . : . . . . 8** **800**

1 29AM 100.0% 100.0%  **NGYGKRTPVADYRLSNRGGKGIKTATITERNGNIVCITTVTGEEDLMVVTNAGVIIRLDVHDISQNGRSAQGVRLMRLGD**

2 TRPF4 100.0% 100.0%  **NGYGKRTPVADYRLSNRGGKGIKTATITERNGNIVCITTVTGEEDLMVVTNAGVIIRLDVHDISQNGRSAQGVRLMRLGD**

3 P912 100.0% 100.0%  **NGYGKRTPVADYRLSNRGGKGIKTATITERNGNIVCITTVTGEEDLMVVTNAGVIIRLDVHDISQNGRSAQGVRLMRLGD**

4 NGS-ED-1001 93.3% 99.9%  **NGYGKRTPVADYRLSNRGGKGIKTATITERNGNIVCITTVTGEEDLMVVTNAGVIIRLDVHDISQNGRSAQGVRLMRLGD**

5 1DB1 100.0% 100.0%  **NGYGKRTPVADYRLSNRGGKGIKTATITERNGNIVCITTVTGEEDLMVVTNAGVIIRLDVHDISQNGRSAQGVRLMRLGD**

6 MGYG-HGUT-02301 100.0% 100.0%  **NGYGKRTPVADYRLSNRGGKGIKTATITERNGNIVCITTVTGEEDLMVVTNAGVIIRLDVHDISQNGRSAQGVRLMRLGD**

7 IIF4SW-P1 100.0% 100.0%  **NGYGKRTPVADYRLSNRGGKGIKTATITERNGNIVCITTVTGEEDLMVVTNAGVIIRLDVHDISQNGRSAQGVRLMRLGD**

8 19428wF1_P912 100.0% 100.0%  **NGYGKRTPVADYRLSNRGGKGIKTATITERNGNIVCITTVTGEEDLMVVTNAGVIIRLDVHDISQNGRSAQGVRLMRLGD**

9 R5992 100.0% 100.0%  **NGYGKRTPVADYRLSNRGGKGIKTATITERNGNIVCITTVTGEEDLMVVTNAGVIIRLDVHDISQNGRSAQGVRLMRLGD**

10 R5990 100.0% 100.0%  **NGYGKRTPVADYRLSNRGGKGIKTATITERNGNIVCITTVTGEEDLMVVTNAGVIIRLDVHDISQNGRSAQGVRLMRLGD**

11 R5991 100.0% 100.0%  **NGYGKRTPVADYRLSNRGGKGIKTATITERNGNIVCITTVTGEEDLMVVTNAGVIIRLDVHDISQNGRSAQGVRLMRLGD**

12 R6028 100.0% 100.0%  **NGYGKRTPVADYRLSNRGGKGIKTATITERNGNIVCITTVTGEEDLMVVTNAGVIIRLDVHDISQNGRSAQGVRLMRLGD**

13 R6027 100.0% 100.0%  **NGYGKRTPVADYRLSNRGGKGIKTATITERNGNIVCITTVTGEEDLMVVTNAGVIIRLDVHDISQNGRSAQGVRLMRLGD**

14 R6024 100.0% 100.0%  **NGYGKRTPVADYRLSNRGGKGIKTATITERNGNIVCITTVTGEEDLMVVTNAGVIIRLDVHDISQNGRSAQGVRLMRLGD**

15 L2_057 100.0% 100.0%  **NGYGKRTPVADYRLSNRGGKGIKTATITERNGNIVCITTVTGEEDLMVVTNAGVIIRLDVHDISQNGRSAQGVRLMRLGD**

16 Cap 100.0% 99.9%  **NGYGKRTPVADYRLSNRGGKGIKTATITERNGNIVCITTVTGEEDLMVVTNAGVIIRLDVHDISQNGRSAQGVRLMRLGD**

17 Ani-LG-057 100.0% 100.0%  **NGYGKRTPVADYRLSNRGGKGIKTATITERNGNIVCITTVTGEEDLMVVTNAGVIIRLDVHDISQNGRSAQGVRLMRLGD**

18 Cap9.2 100.0% 99.9%  **NGYGKRTPVADYRLSNRGGKGIKTATITERNGNIVCITTVTGEEDLMVVTNAGVIIRLDVHDISQNGRSAQGVRLMRLGD**

19 Cap10.1 100.0% 99.9%  **NGYGKRTPVADYRLSNRGGKGIKTATITERNGNIVCITTVTGEEDLMVVTNAGVIIRLDVHDISQNGRSAQGVRLMRLGD**

20 Cap9.1 100.0% 99.9%  **NGYGKRTPVADYRLSNRGGKGIKTATITERNGNIVCITTVTGEEDLMVVTNAGVIIRLDVHDISQNGRSAQGVRLMRLGD**

21 Cap10.2 100.0% 99.9%  **NGYGKRTPVADYRLSNRGGKGIKTATITERNGNIVCITTVTGEEDLMVVTNAGVIIRLDVHDISQNGRSAQGVRLMRLGD**

22 acrok 100.0% 99.9%  **NGYGKRTPVADYRLSNRGGKGIKTATITERNGNIVCITTVTGEEDLMVVTNAGVIIRLDVHDISQNGRSAQGVRLMRLGD**

23 acroj 100.0% 99.9%  **NGYGKRTPVADYRLSNRGGKGIKTATITERNGNIVCITTVTGEEDLMVVTNAGVIIRLDVHDISQNGRSAQGVRLMRLGD**

24 acror 100.0% 100.0%  **NGYGKRTPVADYRLSNRGGKGIKTATITERNGNIVCITTVTGEEDLMVVTNAGVIIRLDVHDISQNGRSAQGVRLMRLGD**

25 NJ6 100.0% 100.0%  **NGYGKRTPVADYRLSNRGGKGIKTATITERNGNIVCITTVTGEEDLMVVTNAGVIIRLDVHDISQNGRSAQGVRLMRLGD**

26 EYE_450 100.0% 100.0%  **NGYGKRTPVADYRLSNRGGKGIKTATITERNGNIVCITTVTGEEDLMVVTNAGVIIRLDVHDISQNGRSAQGVRLMRLGD**

27 EYE_117 100.0% 100.0%  **NGYGKRTPVADYRLSNRGGKGIKTATITERNGNIVCITTVTGEEDLMVVTNAGVIIRLDVHDISQNGRSAQGVRLMRLGD**

28 EYE_410 100.0% 100.0%  **NGYGKRTPVADYRLSNRGGKGIKTATITERNGNIVCITTVTGEEDLMVVTNAGVIIRLDVHDISQNGRSAQGVRLMRLGD**

29 EYE_411 100.0% 100.0%  **NGYGKRTPVADYRLSNRGGKGIKTATITERNGNIVCITTVTGEEDLMVVTNAGVIIRLDVHDISQNGRSAQGVRLMRLGD**

30 MERTA18 100.0% 100.0%  **NGYGKRTPVADYRLSNRGGKGIKTATITERNGNIVCITTVTGEEDLMVVTNAGVIIRLDVHDISQNGRSAQGVRLMRLGD**

31 FAIRING19B-1.2 100.0% 100.0%  **NGYGKRTPVADYRLSNRGGKGIKTATITERNGNIVCITTVTGEEDLMVVTNAGVIIRLDVHDISQNGRSAQGVRLMRLGD**

32 FDAARGOS_151 100.0% 100.0%  **NGYGKRTPVADYRLSNRGGKGIKTATITERNGNIVCITTVTGEEDLMVVTNAGVIIRLDVHDISQNGRSAQGVRLMRLGD**

33 SNUC5989 100.0% 100.0%  **NGYGKRTPVADYRLSNRGGKGIKTATITERNGNIVCITTVTGEEDLMVVTNAGVIIRLDVHDISQNGRSAQGVRLMRLGD**

34 SNUC3412 100.0% 100.0%  **NGYGKRTPVADYRLSNRGGKGIKTATITERNGNIVCITTVTGEEDLMVVTNAGVIIRLDVHDISQNGRSAQGVRLMRLGD**

35 OM08-17AT 100.0% 100.0%  **NGYGKRTPVADYRLSNRGGKGIKTATITERNGNIVCITTVTGEEDLMVVTNAGVIIRLDVHDISQNGRSAQGVRLMRLGD**

36 SWO 100.0% 100.0%  **NGYGKRTPVADYRLSNRGGKGIKTATITERNGNIVCITTVTGEEDLMVVTNAGVIIRLDVHDISQNGRSAQGVRLMRLGD**

37 VCU121 93.3% 100.0%  **NGYGKRTPVADYRLSNRGGKGIKTATITERNGNIVCITTVTGEEDLMVVTNAGVIIRLDVHDISQNGRSAQGVRLMRLGD**

consensus/100%  **NGYGKRTPVADYRLSNRGGKGIKTATITERNGNIVCITTVTGEEDLMVVTNAGVIIRLDVHDISQNGRSAQGVRLMRLGD**

consensus/90%  **NGYGKRTPVADYRLSNRGGKGIKTATITERNGNIVCITTVTGEEDLMVVTNAGVIIRLDVHDISQNGRSAQGVRLMRLGD**

consensus/80%  **NGYGKRTPVADYRLSNRGGKGIKTATITERNGNIVCITTVTGEEDLMVVTNAGVIIRLDVHDISQNGRSAQGVRLMRLGD**

consensus/70%  **NGYGKRTPVADYRLSNRGGKGIKTATITERNGNIVCITTVTGEEDLMVVTNAGVIIRLDVHDISQNGRSAQGVRLMRLGD**

cov pid **801**  **. . . . : . . .** **880**

1 29AM 100.0% 100.0%  **DQFVSTVAKVKQEDDADEAESDIESDNAQGESSNEEVIEGGAPGKAIQTEVTDEEQGSEDTQDGDERIEVRQDFMDRVNE**

2 TRPF4 100.0% 100.0%  **DQFVSTVAKVKQEDDADEAESDIESDNAQGESSNEEVIEGGAPGKAIQTEVTDEEQGSEDTQDGDERIEVRQDFMDRVNE**

3 P912 100.0% 100.0%  **DQFVSTVAKVKQEDDADEAESDIESDNAQGESSNEEVIEGGAPGKAIQTEVTDEEQGSEDTQDGDERIEVRQDFMDRVNE**

4 NGS-ED-1001 93.3% 99.9%  **DQFVSTVAKVKQEDDADEAESDIESDNAQGESSNEEVIEGGAPGKAIQTEVTDEEQGSEDTQDGDERIEVRQDFMDRVNE**

5 1DB1 100.0% 100.0%  **DQFVSTVAKVKQEDDADEAESDIESDNAQGESSNEEVIEGGAPGKAIQTEVTDEEQGSEDTQDGDERIEVRQDFMDRVNE**

6 MGYG-HGUT-02301 100.0% 100.0%  **DQFVSTVAKVKQEDDADEAESDIESDNAQGESSNEEVIEGGAPGKAIQTEVTDEEQGSEDTQDGDERIEVRQDFMDRVNE**

7 IIF4SW-P1 100.0% 100.0%  **DQFVSTVAKVKQEDDADEAESDIESDNAQGESSNEEVIEGGAPGKAIQTEVTDEEQGSEDTQDGDERIEVRQDFMDRVNE**

8 19428wF1_P912 100.0% 100.0%  **DQFVSTVAKVKQEDDADEAESDIESDNAQGESSNEEVIEGGAPGKAIQTEVTDEEQGSEDTQDGDERIEVRQDFMDRVNE**

9 R5992 100.0% 100.0%  **DQFVSTVAKVKQEDDADEAESDIESDNAQGESSNEEVIEGGAPGKAIQTEVTDEEQGSEDTQDGDERIEVRQDFMDRVNE**

10 R5990 100.0% 100.0%  **DQFVSTVAKVKQEDDADEAESDIESDNAQGESSNEEVIEGGAPGKAIQTEVTDEEQGSEDTQDGDERIEVRQDFMDRVNE**

11 R5991 100.0% 100.0%  **DQFVSTVAKVKQEDDADEAESDIESDNAQGESSNEEVIEGGAPGKAIQTEVTDEEQGSEDTQDGDERIEVRQDFMDRVNE**

12 R6028 100.0% 100.0%  **DQFVSTVAKVKQEDDADEAESDIESDNAQGESSNEEVIEGGAPGKAIQTEVTDEEQGSEDTQDGDERIEVRQDFMDRVNE**

13 R6027 100.0% 100.0%  **DQFVSTVAKVKQEDDADEAESDIESDNAQGESSNEEVIEGGAPGKAIQTEVTDEEQGSEDTQDGDERIEVRQDFMDRVNE**

14 R6024 100.0% 100.0%  **DQFVSTVAKVKQEDDADEAESDIESDNAQGESSNEEVIEGGAPGKAIQTEVTDEEQGSEDTQDGDERIEVRQDFMDRVNE**

15 L2_057 100.0% 100.0%  **DQFVSTVAKVKQEDDADEAESDIESDNAQGESSNEEVIEGGAPGKAIQTEVTDEEQGSEDTQDGDERIEVRQDFMDRVNE**

16 Cap 100.0% 99.9%  **DQFVSTVAKVKQEDDADEAESDIESDNAQGESSNEEVIEGGAPGKAIQTEVTDEEQGSEDTQDGDERIEVRQDFMDRVNE**

17 Ani-LG-057 100.0% 100.0%  **DQFVSTVAKVKQEDDADEAESDIESDNAQGESSNEEVIEGGAPGKAIQTEVTDEEQGSEDTQDGDERIEVRQDFMDRVNE**

18 Cap9.2 100.0% 99.9%  **DQFVSTVAKVKQEDDADEAESDIESDNAQGESSNEEVIEGGAPGKAIQTEVTDEEQGSEDTQDGDERIEVRQDFMDRVNE**

19 Cap10.1 100.0% 99.9%  **DQFVSTVAKVKQEDDADEAESDIESDNAQGESSNEEVIEGGAPGKAIQTEVTDEEQGSEDTQDGDERIEVRQDFMDRVNE**

20 Cap9.1 100.0% 99.9%  **DQFVSTVAKVKQEDDADEAESDIESDNAQGESSNEEVIEGGAPGKAIQTEVTDEEQGSEDTQDGDERIEVRQDFMDRVNE**

21 Cap10.2 100.0% 99.9%  **DQFVSTVAKVKQEDDADEAESDIESDNAQGESSNEEVIEGGAPGKAIQTEVTDEEQGSEDTQDGDERIEVRQDFMDRVNE**

22 acrok 100.0% 99.9%  **DQFVSTVAKVKQEDDADEAESDIESDNAQGESSNEEVIEGGAPGKAIQTEVTDEEQGSEDTQDGDERIEVRQDFMDRVNE**

23 acroj 100.0% 99.9%  **DQFVSTVAKVKQEDDADEAESDIESDNAQGESSNEEVIEGGAPGKAIQTEVTDEEQGSEDTQDGDERIEVRQDFMDRVNE**

24 acror 100.0% 100.0%  **DQFVSTVAKVKQEDDADEAESDIESDNAQGESSNEEVIEGGAPGKAIQTEVTDEEQGSEDTQDGDERIEVRQDFMDRVNE**

25 NJ6 100.0% 100.0%  **DQFVSTVAKVKQEDDADEAESDIESDNAQGESSNEEVIEGGAPGKAIQTEVTDEEQGSEDTQDGDERIEVRQDFMDRVNE**

26 EYE_450 100.0% 100.0%  **DQFVSTVAKVKQEDDADEAESDIESDNAQGESSNEEVIEGGAPGKAIQTEVTDEEQGSEDTQDGDERIEVRQDFMDRVNE**

27 EYE_117 100.0% 100.0%  **DQFVSTVAKVKQEDDADEAESDIESDNAQGESSNEEVIEGGAPGKAIQTEVTDEEQGSEDTQDGDERIEVRQDFMDRVNE**

28 EYE_410 100.0% 100.0%  **DQFVSTVAKVKQEDDADEAESDIESDNAQGESSNEEVIEGGAPGKAIQTEVTDEEQGSEDTQDGDERIEVRQDFMDRVNE**

29 EYE_411 100.0% 100.0%  **DQFVSTVAKVKQEDDADEAESDIESDNAQGESSNEEVIEGGAPGKAIQTEVTDEEQGSEDTQDGDERIEVRQDFMDRVNE**

30 MERTA18 100.0% 100.0%  **DQFVSTVAKVKQEDDADEAESDIESDNAQGESSNEEVIEGGAPGKAIQTEVTDEEQGSEDTQDGDERIEVRQDFMDRVNE**

31 FAIRING19B-1.2 100.0% 100.0%  **DQFVSTVAKVKQEDDADEAESDIESDNAQGESSNEEVIEGGAPGKAIQTEVTDEEQGSEDTQDGDERIEVRQDFMDRVNE**

32 FDAARGOS_151 100.0% 100.0%  **DQFVSTVAKVKQEDDADEAESDIESDNAQGESSNEEVIEGGAPGKAIQTEVTDEEQGSEDTQDGDERIEVRQDFMDRVNE**

33 SNUC5989 100.0% 100.0%  **DQFVSTVAKVKQEDDADEAESDIESDNAQGESSNEEVIEGGAPGKAIQTEVTDEEQGSEDTQDGDERIEVRQDFMDRVNE**

34 SNUC3412 100.0% 100.0%  **DQFVSTVAKVKQEDDADEAESDIESDNAQGESSNEEVIEGGAPGKAIQTEVTDEEQGSEDTQDGDERIEVRQDFMDRVNE**

35 OM08-17AT 100.0% 100.0%  **DQFVSTVAKVKQEDDADEAESDIESDNAQGESSNEEVIEGGAPGKAIQTEVTDEEQGSEDTQDGDERIEVRQDFMDRVNE**

36 SWO 100.0% 100.0%  **DQFVSTVAKVKQEDDADEAESDIESDNAQGESSNEEVIEGGAPGKAIQTEVTDEEQGSEDTQDGDERIEVRQDFMDRVNE**

37 VCU121 93.3% 100.0%  **DQFVSTVAKVKQEDDADEAESDIESDNAQGESSNEEVIEGGAPGKAIQTEVTDEEQGSEDTQDGDERIEVRQDFMDRVNE**

consensus/100%  **DQFVSTVAKVKQEDDADEAESDIESDNAQGESSNEEVIEGGAPGKAIQTEVTDEEQGSEDTQDGDERIEVRQDFMDRVNE**

consensus/90%  **DQFVSTVAKVKQEDDADEAESDIESDNAQGESSNEEVIEGGAPGKAIQTEVTDEEQGSEDTQDGDERIEVRQDFMDRVNE**

consensus/80%  **DQFVSTVAKVKQEDDADEAESDIESDNAQGESSNEEVIEGGAPGKAIQTEVTDEEQGSEDTQDGDERIEVRQDFMDRVNE**

consensus/70%  **DQFVSTVAKVKQEDDADEAESDIESDNAQGESSNEEVIEGGAPGKAIQTEVTDEEQGSEDTQDGDERIEVRQDFMDRVNE**

cov pid **881**  **. ]** **892**

1 29AM 100.0% 100.0%  **DIENDSEDNEDE**

2 TRPF4 100.0% 100.0%  **DIENDSEDNEDE**

3 P912 100.0% 100.0%  **DIENDSEDNEDE**

4 NGS-ED-1001 93.3% 99.9%  **DIENDSEDNEDE**

5 1DB1 100.0% 100.0%  **DIENDSEDNEDE**

6 MGYG-HGUT-02301 100.0% 100.0%  **DIENDSEDNEDE**

7 IIF4SW-P1 100.0% 100.0%  **DIENDSEDNEDE**

8 19428wF1_P912 100.0% 100.0%  **DIENDSEDNEDE**

9 R5992 100.0% 100.0%  **DIENDSEDNEDE**

10 R5990 100.0% 100.0%  **DIENDSEDNEDE**

11 R5991 100.0% 100.0%  **DIENDSEDNEDE**

12 R6028 100.0% 100.0%  **DIENDSEDNEDE**

13 R6027 100.0% 100.0%  **DIENDSEDNEDE**

14 R6024 100.0% 100.0%  **DIENDSEDNEDE**

15 L2_057 100.0% 100.0%  **DIENDSEDNEDE**

16 Cap 100.0% 99.9%  **DIENDSEDNEDE**

17 Ani-LG-057 100.0% 100.0%  **DIENDSEDNEDE**

18 Cap9.2 100.0% 99.9%  **DIENDSEDNEDE**

19 Cap10.1 100.0% 99.9%  **DIENDSEDNEDE**

20 Cap9.1 100.0% 99.9%  **DIENDSEDNEDE**

21 Cap10.2 100.0% 99.9%  **DIENDSEDNEDE**

22 acrok 100.0% 99.9%  **DIENDSEDNEDE**

23 acroj 100.0% 99.9%  **DIENDSEDNEDE**

24 acror 100.0% 100.0%  **DIENDSEDNEDE**

25 NJ6 100.0% 100.0%  **DIENDSEDNEDE**

26 EYE_450 100.0% 100.0%  **DIENDSEDNEDE**

27 EYE_117 100.0% 100.0%  **DIENDSEDNEDE**

28 EYE_410 100.0% 100.0%  **DIENDSEDNEDE**

29 EYE_411 100.0% 100.0%  **DIENDSEDNEDE**

30 MERTA18 100.0% 100.0%  **DIENDSEDNEDE**

31 FAIRING19B-1.2 100.0% 100.0%  **DIENDSEDNEDE**

32 FDAARGOS_151 100.0% 100.0%  **DIENDSEDNEDE**

33 SNUC5989 100.0% 100.0%  **DIENDSEDNEDE**

34 SNUC3412 100.0% 100.0%  **DIENDSEDNEDE**

35 OM08-17AT 100.0% 100.0%  **DIENDSEDNEDE**

36 SWO 100.0% 100.0%  **DIENDSEDNEDE**

37 VCU121 93.3% 100.0%  **DIENDSEDNEDE**

consensus/100%  **DIENDSEDNEDE**

consensus/90%  **DIENDSEDNEDE**

consensus/80%  **DIENDSEDNEDE**

consensus/70%  **DIENDSEDNEDE**

**Supplementary Figure 6.** Multiple sequence alignment of the predicted amino acid sequences of GyrA in *S. warneri* isolate 29AM compared to that of the closely related *S. warneri* genomes retrieved from the BV-BRC database. The alignment was visualized using MView version 1.63 hosted by the EMBL-EBI; cov, coverage; pid, percent identity.

cov pid  **1** **[ . . . . : . . .** **80**

1 29AM 100.0% 100.0%  **MVNTLSDVNNTDNYGAGQIQVLEGLEAVRKRPGMYIGSTSERGLHHLVWEIVDNSIDEALAGYADEINVTIEKDNWIKVT**

2 TRPF4 100.0% 100.0%  **MVNTLSDVNNTDNYGAGQIQVLEGLEAVRKRPGMYIGSTSERGLHHLVWEIVDNSIDEALAGYADEINVTIEKDNWIKVT**

3 P912 100.0% 100.0%  **MVNTLSDVNNTDNYGAGQIQVLEGLEAVRKRPGMYIGSTSERGLHHLVWEIVDNSIDEALAGYADEINVTIEKDNWIKVT**

4 NGS-ED-1001 100.0% 100.0%  **MVNTLSDVNNTDNYGAGQIQVLEGLEAVRKRPGMYIGSTSERGLHHLVWEIVDNSIDEALAGYADEINVTIEKDNWIKVT**

5 1DB1 100.0% 100.0%  **MVNTLSDVNNTDNYGAGQIQVLEGLEAVRKRPGMYIGSTSERGLHHLVWEIVDNSIDEALAGYADEINVTIEKDNWIKVT**

6 MGYG-HGUT-02301 100.0% 100.0%  **MVNTLSDVNNTDNYGAGQIQVLEGLEAVRKRPGMYIGSTSERGLHHLVWEIVDNSIDEALAGYADEINVTIEKDNWIKVT**

7 IIF4SW-P1 100.0% 100.0%  **MVNTLSDVNNTDNYGAGQIQVLEGLEAVRKRPGMYIGSTSERGLHHLVWEIVDNSIDEALAGYADEINVTIEKDNWIKVT**

8 19428wF1_P912 100.0% 100.0%  **MVNTLSDVNNTDNYGAGQIQVLEGLEAVRKRPGMYIGSTSERGLHHLVWEIVDNSIDEALAGYADEINVTIEKDNWIKVT**

9 R5992 100.0% 100.0%  **MVNTLSDVNNTDNYGAGQIQVLEGLEAVRKRPGMYIGSTSERGLHHLVWEIVDNSIDEALAGYADEINVTIEKDNWIKVT**

10 R5990 100.0% 100.0%  **MVNTLSDVNNTDNYGAGQIQVLEGLEAVRKRPGMYIGSTSERGLHHLVWEIVDNSIDEALAGYADEINVTIEKDNWIKVT**

11 R5991 100.0% 100.0%  **MVNTLSDVNNTDNYGAGQIQVLEGLEAVRKRPGMYIGSTSERGLHHLVWEIVDNSIDEALAGYADEINVTIEKDNWIKVT**

12 R6028 100.0% 100.0%  **MVNTLSDVNNTDNYGAGQIQVLEGLEAVRKRPGMYIGSTSERGLHHLVWEIVDNSIDEALAGYADEINVTIEKDNWIKVT**

13 R6027 100.0% 100.0%  **MVNTLSDVNNTDNYGAGQIQVLEGLEAVRKRPGMYIGSTSERGLHHLVWEIVDNSIDEALAGYADEINVTIEKDNWIKVT**

14 R6024 90.7% 100.0%  **------------------------------------------------------------AGYADEINVTIEKDNWIKVT**

15 L2_057 100.0% 100.0%  **MVNTLSDVNNTDNYGAGQIQVLEGLEAVRKRPGMYIGSTSERGLHHLVWEIVDNSIDEALAGYADEINVTIEKDNWIKVT**

16 Cap100.1 100.0% 100.0%  **MVNTLSDVNNTDNYGAGQIQVLEGLEAVRKRPGMYIGSTSERGLHHLVWEIVDNSIDEALAGYADEINVTIEKDNWIKVT**

17 Ani-LG-057 100.0% 100.0%  **MVNTLSDVNNTDNYGAGQIQVLEGLEAVRKRPGMYIGSTSERGLHHLVWEIVDNSIDEALAGYADEINVTIEKDNWIKVT**

18 Cap9.2 100.0% 100.0%  **MVNTLSDVNNTDNYGAGQIQVLEGLEAVRKRPGMYIGSTSERGLHHLVWEIVDNSIDEALAGYADEINVTIEKDNWIKVT**

19 Cap10.1 100.0% 100.0%  **MVNTLSDVNNTDNYGAGQIQVLEGLEAVRKRPGMYIGSTSERGLHHLVWEIVDNSIDEALAGYADEINVTIEKDNWIKVT**

20 Cap9.1 100.0% 100.0%  **MVNTLSDVNNTDNYGAGQIQVLEGLEAVRKRPGMYIGSTSERGLHHLVWEIVDNSIDEALAGYADEINVTIEKDNWIKVT**

21 Cap10.2 100.0% 100.0%  **MVNTLSDVNNTDNYGAGQIQVLEGLEAVRKRPGMYIGSTSERGLHHLVWEIVDNSIDEALAGYADEINVTIEKDNWIKVT**

22 acrok 100.0% 100.0%  **MVNTLSDVNNTDNYGAGQIQVLEGLEAVRKRPGMYIGSTSERGLHHLVWEIVDNSIDEALAGYADEINVTIEKDNWIKVT**

23 acroj 100.0% 100.0%  **MVNTLSDVNNTDNYGAGQIQVLEGLEAVRKRPGMYIGSTSERGLHHLVWEIVDNSIDEALAGYADEINVTIEKDNWIKVT**

24 acror 100.0% 100.0%  **MVNTLSDVNNTDNYGAGQIQVLEGLEAVRKRPGMYIGSTSERGLHHLVWEIVDNSIDEALAGYADEINVTIEKDNWIKVT**

25 NJ6 100.0% 100.0%  **MVNTLSDVNNTDNYGAGQIQVLEGLEAVRKRPGMYIGSTSERGLHHLVWEIVDNSIDEALAGYADEINVTIEKDNWIKVT**

26 EYE_450 100.0% 100.0%  **MVNTLSDVNNTDNYGAGQIQVLEGLEAVRKRPGMYIGSTSERGLHHLVWEIVDNSIDEALAGYADEINVTIEKDNWIKVT**

27 EYE_117 100.0% 100.0%  **MVNTLSDVNNTDNYGAGQIQVLEGLEAVRKRPGMYIGSTSERGLHHLVWEIVDNSIDEALAGYADEINVTIEKDNWIKVT**

28 EYE_410 100.0% 100.0%  **MVNTLSDVNNTDNYGAGQIQVLEGLEAVRKRPGMYIGSTSERGLHHLVWEIVDNSIDEALAGYADEINVTIEKDNWIKVT**

29 EYE_411 100.0% 100.0%  **MVNTLSDVNNTDNYGAGQIQVLEGLEAVRKRPGMYIGSTSERGLHHLVWEIVDNSIDEALAGYADEINVTIEKDNWIKVT**

30 MERTA18 100.0% 100.0%  **MVNTLSDVNNTDNYGAGQIQVLEGLEAVRKRPGMYIGSTSERGLHHLVWEIVDNSIDEALAGYADEINVTIEKDNWIKVT**

31 FAIRING19B-1.2 100.0% 100.0%  **MVNTLSDVNNTDNYGAGQIQVLEGLEAVRKRPGMYIGSTSERGLHHLVWEIVDNSIDEALAGYADEINVTIEKDNWIKVT**

32 FDAARGOS_151 90.7% 100.0%  **------------------------------------------------------------AGYADEINVTIEKDNWIKVT**

33 SNUC5989 100.0% 100.0%  **MVNTLSDVNNTDNYGAGQIQVLEGLEAVRKRPGMYIGSTSERGLHHLVWEIVDNSIDEALAGYADEINVTIEKDNWIKVT**

34 SNUC3412 100.0% 99.8%  **MVNTLSDVNNTDNYGAGQIQVLEGLEAVRKRPGMYIGSTSERGLHHLVWEIVDNSIDEALAGYADEINVTIEKDNWIKVT**

35 OM08-17AT 90.7% 100.0%  **------------------------------------------------------------AGYADEINVTIEKDNWIKVT**

36 SWO 100.0% 100.0%  **MVNTLSDVNNTDNYGAGQIQVLEGLEAVRKRPGMYIGSTSERGLHHLVWEIVDNSIDEALAGYADEINVTIEKDNWIKVT**

37 VCU121 99.4% 99.8%  **----MSDVNNTDNYGAGQIQVLEGLEAVRKRPGMYIGSTSERGLHHLVWEIVDNSIDEALAGYADEINVTIEKDNWIKVT**

consensus/100%  **............................................................AGYADEINVTIEKDNWIKVT**

consensus/90%  **....hSDVNNTDNYGAGQIQVLEGLEAVRKRPGMYIGSTSERGLHHLVWEIVDNSIDEALAGYADEINVTIEKDNWIKVT**

consensus/80%  **MVNTLSDVNNTDNYGAGQIQVLEGLEAVRKRPGMYIGSTSERGLHHLVWEIVDNSIDEALAGYADEINVTIEKDNWIKVT**

consensus/70%  **MVNTLSDVNNTDNYGAGQIQVLEGLEAVRKRPGMYIGSTSERGLHHLVWEIVDNSIDEALAGYADEINVTIEKDNWIKVT**

cov pid  **81**  **. 1 . . . . : .** **160**

1 29AM 100.0% 100.0%  **DNGRGIPVDIQEKMGRPAVEVILTVLHAGGKFGGGGYKVSGGLHGVGSSVVNALSEDLEVYVHRNDTIYHQAYKKGVPQF**

2 TRPF4 100.0% 100.0%  **DNGRGIPVDIQEKMGRPAVEVILTVLHAGGKFGGGGYKVSGGLHGVGSSVVNALSEDLEVYVHRNDTIYHQAYKKGVPQF**

3 P912 100.0% 100.0%  **DNGRGIPVDIQEKMGRPAVEVILTVLHAGGKFGGGGYKVSGGLHGVGSSVVNALSEDLEVYVHRNDTIYHQAYKKGVPQF**

4 NGS-ED-1001 100.0% 100.0%  **DNGRGIPVDIQEKMGRPAVEVILTVLHAGGKFGGGGYKVSGGLHGVGSSVVNALSEDLEVYVHRNDTIYHQAYKKGVPQF**

5 1DB1 100.0% 100.0%  **DNGRGIPVDIQEKMGRPAVEVILTVLHAGGKFGGGGYKVSGGLHGVGSSVVNALSEDLEVYVHRNDTIYHQAYKKGVPQF**

6 MGYG-HGUT-02301 100.0% 100.0%  **DNGRGIPVDIQEKMGRPAVEVILTVLHAGGKFGGGGYKVSGGLHGVGSSVVNALSEDLEVYVHRNDTIYHQAYKKGVPQF**

7 IIF4SW-P1 100.0% 100.0%  **DNGRGIPVDIQEKMGRPAVEVILTVLHAGGKFGGGGYKVSGGLHGVGSSVVNALSEDLEVYVHRNDTIYHQAYKKGVPQF**

8 19428wF1_P912 100.0% 100.0%  **DNGRGIPVDIQEKMGRPAVEVILTVLHAGGKFGGGGYKVSGGLHGVGSSVVNALSEDLEVYVHRNDTIYHQAYKKGVPQF**

9 R5992 100.0% 100.0%  **DNGRGIPVDIQEKMGRPAVEVILTVLHAGGKFGGGGYKVSGGLHGVGSSVVNALSEDLEVYVHRNDTIYHQAYKKGVPQF**

10 R5990 100.0% 100.0%  **DNGRGIPVDIQEKMGRPAVEVILTVLHAGGKFGGGGYKVSGGLHGVGSSVVNALSEDLEVYVHRNDTIYHQAYKKGVPQF**

11 R5991 100.0% 100.0%  **DNGRGIPVDIQEKMGRPAVEVILTVLHAGGKFGGGGYKVSGGLHGVGSSVVNALSEDLEVYVHRNDTIYHQAYKKGVPQF**

12 R6028 100.0% 100.0%  **DNGRGIPVDIQEKMGRPAVEVILTVLHAGGKFGGGGYKVSGGLHGVGSSVVNALSEDLEVYVHRNDTIYHQAYKKGVPQF**

13 R6027 100.0% 100.0%  **DNGRGIPVDIQEKMGRPAVEVILTVLHAGGKFGGGGYKVSGGLHGVGSSVVNALSEDLEVYVHRNDTIYHQAYKKGVPQF**

14 R6024 90.7% 100.0%  **DNGRGIPVDIQEKMGRPAVEVILTVLHAGGKFGGGGYKVSGGLHGVGSSVVNALSEDLEVYVHRNDTIYHQAYKKGVPQF**

15 L2_057 100.0% 100.0%  **DNGRGIPVDIQEKMGRPAVEVILTVLHAGGKFGGGGYKVSGGLHGVGSSVVNALSEDLEVYVHRNDTIYHQAYKKGVPQF**

16 Cap100.1 100.0% 100.0%  **DNGRGIPVDIQEKMGRPAVEVILTVLHAGGKFGGGGYKVSGGLHGVGSSVVNALSEDLEVYVHRNDTIYHQAYKKGVPQF**

17 Ani-LG-057 100.0% 100.0%  **DNGRGIPVDIQEKMGRPAVEVILTVLHAGGKFGGGGYKVSGGLHGVGSSVVNALSEDLEVYVHRNDTIYHQAYKKGVPQF**

18 Cap9.2 100.0% 100.0%  **DNGRGIPVDIQEKMGRPAVEVILTVLHAGGKFGGGGYKVSGGLHGVGSSVVNALSEDLEVYVHRNDTIYHQAYKKGVPQF**

19 Cap10.1 100.0% 100.0%  **DNGRGIPVDIQEKMGRPAVEVILTVLHAGGKFGGGGYKVSGGLHGVGSSVVNALSEDLEVYVHRNDTIYHQAYKKGVPQF**

20 Cap9.1 100.0% 100.0%  **DNGRGIPVDIQEKMGRPAVEVILTVLHAGGKFGGGGYKVSGGLHGVGSSVVNALSEDLEVYVHRNDTIYHQAYKKGVPQF**

21 Cap10.2 100.0% 100.0%  **DNGRGIPVDIQEKMGRPAVEVILTVLHAGGKFGGGGYKVSGGLHGVGSSVVNALSEDLEVYVHRNDTIYHQAYKKGVPQF**

22 acrok 100.0% 100.0%  **DNGRGIPVDIQEKMGRPAVEVILTVLHAGGKFGGGGYKVSGGLHGVGSSVVNALSEDLEVYVHRNDTIYHQAYKKGVPQF**

23 acroj 100.0% 100.0%  **DNGRGIPVDIQEKMGRPAVEVILTVLHAGGKFGGGGYKVSGGLHGVGSSVVNALSEDLEVYVHRNDTIYHQAYKKGVPQF**

24 acror 100.0% 100.0%  **DNGRGIPVDIQEKMGRPAVEVILTVLHAGGKFGGGGYKVSGGLHGVGSSVVNALSEDLEVYVHRNDTIYHQAYKKGVPQF**

25 NJ6 100.0% 100.0%  **DNGRGIPVDIQEKMGRPAVEVILTVLHAGGKFGGGGYKVSGGLHGVGSSVVNALSEDLEVYVHRNDTIYHQAYKKGVPQF**

26 EYE_450 100.0% 100.0%  **DNGRGIPVDIQEKMGRPAVEVILTVLHAGGKFGGGGYKVSGGLHGVGSSVVNALSEDLEVYVHRNDTIYHQAYKKGVPQF**

27 EYE_117 100.0% 100.0%  **DNGRGIPVDIQEKMGRPAVEVILTVLHAGGKFGGGGYKVSGGLHGVGSSVVNALSEDLEVYVHRNDTIYHQAYKKGVPQF**

28 EYE_410 100.0% 100.0%  **DNGRGIPVDIQEKMGRPAVEVILTVLHAGGKFGGGGYKVSGGLHGVGSSVVNALSEDLEVYVHRNDTIYHQAYKKGVPQF**

29 EYE_411 100.0% 100.0%  **DNGRGIPVDIQEKMGRPAVEVILTVLHAGGKFGGGGYKVSGGLHGVGSSVVNALSEDLEVYVHRNDTIYHQAYKKGVPQF**

30 MERTA18 100.0% 100.0%  **DNGRGIPVDIQEKMGRPAVEVILTVLHAGGKFGGGGYKVSGGLHGVGSSVVNALSEDLEVYVHRNDTIYHQAYKKGVPQF**

31 FAIRING19B-1.2 100.0% 100.0%  **DNGRGIPVDIQEKMGRPAVEVILTVLHAGGKFGGGGYKVSGGLHGVGSSVVNALSEDLEVYVHRNDTIYHQAYKKGVPQF**

32 FDAARGOS_151 90.7% 100.0%  **DNGRGIPVDIQEKMGRPAVEVILTVLHAGGKFGGGGYKVSGGLHGVGSSVVNALSEDLEVYVHRNDTIYHQAYKKGVPQF**

33 SNUC5989 100.0% 100.0%  **DNGRGIPVDIQEKMGRPAVEVILTVLHAGGKFGGGGYKVSGGLHGVGSSVVNALSEDLEVYVHRNDTIYHQAYKKGVPQF**

34 SNUC3412 100.0% 99.8%  **DNGRGIPVDIQEKMGRPAVEVILTVLHAGGKFGGGGYKVSGGLHGVGSSVVNALSEDLEVYVHLNDTIYHQAYKKGVPQF**

35 OM08-17AT 90.7% 100.0%  **DNGRGIPVDIQEKMGRPAVEVILTVLHAGGKFGGGGYKVSGGLHGVGSSVVNALSEDLEVYVHRNDTIYHQAYKKGVPQF**

36 SWO 100.0% 100.0%  **DNGRGIPVDIQEKMGRPAVEVILTVLHAGGKFGGGGYKVSGGLHGVGSSVVNALSEDLEVYVHRNDTIYHQAYKKGVPQF**

37 VCU121 99.4% 99.8%  **DNGRGIPVDIQEKMGRPAVEVILTVLHAGGKFGGGGYKVSGGLHGVGSSVVNALSEDLEVYVHRNDTIYHQAYKKGVPQF**

consensus/100%  **DNGRGIPVDIQEKMGRPAVEVILTVLHAGGKFGGGGYKVSGGLHGVGSSVVNALSEDLEVYVHhNDTIYHQAYKKGVPQF**

consensus/90%  **DNGRGIPVDIQEKMGRPAVEVILTVLHAGGKFGGGGYKVSGGLHGVGSSVVNALSEDLEVYVHRNDTIYHQAYKKGVPQF**

consensus/80%  **DNGRGIPVDIQEKMGRPAVEVILTVLHAGGKFGGGGYKVSGGLHGVGSSVVNALSEDLEVYVHRNDTIYHQAYKKGVPQF**

consensus/70%  **DNGRGIPVDIQEKMGRPAVEVILTVLHAGGKFGGGGYKVSGGLHGVGSSVVNALSEDLEVYVHRNDTIYHQAYKKGVPQF**

cov pid **161**  **. . . 2 . . . .** **240**

1 29AM 100.0% 100.0%  **DLKEIGSTDKTGTAIRFKADSSIFTETTVYNYETLQQRIRELAFLNKGIQITLTDERDEDDIRQDSYYYEGGIKSYVEML**

2 TRPF4 100.0% 100.0%  **DLKEIGSTDKTGTAIRFKADSSIFTETTVYNYETLQQRIRELAFLNKGIQITLTDERDEDDIRQDSYYYEGGIKSYVEML**

3 P912 100.0% 100.0%  **DLKEIGSTDKTGTAIRFKADSSIFTETTVYNYETLQQRIRELAFLNKGIQITLTDERDEDDIRQDSYYYEGGIKSYVEML**

4 NGS-ED-1001 100.0% 100.0%  **DLKEIGSTDKTGTAIRFKADSSIFTETTVYNYETLQQRIRELAFLNKGIQITLTDERDEDDIRQDSYYYEGGIKSYVEML**

5 1DB1 100.0% 100.0%  **DLKEIGSTDKTGTAIRFKADSSIFTETTVYNYETLQQRIRELAFLNKGIQITLTDERDEDDIRQDSYYYEGGIKSYVEML**

6 MGYG-HGUT-02301 100.0% 100.0%  **DLKEIGSTDKTGTAIRFKADSSIFTETTVYNYETLQQRIRELAFLNKGIQITLTDERDEDDIRQDSYYYEGGIKSYVEML**

7 IIF4SW-P1 100.0% 100.0%  **DLKEIGSTDKTGTAIRFKADSSIFTETTVYNYETLQQRIRELAFLNKGIQITLTDERDEDDIRQDSYYYEGGIKSYVEML**

8 19428wF1_P912 100.0% 100.0%  **DLKEIGSTDKTGTAIRFKADSSIFTETTVYNYETLQQRIRELAFLNKGIQITLTDERDEDDIRQDSYYYEGGIKSYVEML**

9 R5992 100.0% 100.0%  **DLKEIGSTDKTGTAIRFKADSSIFTETTVYNYETLQQRIRELAFLNKGIQITLTDERDEDDIRQDSYYYEGGIKSYVEML**

10 R5990 100.0% 100.0%  **DLKEIGSTDKTGTAIRFKADSSIFTETTVYNYETLQQRIRELAFLNKGIQITLTDERDEDDIRQDSYYYEGGIKSYVEML**

11 R5991 100.0% 100.0%  **DLKEIGSTDKTGTAIRFKADSSIFTETTVYNYETLQQRIRELAFLNKGIQITLTDERDEDDIRQDSYYYEGGIKSYVEML**

12 R6028 100.0% 100.0%  **DLKEIGSTDKTGTAIRFKADSSIFTETTVYNYETLQQRIRELAFLNKGIQITLTDERDEDDIRQDSYYYEGGIKSYVEML**

13 R6027 100.0% 100.0%  **DLKEIGSTDKTGTAIRFKADSSIFTETTVYNYETLQQRIRELAFLNKGIQITLTDERDEDDIRQDSYYYEGGIKSYVEML**

14 R6024 90.7% 100.0%  **DLKEIGSTDKTGTAIRFKADSSIFTETTVYNYETLQQRIRELAFLNKGIQITLTDERDEDDIRQDSYYYEGGIKSYVEML**

15 L2_057 100.0% 100.0%  **DLKEIGSTDKTGTAIRFKADSSIFTETTVYNYETLQQRIRELAFLNKGIQITLTDERDEDDIRQDSYYYEGGIKSYVEML**

16 Cap100.1 100.0% 100.0%  **DLKEIGSTDKTGTAIRFKADSSIFTETTVYNYETLQQRIRELAFLNKGIQITLTDERDEDDIRQDSYYYEGGIKSYVEML**

17 Ani-LG-057 100.0% 100.0%  **DLKEIGSTDKTGTAIRFKADSSIFTETTVYNYETLQQRIRELAFLNKGIQITLTDERDEDDIRQDSYYYEGGIKSYVEML**

18 Cap9.2 100.0% 100.0%  **DLKEIGSTDKTGTAIRFKADSSIFTETTVYNYETLQQRIRELAFLNKGIQITLTDERDEDDIRQDSYYYEGGIKSYVEML**

19 Cap10.1 100.0% 100.0%  **DLKEIGSTDKTGTAIRFKADSSIFTETTVYNYETLQQRIRELAFLNKGIQITLTDERDEDDIRQDSYYYEGGIKSYVEML**

20 Cap9.1 100.0% 100.0%  **DLKEIGSTDKTGTAIRFKADSSIFTETTVYNYETLQQRIRELAFLNKGIQITLTDERDEDDIRQDSYYYEGGIKSYVEML**

21 Cap10.2 100.0% 100.0%  **DLKEIGSTDKTGTAIRFKADSSIFTETTVYNYETLQQRIRELAFLNKGIQITLTDERDEDDIRQDSYYYEGGIKSYVEML**

22 acrok 100.0% 100.0%  **DLKEIGSTDKTGTAIRFKADSSIFTETTVYNYETLQQRIRELAFLNKGIQITLTDERDEDDIRQDSYYYEGGIKSYVEML**

23 acroj 100.0% 100.0%  **DLKEIGSTDKTGTAIRFKADSSIFTETTVYNYETLQQRIRELAFLNKGIQITLTDERDEDDIRQDSYYYEGGIKSYVEML**

24 acror 100.0% 100.0%  **DLKEIGSTDKTGTAIRFKADSSIFTETTVYNYETLQQRIRELAFLNKGIQITLTDERDEDDIRQDSYYYEGGIKSYVEML**

25 NJ6 100.0% 100.0%  **DLKEIGSTDKTGTAIRFKADSSIFTETTVYNYETLQQRIRELAFLNKGIQITLTDERDEDDIRQDSYYYEGGIKSYVEML**

26 EYE_450 100.0% 100.0%  **DLKEIGSTDKTGTAIRFKADSSIFTETTVYNYETLQQRIRELAFLNKGIQITLTDERDEDDIRQDSYYYEGGIKSYVEML**

27 EYE_117 100.0% 100.0%  **DLKEIGSTDKTGTAIRFKADSSIFTETTVYNYETLQQRIRELAFLNKGIQITLTDERDEDDIRQDSYYYEGGIKSYVEML**

28 EYE_410 100.0% 100.0%  **DLKEIGSTDKTGTAIRFKADSSIFTETTVYNYETLQQRIRELAFLNKGIQITLTDERDEDDIRQDSYYYEGGIKSYVEML**

29 EYE_411 100.0% 100.0%  **DLKEIGSTDKTGTAIRFKADSSIFTETTVYNYETLQQRIRELAFLNKGIQITLTDERDEDDIRQDSYYYEGGIKSYVEML**

30 MERTA18 100.0% 100.0%  **DLKEIGSTDKTGTAIRFKADSSIFTETTVYNYETLQQRIRELAFLNKGIQITLTDERDEDDIRQDSYYYEGGIKSYVEML**

31 FAIRING19B-1.2 100.0% 100.0%  **DLKEIGSTDKTGTAIRFKADSSIFTETTVYNYETLQQRIRELAFLNKGIQITLTDERDEDDIRQDSYYYEGGIKSYVEML**

32 FDAARGOS_151 90.7% 100.0%  **DLKEIGSTDKTGTAIRFKADSSIFTETTVYNYETLQQRIRELAFLNKGIQITLTDERDEDDIRQDSYYYEGGIKSYVEML**

33 SNUC5989 100.0% 100.0%  **DLKEIGSTDKTGTAIRFKADSSIFTETTVYNYETLQQRIRELAFLNKGIQITLTDERDEDDIRQDSYYYEGGIKSYVEML**

34 SNUC3412 100.0% 99.8%  **DLKEIGSTDKTGTAIRFKADSSIFTETTVYNYETLQQRIRELAFLNKGIQITLTDERDEDDIRQDSYYYEGGIKSYVEML**

35 OM08-17AT 90.7% 100.0%  **DLKEIGSTDKTGTAIRFKADSSIFTETTVYNYETLQQRIRELAFLNKGIQITLTDERDEDDIRQDSYYYEGGIKSYVEML**

36 SWO 100.0% 100.0%  **DLKEIGSTDKTGTAIRFKADSSIFTETTVYNYETLQQRIRELAFLNKGIQITLTDERDEDDIRQDSYYYEGGIKSYVEML**

37 VCU121 99.4% 99.8%  **DLKEIGSTDKTGTAIRFKADSSIFTETTVYNYETLQQRIRELAFLNKGIQITLTDERDEDDIRQDSYYYEGGIKSYVEML**

consensus/100%  **DLKEIGSTDKTGTAIRFKADSSIFTETTVYNYETLQQRIRELAFLNKGIQITLTDERDEDDIRQDSYYYEGGIKSYVEML**

consensus/90%  **DLKEIGSTDKTGTAIRFKADSSIFTETTVYNYETLQQRIRELAFLNKGIQITLTDERDEDDIRQDSYYYEGGIKSYVEML**

consensus/80%  **DLKEIGSTDKTGTAIRFKADSSIFTETTVYNYETLQQRIRELAFLNKGIQITLTDERDEDDIRQDSYYYEGGIKSYVEML**

consensus/70%  **DLKEIGSTDKTGTAIRFKADSSIFTETTVYNYETLQQRIRELAFLNKGIQITLTDERDEDDIRQDSYYYEGGIKSYVEML**

cov pid **241**  **: . . . . 3 . .** **320**

1 29AM 100.0% 100.0%  **NENKEPIHGEPIYIHQSKDDIEVEIAIQYNKGYSTNLLTYANNIHTYEGGTHEDGFKRALTRVLNSYGMNSKIIKEDKDR**

2 TRPF4 100.0% 100.0%  **NENKEPIHGEPIYIHQSKDDIEVEIAIQYNKGYSTNLLTYANNIHTYEGGTHEDGFKRALTRVLNSYGMNSKIIKEDKDR**

3 P912 100.0% 100.0%  **NENKEPIHGEPIYIHQSKDDIEVEIAIQYNKGYSTNLLTYANNIHTYEGGTHEDGFKRALTRVLNSYGMNSKIIKEDKDR**

4 NGS-ED-1001 100.0% 100.0%  **NENKEPIHGEPIYIHQSKDDIEVEIAIQYNKGYSTNLLTYANNIHTYEGGTHEDGFKRALTRVLNSYGMNSKIIKEDKDR**

5 1DB1 100.0% 100.0%  **NENKEPIHGEPIYIHQSKDDIEVEIAIQYNKGYSTNLLTYANNIHTYEGGTHEDGFKRALTRVLNSYGMNSKIIKEDKDR**

6 MGYG-HGUT-02301 100.0% 100.0%  **NENKEPIHGEPIYIHQSKDDIEVEIAIQYNKGYSTNLLTYANNIHTYEGGTHEDGFKRALTRVLNSYGMNSKIIKEDKDR**

7 IIF4SW-P1 100.0% 100.0%  **NENKEPIHGEPIYIHQSKDDIEVEIAIQYNKGYSTNLLTYANNIHTYEGGTHEDGFKRALTRVLNSYGMNSKIIKEDKDR**

8 19428wF1_P912 100.0% 100.0%  **NENKEPIHGEPIYIHQSKDDIEVEIAIQYNKGYSTNLLTYANNIHTYEGGTHEDGFKRALTRVLNSYGMNSKIIKEDKDR**

9 R5992 100.0% 100.0%  **NENKEPIHGEPIYIHQSKDDIEVEIAIQYNKGYSTNLLTYANNIHTYEGGTHEDGFKRALTRVLNSYGMNSKIIKEDKDR**

10 R5990 100.0% 100.0%  **NENKEPIHGEPIYIHQSKDDIEVEIAIQYNKGYSTNLLTYANNIHTYEGGTHEDGFKRALTRVLNSYGMNSKIIKEDKDR**

11 R5991 100.0% 100.0%  **NENKEPIHGEPIYIHQSKDDIEVEIAIQYNKGYSTNLLTYANNIHTYEGGTHEDGFKRALTRVLNSYGMNSKIIKEDKDR**

12 R6028 100.0% 100.0%  **NENKEPIHGEPIYIHQSKDDIEVEIAIQYNKGYSTNLLTYANNIHTYEGGTHEDGFKRALTRVLNSYGMNSKIIKEDKDR**

13 R6027 100.0% 100.0%  **NENKEPIHGEPIYIHQSKDDIEVEIAIQYNKGYSTNLLTYANNIHTYEGGTHEDGFKRALTRVLNSYGMNSKIIKEDKDR**

14 R6024 90.7% 100.0%  **NENKEPIHGEPIYIHQSKDDIEVEIAIQYNKGYSTNLLTYANNIHTYEGGTHEDGFKRALTRVLNSYGMNSKIIKEDKDR**

15 L2_057 100.0% 100.0%  **NENKEPIHGEPIYIHQSKDDIEVEIAIQYNKGYSTNLLTYANNIHTYEGGTHEDGFKRALTRVLNSYGMNSKIIKEDKDR**

16 Cap100.1 100.0% 100.0%  **NENKEPIHGEPIYIHQSKDDIEVEIAIQYNKGYSTNLLTYANNIHTYEGGTHEDGFKRALTRVLNSYGMNSKIIKEDKDR**

17 Ani-LG-057 100.0% 100.0%  **NENKEPIHGEPIYIHQSKDDIEVEIAIQYNKGYSTNLLTYANNIHTYEGGTHEDGFKRALTRVLNSYGMNSKIIKEDKDR**

18 Cap9.2 100.0% 100.0%  **NENKEPIHGEPIYIHQSKDDIEVEIAIQYNKGYSTNLLTYANNIHTYEGGTHEDGFKRALTRVLNSYGMNSKIIKEDKDR**

19 Cap10.1 100.0% 100.0%  **NENKEPIHGEPIYIHQSKDDIEVEIAIQYNKGYSTNLLTYANNIHTYEGGTHEDGFKRALTRVLNSYGMNSKIIKEDKDR**

20 Cap9.1 100.0% 100.0%  **NENKEPIHGEPIYIHQSKDDIEVEIAIQYNKGYSTNLLTYANNIHTYEGGTHEDGFKRALTRVLNSYGMNSKIIKEDKDR**

21 Cap10.2 100.0% 100.0%  **NENKEPIHGEPIYIHQSKDDIEVEIAIQYNKGYSTNLLTYANNIHTYEGGTHEDGFKRALTRVLNSYGMNSKIIKEDKDR**

22 acrok 100.0% 100.0%  **NENKEPIHGEPIYIHQSKDDIEVEIAIQYNKGYSTNLLTYANNIHTYEGGTHEDGFKRALTRVLNSYGMNSKIIKEDKDR**

23 acroj 100.0% 100.0%  **NENKEPIHGEPIYIHQSKDDIEVEIAIQYNKGYSTNLLTYANNIHTYEGGTHEDGFKRALTRVLNSYGMNSKIIKEDKDR**

24 acror 100.0% 100.0%  **NENKEPIHGEPIYIHQSKDDIEVEIAIQYNKGYSTNLLTYANNIHTYEGGTHEDGFKRALTRVLNSYGMNSKIIKEDKDR**

25 NJ6 100.0% 100.0%  **NENKEPIHGEPIYIHQSKDDIEVEIAIQYNKGYSTNLLTYANNIHTYEGGTHEDGFKRALTRVLNSYGMNSKIIKEDKDR**

26 EYE_450 100.0% 100.0%  **NENKEPIHGEPIYIHQSKDDIEVEIAIQYNKGYSTNLLTYANNIHTYEGGTHEDGFKRALTRVLNSYGMNSKIIKEDKDR**

27 EYE_117 100.0% 100.0%  **NENKEPIHGEPIYIHQSKDDIEVEIAIQYNKGYSTNLLTYANNIHTYEGGTHEDGFKRALTRVLNSYGMNSKIIKEDKDR**

28 EYE_410 100.0% 100.0%  **NENKEPIHGEPIYIHQSKDDIEVEIAIQYNKGYSTNLLTYANNIHTYEGGTHEDGFKRALTRVLNSYGMNSKIIKEDKDR**

29 EYE_411 100.0% 100.0%  **NENKEPIHGEPIYIHQSKDDIEVEIAIQYNKGYSTNLLTYANNIHTYEGGTHEDGFKRALTRVLNSYGMNSKIIKEDKDR**

30 MERTA18 100.0% 100.0%  **NENKEPIHGEPIYIHQSKDDIEVEIAIQYNKGYSTNLLTYANNIHTYEGGTHEDGFKRALTRVLNSYGMNSKIIKEDKDR**

31 FAIRING19B-1.2 100.0% 100.0%  **NENKEPIHGEPIYIHQSKDDIEVEIAIQYNKGYSTNLLTYANNIHTYEGGTHEDGFKRALTRVLNSYGMNSKIIKEDKDR**

32 FDAARGOS_151 90.7% 100.0%  **NENKEPIHGEPIYIHQSKDDIEVEIAIQYNKGYSTNLLTYANNIHTYEGGTHEDGFKRALTRVLNSYGMNSKIIKEDKDR**

33 SNUC5989 100.0% 100.0%  **NENKEPIHGEPIYIHQSKDDIEVEIAIQYNKGYSTNLLTYANNIHTYEGGTHEDGFKRALTRVLNSYGMNSKIIKEDKDR**

34 SNUC3412 100.0% 99.8%  **NENKEPIHGEPIYIHQSKDDIEVEIAIQYNKGYSTNLLTYANNIHTYEGGTHEDGFKRALTRVLNSYGMNSKIIKEDKDR**

35 OM08-17AT 90.7% 100.0%  **NENKEPIHGEPIYIHQSKDDIEVEIAIQYNKGYSTNLLTYANNIHTYEGGTHEDGFKRALTRVLNSYGMNSKIIKEDKDR**

36 SWO 100.0% 100.0%  **NENKEPIHGEPIYIHQSKDDIEVEIAIQYNKGYSTNLLTYANNIHTYEGGTHEDGFKRALTRVLNSYGMNSKIIKEDKDR**

37 VCU121 99.4% 99.8%  **NENKEPIHGEPIYIHQSKDDIEVEIAIQYNKGYSTNLLTYANNIHTYEGGTHEDGFKRALTRVLNSYGMNSKIIKEDKDR**

consensus/100%  **NENKEPIHGEPIYIHQSKDDIEVEIAIQYNKGYSTNLLTYANNIHTYEGGTHEDGFKRALTRVLNSYGMNSKIIKEDKDR**

consensus/90%  **NENKEPIHGEPIYIHQSKDDIEVEIAIQYNKGYSTNLLTYANNIHTYEGGTHEDGFKRALTRVLNSYGMNSKIIKEDKDR**

consensus/80%  **NENKEPIHGEPIYIHQSKDDIEVEIAIQYNKGYSTNLLTYANNIHTYEGGTHEDGFKRALTRVLNSYGMNSKIIKEDKDR**

consensus/70%  **NENKEPIHGEPIYIHQSKDDIEVEIAIQYNKGYSTNLLTYANNIHTYEGGTHEDGFKRALTRVLNSYGMNSKIIKEDKDR**

cov pid **321**  **. . : . . . . 4** **400**

1 29AM 100.0% 100.0%  **LSGEDTREGLTAIISIKHGDPQFEGQTKTKLGNSEVRQVVDKLFSEHFERFLYENPSVGRIIVEKGIMASRARVAAKKAR**

2 TRPF4 100.0% 100.0%  **LSGEDTREGLTAIISIKHGDPQFEGQTKTKLGNSEVRQVVDKLFSEHFERFLYENPSVGRIIVEKGIMASRARVAAKKAR**

3 P912 100.0% 100.0%  **LSGEDTREGLTAIISIKHGDPQFEGQTKTKLGNSEVRQVVDKLFSEHFERFLYENPSVGRIIVEKGIMASRARVAAKKAR**

4 NGS-ED-1001 100.0% 100.0%  **LSGEDTREGLTAIISIKHGDPQFEGQTKTKLGNSEVRQVVDKLFSEHFERFLYENPSVGRIIVEKGIMASRARVAAKKAR**

5 1DB1 100.0% 100.0%  **LSGEDTREGLTAIISIKHGDPQFEGQTKTKLGNSEVRQVVDKLFSEHFERFLYENPSVGRIIVEKGIMASRARVAAKKAR**

6 MGYG-HGUT-02301 100.0% 100.0%  **LSGEDTREGLTAIISIKHGDPQFEGQTKTKLGNSEVRQVVDKLFSEHFERFLYENPSVGRIIVEKGIMASRARVAAKKAR**

7 IIF4SW-P1 100.0% 100.0%  **LSGEDTREGLTAIISIKHGDPQFEGQTKTKLGNSEVRQVVDKLFSEHFERFLYENPSVGRIIVEKGIMASRARVAAKKAR**

8 19428wF1_P912 100.0% 100.0%  **LSGEDTREGLTAIISIKHGDPQFEGQTKTKLGNSEVRQVVDKLFSEHFERFLYENPSVGRIIVEKGIMASRARVAAKKAR**

9 R5992 100.0% 100.0%  **LSGEDTREGLTAIISIKHGDPQFEGQTKTKLGNSEVRQVVDKLFSEHFERFLYENPSVGRIIVEKGIMASRARVAAKKAR**

10 R5990 100.0% 100.0%  **LSGEDTREGLTAIISIKHGDPQFEGQTKTKLGNSEVRQVVDKLFSEHFERFLYENPSVGRIIVEKGIMASRARVAAKKAR**

11 R5991 100.0% 100.0%  **LSGEDTREGLTAIISIKHGDPQFEGQTKTKLGNSEVRQVVDKLFSEHFERFLYENPSVGRIIVEKGIMASRARVAAKKAR**

12 R6028 100.0% 100.0%  **LSGEDTREGLTAIISIKHGDPQFEGQTKTKLGNSEVRQVVDKLFSEHFERFLYENPSVGRIIVEKGIMASRARVAAKKAR**

13 R6027 100.0% 100.0%  **LSGEDTREGLTAIISIKHGDPQFEGQTKTKLGNSEVRQVVDKLFSEHFERFLYENPSVGRIIVEKGIMASRARVAAKKAR**

14 R6024 90.7% 100.0%  **LSGEDTREGLTAIISIKHGDPQFEGQTKTKLGNSEVRQVVDKLFSEHFERFLYENPSVGRIIVEKGIMASRARVAAKKAR**

15 L2_057 100.0% 100.0%  **LSGEDTREGLTAIISIKHGDPQFEGQTKTKLGNSEVRQVVDKLFSEHFERFLYENPSVGRIIVEKGIMASRARVAAKKAR**

16 Cap100.1 100.0% 100.0%  **LSGEDTREGLTAIISIKHGDPQFEGQTKTKLGNSEVRQVVDKLFSEHFERFLYENPSVGRIIVEKGIMASRARVAAKKAR**

17 Ani-LG-057 100.0% 100.0%  **LSGEDTREGLTAIISIKHGDPQFEGQTKTKLGNSEVRQVVDKLFSEHFERFLYENPSVGRIIVEKGIMASRARVAAKKAR**

18 Cap9.2 100.0% 100.0%  **LSGEDTREGLTAIISIKHGDPQFEGQTKTKLGNSEVRQVVDKLFSEHFERFLYENPSVGRIIVEKGIMASRARVAAKKAR**

19 Cap10.1 100.0% 100.0%  **LSGEDTREGLTAIISIKHGDPQFEGQTKTKLGNSEVRQVVDKLFSEHFERFLYENPSVGRIIVEKGIMASRARVAAKKAR**

20 Cap9.1 100.0% 100.0%  **LSGEDTREGLTAIISIKHGDPQFEGQTKTKLGNSEVRQVVDKLFSEHFERFLYENPSVGRIIVEKGIMASRARVAAKKAR**

21 Cap10.2 100.0% 100.0%  **LSGEDTREGLTAIISIKHGDPQFEGQTKTKLGNSEVRQVVDKLFSEHFERFLYENPSVGRIIVEKGIMASRARVAAKKAR**

22 acrok 100.0% 100.0%  **LSGEDTREGLTAIISIKHGDPQFEGQTKTKLGNSEVRQVVDKLFSEHFERFLYENPSVGRIIVEKGIMASRARVAAKKAR**

23 acroj 100.0% 100.0%  **LSGEDTREGLTAIISIKHGDPQFEGQTKTKLGNSEVRQVVDKLFSEHFERFLYENPSVGRIIVEKGIMASRARVAAKKAR**

24 acror 100.0% 100.0%  **LSGEDTREGLTAIISIKHGDPQFEGQTKTKLGNSEVRQVVDKLFSEHFERFLYENPSVGRIIVEKGIMASRARVAAKKAR**

25 NJ6 100.0% 100.0%  **LSGEDTREGLTAIISIKHGDPQFEGQTKTKLGNSEVRQVVDKLFSEHFERFLYENPSVGRIIVEKGIMASRARVAAKKAR**

26 EYE_450 100.0% 100.0%  **LSGEDTREGLTAIISIKHGDPQFEGQTKTKLGNSEVRQVVDKLFSEHFERFLYENPSVGRIIVEKGIMASRARVAAKKAR**

27 EYE_117 100.0% 100.0%  **LSGEDTREGLTAIISIKHGDPQFEGQTKTKLGNSEVRQVVDKLFSEHFERFLYENPSVGRIIVEKGIMASRARVAAKKAR**

28 EYE_410 100.0% 100.0%  **LSGEDTREGLTAIISIKHGDPQFEGQTKTKLGNSEVRQVVDKLFSEHFERFLYENPSVGRIIVEKGIMASRARVAAKKAR**

29 EYE_411 100.0% 100.0%  **LSGEDTREGLTAIISIKHGDPQFEGQTKTKLGNSEVRQVVDKLFSEHFERFLYENPSVGRIIVEKGIMASRARVAAKKAR**

30 MERTA18 100.0% 100.0%  **LSGEDTREGLTAIISIKHGDPQFEGQTKTKLGNSEVRQVVDKLFSEHFERFLYENPSVGRIIVEKGIMASRARVAAKKAR**

31 FAIRING19B-1.2 100.0% 100.0%  **LSGEDTREGLTAIISIKHGDPQFEGQTKTKLGNSEVRQVVDKLFSEHFERFLYENPSVGRIIVEKGIMASRARVAAKKAR**

32 FDAARGOS_151 90.7% 100.0%  **LSGEDTREGLTAIISIKHGDPQFEGQTKTKLGNSEVRQVVDKLFSEHFERFLYENPSVGRIIVEKGIMASRARVAAKKAR**

33 SNUC5989 100.0% 100.0%  **LSGEDTREGLTAIISIKHGDPQFEGQTKTKLGNSEVRQVVDKLFSEHFERFLYENPSVGRIIVEKGIMASRARVAAKKAR**

34 SNUC3412 100.0% 99.8%  **LSGEDTREGLTAIISIKHGDPQFEGQTKTKLGNSEVRQVVDKLFSEHFERFLYENPSVGRIIVEKGIMASRARVAAKKAR**

35 OM08-17AT 90.7% 100.0%  **LSGEDTREGLTAIISIKHGDPQFEGQTKTKLGNSEVRQVVDKLFSEHFERFLYENPSVGRIIVEKGIMASRARVAAKKAR**

36 SWO 100.0% 100.0%  **LSGEDTREGLTAIISIKHGDPQFEGQTKTKLGNSEVRQVVDKLFSEHFERFLYENPSVGRIIVEKGIMASRARVAAKKAR**

37 VCU121 99.4% 99.8%  **LSGEDTREGLTAIISIKHGDPQFEGQTKTKLGNSEVRQVVDKLFSEHFERFLYENPSVGRIIVEKGIMASRARVAAKKAR**

consensus/100%  **LSGEDTREGLTAIISIKHGDPQFEGQTKTKLGNSEVRQVVDKLFSEHFERFLYENPSVGRIIVEKGIMASRARVAAKKAR**

consensus/90%  **LSGEDTREGLTAIISIKHGDPQFEGQTKTKLGNSEVRQVVDKLFSEHFERFLYENPSVGRIIVEKGIMASRARVAAKKAR**

consensus/80%  **LSGEDTREGLTAIISIKHGDPQFEGQTKTKLGNSEVRQVVDKLFSEHFERFLYENPSVGRIIVEKGIMASRARVAAKKAR**

consensus/70%  **LSGEDTREGLTAIISIKHGDPQFEGQTKTKLGNSEVRQVVDKLFSEHFERFLYENPSVGRIIVEKGIMASRARVAAKKAR**

cov pid **401**  **. . . . : . . .** **480**

1 29AM 100.0% 100.0%  **EVTRRKSALEISSLPGKLADCSSKNPEESEIFLVEGDSAGGSTKSGRDSSTQAILPLRGKILNVEKARLDRILNNNEIRS**

2 TRPF4 100.0% 100.0%  **EVTRRKSALEISSLPGKLADCSSKNPEESEIFLVEGDSAGGSTKSGRDSSTQAILPLRGKILNVEKARLDRILNNNEIRS**

3 P912 100.0% 100.0%  **EVTRRKSALEISSLPGKLADCSSKNPEESEIFLVEGDSAGGSTKSGRDSSTQAILPLRGKILNVEKARLDRILNNNEIRS**

4 NGS-ED-1001 100.0% 100.0%  **EVTRRKSALEISSLPGKLADCSSKNPEESEIFLVEGDSAGGSTKSGRDSSTQAILPLRGKILNVEKARLDRILNNNEIRS**

5 1DB1 100.0% 100.0%  **EVTRRKSALEISSLPGKLADCSSKNPEESEIFLVEGDSAGGSTKSGRDSSTQAILPLRGKILNVEKARLDRILNNNEIRS**

6 MGYG-HGUT-02301 100.0% 100.0%  **EVTRRKSALEISSLPGKLADCSSKNPEESEIFLVEGDSAGGSTKSGRDSSTQAILPLRGKILNVEKARLDRILNNNEIRS**

7 IIF4SW-P1 100.0% 100.0%  **EVTRRKSALEISSLPGKLADCSSKNPEESEIFLVEGDSAGGSTKSGRDSSTQAILPLRGKILNVEKARLDRILNNNEIRS**

8 19428wF1_P912 100.0% 100.0%  **EVTRRKSALEISSLPGKLADCSSKNPEESEIFLVEGDSAGGSTKSGRDSSTQAILPLRGKILNVEKARLDRILNNNEIRS**

9 R5992 100.0% 100.0%  **EVTRRKSALEISSLPGKLADCSSKNPEESEIFLVEGDSAGGSTKSGRDSSTQAILPLRGKILNVEKARLDRILNNNEIRS**

10 R5990 100.0% 100.0%  **EVTRRKSALEISSLPGKLADCSSKNPEESEIFLVEGDSAGGSTKSGRDSSTQAILPLRGKILNVEKARLDRILNNNEIRS**

11 R5991 100.0% 100.0%  **EVTRRKSALEISSLPGKLADCSSKNPEESEIFLVEGDSAGGSTKSGRDSSTQAILPLRGKILNVEKARLDRILNNNEIRS**

12 R6028 100.0% 100.0%  **EVTRRKSALEISSLPGKLADCSSKNPEESEIFLVEGDSAGGSTKSGRDSSTQAILPLRGKILNVEKARLDRILNNNEIRS**

13 R6027 100.0% 100.0%  **EVTRRKSALEISSLPGKLADCSSKNPEESEIFLVEGDSAGGSTKSGRDSSTQAILPLRGKILNVEKARLDRILNNNEIRS**

14 R6024 90.7% 100.0%  **EVTRRKSALEISSLPGKLADCSSKNPEESEIFLVEGDSAGGSTKSGRDSSTQAILPLRGKILNVEKARLDRILNNNEIRS**

15 L2_057 100.0% 100.0%  **EVTRRKSALEISSLPGKLADCSSKNPEESEIFLVEGDSAGGSTKSGRDSSTQAILPLRGKILNVEKARLDRILNNNEIRS**

16 Cap100.1 100.0% 100.0%  **EVTRRKSALEISSLPGKLADCSSKNPEESEIFLVEGDSAGGSTKSGRDSSTQAILPLRGKILNVEKARLDRILNNNEIRS**

17 Ani-LG-057 100.0% 100.0%  **EVTRRKSALEISSLPGKLADCSSKNPEESEIFLVEGDSAGGSTKSGRDSSTQAILPLRGKILNVEKARLDRILNNNEIRS**

18 Cap9.2 100.0% 100.0%  **EVTRRKSALEISSLPGKLADCSSKNPEESEIFLVEGDSAGGSTKSGRDSSTQAILPLRGKILNVEKARLDRILNNNEIRS**

19 Cap10.1 100.0% 100.0%  **EVTRRKSALEISSLPGKLADCSSKNPEESEIFLVEGDSAGGSTKSGRDSSTQAILPLRGKILNVEKARLDRILNNNEIRS**

20 Cap9.1 100.0% 100.0%  **EVTRRKSALEISSLPGKLADCSSKNPEESEIFLVEGDSAGGSTKSGRDSSTQAILPLRGKILNVEKARLDRILNNNEIRS**

21 Cap10.2 100.0% 100.0%  **EVTRRKSALEISSLPGKLADCSSKNPEESEIFLVEGDSAGGSTKSGRDSSTQAILPLRGKILNVEKARLDRILNNNEIRS**

22 acrok 100.0% 100.0%  **EVTRRKSALEISSLPGKLADCSSKNPEESEIFLVEGDSAGGSTKSGRDSSTQAILPLRGKILNVEKARLDRILNNNEIRS**

23 acroj 100.0% 100.0%  **EVTRRKSALEISSLPGKLADCSSKNPEESEIFLVEGDSAGGSTKSGRDSSTQAILPLRGKILNVEKARLDRILNNNEIRS**

24 acror 100.0% 100.0%  **EVTRRKSALEISSLPGKLADCSSKNPEESEIFLVEGDSAGGSTKSGRDSSTQAILPLRGKILNVEKARLDRILNNNEIRS**

25 NJ6 100.0% 100.0%  **EVTRRKSALEISSLPGKLADCSSKNPEESEIFLVEGDSAGGSTKSGRDSSTQAILPLRGKILNVEKARLDRILNNNEIRS**

26 EYE_450 100.0% 100.0%  **EVTRRKSALEISSLPGKLADCSSKNPEESEIFLVEGDSAGGSTKSGRDSSTQAILPLRGKILNVEKARLDRILNNNEIRS**

27 EYE_117 100.0% 100.0%  **EVTRRKSALEISSLPGKLADCSSKNPEESEIFLVEGDSAGGSTKSGRDSSTQAILPLRGKILNVEKARLDRILNNNEIRS**

28 EYE_410 100.0% 100.0%  **EVTRRKSALEISSLPGKLADCSSKNPEESEIFLVEGDSAGGSTKSGRDSSTQAILPLRGKILNVEKARLDRILNNNEIRS**

29 EYE_411 100.0% 100.0%  **EVTRRKSALEISSLPGKLADCSSKNPEESEIFLVEGDSAGGSTKSGRDSSTQAILPLRGKILNVEKARLDRILNNNEIRS**

30 MERTA18 100.0% 100.0%  **EVTRRKSALEISSLPGKLADCSSKNPEESEIFLVEGDSAGGSTKSGRDSSTQAILPLRGKILNVEKARLDRILNNNEIRS**

31 FAIRING19B-1.2 100.0% 100.0%  **EVTRRKSALEISSLPGKLADCSSKNPEESEIFLVEGDSAGGSTKSGRDSSTQAILPLRGKILNVEKARLDRILNNNEIRS**

32 FDAARGOS_151 90.7% 100.0%  **EVTRRKSALEISSLPGKLADCSSKNPEESEIFLVEGDSAGGSTKSGRDSSTQAILPLRGKILNVEKARLDRILNNNEIRS**

33 SNUC5989 100.0% 100.0%  **EVTRRKSALEISSLPGKLADCSSKNPEESEIFLVEGDSAGGSTKSGRDSSTQAILPLRGKILNVEKARLDRILNNNEIRS**

34 SNUC3412 100.0% 99.8%  **EVTRRKSALEISSLPGKLADCSSKNPEESEIFLVEGDSAGGSTKSGRDSSTQAILPLRGKILNVEKARLDRILNNNEIRS**

35 OM08-17AT 90.7% 100.0%  **EVTRRKSALEISSLPGKLADCSSKNPEESEIFLVEGDSAGGSTKSGRDSSTQAILPLRGKILNVEKARLDRILNNNEIRS**

36 SWO 100.0% 100.0%  **EVTRRKSALEISSLPGKLADCSSKNPEESEIFLVEGDSAGGSTKSGRDSSTQAILPLRGKILNVEKARLDRILNNNEIRS**

37 VCU121 99.4% 99.8%  **EVTRRKSALEISSLPGKLADCSSKNPEESEIFLVEGDSAGGSTKSGRDSSTQAILPLRGKILNVEKARLDRILNNNEIRS**

consensus/100%  **EVTRRKSALEISSLPGKLADCSSKNPEESEIFLVEGDSAGGSTKSGRDSSTQAILPLRGKILNVEKARLDRILNNNEIRS**

consensus/90%  **EVTRRKSALEISSLPGKLADCSSKNPEESEIFLVEGDSAGGSTKSGRDSSTQAILPLRGKILNVEKARLDRILNNNEIRS**

consensus/80%  **EVTRRKSALEISSLPGKLADCSSKNPEESEIFLVEGDSAGGSTKSGRDSSTQAILPLRGKILNVEKARLDRILNNNEIRS**

consensus/70%  **EVTRRKSALEISSLPGKLADCSSKNPEESEIFLVEGDSAGGSTKSGRDSSTQAILPLRGKILNVEKARLDRILNNNEIRS**

cov pid **481**  **. 5 . . . . : .** **560**

1 29AM 100.0% 100.0%  **MITAFGTGIGGEFDISKARYHKIVIMTDADVDGAHIRTLLLTFFYRFMRPLIEAGYVYIAQPPLYKLTQGKQKYYVFNDR**

2 TRPF4 100.0% 100.0%  **MITAFGTGIGGEFDISKARYHKIVIMTDADVDGAHIRTLLLTFFYRFMRPLIEAGYVYIAQPPLYKLTQGKQKYYVFNDR**

3 P912 100.0% 100.0%  **MITAFGTGIGGEFDISKARYHKIVIMTDADVDGAHIRTLLLTFFYRFMRPLIEAGYVYIAQPPLYKLTQGKQKYYVFNDR**

4 NGS-ED-1001 100.0% 100.0%  **MITAFGTGIGGEFDISKARYHKIVIMTDADVDGAHIRTLLLTFFYRFMRPLIEAGYVYIAQPPLYKLTQGKQKYYVFNDR**

5 1DB1 100.0% 100.0%  **MITAFGTGIGGEFDISKARYHKIVIMTDADVDGAHIRTLLLTFFYRFMRPLIEAGYVYIAQPPLYKLTQGKQKYYVFNDR**

6 MGYG-HGUT-02301 100.0% 100.0%  **MITAFGTGIGGEFDISKARYHKIVIMTDADVDGAHIRTLLLTFFYRFMRPLIEAGYVYIAQPPLYKLTQGKQKYYVFNDR**

7 IIF4SW-P1 100.0% 100.0%  **MITAFGTGIGGEFDISKARYHKIVIMTDADVDGAHIRTLLLTFFYRFMRPLIEAGYVYIAQPPLYKLTQGKQKYYVFNDR**

8 19428wF1_P912 100.0% 100.0%  **MITAFGTGIGGEFDISKARYHKIVIMTDADVDGAHIRTLLLTFFYRFMRPLIEAGYVYIAQPPLYKLTQGKQKYYVFNDR**

9 R5992 100.0% 100.0%  **MITAFGTGIGGEFDISKARYHKIVIMTDADVDGAHIRTLLLTFFYRFMRPLIEAGYVYIAQPPLYKLTQGKQKYYVFNDR**

10 R5990 100.0% 100.0%  **MITAFGTGIGGEFDISKARYHKIVIMTDADVDGAHIRTLLLTFFYRFMRPLIEAGYVYIAQPPLYKLTQGKQKYYVFNDR**

11 R5991 100.0% 100.0%  **MITAFGTGIGGEFDISKARYHKIVIMTDADVDGAHIRTLLLTFFYRFMRPLIEAGYVYIAQPPLYKLTQGKQKYYVFNDR**

12 R6028 100.0% 100.0%  **MITAFGTGIGGEFDISKARYHKIVIMTDADVDGAHIRTLLLTFFYRFMRPLIEAGYVYIAQPPLYKLTQGKQKYYVFNDR**

13 R6027 100.0% 100.0%  **MITAFGTGIGGEFDISKARYHKIVIMTDADVDGAHIRTLLLTFFYRFMRPLIEAGYVYIAQPPLYKLTQGKQKYYVFNDR**

14 R6024 90.7% 100.0%  **MITAFGTGIGGEFDISKARYHKIVIMTDADVDGAHIRTLLLTFFYRFMRPLIEAGYVYIAQPPLYKLTQGKQKYYVFNDR**

15 L2_057 100.0% 100.0%  **MITAFGTGIGGEFDISKARYHKIVIMTDADVDGAHIRTLLLTFFYRFMRPLIEAGYVYIAQPPLYKLTQGKQKYYVFNDR**

16 Cap100.1 100.0% 100.0%  **MITAFGTGIGGEFDISKARYHKIVIMTDADVDGAHIRTLLLTFFYRFMRPLIEAGYVYIAQPPLYKLTQGKQKYYVFNDR**

17 Ani-LG-057 100.0% 100.0%  **MITAFGTGIGGEFDISKARYHKIVIMTDADVDGAHIRTLLLTFFYRFMRPLIEAGYVYIAQPPLYKLTQGKQKYYVFNDR**

18 Cap9.2 100.0% 100.0%  **MITAFGTGIGGEFDISKARYHKIVIMTDADVDGAHIRTLLLTFFYRFMRPLIEAGYVYIAQPPLYKLTQGKQKYYVFNDR**

19 Cap10.1 100.0% 100.0%  **MITAFGTGIGGEFDISKARYHKIVIMTDADVDGAHIRTLLLTFFYRFMRPLIEAGYVYIAQPPLYKLTQGKQKYYVFNDR**

20 Cap9.1 100.0% 100.0%  **MITAFGTGIGGEFDISKARYHKIVIMTDADVDGAHIRTLLLTFFYRFMRPLIEAGYVYIAQPPLYKLTQGKQKYYVFNDR**

21 Cap10.2 100.0% 100.0%  **MITAFGTGIGGEFDISKARYHKIVIMTDADVDGAHIRTLLLTFFYRFMRPLIEAGYVYIAQPPLYKLTQGKQKYYVFNDR**

22 acrok 100.0% 100.0%  **MITAFGTGIGGEFDISKARYHKIVIMTDADVDGAHIRTLLLTFFYRFMRPLIEAGYVYIAQPPLYKLTQGKQKYYVFNDR**

23 acroj 100.0% 100.0%  **MITAFGTGIGGEFDISKARYHKIVIMTDADVDGAHIRTLLLTFFYRFMRPLIEAGYVYIAQPPLYKLTQGKQKYYVFNDR**

24 acror 100.0% 100.0%  **MITAFGTGIGGEFDISKARYHKIVIMTDADVDGAHIRTLLLTFFYRFMRPLIEAGYVYIAQPPLYKLTQGKQKYYVFNDR**

25 NJ6 100.0% 100.0%  **MITAFGTGIGGEFDISKARYHKIVIMTDADVDGAHIRTLLLTFFYRFMRPLIEAGYVYIAQPPLYKLTQGKQKYYVFNDR**

26 EYE_450 100.0% 100.0%  **MITAFGTGIGGEFDISKARYHKIVIMTDADVDGAHIRTLLLTFFYRFMRPLIEAGYVYIAQPPLYKLTQGKQKYYVFNDR**

27 EYE_117 100.0% 100.0%  **MITAFGTGIGGEFDISKARYHKIVIMTDADVDGAHIRTLLLTFFYRFMRPLIEAGYVYIAQPPLYKLTQGKQKYYVFNDR**

28 EYE_410 100.0% 100.0%  **MITAFGTGIGGEFDISKARYHKIVIMTDADVDGAHIRTLLLTFFYRFMRPLIEAGYVYIAQPPLYKLTQGKQKYYVFNDR**

29 EYE_411 100.0% 100.0%  **MITAFGTGIGGEFDISKARYHKIVIMTDADVDGAHIRTLLLTFFYRFMRPLIEAGYVYIAQPPLYKLTQGKQKYYVFNDR**

30 MERTA18 100.0% 100.0%  **MITAFGTGIGGEFDISKARYHKIVIMTDADVDGAHIRTLLLTFFYRFMRPLIEAGYVYIAQPPLYKLTQGKQKYYVFNDR**

31 FAIRING19B-1.2 100.0% 100.0%  **MITAFGTGIGGEFDISKARYHKIVIMTDADVDGAHIRTLLLTFFYRFMRPLIEAGYVYIAQPPLYKLTQGKQKYYVFNDR**

32 FDAARGOS_151 90.7% 100.0%  **MITAFGTGIGGEFDISKARYHKIVIMTDADVDGAHIRTLLLTFFYRFMRPLIEAGYVYIAQPPLYKLTQGKQKYYVFNDR**

33 SNUC5989 100.0% 100.0%  **MITAFGTGIGGEFDISKARYHKIVIMTDADVDGAHIRTLLLTFFYRFMRPLIEAGYVYIAQPPLYKLTQGKQKYYVFNDR**

34 SNUC3412 100.0% 99.8%  **MITAFGTGIGGEFDISKARYHKIVIMTDADVDGAHIRTLLLTFFYRFMRPLIEAGYVYIAQPPLYKLTQGKQKYYVFNDR**

35 OM08-17AT 90.7% 100.0%  **MITAFGTGIGGEFDISKARYHKIVIMTDADVDGAHIRTLLLTFFYRFMRPLIEAGYVYIAQPPLYKLTQGKQKYYVFNDR**

36 SWO 100.0% 100.0%  **MITAFGTGIGGEFDISKARYHKIVIMTDADVDGAHIRTLLLTFFYRFMRPLIEAGYVYIAQPPLYKLTQGKQKYYVFNDR**

37 VCU121 99.4% 99.8%  **MITAFGTGIGGEFDISKARYHKIVIMTDADVDGAHIRTLLLTFFYRFMRPLIEAGYVYIAQPPLYKLTQGKQKYYVFNDR**

consensus/100%  **MITAFGTGIGGEFDISKARYHKIVIMTDADVDGAHIRTLLLTFFYRFMRPLIEAGYVYIAQPPLYKLTQGKQKYYVFNDR**

consensus/90%  **MITAFGTGIGGEFDISKARYHKIVIMTDADVDGAHIRTLLLTFFYRFMRPLIEAGYVYIAQPPLYKLTQGKQKYYVFNDR**

consensus/80%  **MITAFGTGIGGEFDISKARYHKIVIMTDADVDGAHIRTLLLTFFYRFMRPLIEAGYVYIAQPPLYKLTQGKQKYYVFNDR**

consensus/70%  **MITAFGTGIGGEFDISKARYHKIVIMTDADVDGAHIRTLLLTFFYRFMRPLIEAGYVYIAQPPLYKLTQGKQKYYVFNDR**

cov pid **561**  **. . . 6 . . . .** **640**

1 29AM 100.0% 100.0%  **ELDKLKAELNPTPKWSIARYKGLGEMNADQLWETTMNPEHRSMLQVTLEDAIDADQTFEMLMGDIVENRRQFIEDNAVYA**

2 TRPF4 100.0% 100.0%  **ELDKLKAELNPTPKWSIARYKGLGEMNADQLWETTMNPEHRSMLQVTLEDAIDADQTFEMLMGDIVENRRQFIEDNAVYA**

3 P912 100.0% 100.0%  **ELDKLKAELNPTPKWSIARYKGLGEMNADQLWETTMNPEHRSMLQVTLEDAIDADQTFEMLMGDIVENRRQFIEDNAVYA**

4 NGS-ED-1001 100.0% 100.0%  **ELDKLKAELNPTPKWSIARYKGLGEMNADQLWETTMNPEHRSMLQVTLEDAIDADQTFEMLMGDIVENRRQFIEDNAVYA**

5 1DB1 100.0% 100.0%  **ELDKLKAELNPTPKWSIARYKGLGEMNADQLWETTMNPEHRSMLQVTLEDAIDADQTFEMLMGDIVENRRQFIEDNAVYA**

6 MGYG-HGUT-02301 100.0% 100.0%  **ELDKLKAELNPTPKWSIARYKGLGEMNADQLWETTMNPEHRSMLQVTLEDAIDADQTFEMLMGDIVENRRQFIEDNAVYA**

7 IIF4SW-P1 100.0% 100.0%  **ELDKLKAELNPTPKWSIARYKGLGEMNADQLWETTMNPEHRSMLQVTLEDAIDADQTFEMLMGDIVENRRQFIEDNAVYA**

8 19428wF1_P912 100.0% 100.0%  **ELDKLKAELNPTPKWSIARYKGLGEMNADQLWETTMNPEHRSMLQVTLEDAIDADQTFEMLMGDIVENRRQFIEDNAVYA**

9 R5992 100.0% 100.0%  **ELDKLKAELNPTPKWSIARYKGLGEMNADQLWETTMNPEHRSMLQVTLEDAIDADQTFEMLMGDIVENRRQFIEDNAVYA**

10 R5990 100.0% 100.0%  **ELDKLKAELNPTPKWSIARYKGLGEMNADQLWETTMNPEHRSMLQVTLEDAIDADQTFEMLMGDIVENRRQFIEDNAVYA**

11 R5991 100.0% 100.0%  **ELDKLKAELNPTPKWSIARYKGLGEMNADQLWETTMNPEHRSMLQVTLEDAIDADQTFEMLMGDIVENRRQFIEDNAVYA**

12 R6028 100.0% 100.0%  **ELDKLKAELNPTPKWSIARYKGLGEMNADQLWETTMNPEHRSMLQVTLEDAIDADQTFEMLMGDIVENRRQFIEDNAVYA**

13 R6027 100.0% 100.0%  **ELDKLKAELNPTPKWSIARYKGLGEMNADQLWETTMNPEHRSMLQVTLEDAIDADQTFEMLMGDIVENRRQFIEDNAVYA**

14 R6024 90.7% 100.0%  **ELDKLKAELNPTPKWSIARYKGLGEMNADQLWETTMNPEHRSMLQVTLEDAIDADQTFEMLMGDIVENRRQFIEDNAVYA**

15 L2_057 100.0% 100.0%  **ELDKLKAELNPTPKWSIARYKGLGEMNADQLWETTMNPEHRSMLQVTLEDAIDADQTFEMLMGDIVENRRQFIEDNAVYA**

16 Cap100.1 100.0% 100.0%  **ELDKLKAELNPTPKWSIARYKGLGEMNADQLWETTMNPEHRSMLQVTLEDAIDADQTFEMLMGDIVENRRQFIEDNAVYA**

17 Ani-LG-057 100.0% 100.0%  **ELDKLKAELNPTPKWSIARYKGLGEMNADQLWETTMNPEHRSMLQVTLEDAIDADQTFEMLMGDIVENRRQFIEDNAVYA**

18 Cap9.2 100.0% 100.0%  **ELDKLKAELNPTPKWSIARYKGLGEMNADQLWETTMNPEHRSMLQVTLEDAIDADQTFEMLMGDIVENRRQFIEDNAVYA**

19 Cap10.1 100.0% 100.0%  **ELDKLKAELNPTPKWSIARYKGLGEMNADQLWETTMNPEHRSMLQVTLEDAIDADQTFEMLMGDIVENRRQFIEDNAVYA**

20 Cap9.1 100.0% 100.0%  **ELDKLKAELNPTPKWSIARYKGLGEMNADQLWETTMNPEHRSMLQVTLEDAIDADQTFEMLMGDIVENRRQFIEDNAVYA**

21 Cap10.2 100.0% 100.0%  **ELDKLKAELNPTPKWSIARYKGLGEMNADQLWETTMNPEHRSMLQVTLEDAIDADQTFEMLMGDIVENRRQFIEDNAVYA**

22 acrok 100.0% 100.0%  **ELDKLKAELNPTPKWSIARYKGLGEMNADQLWETTMNPEHRSMLQVTLEDAIDADQTFEMLMGDIVENRRQFIEDNAVYA**

23 acroj 100.0% 100.0%  **ELDKLKAELNPTPKWSIARYKGLGEMNADQLWETTMNPEHRSMLQVTLEDAIDADQTFEMLMGDIVENRRQFIEDNAVYA**

24 acror 100.0% 100.0%  **ELDKLKAELNPTPKWSIARYKGLGEMNADQLWETTMNPEHRSMLQVTLEDAIDADQTFEMLMGDIVENRRQFIEDNAVYA**

25 NJ6 100.0% 100.0%  **ELDKLKAELNPTPKWSIARYKGLGEMNADQLWETTMNPEHRSMLQVTLEDAIDADQTFEMLMGDIVENRRQFIEDNAVYA**

26 EYE_450 100.0% 100.0%  **ELDKLKAELNPTPKWSIARYKGLGEMNADQLWETTMNPEHRSMLQVTLEDAIDADQTFEMLMGDIVENRRQFIEDNAVYA**

27 EYE_117 100.0% 100.0%  **ELDKLKAELNPTPKWSIARYKGLGEMNADQLWETTMNPEHRSMLQVTLEDAIDADQTFEMLMGDIVENRRQFIEDNAVYA**

28 EYE_410 100.0% 100.0%  **ELDKLKAELNPTPKWSIARYKGLGEMNADQLWETTMNPEHRSMLQVTLEDAIDADQTFEMLMGDIVENRRQFIEDNAVYA**

29 EYE_411 100.0% 100.0%  **ELDKLKAELNPTPKWSIARYKGLGEMNADQLWETTMNPEHRSMLQVTLEDAIDADQTFEMLMGDIVENRRQFIEDNAVYA**

30 MERTA18 100.0% 100.0%  **ELDKLKAELNPTPKWSIARYKGLGEMNADQLWETTMNPEHRSMLQVTLEDAIDADQTFEMLMGDIVENRRQFIEDNAVYA**

31 FAIRING19B-1.2 100.0% 100.0%  **ELDKLKAELNPTPKWSIARYKGLGEMNADQLWETTMNPEHRSMLQVTLEDAIDADQTFEMLMGDIVENRRQFIEDNAVYA**

32 FDAARGOS_151 90.7% 100.0%  **ELDKLKAELNPTPKWSIARYKGLGEMNADQLWETTMNPEHRSMLQVTLEDAIDADQTFEMLMGDIVENRRQFIEDNAVYA**

33 SNUC5989 100.0% 100.0%  **ELDKLKAELNPTPKWSIARYKGLGEMNADQLWETTMNPEHRSMLQVTLEDAIDADQTFEMLMGDIVENRRQFIEDNAVYA**

34 SNUC3412 100.0% 99.8%  **ELDKLKAELNPTPKWSIARYKGLGEMNADQLWETTMNPEHRSMLQVTLEDAIDADQTFEMLMGDIVENRRQFIEDNAVYA**

35 OM08-17AT 90.7% 100.0%  **ELDKLKAELNPTPKWSIARYKGLGEMNADQLWETTMNPEHRSMLQVTLEDAIDADQTFEMLMGDIVENRRQFIEDNAVYA**

36 SWO 100.0% 100.0%  **ELDKLKAELNPTPKWSIARYKGLGEMNADQLWETTMNPEHRSMLQVTLEDAIDADQTFEMLMGDIVENRRQFIEDNAVYA**

37 VCU121 99.4% 99.8%  **ELDKLKAELNPTPKWSIARYKGLGEMNADQLWETTMNPEHRSMLQVTLEDAIDADQTFEMLMGDIVENRRQFIEDNAVYA**

consensus/100%  **ELDKLKAELNPTPKWSIARYKGLGEMNADQLWETTMNPEHRSMLQVTLEDAIDADQTFEMLMGDIVENRRQFIEDNAVYA**

consensus/90%  **ELDKLKAELNPTPKWSIARYKGLGEMNADQLWETTMNPEHRSMLQVTLEDAIDADQTFEMLMGDIVENRRQFIEDNAVYA**

consensus/80%  **ELDKLKAELNPTPKWSIARYKGLGEMNADQLWETTMNPEHRSMLQVTLEDAIDADQTFEMLMGDIVENRRQFIEDNAVYA**

consensus/70%  **ELDKLKAELNPTPKWSIARYKGLGEMNADQLWETTMNPEHRSMLQVTLEDAIDADQTFEMLMGDIVENRRQFIEDNAVYA**

cov pid **641**  **]** **644**

1 29AM 100.0% 100.0%  **NLDF**

2 TRPF4 100.0% 100.0%  **NLDF**

3 P912 100.0% 100.0%  **NLDF**

4 NGS-ED-1001 100.0% 100.0%  **NLDF**

5 1DB1 100.0% 100.0%  **NLDF**

6 MGYG-HGUT-02301 100.0% 100.0%  **NLDF**

7 IIF4SW-P1 100.0% 100.0%  **NLDF**

8 19428wF1_P912 100.0% 100.0%  **NLDF**

9 R5992 100.0% 100.0%  **NLDF**

10 R5990 100.0% 100.0%  **NLDF**

11 R5991 100.0% 100.0%  **NLDF**

12 R6028 100.0% 100.0%  **NLDF**

13 R6027 100.0% 100.0%  **NLDF**

14 R6024 90.7% 100.0%  **NLDF**

15 L2_057 100.0% 100.0%  **NLDF**

16 Cap100.1 100.0% 100.0%  **NLDF**

17 Ani-LG-057 100.0% 100.0%  **NLDF**

18 Cap9.2 100.0% 100.0%  **NLDF**

19 Cap10.1 100.0% 100.0%  **NLDF**

20 Cap9.1 100.0% 100.0%  **NLDF**

21 Cap10.2 100.0% 100.0%  **NLDF**

22 acrok 100.0% 100.0%  **NLDF**

23 acroj 100.0% 100.0%  **NLDF**

24 acror 100.0% 100.0%  **NLDF**

25 NJ6 100.0% 100.0%  **NLDF**

26 EYE_450 100.0% 100.0%  **NLDF**

27 EYE_117 100.0% 100.0%  **NLDF**

28 EYE_410 100.0% 100.0%  **NLDF**

29 EYE_411 100.0% 100.0%  **NLDF**

30 MERTA18 100.0% 100.0%  **NLDF**

31 FAIRING19B-1.2 100.0% 100.0%  **NLDF**

32 FDAARGOS_151 90.7% 100.0%  **NLDF**

33 SNUC5989 100.0% 100.0%  **NLDF**

34 SNUC3412 100.0% 99.8%  **NLDF**

35 OM08-17AT 90.7% 100.0%  **NLDF**

36 SWO 100.0% 100.0%  **NLDF**

37 VCU121 99.4% 99.8%  **NLDF**

consensus/100%  **NLDF**

consensus/90%  **NLDF**

consensus/80%  **NLDF**

consensus/70%  **NLDF**

**Supplementary Figure 7.** Multiple sequence alignment of the predicted amino acid sequences of GyrB in *S. warneri* isolate 29AM compared to that of the closely related *S. warneri* genomes retrieved from the BV-BRC database. The alignment was visualized using MView version 1.63 hosted by the EMBL-EBI; cov, coverage; pid, percent identity.

cov pid  **1** **[ . . . . : . . .** **80**

1 29AM 100.0% 100.0%  **MSEIIQDLSLEDVIGDRFGRYSKYIIQERALPDVRDGLKPVQRRILYAMYSSGNTFDKNFRKSAKTVGDVIGQYHPHGDS**

2 TRPF4 100.0% 100.0%  **MSEIIQDLSLEDVIGDRFGRYSKYIIQERALPDVRDGLKPVQRRILYAMYSSGNTFDKNFRKSAKTVGDVIGQYHPHGDS**

3 P912 100.0% 100.0%  **MSEIIQDLSLEDVIGDRFGRYSKYIIQERALPDVRDGLKPVQRRILYAMYSSGNTFDKNFRKSAKTVGDVIGQYHPHGDS**

4 NGS-ED-1001 92.5% 100.0%  **------------------------------------------------------------RKSAKTVGDVIGQYHPHGDS**

5 1DB1 92.5% 100.0%  **------------------------------------------------------------RKSAKTVGDVIGQYHPHGDS**

6 MGYG-HGUT-02301 92.5% 100.0%  **------------------------------------------------------------RKSAKTVGDVIGQYHPHGDS**

7 IIF4SW-P1 100.0% 100.0%  **MSEIIQDLSLEDVIGDRFGRYSKYIIQERALPDVRDGLKPVQRRILYAMYSSGNTFDKNFRKSAKTVGDVIGQYHPHGDS**

8 19428wF1_P912 92.5% 100.0%  **------------------------------------------------------------RKSAKTVGDVIGQYHPHGDS**

9 R5992 92.5% 100.0%  **------------------------------------------------------------RKSAKTVGDVIGQYHPHGDS**

10 R5990 92.5% 100.0%  **------------------------------------------------------------RKSAKTVGDVIGQYHPHGDS**

11 R5991 92.5% 100.0%  **------------------------------------------------------------RKSAKTVGDVIGQYHPHGDS**

12 R6028 92.5% 99.9%  **------------------------------------------------------------RKSAKTVGDVIGQYHPHGDS**

13 R6027 92.5% 99.9%  **------------------------------------------------------------RKSAKTVGDVIGQYHPHGDS**

14 R6024 100.0% 99.9%  **MSEIIQDLSLEDVIGDRFGRYSKYIIQERALPDVRDGLKPVQRRILYAMYSSGNTFDKNFRKSAKTVGDVIGQYHPHGDS**

15 L2_057 100.0% 100.0%  **MSEIIQDLSLEDVIGDRFGRYSKYIIQERALPDVRDGLKPVQRRILYAMYSSGNTFDKNFRKSAKTVGDVIGQYHPHGDS**

16 Cap100.1 100.0% 100.0%  **MSEIIQDLSLEDVIGDRFGRYSKYIIQERALPDVRDGLKPVQRRILYAMYSSGNTFDKNFRKSAKTVGDVIGQYHPHGDS**

17 Ani-LG-057 100.0% 100.0%  **MSEIIQDLSLEDVIGDRFGRYSKYIIQERALPDVRDGLKPVQRRILYAMYSSGNTFDKNFRKSAKTVGDVIGQYHPHGDS**

18 Cap9.2 92.5% 100.0%  **------------------------------------------------------------RKSAKTVGDVIGQYHPHGDS**

19 Cap10.1 92.5% 100.0%  **------------------------------------------------------------RKSAKTVGDVIGQYHPHGDS**

20 Cap9.1 100.0% 100.0%  **MSEIIQDLSLEDVIGDRFGRYSKYIIQERALPDVRDGLKPVQRRILYAMYSSGNTFDKNFRKSAKTVGDVIGQYHPHGDS**

21 Cap10.2 100.0% 100.0%  **MSEIIQDLSLEDVIGDRFGRYSKYIIQERALPDVRDGLKPVQRRILYAMYSSGNTFDKNFRKSAKTVGDVIGQYHPHGDS**

22 acrok 100.0% 100.0%  **MSEIIQDLSLEDVIGDRFGRYSKYIIQERALPDVRDGLKPVQRRILYAMYSSGNTFDKNFRKSAKTVGDVIGQYHPHGDS**

23 acroj 92.5% 100.0%  **------------------------------------------------------------RKSAKTVGDVIGQYHPHGDS**

24 acror 92.5% 100.0%  **------------------------------------------------------------RKSAKTVGDVIGQYHPHGDS**

25 NJ6 100.0% 100.0%  **MSEIIQDLSLEDVIGDRFGRYSKYIIQERALPDVRDGLKPVQRRILYAMYSSGNTFDKNFRKSAKTVGDVIGQYHPHGDS**

26 EYE_450 100.0% 100.0%  **MSEIIQDLSLEDVIGDRFGRYSKYIIQERALPDVRDGLKPVQRRILYAMYSSGNTFDKNFRKSAKTVGDVIGQYHPHGDS**

27 EYE_117 92.5% 100.0%  **------------------------------------------------------------RKSAKTVGDVIGQYHPHGDS**

28 EYE_410 92.5% 100.0%  **------------------------------------------------------------RKSAKTVGDVIGQYHPHGDS**

29 EYE_411 92.5% 100.0%  **------------------------------------------------------------RKSAKTVGDVIGQYHPHGDS**

30 MERTA18 92.5% 100.0%  **------------------------------------------------------------RKSAKTVGDVIGQYHPHGDS**

31 FAIRING19B-1.2 100.0% 100.0%  **MSEIIQDLSLEDVIGDRFGRYSKYIIQERALPDVRDGLKPVQRRILYAMYSSGNTFDKNFRKSAKTVGDVIGQYHPHGDS**

32 FDAARGOS_151 92.5% 99.7%  **------------------------------------------------------------RKSAKTVGDVIGQYHPHGDS**

33 SNUC5989 100.0% 100.0%  **MSEIIQDLSLEDVIGDRFGRYSKYIIQERALPDVRDGLKPVQRRILYAMYSSGNTFDKNFRKSAKTVGDVIGQYHPHGDS**

34 SNUC3412 100.0% 100.0%  **MSEIIQDLSLEDVIGDRFGRYSKYIIQERALPDVRDGLKPVQRRILYAMYSSGNTFDKNFRKSAKTVGDVIGQYHPHGDS**

35 OM08-17AT 100.0% 100.0%  **MSEIIQDLSLEDVIGDRFGRYSKYIIQERALPDVRDGLKPVQRRILYAMYSSGNTFDKNFRKSAKTVGDVIGQYHPHGDS**

36 SWO 100.0% 100.0%  **MSEIIQDLSLEDVIGDRFGRYSKYIIQERALPDVRDGLKPVQRRILYAMYSSGNTFDKNFRKSAKTVGDVIGQYHPHGDS**

consensus/100%  **............................................................RKSAKTVGDVIGQYHPHGDS**

consensus/90%  **............................................................RKSAKTVGDVIGQYHPHGDS**

consensus/80%  **............................................................RKSAKTVGDVIGQYHPHGDS**

consensus/70%  **............................................................RKSAKTVGDVIGQYHPHGDS**

cov pid  **81**  **. 1 . . . . : .** **160**

1 29AM 100.0% 100.0%  **SVYDAMVRLSQDWKLRHVLIEMHGNNGSIDNDPPAAMRYTEAKLSQLSEELLRDINKETVSFIPNYDDTTLEPMVLPARF**

2 TRPF4 100.0% 100.0%  **SVYDAMVRLSQDWKLRHVLIEMHGNNGSIDNDPPAAMRYTEAKLSQLSEELLRDINKETVSFIPNYDDTTLEPMVLPARF**

3 P912 100.0% 100.0%  **SVYDAMVRLSQDWKLRHVLIEMHGNNGSIDNDPPAAMRYTEAKLSQLSEELLRDINKETVSFIPNYDDTTLEPMVLPARF**

4 NGS-ED-1001 92.5% 100.0%  **SVYDAMVRLSQDWKLRHVLIEMHGNNGSIDNDPPAAMRYTEAKLSQLSEELLRDINKETVSFIPNYDDTTLEPMVLPARF**

5 1DB1 92.5% 100.0%  **SVYDAMVRLSQDWKLRHVLIEMHGNNGSIDNDPPAAMRYTEAKLSQLSEELLRDINKETVSFIPNYDDTTLEPMVLPARF**

6 MGYG-HGUT-02301 92.5% 100.0%  **SVYDAMVRLSQDWKLRHVLIEMHGNNGSIDNDPPAAMRYTEAKLSQLSEELLRDINKETVSFIPNYDDTTLEPMVLPARF**

7 IIF4SW-P1 100.0% 100.0%  **SVYDAMVRLSQDWKLRHVLIEMHGNNGSIDNDPPAAMRYTEAKLSQLSEELLRDINKETVSFIPNYDDTTLEPMVLPARF**

8 19428wF1_P912 92.5% 100.0%  **SVYDAMVRLSQDWKLRHVLIEMHGNNGSIDNDPPAAMRYTEAKLSQLSEELLRDINKETVSFIPNYDDTTLEPMVLPARF**

9 R5992 92.5% 100.0%  **SVYDAMVRLSQDWKLRHVLIEMHGNNGSIDNDPPAAMRYTEAKLSQLSEELLRDINKETVSFIPNYDDTTLEPMVLPARF**

10 R5990 92.5% 100.0%  **SVYDAMVRLSQDWKLRHVLIEMHGNNGSIDNDPPAAMRYTEAKLSQLSEELLRDINKETVSFIPNYDDTTLEPMVLPARF**

11 R5991 92.5% 100.0%  **SVYDAMVRLSQDWKLRHVLIEMHGNNGSIDNDPPAAMRYTEAKLSQLSEELLRDINKETVSFIPNYDDTTLEPMVLPARF**

12 R6028 92.5% 99.9%  **SVYDAMVRLSQDWKLRHVLIEMHGNNGSIDNDPPAAMRYTEAKLSQLSEELLRDINKETVSFIPNYDDTTLEPMVLPARF**

13 R6027 92.5% 99.9%  **SVYDAMVRLSQDWKLRHVLIEMHGNNGSIDNDPPAAMRYTEAKLSQLSEELLRDINKETVSFIPNYDDTTLEPMVLPARF**

14 R6024 100.0% 99.9%  **SVYDAMVRLSQDWKLRHVLIEMHGNNGSIDNDPPAAMRYTEAKLSQLSEELLRDINKETVSFIPNYDDTTLEPMVLPARF**

15 L2_057 100.0% 100.0%  **SVYDAMVRLSQDWKLRHVLIEMHGNNGSIDNDPPAAMRYTEAKLSQLSEELLRDINKETVSFIPNYDDTTLEPMVLPARF**

16 Cap100.1 100.0% 100.0%  **SVYDAMVRLSQDWKLRHVLIEMHGNNGSIDNDPPAAMRYTEAKLSQLSEELLRDINKETVSFIPNYDDTTLEPMVLPARF**

17 Ani-LG-057 100.0% 100.0%  **SVYDAMVRLSQDWKLRHVLIEMHGNNGSIDNDPPAAMRYTEAKLSQLSEELLRDINKETVSFIPNYDDTTLEPMVLPARF**

18 Cap9.2 92.5% 100.0%  **SVYDAMVRLSQDWKLRHVLIEMHGNNGSIDNDPPAAMRYTEAKLSQLSEELLRDINKETVSFIPNYDDTTLEPMVLPARF**

19 Cap10.1 92.5% 100.0%  **SVYDAMVRLSQDWKLRHVLIEMHGNNGSIDNDPPAAMRYTEAKLSQLSEELLRDINKETVSFIPNYDDTTLEPMVLPARF**

20 Cap9.1 100.0% 100.0%  **SVYDAMVRLSQDWKLRHVLIEMHGNNGSIDNDPPAAMRYTEAKLSQLSEELLRDINKETVSFIPNYDDTTLEPMVLPARF**

21 Cap10.2 100.0% 100.0%  **SVYDAMVRLSQDWKLRHVLIEMHGNNGSIDNDPPAAMRYTEAKLSQLSEELLRDINKETVSFIPNYDDTTLEPMVLPARF**

22 acrok 100.0% 100.0%  **SVYDAMVRLSQDWKLRHVLIEMHGNNGSIDNDPPAAMRYTEAKLSQLSEELLRDINKETVSFIPNYDDTTLEPMVLPARF**

23 acroj 92.5% 100.0%  **SVYDAMVRLSQDWKLRHVLIEMHGNNGSIDNDPPAAMRYTEAKLSQLSEELLRDINKETVSFIPNYDDTTLEPMVLPARF**

24 acror 92.5% 100.0%  **SVYDAMVRLSQDWKLRHVLIEMHGNNGSIDNDPPAAMRYTEAKLSQLSEELLRDINKETVSFIPNYDDTTLEPMVLPARF**

25 NJ6 100.0% 100.0%  **SVYDAMVRLSQDWKLRHVLIEMHGNNGSIDNDPPAAMRYTEAKLSQLSEELLRDINKETVSFIPNYDDTTLEPMVLPARF**

26 EYE_450 100.0% 100.0%  **SVYDAMVRLSQDWKLRHVLIEMHGNNGSIDNDPPAAMRYTEAKLSQLSEELLRDINKETVSFIPNYDDTTLEPMVLPARF**

27 EYE_117 92.5% 100.0%  **SVYDAMVRLSQDWKLRHVLIEMHGNNGSIDNDPPAAMRYTEAKLSQLSEELLRDINKETVSFIPNYDDTTLEPMVLPARF**

28 EYE_410 92.5% 100.0%  **SVYDAMVRLSQDWKLRHVLIEMHGNNGSIDNDPPAAMRYTEAKLSQLSEELLRDINKETVSFIPNYDDTTLEPMVLPARF**

29 EYE_411 92.5% 100.0%  **SVYDAMVRLSQDWKLRHVLIEMHGNNGSIDNDPPAAMRYTEAKLSQLSEELLRDINKETVSFIPNYDDTTLEPMVLPARF**

30 MERTA18 92.5% 100.0%  **SVYDAMVRLSQDWKLRHVLIEMHGNNGSIDNDPPAAMRYTEAKLSQLSEELLRDINKETVSFIPNYDDTTLEPMVLPARF**

31 FAIRING19B-1.2 100.0% 100.0%  **SVYDAMVRLSQDWKLRHVLIEMHGNNGSIDNDPPAAMRYTEAKLSQLSEELLRDINKETVSFIPNYDDTTLEPMVLPARF**

32 FDAARGOS_151 92.5% 99.7%  **SVYDAMVRLSQDWKLRHVLIEMHGNNGSIDNDPSAAMRYTEAKLSQLSEELLRDINKETVSFIPNYDDTTLEPMVLPARF**

33 SNUC5989 100.0% 100.0%  **SVYDAMVRLSQDWKLRHVLIEMHGNNGSIDNDPPAAMRYTEAKLSQLSEELLRDINKETVSFIPNYDDTTLEPMVLPARF**

34 SNUC3412 100.0% 100.0%  **SVYDAMVRLSQDWKLRHVLIEMHGNNGSIDNDPPAAMRYTEAKLSQLSEELLRDINKETVSFIPNYDDTTLEPMVLPARF**

35 OM08-17AT 100.0% 100.0%  **SVYDAMVRLSQDWKLRHVLIEMHGNNGSIDNDPPAAMRYTEAKLSQLSEELLRDINKETVSFIPNYDDTTLEPMVLPARF**

36 SWO 100.0% 100.0%  **SVYDAMVRLSQDWKLRHVLIEMHGNNGSIDNDPPAAMRYTEAKLSQLSEELLRDINKETVSFIPNYDDTTLEPMVLPARF**

consensus/100%  **SVYDAMVRLSQDWKLRHVLIEMHGNNGSIDNDPsAAMRYTEAKLSQLSEELLRDINKETVSFIPNYDDTTLEPMVLPARF**

consensus/90%  **SVYDAMVRLSQDWKLRHVLIEMHGNNGSIDNDPPAAMRYTEAKLSQLSEELLRDINKETVSFIPNYDDTTLEPMVLPARF**

consensus/80%  **SVYDAMVRLSQDWKLRHVLIEMHGNNGSIDNDPPAAMRYTEAKLSQLSEELLRDINKETVSFIPNYDDTTLEPMVLPARF**

consensus/70%  **SVYDAMVRLSQDWKLRHVLIEMHGNNGSIDNDPPAAMRYTEAKLSQLSEELLRDINKETVSFIPNYDDTTLEPMVLPARF**

cov pid **161**  **. . . 2 . . . .** **240**

1 29AM 100.0% 100.0%  **PNLLINGSTGISAGYATDIPPHNLGEVIQATLKYIDNPDITVSQLMKYIKGPDFPTGGIIQGLDGIKKAYESGKGKIVVR**

2 TRPF4 100.0% 100.0%  **PNLLINGSTGISAGYATDIPPHNLGEVIQATLKYIDNPDITVSQLMKYIKGPDFPTGGIIQGLDGIKKAYESGKGKIVVR**

3 P912 100.0% 100.0%  **PNLLINGSTGISAGYATDIPPHNLGEVIQATLKYIDNPDITVSQLMKYIKGPDFPTGGIIQGLDGIKKAYESGKGKIVVR**

4 NGS-ED-1001 92.5% 100.0%  **PNLLINGSTGISAGYATDIPPHNLGEVIQATLKYIDNPDITVSQLMKYIKGPDFPTGGIIQGLDGIKKAYESGKGKIVVR**

5 1DB1 92.5% 100.0%  **PNLLINGSTGISAGYATDIPPHNLGEVIQATLKYIDNPDITVSQLMKYIKGPDFPTGGIIQGLDGIKKAYESGKGKIVVR**

6 MGYG-HGUT-02301 92.5% 100.0%  **PNLLINGSTGISAGYATDIPPHNLGEVIQATLKYIDNPDITVSQLMKYIKGPDFPTGGIIQGLDGIKKAYESGKGKIVVR**

7 IIF4SW-P1 100.0% 100.0%  **PNLLINGSTGISAGYATDIPPHNLGEVIQATLKYIDNPDITVSQLMKYIKGPDFPTGGIIQGLDGIKKAYESGKGKIVVR**

8 19428wF1_P912 92.5% 100.0%  **PNLLINGSTGISAGYATDIPPHNLGEVIQATLKYIDNPDITVSQLMKYIKGPDFPTGGIIQGLDGIKKAYESGKGKIVVR**

9 R5992 92.5% 100.0%  **PNLLINGSTGISAGYATDIPPHNLGEVIQATLKYIDNPDITVSQLMKYIKGPDFPTGGIIQGLDGIKKAYESGKGKIVVR**

10 R5990 92.5% 100.0%  **PNLLINGSTGISAGYATDIPPHNLGEVIQATLKYIDNPDITVSQLMKYIKGPDFPTGGIIQGLDGIKKAYESGKGKIVVR**

11 R5991 92.5% 100.0%  **PNLLINGSTGISAGYATDIPPHNLGEVIQATLKYIDNPDITVSQLMKYIKGPDFPTGGIIQGLDGIKKAYESGKGKIVVR**

12 R6028 92.5% 99.9%  **PNLLINGSTGISAGYATDIPPHNLGEVIQATLKYIDNPDITVSQLMKYIKGPDFPTGGIIQGLDGIKKAYESGKGKIVVR**

13 R6027 92.5% 99.9%  **PNLLINGSTGISAGYATDIPPHNLGEVIQATLKYIDNPDITVSQLMKYIKGPDFPTGGIIQGLDGIKKAYESGKGKIVVR**

14 R6024 100.0% 99.9%  **PNLLINGSTGISAGYATDIPPHNLGEVIQATLKYIDNPDITVSQLMKYIKGPDFPTGGIIQGLDGIKKAYESGKGKIVVR**

15 L2_057 100.0% 100.0%  **PNLLINGSTGISAGYATDIPPHNLGEVIQATLKYIDNPDITVSQLMKYIKGPDFPTGGIIQGLDGIKKAYESGKGKIVVR**

16 Cap100.1 100.0% 100.0%  **PNLLINGSTGISAGYATDIPPHNLGEVIQATLKYIDNPDITVSQLMKYIKGPDFPTGGIIQGLDGIKKAYESGKGKIVVR**

17 Ani-LG-057 100.0% 100.0%  **PNLLINGSTGISAGYATDIPPHNLGEVIQATLKYIDNPDITVSQLMKYIKGPDFPTGGIIQGLDGIKKAYESGKGKIVVR**

18 Cap9.2 92.5% 100.0%  **PNLLINGSTGISAGYATDIPPHNLGEVIQATLKYIDNPDITVSQLMKYIKGPDFPTGGIIQGLDGIKKAYESGKGKIVVR**

19 Cap10.1 92.5% 100.0%  **PNLLINGSTGISAGYATDIPPHNLGEVIQATLKYIDNPDITVSQLMKYIKGPDFPTGGIIQGLDGIKKAYESGKGKIVVR**

20 Cap9.1 100.0% 100.0%  **PNLLINGSTGISAGYATDIPPHNLGEVIQATLKYIDNPDITVSQLMKYIKGPDFPTGGIIQGLDGIKKAYESGKGKIVVR**

21 Cap10.2 100.0% 100.0%  **PNLLINGSTGISAGYATDIPPHNLGEVIQATLKYIDNPDITVSQLMKYIKGPDFPTGGIIQGLDGIKKAYESGKGKIVVR**

22 acrok 100.0% 100.0%  **PNLLINGSTGISAGYATDIPPHNLGEVIQATLKYIDNPDITVSQLMKYIKGPDFPTGGIIQGLDGIKKAYESGKGKIVVR**

23 acroj 92.5% 100.0%  **PNLLINGSTGISAGYATDIPPHNLGEVIQATLKYIDNPDITVSQLMKYIKGPDFPTGGIIQGLDGIKKAYESGKGKIVVR**

24 acror 92.5% 100.0%  **PNLLINGSTGISAGYATDIPPHNLGEVIQATLKYIDNPDITVSQLMKYIKGPDFPTGGIIQGLDGIKKAYESGKGKIVVR**

25 NJ6 100.0% 100.0%  **PNLLINGSTGISAGYATDIPPHNLGEVIQATLKYIDNPDITVSQLMKYIKGPDFPTGGIIQGLDGIKKAYESGKGKIVVR**

26 EYE_450 100.0% 100.0%  **PNLLINGSTGISAGYATDIPPHNLGEVIQATLKYIDNPDITVSQLMKYIKGPDFPTGGIIQGLDGIKKAYESGKGKIVVR**

27 EYE_117 92.5% 100.0%  **PNLLINGSTGISAGYATDIPPHNLGEVIQATLKYIDNPDITVSQLMKYIKGPDFPTGGIIQGLDGIKKAYESGKGKIVVR**

28 EYE_410 92.5% 100.0%  **PNLLINGSTGISAGYATDIPPHNLGEVIQATLKYIDNPDITVSQLMKYIKGPDFPTGGIIQGLDGIKKAYESGKGKIVVR**

29 EYE_411 92.5% 100.0%  **PNLLINGSTGISAGYATDIPPHNLGEVIQATLKYIDNPDITVSQLMKYIKGPDFPTGGIIQGLDGIKKAYESGKGKIVVR**

30 MERTA18 92.5% 100.0%  **PNLLINGSTGISAGYATDIPPHNLGEVIQATLKYIDNPDITVSQLMKYIKGPDFPTGGIIQGLDGIKKAYESGKGKIVVR**

31 FAIRING19B-1.2 100.0% 100.0%  **PNLLINGSTGISAGYATDIPPHNLGEVIQATLKYIDNPDITVSQLMKYIKGPDFPTGGIIQGLDGIKKAYESGKGKIVVR**

32 FDAARGOS_151 92.5% 99.7%  **PNLLINGSTGISAGYATDIPPHNLGEVIQATLKYIDNPDITVSQLMKYIKGPDFPTGGIIQGLDGIKKAYESGKGKIVVR**

33 SNUC5989 100.0% 100.0%  **PNLLINGSTGISAGYATDIPPHNLGEVIQATLKYIDNPDITVSQLMKYIKGPDFPTGGIIQGLDGIKKAYESGKGKIVVR**

34 SNUC3412 100.0% 100.0%  **PNLLINGSTGISAGYATDIPPHNLGEVIQATLKYIDNPDITVSQLMKYIKGPDFPTGGIIQGLDGIKKAYESGKGKIVVR**

35 OM08-17AT 100.0% 100.0%  **PNLLINGSTGISAGYATDIPPHNLGEVIQATLKYIDNPDITVSQLMKYIKGPDFPTGGIIQGLDGIKKAYESGKGKIVVR**

36 SWO 100.0% 100.0%  **PNLLINGSTGISAGYATDIPPHNLGEVIQATLKYIDNPDITVSQLMKYIKGPDFPTGGIIQGLDGIKKAYESGKGKIVVR**

consensus/100%  **PNLLINGSTGISAGYATDIPPHNLGEVIQATLKYIDNPDITVSQLMKYIKGPDFPTGGIIQGLDGIKKAYESGKGKIVVR**

consensus/90%  **PNLLINGSTGISAGYATDIPPHNLGEVIQATLKYIDNPDITVSQLMKYIKGPDFPTGGIIQGLDGIKKAYESGKGKIVVR**

consensus/80%  **PNLLINGSTGISAGYATDIPPHNLGEVIQATLKYIDNPDITVSQLMKYIKGPDFPTGGIIQGLDGIKKAYESGKGKIVVR**

consensus/70%  **PNLLINGSTGISAGYATDIPPHNLGEVIQATLKYIDNPDITVSQLMKYIKGPDFPTGGIIQGLDGIKKAYESGKGKIVVR**

cov pid **241**  **: . . . . 3 . .** **320**

1 29AM 100.0% 100.0%  **SKVDEEELRNGRKELIVTEIPYEVNKSSLVKRIDELRADKKVDGIVEVRDETDRTGLRIAIELKKDVNSEAIKNFLYKNS**

2 TRPF4 100.0% 100.0%  **SKVDEEELRNGRKELIVTEIPYEVNKSSLVKRIDELRADKKVDGIVEVRDETDRTGLRIAIELKKDVNSEAIKNFLYKNS**

3 P912 100.0% 100.0%  **SKVDEEELRNGRKELIVTEIPYEVNKSSLVKRIDELRADKKVDGIVEVRDETDRTGLRIAIELKKDVNSEAIKNFLYKNS**

4 NGS-ED-1001 92.5% 100.0%  **SKVDEEELRNGRKELIVTEIPYEVNKSSLVKRIDELRADKKVDGIVEVRDETDRTGLRIAIELKKDVNSEAIKNFLYKNS**

5 1DB1 92.5% 100.0%  **SKVDEEELRNGRKELIVTEIPYEVNKSSLVKRIDELRADKKVDGIVEVRDETDRTGLRIAIELKKDVNSEAIKNFLYKNS**

6 MGYG-HGUT-02301 92.5% 100.0%  **SKVDEEELRNGRKELIVTEIPYEVNKSSLVKRIDELRADKKVDGIVEVRDETDRTGLRIAIELKKDVNSEAIKNFLYKNS**

7 IIF4SW-P1 100.0% 100.0%  **SKVDEEELRNGRKELIVTEIPYEVNKSSLVKRIDELRADKKVDGIVEVRDETDRTGLRIAIELKKDVNSEAIKNFLYKNS**

8 19428wF1_P912 92.5% 100.0%  **SKVDEEELRNGRKELIVTEIPYEVNKSSLVKRIDELRADKKVDGIVEVRDETDRTGLRIAIELKKDVNSEAIKNFLYKNS**

9 R5992 92.5% 100.0%  **SKVDEEELRNGRKELIVTEIPYEVNKSSLVKRIDELRADKKVDGIVEVRDETDRTGLRIAIELKKDVNSEAIKNFLYKNS**

10 R5990 92.5% 100.0%  **SKVDEEELRNGRKELIVTEIPYEVNKSSLVKRIDELRADKKVDGIVEVRDETDRTGLRIAIELKKDVNSEAIKNFLYKNS**

11 R5991 92.5% 100.0%  **SKVDEEELRNGRKELIVTEIPYEVNKSSLVKRIDELRADKKVDGIVEVRDETDRTGLRIAIELKKDVNSEAIKNFLYKNS**

12 R6028 92.5% 99.9%  **SKVDDEELRNGRKELIVTEIPYEVNKSSLVKRIDELRADKKVDGIVEVRDETDRTGLRIAIELKKDVNSEAIKNFLYKNS**

13 R6027 92.5% 99.9%  **SKVDDEELRNGRKELIVTEIPYEVNKSSLVKRIDELRADKKVDGIVEVRDETDRTGLRIAIELKKDVNSEAIKNFLYKNS**

14 R6024 100.0% 99.9%  **SKVDDEELRNGRKELIVTEIPYEVNKSSLVKRIDELRADKKVDGIVEVRDETDRTGLRIAIELKKDVNSEAIKNFLYKNS**

15 L2_057 100.0% 100.0%  **SKVDEEELRNGRKELIVTEIPYEVNKSSLVKRIDELRADKKVDGIVEVRDETDRTGLRIAIELKKDVNSEAIKNFLYKNS**

16 Cap100.1 100.0% 100.0%  **SKVDEEELRNGRKELIVTEIPYEVNKSSLVKRIDELRADKKVDGIVEVRDETDRTGLRIAIELKKDVNSEAIKNFLYKNS**

17 Ani-LG-057 100.0% 100.0%  **SKVDEEELRNGRKELIVTEIPYEVNKSSLVKRIDELRADKKVDGIVEVRDETDRTGLRIAIELKKDVNSEAIKNFLYKNS**

18 Cap9.2 92.5% 100.0%  **SKVDEEELRNGRKELIVTEIPYEVNKSSLVKRIDELRADKKVDGIVEVRDETDRTGLRIAIELKKDVNSEAIKNFLYKNS**

19 Cap10.1 92.5% 100.0%  **SKVDEEELRNGRKELIVTEIPYEVNKSSLVKRIDELRADKKVDGIVEVRDETDRTGLRIAIELKKDVNSEAIKNFLYKNS**

20 Cap9.1 100.0% 100.0%  **SKVDEEELRNGRKELIVTEIPYEVNKSSLVKRIDELRADKKVDGIVEVRDETDRTGLRIAIELKKDVNSEAIKNFLYKNS**

21 Cap10.2 100.0% 100.0%  **SKVDEEELRNGRKELIVTEIPYEVNKSSLVKRIDELRADKKVDGIVEVRDETDRTGLRIAIELKKDVNSEAIKNFLYKNS**

22 acrok 100.0% 100.0%  **SKVDEEELRNGRKELIVTEIPYEVNKSSLVKRIDELRADKKVDGIVEVRDETDRTGLRIAIELKKDVNSEAIKNFLYKNS**

23 acroj 92.5% 100.0%  **SKVDEEELRNGRKELIVTEIPYEVNKSSLVKRIDELRADKKVDGIVEVRDETDRTGLRIAIELKKDVNSEAIKNFLYKNS**

24 acror 92.5% 100.0%  **SKVDEEELRNGRKELIVTEIPYEVNKSSLVKRIDELRADKKVDGIVEVRDETDRTGLRIAIELKKDVNSEAIKNFLYKNS**

25 NJ6 100.0% 100.0%  **SKVDEEELRNGRKELIVTEIPYEVNKSSLVKRIDELRADKKVDGIVEVRDETDRTGLRIAIELKKDVNSEAIKNFLYKNS**

26 EYE_450 100.0% 100.0%  **SKVDEEELRNGRKELIVTEIPYEVNKSSLVKRIDELRADKKVDGIVEVRDETDRTGLRIAIELKKDVNSEAIKNFLYKNS**

27 EYE_117 92.5% 100.0%  **SKVDEEELRNGRKELIVTEIPYEVNKSSLVKRIDELRADKKVDGIVEVRDETDRTGLRIAIELKKDVNSEAIKNFLYKNS**

28 EYE_410 92.5% 100.0%  **SKVDEEELRNGRKELIVTEIPYEVNKSSLVKRIDELRADKKVDGIVEVRDETDRTGLRIAIELKKDVNSEAIKNFLYKNS**

29 EYE_411 92.5% 100.0%  **SKVDEEELRNGRKELIVTEIPYEVNKSSLVKRIDELRADKKVDGIVEVRDETDRTGLRIAIELKKDVNSEAIKNFLYKNS**

30 MERTA18 92.5% 100.0%  **SKVDEEELRNGRKELIVTEIPYEVNKSSLVKRIDELRADKKVDGIVEVRDETDRTGLRIAIELKKDVNSEAIKNFLYKNS**

31 FAIRING19B-1.2 100.0% 100.0%  **SKVDEEELRNGRKELIVTEIPYEVNKSSLVKRIDELRADKKVDGIVEVRDETDRTGLRIAIELKKDVNSEAIKNFLYKNS**

32 FDAARGOS_151 92.5% 99.7%  **SKVDEEELRNGRKELIVTEIPYEVNKSSLVKRIDELRADKKVDGIVEVRDETDRTGLRIAIELKKDVNSEAIKNFLYKNS**

33 SNUC5989 100.0% 100.0%  **SKVDEEELRNGRKELIVTEIPYEVNKSSLVKRIDELRADKKVDGIVEVRDETDRTGLRIAIELKKDVNSEAIKNFLYKNS**

34 SNUC3412 100.0% 100.0%  **SKVDEEELRNGRKELIVTEIPYEVNKSSLVKRIDELRADKKVDGIVEVRDETDRTGLRIAIELKKDVNSEAIKNFLYKNS**

35 OM08-17AT 100.0% 100.0%  **SKVDEEELRNGRKELIVTEIPYEVNKSSLVKRIDELRADKKVDGIVEVRDETDRTGLRIAIELKKDVNSEAIKNFLYKNS**

36 SWO 100.0% 100.0%  **SKVDEEELRNGRKELIVTEIPYEVNKSSLVKRIDELRADKKVDGIVEVRDETDRTGLRIAIELKKDVNSEAIKNFLYKNS**

consensus/100%  **SKVD-EELRNGRKELIVTEIPYEVNKSSLVKRIDELRADKKVDGIVEVRDETDRTGLRIAIELKKDVNSEAIKNFLYKNS**

consensus/90%  **SKVDEEELRNGRKELIVTEIPYEVNKSSLVKRIDELRADKKVDGIVEVRDETDRTGLRIAIELKKDVNSEAIKNFLYKNS**

consensus/80%  **SKVDEEELRNGRKELIVTEIPYEVNKSSLVKRIDELRADKKVDGIVEVRDETDRTGLRIAIELKKDVNSEAIKNFLYKNS**

consensus/70%  **SKVDEEELRNGRKELIVTEIPYEVNKSSLVKRIDELRADKKVDGIVEVRDETDRTGLRIAIELKKDVNSEAIKNFLYKNS**

cov pid **321**  **. . : . . . . 4** **400**

1 29AM 100.0% 100.0%  **DLQISYNFNMVAISDGRPKLMGIKQIIDSYINHQIEVVANRTKFDLEHAEKRMHIVEGLMKALSILDEVIALIRNSKNKK**

2 TRPF4 100.0% 100.0%  **DLQISYNFNMVAISDGRPKLMGIKQIIDSYINHQIEVVANRTKFDLEHAEKRMHIVEGLMKALSILDEVIALIRNSKNKK**

3 P912 100.0% 100.0%  **DLQISYNFNMVAISDGRPKLMGIKQIIDSYINHQIEVVANRTKFDLEHAEKRMHIVEGLMKALSILDEVIALIRNSKNKK**

4 NGS-ED-1001 92.5% 100.0%  **DLQISYNFNMVAISDGRPKLMGIKQIIDSYINHQIEVVANRTKFDLEHAEKRMHIVEGLMKALSILDEVIALIRNSKNKK**

5 1DB1 92.5% 100.0%  **DLQISYNFNMVAISDGRPKLMGIKQIIDSYINHQIEVVANRTKFDLEHAEKRMHIVEGLMKALSILDEVIALIRNSKNKK**

6 MGYG-HGUT-02301 92.5% 100.0%  **DLQISYNFNMVAISDGRPKLMGIKQIIDSYINHQIEVVANRTKFDLEHAEKRMHIVEGLMKALSILDEVIALIRNSKNKK**

7 IIF4SW-P1 100.0% 100.0%  **DLQISYNFNMVAISDGRPKLMGIKQIIDSYINHQIEVVANRTKFDLEHAEKRMHIVEGLMKALSILDEVIALIRNSKNKK**

8 19428wF1_P912 92.5% 100.0%  **DLQISYNFNMVAISDGRPKLMGIKQIIDSYINHQIEVVANRTKFDLEHAEKRMHIVEGLMKALSILDEVIALIRNSKNKK**

9 R5992 92.5% 100.0%  **DLQISYNFNMVAISDGRPKLMGIKQIIDSYINHQIEVVANRTKFDLEHAEKRMHIVEGLMKALSILDEVIALIRNSKNKK**

10 R5990 92.5% 100.0%  **DLQISYNFNMVAISDGRPKLMGIKQIIDSYINHQIEVVANRTKFDLEHAEKRMHIVEGLMKALSILDEVIALIRNSKNKK**

11 R5991 92.5% 100.0%  **DLQISYNFNMVAISDGRPKLMGIKQIIDSYINHQIEVVANRTKFDLEHAEKRMHIVEGLMKALSILDEVIALIRNSKNKK**

12 R6028 92.5% 99.9%  **DLQISYNFNMVAISDGRPKLMGIKQIIDSYINHQIEVVANRTKFDLEHAEKRMHIVEGLMKALSILDEVIALIRNSKNKK**

13 R6027 92.5% 99.9%  **DLQISYNFNMVAISDGRPKLMGIKQIIDSYINHQIEVVANRTKFDLEHAEKRMHIVEGLMKALSILDEVIALIRNSKNKK**

14 R6024 100.0% 99.9%  **DLQISYNFNMVAISDGRPKLMGIKQIIDSYINHQIEVVANRTKFDLEHAEKRMHIVEGLMKALSILDEVIALIRNSKNKK**

15 L2_057 100.0% 100.0%  **DLQISYNFNMVAISDGRPKLMGIKQIIDSYINHQIEVVANRTKFDLEHAEKRMHIVEGLMKALSILDEVIALIRNSKNKK**

16 Cap100.1 100.0% 100.0%  **DLQISYNFNMVAISDGRPKLMGIKQIIDSYINHQIEVVANRTKFDLEHAEKRMHIVEGLMKALSILDEVIALIRNSKNKK**

17 Ani-LG-057 100.0% 100.0%  **DLQISYNFNMVAISDGRPKLMGIKQIIDSYINHQIEVVANRTKFDLEHAEKRMHIVEGLMKALSILDEVIALIRNSKNKK**

18 Cap9.2 92.5% 100.0%  **DLQISYNFNMVAISDGRPKLMGIKQIIDSYINHQIEVVANRTKFDLEHAEKRMHIVEGLMKALSILDEVIALIRNSKNKK**

19 Cap10.1 92.5% 100.0%  **DLQISYNFNMVAISDGRPKLMGIKQIIDSYINHQIEVVANRTKFDLEHAEKRMHIVEGLMKALSILDEVIALIRNSKNKK**

20 Cap9.1 100.0% 100.0%  **DLQISYNFNMVAISDGRPKLMGIKQIIDSYINHQIEVVANRTKFDLEHAEKRMHIVEGLMKALSILDEVIALIRNSKNKK**

21 Cap10.2 100.0% 100.0%  **DLQISYNFNMVAISDGRPKLMGIKQIIDSYINHQIEVVANRTKFDLEHAEKRMHIVEGLMKALSILDEVIALIRNSKNKK**

22 acrok 100.0% 100.0%  **DLQISYNFNMVAISDGRPKLMGIKQIIDSYINHQIEVVANRTKFDLEHAEKRMHIVEGLMKALSILDEVIALIRNSKNKK**

23 acroj 92.5% 100.0%  **DLQISYNFNMVAISDGRPKLMGIKQIIDSYINHQIEVVANRTKFDLEHAEKRMHIVEGLMKALSILDEVIALIRNSKNKK**

24 acror 92.5% 100.0%  **DLQISYNFNMVAISDGRPKLMGIKQIIDSYINHQIEVVANRTKFDLEHAEKRMHIVEGLMKALSILDEVIALIRNSKNKK**

25 NJ6 100.0% 100.0%  **DLQISYNFNMVAISDGRPKLMGIKQIIDSYINHQIEVVANRTKFDLEHAEKRMHIVEGLMKALSILDEVIALIRNSKNKK**

26 EYE_450 100.0% 100.0%  **DLQISYNFNMVAISDGRPKLMGIKQIIDSYINHQIEVVANRTKFDLEHAEKRMHIVEGLMKALSILDEVIALIRNSKNKK**

27 EYE_117 92.5% 100.0%  **DLQISYNFNMVAISDGRPKLMGIKQIIDSYINHQIEVVANRTKFDLEHAEKRMHIVEGLMKALSILDEVIALIRNSKNKK**

28 EYE_410 92.5% 100.0%  **DLQISYNFNMVAISDGRPKLMGIKQIIDSYINHQIEVVANRTKFDLEHAEKRMHIVEGLMKALSILDEVIALIRNSKNKK**

29 EYE_411 92.5% 100.0%  **DLQISYNFNMVAISDGRPKLMGIKQIIDSYINHQIEVVANRTKFDLEHAEKRMHIVEGLMKALSILDEVIALIRNSKNKK**

30 MERTA18 92.5% 100.0%  **DLQISYNFNMVAISDGRPKLMGIKQIIDSYINHQIEVVANRTKFDLEHAEKRMHIVEGLMKALSILDEVIALIRNSKNKK**

31 FAIRING19B-1.2 100.0% 100.0%  **DLQISYNFNMVAISDGRPKLMGIKQIIDSYINHQIEVVANRTKFDLEHAEKRMHIVEGLMKALSILDEVIALIRNSKNKK**

32 FDAARGOS_151 92.5% 99.7%  **DLQISYNFNMVAISDGRPKLMGIKQIIDSYINHQIEVVANRTKFDLEHAEKRMHIVEGLMKALSILDEVIALIRNSKNKK**

33 SNUC5989 100.0% 100.0%  **DLQISYNFNMVAISDGRPKLMGIKQIIDSYINHQIEVVANRTKFDLEHAEKRMHIVEGLMKALSILDEVIALIRNSKNKK**

34 SNUC3412 100.0% 100.0%  **DLQISYNFNMVAISDGRPKLMGIKQIIDSYINHQIEVVANRTKFDLEHAEKRMHIVEGLMKALSILDEVIALIRNSKNKK**

35 OM08-17AT 100.0% 100.0%  **DLQISYNFNMVAISDGRPKLMGIKQIIDSYINHQIEVVANRTKFDLEHAEKRMHIVEGLMKALSILDEVIALIRNSKNKK**

36 SWO 100.0% 100.0%  **DLQISYNFNMVAISDGRPKLMGIKQIIDSYINHQIEVVANRTKFDLEHAEKRMHIVEGLMKALSILDEVIALIRNSKNKK**

consensus/100%  **DLQISYNFNMVAISDGRPKLMGIKQIIDSYINHQIEVVANRTKFDLEHAEKRMHIVEGLMKALSILDEVIALIRNSKNKK**

consensus/90%  **DLQISYNFNMVAISDGRPKLMGIKQIIDSYINHQIEVVANRTKFDLEHAEKRMHIVEGLMKALSILDEVIALIRNSKNKK**

consensus/80%  **DLQISYNFNMVAISDGRPKLMGIKQIIDSYINHQIEVVANRTKFDLEHAEKRMHIVEGLMKALSILDEVIALIRNSKNKK**

consensus/70%  **DLQISYNFNMVAISDGRPKLMGIKQIIDSYINHQIEVVANRTKFDLEHAEKRMHIVEGLMKALSILDEVIALIRNSKNKK**

cov pid **401**  **. . . . : . . .** **480**

1 29AM 100.0% 100.0%  **DAKDNLVAEFDFTEAQAEAIVMLQLYRLTNTDIVALEQEHDELSNLIKDLRHILDDHDALLNVIKNELTEIRKKFKTDRL**

2 TRPF4 100.0% 100.0%  **DAKDNLVAEFDFTEAQAEAIVMLQLYRLTNTDIVALEQEHDELSNLIKDLRHILDDHDALLNVIKNELTEIRKKFKTDRL**

3 P912 100.0% 100.0%  **DAKDNLVAEFDFTEAQAEAIVMLQLYRLTNTDIVALEQEHDELSNLIKDLRHILDDHDALLNVIKNELTEIRKKFKTDRL**

4 NGS-ED-1001 92.5% 100.0%  **DAKDNLVAEFDFTEAQAEAIVMLQLYRLTNTDIVALEQEHDELSNLIKDLRHILDDHDALLNVIKNELTEIRKKFKTDRL**

5 1DB1 92.5% 100.0%  **DAKDNLVAEFDFTEAQAEAIVMLQLYRLTNTDIVALEQEHDELSNLIKDLRHILDDHDALLNVIKNELTEIRKKFKTDRL**

6 MGYG-HGUT-02301 92.5% 100.0%  **DAKDNLVAEFDFTEAQAEAIVMLQLYRLTNTDIVALEQEHDELSNLIKDLRHILDDHDALLNVIKNELTEIRKKFKTDRL**

7 IIF4SW-P1 100.0% 100.0%  **DAKDNLVAEFDFTEAQAEAIVMLQLYRLTNTDIVALEQEHDELSNLIKDLRHILDDHDALLNVIKNELTEIRKKFKTDRL**

8 19428wF1_P912 92.5% 100.0%  **DAKDNLVAEFDFTEAQAEAIVMLQLYRLTNTDIVALEQEHDELSNLIKDLRHILDDHDALLNVIKNELTEIRKKFKTDRL**

9 R5992 92.5% 100.0%  **DAKDNLVAEFDFTEAQAEAIVMLQLYRLTNTDIVALEQEHDELSNLIKDLRHILDDHDALLNVIKNELTEIRKKFKTDRL**

10 R5990 92.5% 100.0%  **DAKDNLVAEFDFTEAQAEAIVMLQLYRLTNTDIVALEQEHDELSNLIKDLRHILDDHDALLNVIKNELTEIRKKFKTDRL**

11 R5991 92.5% 100.0%  **DAKDNLVAEFDFTEAQAEAIVMLQLYRLTNTDIVALEQEHDELSNLIKDLRHILDDHDALLNVIKNELTEIRKKFKTDRL**

12 R6028 92.5% 99.9%  **DAKDNLVAEFDFTEAQAEAIVMLQLYRLTNTDIVALEQEHDELSNLIKDLRHILDDHDALLNVIKNELTEIRKKFKTDRL**

13 R6027 92.5% 99.9%  **DAKDNLVAEFDFTEAQAEAIVMLQLYRLTNTDIVALEQEHDELSNLIKDLRHILDDHDALLNVIKNELTEIRKKFKTDRL**

14 R6024 100.0% 99.9%  **DAKDNLVAEFDFTEAQAEAIVMLQLYRLTNTDIVALEQEHDELSNLIKDLRHILDDHDALLNVIKNELTEIRKKFKTDRL**

15 L2_057 100.0% 100.0%  **DAKDNLVAEFDFTEAQAEAIVMLQLYRLTNTDIVALEQEHDELSNLIKDLRHILDDHDALLNVIKNELTEIRKKFKTDRL**

16 Cap100.1 100.0% 100.0%  **DAKDNLVAEFDFTEAQAEAIVMLQLYRLTNTDIVALEQEHDELSNLIKDLRHILDDHDALLNVIKNELTEIRKKFKTDRL**

17 Ani-LG-057 100.0% 100.0%  **DAKDNLVAEFDFTEAQAEAIVMLQLYRLTNTDIVALEQEHDELSNLIKDLRHILDDHDALLNVIKNELTEIRKKFKTDRL**

18 Cap9.2 92.5% 100.0%  **DAKDNLVAEFDFTEAQAEAIVMLQLYRLTNTDIVALEQEHDELSNLIKDLRHILDDHDALLNVIKNELTEIRKKFKTDRL**

19 Cap10.1 92.5% 100.0%  **DAKDNLVAEFDFTEAQAEAIVMLQLYRLTNTDIVALEQEHDELSNLIKDLRHILDDHDALLNVIKNELTEIRKKFKTDRL**

20 Cap9.1 100.0% 100.0%  **DAKDNLVAEFDFTEAQAEAIVMLQLYRLTNTDIVALEQEHDELSNLIKDLRHILDDHDALLNVIKNELTEIRKKFKTDRL**

21 Cap10.2 100.0% 100.0%  **DAKDNLVAEFDFTEAQAEAIVMLQLYRLTNTDIVALEQEHDELSNLIKDLRHILDDHDALLNVIKNELTEIRKKFKTDRL**

22 acrok 100.0% 100.0%  **DAKDNLVAEFDFTEAQAEAIVMLQLYRLTNTDIVALEQEHDELSNLIKDLRHILDDHDALLNVIKNELTEIRKKFKTDRL**

23 acroj 92.5% 100.0%  **DAKDNLVAEFDFTEAQAEAIVMLQLYRLTNTDIVALEQEHDELSNLIKDLRHILDDHDALLNVIKNELTEIRKKFKTDRL**

24 acror 92.5% 100.0%  **DAKDNLVAEFDFTEAQAEAIVMLQLYRLTNTDIVALEQEHDELSNLIKDLRHILDDHDALLNVIKNELTEIRKKFKTDRL**

25 NJ6 100.0% 100.0%  **DAKDNLVAEFDFTEAQAEAIVMLQLYRLTNTDIVALEQEHDELSNLIKDLRHILDDHDALLNVIKNELTEIRKKFKTDRL**

26 EYE_450 100.0% 100.0%  **DAKDNLVAEFDFTEAQAEAIVMLQLYRLTNTDIVALEQEHDELSNLIKDLRHILDDHDALLNVIKNELTEIRKKFKTDRL**

27 EYE_117 92.5% 100.0%  **DAKDNLVAEFDFTEAQAEAIVMLQLYRLTNTDIVALEQEHDELSNLIKDLRHILDDHDALLNVIKNELTEIRKKFKTDRL**

28 EYE_410 92.5% 100.0%  **DAKDNLVAEFDFTEAQAEAIVMLQLYRLTNTDIVALEQEHDELSNLIKDLRHILDDHDALLNVIKNELTEIRKKFKTDRL**

29 EYE_411 92.5% 100.0%  **DAKDNLVAEFDFTEAQAEAIVMLQLYRLTNTDIVALEQEHDELSNLIKDLRHILDDHDALLNVIKNELTEIRKKFKTDRL**

30 MERTA18 92.5% 100.0%  **DAKDNLVAEFDFTEAQAEAIVMLQLYRLTNTDIVALEQEHDELSNLIKDLRHILDDHDALLNVIKNELTEIRKKFKTDRL**

31 FAIRING19B-1.2 100.0% 100.0%  **DAKDNLVAEFDFTEAQAEAIVMLQLYRLTNTDIVALEQEHDELSNLIKDLRHILDDHDALLNVIKNELTEIRKKFKTDRL**

32 FDAARGOS_151 92.5% 99.7%  **DAKDNLVAEFDFTEAQAEAIVMLQLYRLTNTDIVALEQEHDELSNLIKDLRHILDDHDALLNVIKNELTEIRKKFKTDRL**

33 SNUC5989 100.0% 100.0%  **DAKDNLVAEFDFTEAQAEAIVMLQLYRLTNTDIVALEQEHDELSNLIKDLRHILDDHDALLNVIKNELTEIRKKFKTDRL**

34 SNUC3412 100.0% 100.0%  **DAKDNLVAEFDFTEAQAEAIVMLQLYRLTNTDIVALEQEHDELSNLIKDLRHILDDHDALLNVIKNELTEIRKKFKTDRL**

35 OM08-17AT 100.0% 100.0%  **DAKDNLVAEFDFTEAQAEAIVMLQLYRLTNTDIVALEQEHDELSNLIKDLRHILDDHDALLNVIKNELTEIRKKFKTDRL**

36 SWO 100.0% 100.0%  **DAKDNLVAEFDFTEAQAEAIVMLQLYRLTNTDIVALEQEHDELSNLIKDLRHILDDHDALLNVIKNELTEIRKKFKTDRL**

consensus/100%  **DAKDNLVAEFDFTEAQAEAIVMLQLYRLTNTDIVALEQEHDELSNLIKDLRHILDDHDALLNVIKNELTEIRKKFKTDRL**

consensus/90%  **DAKDNLVAEFDFTEAQAEAIVMLQLYRLTNTDIVALEQEHDELSNLIKDLRHILDDHDALLNVIKNELTEIRKKFKTDRL**

consensus/80%  **DAKDNLVAEFDFTEAQAEAIVMLQLYRLTNTDIVALEQEHDELSNLIKDLRHILDDHDALLNVIKNELTEIRKKFKTDRL**

consensus/70%  **DAKDNLVAEFDFTEAQAEAIVMLQLYRLTNTDIVALEQEHDELSNLIKDLRHILDDHDALLNVIKNELTEIRKKFKTDRL**

cov pid **481**  **. 5 . . . . : .** **560**

1 29AM 100.0% 100.0%  **STIEAEISEIKIDKEVMVPSEEVVLSLTRHGYIKRTTTRSFNASGVSEVGLKDGDSLLKYLDVNTQDTALVFTNKGRYLF**

2 TRPF4 100.0% 100.0%  **STIEAEISEIKIDKEVMVPSEEVVLSLTRHGYIKRTTTRSFNASGVSEVGLKDGDSLLKYLDVNTQDTALVFTNKGRYLF**

3 P912 100.0% 100.0%  **STIEAEISEIKIDKEVMVPSEEVVLSLTRHGYIKRTTTRSFNASGVSEVGLKDGDSLLKYLDVNTQDTALVFTNKGRYLF**

4 NGS-ED-1001 92.5% 100.0%  **STIEAEISEIKIDKEVMVPSEEVVLSLTRHGYIKRTTTRSFNASGVSEVGLKDGDSLLKYLDVNTQDTALVFTNKGRYLF**

5 1DB1 92.5% 100.0%  **STIEAEISEIKIDKEVMVPSEEVVLSLTRHGYIKRTTTRSFNASGVSEVGLKDGDSLLKYLDVNTQDTALVFTNKGRYLF**

6 MGYG-HGUT-02301 92.5% 100.0%  **STIEAEISEIKIDKEVMVPSEEVVLSLTRHGYIKRTTTRSFNASGVSEVGLKDGDSLLKYLDVNTQDTALVFTNKGRYLF**

7 IIF4SW-P1 100.0% 100.0%  **STIEAEISEIKIDKEVMVPSEEVVLSLTRHGYIKRTTTRSFNASGVSEVGLKDGDSLLKYLDVNTQDTALVFTNKGRYLF**

8 19428wF1_P912 92.5% 100.0%  **STIEAEISEIKIDKEVMVPSEEVVLSLTRHGYIKRTTTRSFNASGVSEVGLKDGDSLLKYLDVNTQDTALVFTNKGRYLF**

9 R5992 92.5% 100.0%  **STIEAEISEIKIDKEVMVPSEEVVLSLTRHGYIKRTTTRSFNASGVSEVGLKDGDSLLKYLDVNTQDTALVFTNKGRYLF**

10 R5990 92.5% 100.0%  **STIEAEISEIKIDKEVMVPSEEVVLSLTRHGYIKRTTTRSFNASGVSEVGLKDGDSLLKYLDVNTQDTALVFTNKGRYLF**

11 R5991 92.5% 100.0%  **STIEAEISEIKIDKEVMVPSEEVVLSLTRHGYIKRTTTRSFNASGVSEVGLKDGDSLLKYLDVNTQDTALVFTNKGRYLF**

12 R6028 92.5% 99.9%  **STIEAEISEIKIDKEVMVPSEEVVLSLTRHGYIKRTTTRSFNASGVSEVGLKDGDSLLKYLDVNTQDTALVFTNKGRYLF**

13 R6027 92.5% 99.9%  **STIEAEISEIKIDKEVMVPSEEVVLSLTRHGYIKRTTTRSFNASGVSEVGLKDGDSLLKYLDVNTQDTALVFTNKGRYLF**

14 R6024 100.0% 99.9%  **STIEAEISEIKIDKEVMVPSEEVVLSLTRHGYIKRTTTRSFNASGVSEVGLKDGDSLLKYLDVNTQDTALVFTNKGRYLF**

15 L2_057 100.0% 100.0%  **STIEAEISEIKIDKEVMVPSEEVVLSLTRHGYIKRTTTRSFNASGVSEVGLKDGDSLLKYLDVNTQDTALVFTNKGRYLF**

16 Cap100.1 100.0% 100.0%  **STIEAEISEIKIDKEVMVPSEEVVLSLTRHGYIKRTTTRSFNASGVSEVGLKDGDSLLKYLDVNTQDTALVFTNKGRYLF**

17 Ani-LG-057 100.0% 100.0%  **STIEAEISEIKIDKEVMVPSEEVVLSLTRHGYIKRTTTRSFNASGVSEVGLKDGDSLLKYLDVNTQDTALVFTNKGRYLF**

18 Cap9.2 92.5% 100.0%  **STIEAEISEIKIDKEVMVPSEEVVLSLTRHGYIKRTTTRSFNASGVSEVGLKDGDSLLKYLDVNTQDTALVFTNKGRYLF**

19 Cap10.1 92.5% 100.0%  **STIEAEISEIKIDKEVMVPSEEVVLSLTRHGYIKRTTTRSFNASGVSEVGLKDGDSLLKYLDVNTQDTALVFTNKGRYLF**

20 Cap9.1 100.0% 100.0%  **STIEAEISEIKIDKEVMVPSEEVVLSLTRHGYIKRTTTRSFNASGVSEVGLKDGDSLLKYLDVNTQDTALVFTNKGRYLF**

21 Cap10.2 100.0% 100.0%  **STIEAEISEIKIDKEVMVPSEEVVLSLTRHGYIKRTTTRSFNASGVSEVGLKDGDSLLKYLDVNTQDTALVFTNKGRYLF**

22 acrok 100.0% 100.0%  **STIEAEISEIKIDKEVMVPSEEVVLSLTRHGYIKRTTTRSFNASGVSEVGLKDGDSLLKYLDVNTQDTALVFTNKGRYLF**

23 acroj 92.5% 100.0%  **STIEAEISEIKIDKEVMVPSEEVVLSLTRHGYIKRTTTRSFNASGVSEVGLKDGDSLLKYLDVNTQDTALVFTNKGRYLF**

24 acror 92.5% 100.0%  **STIEAEISEIKIDKEVMVPSEEVVLSLTRHGYIKRTTTRSFNASGVSEVGLKDGDSLLKYLDVNTQDTALVFTNKGRYLF**

25 NJ6 100.0% 100.0%  **STIEAEISEIKIDKEVMVPSEEVVLSLTRHGYIKRTTTRSFNASGVSEVGLKDGDSLLKYLDVNTQDTALVFTNKGRYLF**

26 EYE_450 100.0% 100.0%  **STIEAEISEIKIDKEVMVPSEEVVLSLTRHGYIKRTTTRSFNASGVSEVGLKDGDSLLKYLDVNTQDTALVFTNKGRYLF**

27 EYE_117 92.5% 100.0%  **STIEAEISEIKIDKEVMVPSEEVVLSLTRHGYIKRTTTRSFNASGVSEVGLKDGDSLLKYLDVNTQDTALVFTNKGRYLF**

28 EYE_410 92.5% 100.0%  **STIEAEISEIKIDKEVMVPSEEVVLSLTRHGYIKRTTTRSFNASGVSEVGLKDGDSLLKYLDVNTQDTALVFTNKGRYLF**

29 EYE_411 92.5% 100.0%  **STIEAEISEIKIDKEVMVPSEEVVLSLTRHGYIKRTTTRSFNASGVSEVGLKDGDSLLKYLDVNTQDTALVFTNKGRYLF**

30 MERTA18 92.5% 100.0%  **STIEAEISEIKIDKEVMVPSEEVVLSLTRHGYIKRTTTRSFNASGVSEVGLKDGDSLLKYLDVNTQDTALVFTNKGRYLF**

31 FAIRING19B-1.2 100.0% 100.0%  **STIEAEISEIKIDKEVMVPSEEVVLSLTRHGYIKRTTTRSFNASGVSEVGLKDGDSLLKYLDVNTQDTALVFTNKGRYLF**

32 FDAARGOS_151 92.5% 99.7%  **STIEAEISEIKIDKEVMVPSEEVVLSLTRHGYIKRTTTRSFNASGVSEVGLKDGDSLLKYLDVNTQDTALVFTNKGRYLF**

33 SNUC5989 100.0% 100.0%  **STIEAEISEIKIDKEVMVPSEEVVLSLTRHGYIKRTTTRSFNASGVSEVGLKDGDSLLKYLDVNTQDTALVFTNKGRYLF**

34 SNUC3412 100.0% 100.0%  **STIEAEISEIKIDKEVMVPSEEVVLSLTRHGYIKRTTTRSFNASGVSEVGLKDGDSLLKYLDVNTQDTALVFTNKGRYLF**

35 OM08-17AT 100.0% 100.0%  **STIEAEISEIKIDKEVMVPSEEVVLSLTRHGYIKRTTTRSFNASGVSEVGLKDGDSLLKYLDVNTQDTALVFTNKGRYLF**

36 SWO 100.0% 100.0%  **STIEAEISEIKIDKEVMVPSEEVVLSLTRHGYIKRTTTRSFNASGVSEVGLKDGDSLLKYLDVNTQDTALVFTNKGRYLF**

consensus/100%  **STIEAEISEIKIDKEVMVPSEEVVLSLTRHGYIKRTTTRSFNASGVSEVGLKDGDSLLKYLDVNTQDTALVFTNKGRYLF**

consensus/90%  **STIEAEISEIKIDKEVMVPSEEVVLSLTRHGYIKRTTTRSFNASGVSEVGLKDGDSLLKYLDVNTQDTALVFTNKGRYLF**

consensus/80%  **STIEAEISEIKIDKEVMVPSEEVVLSLTRHGYIKRTTTRSFNASGVSEVGLKDGDSLLKYLDVNTQDTALVFTNKGRYLF**

consensus/70%  **STIEAEISEIKIDKEVMVPSEEVVLSLTRHGYIKRTTTRSFNASGVSEVGLKDGDSLLKYLDVNTQDTALVFTNKGRYLF**

cov pid **561**  **. . . 6 . . . .** **640**

1 29AM 100.0% 100.0%  **IPVHKLAEIRWKELGQHVSQIVSIDEDEQVIDVFNEKDFSQHEAFYIMATRNGMIKKSSVPQFKTTRYNKPLIAMKVKDQ**

2 TRPF4 100.0% 100.0%  **IPVHKLAEIRWKELGQHVSQIVSIDEDEQVIDVFNEKDFSQHEAFYIMATRNGMIKKSSVPQFKTTRYNKPLIAMKVKDQ**

3 P912 100.0% 100.0%  **IPVHKLAEIRWKELGQHVSQIVSIDEDEQVIDVFNEKDFSQHEAFYIMATRNGMIKKSSVPQFKTTRYNKPLIAMKVKDQ**

4 NGS-ED-1001 92.5% 100.0%  **IPVHKLAEIRWKELGQHVSQIVSIDEDEQVIDVFNEKDFSQHEAFYIMATRNGMIKKSSVPQFKTTRYNKPLIAMKVKDQ**

5 1DB1 92.5% 100.0%  **IPVHKLAEIRWKELGQHVSQIVSIDEDEQVIDVFNEKDFSQHEAFYIMATRNGMIKKSSVPQFKTTRYNKPLIAMKVKDQ**

6 MGYG-HGUT-02301 92.5% 100.0%  **IPVHKLAEIRWKELGQHVSQIVSIDEDEQVIDVFNEKDFSQHEAFYIMATRNGMIKKSSVPQFKTTRYNKPLIAMKVKDQ**

7 IIF4SW-P1 100.0% 100.0%  **IPVHKLAEIRWKELGQHVSQIVSIDEDEQVIDVFNEKDFSQHEAFYIMATRNGMIKKSSVPQFKTTRYNKPLIAMKVKDQ**

8 19428wF1_P912 92.5% 100.0%  **IPVHKLAEIRWKELGQHVSQIVSIDEDEQVIDVFNEKDFSQHEAFYIMATRNGMIKKSSVPQFKTTRYNKPLIAMKVKDQ**

9 R5992 92.5% 100.0%  **IPVHKLAEIRWKELGQHVSQIVSIDEDEQVIDVFNEKDFSQHEAFYIMATRNGMIKKSSVPQFKTTRYNKPLIAMKVKDQ**

10 R5990 92.5% 100.0%  **IPVHKLAEIRWKELGQHVSQIVSIDEDEQVIDVFNEKDFSQHEAFYIMATRNGMIKKSSVPQFKTTRYNKPLIAMKVKDQ**

11 R5991 92.5% 100.0%  **IPVHKLAEIRWKELGQHVSQIVSIDEDEQVIDVFNEKDFSQHEAFYIMATRNGMIKKSSVPQFKTTRYNKPLIAMKVKDQ**

12 R6028 92.5% 99.9%  **IPVHKLAEIRWKELGQHVSQIVSIDEDEQVIDVFNEKDFSQHEAFYIMATRNGMIKKSSVPQFKTTRYNKPLIAMKVKDQ**

13 R6027 92.5% 99.9%  **IPVHKLAEIRWKELGQHVSQIVSIDEDEQVIDVFNEKDFSQHEAFYIMATRNGMIKKSSVPQFKTTRYNKPLIAMKVKDQ**

14 R6024 100.0% 99.9%  **IPVHKLAEIRWKELGQHVSQIVSIDEDEQVIDVFNEKDFSQHEAFYIMATRNGMIKKSSVPQFKTTRYNKPLIAMKVKDQ**

15 L2_057 100.0% 100.0%  **IPVHKLAEIRWKELGQHVSQIVSIDEDEQVIDVFNEKDFSQHEAFYIMATRNGMIKKSSVPQFKTTRYNKPLIAMKVKDQ**

16 Cap100.1 100.0% 100.0%  **IPVHKLAEIRWKELGQHVSQIVSIDEDEQVIDVFNEKDFSQHEAFYIMATRNGMIKKSSVPQFKTTRYNKPLIAMKVKDQ**

17 Ani-LG-057 100.0% 100.0%  **IPVHKLAEIRWKELGQHVSQIVSIDEDEQVIDVFNEKDFSQHEAFYIMATRNGMIKKSSVPQFKTTRYNKPLIAMKVKDQ**

18 Cap9.2 92.5% 100.0%  **IPVHKLAEIRWKELGQHVSQIVSIDEDEQVIDVFNEKDFSQHEAFYIMATRNGMIKKSSVPQFKTTRYNKPLIAMKVKDQ**

19 Cap10.1 92.5% 100.0%  **IPVHKLAEIRWKELGQHVSQIVSIDEDEQVIDVFNEKDFSQHEAFYIMATRNGMIKKSSVPQFKTTRYNKPLIAMKVKDQ**

20 Cap9.1 100.0% 100.0%  **IPVHKLAEIRWKELGQHVSQIVSIDEDEQVIDVFNEKDFSQHEAFYIMATRNGMIKKSSVPQFKTTRYNKPLIAMKVKDQ**

21 Cap10.2 100.0% 100.0%  **IPVHKLAEIRWKELGQHVSQIVSIDEDEQVIDVFNEKDFSQHEAFYIMATRNGMIKKSSVPQFKTTRYNKPLIAMKVKDQ**

22 acrok 100.0% 100.0%  **IPVHKLAEIRWKELGQHVSQIVSIDEDEQVIDVFNEKDFSQHEAFYIMATRNGMIKKSSVPQFKTTRYNKPLIAMKVKDQ**

23 acroj 92.5% 100.0%  **IPVHKLAEIRWKELGQHVSQIVSIDEDEQVIDVFNEKDFSQHEAFYIMATRNGMIKKSSVPQFKTTRYNKPLIAMKVKDQ**

24 acror 92.5% 100.0%  **IPVHKLAEIRWKELGQHVSQIVSIDEDEQVIDVFNEKDFSQHEAFYIMATRNGMIKKSSVPQFKTTRYNKPLIAMKVKDQ**

25 NJ6 100.0% 100.0%  **IPVHKLAEIRWKELGQHVSQIVSIDEDEQVIDVFNEKDFSQHEAFYIMATRNGMIKKSSVPQFKTTRYNKPLIAMKVKDQ**

26 EYE_450 100.0% 100.0%  **IPVHKLAEIRWKELGQHVSQIVSIDEDEQVIDVFNEKDFSQHEAFYIMATRNGMIKKSSVPQFKTTRYNKPLIAMKVKDQ**

27 EYE_117 92.5% 100.0%  **IPVHKLAEIRWKELGQHVSQIVSIDEDEQVIDVFNEKDFSQHEAFYIMATRNGMIKKSSVPQFKTTRYNKPLIAMKVKDQ**

28 EYE_410 92.5% 100.0%  **IPVHKLAEIRWKELGQHVSQIVSIDEDEQVIDVFNEKDFSQHEAFYIMATRNGMIKKSSVPQFKTTRYNKPLIAMKVKDQ**

29 EYE_411 92.5% 100.0%  **IPVHKLAEIRWKELGQHVSQIVSIDEDEQVIDVFNEKDFSQHEAFYIMATRNGMIKKSSVPQFKTTRYNKPLIAMKVKDQ**

30 MERTA18 92.5% 100.0%  **IPVHKLAEIRWKELGQHVSQIVSIDEDEQVIDVFNEKDFSQHEAFYIMATRNGMIKKSSVPQFKTTRYNKPLIAMKVKDQ**

31 FAIRING19B-1.2 100.0% 100.0%  **IPVHKLAEIRWKELGQHVSQIVSIDEDEQVIDVFNEKDFSQHEAFYIMATRNGMIKKSSVPQFKTTRYNKPLIAMKVKDQ**

32 FDAARGOS_151 92.5% 99.7%  **IPVHKLAEIRWKELGQHVSQIVSIDEDEQVIDVFNEKDFSQHEAFYIMATRNGMIKKSSVPQFKTTRYNKPLIAMKVKDQ**

33 SNUC5989 100.0% 100.0%  **IPVHKLAEIRWKELGQHVSQIVSIDEDEQVIDVFNEKDFSQHEAFYIMATRNGMIKKSSVPQFKTTRYNKPLIAMKVKDQ**

34 SNUC3412 100.0% 100.0%  **IPVHKLAEIRWKELGQHVSQIVSIDEDEQVIDVFNEKDFSQHEAFYIMATRNGMIKKSSVPQFKTTRYNKPLIAMKVKDQ**

35 OM08-17AT 100.0% 100.0%  **IPVHKLAEIRWKELGQHVSQIVSIDEDEQVIDVFNEKDFSQHEAFYIMATRNGMIKKSSVPQFKTTRYNKPLIAMKVKDQ**

36 SWO 100.0% 100.0%  **IPVHKLAEIRWKELGQHVSQIVSIDEDEQVIDVFNEKDFSQHEAFYIMATRNGMIKKSSVPQFKTTRYNKPLIAMKVKDQ**

consensus/100%  **IPVHKLAEIRWKELGQHVSQIVSIDEDEQVIDVFNEKDFSQHEAFYIMATRNGMIKKSSVPQFKTTRYNKPLIAMKVKDQ**

consensus/90%  **IPVHKLAEIRWKELGQHVSQIVSIDEDEQVIDVFNEKDFSQHEAFYIMATRNGMIKKSSVPQFKTTRYNKPLIAMKVKDQ**

consensus/80%  **IPVHKLAEIRWKELGQHVSQIVSIDEDEQVIDVFNEKDFSQHEAFYIMATRNGMIKKSSVPQFKTTRYNKPLIAMKVKDQ**

consensus/70%  **IPVHKLAEIRWKELGQHVSQIVSIDEDEQVIDVFNEKDFSQHEAFYIMATRNGMIKKSSVPQFKTTRYNKPLIAMKVKDQ**

cov pid **641**  **: . . . . 7 . .** **720**

1 29AM 100.0% 100.0%  **DEVINIMRVESDQLITVITHKGMSLTYSTNELSDTGLRAAGVKSINLKDEDFVVMTQMINHSESVMMATQRGAIKHIGFK**

2 TRPF4 100.0% 100.0%  **DEVINIMRVESDQLITVITHKGMSLTYSTNELSDTGLRAAGVKSINLKDEDFVVMTQMINHSESVMMATQRGAIKHIGFK**

3 P912 100.0% 100.0%  **DEVINIMRVESDQLITVITHKGMSLTYSTNELSDTGLRAAGVKSINLKDEDFVVMTQMINHSESVMMATQRGAIKHIGFK**

4 NGS-ED-1001 92.5% 100.0%  **DEVINIMRVESDQLITVITHKGMSLTYSTNELSDTGLRAAGVKSINLKDEDFVVMTQMINHSESVMMATQRGAIKHIGFK**

5 1DB1 92.5% 100.0%  **DEVINIMRVESDQLITVITHKGMSLTYSTNELSDTGLRAAGVKSINLKDEDFVVMTQMINHSESVMMATQRGAIKHIGFK**

6 MGYG-HGUT-02301 92.5% 100.0%  **DEVINIMRVESDQLITVITHKGMSLTYSTNELSDTGLRAAGVKSINLKDEDFVVMTQMINHSESVMMATQRGAIKHIGFK**

7 IIF4SW-P1 100.0% 100.0%  **DEVINIMRVESDQLITVITHKGMSLTYSTNELSDTGLRAAGVKSINLKDEDFVVMTQMINHSESVMMATQRGAIKHIGFK**

8 19428wF1_P912 92.5% 100.0%  **DEVINIMRVESDQLITVITHKGMSLTYSTNELSDTGLRAAGVKSINLKDEDFVVMTQMINHSESVMMATQRGAIKHIGFK**

9 R5992 92.5% 100.0%  **DEVINIMRVESDQLITVITHKGMSLTYSTNELSDTGLRAAGVKSINLKDEDFVVMTQMINHSESVMMATQRGAIKHIGFK**

10 R5990 92.5% 100.0%  **DEVINIMRVESDQLITVITHKGMSLTYSTNELSDTGLRAAGVKSINLKDEDFVVMTQMINHSESVMMATQRGAIKHIGFK**

11 R5991 92.5% 100.0%  **DEVINIMRVESDQLITVITHKGMSLTYSTNELSDTGLRAAGVKSINLKDEDFVVMTQMINHSESVMMATQRGAIKHIGFK**

12 R6028 92.5% 99.9%  **DEVINIMRVESDQLITVITHKGMSLTYSTNELSDTGLRAAGVKSINLKDEDFVVMTQMINHSESVMMATQRGAIKHIGFK**

13 R6027 92.5% 99.9%  **DEVINIMRVESDQLITVITHKGMSLTYSTNELSDTGLRAAGVKSINLKDEDFVVMTQMINHSESVMMATQRGAIKHIGFK**

14 R6024 100.0% 99.9%  **DEVINIMRVESDQLITVITHKGMSLTYSTNELSDTGLRAAGVKSINLKDEDFVVMTQMINHSESVMMATQRGAIKHIGFK**

15 L2_057 100.0% 100.0%  **DEVINIMRVESDQLITVITHKGMSLTYSTNELSDTGLRAAGVKSINLKDEDFVVMTQMINHSESVMMATQRGAIKHIGFK**

16 Cap100.1 100.0% 100.0%  **DEVINIMRVESDQLITVITHKGMSLTYSTNELSDTGLRAAGVKSINLKDEDFVVMTQMINHSESVMMATQRGAIKHIGFK**

17 Ani-LG-057 100.0% 100.0%  **DEVINIMRVESDQLITVITHKGMSLTYSTNELSDTGLRAAGVKSINLKDEDFVVMTQMINHSESVMMATQRGAIKHIGFK**

18 Cap9.2 92.5% 100.0%  **DEVINIMRVESDQLITVITHKGMSLTYSTNELSDTGLRAAGVKSINLKDEDFVVMTQMINHSESVMMATQRGAIKHIGFK**

19 Cap10.1 92.5% 100.0%  **DEVINIMRVESDQLITVITHKGMSLTYSTNELSDTGLRAAGVKSINLKDEDFVVMTQMINHSESVMMATQRGAIKHIGFK**

20 Cap9.1 100.0% 100.0%  **DEVINIMRVESDQLITVITHKGMSLTYSTNELSDTGLRAAGVKSINLKDEDFVVMTQMINHSESVMMATQRGAIKHIGFK**

21 Cap10.2 100.0% 100.0%  **DEVINIMRVESDQLITVITHKGMSLTYSTNELSDTGLRAAGVKSINLKDEDFVVMTQMINHSESVMMATQRGAIKHIGFK**

22 acrok 100.0% 100.0%  **DEVINIMRVESDQLITVITHKGMSLTYSTNELSDTGLRAAGVKSINLKDEDFVVMTQMINHSESVMMATQRGAIKHIGFK**

23 acroj 92.5% 100.0%  **DEVINIMRVESDQLITVITHKGMSLTYSTNELSDTGLRAAGVKSINLKDEDFVVMTQMINHSESVMMATQRGAIKHIGFK**

24 acror 92.5% 100.0%  **DEVINIMRVESDQLITVITHKGMSLTYSTNELSDTGLRAAGVKSINLKDEDFVVMTQMINHSESVMMATQRGAIKHIGFK**

25 NJ6 100.0% 100.0%  **DEVINIMRVESDQLITVITHKGMSLTYSTNELSDTGLRAAGVKSINLKDEDFVVMTQMINHSESVMMATQRGAIKHIGFK**

26 EYE_450 100.0% 100.0%  **DEVINIMRVESDQLITVITHKGMSLTYSTNELSDTGLRAAGVKSINLKDEDFVVMTQMINHSESVMMATQRGAIKHIGFK**

27 EYE_117 92.5% 100.0%  **DEVINIMRVESDQLITVITHKGMSLTYSTNELSDTGLRAAGVKSINLKDEDFVVMTQMINHSESVMMATQRGAIKHIGFK**

28 EYE_410 92.5% 100.0%  **DEVINIMRVESDQLITVITHKGMSLTYSTNELSDTGLRAAGVKSINLKDEDFVVMTQMINHSESVMMATQRGAIKHIGFK**

29 EYE_411 92.5% 100.0%  **DEVINIMRVESDQLITVITHKGMSLTYSTNELSDTGLRAAGVKSINLKDEDFVVMTQMINHSESVMMATQRGAIKHIGFK**

30 MERTA18 92.5% 100.0%  **DEVINIMRVESDQLITVITHKGMSLTYSTNELSDTGLRAAGVKSINLKDEDFVVMTQMINHSESVMMATQRGAIKHIGFK**

31 FAIRING19B-1.2 100.0% 100.0%  **DEVINIMRVESDQLITVITHKGMSLTYSTNELSDTGLRAAGVKSINLKDEDFVVMTQMINHSESVMMATQRGAIKHIGFK**

32 FDAARGOS_151 92.5% 99.7%  **DEVINIMRVESDQLITVITHKGMSLTYSTNELSDTGLRAAGVKSINLKDEDFVVMTQMINHSESVMMATQRGAIKHIGFK**

33 SNUC5989 100.0% 100.0%  **DEVINIMRVESDQLITVITHKGMSLTYSTNELSDTGLRAAGVKSINLKDEDFVVMTQMINHSESVMMATQRGAIKHIGFK**

34 SNUC3412 100.0% 100.0%  **DEVINIMRVESDQLITVITHKGMSLTYSTNELSDTGLRAAGVKSINLKDEDFVVMTQMINHSESVMMATQRGAIKHIGFK**

35 OM08-17AT 100.0% 100.0%  **DEVINIMRVESDQLITVITHKGMSLTYSTNELSDTGLRAAGVKSINLKDEDFVVMTQMINHSESVMMATQRGAIKHIGFK**

36 SWO 100.0% 100.0%  **DEVINIMRVESDQLITVITHKGMSLTYSTNELSDTGLRAAGVKSINLKDEDFVVMTQMINHSESVMMATQRGAIKHIGFK**

consensus/100%  **DEVINIMRVESDQLITVITHKGMSLTYSTNELSDTGLRAAGVKSINLKDEDFVVMTQMINHSESVMMATQRGAIKHIGFK**

consensus/90%  **DEVINIMRVESDQLITVITHKGMSLTYSTNELSDTGLRAAGVKSINLKDEDFVVMTQMINHSESVMMATQRGAIKHIGFK**

consensus/80%  **DEVINIMRVESDQLITVITHKGMSLTYSTNELSDTGLRAAGVKSINLKDEDFVVMTQMINHSESVMMATQRGAIKHIGFK**

consensus/70%  **DEVINIMRVESDQLITVITHKGMSLTYSTNELSDTGLRAAGVKSINLKDEDFVVMTQMINHSESVMMATQRGAIKHIGFK**

cov pid **721**  **. . : . . . . 8** **800**

1 29AM 100.0% 100.0%  **VLQEAKRAQRGITLLKELKKAPHRIVAADVVRHNYTTYTLYSDNNQESGEIASIHKSEQYTNGSFIVDIDDFGEVKGMYL**

2 TRPF4 100.0% 100.0%  **VLQEAKRAQRGITLLKELKKAPHRIVAADVVRHNYTTYTLYSDNNQESGEIASIHKSEQYTNGSFIVDIDDFGEVKGMYL**

3 P912 100.0% 100.0%  **VLQEAKRAQRGITLLKELKKAPHRIVAADVVRHNYTTYTLYSDNNQESGEIASIHKSEQYTNGSFIVDIDDFGEVKGMYL**
[truncated: 111,727 more chars]
